# ﻿A new start? Revision of the genera *Anauchen*, *Bensonella*, *Gyliotrachela* and *Hypselostoma* (Gastropoda, Eupulmonata, Hypselostomatidae) of Southeast Asia with description of 46 new species

**DOI:** 10.3897/zookeys.1235.145281

**Published:** 2025-04-23

**Authors:** Vukašin Gojšina, András Hunyadi, Chirasak Sutcharit, Piyoros Tongkerd, Kurt Auffenberg, Jozef Grego, Jaap J. Vermeulen, Alexander Reischütz, Barna Páll-Gergely

**Affiliations:** 1 Department of Morphology, Systematics and Phylogeny of Animals, University of Belgrade, Faculty of Biology, Studentski trg 16, 11000, Belgrade, Serbia University of Belgrade Belgrade Serbia; 2 Adria sétány 10G 2/5., H-1148 Budapest, Hungary Unaffiliated Budapest Hungary; 3 Animal Systematics Research Unit, Department of Biology, Faculty of Science, Chulalongkorn University, Bangkok 10330, Thailand Chulalongkorn University Bangkok Thailand; 4 Florida Museum of Natural History, University of Florida, 1659 Museum Road, Gainesville, FL, 32611, USA University of Florida Gainesville United States of America; 5 Horná Mičiná 219, SK-97401 Banská Bystrica, Slovakia Unaffiliated Banská Bystrica Slovakia; 6 JK Art and Science, Lauwerbes 8, 2318 AT Leiden, Netherlands JK Art and Science Leiden Netherlands; 7 Puechhaimgasse 52, 3580 Horn, Austria Unaffiliated Horn Austria; 8 Plant Protection Institute, HUN-REN Centre for Agricultural Research, Brunszvik u. 2., Martonvásár, 2462, Hungary Plant Protection Institute, HUN-REN Centre for Agricultural Research Martonvásár Hungary; 9 Department of Water Management and Natural Ecosystems, Albert Kázmér Faculty of Mosonmagyaróvár, Széchenyi István University, Vár 2., 9200 Mosonmagyaróvár, Hungary Széchenyi István University Mosonmagyaróvár Hungary

**Keywords:** Distribution, limestone habitats, Pupilloidea, taxonomy

## Abstract

Hypselostomatidae is a large family of terrestrial pulmonate molluscs currently represented by 284 extant species, most confined to Southeast Asia. The current system of grouping species into genera is based on the morphology of the apertural barriers and the level of last whorl detachment. However, these characters overlap, challenging generic definitions. In this paper, these characters are evaluated and a novel classification proposed for hypselostomatid species belonging to the genera *Anauchen* (17 species), *Bensonella* (36 species), *Boysidia* (7 species, in part) and *Hypselostoma* (85 species). We assigned all species belonging to the genera *Bensonella* and *Hypselostoma* into two and four species groups respectively, which are characterised by combinations of morphological traits. Altogether 46 new species are described, seven species in *Anauchen*: *A.crassus* Gojšina, Hunyadi & Páll-Gergely, **sp. nov.**, *A.evanidus* Gojšina & Páll-Gergely, **sp. nov.**, *A.grandiportus* Gojšina, Grego & Páll-Gergely, **sp. nov.**, *A.obesus* Gojšina, Hunyadi & Páll-Gergely, **sp. nov.**, *A.picasso* Gojšina & Páll-Gergely, **sp. nov.**, *A.turritus* Gojšina, Hunyadi & Páll-Gergely, **sp. nov.**, *A.jokaii* Gojšina & Páll-Gergely, **sp. nov.**; 19 species in *Bensonella*: *B.alycaeus* Gojšina & Páll-Gergely, **sp. nov.**, *B.cardiostoma* Gojšina, Vermeulen & Páll-Gergely, **sp. nov.**, *B.cristatissima* Gojšina, Hunyadi & Páll-Gergely, **sp. nov.**, *B.dha* Gojšina, Hunyadi & Páll-Gergely, **sp. nov.**, *B.dracula* Gojšina, Hunyadi & Páll-Gergely, **sp. nov.**, *B.exploda* Gojšina, Hunyadi & Páll-Gergely, **sp. nov.**, *B.fracta* Gojšina, Hunyadi & Páll-Gergely, **sp. nov.**, *B.microdentata* Gojšina & Páll-Gergely, **sp. nov.**, *B.mitochondria* Gojšina, Vermeulen & Páll-Gergely, **sp. nov.**, *B.mirabilis* Gojšina, Hunyadi & Páll-Gergely, **sp. nov.**, *B.montawa* Gojšina, Hunyadi & Páll-Gergely, **sp. nov.**, *B.multidentata* Gojšina, A. Reischütz & Páll-Gergely, **sp. nov.**, *B.nitens* Gojšina & Páll-Gergely, **sp. nov.**, *B.obex* Gojšina, Hunyadi & Páll-Gergely, **sp. nov.**, *B.perfecta* Gojšina & Páll-Gergely, **sp. nov.**, *B.sericata* Gojšina & Páll-Gergely, **sp. nov.**, *B.serrata* Gojšina, Hunyadi & Páll-Gergely, **sp. nov.**, *B.spelaea* Gojšina, Grego & Páll-Gergely, **sp. nov.**, *B.spinosa* Gojšina, Hunyadi & Páll-Gergely, **sp. nov.**; 20 species in *Hypselostoma*: *H.aquila* Gojšina, Hunyadi & Páll-Gergely, **sp. nov.**, *H.bubalus* Gojšina, Hunyadi & Páll-Gergely, **sp. nov.**, *H.circumcarinatum* Gojšina, Auffenberg & Páll-Gergely, **sp. nov.**, *H.coriaceum* Gojšina & Páll-Gergely, **sp. nov.**, *H.aenigma* Gojšina, Grego & Páll-Gergely, **sp. nov.**, *H.fortunatum* Gojšina, Hunyadi & Páll-Gergely, **sp. nov.**, *H.fungus* Gojšina, Hunyadi & Páll-Gergely, **sp. nov.**, *H.geckophilum* Gojšina, Hunyadi & Páll-Gergely, **sp. nov.**, *H.iunior* Gojšina & Páll-Gergely, **sp. nov.**, *H.ophis* Gojšina, Hunyadi & Páll-Gergely, **sp. nov.**, *H.platybasis* Gojšina, Hunyadi & Páll-Gergely, **sp. nov.**, *H.populare* Gojšina, Hunyadi & Páll-Gergely, **sp. nov.**, *H.sculpturatum* Gojšina, Hunyadi & Páll-Gergely, **sp. nov.**, *H.similare* Gojšina, Hunyadi & Páll-Gergely, **sp. nov.**, *H.sorormajor* Gojšina, Hunyadi & Páll-Gergely, **sp. nov.**, *H.sororminor* Gojšina, Hunyadi & Páll-Gergely, **sp. nov.**, *H.torta* Gojšina, Auffenberg & Páll-Gergely, **sp. nov.**, *H.vesovici* Gojšina & Páll-Gergely, **sp. nov.**, *H.vicinum* Gojšina, Auffenberg & Páll-Gergely, **sp. nov.**, *H.vujici* Gojšina & Páll-Gergely, **sp. nov.** One replacement name is proposed: *H.tertiusfrater* Gojšina & Páll-Gergely, **nom. nov.** pro *Boysidiasalpinx* F. G. Thompson & Dance, 1983, non *Hypselostomasalpinx* (van Benthem Jutting, 1961) (originally described as *Gyliotrachela*). *Gyliotrachela* and *Antroapiculus* are both treated as junior synonyms of *Hypselostoma*. An additional 28 species and subspecies are reassigned to the synonymies of other taxa.

## ﻿﻿Introduction

Hypselostomatidae is a large family of terrestrial pulmonate microsnails (shell size less than 5 mm) that occurs mostly in Southeast Asia (including the former Indochina, Indonesia, and the Philippines) and China (Schileyko 1998). The distribution of the family further extends westwards across India to Pakistan, with a much-reduced diversity (Pokryszko et al. 2009; Páll-Gergely and White 2023). Eastwards and southwards, the distribution of the family reaches Japan (two species) and Australia (one species) (Pilsbry 1908; Schileyko 1998; Páll-Gergely and White 2023). Representatives are usually restricted to limestone habitats and many species are narrow-range endemics. The family is currently represented by 284 species, which are divided into 15 genera (MolluscaBase eds. 2024). The most species-rich genus is *Angustopila* Jochum, Slapnik & Páll-Gergely, 2014 (Páll-Gergely et al. 2023) with 53 species. The typical shell shape of hypselostomatids is conical, conical-ovoid, or depressed conical, however, the last whorl is often detached from the penultimate (Hoekstra and Schilthuizen 2011; Chen et al. 2022), altering the shell shape drastically. Besides the shell shape, the species recognition is largely based on the number and arrangement of the apertural barriers.

Early study of hypselostomatids originated in the middle 19^th^ and beginning of 20^th^ century when the first genera and species were described (Pfeiffer 1849; Benson 1856; Benson 1860; Ancey 1881; Möllendorff 1890; Bavay and Dautzenberg 1909; Pilsbry 1917). The first genus described within the family was *Boysia* L. Pfeiffer, 1849 (type species *Tomogeresboysii* L. Pfeiffer, 1846). *Tanystoma* W. H. Benson, 1856 (type species *Tanystomatubiferum* W. H. Benson, 1856) was next described, a junior homonym of *Tanystoma* Motschulsky, 1845 (Coleoptera, Carabidae) and *Hypselostoma* W. H. Benson, 1856 was given as a replacement name (Benson 1856a, b). The genus *Systenostoma* Bavay & Dautzenberg, 1909 (type species Helix (Systenostoma) pauperrima Bavay & Dautzenberg, 1909) (not *Systenostoma* Marsson, 1887, Bryozoa) was later described and replaced by the name *Tonkinospira* Jochum, Slapnik & Páll-Gergely, 2014 (Jochum et al. 2014). *Gyliauchen* Pilsbry, 1917 (type species *Hypselostomahungerfordianum* Moellendorff, 1891) was erected in the new classification proposed by Pilsbry (1917), only to become a junior homonym of *Gyliauchen* Nicoll, 1915 (Platyhelminthes, Trematodes), and *Gyliotrachela* Tomlin, 1930 was proposed as a replacement name (Tomlin 1930). Van Benthem Jutting provided the first comprehensive data and described several species from Malaysia and the bordering regions between Vietnam and Cambodia (van Benthem Jutting 1950, 1961, 1962). A major acceleration in the description of hypselostomatid species occurred in the late 20^th^ and early 21^st^ century, when numerous papers were published describing mainly species from China and Thailand (e.g., Chen et al. 1995, 1999; Panha 1998a, b, c; Thompson and Upatham 1997; Panha and Burch 2002a, b, c, d, e; Luo et al. 2000; Burch et al. 2003; Panha et al. 2004; Guo et al. 2006).

Several genera were also misclassified within or outside this family, only to be subsequently reassigned, i.e., *Campolaemus* Pilsbry, 1892 and *Clostophis* W. H. Benson, 1860. *Campolaemus* was previously placed in the Hypselostomatidae by Schileyko (1998), only to be reassigned to the Streptaxidae by Páll-Gergely (2020) due to biogeography and greater morphological similarity to some African species. *Clostophis* was for some time considered a monotypic genus of diplommatinids (Benson 1860; Kobelt 1902; Egorov 2013). Panha and Burch (2002a) described the genus *Montapiculus*, to which *M.proboscidea* Panha & Burch, 2002 was assigned. Páll-Gergely et al. (2020) have shown that *M.proboscidea* is better assigned to *Clostophis* when *Montapiculus* was synonymised with *Clostophis*, which is included in the Hypselostomatidae. Nowadays, the number of hypselostomatid species is increasing even faster due to new discoveries throughout Southeast Asia (e.g., Páll-Gergely et al. 2015, 2019, 2022, 2023; Inkhavilay et al. 2016; Tanmuangpak and Dumrongrojwattana 2022; Chen 2023; Gojšina et al. 2023; Sutcharit et al. 2023; Tongkerd et al. 2024).

The work of Pilsbry (1917) introduced a classification which remained in use nowadays. His classification scheme of the “*Hypselostoma*-*Boysidia*“ group was based on two features: the appearance of the last whorl (detached or not detached from the penultimate whorl) and the appearance of the lamellae on the parietal wall (angular and parietal lamellae separated/concrescent or no angular lamella). This scheme was sufficient at the time, as only four genera (*Boysidia*, *Gyliauchen* (= *Gyliotrachela*), *Hypselostoma*, and *Paraboysidia*) were known and the distinctions were clear. Pilsbry noted that these characters can overlap and the classification may require alteration as new species and genera are described. As predicted, the characters overlapped a great deal, making it very difficult to develop a new classification scheme that would overcome the confusion. This also led to the description of species that were provisionally assigned to various genera because it was not clear which genus was appropriate (e.g., Páll-Gergely 2023a; Gojšina et al. 2023). *Anauchen*, whose type species is characterised by the last whorl attached to the penultimate and by the absence of an angular lamella, presently contains species with a detached last whorl (see Vermeulen et al. 2019) and even signs of a bifid angulo-parietal lamella (e.g., see Burch and Panha 2002 for *A.utaithaniensis* Panha, 2002). The type species of *Anauchen* (*Boysidiagereti* Bavay & Dautzenberg, 1904, now considered a synonym of *Anauchenrochebruni* (Mabille, 1887) (Páll-Gergely 2023a)), is relatively large (~ 3 mm) and thus strikingly different from some very small species tentatively placed in this genus (e.g., *A.kozari* Páll-Gergely, 2023 and *A.palawanensis* Gojšina, Schilthuizen & Páll-Gergely, 2023 which are both < 1.5 mm high).

The genital apparatus of hypselostomatids has only been described for a few species. First to deal with this subject was Berry (1963), who described the genital apparatus of *H.depressispira* van Benthem Jutting, 1949. After Berry’s work, the genital system was described for several other species such as *H.cultura* Tanmuangpak & Dumrongrojwattana, 2022, *H.diarmaidi* Panha & Burch, 2003, *H.srakeoense* Panha & Burch, 2004, and *Boysidiasalwiniana* (Theobald, 1870) (Saenkamon et al. 2022; Tanmuangpak and Dumrongrojwattana 2022; Tongkerd et al. 2024). Recently, the genitalia and radula have been described for four *Aulacospira* species from Thailand: *A.pluangtong* Panha & Burch, 2004, *A.tekavongae* Dumrongrojwattana & Tanmuangpak, 2020, *A.nutadhirai* Dumrongrojwattana & Tanmuangpak, 2020 and *A.vanwalleghemi* Dumrongrojwattana & Tanmuangpak, 2020 (Dumrongrojwattana and Tanmuangpak 2020). Lastly, genitalia were described for *Angustopilaerawanica* Páll-Gergely & Dumrongrojwattana, 2023, which is also the smallest hypselostomatid with known genital anatomy (Páll-Gergely et al. 2023).

Phylogenetic analyses on the Hypselostomatidae are scarce (Tongkerd et al. 2004; Hoekstra and Schilthuizen 2011). Unfortunately, there are no works that include many species from different genera, nor *Gastrocopta* Wollaston, 1878, which is why the relations of analysed species/genera in this group remain unclear.

The number of species of the group has increased dramatically since Pilsbry’s (1917) classification (38 species of *Anauchen*, *Boysidia* [including the subgenus Bensonella], *Gyliauchen*, *Hypselostoma*, *Paraboysidia* to 155 today), but the species remain classified according to the principles of Pilsbry (1917) even though it was well-known that his classification was faulty. For example, some representatives of the genera *Gyliotrachela* and *Hypselostoma* are very similar in basic shell morphology, the only character separating them being the appearance of the angulo-parietal lamellae, mentioned by Pilsbry as the key character to distinguish the two genera (i.e., *Gyliotracheladepressispira*/*Hypselostomatubiferum* and *Gyliotrachelaluctans* van Benthem Jutting, 1950/*Hypselostomapiconis* van Benthem Jutting, 1949).

*Paraboysidia* Pilsbry, 1917 was introduced as a genus that differs from *Bensonella* Pilsbry & Vanatta, 1900 in having non-hooked apertural barriers. However, this character has been proven incorrect, as the type species of *Bensonella* (*Pupaplicidens* W. H. Benson, 1849) has non-hooked barriers, so *Paraboysidia* is now treated as a junior synonym of *Bensonella* (see Páll-Gergely and White 2023).

In this work we have revised all species of the genera *Anauchen*, *Bensonella*, *Gyliotrachela*, and *Hypselostoma* from Southeast Asia except for the species inhabiting the Philippines which are not treated in this work and will be revised separately. *Gyliotrachela* and *Antroapiculus* are both considered junior synonyms of *Hypselostoma*. In total, 46 species are described as new to science and 28 new synonyms are proposed. We also provide a new classification method within the family by sorting them into several morphologically similar groups, utilising the overall shell shape, shell surface texture, and the arrangement of the apertural barriers. In previous studies (e.g., Páll-Gergely et al. 2016; Jirapatrasilp et al. 2024), it has been shown that several morphological characters of a hypselostomatid shell such as the surface sculpture, level of the last whorl detachment and ascension/ descension or number of apertural barriers can vary within a single species. Hence, the intraspecific variability and reliability of all shell characters are discussed here in detail.

## ﻿﻿Materials and methods

**Figure 1.**
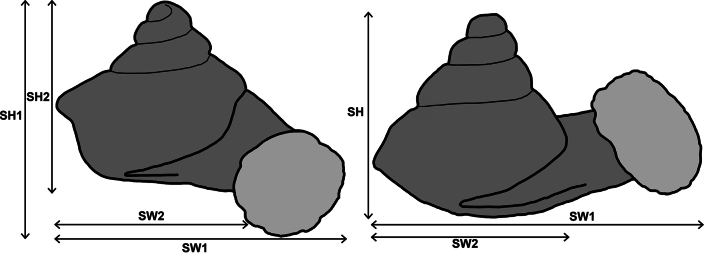
Basic measurements of a hypselostomatid shell.

Type specimens of all species of interest were examined when the information provided in the original descriptions were not sufficient, or images/drawings were poor. New species have been compared to all similar congeners from Southeast Asia. Shells were photographed via a Nikon SMZ25 digital microscope with Nikon Nis-Elements software or directly observed without coating under a low vacuum using a Hitachi FlexSEM 1000 II scanning electron microscope in the Plant Protection Institute of the HUN-REN Centre for Agricultural Research (Martonvásár, Hungary). Shells were measured with a Nikon DS-L3 control unit. Shell width or height is greatly influenced if the last whorl is strongly detached. In these cases, shell width/height was measured twice: including the last whorl near the aperture (Fig. 1, SW1/SH1) and excluding it (Fig. 1, SW2/SH2). Distribution maps were made with Google Earth Pro. Literature citations were checked via MolluscaBase (MolluscaBase eds. 2024).

### ﻿﻿Examined characters

#### ﻿﻿Shell shape

**Figure 2.**
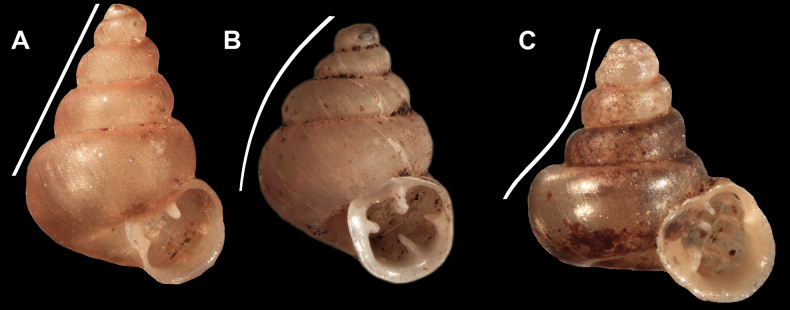
Shell shape variability of hypselostomatid genera (not to scale) **A** conical **B** conical-ovoid **C** concave-conical.

Examined species could be separated into three main shell shapes: conical, conical-ovoid, and concave-conical. We have defined these main shell shapes as follows: i) Conical – an imaginary straight line contacts all whorls (Fig. 2A); ii) Conical-ovoid – an imaginary curved, convex line contacts all whorls (Fig. 2B); iii) Concave-conical – an imaginary curved, concave line contacts all whorls (Fig. 2C).

#### ﻿﻿Surface sculpture of the teleoconch

**Figure 3.**
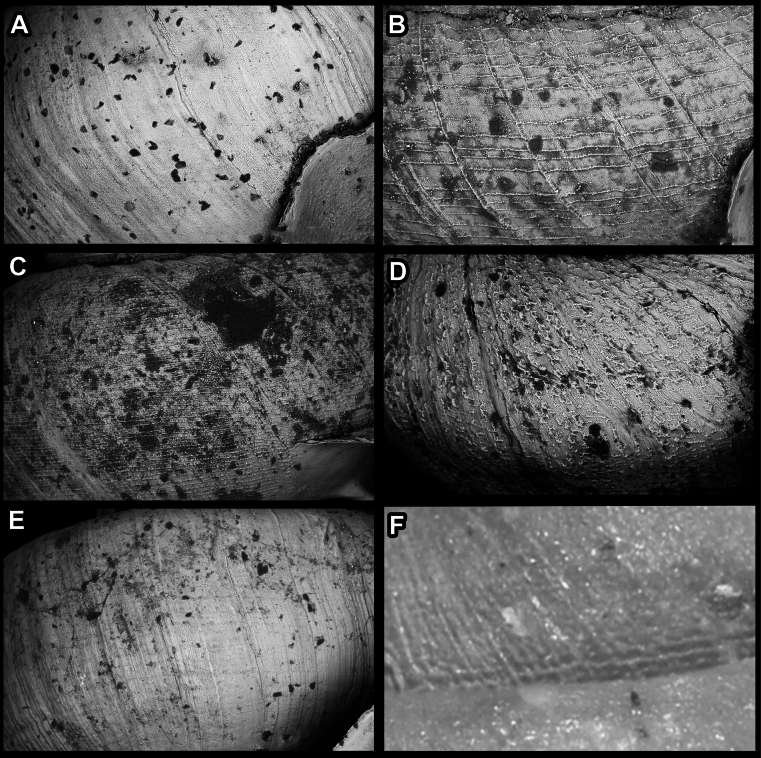
Shell surface variability of hypselostomatid genera **A** finely dimpled, pasty surface **B** strong, raised spiral striae occasionally crossed by radial growth lines **C** very fine, dense spiral striation occasionally crossed by radial growth lines **D** rough, wrinkled (sandpaper) surface **E** weak, non-raised spiral striae crossed by radial lines **F** reticulated, consisting of rib-like radial growth lines which are crossed by equally strong spiral striae.

Teleoconch surface sculpture varies a great deal among hypselostomatid species. We distinguished several different types: i) finely dimpled, pasty surface (reminiscent of floury dough) without spiral striation and with only radial growth lines (Fig. 3A); ii) strong, raised spiral striae which are sometimes crossed by radial growth lines or whitish radial streaks (Fig. 3B); iii) very fine, dense spiral striation occasionally crossed by some radial growth lines (Fig. 3C); iv) rough, reminiscent of the surface of sandpaper (Fig. 3D); v) weak spiral striae usually crossed by radial lines (Fig. 3E); vi) reticulated surface, consisting of rib-like radial growth lines crossed by equally strong spiral striae (Fig. 3F).

#### ﻿﻿Surface sculpture of the protoconch

**Figure 4.**
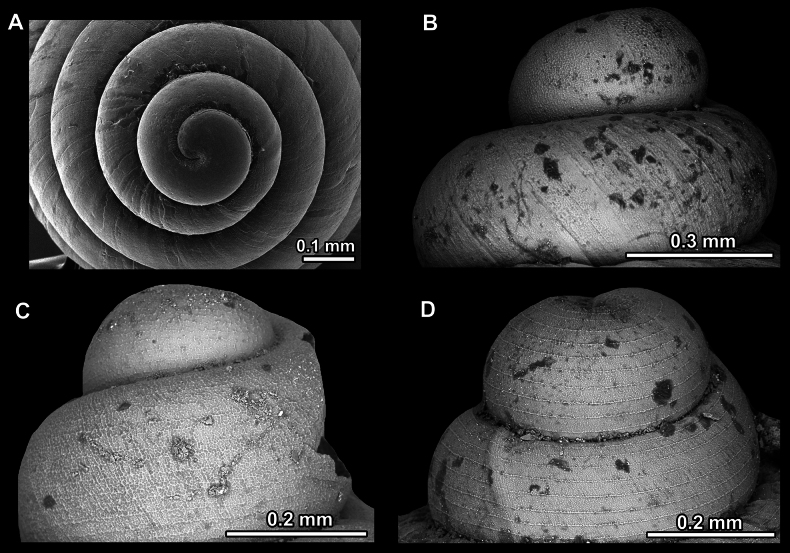
Protoconch surface sculpture variability of hypselostomatid genera **A** smooth **B** pitted, no spiralling pattern **C** pitted, spiral pattern **D** spirally striated.

Several types of protoconch surface sculpture exists: i) smooth (Fig. 4A); ii) pitted (dimpled, finely or roughly), pasty, showing no spiral pattern (Fig. 4B); iii) dimpled (finely or roughly), pasty, showing a spiral pattern, but no clear spiral striae (Fig. 4C); iv) spirally striated (Fig. 4D).

#### ﻿﻿Last whorl

**Figure 5.**
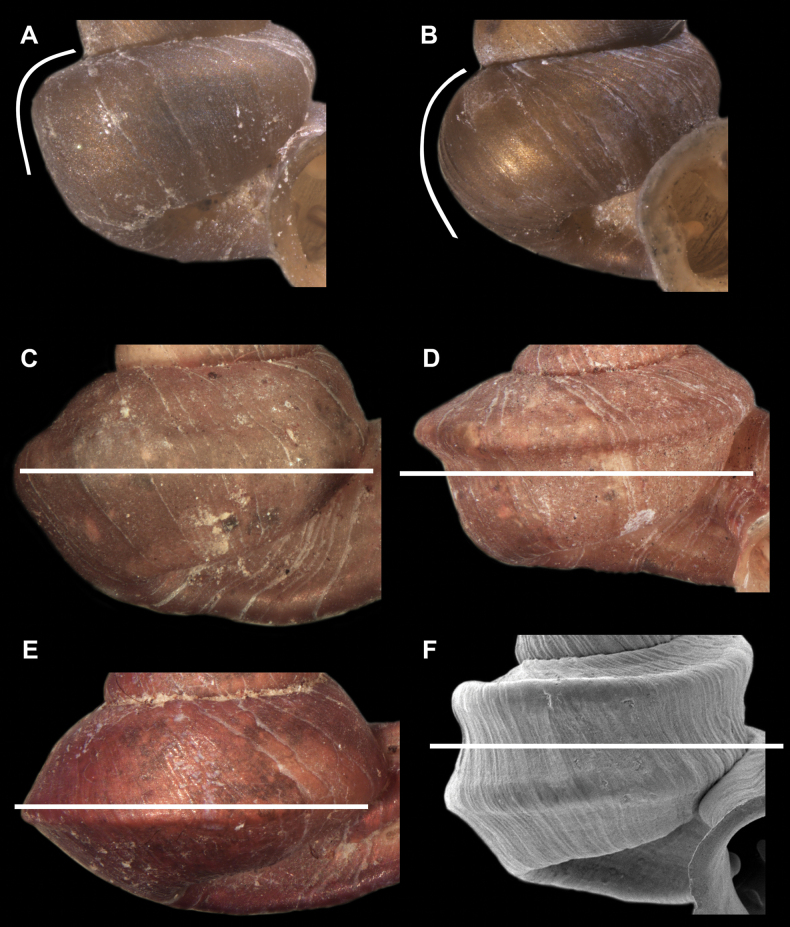
Appearance of the last whorl **A** shouldered **B** rounded **C** keeled at the centre of the periphery **D** keeled above the centre of the periphery **E** keeled below the centre of the periphery **F** double keeled.

We were able to distinguish the following traits of the last whorl: i) last whorl rounded (Fig. 5B); ii) last whorl keeled (a ridge occurs near the centre of the periphery); keel positioned at centre of the periphery, above or below it (Fig. 5C, D, E). Only one species, *H.phupaman* Panha & Burch, 2002 has two keels (one above and one below the periphery), so that the last whorl is double-keeled (Fig. 5F); iii) last whorl shouldered (at top of the last whorl or bottom of the last whorl) (Fig. 5A).

**Figure 6.**
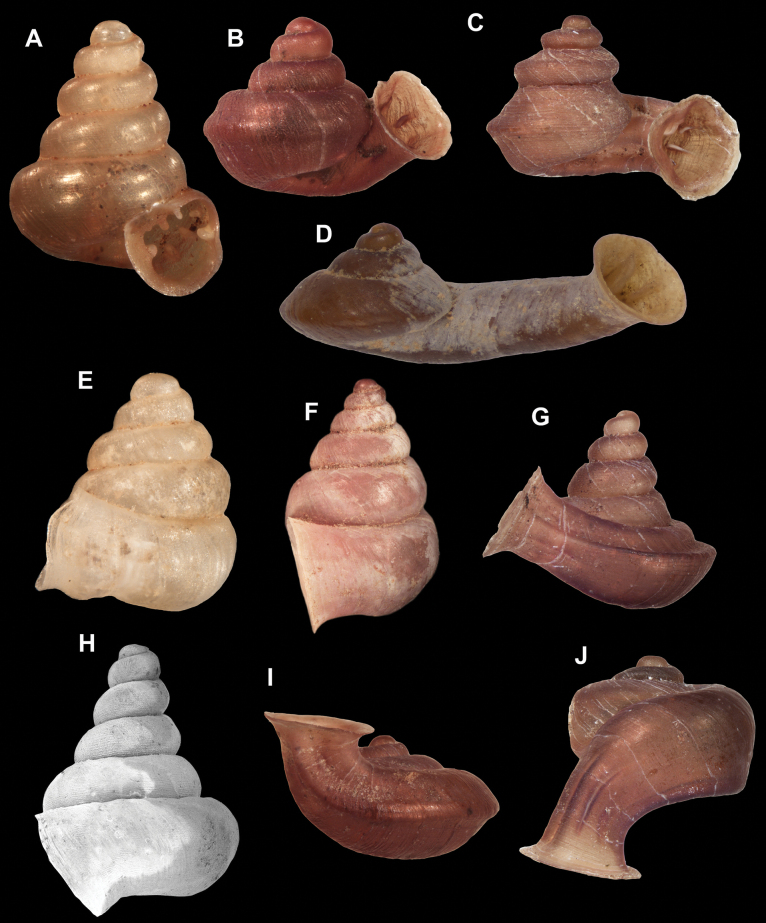
**A–D** detachment of last whorl **E–J** direction of the last whorl **A** last whorl adnate to the penultimate **B** last whorl slightly detached **C** last whorl moderately detached **D** last whorl strongly detached **E** last whorl slightly ascending **F** last whorl slightly descending **G** last whorl moderately ascending **H** last whorl moderately descending **I** last whorl strongly ascending **J** last whorl strongly descending.

Since the last whorl is often detached from the penultimate (especially in *Hypselostoma*), we distinguished four categories of detachment level in adult specimens: i) adnate (not detached) (Fig. 6A); ii) slightly detached (Fig. 6B); iii) moderately detached (Fig. 6C); iv) strongly detached (Fig. 6D).

Regarding the descending/ascending last whorl, we were able to distinguish three categories: i) slightly ascending/descending (0–30 degrees compared to the shell axis) (Fig. 6E, F); ii) moderately ascending/descending (31–59 degrees compared to the shell axis) (Fig. 6G, H); iii) strongly ascending/descending (60–90 degrees compared to the shell axis) (Fig. 6I, J).

#### ﻿﻿Apertural barriers on the parietal side

**Figure 7.**
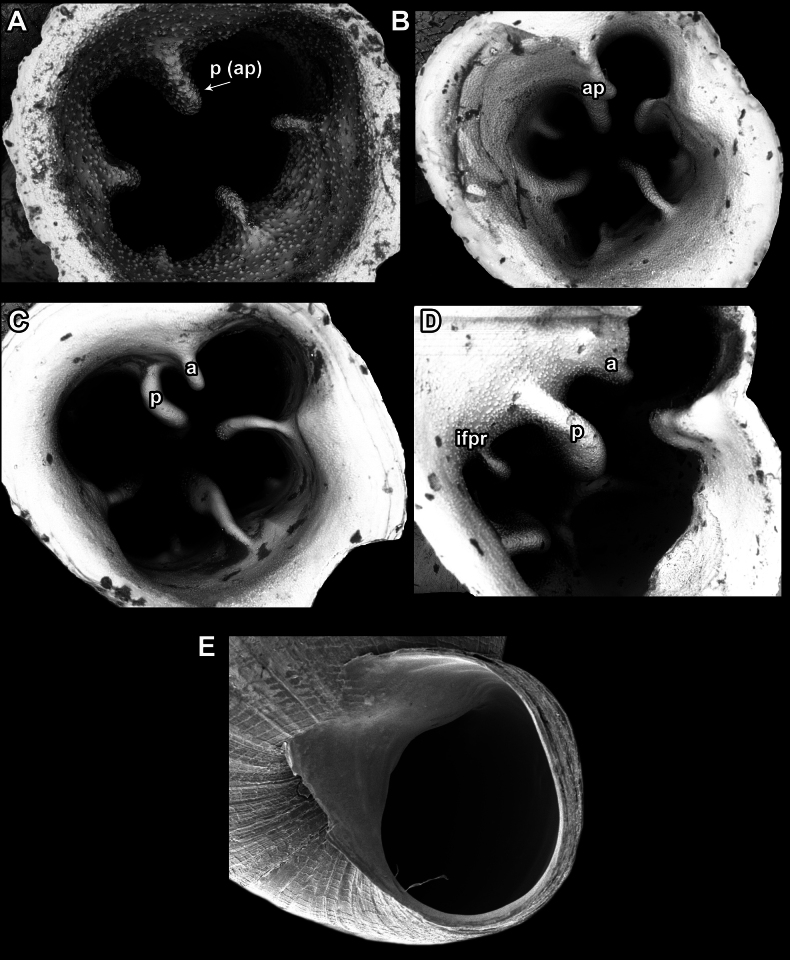
Appearance of the barriers on the parietal side **A** single, parietal lamella **B** single, bifid angulo-parietal lamella **C** angular and parietal lamellae separate **D** three lamellae on the parietal side (angular, parietal, infraparietal) **E** aperture without barriers. Abbreviations: a, angular lamella; ap, angulo parietal lamella; ifpr, infraparietal lamella; p, parietal lamella.

Apertural barriers (especially on the parietal side) are very important for distinguishing between species or species groups. These barriers can appear as follows: i) single, parietal lamella on the parietal side (Fig. 7A); ii) single, bifid angulo-parietal lamella (with more or less clearly discernible angular and parietal part of the fused lamellae) (Fig. 7B); iii) two separate lamellae on the parietal side (angular and parietal) (Fig. 7C); iv) three lamellae on the parietal side (angular, parietal and infraparietal) and an additional palatal tubercle situated at the edge of the palatal lip (Fig. 7D); v) aperture without barriers (Fig. 7E).

**Figure 8.**
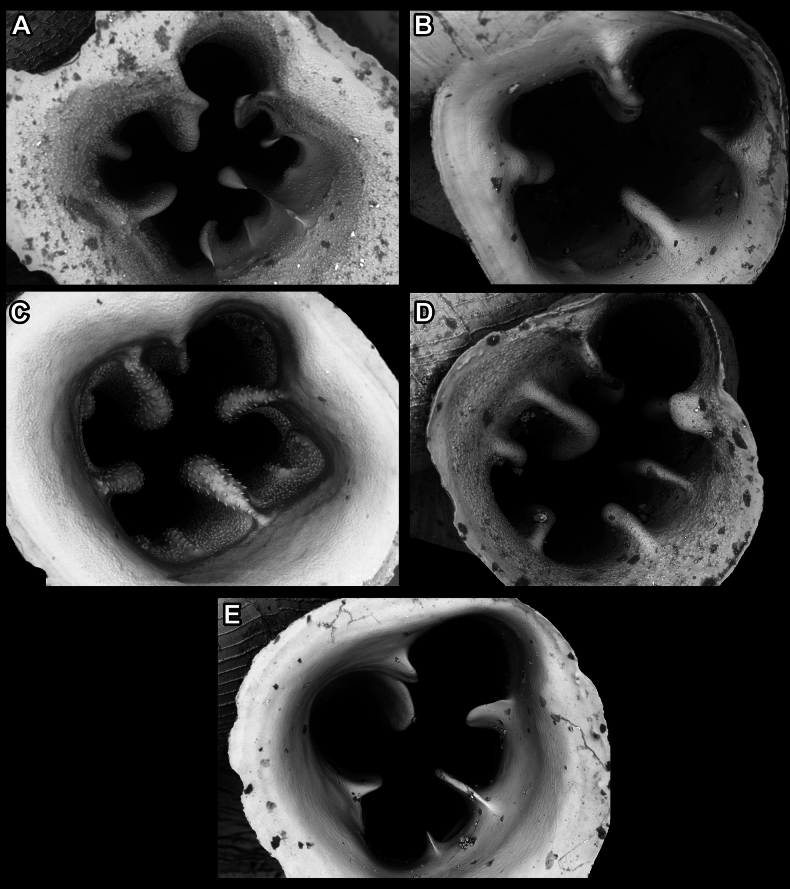
Shape and surface sculpture of the apertural barriers **A** hooked barriers **B** blunt barriers **C** surface equipped with spines **D** surface granulated **E** surface smooth.

Barriers can also vary in shape and surface sculpture: i) hooked, pointing outside or inside the aperture (Fig. 8A); ii) blunt (Fig. 8B); iii) equipped with spines (spiniferous) (roughly or finely) (Fig. 8C); iv) granulated (roughly of finely) (Fig. 8D); v) smooth, without spines or granules (Fig. 8E).

#### ﻿﻿Nomenclature of the barriers on the palatal and columellar sides

**Figure 9.**
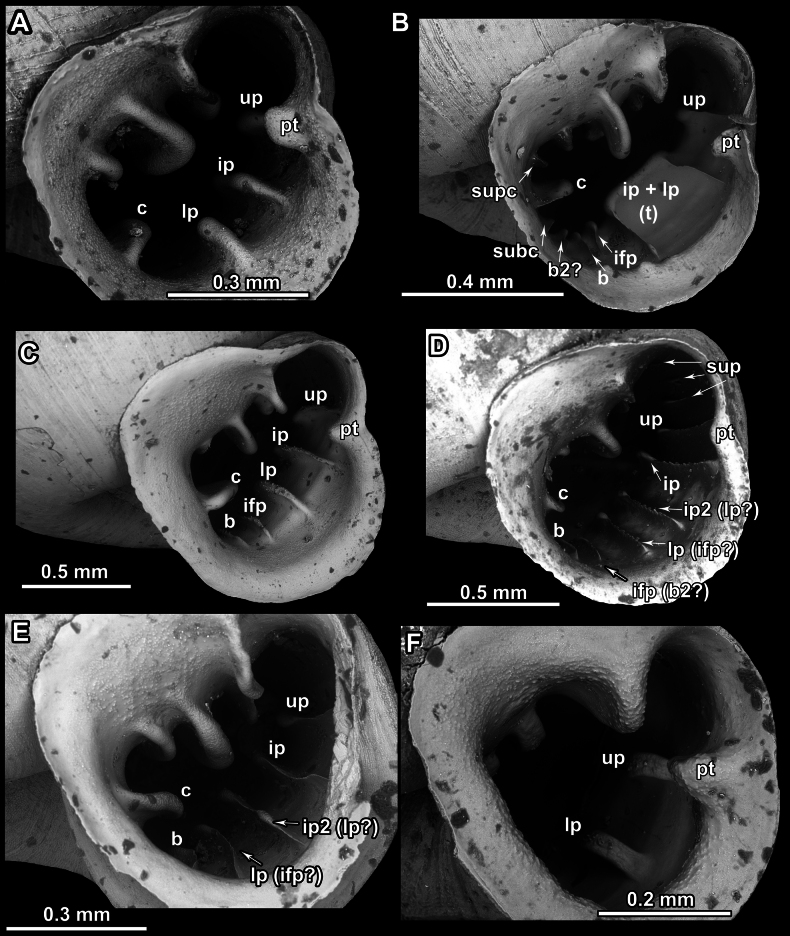
Apertural barriers on the palatal and columellar sides (example on *Bensonella*). Abbreviations: b, basal plica; c, columellar lamella; ifp, infrapalatal plica; ip, interpalatal plica; lp, lower palatal plica; pt, palatal tubercle; subc; subcolumellar lamella; sup, suprapalatal plica; supc, supracolumellar lamella; t, transversal plica; up, upper palatal plica.

We hypothesise the homology of palatal barriers in hypselostomatids (which can be especially challenging in *Bensonella*) based on their topology. The upper palatal plica is located behind the palatal tubercle or immediately above it at the beginning of the sinulus. The interpalatal plica is located slightly below the palatal tubercle. The lower palatal plica is located roughly halfway between the palatal tubercle and the basal plica. The suprapalatal plicae are always located deeper in the sinulus. The infrapalatal plica is located below the lower palatal and situated next to the basal. The columellar lamella is a strong barrier in the middle of the columellar side. Small barriers right above or below it are called supracolumellar and subcolumellar respectively. Basal plica is between the subcolumellar (sometimes columellar) and infrapalatal (sometimes lower palatal) (Fig. 9).

### ﻿Taxonomic treatment

#### ﻿﻿Genus and species-group level

None of the hypselostomatid genera we have reviewed in this paper can be diagnosed on the basis of a single, unambiguous character. Therefore, the diagnoses of the genera must be based on the combination of morphological characters which, however, do not always allow a clear assignment of a species to a genus. These characters are all listed under the genus diagnosis section. In some cases (e.g., *A.kozari*, *A.grandiportus* sp. nov., *H.aenigma* sp. nov.), a species was provisionally placed in a genus due to the unique combination of morphological characters which may require a future introduction of a separate genus.

We were able to separate species groups within the genera *Bensonella* (two species groups) and *Hypselostoma* (four species groups). In *Bensonella*, the subdivision was based on a largely consistent shell shape and apertural barrier arrangement (three barriers on the parietal side and a palatal tubercle). In *Hypselostoma*, we relied on results of Tongkerd et al. (2004). These authors have shown that morphologically very different species can be very closely related, but that in this case, they share the same (or very similar) surface sculpture. Consequently, our subdivision of *Hypselostoma* was based on similarities in the shell surface sculpture.

#### ﻿﻿Species level

During the examination of empty shells, we determined which characters were useful for species distinction. Species are regarded as distinct if the surface sculpture is strikingly different (e.g., granulated vs raised spirally striated). Variability in the strength (e.g., more or less thick spiral striae) of the shell surface sculpture (present in some widespread species such as *H.khaowongense*, *H.crossei*) was not considered for species separation unless it was coupled with other differences in shell morphology. On the other hand, species with different types of spiral striation (e.g., very dense vs coarse (see under *Hypselostomaiunior* sp. nov. for its comparison with *H.panhai*), raised vs non-raised) were considered distinct on the species level.

If otherwise similar species had concrescent or separate angular and parietal lamellae, we regarded them as distinct. However, the level of fusion (completely fused vs bifid) was not important for species distinction (i.e., there was an overlap within one population, see under *A.utaithaniensis*). The appearance of the minor barriers (between the main ones) was variable within populations and was not considered important if they did not differ clearly and consistently in number or arrangement between populations. The strength of apertural barriers was not considered informative for species distinction if it was not coupled with a different arrangement or morphology of barriers.

Whether barriers are blunt or hooked is important for species identification. If two species were virtually identical but one had blunt and other hooked barriers, they were always considered distinct (even though in these cases, we were usually able to find additional differences in shell morphology). The surface of the barriers (and even peristome) can be smooth, granulated or spiniferous which is also considered informative (e.g., see under *B.spinosa* sp. nov.).

The width of the umbilicus was considered important only if it was clearly and consistently different between the populations (i.e., there was no overlap between populations).

Appearance of the last whorl (rounded, keeled, shouldered) was considered important for the separation of species. However, this was never the only character on which we based our conclusions (i.e., usually coupled with other shell traits if the differences of the last whorl are not striking). This is especially true for e.g., *H.khaowongense* in which we have found that the level of the shoulderness of the last whorl can vary (i.e., shouldered part can be more or less sharp (distinct)) and sometimes it can even resemble a keel.

#### ﻿﻿Abbreviations

**AH** aperture height

**AW** aperture width

**SH** shell height

**SW** shell width

#### ﻿﻿Depository acronyms

**ANSP** Academy of Natural Sciences (Philadelphia, USA)

**CUMZ** Chulalongkorn University Museum of Zoology (Bangkok, Thailand)

**HA** Collection of András Hunyadi (Budapest, Hungary)

**IZCAS** National Zoological Museum of China, Institute of Zoology, Chinese Academy of Sciences (Beijing, China)

**IEBR** Institute of Ecology and Biological Resources (Hanoi, Vietnam)

**JG** Collection of Jozef Grego (Banská Bystrica, Slovakia)

**JJV** Collection of Jaap J. Vermeulen (Leiden, The Netherlands)

**MNHN** Muséum National d’Histoire Naturelle (Paris, France)

**NHMUK** The Natural History Museum (London, UK)

**NHMW** Naturhistorisches Museum Wien (Vienna, Austria)

**NMNS** National Museum of Natural Science (Taichung, Taiwan)

**PGB** Collection of Barna Páll-Gergely (Budapest, Hungary)

**REI** Collection of Alexander & Peter L. Reischütz (Horn, Austria)

**RMNH** National Museum of Natural History, formerly Rijksmuseum van Natuurlijke Historie (Leiden, The Netherlands)

**SMF** Senckenberg Froschunginstitut und Naturmuseum (Frankfurt am Main, Germany)

**SMNH** Swedish Museum of Natural History [Naturhistoriska Riksmuseet] (Stockholm, Sweden)

**UF** Florida Museum of Natural History, University of Florida (Gainesville, USA)

**UMZC** University Museum of Zoology (Cambridge, United Kingdom)

**VG** Collection of Vukašin Gojšina (Belgrade, Serbia)

## ﻿Results

By examining the entire literature and studying all species within the genera, we were able to draw several important conclusions:

1. Currently, it seems that there are no clear genus-specific shell characters which allow unambiguous assignment of a species to a genus. However, there are some combinations of characters which allow at least provisional placement (these combinations are listed below every genus section in Results).
2. Species pairs with similar shell appearance, but one with fused and one with separated angular and parietal lamellae, are not assigned to different genera (e.g., *Hypselostomatubiferum* and *Gyliotracheladepressispira*; *Hypselostomapiconis* and *Gyliotrachelaluctans*). Consequently, this trait used by Pilsbry (1917) should be treated with caution and cannot serve as a key trait to distinguish genera.
3. Normally, there are four or five main apertural barriers, which are always present and are not significantly variable within a population. These barriers are the angular, parietal (sometimes as angulo-parietal or only parietal), upper palatal, lower palatal and columellar. In addition, there are also sometimes minor barriers that lie between the main ones. These barriers are highly variable in number and appearance (thickness, length, height) between genera or even within a genus (especially *Hypselostoma*) and have little taxonomic significance. Also, their nomenclature is far from clear. It is not obvious whether a small lamella usually present exactly between the columellar and the parietal lamella is actually the infraparietal or the supracolumellar lamella. If barriers are very numerous and variable (e.g., *H.depressispira*, *H.crossei*), it is impossible to confidently assign a name to each.
4. The degree of separation of the last whorl from the penultimate whorl is very variable in some species (e.g., *H.crossei*, *H.hungerfordianum*, *H.khaowongense*) and is not a clear feature for differentiating genera or similar species.
5. The homology of some barriers is important, but not unambiguous. For example, it is not clear whether a single lamella in *Anauchen* is homologous only to the parietal lamella or to both the parietal and angular lamellae. Since we have found some specimens of *Anauchen* with an additional small, pointed, angular part on the parietal lamella (instead of only one single lamella, see e.g., Fig. 35E, F), we consider the latter to be more likely. In addition, the genus *Bensonella* has a pronounced strong palatal tubercle on the palatal lip, which makes the genus relatively easy to recognise. There are also some similar thickenings in other genera (for *Hypselostoma* see Gojšina et al. (2023), for *Anauchen* see Páll-Gergely 2023a), which could be homologous with the mentioned tubercle. Finally, sometimes homology of the barriers is virtually unknown (see under *Anauchengrandiportus* sp. nov.).

### ﻿﻿Taxonomic part

#### ﻿﻿Family Hypselostomatidae

Taxon classificationAnimaliaStylommatophoraHypselostomatidae

Zilch, 1959

210A87A6-28B7-5980-882D-8C1D5113D8AD

##### Remarks.

Hypselostomatidae were originally introduced as a subfamily of Chondrinidae (Zilch 1959). In the same work, the subfamily Aulacospirinae Zilch, 1959 was erected, later synonymised by Schileyko (1998). Bouchet et al. (2017) treated Hypselostomatinae as a subfamily of Gastrocoptidae, while some authors used Hypselostomatidae (Páll-Gergely 2023a, b; Páll-Gergely and White 2023; Gojšina et al. 2022) Vertiginidae (Panha 1998a; Tanmuangpak and Dumrongrojwattana 2022) or Pupillidae (Burch and Panha 2002; Panha and Burch 2002a) to classify these representatives.

#### ﻿﻿Genus Anauchen

Taxon classificationAnimaliaStylommatophoraHypselostomatidae

Pilsbry, 1917

5A58BAED-0891-523E-B1CA-23BA19BFFE3B

Anauchen Pilsbry, 1917: 188.

##### Diagnosis.

This genus is characterised by the combination of characters: i) a single lamella which is formed by a fusion of angular and parietal lamellae (it can appear bifid in some species, see below); ii) last whorl adnate to, or only very slightly detached from the penultimate; iii) shell surface pasty (dimpled) or finely spirally striated (never rough, sandpaper-like surface); iv) surface of apertural barriers smooth.

##### Remarks.

The following species possess some exceptions from the aforementioned combination of diagnostic characters for the genus: *Anauchenkozari* and *Anauchenevanidus* sp. nov. Both of them have rough (granulated) surfaces of the apertural barriers. Furthermore, *A.kozari* is much smaller (height 1.25 mm, width 1.3 mm) than all other congeners and has a palatal tubercle (typical for many *Bensonella* species). However, both species are retained in Anauchen for reasons listed under “Remarks” section for these species.

#### ﻿ Anauchen angthongensis

Taxon classificationAnimaliaStylommatophoraHypselostomatidae

Panha, 2002

616869CC-0FEC-5DDA-B3D8-4C8E1BC517E3

Figs 10A , 11 , 37

Anauchen angthongense Panha in Burch & Panha, 2002: 245–246, fig. 4.
Anauchen angthongense — Panha and Burch 2008: 48, fig. 44; Dumrongrojwattana and Wongkamhaeng 2024: 323, fig. 7.

##### Type material examined.

**Thailand** • 1 paratype; from the type locality; S. Panha leg.; SMF 3311444.

##### Type locality.

“Angthong National Park, Suratthani Province Thailand, 9°38'6"N, 99°41′16"E, 60 meters elevation…”.

##### Differential diagnosis.

This species is similar to many congeners, mostly based on the appearance of the apertural barriers. For differences, see under the following species: *A.crassus* sp. nov., *A.huaykhakang*, *A.informis*, *A.obesus* sp. nov., *A.utaithaniensis* and *A.banmiensis*.

**Figure 10.**
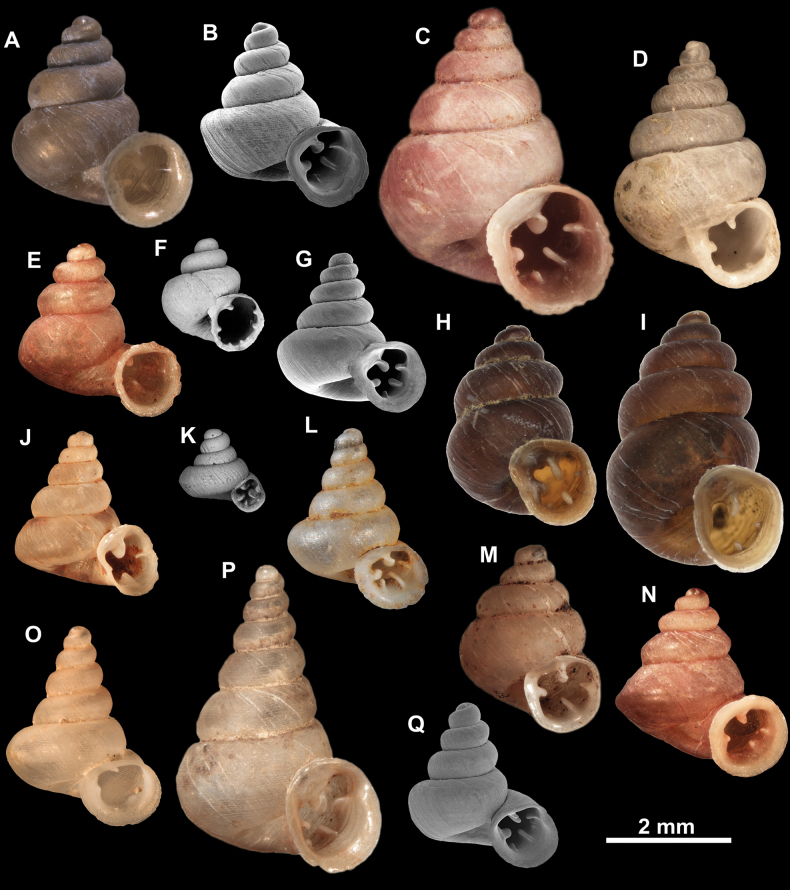
Synoptic view of the species belonging to the genus *Anauchen***A***A.angthongensis***B***A.banmiensis***C***A.crassus* sp. nov. **D***A.eotvosi***E***A.evanidus* sp. nov. **F***A.grandiportus* sp. nov. **G***A.huaykhakang***H***A.informisinformis***I***A.informisparcedentata***J***A.jokaii* sp. nov. **K***A.kozari***L***A.messageri***M***A.obesus* sp. nov. **N***A.picasso* sp. nov. **O***A.rochebruni***P***A.turritus* sp. nov. **Q***A.utaithaniensis*.

**Figure 11.**
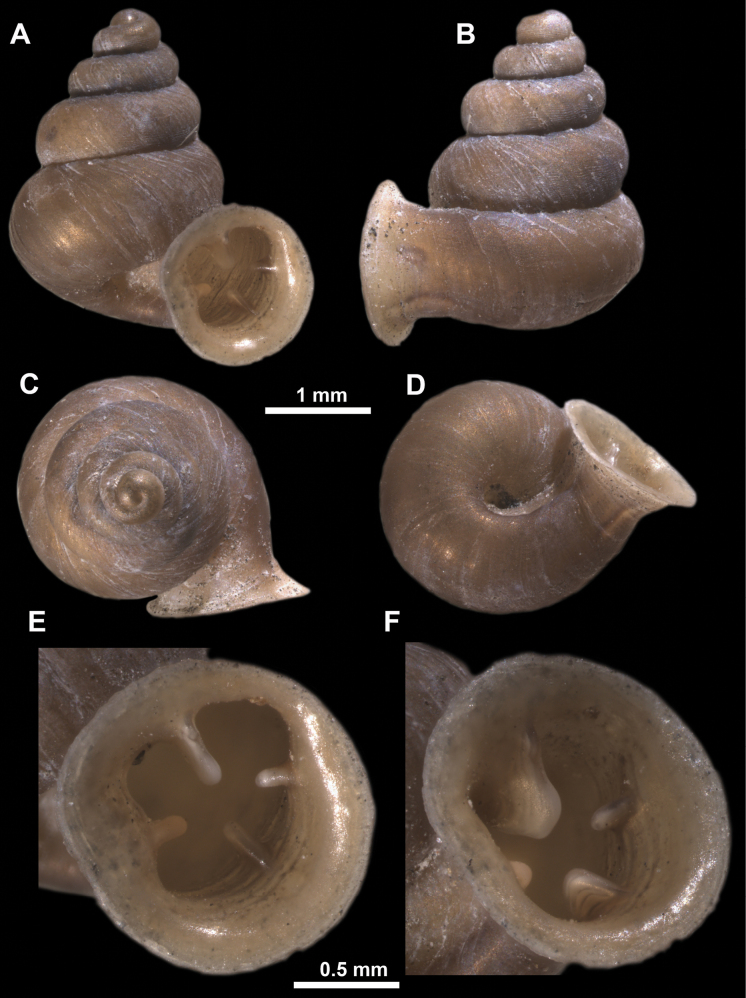
*Anauchenangthongensis*, paratype (SMF 331444) **A–D** shell **E, F** enlarged apertural views.

##### Distribution.

This species is known only from the type locality.

##### Remarks.

Basal plica can be present or absent.

#### ﻿ Anauchen banmiensis

Taxon classificationAnimaliaStylommatophoraHypselostomatidae

Panha & J. B. Burch, 2004

572693BB-1118-5749-B4C3-7128EC9939C6

Figs 10B , 12 , 37

Anauchen banmiensis Panha & Burch in Panha et al. 2004: 59–60, fig. 2.
Hypselostoma banmiensis — Panha and Burch 2008: 89–90, fig. 76; Dumrongrojwattana and Wongkamhaeng 2024: 324, fig. 8.

##### Type material examined.

**Thailand** • 1 paratype; from the type locality; S. Panha leg.; CUMZ ver.44026.

##### Type locality.

“Chang Peuag Cave Mountain, Banmi District, Lopburi Province, 14°54'10"N, 100°30'8"E, 15 meters elevation” (Thailand).

##### Differential diagnosis.

This species differs from *A.angthongensis* by its shouldered whorls (not rounded as in the latter) and more enlarged last whorl. For differences from further similar species, see under *A.crassus* sp. nov., *A.huaykhakang*, *A.informis*, and *A.utaithaniensis*.

**Figure 12.**
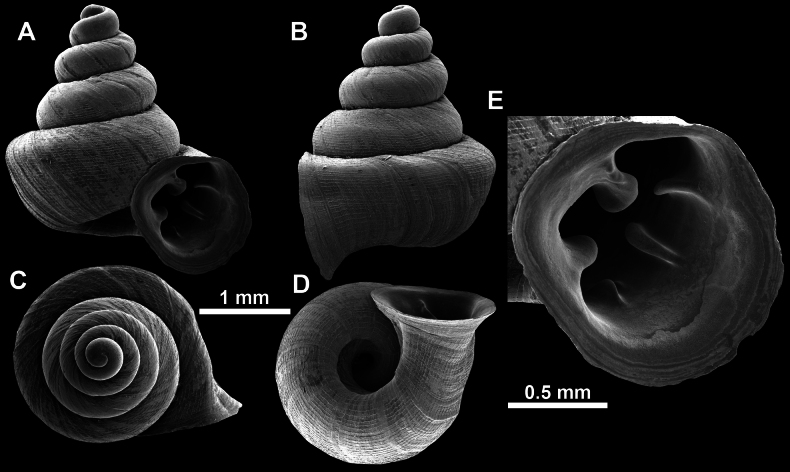
*Anauchenbanmiensis*, paratype (CUMZ ver.44026) **A–D** shell **E** enlarged apertural view.

##### Distribution.

This species is known only from the type locality.

##### Remarks.

This species was also considered a member of *Hypselostoma* (Panha and Burch 2008) but given the fact that the shell shape and microsculpture resembles some *Anauchen* species (*A.huaykhakang*, *A.utaithaniensis*), we assign this species to the latter.

#### ﻿ Anauchen crassus

Taxon classificationAnimaliaStylommatophoraHypselostomatidae

Gojšina, Hunyadi & Páll-Gergely sp. nov.

C0322615-9ACA-5868-AB79-A36EBACD07A2

https://zoobank.org/A13D15C6-380C-407C-89F0-921DC93FDA33

**Figure 13.**
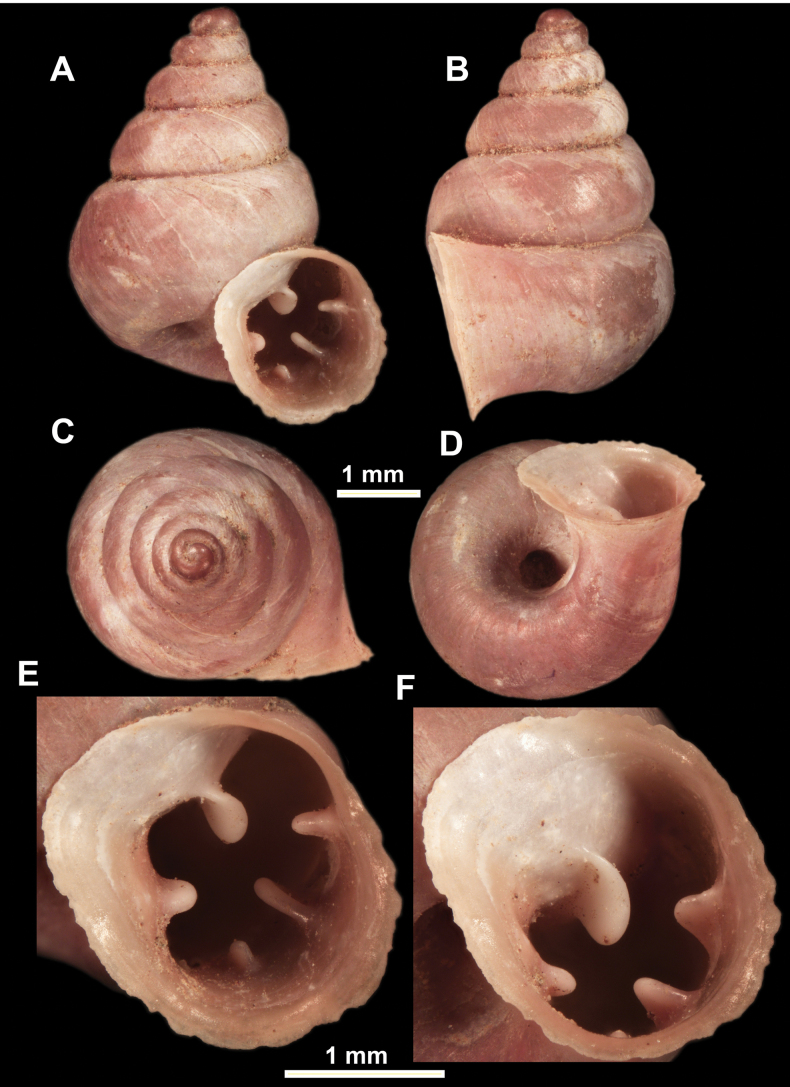
*Anauchencrassus* Gojšina, Hunyadi & Páll-Gergely, sp. nov., holotype (CUMZ 14427) **A–D** shell **E, F** enlarged apertural views.

**Figure 14.**
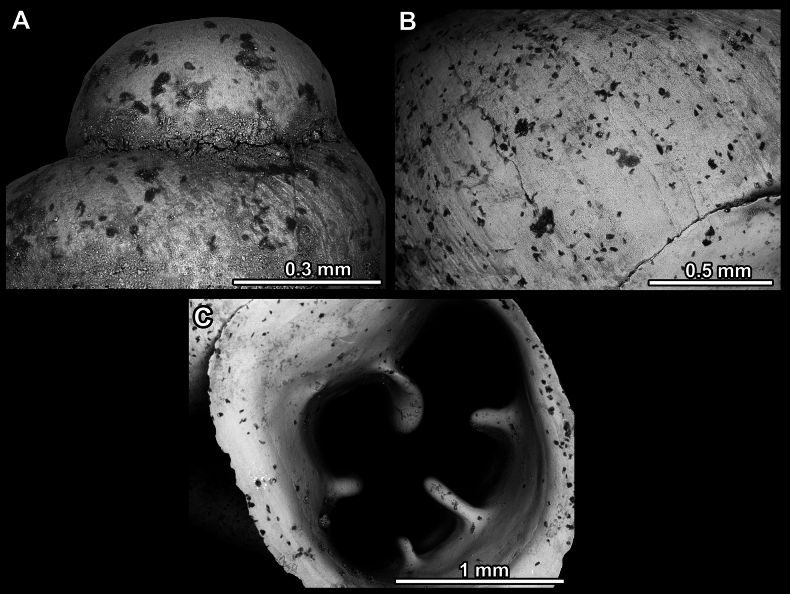
SEM imaging of *Anauchencrassus* Gojšina, Hunyadi & Páll-Gergely, sp. nov., holotype (CUMZ 14427) **A** protoconch surface **B** teleoconch surface **C** enlarged apertural view.

Figs 10C , 13 , 14 , 37

##### Type material.

***Holotype*. Thailand** • 1 empty shell (SH: 4.83 mm, SW: 3.54 mm); Ranong Province, south of Kra Buri, Tham Phra Kayang, southeastern part of the rock; 10°19.480'N, 98°45.933'E; 10 m a.s.l.; 22 Feb. 2015; A. Hunyadi leg.; CUMZ 14427. ***Paratypes*. Thailand** • 6 shells; same data as for holotype; coll. HA.

##### Additional material examined.

**Thailand** • 1 shell (damaged, not paratype); same data as for holotype; coll. HA.

##### Type locality.

Thailand, Ranong Province, south of Kra Buri, Tham Phra Kayang, southeastern part of the rock; 10°19.480'N, 98°45.933'E; 10 m a.s.l.

##### Diagnosis.

*Anauchen* species with finely dimpled (pasty) shell surface sculpture (no spiral striae), aperture adnate to last whorl, five strong apertural barriers (parietal, upper palatal, lower palatal, basal, columellar).

##### Description.

Shell conical, consisting of 5.5–6 thick-walled, regularly increasing, rounded, convex whorls separated by a moderately deep suture. All shells from the type material are somewhat weathered but colouration is dark reddish brown to pinkish, opaque. Protoconch weathered so the surface sculpture is not clear, not spirally striated, consisting of ~ 1.5–1.75 whorls, glossy and slightly darker than the rest of the shell. Teleoconch pasty, very finely radially striated, with no sign of spiral striation. Last whorl adnate to the penultimate and slightly descending near the aperture (~ 10 ° compared to the shell axis) making the aperture profile prosocline to the shell axis. Peristome continuous, leaning on the penultimate whorl at the parietal side in form of a thick and distinct callus. It is slightly paler in colour than the rest of the shell, expanded (particularly on the parietal side where it forms a distinct callus) but not reflected. Aperture equipped with five strong, high, relatively short, and thick barriers (parietal, upper palatal, lower palatal, basal, and columellar). Parietal lamella is the strongest in the aperture, blade-like, slightly oblique, its outer part curved towards the palatal side and only almost reaching the expanding peristome. Upper palatal and lower palatal plicae are similar, the lower one being stronger, more slender, higher, and positioned slightly deeper in the aperture. Basal plica small and very short, knob-like. Columellar lamella thick and horizontal, situated in the middle of the columellar wall. None of these barriers reach the expanding peristome. Surface of all apertural barriers is smooth. Sinulus wide and parabolic (due to the position of angular lamella and upper palatal plica). Umbilicus open and deep but moderately wide, measuring ~ 1/6 of the shell width. Only the penultimate whorl is visible through the umbilicus. A very short and shallow groove is present on the last ~ ¼ of the body whorl, running from the peristome towards the umbilicus.

##### Differential diagnosis.

This species is similar to *Anauchenangthongensis* Panha, 2002 from which it can be distinguished by the lack of spiral striation and much wider umbilicus. Additionally, *A.crassus* sp. nov. has a shallower suture resulting in less convex whorls than in *A.angthongensis*. *Anaucheninformis* Vermeulen, Phung & Truong, 2007 is also spirally striated, has narrower umbilicus and its whorls are more convex and less regularly increasing. *Anauchenbanmiensis* is spirally striated and has a shouldered last whorl. For differences from *A.obesus* sp. nov., see under that species. *Boysidiaphatangensis* Dumrongrojwattana & Assawawattagee, 2018 is more slender and has more whorls which are more convex, with deeper sutures. It usually also has an additional lamella in the columellar-parietal boundary region, which was not (but may be) present in *A.crassus* sp. nov. *Boysidiaphatangensis* has a bifid lamella (concrescent angular and parietal) on the parietal side while *A.crassus* sp. nov. has one blade-like lamella. Basal plica in *B.phatangensis* is seemingly more closely positioned to columellar lamella, while that of *A.crassus* sp. nov. is somewhere between the lower palatal and columellar or even closer to the lower palatal. Infraparietal lamella is absent in the new species, but present in *B.phatangensis*. Lastly, the umbilicus of *B.phatangensis* is slightly more excentrical and wider than in *A.crassus* sp. nov.

##### Measurements

**(in mm, *n* = 5).**SH = 4.83–5.39; SW = 3.54–4.04; AH = 2.15–2.24; 2.04–2.23.

##### Etymology.

The specific epithet refers to the thick (Lat. *crassus*) shell and apertural barriers of this species.

##### Distribution.

Only known from the type locality.

#### ﻿ Anauchen eotvosi

Taxon classificationAnimaliaStylommatophoraHypselostomatidae

Páll-Gergely, 2023

DFE7170F-C1AC-5C33-9646-558EB32FA710

Figs 10D , 15 , 37

Anauchen eotvosi Páll-Gergely, 2023b: 452–454, fig. 1.
Anauchen eotvosi — Tongkerd et al. 2024: 165.

##### Type material examined.

***Holotype*. Thailand-Myanmar** • Shan-Siam boundary; Woodthorpe coll.; NHMUK 1903.7.1.1227.1. ***Paratypes*. Thailand-Myanmar** •; 6, same data as for holotype; NHMUK 1903.7.1.1227.2–7.

##### Type locality.

“Shan [Shan States, Myanmar]-Siam [Thailand] Boundary”.

##### Differential diagnosis.

This species differs from its congeners by the presence of weak barriers situated close to the peristome edge. All other congeners similar in shape (e.g., *A.angthongensis*, *A.crassus* sp. nov., *A.informis*), have barriers much stronger and higher. See also under *H.iunior* sp. nov.

**Figure 15.**
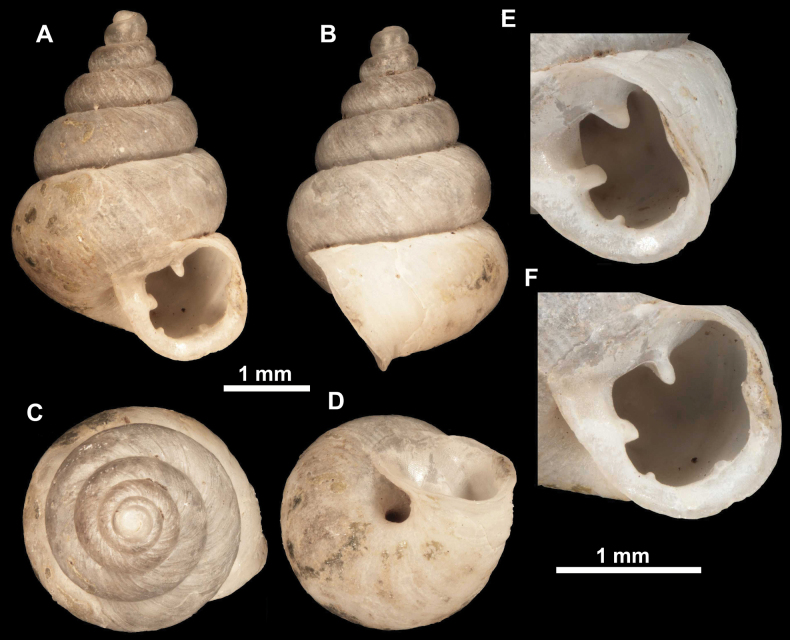
*Anaucheneotvosi*, holotype (NHMUK 1903.7.1.1227.1) **A–D** shell **E, F** enlarged apertural views (from Páll-Gergely 2023b).

##### Distribution.

This species is known only from the imprecise type locality (Shan-Siam boundary).

#### ﻿ Anauchen evanidus

Taxon classificationAnimaliaStylommatophoraHypselostomatidae

Gojšina & Páll-Gergely sp. nov.

B2B8499F-32AE-582C-AB4D-56CB0486E448

https://zoobank.org/288ECCE3-5ACC-436B-9331-13F8BC271146

**Figure 16.**
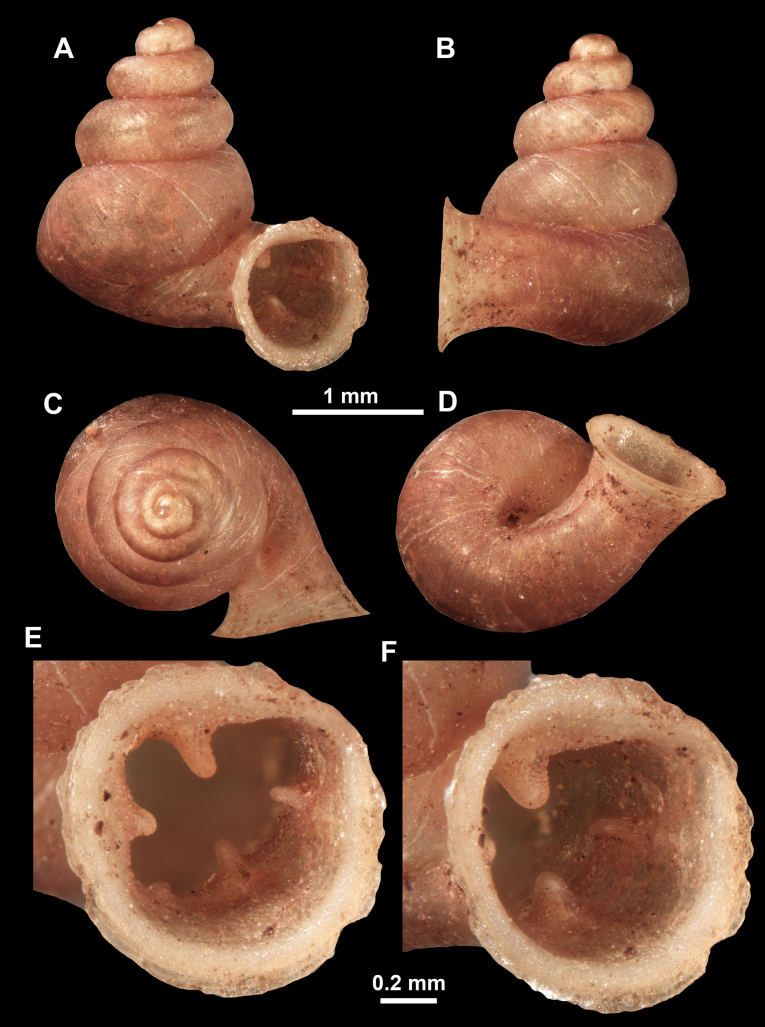
*Anauchenevanidus* Gojšina & Páll-Gergely, sp. nov., holotype (UF 530655) **A–D** shell **E, F** enlarged apertural views.

**Figure 17.**
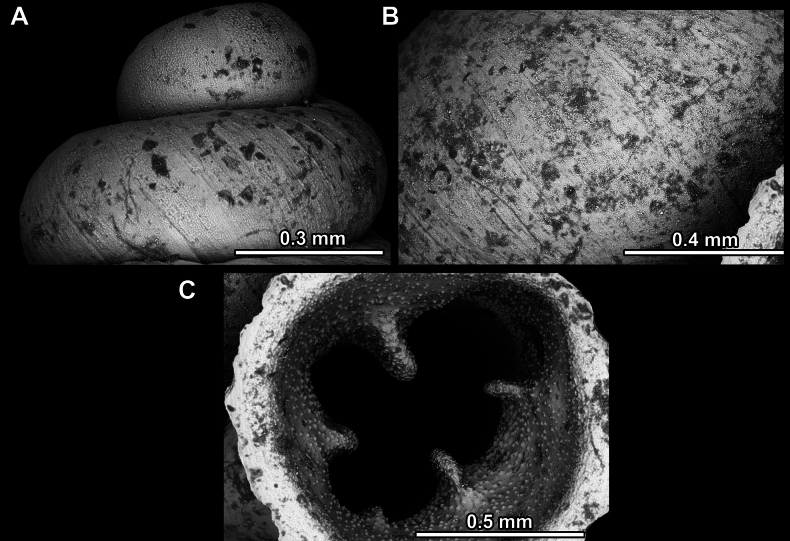
SEM imaging of *Anauchenevanidus* Gojšina & Páll-Gergely, sp. nov., holotype (UF 530655) **A** protoconch surface **B** teleoconch surface **C** enlarged apertural view.

Figs 10E , 16 , 17 , 37

##### Type material.

***Holotype*. Thailand** • 1 shell (SH: 2.57 mm, SW: 2.23 mm); Chanthaburi Province, 3.0 km W of Na Yai Am; 12.738°N, 101.881°E; 23 Apr. 1987; F.G. Thompson leg.; UF 530655. ***Paratypes*. Thailand** • 2 shells; same data as for holotype; CUMZ 14431 • 33 shells; same data as for holotype; UF 591355.

##### Additional material examined.

**Thailand** • 2 shells (1 damaged and 1 juvenile, not paratypes); same data as for holotype; UF 583732.

##### Type locality.

Thailand, Chanthaburi Province, 3.0 km W of Na Yai Am; 12.738°N, 101.881°E.

##### Diagnosis.

*Anauchen* with very slightly keeled last whorl (below the centre of the periphery) which is also detached from the penultimate. Teleoconch finely dimpled, pasty sculptured, with no spiral striae. Five or six apertural barriers present (parietal, infraparietal, upper palatal, lower palatal, basal and columellar), all strongly spiniferous.

##### Description.

Shell conical, brown, consisting of 4.5–5 convex whorls separated by a deep suture. Protoconch similar in colour as the rest of the shell, finely pitted, showing no spiralling pattern or spiral striae and consisting of 1.25–1.5 whorls. Teleoconch surface sculpture fine, pasty, without spiral striae and with several irregularly spaced whitish radial streaks which are most prominent on the last whorl. Last whorl convex but weakly keeled below the centre of the periphery, slightly detached from the penultimate near the aperture and very slightly ascending (below 5 ° compared to the shell axis). Peristome dirty white, expanded but not reflected. Aperture rounded, equally as wide as high. It is equipped with five or six simple apertural barriers (parietal, infraparietal, upper palatal, lower palatal, basal and columellar). Parietal lamella high and moderately strong, not reaching the expanding peristome. Upper and lower palatal plicae equally strong but the lower palatal is usually slightly higher. Basal plica present as a very weak, dot-like swelling situated halfway between the lower palatal plica and columellar lamella. The latter is sometimes almost as wide as long, provoking a rounded shape. The columellar wall of the aperture is weakly swollen around the columellar lamella. Infraparietal lamella present as a small swelling or completely absent. None of the apertural barriers are reaching the peristome (although the parietal lamella is the closest) and all are roughly spiniferous as well as the apertural surface. Sinulus wide and not strongly isolated from the rest of the aperture due to the relatively weak upper palatal plica. Umbilicus very narrow, measuring ~ 1/10 of the shell width. The umbilical groove is present running alongside it. This groove is the broadest near the peristome and regularly tapering towards the umbilicus, ending as a narrow canal at the last ~ ¼ of the body whorl.

##### Differential diagnosis.

This species is the most similar to *A.picasso* sp. nov. and for differences, see under that species. The new species also bears some resemblance to *Aulacospirapluangtong* Panha & Burch, 2004, but the latter has less rounded whorls, more elongated umbilicus, smooth apertural barriers (not spiniferous) and it lacks the basal plica.

##### Measurements

**(in mm, *n* = 5).**SW = 2.10–2.58; SH = 2.39–2.82; AH = 1.10–1.22; AW = 1.09–1.28.

##### Etymology.

The Latin word *evanidus* means vanishing, which refers to the quarrying of the type locality of this species.

##### Distribution.

This species is known only from the type locality.

##### Remarks.

Unlike congeners (except for *A.kozari*), this species has rough, spiniferous surface of the apertural barriers. It is retained in *Anauchen* because of the similar barrier arrangement to other species (e.g., *A.angthongensis*, *A.crassus* sp. nov., *A.huaykhakang*, *A.obesus* sp. nov.) and a single lamella on the parietal side.

#### ﻿ Anauchen grandiportus

Taxon classificationAnimaliaStylommatophoraHypselostomatidae

Gojšina, Grego & Páll-Gergely sp. nov.

29EF1077-1C8F-5F4A-8D1D-C9331002CA1F

https://zoobank.org/2512BC05-6D55-4EFE-AA32-E4D086744D1A

Figs 10F , 18 , 37

##### Type material.

***Holotype*. Laos** • 1 empty shell (SH: 1.71 mm, SW: 1.92 mm); Khammouane Province, NE foot of Mount Pha Soung, caverns among slope boulders; 17°33.108'N, 104°52.301'E; 08 Feb. 2017; J. Grego leg.; CUMZ 14428.

##### Type locality.

Laos, Khammouane Province, NE foot of Mount Pha Soung, caverns among slope boulders; 17°33.108'N, 104°52.301'E.

##### Diagnosis.

Shell conical-ovoid, teleoconch radially and not spirally striated. Last whorl not detached from the penultimate and slightly descending. Aperture large, with seven weak barriers sitting on the peristome edge. Umbilicus narrow.

##### Description.

Shell brown, conical-ovoid with deep suture and bulging whorls. Whorls 3.25, rounded, regularly increasing. Protoconch consists of slightly > 1 whorl, finely pitted, its terminal part with ~ 12 equidistant spiral striae. Teleoconch with inconspicuous, irregular growth lines and finely dimpled (pasty), spiral striation absent. Last whorl rounded, adnate to the penultimate and slightly descending near the aperture (~ 15 ° compared to the shell axis), aperture slightly prosocline to shell axis in lateral view. Aperture conspicuously large to the shell size, parietal part adnate onto penultimate whorl forming a weak callus. Peristome slightly expanded but not reflected. Altogether seven apertural barriers could be found, all being blunt, knob-like, situated on peristome edge, their homologies with traditionally recognised barriers somewhat questionable. Parietal lamella is the largest, but still weak. Suprapalatal and upper palatal plicae situated close to each other, after some distance the next plica is the lower palatal. Lower palatal, basal, subcolumellar, and columellar situated in approximately equal distance from one another, gap between columellar and parietal is approximately as large as between parietal and the suprapalatal. There is a very slight thickening next to lower palatal plica, although it is questionable whether it would develop into a barrier. Surfaces of all apertural barriers are very finely granulated. Sinulus wide but low, not strongly isolated from the rest of the aperture. Umbilicus narrow, elongate, only shows body whorl.

**Figure 18.**
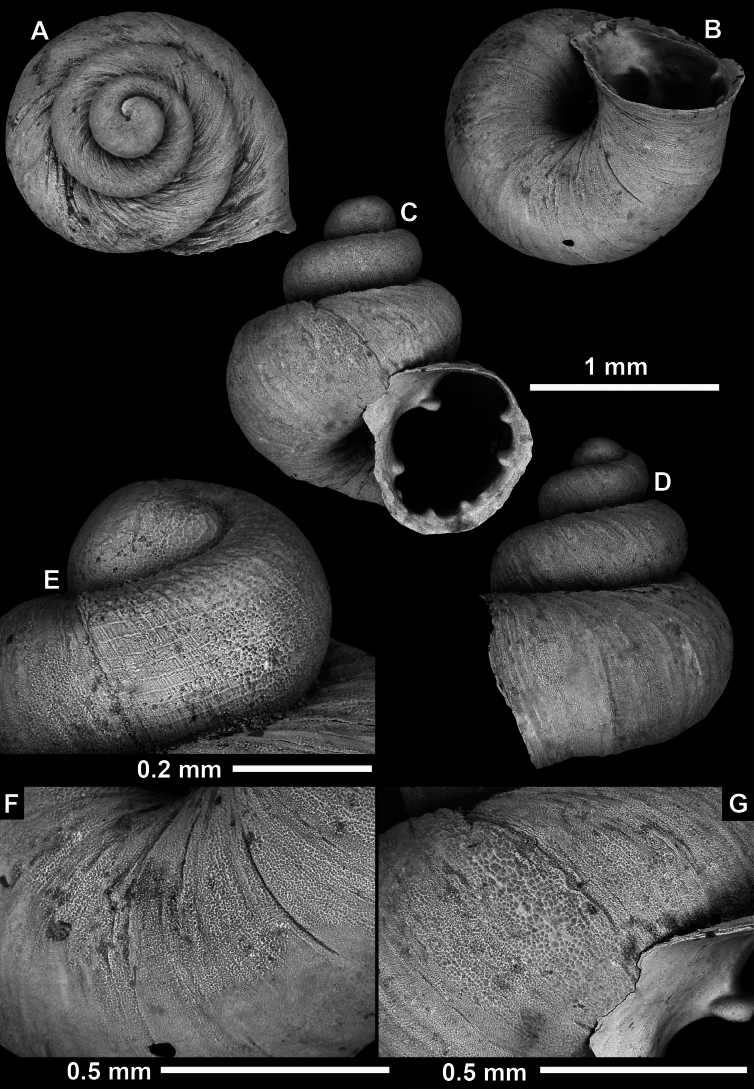
*Anauchengrandiportus* Gojšina, Grego & Páll-Gergely, sp. nov., holotype (CUMZ 14428) **A–D** shell **E** enlarged protoconch view **F, G** enlarged view of the surface of the last whorl.

##### Differential diagnosis.

*Anauchengrandiportus* sp. nov. differs from all other species assigned to *Anauchen* on the basis of the seven small, marginal denticles and the greatly enlarged aperture.

##### Measurements

**(in mm, *n* = 1).**SH = 1.92; SW = 1.71, AH = 0.96, AW = 0.87.

##### Etymology.

The specific epithet refers to the relatively large aperture when compared to the shell size.

##### Distribution.

This species is known only from the type locality.

#### ﻿ Anauchen huaykhakang

Taxon classificationAnimaliaStylommatophoraHypselostomatidae

Panha, 2002

4805BBAC-C57E-500E-8508-E7C56A4494C2

Figs 10G , 19 , 37

Anauchen huaykhakang Panha in Burch & Panha, 2002: 239–244, fig. 2.
Anauchen huaykhakang — Panha and Burch 2008: 51–52, fig. 47; Dumrongrojwattana and Wongkamhaeng 2024: 323, fig. 7.

##### Type material examined.

**Thailand** • 1 paratype; from the type locality; 1997; S. Panha leg.; CUMZ ver. 038.

##### Type locality.

“Subkao, Huaykhakang Wildlife Sanctuary, Lansak District, Utaithani Province, 15°35'7"N, 99°18'8"E, 310 meters elevation…” (Thailand).

##### Differential diagnosis.

This species differs from *A.angthongensis* by the shouldered last whorl and concave-conical shell (due to the strongly enlarged last whorl). *Anauchenbanmiensis* is much more strongly spirally striated, it last whorl is more distinctly shouldered and has a wider umbilicus with a deep groove inside it (absent in *A.huaykhakang*). For differences from *A.utaithaniensis*, see under that species.

**Figure 19.**
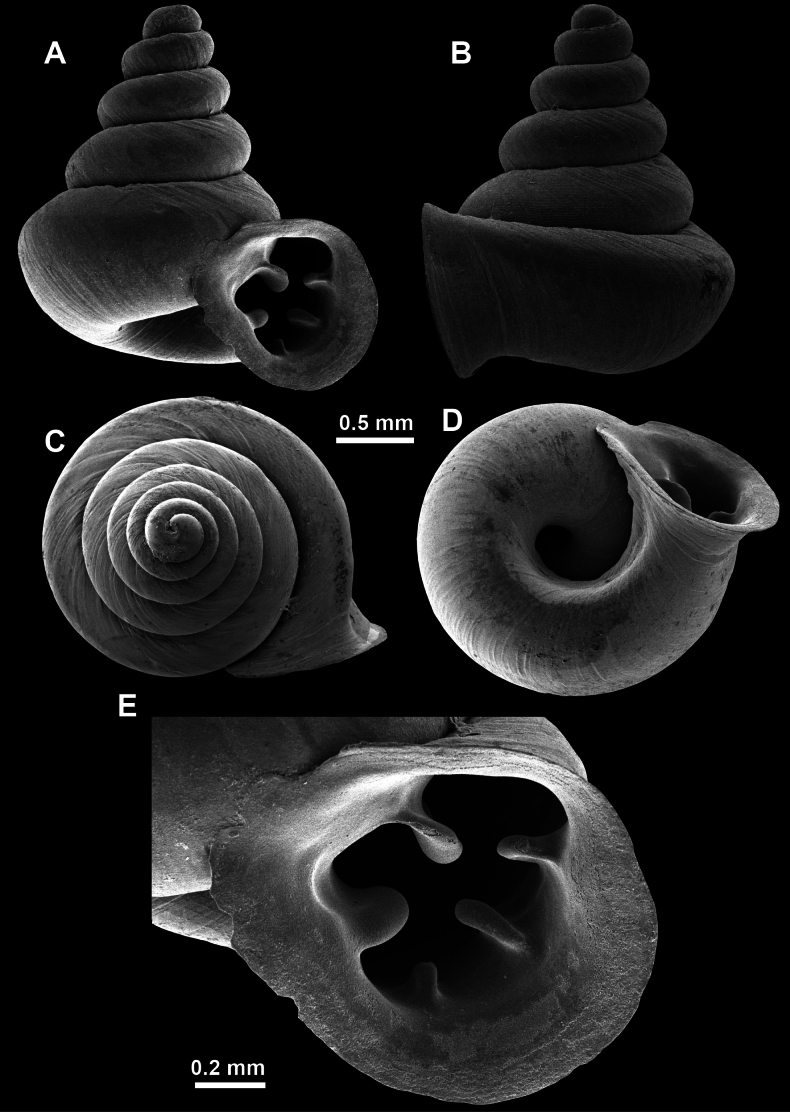
*Anauchenhuaykhakang*, paratype (CUMZ ver. 038) **A–D** shell **E** enlarged apertural view.

##### Distribution.

This species is known only from the type locality.

#### ﻿ Anauchen informis informis

Taxon classificationAnimaliaStylommatophoraHypselostomatidae

Vermeulen, Phung & Truong, 2007

E7E18EFE-1D36-515C-9F43-4D1716E4365F

Figs 10H , 20 , 37

Anauchen informis informis Vermeulen, Phung & Truong, 2007: 87–89, fig. 6.
Anauchen informis informis — Schileyko 2011: 4.

##### Type material examined.

**Vietnam** • holotype; RMNH 108986.

##### Type locality.

Vietnam, Kien Giang Prov., Kien Luong, Hon Chong hill, doline along East flank.

##### Differential diagnosis.

This species is the most similar to *A.angthongensis* from which it differs by its more bulging whorls and more ovoid shell. *A.banmiensis* has a shouldered last whorl and stronger barriers.

**Figure 20.**
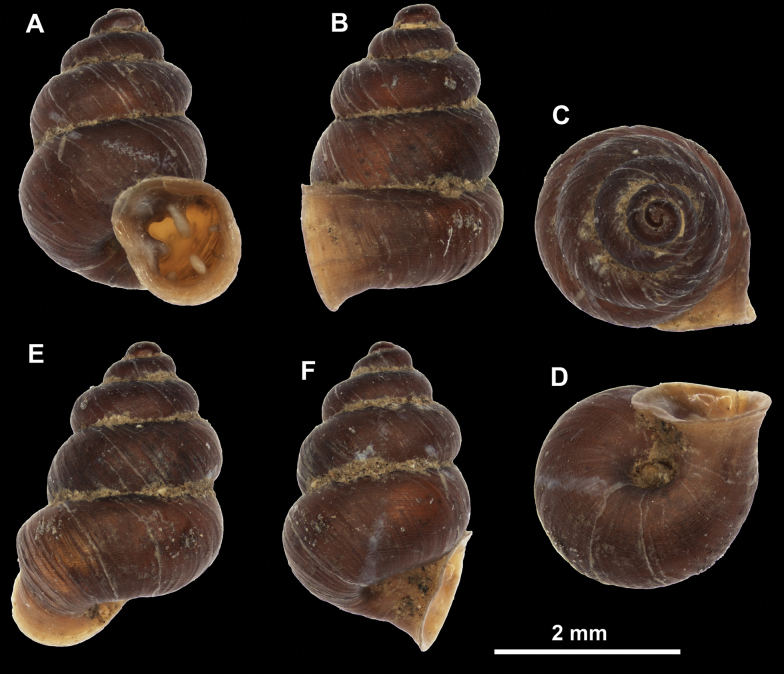
*Anaucheninformisinformis*, holotype (RMNH.Moll.108986) **A–F** shell.

##### Distribution.

This subspecies is known only from the type locality.

#### ﻿ Anauchen informis parcedentata

Taxon classificationAnimaliaStylommatophoraHypselostomatidae

Vermeulen, Phung & Truong, 2007

EEEA001C-BB8D-5A5B-8BED-CFADE9E22A40

Figs 10I , 21 , 37

Anauchen informis parcedentata Vermeulen, Phung & Truong, 2007: 89, fig. 7.
Anauchen informis parcedentata — Schileyko 2011: 4.

##### Type material examined.

**Vietnam** • holotype; RMNH 108987.

##### Type locality.

“Vietnam, Kien Giang prov., Kien Luong, Ba Tai hill”.

##### Differential diagnosis.

Similar to the nominotypical subspecies but with 1–3 barriers in the aperture (parietal always present, both palatals occasionally) and usually more whorls.

**Figure 21.**
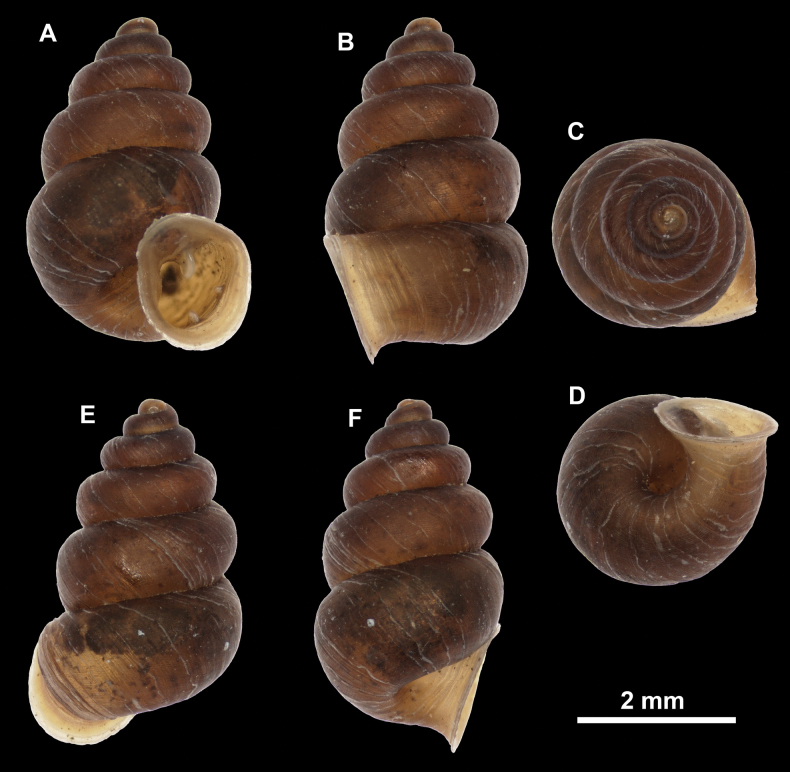
*Anaucheninformisparcedentata*, holotype (RMNH.Moll.108987) **A–F** shell.

##### Distribution.

This subspecies is known only from the type locality.

#### ﻿ Anauchen jokaii

Taxon classificationAnimaliaStylommatophoraHypselostomatidae

Gojšina & Páll-Gergely sp. nov.

2921D2B8-4C54-5541-B001-29D2DF03FB65

https://zoobank.org/E3383AEE-7FCC-4296-9530-4447C33DC952

**Figure 22.**
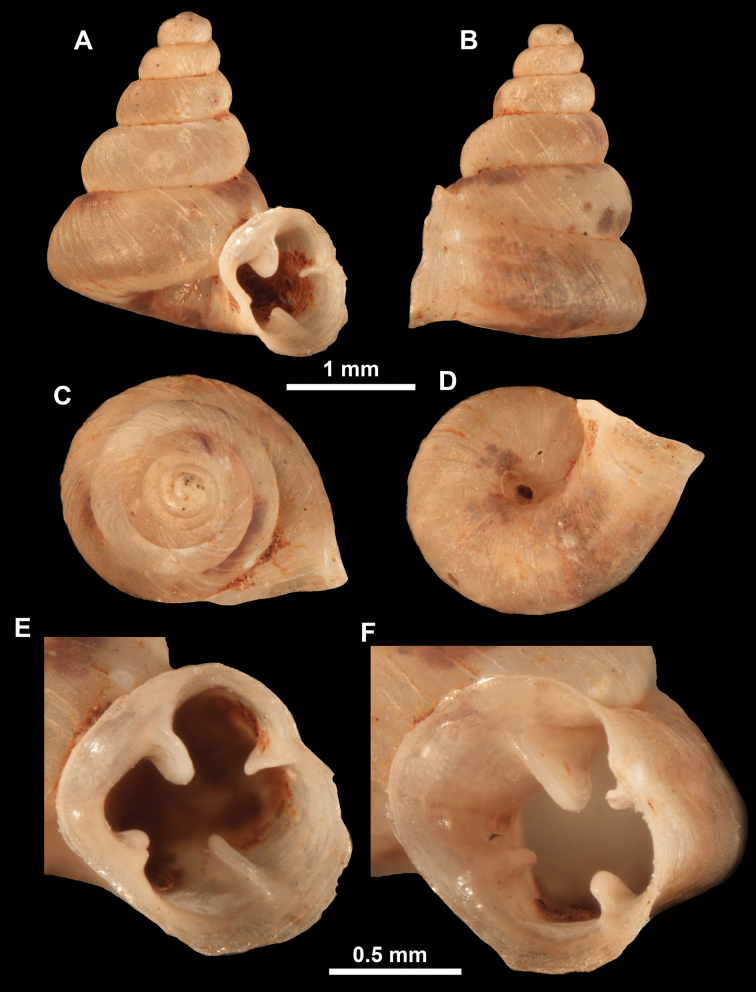
*Anauchenjokaii* Gojšina & Páll-Gergely, sp. nov., holotype (IEBR_LS_Anauchen.002H) **A–D** shell **E, F** enlarged apertural views.

**Figure 23.**
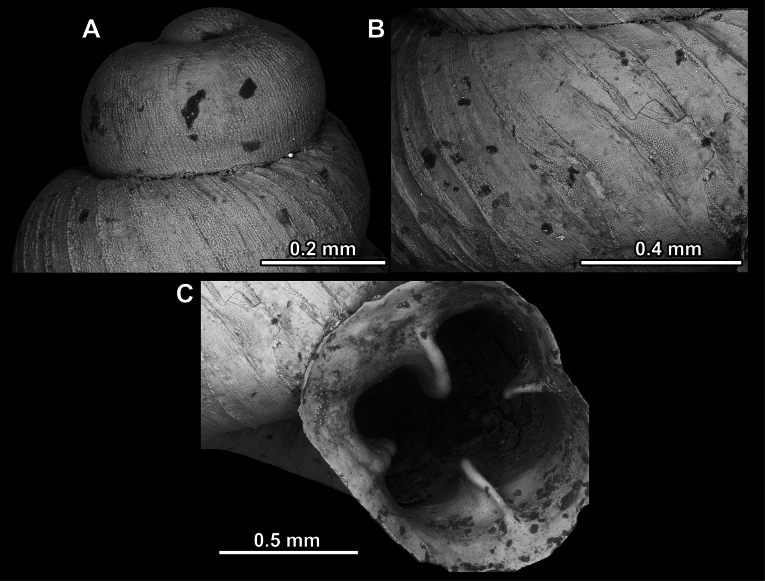
SEM imaging of *Anauchenjokaii* Gojšina & Páll-Gergely, sp. nov., holotype (IEBR_LS_Anauchen.002H) **A** protoconch surface **B** teleoconch surface **C** enlarged apertural view.

Figs 10J , 22 , 23 , 37

##### Type material.

***Holotype*. Vietnam** • 1 shell (SH: 2.48 mm, SW: 2.31 mm); Nam Pia village, Hung Ba commune, Vi Xugen district, Ha Giang Province; 22°52.064'N, 105°4.814'E; 216 m a.s.l.; L.V. Hao, T. Ishibe, Y. Nakahara, K. Ohara, K. Okubo, J.U. Otani, V.P. Sang leg.; IEBR_LS_Anauchen.002H.

##### Type locality.

Vietnam, Nam Pia village, Hung Ba commune, Vi Xugen district, Ha Giang Province; 22°52.064'N, 105°4.814'E; 216 m a.s.l.

##### Diagnosis.

*Anauchen* species with conical shell, last whorl keeled at its base and slightly ascending near the aperture. Spiral striation absent. Aperture armed with four strong barriers, umbilicus narrow.

##### Description.

Shell conical, consisting of 5.5 regularly increasing, convex whorls separated by a deep suture. Colour light brown. There is not a clear boundary between the protoconch and the teleoconch due to the similar surface sculpture. Teleoconch pasty, very finely but irregularly radially striated, with no signs of spiral striation. Last whorl adnate to the penultimate and its last quarter slightly ascending near the aperture (~ 10° compared to the shell axis), making the aperture profile opisthocline to the shell axis. Last whorl with a blunt keel at its base, making the shell strongly triangular. Peristome continuous and thick, not very strongly expanded on the parietal side and not fully leaning on the penultimate whorl. It is slightly lighter in colour than the rest of the shell, not reflected. Sinulus rounded and well separated from the rest of the aperture. Aperture equipped with four strong but short barriers (parietal, upper palatal, lower palatal and columellar). Parietal lamella is the strongest in the aperture, blade-like. Upper palatal plica ~ 1/2 as strong as the parietal lamella and very slightly leaning towards the lower palatal plica. The latter is strong (only slightly weaker than the parietal) and moderately long. There is no basal plica. Columellar lamella thick and relatively low, situated in the middle of the columellar wall. All apertural barriers almost reach the expanding peristome. Surface of all apertural barriers is smooth. Umbilicus rounded, open but narrow, measuring ~ 1/11 of the shell width.

##### Differential diagnosis.

See under *A.messageri*, *A.rochebruni*, *A.pentadens* and *A.turritus* sp. nov.

##### Measurements

**(in mm, *n* = 1).**SH = 2.48; SW = 2.31; AH = 1.12; AW = 1.04.

##### Etymology.

This species is named after and dedicated to Mór Jókai (1825–1904), Hungarian novelist, dramatist, to commemorate the bicentenary of his birth. Jókai was interested in malacology, he had a large collection of (mostly marine) shells, and even published a satirical novel entitled “Novel of snails” (A csigák regénye).

##### Distribution.

Only known from the type locality.

#### ﻿ Anauchen kozari

Taxon classificationAnimaliaStylommatophoraHypselostomatidae

Páll-Gergely, 2023

54F9A976-131F-5612-ADA2-7504B83BAF42

Figs 10K , 24 , 37

Anauchen kozari Páll-Gergely, 2023a: 64–66, figs 1D, F, 2, 3.

##### Type material examined.

**Laos** • holotype; Luang Prabang Province, Phou Xuang Mountain, ca 1.5 km NE of Ban Lak Sip, ca 5 km SE of Luang Prabang, under rocks and logs in old secondary forest under cliff; 19°851.6050'N, 10°2811.0810'E; 640 m a.s.l.; A. Abdou, I.V Muratov leg.; 24 Nov. 2006; MNHN-IM-2012-27269.

##### Type locality.

“Central Laos, Luang Prabang Province, Phou Xuang mountain, ca 1.5 km NE of Ban Lak Sip, ca 5 km SE of Luang Prabang, under rocks and logs in old secondary forest under cliff, 19°851.6050'N, 10°2811.0810'E, alt. 640 m”.

##### Differential diagnosis.

See under *A.messageri*.

##### Distribution.

This species is known only from the type locality.

##### Remarks.

This species is not a typical representative of its genus since it has a palatal tubercle frequently found in *Bensonella* and a granulated, rough surface of the apertural barriers frequently found in *Hypselostoma*. It is also the smallest of all *Anauchen* species. It is however retained in *Anauchen* for the following reasons: i) the arrangement of apertural barriers is highly reminiscent to those of *A.messageri* (see Páll-Gergely 2023a); ii) there is a single lamella on the parietal side; iii) last whorl adnate to the penultimate.

**Figure 24.**
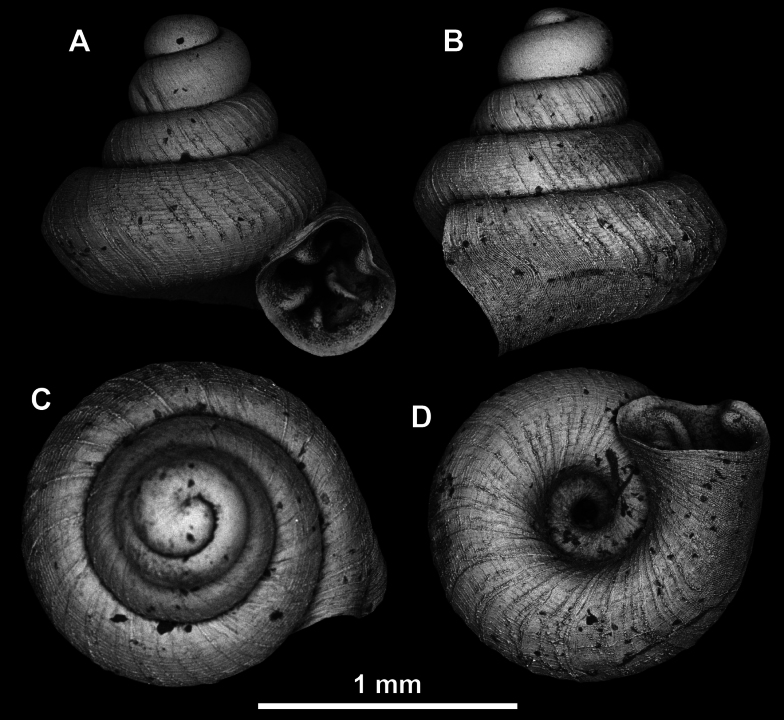
*Anauchenkozari* Páll-Gergely, 2023, holotype (MNHN-IM-2012-27269) (from Páll-Gergely 2023a) **A–D** shell.

#### ﻿ Anauchen messageri

Taxon classificationAnimaliaStylommatophoraHypselostomatidae

(Bavay & Dautzenberg, 1904)

AE544CE2-E66A-5728-9A1D-68A7773350CB

Boysidia messageri Bavay & Dautzenberg, 1904: 211, pl. 9, figs 1–4.
Anauchen messageri — Pilsbry 1917: 189, pl. 33, figs 4–7; Schileyko 2011: 5; Páll-Gergely 2023a: 67, fig. 1C.
Anauchen massageri [sic!] — Burch and Panha 2002: 242, 245, 247.
Anauchen whitteni Chen, 2023: 90–91, figs 1, 2. syn. nov.

##### Type material examined.

**Vietnam** • 1 syntype of *A.messageri*; from the type locality; C. Messager leg.; MNHN-IM-2000-35159.

##### Additional material examined.

**Vietnam** • 2 shells; Cao Bang Province, Đeo Ma Phuc - Quang Uyen, left side of the road, rock crevice; 22°43.981'N, 106°20.333'E; 610 m a.s.l.; 14 Nov. 2011; A. Hunyadi leg.; coll. HA • 1 shell; Cao Bang Province, west of Quang Uyen, Phi Hao-Đau Tuyen; 22°42.188'N, 106°26.358'E; 500 m a.s.l.; 16. Nov 2011.; A. Hunyadi leg.; coll. HA • 8 shells; Lang Son Province, Huu Lung district, Minh Tien, 1400 m northeast of Cau Cheo Minh Tien; 21°34.024'N, 106°17.790'E; 30 m a.s.l.; 20 Feb. 2020; A. Hunyadi leg.; coll. HA • 13 shells; Lang Son Province, Huu Lung district, Huu Lien, 33.5 km from junction of roads 1B and 241 towards Ba Nang, left side of the road; 21°41.064'N, 106°22.871'E; 220 m a.s.l.;19 Feb. 2020; A. Hunyadi leg; coll. HA • 47 shells; Hoa Binh Province, Kim Boi district, Cao Duong, north of Đong Phu, 58 km from Nho Quan towards Hanoi on the Ho Chi Minh road; 20°42.591'N, 105°39.299'E; 10 m a.s.l.; 16 Feb. 2020; A. Hunyadi, H.V. Luong, J.U. Otani & S.V. Pham leg.; coll. HA • 1 shell; Thanh Hoa Province, Xa Ban Cong, Xom Sat; 20°22.897'N, 105°12.712'E; 60 m a.s.l.; 18. May 2012¸ A. Hunyadi leg.; coll. HA • 15 shells; Thanh Hoa Province, 9.8 km from centre of Ngoc Lac towards Lang Chanh, Ngoc Khe, left side of the road, 115 km; 20°7.192'N, 105°18.277'E; 13 Feb. 2020; A. Hunyadi, H.V. Luong, J.U. Otani & S.V. Pham leg.; coll. HA. **China** • 9 shells; Guangxi, Bose Shi, Leye Xian, Buliu He; 24°39.436'N, 106°43.245'E; 540 m a.s.l.; 08 Sept. 2013; A. Hunyadi leg.; coll. HA • 74 shells; Guangxi, Hechi Shi, Huanjiang Maonanzu Zizhixian, south of Mulun Guojiaji Ziran Baohuqu, Dongning; 25°05.970'N, 107°57.639'E; 530 m a.s.l.; 17 Sept. 2013, A. Hunyadi leg.; coll. HA • 3 shells; Guangxi, Laibin Shi, Xingbin Qu, Qidong Xiang; 24°00.512'N, 109°04.288'E; 150 m a.s.l.; 20 Sept. 2013; A. Hunyadi leg. • 2 shells; coll. HA • 2 shells; Guangxi, Hechi Shi, Du an Yaozu Zizhixian, Gaoling Xiang, 2 km west of Dingfucun; 24°03.197'N, 108°01.290'E; 320 m a.s.l.; 08 Oct. 2009; A. Hunyadi leg.; coll. HA • 171 shells; Guangxi, Hechi Shi, Bama Yaozu Zizhixian, southern edge of Jiaolecun; 24°07.045'N, 107°07.847'E; 590 m a.s.l.; 10 Sept. 2013; A. Hunyadi leg.; coll. HA • 24 shells; Guangxi, Hechi Shi, Nandan Xian, Lihuyaozu Xiang, southeast of Encun, rocks above the road; 25°04.163'N, 107°36.176'E; 630 m a.s.l.; 11 Sept. 2013; A. Hunyadi leg.; coll. HA • 10 shells; Guangxi, Guilin Shi, Guilin, Ludiyan Gongyuan, northeastern side of Moupanshan; 25°18.466'N, 110°15.862'E 145 m a.s.l.; 22 Sept. 2013; A. Hunyadi leg.; coll. HA • 14 shells; Guangxi, Chongzuo Shi, Longzhou Xian, Wude Xiang, vicinity of the junction towards Banxintun; 22°35.239'N, 106°46.096'E; 350 m a.s.l.; 24 Sept. 2013; A. Hunyadi leg.; coll. HA • 257 shells; Guangxi, Hechi Shi, Tiane Xian, Qimu Xiang, junction towards Lahaiyan, rock above the tomb; 24°51.130'N, 107°11.670'E; 600 m a.s.l.; 12 Sept. 2013; A. Hunyadi & M. Szekeres leg.; coll. HA • 5 shells; Guangxi, Guilin Shi, Yangshuo Xian, 5 km north of Xingpingzhen; 24°57.358'N, 110°31.857'E; 150 m a.s.l.; 15 Oct. 2009; A. Hunyadi leg.; coll. HA • 13 shells; Yueliangshan, Gaotianzhen, Yangshuoxian, Guangxi Zhuangzu Zizhiqu; 24°43.45'N, 110°28.32'E; K. Ohara, K. Okubo, J.U. Otani leg.; coll. PGB • 17 shells; Daxiaojingfengjingqu, Moyangzhen, Luodianxian, Guizhousheng; 25°33.73'N, 106°51.4333'E; 450 m a.s.l.; T. Ishibe, K. Okubo, J.U. Otani leg.; coll. PGB • 4 shells; near Hudiequan bridge, Gaotianzhen, Yangshuoxian, Guangxi Zhuangzu Zizhiqu; 24°44.45'N, 110°29.62'E; K. Ohara, K. Okubo, J.U. Otani leg.; coll. PGB.

##### Type localities.

“Haut-Tonkin” (Vietnam) (*A.messageri*); “Collected from the rocks, Mt. Nanshe [南蛇山], Yizhou District [宜州区], Hechi City [河池市], Guangxi Zhuang Autonomous Region, China. 24°30'22"N, 108°38'51"E, 270 m…” (*A.whitteni*).

##### Differential diagnosis.

*Anauchenjokaii* sp. nov. lacks the spiral striation and has its last whorl keeled at the base which separates it from *A.messageri*. *Anauchenkozari* is smaller, more depressed and has a palatal tubercle. See also under *A.pentadens*, *A.rochebruni* and *A.turritus* sp. nov.

##### Distribution.

This species has a wide distribution across Vietnam and China. Its northernmost known localities are situated in northern parts of Guangxi province (China) while the southernmost are from Thanh Hoa province (Vietnam).

##### Remarks.

**Figure 25.**
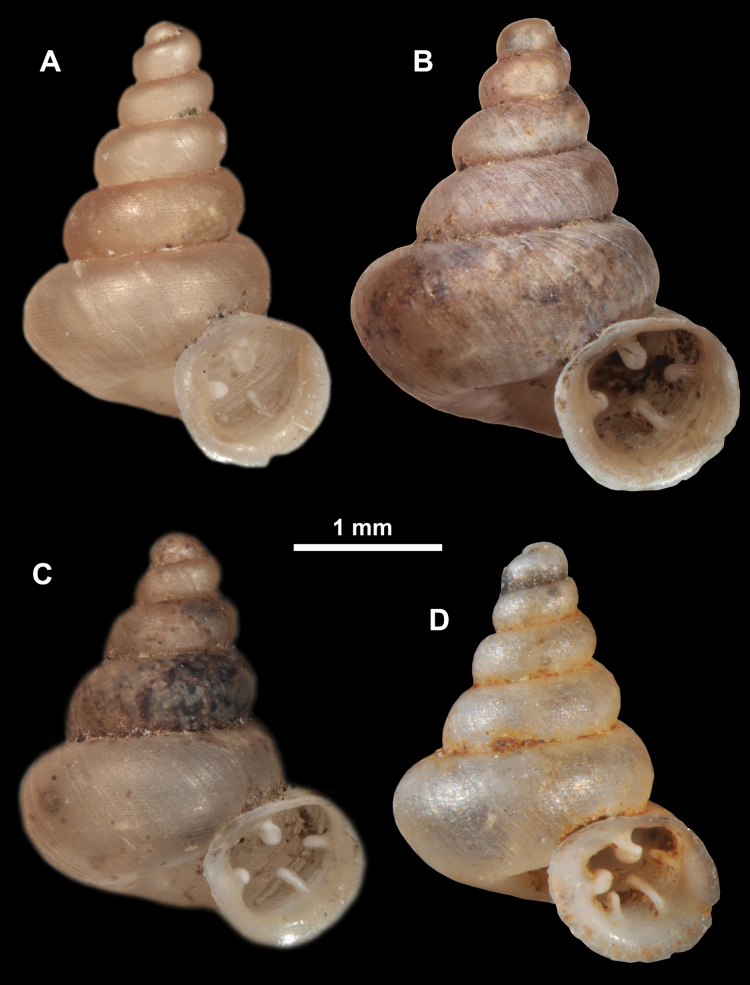
*Anauchenmessageri* from different localities **A** China, Guangxi province, Hechi Shi (coll. HA) **B** China, Guangxi province, Bose Shi (coll. HA) **C** China, Guangxi province, Guilin Shi (coll. HA) **D** Vietnam, Tonkin, syntype (MNHN-IM-2000-35159 (from Páll-Gergely 2023a)).

We have observed high morphological variability within this species, particularly in the slenderness of the shell (Fig. 25). The basal plica is also occasionally present or sometimes absent. *Anauchenwhitteni* Z.-Y. Chen, 2023 was described as a distinct species based on the absence of the basal plica. Since the basal plica was found here as a variable character and the species does not differ from *A.messageri* in any other characters, it is treated here as a junior synonym.

#### ﻿ Anauchen obesus

Taxon classificationAnimaliaStylommatophoraHypselostomatidae

Gojšina, Hunyadi & Páll-Gergely sp. nov.

29907147-B2E2-503C-87BE-AA83B7395662

https://zoobank.org/C9C955AA-7176-479B-A159-B8487F31196A

**Figure 26.**
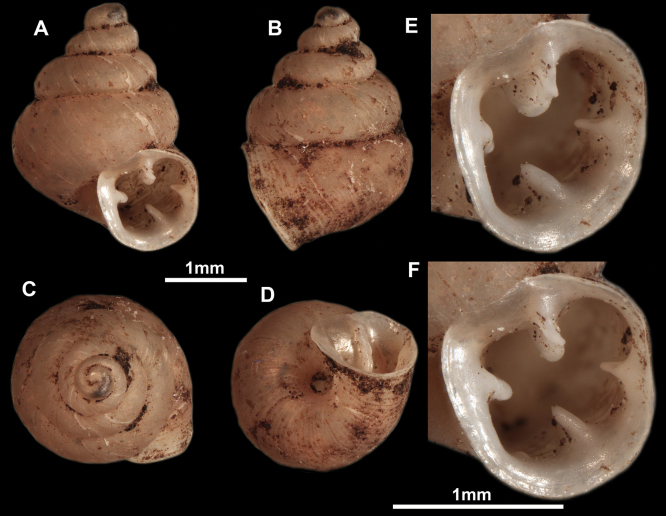
*Anauchenobesus* Gojšina, Hunyadi & Páll-Gergely, sp. nov., holotype (CUMZ 14429) **A–D** shell **E, F** enlarged apertural views.

**Figure 27.**
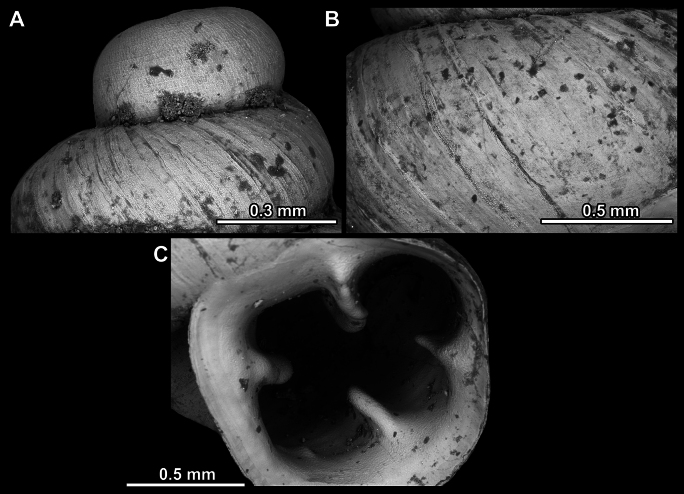
SEM imaging of *Anauchenobesus* Gojšina, Hunyadi & Páll-Gergely, sp. nov., holotype (CUMZ 14429) **A** protoconch surface **B** teleoconch surface **C** enlarged apertural view.

Figs 10M , 26 , 27 , 37

##### Type material.

***Holotype*. Thailand** • 1 shell (SH: 2.94 mm, SW: 2.26 mm); Chiang Rai Province, Doi Tung, 50 metres before Wat Phra That Doi Tung, vicinity of the parking lot; 20°19.540'N, 99°49.987'E; 1350 m; 12 Feb. 2015; A. Hunyadi leg.; CUMZ 14429. ***Paratypes*. Thailand** • 15 shells; same data as for holotype; coll. HA • 10 shells; Chiang Rai Province, Doi Tung; 20°20′32″N, 99°50′21″E; 1320 m a.s.l.; 08. May 1988; F.G. Thompson leg.; locality code FGT-4417; UF 591335.

##### Additional material examined.

**Thailand** • 1 shell (juvenile, not paratype); Chiang Rai Province, Doi Tung; 20°20′32″N, 99°50′21″E; 1320 m a.s.l.; 08. May 1988; F.G. Thompson leg.; locality code FGT-4417; UF 583720.

##### Type locality.

Thailand, Chiang Rai Province, Doi Tung, 50 metres before Wat Phra That Doi Tung, vicinity of the parking lot; 20°19.540'N, 99°49.987'E; 1350 m.

##### Diagnosis.

*Anauchen* species with conical-ovoid, brown shell, pasty-like surface, last whorl adnate to the penultimate, five apertural barriers (parietal (angulo-parietal), upper palatal, lower palatal, weak basal, and columellar) and a narrow umbilicus.

##### Description.

Shell conical-ovoid, brown, with 4.5–5 whorls separated by a deep suture. All whorls convex, bulging, rounded. Protoconch consists of ~ 1.5 whorls, finely pitted and with moderate spiral striae, the same colour as the rest of the shell. Teleoconch fine, pasty, with inconspicuous, irregular growth radial lines which are rarely more pronounced and in form of whitish streaks, most dense right behind the peristome. Spiral striation absent. Last whorl adnate to the penultimate and slightly descending (~ 25 ° compared to the shell axis) making the aperture profile prosocline to the shell axis. Peristome continuous, in form of a short and not much expanded callus leaning on the penultimate whorl at the parietal side. It is white, expanded but not reflected. Aperture equipped with five barriers (parietal (angulo-parietal), upper palatal, lower palatal, basal and columellar). Parietal (angulo-parietal) lamella almost fully reaching the expanded peristome edge. It is the largest in the aperture, sometimes single and sometimes with an additional smaller part, which is probably homologous with the angular lamella in other genera, pointed towards the palatal side. Upper palatal plica is weaker, lower, and less slender than the lower palatal. Lower palatal plica almost as strong as the angulo-parietal lamella. Basal plica in the form of inconspicuous knob, like a very slight swelling in the basal-columellar transition area. Columellar lamella almost reaching the expanded peristome, highest in its middle part and sloping towards the peristome edge where it becomes quite low. It is leaned towards the lower palatal plica. Surface of all apertural barriers is very finely granulated. Sinulus rounded, parabolic and well separated from the rest of the aperture. Umbilicus narrow, ~ 1/8 of the shell width, previous whorls not clearly visible through it. Umbilical groove absent.

##### Differential diagnosis.

*Anauchenobesus* sp. nov. is similar in shell shape to *A.angthongensis*, *A.crassus* sp. nov., *A.eotvosi* and *A.informis*. However, *A.obesus* sp. nov. can be separated by its smaller size and more globular shell.

##### Measurements

**(in mm, *n* = 4).**SH = 2.71–3; SW = 1.95–2.26; AH = 1.1–1.27; AW = 1.06–1.23.

##### Etymology.

This species is named for its globose shell.

##### Distribution.

This species is known only from Doi Tung mountain.

#### ﻿ Anauchen picasso

Taxon classificationAnimaliaStylommatophoraHypselostomatidae

Gojšina & Páll-Gergely sp. nov.

67988AA5-6FEB-55D0-88F1-104C261F45B0

https://zoobank.org/8758A63B-ACBB-43C6-B654-53D5FF312907

**Figure 28.**
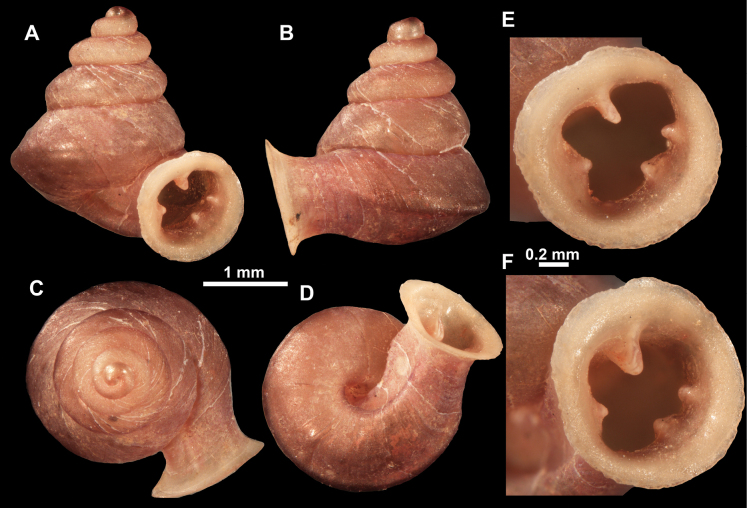
*Anauchenpicasso* Gojšina & Páll-Gergely, sp. nov., holotype (UF 529706) **A–D** shell **E, F** enlarged apertural views.

**Figure 29.**
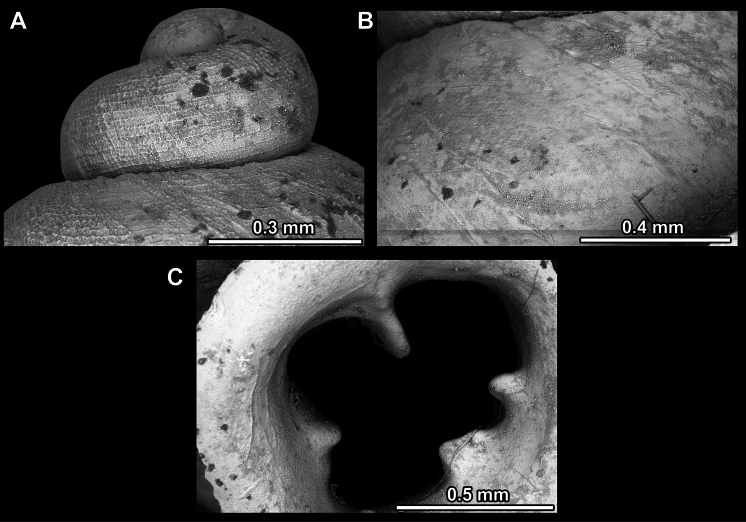
SEM imaging of *Anauchenpicasso* Gojšina & Páll-Gergely, sp. nov., holotype (UF 529706) **A** protoconch surface **B** teleoconch surface **C** enlarged apertural view.

Figs 10N , 28 , 29 , 37

##### Type material.

***Holotype*. Thailand** • 1 shell (SH: 2.95 mm, SW: 2.79 mm); Prachuap Khiri Khan Province, Khao Sam Roi Yot National Park, 3 km S of Park Entrance; 12.208°N, 99.993°E; 31 May 1987; F.G. Thompson leg.; UF 529706. ***Paratypes*. Thailand** • 1 shell; same data as for holotype; CUMZ 14430; 8 shells; same data as for holotype; UF 591339.

##### Additional material examined.

**Thailand** • 1 shell (juvenile/broken, not paratype); same data as for holotype; UF 583731.

##### Type locality.

Thailand, Prachuap Khiri Khan Province, Khao Sam Roi Yot National Park, 3 km S of Park Entrance; 12.208°N, 99.993°E.

##### Diagnosis.

*Anauchen* species with distinctly keeled last whorl (slightly below the centre of the periphery) which is also detached near the aperture and descending. Teleoconch spirally striated. Only four weak apertural barriers (parietal, upper palatal, lower palatal and columellar).

##### Description.

Shell conical or very slightly conical-ovoid, brown, consisting of 4.5–5 whorls separated by a deep suture. Protoconch not much lighter than the rest of the shell, roughly pitted and terminally spirally striated, consisting of 1.25 whorls. Initial teleoconch whorls and the penultimate whorl slightly keeled. Fine spiral striation visible on the teleoconch as well, sometimes stronger near the protoconch (on initial teleoconch whorls), sometimes stronger on the last whorl. There are several irregularly spaced whitish radial streaks crossing the spiral striae. Last whorl enlarged and distinctly keeled slightly below the centre of the periphery. The keel is equally strong along its length but is getting lost right behind the peristome. Last whorl slightly detached from the penultimate near the aperture and slightly descending (~ 15 ° compared to the shell axis), thus making the aperture profile prosocline to the shell axis. Peristome is dirty white, very thick, expanded but not reflected. Aperture rounded, almost as high as wide. There are only four relatively weak apertural barriers (parietal, upper palatal, lower palatal and columellar). Of them, parietal lamella is the strongest and highest and reaching the expanding peristome. Both palatal plicae (upper and lower) are of similar appearance (but lower is usually slightly stronger than the upper), both weak (low and short) and tubercle-like. They are positioned close to each other, probably closer than in any other congener. Columellar lamella is weak and dot-like, usually not present as a distinct barrier but rather as a columellar thickening which is gradually lowering towards the expanding peristome. Surface of all apertural barriers is very finely granulated. Sinulus wide and not strongly isolated from the rest of the aperture due to the relatively weak upper palatal plica. Umbilicus very narrow, elongated and measuring 1/8–1/9 of the shell width. There is a strong umbilical groove running along the umbilicus. This groove is very wide near the peristome and then regularly tapering towards the umbilicus, finally terminating at the ~ ½ of the last whorl.

##### Differential diagnosis.

This species is different from other congeners by the strongly keeled last whorl below the centre of the periphery and the presence of four apertural barriers which are quite weak. It is most similar to *A.evanidus* sp. nov. but *A.picasso* sp. nov. has a more strongly keeled last whorl, weaker apertural barriers, and is spirally striated.

##### Measurements

**(in mm, *n* = 5).**SH = 2.64–2.95; SW = 2.63–2.82; AH = 1.23–1.38; AW = 1.27–1.37.

##### Etymology.

This species looks like an *Anauchen* with rounded whorls painted in a Pablo Picasso style (i.e., resembling the art style known as Cubism). The specific epithet is to be used as a noun in apposition.

##### Distribution.

This species is known only from the type locality.

#### ﻿ Anauchen pentadens

Taxon classificationAnimaliaStylommatophoraHypselostomatidae

(D.-N. Chen, M. Wu & G.-Q. Zhang, 1999)

6B2E5B70-A63C-58D9-A55D-6C11D0619540

Fig. 37

Boysidia (Boysidia) pentadens Chen, Wu & Zhang, 1999: 5 (Chinese description), 11 (English description), fig. 2. — Chen and Zhang 2002: 692.
Boysidia pentadens — Qian and Zhou 2014: 90–91; Chen et al. 2016: 50; Dumrongrojwattana and Assawawattagee 2018: 216.
Anauchen pentadens — Chen 2023: 89–90.

##### Material examined.

None.

##### Type locality.

“collected from Menglun Town (21°5'N, 100°6'E). Mengla County, Yunnan Province” (China).

##### Differential diagnosis.

This species differs from *A.messageri* by its less triangular shell, narrower umbilicus, absence of spiral striation, and presence of strong radial ribs on the last whorl (especially near the aperture). *Anauchenpentadens* differs from *A.jokaii* sp. nov. by its wider umbilicus, strong ribs on the last whorl, especially near the aperture, and a basal plica. Its last whorl is also regularly rounded, so the is shell less triangular. See also under *A.rochebruni*.

##### Distribution.

This species is known only from Yunnan Province, China.

##### Remarks.

According to the figures presented in Qian and Zhou (2014) and Chen et al. (2016), Chen (2023) considered it a member of *Anauchen* rather than *Boysidia* which we follow herein.

#### ﻿ Anauchen rochebruni

Taxon classificationAnimaliaStylommatophoraHypselostomatidae

(Mabille, 1887)

C85319C0-F7BE-53FB-BF7E-1987BC4A0D43

**Figure 30.**
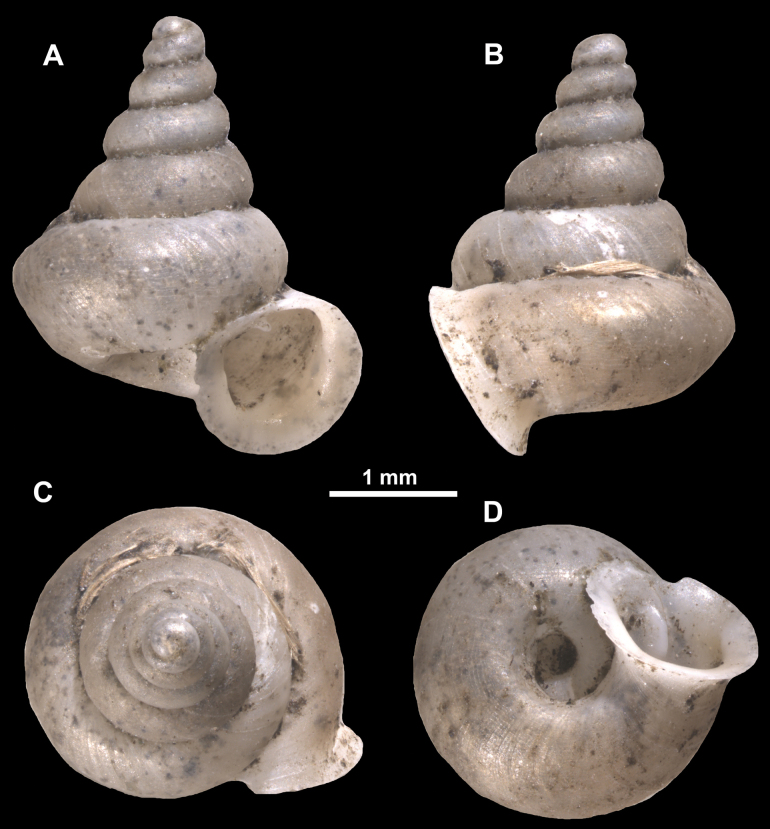
*Anauchenrochebruni* from Hunan, China (SMF 42108) **A–D** shell.

**Figure 31.**
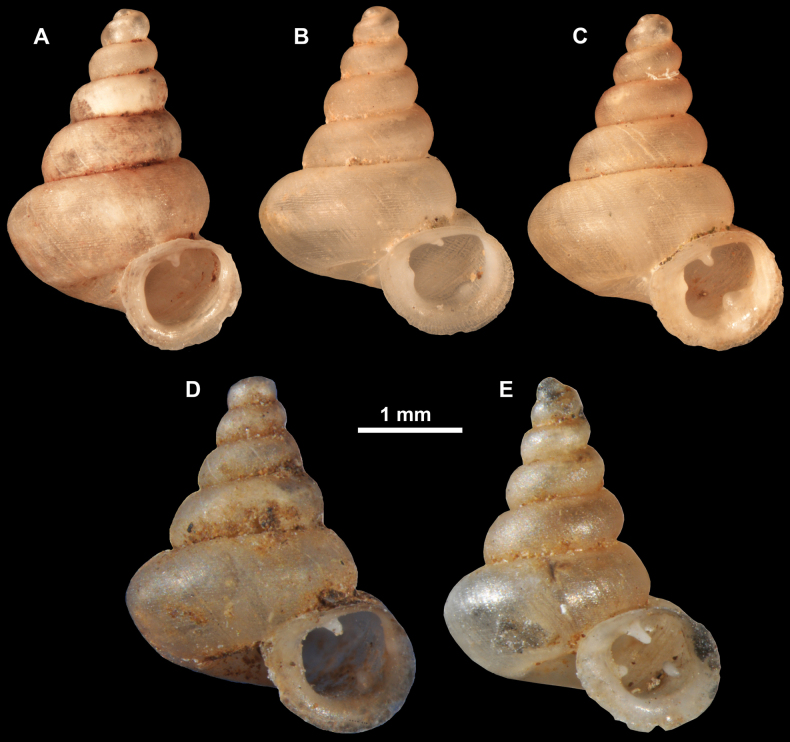
*Anauchenrochebruni* from different localities **A** Vietnam, Cao Bang province (coll. HA) **B** China, Guangxi province (coll. HA) **C** Vietnam, Lang Son province (coll. HA) **D** Vietnam, Tonkin (syntype, MNHN-IM-2000-35156 (from Páll-Gergely 2023a)) **E** Vietnam, Tonkin, syntype of *B.gereti* (MNHN-IM-2000-35161 (from Páll-Gergely 2023a)).

Figs 10O , 30 , 31 , 37 , 38

Hypselostoma rochebruni Mabille, 1887a: 8.
*Pupaangulina* Gredler, 1885: 7. syn. nov.
Hypselostoma rochebruni — Mabille 1887b: 121–122.
Boysidia gereti Bavay & Dautzenberg, 1904: 212, pl. 9, figs 5–8.
Hypselostoma rochebrunei [sic] — Fischer and Dautzenberg 1904: 408.
Anauchen angulinus — Pilsbry 1917: 191, pl. 33, figs 5, 6.
Anauchen rochebruni — Pilsbry 1917: 190–191; Páll-Gergely 2023a: 63, 67, figs 1A, B.
Anauchen gereti — Pilsbry 1917: 189–190, pl. 33, figs 1–3; Schileyko 1998: 138, fig. 158; Schileyko 2011: 4.
Hypselostoma rochebruni — Schileyko 2011: 3.
Boysidia gereti — Páll-Gergely 2023a: 67, fig. 1A.

##### Type material examined.

**Vietnam** • 1 syntype of *A.rochebruni*; from the type locality; collector unknown; MNHN-IM-2000-35156 • 1 syntype of *B.gereti*; from the type locality; C. Messager leg.; MNHN-IM-2000-35161.

##### Additional material examined.

**Vietnam** • 59 shells; Lang Son Province, Binh Gia, eastern edge of the village, Di Chi Keo Leng, rock wall; 21°56.302'N, 106°23.819'E; 375 m a.s.l.; 17 Feb. 2020; A. Hunyadi leg.; coll. HA • 55 shells; Lang Son Province, Bac Son district, Quinh Son, northern part of the village; 21°53.411'N, 106°20.944'E; 400 m a.s.l.; 18 Feb. 2020; A. Hunyadi leg.; coll. HA • 31 shells; Lang Son Province, Bac Son district, Quinh Son, northeastern part of the village, rock wall; 21°54.616'N, 106°20.542'E; 420 m a.s.l.; 18 Feb. 2020; A. Hunyadi leg.; coll. HA • 25 shells; Lang Son Province, Bac Son district, Long Dong, 3.8 km north from junction of roads 1B and 241; 21°56.728'N, 106°19.447'E; 390 m a.s.l.; 18 Feb. 2020; A. Hunyadi leg.; coll. HA • 4 shells; Lang Son, vicinity of Chua Tam Than; 21°51.353'N, 106°44.809'E; 265 m a.s.l.; 21 Feb. 2020; A. Hunyadi leg.; coll. HA • 1 shell; Ha Giang Province, Ha Giang 105.5 km - Đong Van, Xa Van Chai, left side of the road; 23°09.084'N, 105°10.774'E; 31. May 2012; A. Hunyadi leg.; coll. HA • 1 shell; Cao Bang Province, Đong Khe 3 km- Đeo Lung Phay, right side of the road; 22°24.223'N, 106°25.937'E; 390 m a.s.l.; 15 Nov. 2011, A. Hunyadi leg.; coll. HA • 1 shell; Cao Ban Province, Tring Khanh district, Canh Tien commune, Pac Rao village; 22°48.9408'N, 106°30.549'E; 544 m a.s.l.; L.V. Hao, T. Ishibe, Y. Nakahara, K. Ohara, K. Okubo, J.U. Otani, V.P. Sang leg.; coll. PGB. • 5 shells; Bac Kan Province, Na Ri district, 2 km S of Ban Dem, W slopes of a deep sinkhole covered with forest, in small cavern, in dense rainforest; 06 Apr. 2012; J. Grego, J. Šteffek leg.; coll. JG. **China** • 2 shells; Hunan; ex. coll. Mollendorff; SMF 42108 • 2 shells; Guangxi, Laibin Shi, Wuxuan Xian, Wuxuan Tongling Zhen str., north of Yagangcun, rocks; 23°29.827'N, 109°38.534'E; 100 m a.s.l.; 21 Sept. 2013; A. Hunyadi & M. Szekeres leg. • 17 shells; Guangxi, Hechi Shi, Bama Yaozu Zizhixian, southern edge of Jiaolecun; 24°07.045'N, 107°07.847'E; 590 m a.s.l.; 10 Sept. 2013; A. Hunyadi & M. Szekeres leg. • 1 shell; Guangxi, Yulin Shi, Xingye Xian, 2 km southeast frim Chenghuang, Lufengshan Scenic Area; 22°36.814'N, 109°45.457'E; 05 Oct. 2009; A. Hunyadi leg.; coll. HA • 147 shells; Guangxi, Chongzuo Shi, Longzhou Xian, north of Lenglei, Nonggang Nat. Res., northern edge; 22°29.161'N, 106°57.357'E; 220 m a.s.l.; 23 Sept. 2013; A. Hunyadi & M. Szekeres leg.; coll. HA • 2 shells; Hunan, coll. Moellendorff; SMF 42108; • 2 shells; Guangxi, Chongzuo Shi, 25 km southeast from the centre of Chongzuo, Chongzuo Shengtai Gongyuan, Langur Reserve; 22°16.293'N, 107°30.359'E; 220 m a.s.l.; 18 Oct. 2009; A. Hunyadi leg.; coll. HA • 51 shells; Guangxi, Chongzuo Shi, Longzhou Xian, Wude Xiang, Banxintun; 22°35.239'N, 106°46.096'E; 350 m a.s.l.; 24 Sept. 2013; A. Hunyadi leg.; coll. HA • 7 shells; Tao cun, Tong’anzhen, Pinglexian, Guangxi Zhuangzu Zizhiqu; 24°34.379'N, 110°56.022'E; 163 m a.s.l.; leg. K. Ohara, K. Okubo, J.U. Otani; coll. PGB.

##### Type localities.

“Tonkin” (Vietnam) (*A.rochebruni*); “Die nähere Heimat dieses Findelkindes von einer Windelschnecke ist nach obigem vorläufig nimmer wohl festzustellen; doch sicher im Innern des centralen China, in Hunan oder Hupé zu suchen.” [type locality questionable: Hunan or Hubei provinces, China] (*A.angulinus*).

##### Differential diagnosis.

The shell shape is most similar to *A.messageri*. However, it differs by the much weaker and fewer apertural barriers. *Anauchenpentadens* is less triangular, has a narrower umbilicus, lacks the spiral striation, has more apertural barriers which are also stronger and has strong ribs on the last whorl (especially near the aperture). *Anauchenjokaii* sp. nov. lacks the spiral striation and has its last whorl keeled at the base which separates it from *A.rochebruni*. The apertural barriers are also much weaker in *A.rochebruni*. See also under *A.turritus* sp. nov.

##### Distribution.

This species has a wide distribution extending from the Hunan province, China (type locality of *A.angulinus*) south to Lang Son province, Vietnam.

##### Remarks.

*Anauchenangulinus* is herein treated a synonym of this species because we observed no significant morphological differences between them.

There is intraspecific variation in the number of apertural barriers. There are usually three (parietal, palatal, columellar) but there can be as few as one (parietal). The last whorl can be more or less swollen, sometimes resulting in a strongly triangular shell shape.

#### ﻿ Anauchen turritus

Taxon classificationAnimaliaStylommatophoraHypselostomatidae

Gojšina, Hunyadi & Páll-Gergely sp. nov.

1C6E6023-08FA-51A5-A002-C3908F2DACFF

https://zoobank.org/89B0FF84-A253-4CC3-8906-BFF5DCD3F240

**Figure 32.**
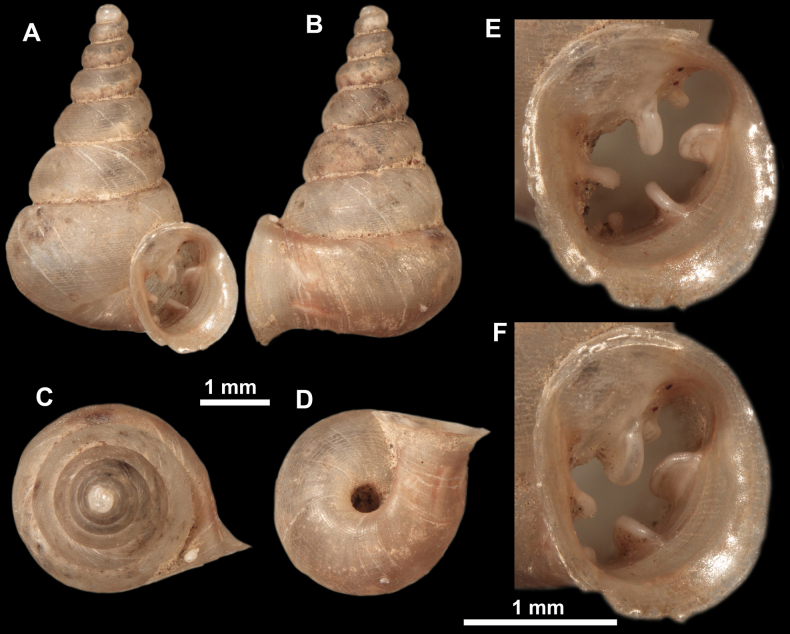
*Anauchenturritus* Gojšina, Hunyadi & Páll-Gergely, sp. nov., holotype (IEBR_LS_Anauchen.001H) (slender form) **A–D** shell **E, F** enlarged apertural views.

**Figure 33.**
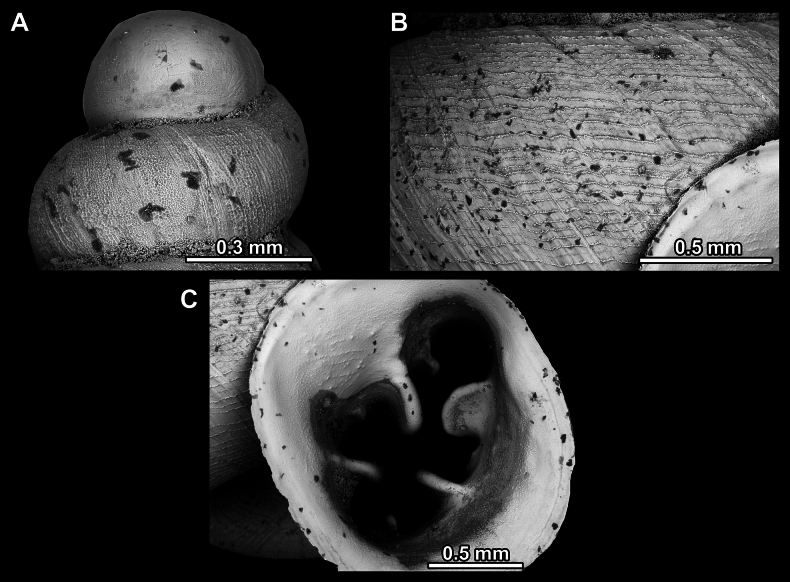
SEM imaging of *Anauchenturritus* Gojšina, Hunyadi & Páll-Gergely, sp. nov., holotype (IEBR_LS_Anauchen.001H) **A** protoconch surface **B** teleoconch surface **C** enlarged apertural view.

**Figure 34.**
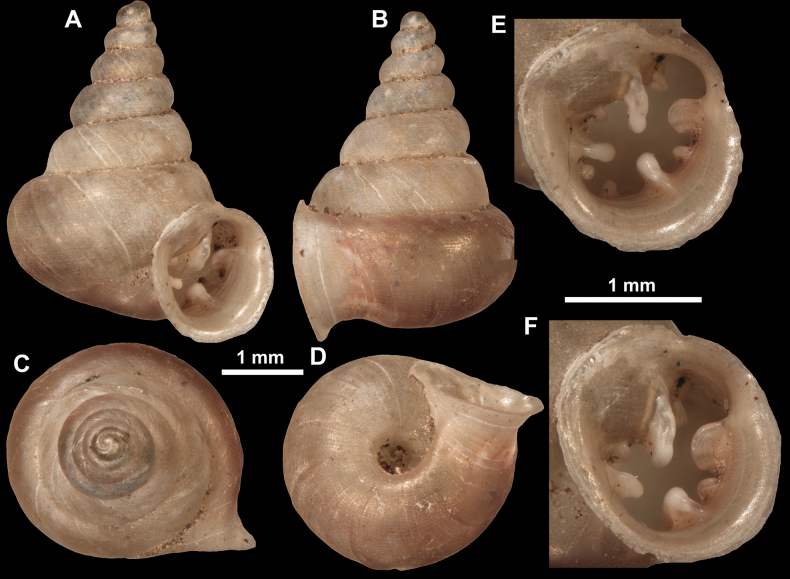
*Anauchenturritus* Gojšina, Hunyadi & Páll-Gergely, sp. nov., paratype (coll. HA) (less slender form) **A–D** shell **E** enlarged apertural view.

Figs 10P , 32 , 33 , 34 , 37

##### Type material.

***Holotype*. Vietnam** • 1 shell (SH: 4.7 mm, SW: 3.2 mm); Thanh Hoa Province, 23.7 km south from centre of Ngoc Lac, Phuc Thinh, Lang Mieng, rock above the village; 19°55.869'N, 105°22.196'E; 65 m a.s.l.; 13 Feb. 2020; A. Hunyadi, H.V. Luong, J.U. Otani & S.V. Pham leg.; IEBR_LS_Anauchen.001H. ***Paratypes*. Vietnam** • 1 shell; same data as for holotype; CUMZ 14432 • 79 shells; same data as for holotype; coll. HA • 1 shell; same data as for holotype; coll. VG • 29 shells; Thanh Hoa Province, Nhur Thanh District, Hai Van, Hang Lo Cao Khang Chien, near cave; 19°37.079'N, 105°34.629'E; 22 m a.s.l.; 14 Feb. 2020; A. Hunyadi, H.V. Luong, J.U. Otani & S.V. Pham leg.; coll. HA.

##### Additional material examined.

**Vietnam** • 13 shells (juveniles, not paratypes); same data as for holotype; coll. HA • 7 shells (juveniles/ damaged, not paratypes); Thanh Hoa Province, Nhur Thanh District, Hai Van, Hang Lo Cao Khang Chien, near cave; 19°37.079'N, 105°34.629'E; 22 m a.s.l.; 14 Feb. 2020; A. Hunyadi, H.V. Luong, J.U. Otani & S.V. Pham leg.; coll. HA.

##### Type locality.

Vietnam, Thanh Hoa Province, 23.7 km south from centre of Ngoc Lac, Phuc Thinh, Lang Mieng, rock above the village; 19°55.869'N, 105°22.196'E; 65 m a.s.l.

##### Diagnosis.

Shell conical, consisting of 7.5–8 whorls. Teleoconch densely spirally striated. Last whorl adnate to the penultimate. Aperture equipped with four main barriers (parietal, upper palatal, lower palatal and columellar), supra-angular and additional two smaller barriers (basal and infraparietal).

##### Description.

Shell conical to weakly concave-conical, light brown-yellowish in colour, opaque. It is consisting of 7–8 regularly increasing whorls separated by a moderately deep suture. All whorls rounded, weakly convex. Protoconch lighter than the rest of the shell, slightly translucent, consisting of ~ 1.25–1.5 weakly spirally striated whorls. Teleoconch finely and densely spirally striated, especially on the last ~ 3 whorls. Spiral striae strong, irregularly spaced, curved, > 40 on the last whorl in a standard view. The space between two spiral striae ranges from the width of one to the width of two or even three spiral striae. These striae are occasionally crossed by a few radial whitish streaks, randomly positioned, but mostly present on the last and the penultimate whorl. Last whorl adnate to the penultimate, slightly ascending (~ 5 ° compared to the shell axis) and making the apertural profile slightly opisthocline to the shell axis. Peristome thick, the same colour as the rest of the shell, expanded and not reflected, very finely pitted. It is especially strongly expanded at the parietal side where it leans against the penultimate whorl and forms a thick callus. Aperture equipped with four main barriers (parietal, upper palatal, lower palatal, and columellar) and several smaller ones. Parietal lamella very strong and high, directed towards the lower palatal plica. Upper palatal plica very strong, developed equally as parietal lamella and slightly leaning towards the lower palatal plica. Parietal lamella and upper palatal plica are closely positioned, leaving a narrow spacing between them. Lower palatal plica is strong but weaker than the upper palatal, directed towards the embayment between columellar and parietal lamellae. Columellar lamella slightly oblique, directed towards the lower palatal plica and developed to the same extent. Between these main barriers, smaller number of minor ones are also observed. Supra-angular lamella present, small, and short, leaning towards the palatal wall. Interpalatal plica sometimes present, positioned rather obliquely. Subcolumellar lamella present as weak or absent. Basal plica present, short. A very slight thickening (swelling) like infraparietal lamella is present. This lamella is sometimes much stronger. Surface of all apertural barriers is finely granulated. Sinulus large, distinctly separated from the rest of the aperture due to the strong angulo-parietal lamella and upper palatal plica. Umbilicus narrow, measuring ~ 1/8 of the shell width and with almost perpendicular walls, not showing previous whorls.

##### Differential diagnosis.

This species is similar to various congeners such as *A.rochebruni* and *A.messageri*. However, it can be clearly separated from them by the separate lamellae on the parietal side (angular and parietal) and a generally more elongated shell with slightly more pronounced spiral threads. *Anauchenturritus* sp. nov. has spiral striation, more apertural barriers and a wider umbilicus than *A.jokaii* sp. nov. See also under *H.annamiticum*.

##### Measurements

**(in mm, *n* = 5).**SH = 4.05–4.7; SW = 2.72–3.43; AH = 1.54–1.88; AW = 0.7–0.87.

##### Etymology.

This species is named after the tower-shaped (turreted) shell.

##### Distribution.

This species is known from two localities in Thanh Hoa Province, Vietnam.

##### Remarks.

We have observed significant intraspecific variability in this species. Shell can be high conical or conical (even weakly concave-conical). Some specimens (Fig. 32) have only six apertural barriers (supra-angular, parietal, columellar, basal, upper, and lower palatal) while others can have up to nine (including interpalatal, infraparietal and subcolumellar) (Fig. 34). Colouration is usually pale brown but colourless specimens also occur.

#### ﻿ Anauchen utaithaniensis

Taxon classificationAnimaliaStylommatophoraHypselostomatidae

Panha, 2002

60D512D3-B2A7-58A6-B131-4737B13FC2FA

**Figure 35.**
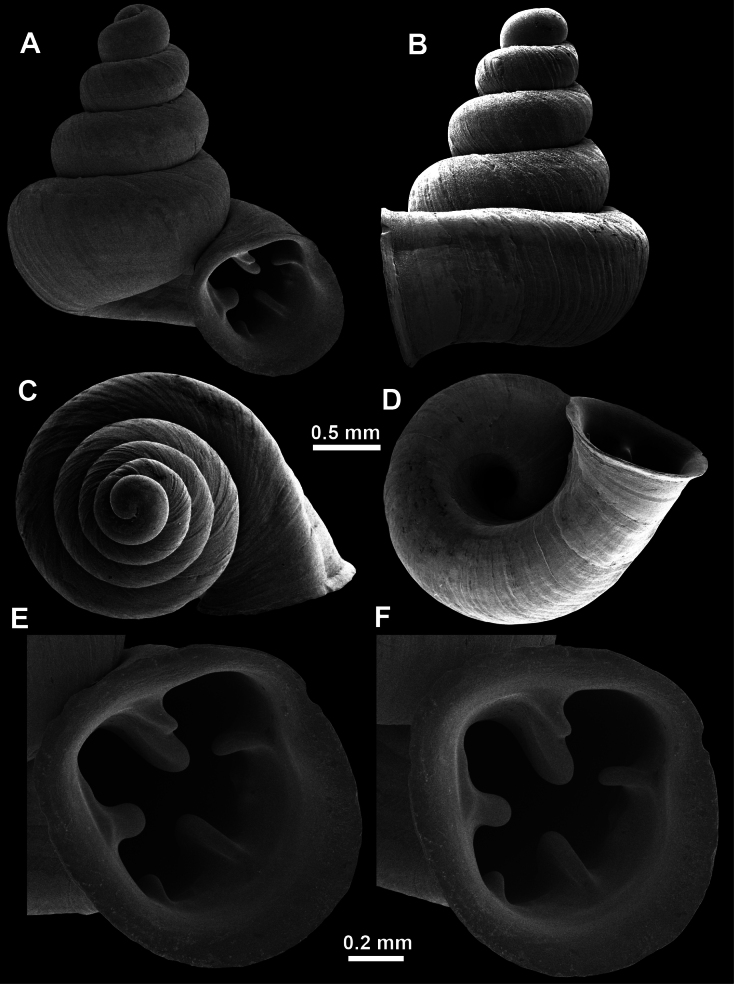
*Anauchenutaithaniensis*, holotype (CUMZ ver. 041) **A–D** shell **E, F** enlarged apertural views.

**Figure 36.**
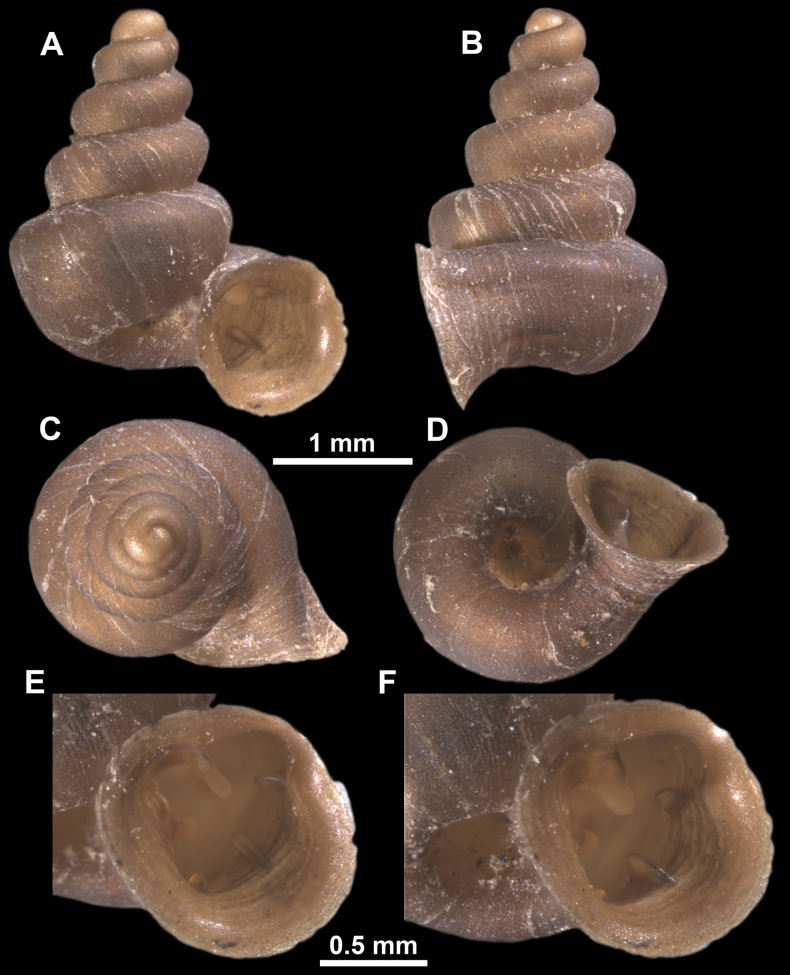
*Anauchenutaithaniensis*, paratype (SMF 331454) **A–D** shell **E, F** enlarged apertural views.

**Figure 37.**
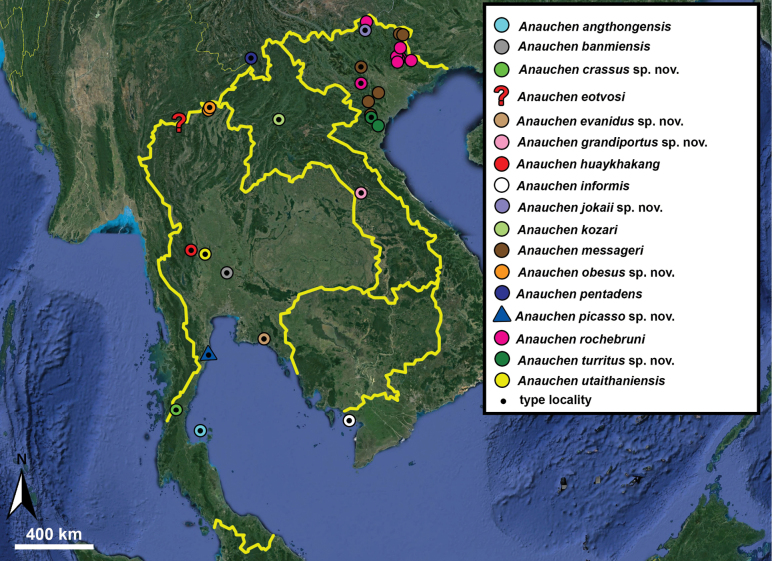
Distribution of species belonging to the genus *Anauchen*.

Figs 10Q , 35 , 36 , 37

Anauchen utaithaniensis Panha in Burch & Panha, 2002: 244–245, fig. 3.
Anauchen utaithaniensis — Tongkerd et al. 2004: 143, fig. 2; Panha and Burch 2008: 53–54, fig. 49; Dumrongrojwattana and Wongkamhaeng 2024: 323, fig. 7.

##### Type material examined.

**Thailand** • holotype; 1997; S. Panha leg.; CUMZ ver. 041 • 2 paratypes; same data as for the holotype; SMF 331454.

##### Type locality.

“Patavi Mountain, Utaithani Province, 15°28'20"N, 99°45'17"E, 70 meters elevation…” (Thailand).

##### Diagnosis.

This species is most similar to *A.huaykhakang* and *A.banmiensis* but they are both spirally striated. *Anauchenangthongensis* is also spirally striated, has it last whorl regularly rounded and has a narrower umbilicus.

##### Distribution.

This species is known only from the type locality.

##### Remarks.

This species shows intra-populational variation of the angulo-parietal lamella. The angulo-parietal lamella of the holotype is bifid, while that of the paratype is almost completely fused.

#### ﻿﻿Genus Bensonella

Taxon classificationAnimaliaStylommatophoraHypselostomatidae

Pilsbry & Vanatta, 1900

674B841E-5AD9-520C-AA49-283BE0141E54

Bifidaria (Bensonella) Pilsbry & Vanatta, 1900: 591.
Boysidia (Bensonella) — Pilsbry 1917: 198.
Boysidia (Paraboysidia) Pilsbry, 1917: 174, 201.

##### Remarks.

We have divided the genus *Bensonella* in two species groups:

Group 1: *Bensonellaplicidens* group which includes the majority of species. This group can be recognised by the combination of the following traits: i) a distinct palatal tubercle on the palatal lip (Fig. 9); ii) three barriers on the parietal side (angular, parietal, and infraparietal) (Fig. 9). Several species within the *Bensonellaplicidens* group are not typical because they either lack a palatal tubercle or some of the three barriers on the parietal side (see remarks sections under those species).

Group 2: *Bensonellawangviangensis* species group includes only four species (*B.wangviangensis*, *B.cardiostoma* sp. nov., *B.fracta* sp. nov., and *B.mitochondria* sp. nov.). This group is recognised by the combination of the following traits: i) triangular-conical shell shape; ii) angular lamella as strong as the parietal or stronger, fully reaching the peristome; iii) palatal tubercle not typical but clearly represents a slightly discontinued part of the upper palatal plica (thus, it is of more lamella-like than tubercle-like). This part of the upper palatal plica is most probably homologous to the palatal tubercle found in other *Bensonella* species; iv) angular lamella and palatal tubercle are situated close together leaving a relatively narrow canal to the sinulus.

**Figure 38.**
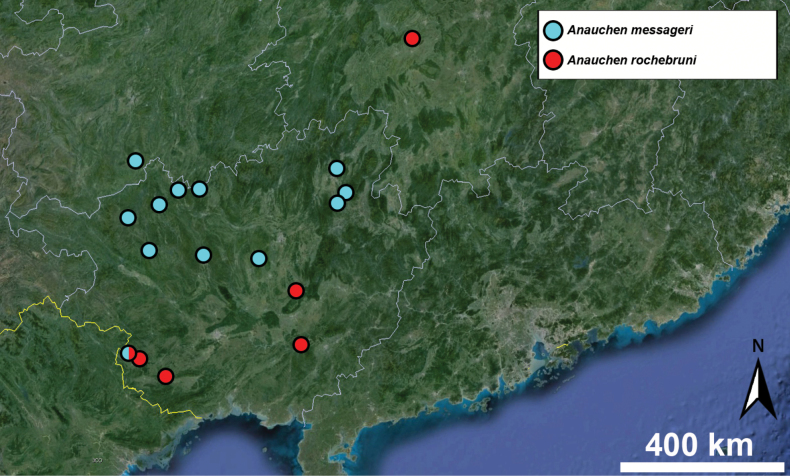
Distribution of *A.messageri* and *A.rochebruni* in China.

###### ﻿1. *Bensonellaplicidens* group

**Diagnosis.** This species group is characterised by a distinct palatal tubercle on the peristome. as well as three barriers on the parietal side (angular, parietal, and infraparietal).

**Remarks.** The group includes 32 species distributed from eastern India all the way to northern Vietnam but also present in Japan, China, Taiwan, and south to Indonesia.

#### ﻿ Bensonella alycaeus

Taxon classificationAnimaliaStylommatophoraHypselostomatidae

Gojšina, Hunyadi & Páll-Gergely sp. nov.

4A0A2A94-98A5-56FD-8FEB-6C1E31E2371A

https://zoobank.org/9CC097A5-130F-4F27-B765-BF8C9D194893

**Figure 39.**
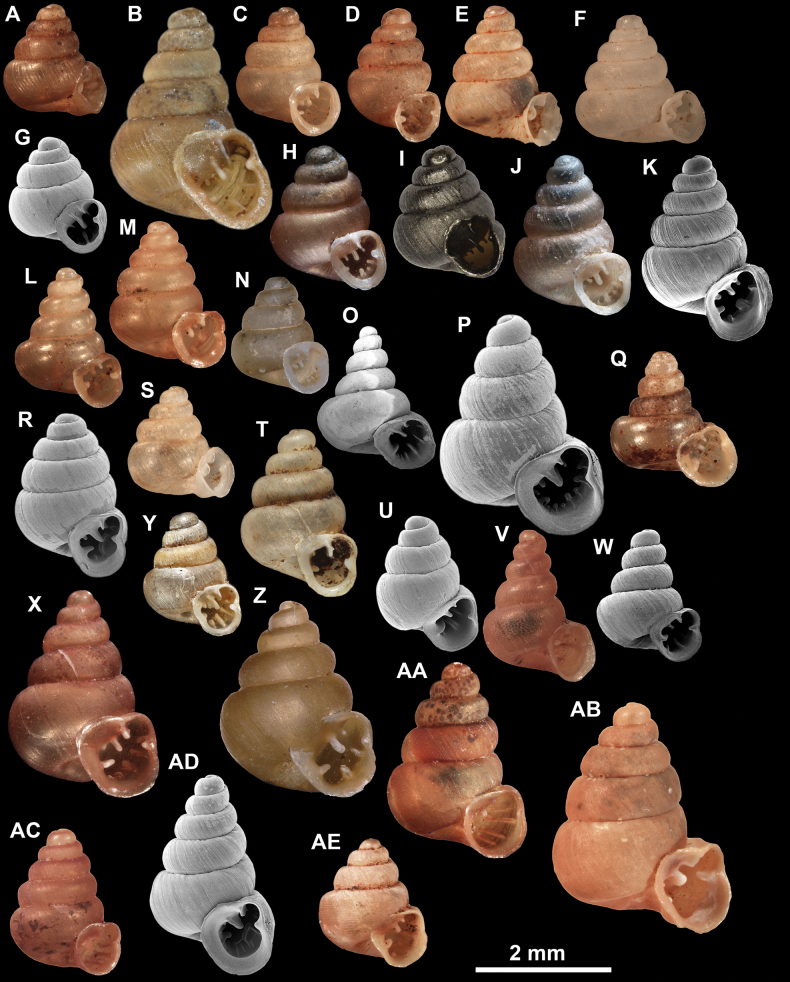
Synoptic view of the species belonging to *Bensonellaplicidens* group **A***B.alycaeus* sp. nov. **B***B.boettgeri***C***B.cristatissima* sp. nov. **D***B.dha* sp. nov. **E***B.dracula* sp. nov. **F***B.exploda* sp. nov. **G***B.geminounca***H***B.hooki***I***B.karoensis***J***B.lakainguta***K***B.lophiodera***L***B.microdentata* sp. nov. **M***B.mirabilis* sp. nov. **N***B.montawa* sp. nov. **O***B.multidentata* sp. nov. **P***B.multihami***Q***B.nitens* sp. nov. **R***B.nordsiecki***S***B.obex* sp. nov. **T***B.pahpetensis***U***B.palatotridens***V***B.pangmapaensis***W***B.paviei***X***B.perfecta* sp. nov. **Y***B.plicidens***Z***B.sericata* sp. nov. **AA***B.serrata* sp. nov. **AB***B.spelaea* sp. nov. **AC***B.spinosa* sp. nov. **AD***B.taiyaiorum***AE***B.tamphathai*.

**Figure 40.**
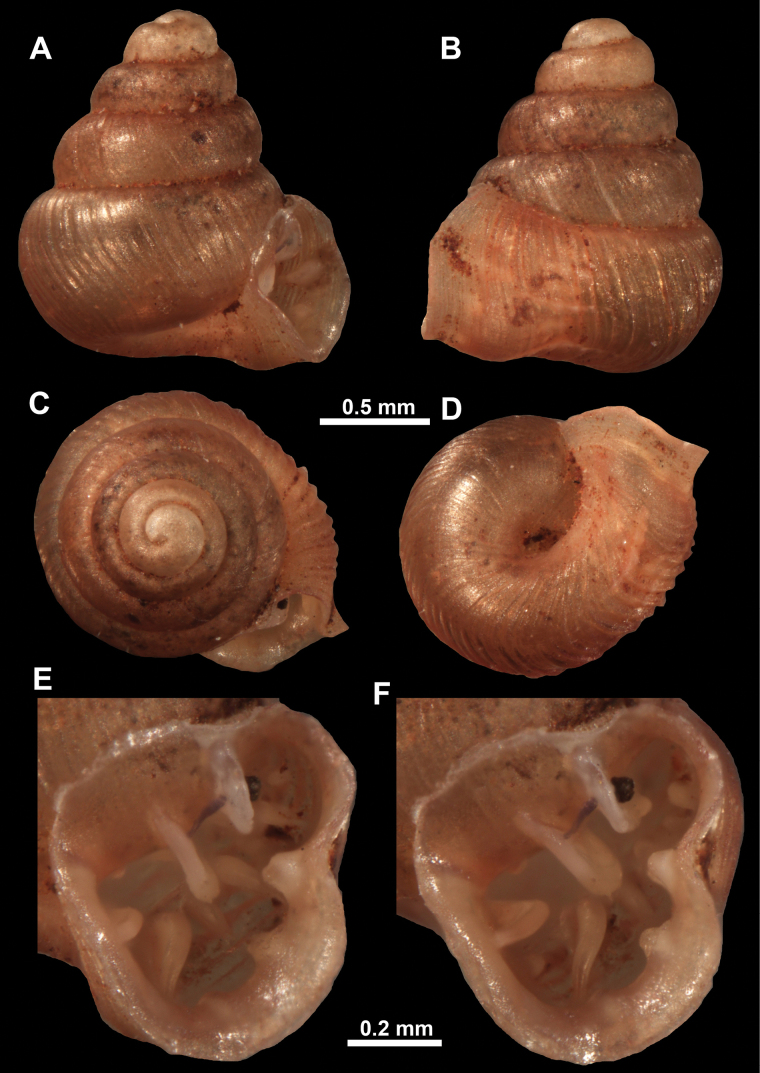
*Bensonellaalycaeus* Gojšina, Hunyadi & Páll-Gergely, sp. nov., holotype (UF 347144) **A–D** shell **E, F** enlarged apertural views.

**Figure 41.**
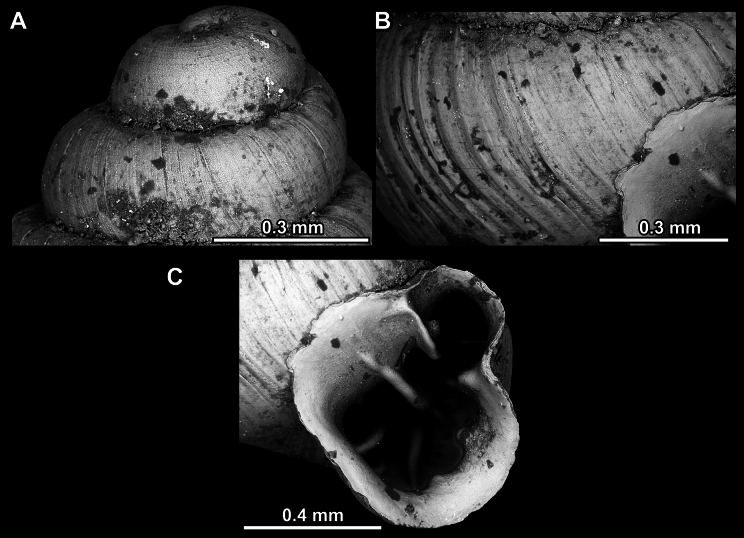
SEM imaging of *Bensonellaalycaeus* Gojšina, Hunyadi & Páll-Gergely, sp. nov., holotype (UF 347144) **A** protoconch surface **B** teleoconch surface **C** enlarged apertural view.

Figs 39A , 40 , 41 , 100

##### Type material.

***Holotype*. Thailand** • 1 shell (SH: 1.6 mm, SW: 1.3 mm); Chiang Rai Province, Doi Thung; 20°20.533'N, 99°50.350'E; 1320 m a.s.l.; 08 May 1988; F.G. Thompson leg. UF 347144. ***Paratypes*. Thailand** • 1 shell; same data as for holotype; CUMZ 14433 • 7 shells; same data as for holotype; UF 591340 • 1 shell; Chiang Rai Province, Doi Tung, 50 meters before Wat Phra That Doi Tung, vicinity of the parking lot; 20°19.563'N, 99°49.990'E; 1350 m a.s.l.; 12 Feb. 2015; A. Hunyadi leg.; coll. HA.

##### Additional material examined.

**Thailand** • 1 shell (juvenile, not paratype); same data as for holotype; UF 583721.

##### Type locality.

Thailand, Chiang Rai Province, Doi Thung; 20°20.533'N, 99°50.350'E; 1320 m a.s.l.

##### Diagnosis.

*Bensonella* species with a triangular-conical shell and strongly radially ribbed last whorl, especially near the aperture. Palatal plicae very strong. Umbilicus very narrow and elongated, dot-like.

##### Description.

Shell triangular, conical-ovoid, brown, consisting of 4.5–5 convex, rounded whorls separated by a moderately deep suture. Protoconch spirally striated (~ 12–15 spiral striae). The protoconch is initially light in colour and then regularly darkening towards the teleoconch. Teleoconch surface very finely spirally striated, striae much weaker than on the protoconch. On the penultimate whorl, relatively coarse, weak, radial growth lines are visible. They become more densely arranged and more prominent on the last whorl and especially near the aperture where they are rib-like (important for species identification!). They are crossed by very delicate and widely spaced spiral striae. Last whorl rounded and adnate to the penultimate. It is slightly ascending near the aperture (~ 20 ° compared to the shell axis), making the aperture profile weakly opisthocline. Peristome lighter than the rest of the shell, not much expanded and not reflected. There is a slight thickening behind the peristome in form of a cervical crest. Aperture equipped with 10–12 barriers. Parietal lamella is the strongest in the aperture, long and curved, sinuated in its middle part. Angular lamella long, thin, and continuous, reaching the peristome. There are four main palatal plicae (upper palatal, two interpalatals, and a lower palatal) and usually one smaller suprapalatal plica. Upper palatal plica strong, highest in its middle part and getting lower towards the peristome. Interpalatal plicae similar to each other and the upper palatal. Lower palatal plica is the strongest. There is a strong palatal tubercle sitting on the palatal lip of the peristome, sometimes of almost rectangular shape. Peristome is distinctly sinuated behind this tubercle. Below the palatal tubercle, there are usually one or two more tubercle-like swellings which are of unknown homology. Basal plica very weak and short. Columellar lamella almost horizontal and slightly weaker than upper and lower palatal plicae. Infraparietal lamella similar to the basal plica. Surface of all apertural barriers is finely granulated. Sinulus small, narrow, and distinctly separated from the rest of the aperture. Umbilicus very narrow, dot like and elongated.

##### Differential diagnosis.

This species is different from other congeners by the strong, rib-like radial growth lines, especially on the last whorl near the aperture and very strong palatal plicae.

##### Measurements

**(in mm, *n* = 5).**SH = 1.55–1.78; SW = 1.3–1.57; AH = 0.65–0.80; AW = 0.65–0.70.

##### Etymology.

The specific epithet refers to the cyclophoroid subfamily Alycaeinae W. T. Blanford, 1864 due to the changing rib density on the last whorl. To be used as a noun in apposition.

##### Distribution.

This species is known only from the type locality.

#### ﻿ Bensonella boettgeri

Taxon classificationAnimaliaStylommatophoraHypselostomatidae

(Möllendorff, 1897)

E6A978DA-C667-5D6D-A73A-0DD2B0B34AD4

**Figure 42.**
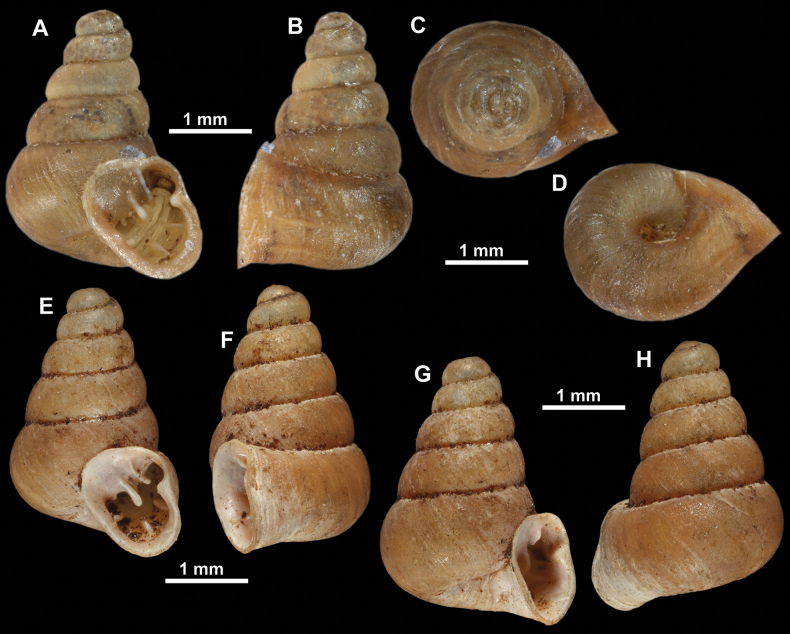
*Bensonellaboettgeri***A–D** lectotype (SMF 4616) **E–H** syntype of *B.novemdentata* (MNHN-IM-2000-33881).

**Figure 43.**
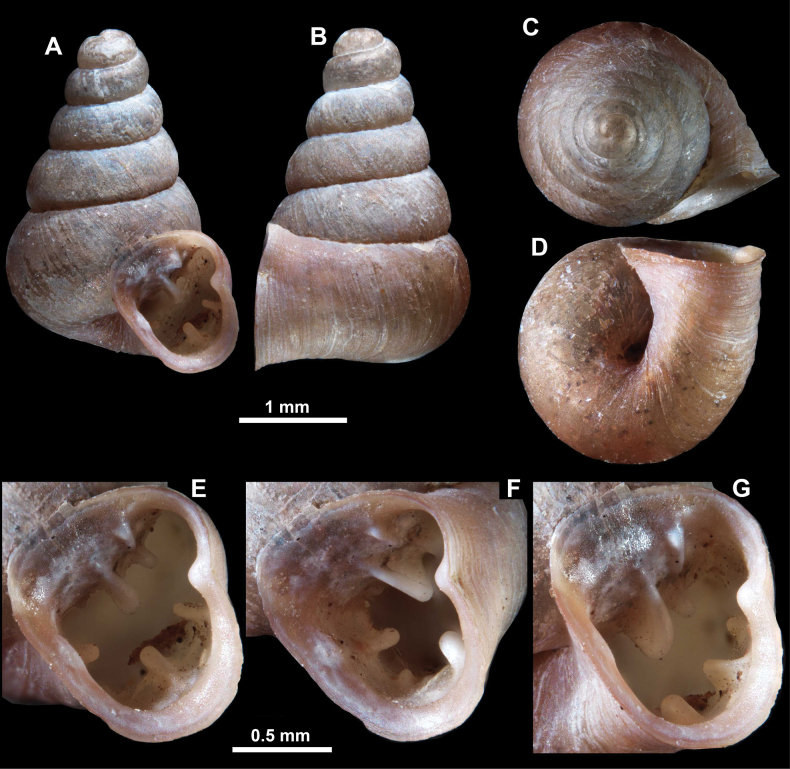
*Bensonellaboettgeri* from Naga hills, India (NHMUK 1903.7.1.2853) **A–D** shell **E–G** enlarged apertural views.

Figs 39B , 42 , 43 , 60

Boysidia boettgeri Möllendorff, 1897: 70.
Boysidia (Paraboysidia) boettgeri — Pilsbry 1917: 208–209, pl. 34, figs 7, 8.
Paraboysidia boettgeri — van Benthem Jutting 1950: 38; van Benthem Jutting 1952: 318; Zilch 1984: 164; Maassen 1999: 123.
Boysidia novemdentata Saurin, 1953: 115–116, fig. 1, pl. 4, fig. 4a–c. syn. nov.
Boysidia novemdentata — Inkhavilay et al. 2019: 59, fig. 26B.
Bensonella novemdentata — Inkhavilay and Sutcharit 2024: 444, fig. 4.

##### Type material examined.

**Indonesia** • lectotype of *B.boettgeri*; W Java; collector unknown; SMF 4616. **Laos** • 1 syntype of *B.novemdentata*; Pah Hia, Laos; collector unknown; MNHN-IM-2000-33881.

##### Additional material examined.

**India** • 6 shells; Nagaland, Naga hills; UF 00112290/5, UF 179749/1 • 3 shells; Eastern Naga Hills; W. Doherty leg.; NHMUK 1903.7.1.2853.

##### Type localities.

Java, Indonesia (*B.boettgeri*); “environs du village méo de Pah Hia, à 100 kilomètres au Sud de Xieng-Khouang, chef-lieu de la province du Tran Ninh, Laos” (probably refers to Ban Namthong, Longchaeng District, Xaisomboun Province, Laos, see Páll-Gergely et al. 2016) (*B.novemdentata*).

##### Differential diagnosis.

Even though there are no peculiarities regarding the apertural dentition of this species, it can be separated from all its congeners by the far most triangular, elongated shell and relatively shallow suture.

##### Distribution.

This species is described from Java, Indonesia, but is also known from its synonym (*B.novemdentata*) in Laos. It has also been found in E India.

##### Remarks.

*Boysidianovemdentata* was described from northeastern Laos, > 2800 km from the type locality of *B.boettgeri*. However, these two species are virtually identical in shell morphology. See the discussion section.

#### ﻿ Bensonella cristatissima

Taxon classificationAnimaliaStylommatophoraHypselostomatidae

Gojšina, Hunyadi & Páll-Gergely sp. nov.

6AF9CBF5-1F87-58D7-ADBE-188AF977CD68

https://zoobank.org/F18FAD0C-C667-4654-97DD-DCCDFBB994DA

**Figure 44.**
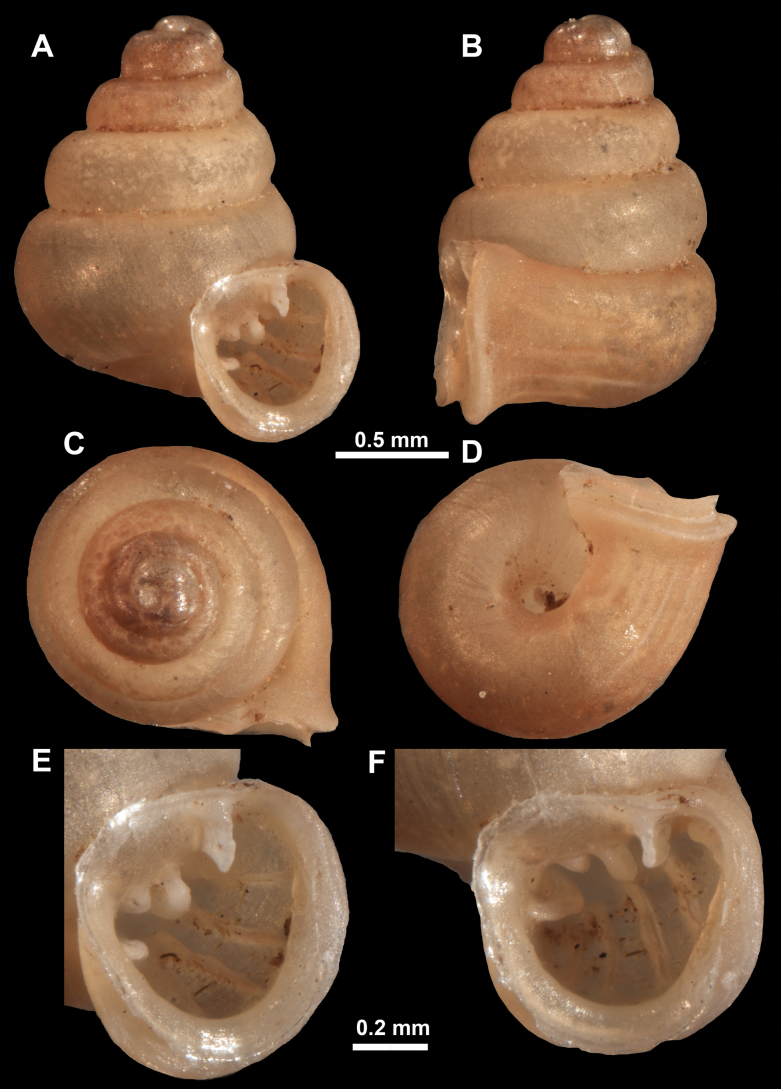
*Bensonellacristatissima* Gojšina, Hunyadi & Páll-Gergely, sp. nov., holotype (CUMZ 14434) **A–D** shell **E, F** enlarged apertural views.

**Figure 45.**
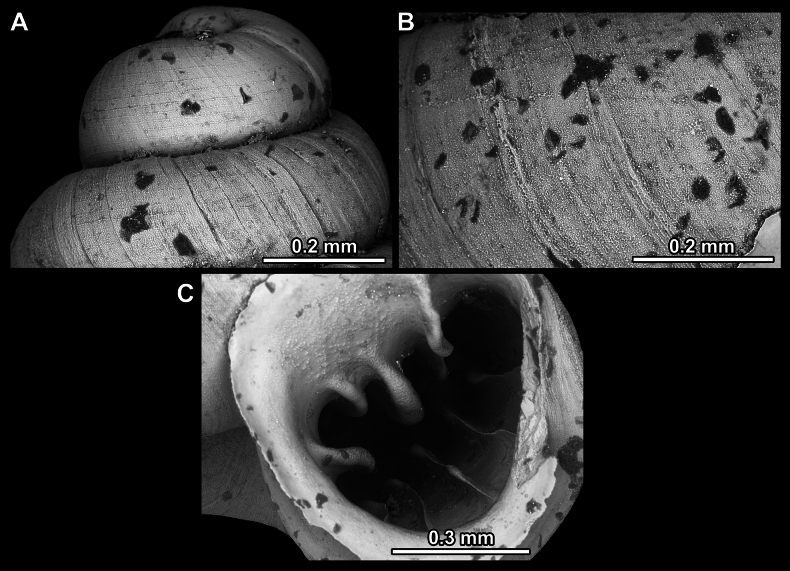
SEM imaging of *Bensonellacristatissima* Gojšina, Hunyadi & Páll-Gergely, sp. nov., holotype (CUMZ 14434) **A** protoconch surface **B** surface of the last whorl **C** apertural view.

Figs 39C , 44 , 45 , 100

##### Type material.

***Holotype*. Myanmar** • 1 shell (SH: 1.77 mm, SW: 1.51 mm); Shan State, Taunggyi, mountainside above Aye Say Tee, Dragon Cave; 20°47.489'N, 97°3.036'E; 1380 m a.s.l.; 08 Oct. 2018; A. Hunyadi, K. Okubo & J.U. Otani leg.; CUMZ 14434. ***Paratypes*. Myanmar** • 95 shells; same data as for holotype; coll. HA • 1 shell; same data as for holotype; coll. VG • 27 shells Shan State, west-southwest from Taunggyi, Montawa cave; 20°45.282'N, 97°1.057'E; 1260 m a.s.l.; 05 Oct. 2018; A. Hunyadi, K. Okubo & J.U. Otani leg.; coll. HA • 10 shells; Shan State, Hopong centre ca 22 km towards Namsang, Hkoche, near Htem Sann Cave; 20°49.084'N, 97°20.119'E; 1240 m a.s.l.; 06 Oct. 2018; A. Hunyadi, K. Okubo & J.U. Otani leg.; coll. HA.

##### Additional material examined.

**Myanmar** • 4 shells (3 juveniles and 1 broken, not paratypes); same data as for holotype; coll. HA.

##### Type locality.

Myanmar Shan State, Taunggyi, mountainside above Aye Say Tee, Dragon Cave; 20°47.489'N, 97°3.036'E; 1380 m a.s.l.

##### Diagnosis.

A small *Bensonella* species with fine, finely dimpled teleoconch sculpture, rounded last whorl adnate to the penultimate, and a strong, sharp cervical crest right behind the expanding peristome. Palatal plicae long and equally developed; umbilicus very narrow to almost closed.

##### Description.

Shell triangular, slightly conical-ovoid, brownish or yellowish, weakly glossy. It is consisting of 4.5–5 regularly increasing, convex, rounded whorls separated by a deep suture. Protoconch consisting of ~ 1.5 coarsely spirally striated whorls. There are ~ 13 spiral striae on the protoconch and they are more densely arranged at the bottom of the whorl. Teleoconch surface finely dimpled (pasty) and with not numerous, weak radial growth lines. Spiral striae present initially on the teleoconch but getting lost so that the surface remains finely pasty-like or a very weak spiralling pattern might be visible (only on SEM images). Last whorl rounded, adnate to the penultimate. Right behind the peristome, there is a strong, thick, sharp, white cervical crest which is wider than the peristome and even visible behind it in the apertural view. Peristome thick and expanded but not reflected. Aperture equipped with numerous barriers, four of which are particularly strong (angular, parietal, infraparietal, and columellar), and situated closer to the peristome edge. Parietal lamella is the strongest and highest in the aperture. Angular lamella much longer than the parietal, very slender but continuous. Its inner and outer parts are higher than its middle part. Columellar lamella similar in length to the parietal only slightly weaker. This lamella is almost horizontal but sometimes slightly curved towards the palatal plicae. Infraparietal lamella is roughly half as strong as the parietal. Palatal plicae equidistant, all of equal height and length, altogether four (one upper palatal, two interpalatals, and a lower palatal). They are all high at their inner parts and then regularly lowering and tapering towards their outer parts. Palatal tubercle moderately strong or absent. Basal plica present, the same length as the palatals or slightly shorter. Surface of all apertural barriers is finely granulated. Sinulus small and narrow (resembling a comma sign), distinctly separated from the rest of the aperture. Umbilicus very narrow (measuring ~ 1/10 of the shell width) or even dot-like, almost closed.

##### Differential diagnosis.

See under *B.dha* sp. nov.

##### Measurements

**(in mm, *n* = 5).**SH = 1.77–1.86; SW = 1.45–1.52; AH = 0.73–0.80; AW = 0.63–0.76.

##### Etymology.

This species is named for the strong cervical crest which is the strongest among all the congeners.

##### Distribution.

This species in known from three localities in Shan State, Myanmar.

#### ﻿ Bensonella dha

Taxon classificationAnimaliaStylommatophoraHypselostomatidae

Gojšina, Hunyadi & Páll-Gergely sp. nov.

83D19BD1-1515-5B0A-A694-B46165FA96BC

https://zoobank.org/B5FFA8EB-20EE-4FE2-A8C7-F305AB8060A7

**Figure 46.**
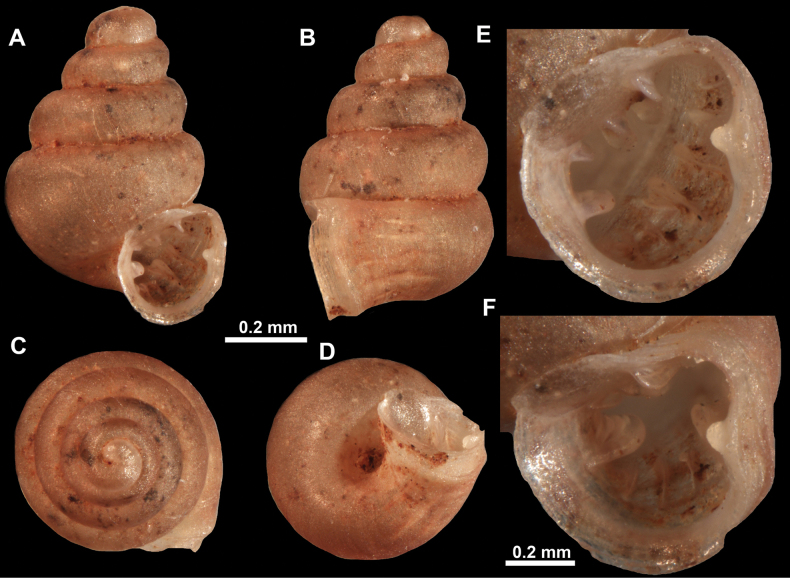
*Bensonelladha* Gojšina, Hunyadi & Páll-Gergely, sp. nov., holotype (CUMZ 14435).**A–D** shell **E, F** enlarged apertural views.

**Figure 47.**
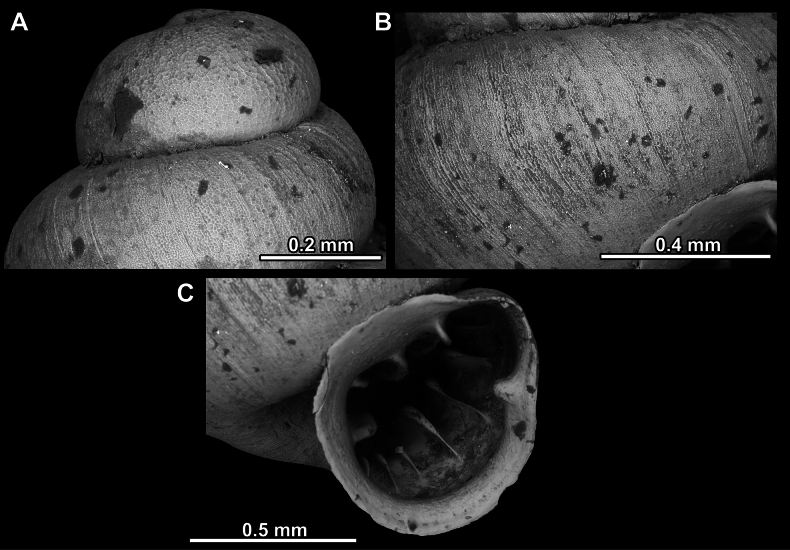
SEM imaging of *Bensonelladha* Gojšina, Hunyadi & Páll-Gergely, sp. nov., holotype (CUMZ 14435) **A** protoconch surface **B** surface of the last whorl **C** apertural view.

Figs 39D , 46 , 47 , 97

##### Type material.

***Holotype*. Myanmar** • 1 shell (SH: 1.9 mm, SW: 1.3 mm); Shan State, 5.8 km from centre of Hopong towards Namsang, left side of road #4, Hopong Spring Cave; 20°49.028'N, 97°13.469'E; 1110 m a.s.l.; 06 Oct. 2018; A. Hunyadi, K. Okubo & J.U. Otani leg.; CUMZ 14435.

***Paratypes*. Myanmar** • 34 shells; same data as for holotype; coll. HA • 1 shell; same data as for holotype; coll. VG.

##### Type locality.

Myanmar, Shan State, 5.8 km from centre of Hopong towards Namsang, left side of road #4, Hopong Spring Cave; 20°49.028'N, 97°13.469'E; 1110 m a.s.l.

##### Additional material examined.

**Myanmar** • 2 shells (juveniles, not paratypes); same data as for holotype; coll. HA • 4 shells; Shan State, 22 km from centre of Hopong towards Namsang, Htem Sann Cave; 20°49.0836'N, 97°20.1192'E; 1240 m a.s.l.; 06 Oct. 2018; A. Hunyadi, K. Okubo & J.U. Otani leg.; coll. HA • 2 shells; Shan State, 13.5 km east-southeast from centre Kalaw, Myinmati Taung; 20°35.426'N, 96°36.794'E; 1350 m a.s.l.; 03 Oct. 2018; A. Hunyadi, K. Okubo & J. U. Otani leg.; coll. HA • 11 shells; Shan State, 7.4 km from centre of Hopong towards Namsang, 5 km north on road #4, Parpant cave; 20°50.963'N, 97°14.267'E; 1170 m a.s.l.; 06 Oct. 2018; A. Hunyadi, K. Okubo & J. U. Otani leg.; coll. HA • 4 shells; Shan State, Kalaw, Shwe Oo Min Paya; 20°37.2616'N, 96°33.4735'E; 1340 m a.s.l.; 02 Oct. 2018; A. Hunyadi, K. Okubo & J.U. Otani leg.; coll. HA.

##### Diagnosis.

*Bensonella* species with conical shell which is not spirally striated. Apertural barriers numerous (angular, parietal, five palatals, one basal, one subcolumellar, one columellar, and one infraparietal) and with additional, strong palatal tubercle. Aperture well rounded.

##### Description.

Shell conical to conical-ovoid, light brownish and weakly glossy, consisting of 4.25–5.5 convex rounded whorls separated by a deep suture. Protoconch roughly pitted, sometimes with a spiralling pattern and consisting of ~ 1.5–1.75 whorls. Teleoconch with fine pasty structure, not spirally striated but with very fine radial growth lines. Last whorl adnate to the penultimate, aperture profile only slightly prosocline to the shell axis due to the slightly descending last whorl (~ 15 ° compared to the shell axis). Peristome slightly lighter than the rest of the shell, expanded but not reflected. It is particularly expanded on the parietal side where it forms a weak callus which is leaned on the penultimate whorl. Aperture equipped with moderately strong, numerous barriers. Parietal lamella moderately strong and high. Angular lamella longer but overall weaker than the parietal. It is higher in its inner part and regularly tapering towards the peristome, which is not reached. There are two or three very weak barriers inside the sinulus. Palatal wall equipped with five long barriers (suprapalatal, upper palatal, interpalatal, lower palatal, and infrapalatal) and a single strong palatal tubercle sitting on the edge of the palatal lip. Suprapalatal plica weak, not much stronger than the sinulus plicae. Upper palatal plica only slightly stronger than the suprapalatal. Interpalatal moderate. Lower palatal is the strongest among all the palatal plicae. Infrapalatal weak and shorter than others. Basal plica developed to the same extent as the infrapalatal. Subcolumellar lamella weak and short (nearly the same as the basal and infrapalatal). Columellar lamella strong as the lower palatal, almost horizontal. Infraparietal lamella moderate, ~ ½ as strong as the columellar. Surface of all apertural barriers is finely granulated. Sinulus small and not very distinctly separated from the rest of the aperture. Umbilicus very narrow, dot-like.

##### Differential diagnosis.

This species differs from *B.cristatissima* sp. nov. by the thinner peristome, less elongated apertural barriers and by the absence of a strong and sharp cervical crest.

##### Measurements

**(in mm, *n* = 13).**SH = 1.56–2.02; SW = 1.14–1.53; AH = 0.64–0.78; AW = 0.64–0.73.

##### Etymology.

Named after the Burmese word for sword/knife (*dha*) referring to the blade-like anterior part of the palatal plicae. To be used as a noun in apposition.

##### Distribution.

This species is known from five localities in Shan State, Myanmar.

##### Remarks.

We have observed some intraspecific variability between the populations. The population from Shwe Oo Min Paya is the smallest and with the most ovoid shell. While the populations from Myinmati Taung and Parpant cave were the most triangular with slightly shallower sutures.

#### ﻿ Bensonella dracula

Taxon classificationAnimaliaStylommatophoraHypselostomatidae

Gojšina, Hunyadi & Páll-Gergely sp. nov.

EDE34498-03FC-5A39-BA15-190A618A5B78

https://zoobank.org/840544DA-EE6E-4FA8-B50A-867E31CA6BBA

**Figure 48.**
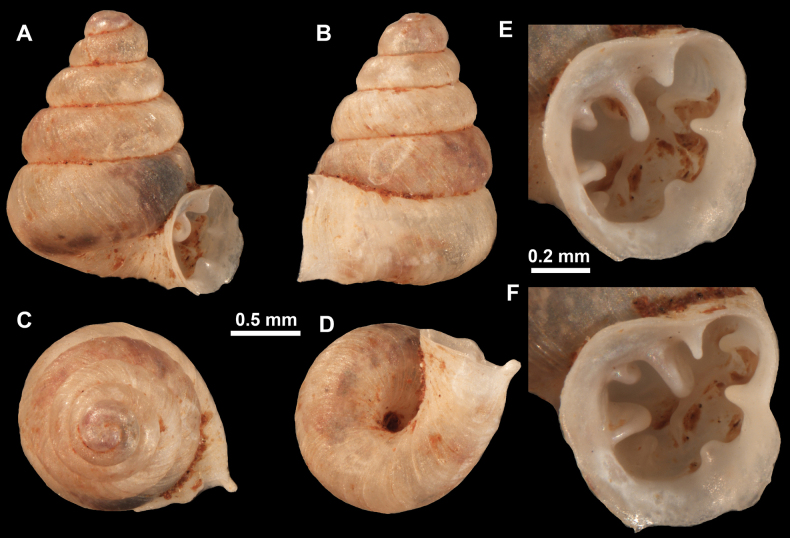
*Bensonelladracula* Gojšina, Hunyadi & Páll-Gergely, sp. nov., holotype (CUMZ 14436) **A–D** shell **E, F** enlarged apertural views.

**Figure 49.**
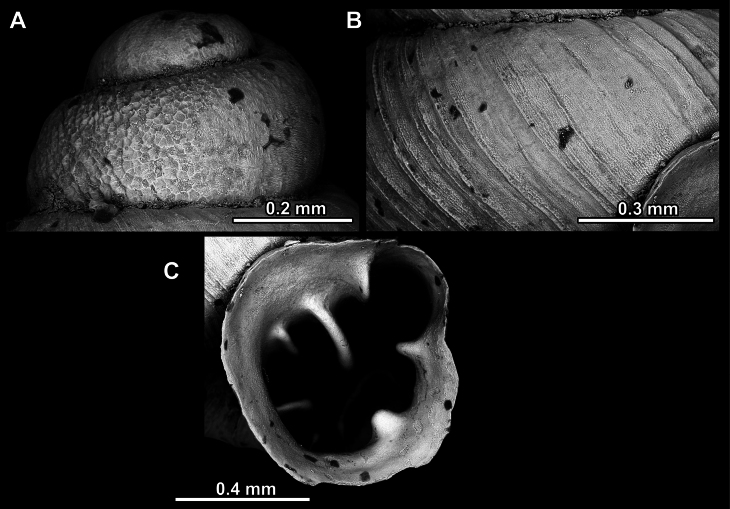
SEM imaging of *Bensonelladracula* Gojšina, Hunyadi & Páll-Gergely, sp. nov., holotype (CUMZ 14436) **A** protoconch surface **B** surface of the last whorl **C** apertural view.

**Figure 50.**
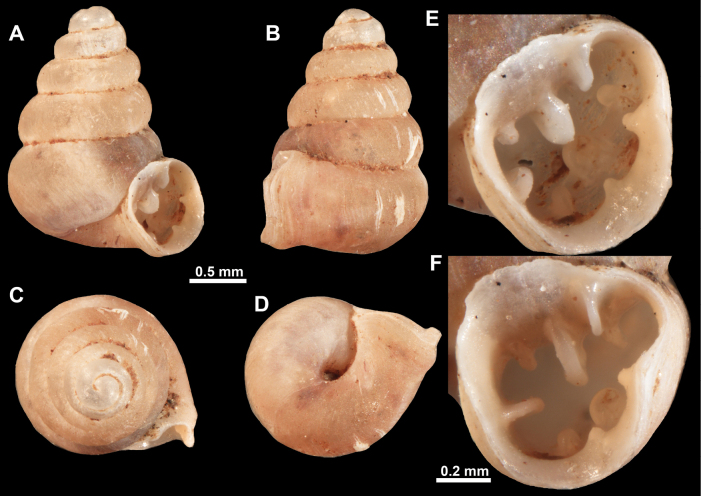
*Bensonelladracula* Gojšina, Hunyadi & Páll-Gergely, sp. nov. from Pinlaung (coll. HA) **A–D** shell **E, F** enlarged apertural views.

**Figure 51.**
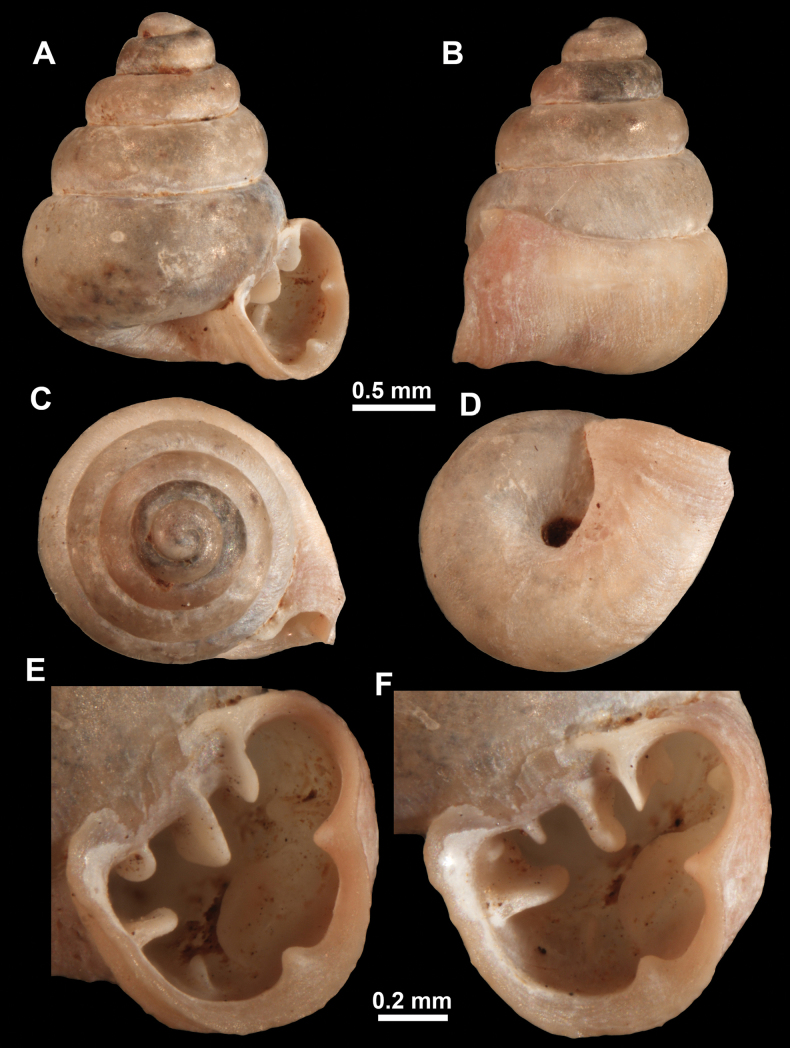
*Bensonelladracula* Gojšina, Hunyadi & Páll-Gergely sp. nov. from Phruno river cave **A–D** shell **E, F** enlarged apertural views.

Figs 39E , 48 , 49 , 50 , 51 , 97

##### Type material.

***Holotype*. Myanmar** • 1 shell (SH: 2 mm, SW: 1.6 mm); Shan State, 22 km from centre of Hopong towards Namsang, Hkoche, near Htem Sann Cave; 20°49.084'N, 97°20.119'E; 1240 m a.s.l.; 06 Oct. 2018; A. Hunyadi, K. Okubo & J.U. Otani leg.; CUMZ 14436. ***Paratypes*. Myanmar** • 16 shells; same data as for holotype; coll. HA.

##### Type locality.

Myanmar, Shan State, 22 km from centre of Hopong towards Namsang, Hkoche, near Htem Sann Cave; 20°49.084'N, 97°20.119'E; 1240 m a.s.l.

##### Additional material examined.

**Myanmar** • 3 shells; Shan State, Pinlaung centre SSW 11 km – Laneli Bridge, Nam Pam, near “Upper Spider Cave”; 20°2.114'N, 96°45.728'E; 1420 m a.s.l.; 04 Oct. 2018; A. Hunyadi, K. Okubo & J.U. Otani leg.; coll. HA • 1 shell; Myanmar, Kayah State, Hpruso district, Maw Thi Do Village, entrance of Phruno river cave; 19°22.744'N, 97°2.570'E; 12 Feb. 2019; J. Grego leg.; coll. JG.

##### Diagnosis.

*Bensonella* species with triangular, conical-ovoid shell and rounded whorls devoid of spiral striation. There are two palatal tubercles in front of a strong and frontally concave transversal plica. A single, strong basal plica present.

##### Description.

Shell triangular, conical-ovoid, consisting of 5–5.5 rounded, convex whorls separated by a deep suture. Protoconch consisting of ~ 1.25–1.5 roughly pitted whorls. Teleoconch devoid of spiral striation but with some fine radial growth lines, otherwise smooth, pasty. Last whorl adnate to penultimate. Aperture profile slightly opisthocline to the shell axis due to the very slightly ascending last whorl (~ 5 ° compared to the shell axis). In this view, a weak cervical crest could be observed behind the peristome. Peristome thick, expanded (especially on the parietal side where it forms a thick callus) but not reflected. Sinulus small and distinctly separated from the rest of the aperture. Aperture equipped with nine barriers (angular, parietal, upper palatal, transversal, two palatal tubercles, basal, columellar, and infraparietal). Parietal lamella very strong and high, curved and blade-like. Angular lamella much weaker and lower but longer, reaching the expanding peristome. Upper palatal plica moderate, higher in its middle parts. Transversal plica strong and with its frontal surface concave. In front of the transversal plica, there are two strong palatal tubercles. The upper one (usually present in all *Bensonella* species) is narrower and more acute. The lower palatal tubercle is wider and blunter. Basal plica present, short and ~ ½ as strong as the columellar lamella. The latter is strong, wide, and almost horizontal. Infraparietal lamella as strong as the basal plica. Surface of all apertural barriers is finely granulated. Umbilicus very narrow and slightly elongated, dot-like.

##### Differential diagnosis.

See under *B.nordsiecki*, *B.obex* sp. nov. and *B.taiyaiorum*.

##### Measurements

**(in mm, *n* = 6).**SH = 1.80–2.21; SW = 1.44–1.80; AH = 0.64–0.96; AW = 0.62–0.94.

##### Etymology.

Two strong palatal tubercles of this species resemble teeth of a vampire. The specific epithet refers to the iconic vampire, Count Dracula. To be used as a noun in apposition.

##### Distribution.

This species is known from the surroundings of Hopong and Pinlaung in Shan State, as well as Phruno river cave, Kayah State.

##### Remarks.

Specimens from Pinlaung (Shan) have a shorter transversal plica and have slightly more slender shells (Fig. 50). Specimen from the Phruno river cave in Kayah State has a more ovoid shell (Fig. 51).

#### ﻿ Bensonella exploda

Taxon classificationAnimaliaStylommatophoraHypselostomatidae

Gojšina, Hunyadi & Páll-Gergely sp. nov.

D7497534-A480-52AD-8846-965726961082

https://zoobank.org/BF362A95-806E-4676-8780-1483D1EB0190

**Figure 52.**
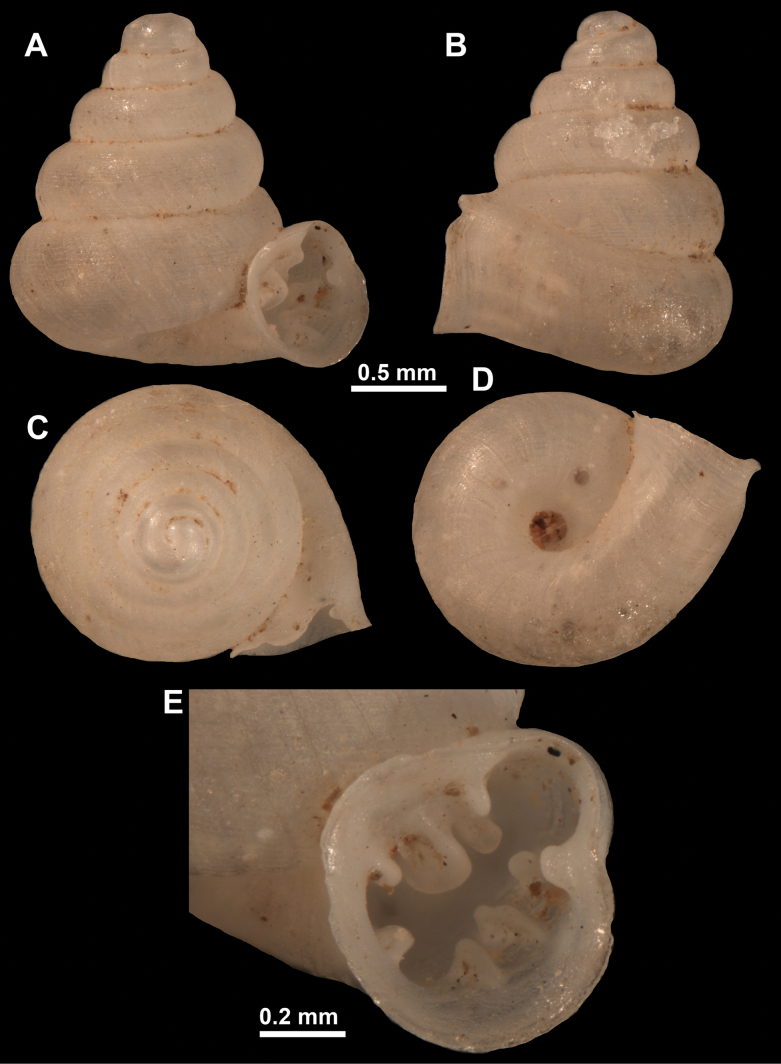
*Bensonellaexploda* Gojšina, Hunyadi & Páll-Gergely, sp. nov., holotype (CUMZ 14446) **A–D** shell **E** enlarged apertural view.

**Figure 53.**
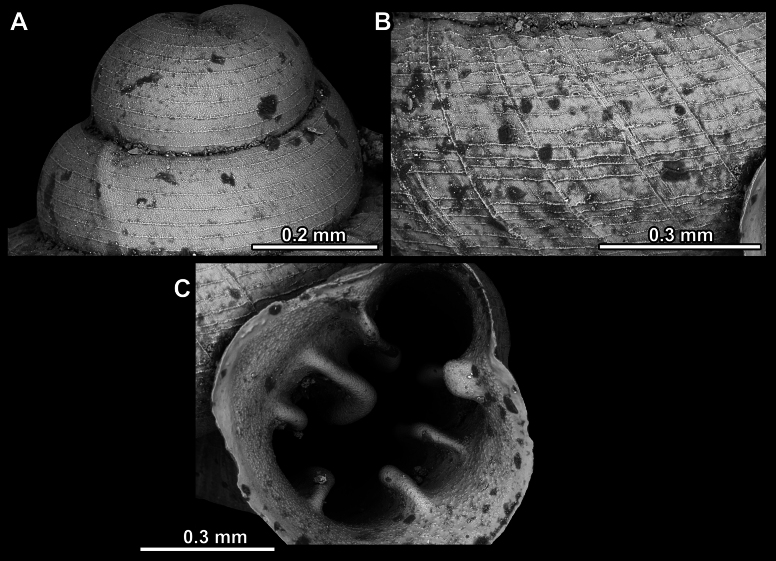
SEM imaging of *Bensonellaexploda* Gojšina, Hunyadi & Páll-Gergely, sp. nov., holotype (CUMZ 14446) **A** protoconch surface **B** surface of the last whorl **C** apertural view.

**Figure 54.**
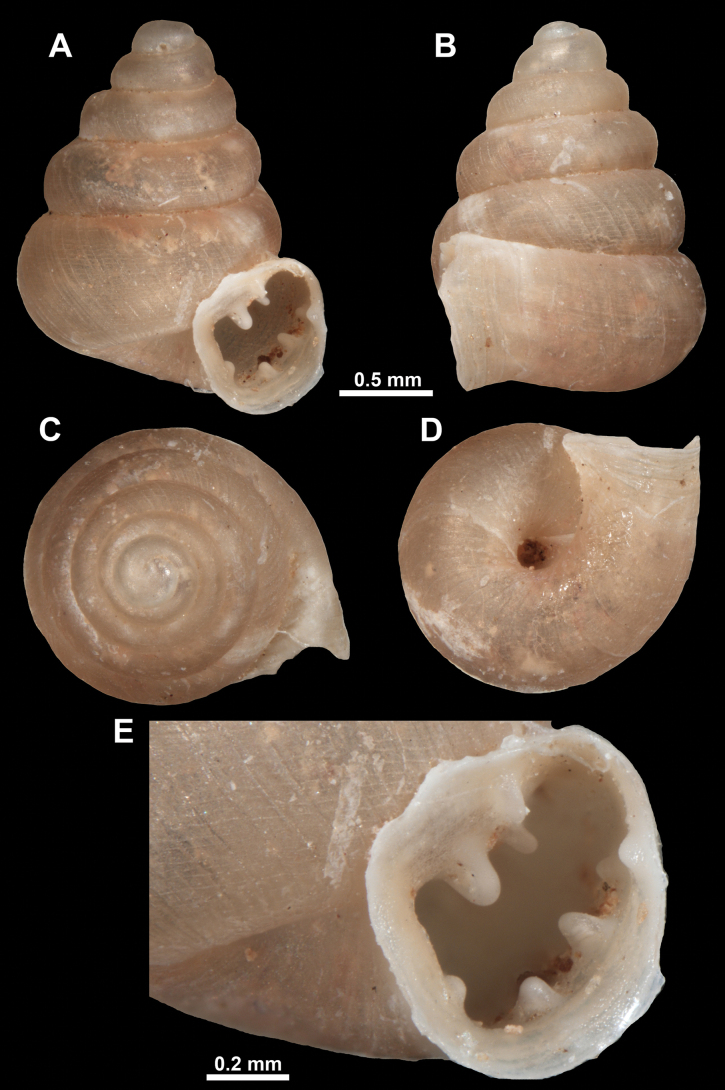
*Bensonellaexploda* Gojšina, Hunyadi & Páll-Gergely, sp. nov. from Wingabar Taung, Shan State (coll. HA) **A–D** shell **E** enlarged apertural view.

Figs 39F , 52 , 53 , 54 , 100

##### Type material.

***Holotype*. Myanmar** • 1 shell (SH: 1.8 mm, SW: 1.8 mm); Shan State, Pinlaung centre N 7.5 km, Tar Kge, near “Big Bang Cave”; 20°10.273'N, 96°47.442'E; 1540 m a.s.l.; 04 Oct. 2018; A. Hunyadi, K. Okubo & J.U. Otani leg.; CUMZ 14446. ***Paratypes*. Myanmar** • 32 shells; same data as for holotype; coll. HA • 1 shell; same data as for holotype; coll. VG.

##### Additional material examined.

**Myanmar** • 3 shells (juveniles, not paratypes); same data as for holotype; coll. HA • 149 shells; Shan State, 5.7 km south-southwest from centre of Pinlaung, Wingabar Taung; 20°4.152'N, 96°46.232'E; 1510 m a.s.l.; 04 Oct. 2018; A. Hunyadi, K. Okubo & J.U. Otani leg.; coll. HA.

##### Type locality.

Myanmar, Shan State, Pinlaung centre N 7.5 km, Tar Kge, near “Big Bang Cave”; 20°10.273'N, 96°47.442'E; 1540 m a.s.l.

##### Diagnosis.

Colourless *Bensonella* species with triangular conical-ovoid and spirally striated whorls. Last whorl slightly ascending near the aperture. Aperture equipped with eight strong barriers (angular, parietal, infraparietal, columellar, lower palatal, interpalatal, upper palatal, and a palatal tubercle). Umbilicus narrow but clearly wider than in majority of congeners.

##### Description.

Shell strongly triangular, conical-ovoid, colourless, consisting of 5–5.5 regularly growing whorls separated by a moderately deep suture. All whorls rounded, convex. Protoconch-teleoconch boundary not clearly visible due to the similar surface sculpture and colouration. Protoconch with ten coarsely and equidistantly spaced spiral striae. Teleoconch also spirally striated but additionally with coarse radial growth lines which are roughly equidistant. Spacing between two spiral striae irregular, it ranges from the width of two to the width of four spiral striae. Last whorl flat at its base, adnate to the penultimate (but peristome not fully leaning on it), slightly ascending near the aperture (~ 5 ° compared to the shell axis) making the apertural profile weakly opisthocline to the shell axis. Peristome white, expanded but not reflected. Sinulus well-rounded and distinctly separated from the rest of the aperture. Aperture equipped with eight barriers (angular, parietal, infraparietal, columellar, lower palatal, interpalatal, upper palatal, and a palatal tubercle). All barriers relatively strong. Parietal lamella is the strongest in the aperture and wavy. Angular lamella is longer than the parietal and reaching closer to the expanding peristome. It is sinuated in its middle part. Infraparietal lamella ~ 1.5 × weaker than the columellar, short. Columellar lamella strong as the lower palatal or slightly stronger. It is positioned obliquely to the shell axis, directed towards the parietal lamella. Basal plica absent. Palatal plicae all equally strong and relatively short, upper palatal is usually the shortest. Interpalatal situated halfway between the upper and lower palatal. Palatal tubercle prominent, sitting on the palatal lip and roughly in the level with upper palatal plica. Surface of all apertural barriers is finely granulated. Umbilicus narrow (but clearly wider than in the majority of congeners), measuring ~ 1/8–1/9 of the shell width.

##### Differential diagnosis.

This species differs from other congeners by the combination of the flat base of the last whorl, spirally striated whorls, and wider umbilicus.

##### Measurements

**(in mm, *n* = 5).**SH = 1.77–1.89; SW = 1.58–1.71; AH = 0.71–0.77; AW = 0.66–0.68.

##### Etymology.

This species is named *exploda* after its type locality, the “Big Bang Cave”.

##### Distribution.

This species is known only from two localities in Shan State, Pinlaung.

##### Remarks.

The specimens from Wingabar Taung (Fig. 54) share all the characters with the specimens from Big Bang Cave but have weaker apertural barriers.

#### ﻿ Bensonella fengxianensis

Taxon classificationAnimaliaStylommatophoraHypselostomatidae

(D.-N. Chen, Y.-H. Liu & W.-X. Wu, 1995)

D8ED8A92-D14B-5EFA-A96C-55CF37D5711F

Figs 55 , 56

Boysidia fengxianensis Chen, Liu & Wu, 1995: 275–276 (Chinese description), 276–277 (English description), fig. 2.
Bensonella fengxianensis — Schileyko 1998: 140; Páll-Gergely and White 2023: 2016.
Boysidia (Boysidia) fengxianensis — Chen and Zhang 2002: 692.
Boysidia (Bensonella) fengxianensis — Zhang et al. 2014: 572.

##### Material examined.

**China** • 7 shells; Shaanxi province, Chang’an district, subtropical broadleaf forest, 1.2 km SE Xiaoqian Goukou village; 33°57.709'N, 108°45.868'E; 780 m a.s.l.; 08 July 2010; D.M. Palatov leg.; coll. JG • 4 shells; Hubei, Enshi Tujiazu Miaozu Zizhizhou, Enshi Shi, Taiyanghe Xian, Suobuya Shilin Jingqu, split rock; 30°34.663'N, 108°34.401'E; 975 m a.s.l.; 06 Nov. 2010; A. Hunyadi leg.; coll. HA • 1 shell; Hubei, Enshi Tujiazu Miaozu Zizhizhou, Badong Xian, E Badong, Bashan Senlin Gongyuan (near Xinglingzhen); 31°01.472'N, 110°25.284'E; 225 m; 03 Nov. 2011; A. Hunyadi leg.; coll. HA • 1 shell; Longfengyan, Hengjiangqiaoxiang, Jingzhoumiaozudongzuzizhixian, Hunansheng; 26°27.382'N, 109°34.650'E; 357 m a.s.l.; K. Ohara, K. Okubo, J. U. Otani leg.; coll. PGB • 5 shells; Sanjiaoyan. Zhilouzangzuxiang, Wenxian, Gansusheng Wenxian, Gansasheng; 32°53.180′N,, 104°24.672'E; 1646 m a.s.l.; Y. Nakahara, K. Okubo, J. U. Otani leg.; coll. PGB • 1 shell; along walking path below Cuying Lake (翠映湖), Qingchenghoushan (青城后山), Qingchengshanzhen (青城山镇), Dujiangyanshì (都江堰市), Chengdushì (成都市), Sìchuansheng (四川省); 30°56.2302'N, 103°28.7484'E; 1310 m a.s.l.; A. Hunyadi, T. Ishibe, K. Okubo, J. U. Otani, M. Szekeres leg.; coll. HA.

##### Type locality.

“Fengxian County (33°08'N, 106°05'E), Shaanxi Province” (China).

##### Distribution.

This species is known only from the type locality.

##### Remarks.

We could not locate the type specimens of this species. The original illustration of the holotype is inadequate. Based on the presence of the palatal tubercle, arrangement of apertural barriers, and shell shape, this species must belong to *Bensonella* (as already suggested by Páll-Gergely and White 2023). The specimens examined by us, collected in Shaanxi province (China) where the type locality of *B.fengxianensis* is located, may belong to this species, but this cannot be confirmed due to the absence of the type specimen. The same can be said for other samples in the nearby provinces (see Fig. 56).

**Figure 55.**
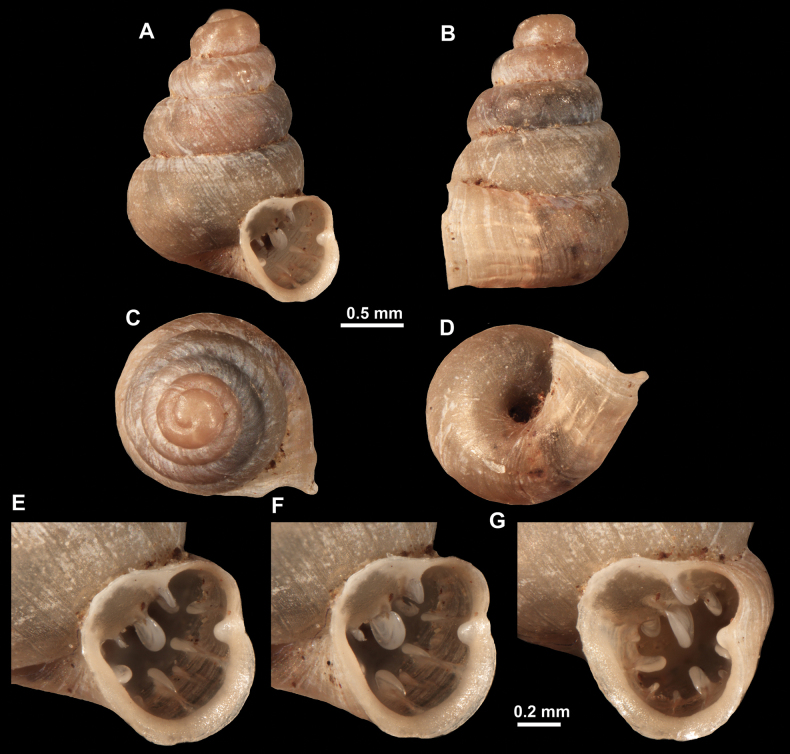
Bensonellacf.fengxianensis from Shaanxi Province (coll. JG) **A–D** shell **E–G** enlarged views of the aperture.

**Figure 56.**
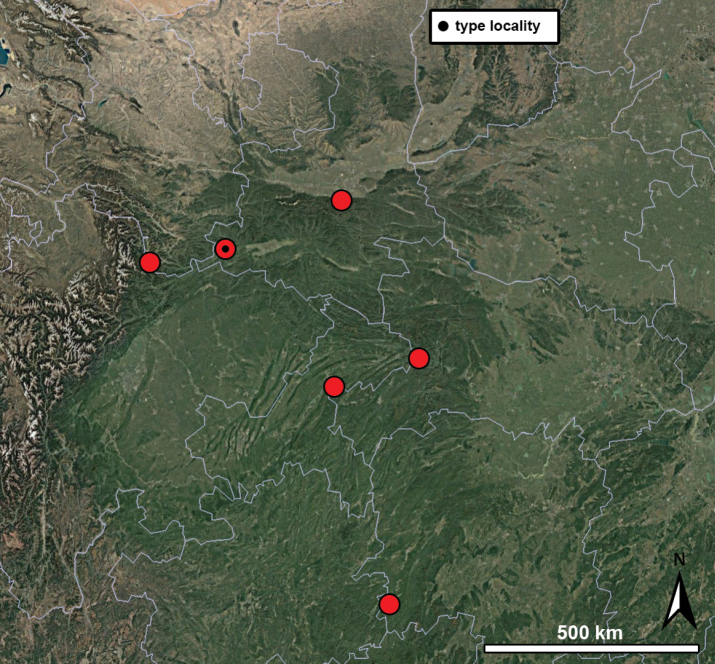
Distribution map of Bensonellacf.fengxianensis.

#### ﻿ Bensonella geminounca

Taxon classificationAnimaliaStylommatophoraHypselostomatidae

Jirapatrasilp & Tongkerd, 2024

A2C203FE-E1E7-52A1-A91C-EDA97DD03617

Figs 39G , 57 , 100

Bensonella geminounca Jirapatrasilp & Tongkerd in Jirapatrasilp et al. 2024: 90–91, fig. 2.

##### Type material examined.

**Thailand** • holotype; 27. May 1997, S. Panha, J. B. Burch, P. Dumrongrojwattana leg.; CUMZ 14366.

##### Type locality.

“Doi Chiang Dao, Chiang Dao Wildlife Sanctuary, Chiang Dao District, Chiang Mai Province, Thailand (19°22′046.3″N, 098°51′08.1″E, 2,015 m a.m.s.l.)”.

##### Differential diagnosis.

This species has a unique appearance of the infraparietal lamella which readily distinguishes it from all other congeners: it is consisting of two hooked parts pointing toward each other, outer (pointing inwards) and inner (pointing outwards).

**Figure 57.**
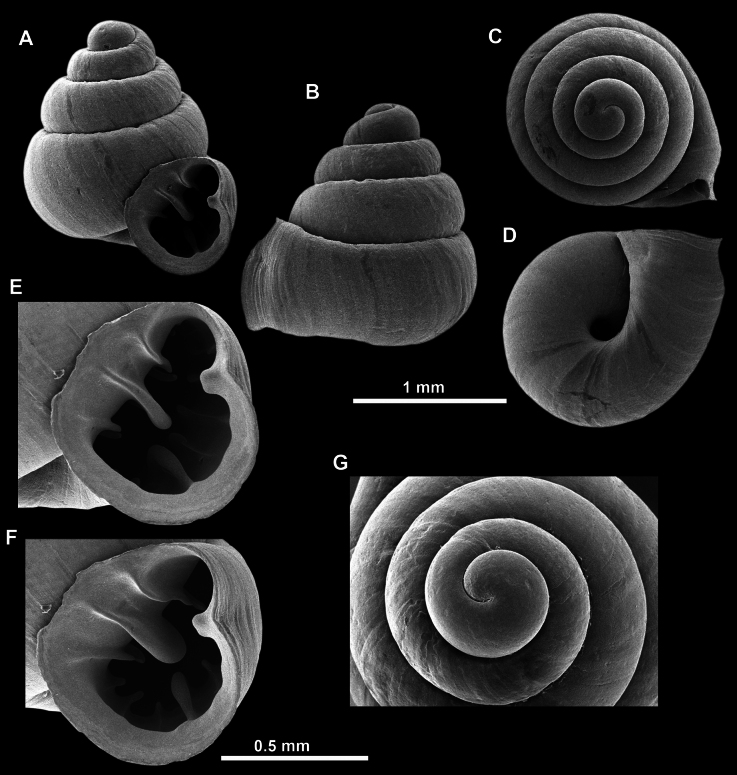
*Bensonellageminounca*, holotype (CUMZ 14366) (from Jirapatrasilp et al. 2024) **A–D** shell **E, F** enlarged apertural view **G** enlarged view of the protoconch.

##### Distribution.

This species is known from different altitudes at the type locality (altitude range: 1650–2015 m).

#### ﻿ Bensonella hooki

Taxon classificationAnimaliaStylommatophoraHypselostomatidae

Páll-Gergely, 2023

7F5AEA59-A815-5019-84DE-A68197701E66

Figs 39H , 58

Bensonella hooki Páll-Gergely in Páll-Gergely and White 2023: 2023–2025, figs 6, 9c, d.

##### Type material examined.

**India** • holotype; Godwin-Austen coll.; NHMUK 20191114.

##### Type locality.

“…Cherrapunji…” (India).

##### Differential diagnosis.

See under *B.lakainguta*, *B.montawa* sp. nov., *B.multihami*, *B.plicidens* and *B.spinosa* sp. nov.

##### Distribution.

SE and SW Himalaya region.

##### Remarks.

After long-lasting taxonomic confusion, it has been shown that this species (hooked apertural barriers) is distinct from *B.plicidens* (blunt barriers) even though both “forms” were found mixed together in the same specimen lot (for details see Páll-Gergely and White 2023).

**Figure 58.**
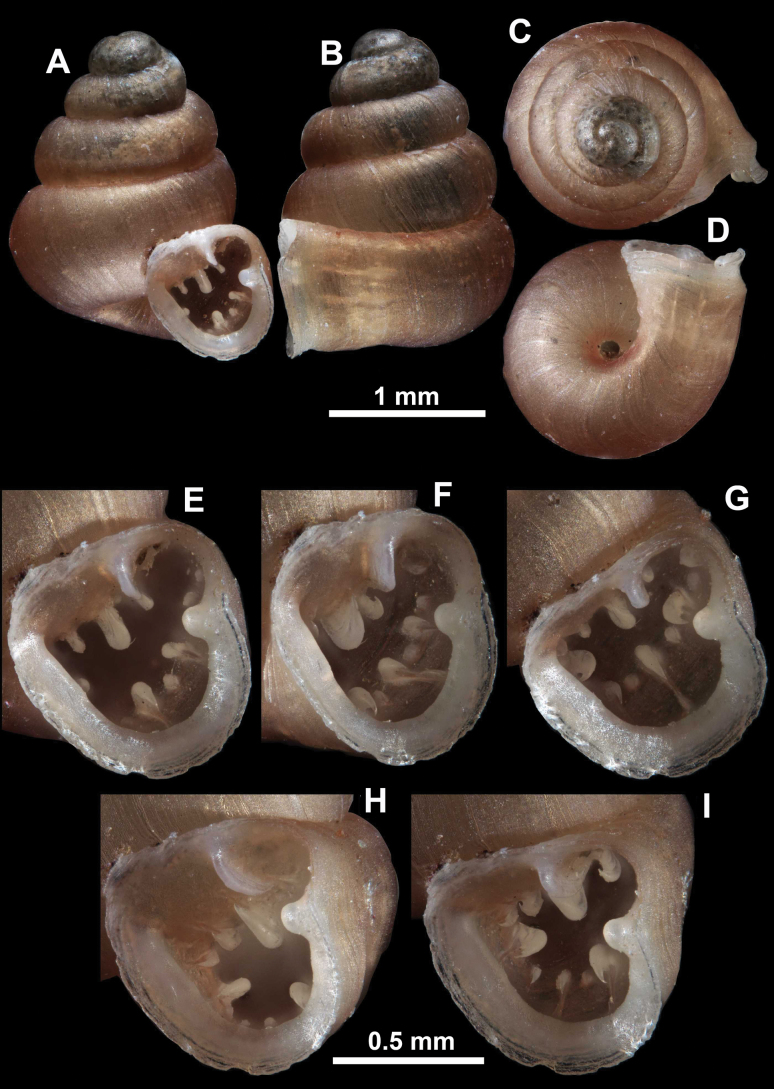
*Bensonellahooki*, holotype (NHMUK 20191114) **A–D** shell **E–I** enlarged apertural views (from Páll-Gergely and White 2023).

#### ﻿ Bensonella karoensis

Taxon classificationAnimaliaStylommatophoraHypselostomatidae

Maassen, 1999

92F5A80D-85FD-53E0-866D-9AF74756E6E0

Figs 39I , 59 , 60

Bensonella karoensis Maassen, 1999: 123–126, figs 8, 9.

##### Type material examined.

**Indonesia** • holotype; Jul 1996; W. Maassen leg.; RMNH.Moll.84438.

##### Type locality.

“N. Sumatra, Karo Highlands, Kuta Buluh, 40 km N of Brastagi, near the entrance of the cave Liangdehar in leaf litter at the foot of limestone rocks”.

##### Differential diagnosis.

See under *B.plicidens*.

##### Distribution.

This species is known only from the type locality.

##### Remarks.

This species is known only by a holotype (Maassen 1999).

**Figure 59.**
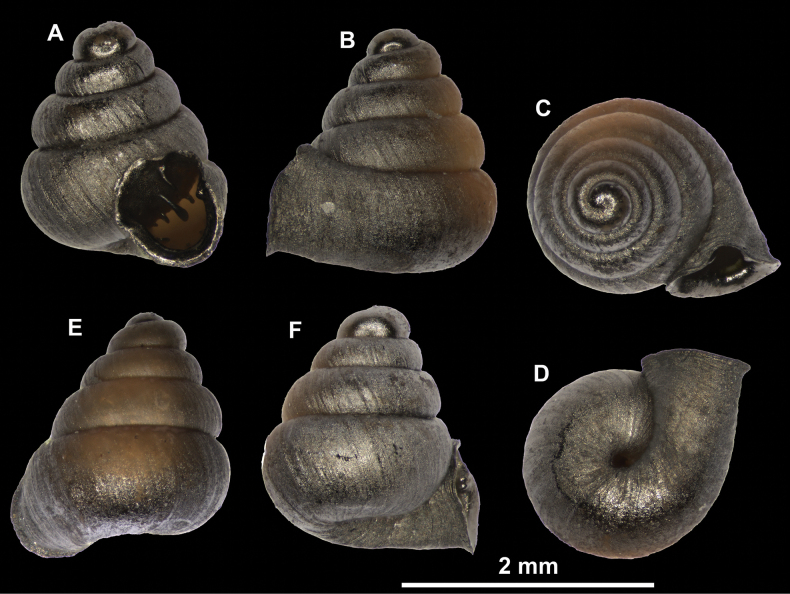
*Bensonellakaroensis*, holotype (RMNH.Moll.84438) **A–F** shell.

**Figure 60.**
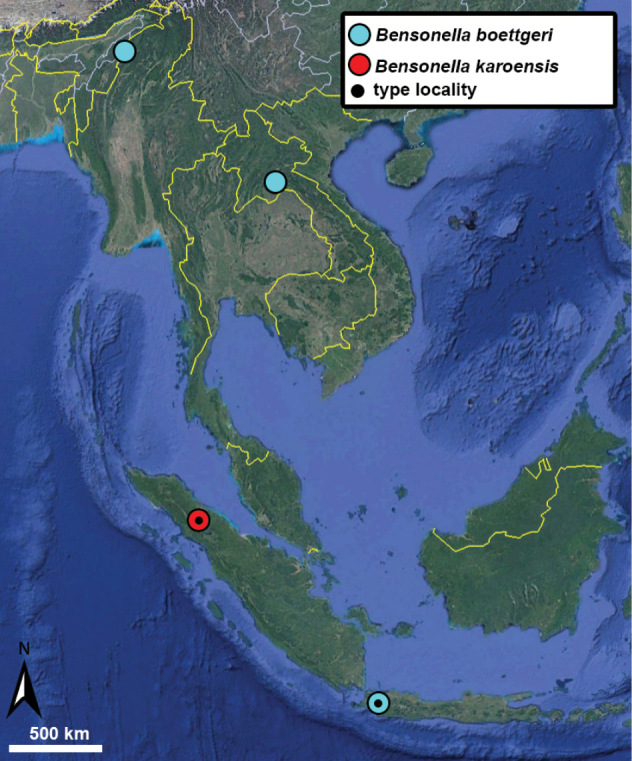
Distribution map of *Bensonellaboettgeri* and *B.karoensis*.

#### ﻿ Bensonella lakainguta

Taxon classificationAnimaliaStylommatophoraHypselostomatidae

C.-C. Hwang, 2014

D00007D2-6014-5D33-AC6E-811CD2C945CC

Figs 39J , 61

Bensonella plicidens lakainguta Hwang, 2014: 18–19, figs 2–4.
Boysidia (Bensonella) qingliangfengensis Fang, Wang & Chen, 2015: 692, fig. 1.
Bensonella lakainguta — Páll-Gergely and White 2023: 2025–2026, figs 7, 8, 9e–h.
Bensonella qingliangfengensis — Páll-Gergely and White 2023: 2020, fig. 7d–f.

##### Type material examined.

**Taiwan** • holotype; 27 Aug. 1998, C-C. Hwang leg.; NMNS-7244-00.

##### Additional material examined.

**Japan** • 4 shells; limestone outcrop along forest rd., upstream Ugui-gawa, Aiga, Tsuchiyama-cho, Koka, Shiga; 34°57.9108'N, 136°22.185'E; 461 m a.s.l.; Kawase, J. U. Otani leg.; coll. PGB.

##### Type locality.

“TAIWAN: Mt. Zhibenzhushan, Wutai, Pingdung, 22°43'32.6"N, 120°52'50.2"E, alt. 2100 m”.

##### Differential diagnosis.

This species differs from *B.hooki* by its more triangular shell, more pointed apex, and parietal callus adnate to the penultimate whorl (not so in *B.hooki*, see Páll-Gergely and White 2023). For differences from *B.montawa* sp. nov., *B.multihami*, *B.plicidens*, and *B.spinosa* sp. nov. see under those species.

**Figure 61.**
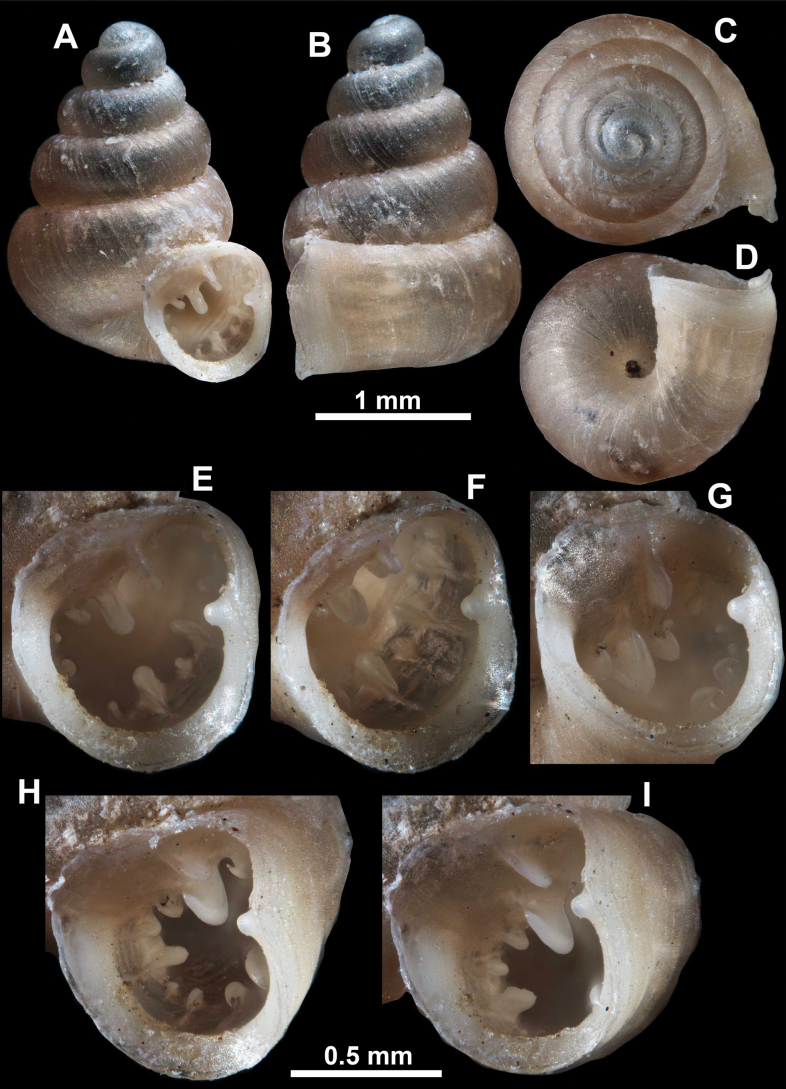
*Bensonellalakainguta*, holotype (NMNS-7244-00) **A–D** shell **E–I** enlarged apertural views (from Páll-Gergely and White 2023).

##### Distribution.

This species is well known from Japan as well as Taiwan and China (where it is known from a synonym, *B.qingliangfengensis*) (Fang et al. 2015; Páll-Gergely and White 2023).

##### Remarks.

This species was originally described as a subspecies of *B.plicidens*. Páll-Gergely and White (2023) have shown that it is distinct as it has hooked and not blunt apertural barriers, so. *B.plicidens* does not inhabit Japan and Taiwan where only *B.lakainguta* occurs.

#### ﻿ Bensonella lophiodera

Taxon classificationAnimaliaStylommatophoraHypselostomatidae

Tongkerd & Panha, 2024

975238FF-B3ED-573D-896F-DCD05D796E5F

**Figure 62.**
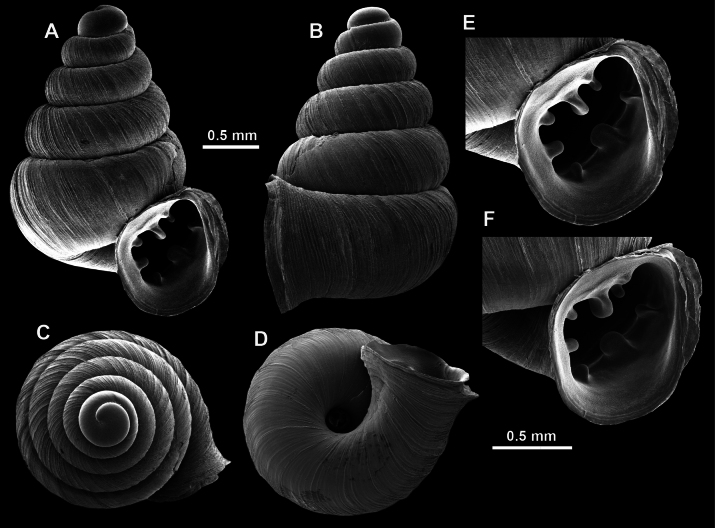
*Bensonellalophiodera*, holotype (CUMZ 14378) (from Tongkerd et al. 2024) **A–D** shell **E, F** enlarged apertural views.

**Figure 63.**
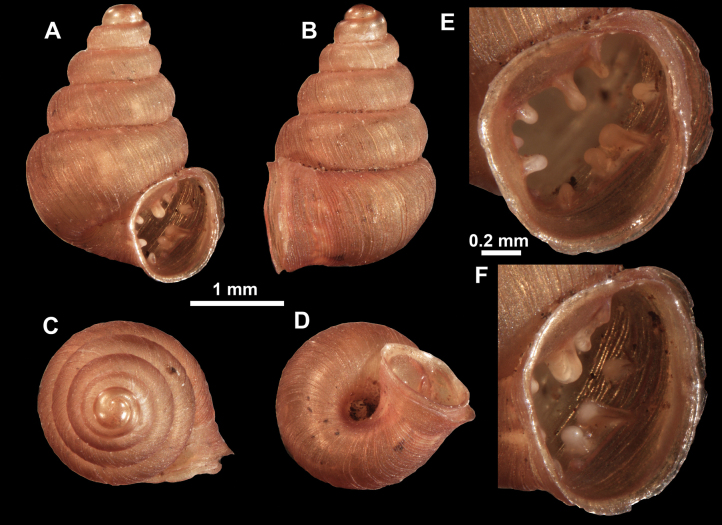
*Bensonellalophiodera* from Shan, Kalaw (Myanmar) (coll. HA) **A–D** shell **E, F** enlarged apertural views.

Figs 39K , 62 , 63 , 97

Bensonella lophiodera Tongkerd & Panha in Tongkerd et al. 2024: 176–178, figs 7C, 9, 13I.

##### Type material examined.

**Myanmar** • holotype; collector unknown; CUMZ 14378.

##### Additional material examined.

**Myanmar** • 158 shells; Shan State, NNE of Kalaw, Osei Mountain Pagoda NW 150 m; 20°39.320'N, 96°34.927'E; 1565 m a.s.l.; 03 Oct. 2018; A. Hunyadi, K. Okubo & J.U. Otani leg.; coll. HA • 181 shells; Shan State, Kalaw ESE 13.5 km, Myinmati Taung; 20°35.4264'N, 96°36.7938'E; 1350 m a.s.l.; 03 Oct. 2018; A. Hunyadi, K. Okubo & J.U. Otani leg; coll. HA.

##### Type locality.

“Myinmati Cave, Kalaw Township, Taunggyi District, Shan State, Myanmar (20°35'24.6"N, 96°36'42.1"E; 1312 m a.s.l.)”.

##### Differential diagnosis.

The shell of this species is relatively large when compared to other geographically approximate congeners and the transversal palatal plica is rather weak and small. See under *B.taiyaiorum*.

##### Distribution.

This species is known from three localities in Kalaw.

##### Remarks.

This species lacks the palatal tubercle but is still clearly a member of *Bensonella* because of the triangular-conical shell shape and three barriers on the parietal side. In our specimens collected from Osei Mountain Pagoda (see above under “Material examined”), there is a transversal plica which is usually only partly merged, i.e., interpalatal and lower palatal plicae are clearly discernible (Fig. 63). Even though in the original description, this barrier is listed as a lower palatal, we believe that it is actually a transversal plica but just much weaker than in other congeners. Furthermore, Tongkerd et al. (2024: fig. 9C) shows a lower palatal plica that is wider than normal and positioned transversally indicating the fusion of the interpalatal and lower palatal plicae. Otherwise, our examined specimens fit perfectly in the description of *B.lophiodera* by all shell characters (size, colour, shape, surface sculpture, and apertural dentition).

#### ﻿ Bensonella microdentata

Taxon classificationAnimaliaStylommatophoraHypselostomatidae

Gojšina & Páll-Gergely sp. nov.

DAA9BE7A-A095-50DD-AE40-43AC30C7B0BF

https://zoobank.org/1E2B0056-0A73-4006-975F-3B458258437F

**Figure 64.**
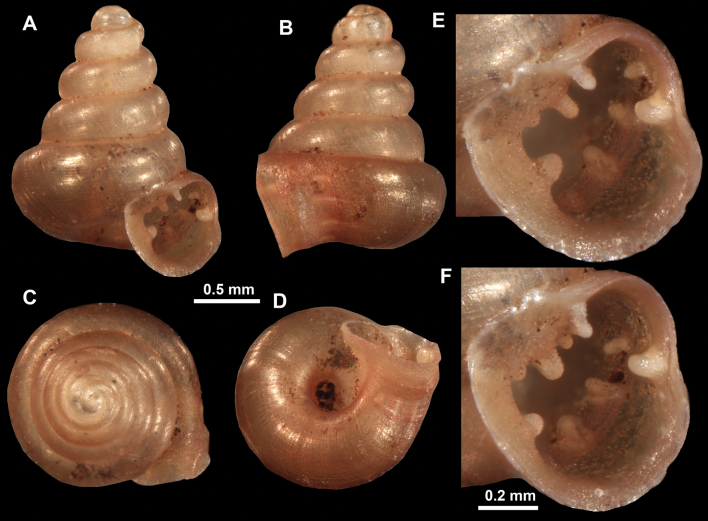
*Bensonellamicrodentata* Gojšina & Páll-Gergely, sp. nov., holotype (UF 346912) **A–D** shell **E, F** enlarged apertural views.

**Figure 65.**
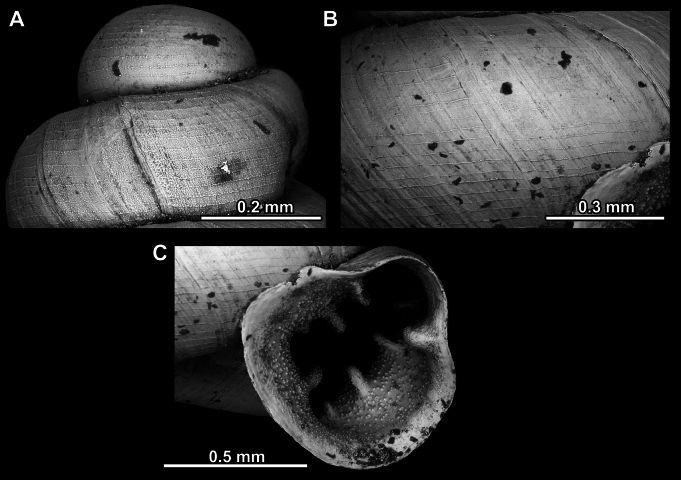
SEM imaging of *Bensonellamicrodentata* Gojšina & Páll-Gergely, sp. nov., holotype (UF 346912) **A** protoconch surface **B** surface of the last whorl **C** apertural view.

Figs 39L , 64 , 65 , 95

##### Type material.

***Holotype*. Thailand** • 1 shell (SH: 2 mm; SW: 1.5 mm); Lampang Province limestone dome at pass 11 km SSW of Ban Pang La; 18°27.9667'N, 99°48.3833'E; 610 m a.s.l.; 14. May 1988; F.G. Thompson leg.; UF 346912. ***Paratypes*. Thailand** • 1 shell; same data as for holotype; CUMZ14437 • 6 shells; same data as for holotype; UF 591333.

##### Additional material examined.

**Thailand** • 3 shells (juveniles, not paratypes); same data as the holotype; UF 583726.

##### Type locality.

Thailand, Lampang Province limestone dome at pass 11 km SSW of Ban Pang La; 18°27.9667'N, 99°48.3833'E; 610 m a.s.l.

##### Diagnosis.

*Bensonella* species with concave-conical, glossy shell that is spirally striated. Apertural barriers (usually eight or nine) and the surface around them prominently spiniferous.

##### Description.

Shell slightly concave-conical, glossy, light brownish, consisting of 5–5.5 convex and rounded whorls separated by a deep suture. Protoconch spirally striated, its boundary with the teleoconch not clear. There are ~ 12 widely spaced spiral striae on the protoconch and they are stronger at the base of the whorl. Teleoconch with relatively coarsely spaced, raised spiral striae (space between two striae equals the width of two or three striae) and with fine radial growth lines. On initial teleoconch whorls, radial lines are more densely arranged (similar to the spacing between the spiral striae) and are crossing the spiral striae forming a reticulated sculpture. Towards the last whorl, radial lines become coarser and the reticulated sculpture becomes weaker. Last whorl rounded, adnate to the penultimate, slightly descending near the aperture (~ 20 ° compared to the shell axis). Peristome whitish, expanded but not reflected. Sinulus parabolic due to the position of the angular lamella and the palatal tubercle. Aperture equipped with numerous barriers which have the typical *Bensonella* arrangement. These are: angular, parietal, upper palatal, interpalatal, lower palatal, palatal tubercle, basal, columellar, and infraparietal. Parietal lamella strong and moderately high, angular separated into inner and outer parts of almost equal height and width and a strong sinuation between them. Upper and interpalatal plicae positioned close together and almost equally as strong. Lower palatal plica situated some distance from other palatals and stronger than both of them. Palatal tubercle strong and sitting on the edge of the palatal lip. Basal plica weak, low, and short. Columellar lamella nearly as strong as the lower palatal. Infraparietal lamella only very slightly stronger than the basal plica. Surface of all apertural barriers is roughly spiniferous. Umbilicus narrow but relatively deep, measuring ~ 1/6 of the shell width.

##### Differential diagnosis.

This species is most similar to *B.nitens* (see under that species for differences). See also under *B.paviei* and *B.tamphathai*.

##### Measurements

**(in mm, *n* = 4).**SH = 1.73–2; SW = 1.47–1.61; AH = 0.73–0.83; AW = 0.63–0.75.

##### Etymology.

This species is named for its small apertural barriers.

##### Distribution.

This species is known only from the type locality.

#### ﻿ Bensonella mirabilis

Taxon classificationAnimaliaStylommatophoraHypselostomatidae

Gojšina, Grego & Páll-Gergely sp. nov.

541B399B-3B52-5192-9281-39DDAF9FB6A5

https://zoobank.org/DD16A504-D9AD-47AC-98D8-CDC0C329D359

**Figure 66.**
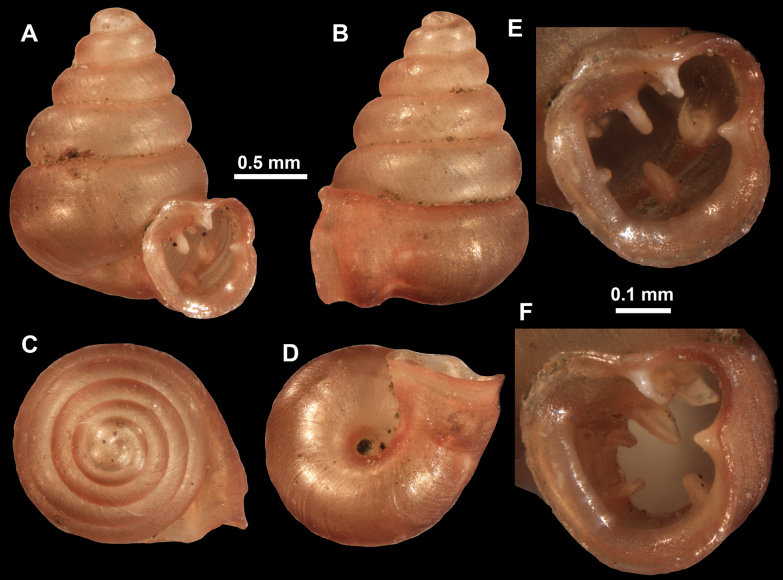
*Bensonellamirabilis* Gojšina, Grego & Páll-Gergely, sp. nov., holotype (CUMZ 14438) **A–D** shell **E, F** enlarged apertural views.

**Figure 67.**
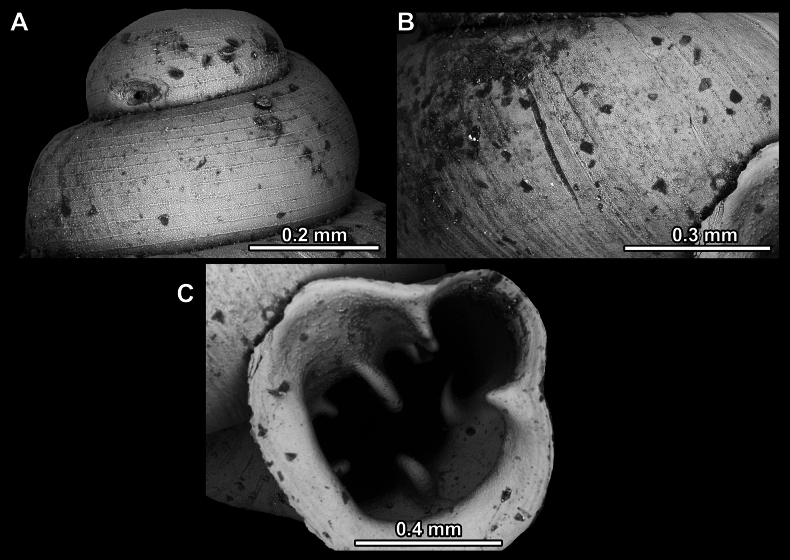
SEM imaging of *Bensonellamirabilis* Gojšina, Grego & Páll-Gergely, sp. nov., holotype (CUMZ 14438) **A** protoconch surface **B** surface of the last whorl **C** apertural view.

Figs 39M , 66 , 67 , 95

##### Type material.

***Holotype*. Myanmar** • 1 shell (SH: 2.05 mm, SW: 1.65 mm); Kayah State, Hpruso district, Maw Thi Do, road towards Han Li village, under bridge over Phruno river; 19°22.966'N, 97°2.153'E; 12 Feb. 2019; J. Grego leg.; CUMZ14438. ***Paratypes*. Myanmar** • 9 shells; same data as for holotype; coll. JG • 13 shells; Kayah state, Hpruso district, Maw Thi Do Village, entrance of Phruno river cave; 19°22.744'N, 97°2.570'E; 12 Feb. 2019; J. Grego leg.; coll. JG • 1 shell; same data as previous; coll. HA • 1 shell; same data as previous; coll. VG • 2 shells; Kayah State, Hpruso distr., Hoyar village, Kwar Yin cave; 19°18.096'N, 96°56.396'E; 13 Feb. 2019; J. Grego leg.; coll. JG • 1 shell; Kayah state, Hpruso district, Maw Thi Do, road towards Han Li village, rocks above bridge over Phruno river; 19°23.011'N, 97°2.108'E; 12 Feb. 2019; J. Grego leg.; coll. JG.

##### Additional material examined.

**Myanmar** • 5 shells (juveniles, not paratypes); same data as for holotype; coll. JG.

##### Type locality.

Myanmar, Kayah State, Hpruso district, Maw Thi Do, road towards Han Li village, under bridge over Phruno river; 19°22.966'N, 97°2.153'E.

##### Diagnosis.

*Bensonella* species with seven apertural barriers and a prominent hooked upper palatal plica which is pointing laterally (toward the palatal wall).

##### Description.

Shell triangular, slightly conical-ovoid, brownish, consisting of 5–5.5 whorls separated by a deep suture. All whorls rounded, convex, regularly increasing. Protoconch coarsely spirally striated, ~ 1.5 whorls. Spiral striae much more prominent terminally on the protoconch. Teleoconch with a fine pasty surface sculpture, without spiral striae but with fine radial growth lines. Last whorl rounded, adnate to the penultimate and straight near the aperture. Peristome brownish as the rest of the shell, expanded but not reflected. It is leaning on the penultimate whorl in form of a strong but not much expanded parietal callus. Palatal side of the peristome sinuated in its middle part, next to the palatal tubercle. Behind the expanding peristome, a weak cervical crest is present. Sinulus small and narrow, distinctly separated from the rest of the aperture. Aperture subrectangular, equipped with a smaller number of barriers (angular, parietal, upper palatal, lower palatal, columellar, infraparietal and palatal tubercle) than usual in *Bensonella*. Parietal lamella long but not reaching the expanding peristome. Angular lamella consisting of two separated parts: inner, which is slightly curved and positioned deeper in the aperture and outer part which is reaching the peristome edge. There is a strong sinuation between the inner and outer parts of the angular lamella. Palatal wall equipped with upper and lower palatal plica. Upper palatal plica of very peculiar appearance: it is very high with a hook positioned laterally (not pointing outside but directly to the palatal wall). Lower palatal plica nearly as strong as the parietal lamella. In front of the upper palatal plica, on the peristome edge, a strong palatal tubercle is observed. Columellar lamella rather weak. Above the columellar lamella, one additional infraparietal is present and it is nearly equally strong as the columellar. Surface of all apertural barriers is finely granulated. Umbilicus oblique (slightly elongated) and narrow, measuring ~ 1/6–1/7 of the shell width.

##### Differential diagnosis.

This species is easily distinguished from all other congeners by the presence of a laterally hooked upper palatal plica.

##### Measurements

**(in mm, *n* = 5).**SH = 2.03–2.16; SW = 1.52–1.70; AH = 0.80–0.88; AW = 0.77–0.82

##### Etymology.

Named for the remarkable upper palatal plica (laterally hooked).

##### Distribution.

Known from four sampling sites in Kayah state, Hpruso district.

#### ﻿ Bensonella montawa

Taxon classificationAnimaliaStylommatophoraHypselostomatidae

Gojšina, Hunyadi & Páll-Gergely sp. nov.

B698D88A-4FC8-59D7-B22E-92907866E666

https://zoobank.org/4B7BFD25-47D3-424E-B2B1-07A6157F9FAF

Figs 39N , 68 , 95

##### Type material.

***Holotype*. Myanmar** • 1 shell (SH: 1.8 mm, SW: 1.5 mm); Shan State, west-southwest from Taunggyi, Montawa cave; 20°45.282'N, 97°1.057'E; 1260 m a.s.l.; 05 Oct. 2018; A. Hunyadi, K. Okubo & J.U. Otani leg.; CUMZ 14471. ***Paratypes*. Myanmar** • 30 shells; same data as for holotype; coll. HA.

##### Type locality.

Myanmar, Shan State, west-southwest from Taunggyi, Montawa cave; 20°45.282'N, 97°1.057'E; 1260 m a.s.l.

##### Diagnosis.

A *Bensonella* species with conical, pale yellowish shell. Aperture equipped with numerous barriers, all of which are in form of sharp hooks pointing outside. Parietal lamella with a blunt but long projection in front of the hook, almost reaching the expanding peristome. Umbilicus narrow, dot-like.

**Figure 68.**
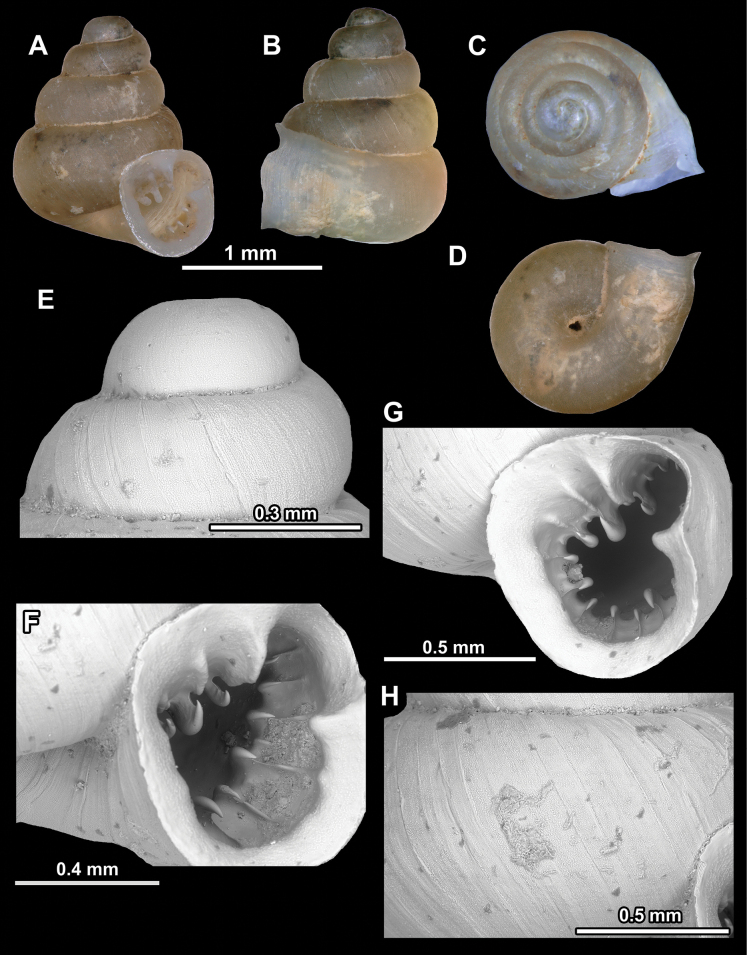
*Bensonellamontawa* Gojšina, Hunyadi & Páll-Gergely, sp. nov., holotype (CUMZ 14471) **A–D** shell **E** enlarged protoconch view **F, G** enlarged views of the aperture **H** enlarged view of the last whorl surface.

##### Description.

Shell pale yellowish, triangular, conical ovoid, whorls 4–4.5, convex, rounded, rather regularly increasing. Protoconch consisting of ~ 1.25 whorls, showing spiralling pattern but with no clear spiral striae. Teleoconch with pasty surface, not spirally striated but with coarsely spaced, irregular radial growth lines. Last whorl adnate to the penultimate and slightly ascending near the aperture (~ 15–20 ° compared to the shell axis) making the aperture profile opisthocline to the shell axis. Peristome strongly expanded, not reflected. Aperture equipped with numerous barriers, all of which are in form of sharp hooks pointing outside the aperture. All barriers have low, slender projections situated in front of the hooks. A blunt, knob-like palatal tubercle sits anterior to the upper palatal plica on the peristome edge. Parietal lamella in form of a strong hook. A blunt, outer part of the parietal lamella is in form of a low, narrow, and blunt projection (situated in front of the hooked part) almost reaching the peristome edge. Angular lamella strong, consisting of inner and outer part separated by a strong sinuation. Inner part smaller, hooked, pointing towards the outer part which is larger but blunt. There are four weak barriers inside the sinulus. Upper palatal plica ~ 2 × weaker than the parietal and situated slightly above the palatal tubercle. First interpalatal plica as strong as the upper palatal, situated slightly below the palatal tubercle. Second interpalatal plica slightly weaker than the first. Lower palatal plica as strong as the first interpalatal and upper palatal. Infrapalatal plica small, as strong as the basal and subcolumellar. These three plicae are roughly equidistant. Above the subcolumellar, there is a clearly stronger columellar (similar in strength to the lower palatal plica) and one slightly stronger supracolumellar. Infraparietal lamella ~ 1/3 weaker than the parietal, with a similar (but shorter) projection in front of the hooked part. There are also some barrier-like swellings, although they never develop into a proper barrier. They can be e.g., between the upper palatal and first interpalatal plica, between the columellar and supracolumellar lamellae, between the supracolumellar and infraparietal lamellae, between the infraparietal and parietal lamellae. Surface of all apertural barriers is smooth. Sinulus parabolic, well separated from the rest of the aperture. Umbilicus very narrow, dot-like.

##### Differential diagnosis.

This species is very similar to *B.hooki* and *B.lakainguta*. *Bensonellahooki* is however more ovoid, reddish-brown, and does not have a slender projection in front of the parietal lamella. The latter also distinguishes the new species from *B.lakainguta*, which is also more triangular (i.e., pointed). See also under *B.multihami*.

##### Measurements

**(in mm, *n* = 3).**SH = 1.8; SW = 1.4–1.6; AH = 0.69–0.77; AW = 0.74–0.79.

##### Etymology.

This species is named after its type locality, the Montawa Cave. The specific epithet is to be used as a noun in apposition.

##### Distribution.

This species is only known from the type locality.

#### ﻿ Bensonella multidentata

Taxon classificationAnimaliaStylommatophoraHypselostomatidae

Gojšina, A. Reischütz & Páll-Gergely sp. nov.

16D64A5B-58B2-501D-BC9E-61C90319576B

https://zoobank.org/7CAB96FE-3096-42D3-9087-7E9D5EEAFFE4

**Figure 69.**
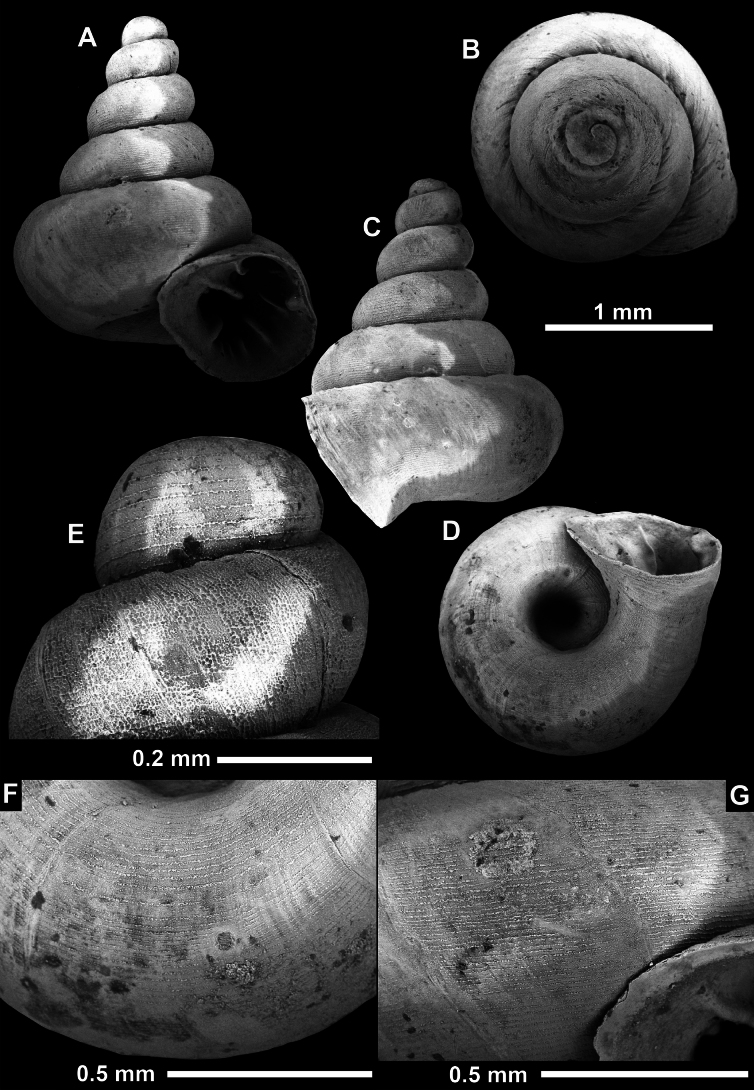
*Bensonellamultidentata* Gojšina, A. Reischütz & Páll-Gergely, sp. nov., holotype (NHMW-MO-113729) **A–D** shell **E** enlarged protoconch view **F, G** enlarged views of the surface of the last whorl.

**Figure 70.**
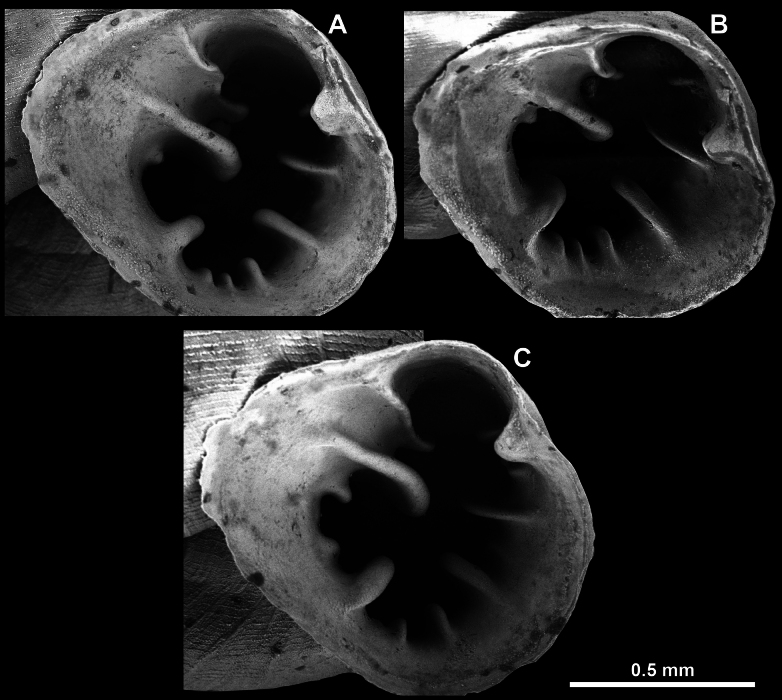
*Bensonellamultidentata* Gojšina, A. Reischütz & Páll-Gergely, sp. nov., enlarged apertural views **A, B** holotype (NHMW-MO-113729) **C** paratype (coll. REI).

**Figure 71.**
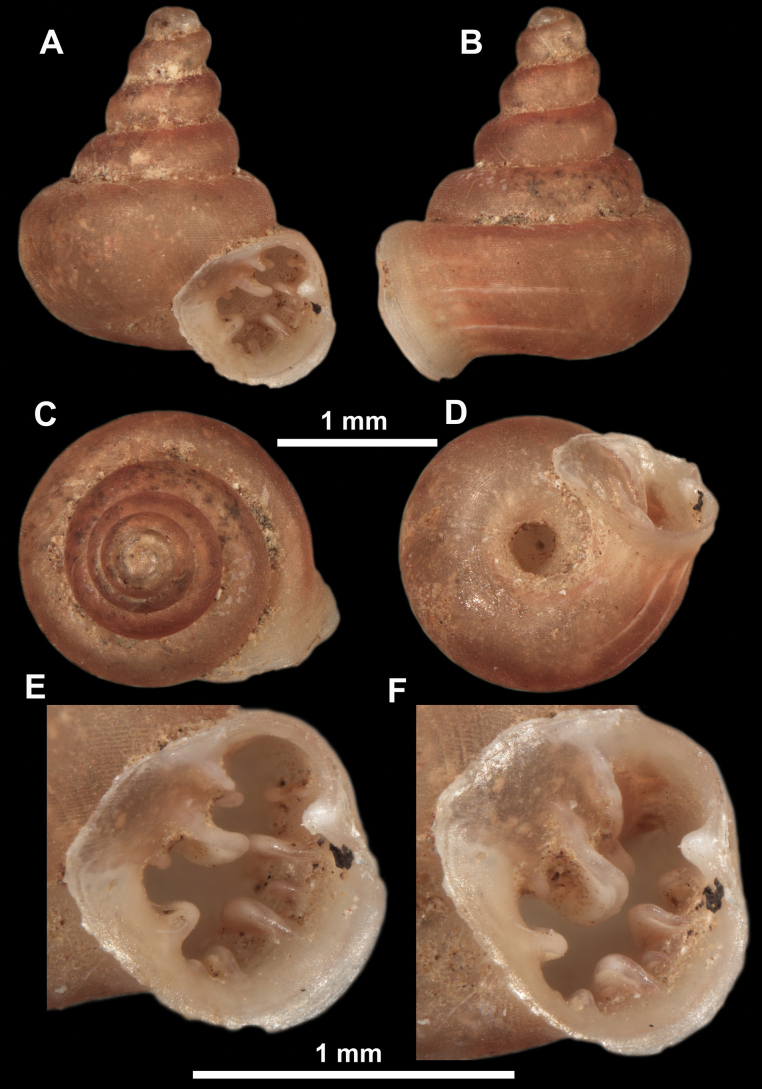
*Bensonellamultidentata* Gojšina, A. Reischütz & Páll-Gergely, sp. nov., paratype (coll. HA) **A–D** shell **E, F** enlarged apertural views.

**Figure 72.**
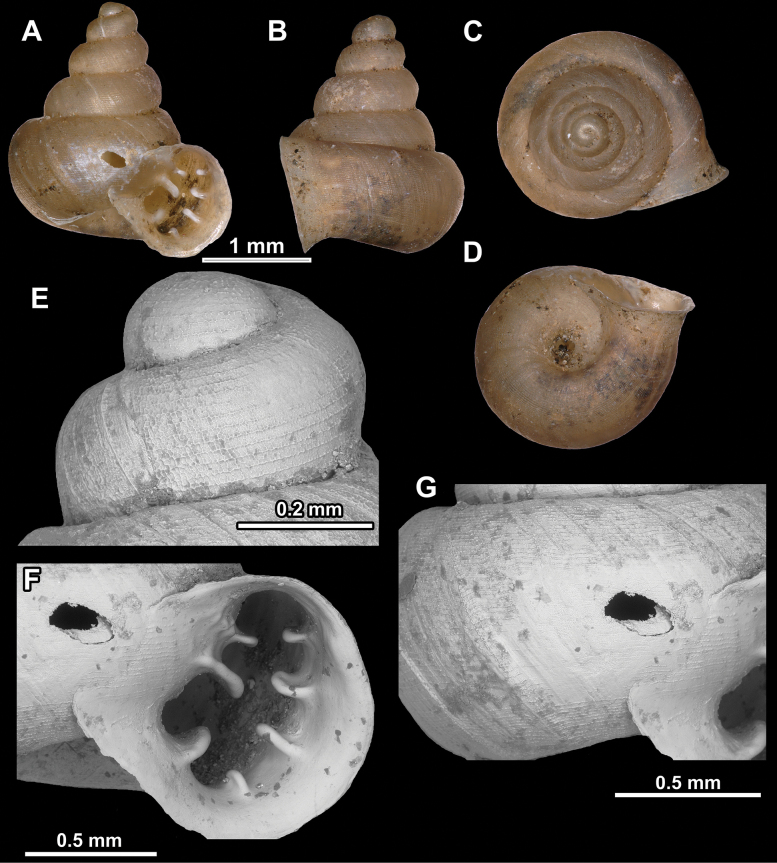
Bensonellacf.multidentata Gojšina, A. Reischütz & Páll-Gergely, sp. nov., specimen found near Ban Mae Suai (UF 347105) **A–D** shell **E** enlarged view of the protoconch **F** enlarged view of the aperture **G** view of the last whorl surface sculpture.

Figs 39O , 69 , 70 , 71 , 72 , 100

##### Type material.

***Holotype*. Thailand** • 1 shell (SH: 2.40 mm, SW: 1.95 mm); Chiang Rai Province, Wat Phra That Tham Doi Khong Khao Meditation Centre, cave clay at the entrance of the cave, ca 6 km W of Chiang Rai; 19°54.771'N, 99°46.607'E; ca 420 m a.s. l.; Sept. 2017; A. Reischütz leg.; NHMW-MO-113729. ***Paratypes*. Thailand** • 1 shell; same data as for holotype; CUMZ 14439 • 29 shells; same data as for holotype; coll. REI • 1 shell; same data as for holotype; coll. VG • 41 shells; Chiang Rai Province, 5 km west from centre of Chiang Rai, vicinity of Wat Phra That Tham Doi Kong Khao; 19°54.820'N, 99°46.642'E; 420 m a.s.l.; 11 Feb. 2015; A. Hunyadi leg.; coll. HA.

##### Additional material examined.

**Thailand** • 3 shells (damaged/ juveniles, not paratypes); same data as for holotype; coll. REI • 2 shells; Chiang Rai Province, south-southwest from Mae Sai, vicinity of Wat Tham Pla; 20°19.723'N, 99°51.817'E; 400 m a.s.l.; 12 Feb. 2015; A. Hunyadi leg.; coll. HA • 22 shells; Chiang Rai Province, limestone Mtn. 6 km by road W of Ban Mae Suai; 19°38′55″N, 99°26′41″E; 650 m a.s.l.; 07. May 1988; F. G. Thompson leg.; locality code FGT-4415; UF 347105.

##### Type locality.

Thailand, Chiang Rai Province, Wat Phra That Tham Doi Khong Khao Meditation Centre, cave clay at the entrance of the cave, ca 6 km NW of Chiang Rai, 19°54.771'N, 99°46.607'E, ca 420 m a.s.l.

##### Diagnosis.

A *Bensonella* species with high triangular, concave-conical, densely spirally striated shell. Last whorl wide, rounded. Multiple (at least 10) well-developed apertural barriers.

##### Description.

Shell light brown, triangular, concave-conical, whorls 5–5.25, convex, rounded, rather regularly increasing. Protoconch consisting of ~ 1.25 whorls, its sculpture consists of ~ 10 equidistant spiral striae. Teleoconch finely, very densely spirally striated, and ornamented with a few, inconspicuous radial growth lines. Last whorl adnate to the penultimate and slightly to moderately descending near the aperture (~ range: 15–32 ° compared to the shell axis) making the aperture profile prosocline to the shell axis. Aperture subcircular, parietal part strongly extended towards penultimate whorl. Peristome strongly expanded, not reflected. Aperture with numerous and strong barriers. A blunt, knob-like palatal tubercle sits anterior to the upper palatal plica on the peristome edge. Sometimes, this tubercle is very weak or completely absent. All other barriers sit deeper, not reaching the peristome edge. Parietal lamella is the highest among all apertural barriers, long, almost straight, only slightly curved downwards. Angular lamella much lower and more slender than the parietal one. Upper palatal plica short and located slightly above the palatal tubercle. First interpalatal and lower palatal plicae equally strong. There is a smaller and shorter second interpalatal plica between them. Basal part of peristome with two or three low plicae (rarely one), they are situated next to each other, the one situated closer to the columellar lamella may be homologous with the subcolumellar lamella, and the other may be basal plicae. Columellar lamella is weaker than parietal and stronger than the palatal plicae, straight, pointing obliquely upwards. Supracolumellar and infraparietal denticles small, knob-like. Surface of all apertural barriers is finely granulated. Sinulus parabolic, wide, and not strongly separated from the rest of the aperture. Umbilicus wide, covers 1/3–1/4 of shell width.

##### Differential diagnosis.

See under *B.nitens* sp. nov., *B.paviei*, *B.sericata* sp. nov. and *B.tamphathai*.

##### Measurements

**(in mm, *n* = 5).**SH = 2.40–2.51; SW = 1.81–1.97; AH = 0.82–0.94; AW = 0.85–0.95.

##### Etymology.

Named for the numerous barriers in the aperture.

##### Distribution.

This species is known from four localities in Chiang Rai province, Thailand.

##### Remarks.

The population found near Ban Mae Suai is characterised by shells that lack the palatal tubercle (or have a very slight swelling indicating its presence) and smaller number of apertural barriers (only one basal and one interpalatal). However, we do not intend to regard this population as distinct due to the lack of extensive material and the fact that main shell characters are shared with *B.multidentata* sp. nov. (shell shape, surface sculpture, umbilicus width and appearance of other barriers).

#### ﻿ Bensonella multihami

Taxon classificationAnimaliaStylommatophoraHypselostomatidae

Jirapatrasilp & Tongkerd, 2024

748DFECA-EA23-5C35-BB5F-71A70DC9EA9C

**Figure 73.**
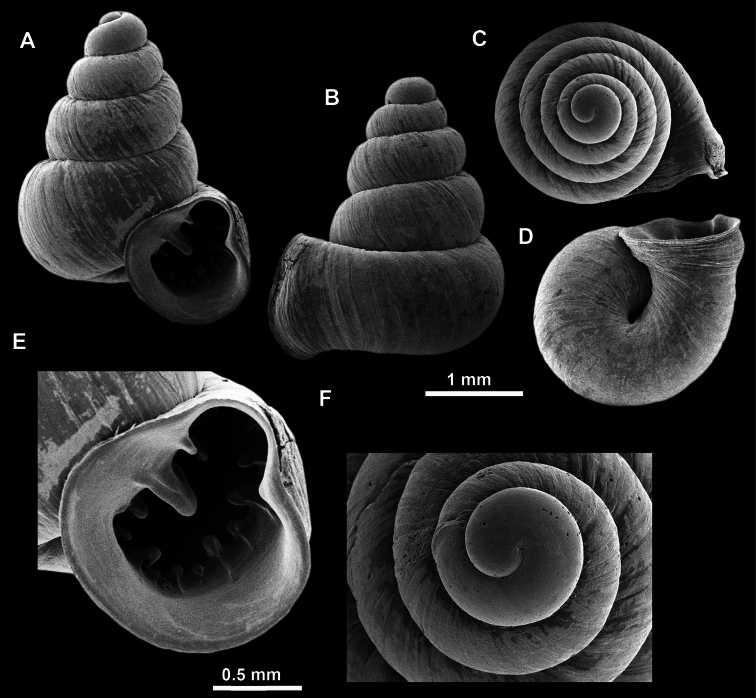
*Bensonellamultihami*, holotype (CUMZ 14366) (from Tongkerd et al. 2024) **A–D** shell **E** enlarged apertural view **F** enlarged view of the protoconch.

Figs 39P , 73 , 88 , 100

Bensonella multihami Jirapatrasilp & Tongkerd in Jirapatrasilp et al. 2024: 95–96, fig. 5.

##### Type material examined.

**Thailand** • holotype; 27. May 1997, S. Panha, J. B. Burch, P. Dumrongrojwattana leg.; CUMZ 14366.

##### Type locality.

“Doi Chiang Dao, Chiang Dao Wildlife Sanctuary, Chiang Dao District, Chiang Mai province, Thailand (19°22′046.3″N, 098°51′08.1″E, 2,015 m. a. m.s.l.)”

##### Differential diagnosis.

This species is strikingly similar to *B.hooki*, *B.lakainguta*, and *B.montawa* sp. nov. from which it differs in its blunt parietal lamella (hooked in former three)

##### Distribution.

This species is, apart from its type locality, known from one additional site: Doi Ang Khang (1400 m).

#### ﻿ Bensonella nitens

Taxon classificationAnimaliaStylommatophoraHypselostomatidae

Gojšina & Páll-Gergely sp. nov.

8AD6F479-46E8-5BFE-897F-DC3453AF4699

https://zoobank.org/BD440FC8-F087-4EA8-80B3-F9C70D61742D

**Figure 74.**
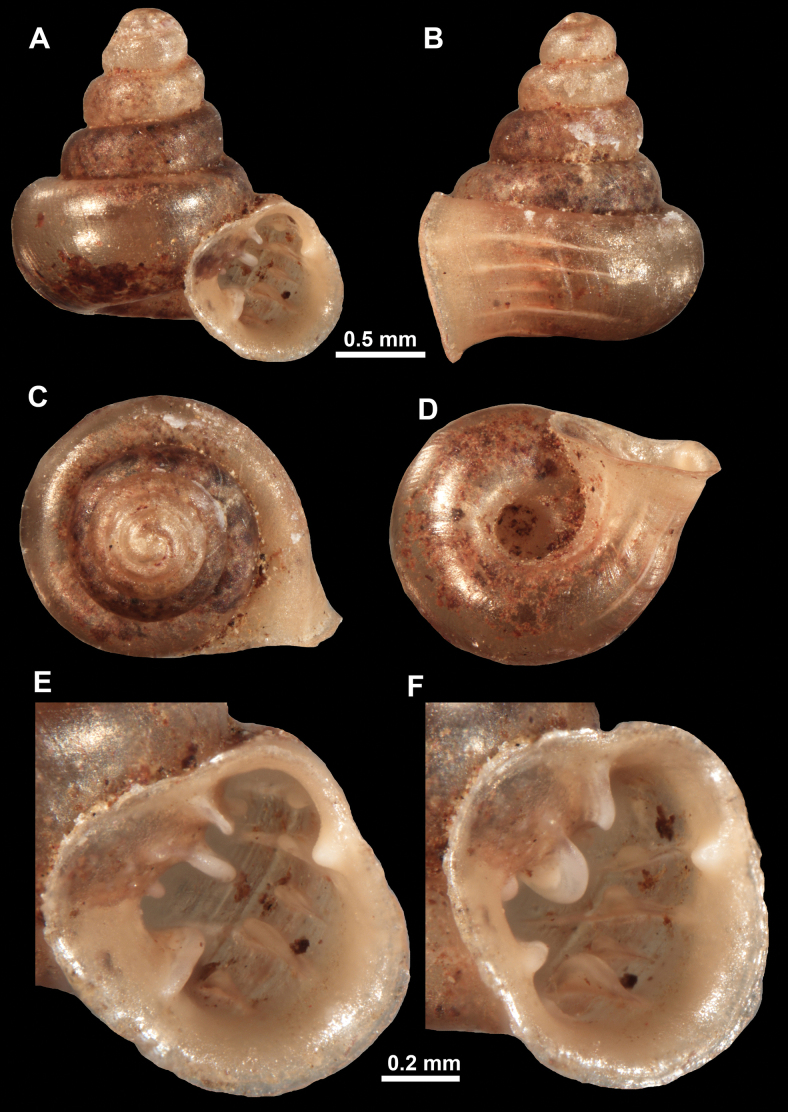
*Bensonellanitens* Gojšina & Páll-Gergely, sp. nov., holotype (UF 346975) **A–D** shell **E, F** enlarged apertural views.

**Figure 75.**
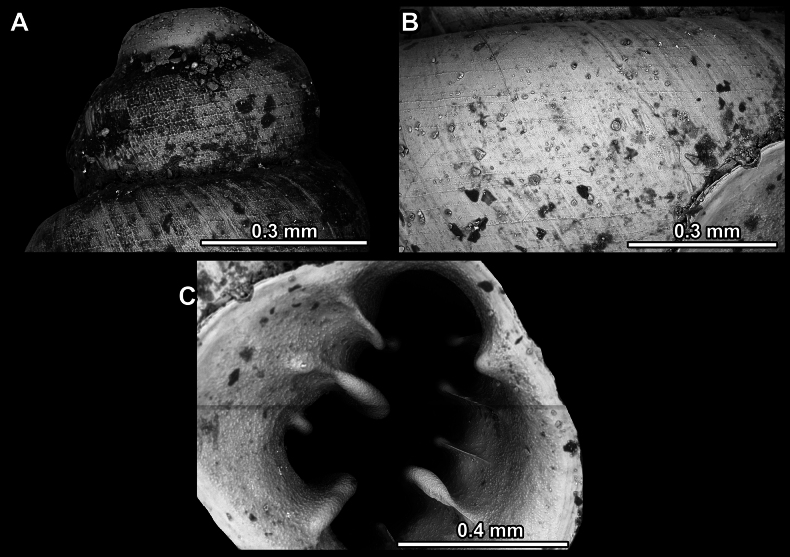
SEM imaging of *Bensonellanitens* Gojšina & Páll-Gergely, sp. nov., holotype (UF 346975) **A** protoconch surface **B** surface of the last whorl **C** apertural view.

Figs 39Q , 74 , 75 , 100

##### Type material.

***Holotype*. Thailand** • 1 shell (SH: 1.91 mm; SW: 1.77 mm); Phrae Province, 11 km W of Phrae, on Road 1023 limestone knoll, leaf litter; 18°10′41″N, 100°4′14″E; 290 m a.s.l.; 16. May 1988; F.G. Thompson leg.; locality code FGT-4447, UF 346975. ***Paratypes*. Thailand** • 2 shells; same data as for holotype; CUMZ 14440 • 45 shells; same data as for holotype; UF 591334.

##### Additional material examined.

**Thailand** • 4 shells (juveniles, not paratypes); same data as for holotype; UF 583730.

##### Type locality.

Thailand, Phrae Province, 11 km W of Phrae, on Road 1023 limestone knoll, leaf litter; 18°10′41″N, 100°4′14″E; 290 m a.s.l.

##### Diagnosis.

Shell concave-conical, glossy, last whorl strongly enlarged. Teleoconch spirally striated. Last whorl adnate to the penultimate. Aperture equipped with numerous barriers, angular and columellar lamellae reaching the peristome. Umbilicus moderately wide.

##### Description.

Shell concave-conical, brownish, consisting of 5–5.5 convex, rounded whorls separated by a deep suture. Last whorl much wider than all preceding ones, resulting in a concave-conical shell shape. Protoconch finely spirally striated initially, more prominently terminally. Its boundary with the teleoconch is not clearly visible due to the similar surface sculpture. Fine spiral striae present across the whole shell surface but quite difficult to observe on initial teleoconch whorls without SEM imaging. They are most clearly visible on the last whorl but still very fine. Spacing between the two spiral striae approximately measures the width of one or two striae. Radial growth lines extremely fine, irregularly spaced and not numerous, most clearly visible on the last and the penultimate whorls. Last whorl rounded, slightly descending (~ 10 ° compared to the shell axis), and adnate to the penultimate near the aperture. Peristome brownish, expanded but not reflected. On the parietal side of the aperture, the peristome leans on the penultimate whorl forming a thin parietal callus. Aperture equipped with numerous barriers (angular, parietal, upper palatal, two interpalatals, lower palatal, palatal tubercle, basal, columellar, and infraparietal). Parietal lamella is the strongest in the aperture, blade like and relatively high. Angular lamella discontinuous, consisting of weaker outer part (which is reaching the expanding peristome) and stronger (higher) inner part. Palatal plicae almost all equidistant. Upper palatal and first interpalatal equally strong, higher in their inner than in outer parts (but upper palatal can be weaker than the interpalatal). There is a strong, white palatal tubercle sitting on the palatal lip of the aperture, in front and between the upper palatal and interpalatal plica. Lower interpalatal plica is the weakest among them all. Lower palatal plica is the strongest among all the palatals. Sometimes, a very weak infrapalatal plica is also present and situated between the lower palatal and basal. All palatal plicae continue as slender projections towards the peristome. Basal plica small and usually the weakest in the aperture. Columellar lamella strong and oblique, leaned towards the parietal lamella. Infraparietal lamella slightly stronger than the basal plica. Surface of all apertural barriers is finely granulated. Sinulus parabolic due to the position of the angular lamella and the palatal tubercle. Umbilicus moderately wide, measuring ~ 1/5 of the shell width.

##### Differential diagnosis.

This species is similar to *Bensonellamicrodentata* sp. nov., but the barriers are more numerous and not spiniferous, the umbilicus is wider and the last whorl is more enlarged. *Bensonellanitens* sp. nov. is smaller, glossier and has a weaker surface sculpture than *B.multidentata* sp. nov. The apertural barrier arrangement is strikingly similar in these two species but *B.multidentata* sp. nov. has stronger barriers and palatal plicae without slender projections towards the peristome. These projections are always present in *B.nitens* sp. nov. See also under *B.sericata* sp. nov. and *B.tamphathai*.

##### Measurements

**(in mm, *n* = 5).**SH = 1.91–2.00; SW = 1.57–1.87; AH = 0.86–0.96; AW = 0.86–0.94.

##### Etymology.

The specific epithet refers to the shiny shell surface.

##### Distribution.

This species is known only from the type locality.

#### ﻿ Bensonella nordsiecki

Taxon classificationAnimaliaStylommatophoraHypselostomatidae

Jirapatrasilp & Tongkerd, 2024

A705D508-F16E-53AF-899F-5E6715BBC51A

Figs 39R , 76 , 100

Bensonella nordsiecki Jirapatrasilp & Tongkerd in Jirapatrasilp et al. 2024: 91–93, fig. 3.

##### Type material examined.

**Thailand** • holotype; 27. May 1997, S. Panha, J. B. Burch, P. Dumrongrojwattana leg.; CUMZ 14372.

##### Type locality.

“Doi Chiang Dao, Chiang Dao Wildlife Sanctuary, Chiang Dao District, Chiang Mai province, Thailand (19°22′046.3″N, 098°51′08.1″E, 2,015 m. a.m.s.l.)”.

##### Differential diagnosis.

This species is much more ovoid (less slender and triangular) than *Bensonelladracula* sp. nov. which also has a strong second palatal tubercle (very weak or absent in *B.nordsiecki*). The umbilicus is wider in *B.dracula* sp. nov. See also under *B.obex* sp. nov.

**Figure 76.**
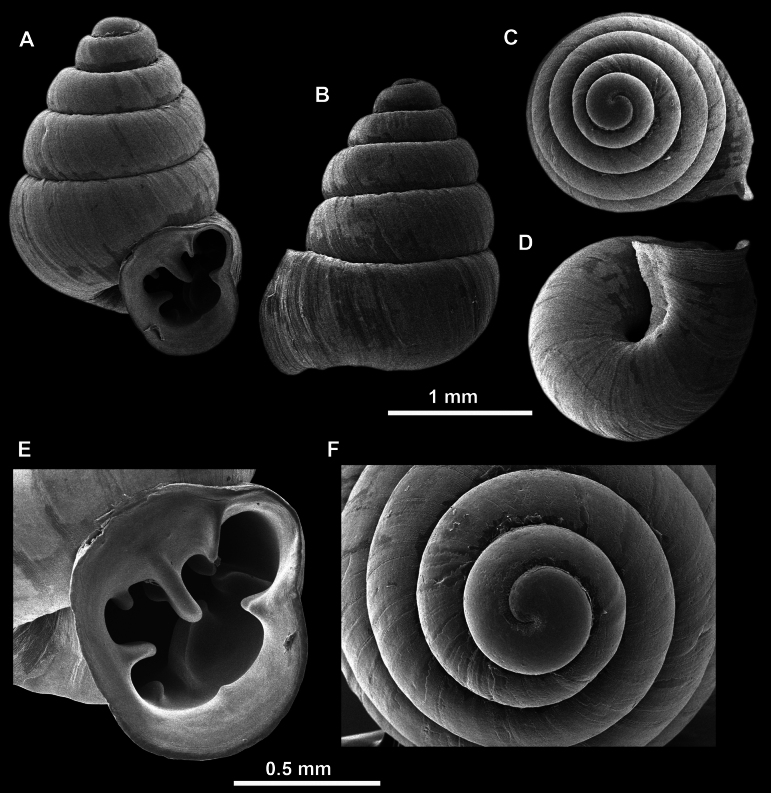
*Bensonellanordsiecki*, holotype (CUMZ 14372) (from Tongkerd et al. 2024) **A–D** shell **E** enlarged apertural view **F** enlarged view of the protoconch.

##### Distribution.

This species is known only from the type locality, with an altitude range from 605 m to 2015 m.

#### ﻿ Bensonella obex

Taxon classificationAnimaliaStylommatophoraHypselostomatidae

Gojšina, Hunyadi & Páll-Gergely sp. nov.

C2E17F1C-DC7F-5F8C-A422-2653DDAB8757

https://zoobank.org/F66E9F17-3824-4452-8EDA-DE95EF593A7A

**Figure 77.**
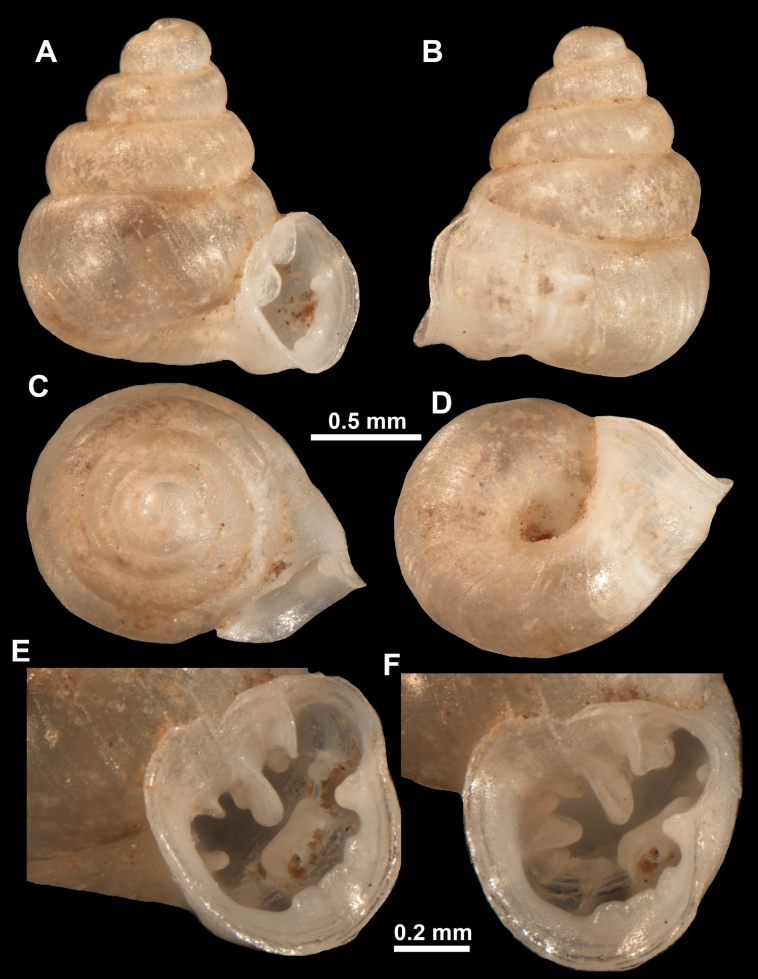
*Bensonellaobex* Gojšina, Hunyadi & Páll-Gergely, sp. nov., holotype (CUMZ 14441) **A–D** shell **E–F** enlarged apertural views.

**Figure 78.**
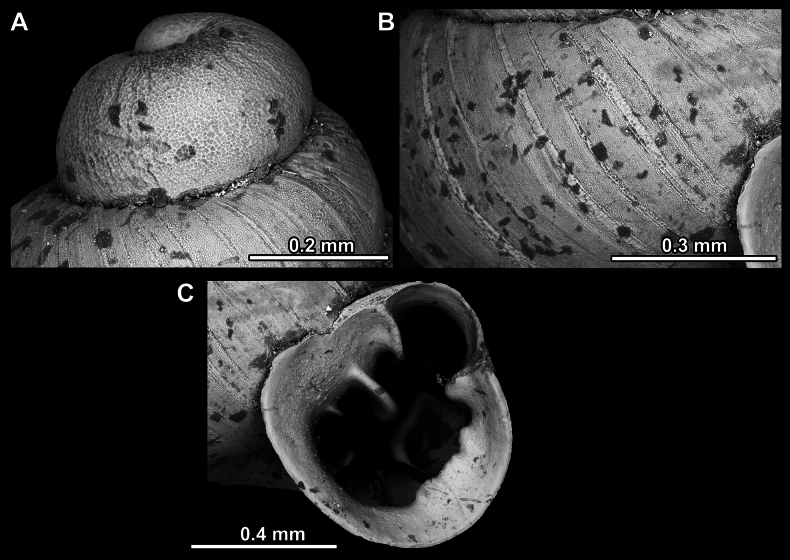
SEM imaging of *Bensonellaobex* Gojšina, Hunyadi & Páll-Gergely, sp. nov., holotype (CUMZ 14441) **A** protoconch surface **B** surface of the last whorl **C** apertural view.

Figs 39S , 77 , 78 , 97

##### Type material.

***Holotype*. Myanmar** • 1 shell (SH: 1.8 mm, SW: 1.5 mm); Shan State, 5.7 km south-southwest from Pinlaung, Wingabar Taung; 20°4.152'N, 96°46.232'E; 1510 m a.s.l.; 04 Oct. 2018; A. Hunyadi, K. Okubo & J.U. Otani leg.; CUMZ 14441. ***Paratypes*. Myanmar** • 32 shells; same data as for holotype; coll. HA.

##### Additional material examined.

**Myanmar** • 12 shells; Shan State, Pinlaung centre N 7.5 km, Tar Kge, near ”Big Bang Cave”; 20°10.273'N, 96°47.442'E; 1540 m a.s.l.; A. Hunyadi, K. Okubo & J.U. Otani leg.; coll. HA • 4 shells; Shan State, Pinlaung centre SSW 11 km – Laneli Bridge, Nam Pam, near “Upper Spider Cave”; 20°2.114'N, 96°45.728'E; 1420 m a.s.l.; 04 Oct. 2018; A. Hunyadi, K. Okubo & J.U. Otani leg.; coll. HA.

##### Type locality.

Myanmar, Shan State, 5.7 km south-southwest from Pinlaung, Wingabar Taung; 20°4.152'N, 96°46.232'E; 1510 m a.s.l.

##### Diagnosis.

*Bensonella* species with a pitted protoconch and conical-ovoid shell. Last whorl slightly ascending near the aperture. Peristome usually wrinkled due to several tubercles. Transversal plica strong and very weakly concave.

##### Description.

Shell conical-ovoid, consisting of 4.5–5 rounded, convex, regularly increasing whorls separated by a deep suture. Shell colour slightly weathered in the holotype but probably light brownish. Protoconch consisting of 1.75 whorls, finely pitted or sometimes spirally striated, especially terminally. Teleoconch finely pitted, with a pasty sculpture and without visible spiral striae but with coarsely and irregularly spaced radial growth lines. Last whorl enlarged, adnate to the penultimate and slightly ascending near the aperture (~ 20–25 ° compared to the shell axis). Aperture profile opisthocline to the shell axis. Peristome white and expanded (at columellar but especially at the parietal side where it forms a distinct callus) but not reflected. Aperture equipped with numerous barriers. Parietal lamella strong and high, almost reaching the expanding peristome. It sometimes has a heart-like appearance due to the distinct sinuation in the middle. Angular lamella weakly wavy, slightly weaker than the parietal but longer, reaching the expanding peristome. It also has a sinuation as in the parietal lamella but weaker. Upper palatal plica very short and moderately strong. Transversal plica strong, slightly concave on the front surface. A strong palatal tubercle is sitting in front and between the upper palatal and transversal plica. Below this tubercle, there are several more tubercle-like swellings which give the peristome a wrinkled appearance. Infrapalatal plica elongated and low. Basal plicae number ranging from one to four, similarly shaped as the infrapalatal plica. Subcolumellar lamella (if present) shares the same appearance as the former two. Columellar lamella moderate, ~ ½ as strong as the parietal. Supracolumellar lamella present but can be strong or quite weak. Infraparietal lamella slightly weaker than the columellar or even half as strong. Surface of all apertural barriers is finely granulated. Sinulus relatively narrow, small, and distinctly separated from the rest of the aperture. Umbilicus very narrow, measuring ~ 1/10 of the shell width, slightly elongated.

##### Differential diagnosis.

*Bensonellanordsiecki* has a smooth protoconch and more ovoid shell. The transversal plica in *B.nordsiecki* is without a concave frontal surface. *Bensonelladracula* sp. nov. is less ovoid, does not have a wrinkled peristome (but two palatal tubercles) and has a wider umbilicus.

##### Measurements

**(in mm, *n* = 10).**SH = 1.42–1.8; SW = 1.36–1.5; AH = 0.61–0.81; AW = 0.63–0.70.

##### Etymology.

The specific epithet comes from a Latin word for a barricade (*obex*), which refers to the transversal palatal plica in the form of a barrier/obstacle. To be used as a noun in apposition.

##### Distribution.

This species is known from three localities around Pinlaung, Shan State.

#### ﻿ Bensonella pahpetensis

Taxon classificationAnimaliaStylommatophoraHypselostomatidae

(Saurin, 1953) comb. nov.

1AD8DA2A-599D-566D-B664-956EEFFC3F2B

Figs 39T , 79 , 100

Boysidia pahpetensis Saurin, 1953: 116, fig. 2, pl. 4, fig. 5a–c.
Boysidia pahpetensis — Inkhavilay et al. 2019: 59, fig. 26C; Inkhavilay and Sutcharit 2024: 442, fig. 2A.

##### Type material examined.

**Laos** • 1 syntype; Pah Hia, Laos; collector unknown; MNHN-IM-2000-33880.

##### Type locality.

“environs du village méo de Pah Hia, à 100 kilomètres au Sud de Xieng-Khouang, chef-lieu de la province du Tran Ninh, Laos” (probably refers to Ban Namthong, Longchaeng District, Xaisomboun Province, Laos, see Páll-Gergely et al. 2016).

##### Differential diagnosis.

This species is unique due to the presence of one concrescent barrier on the parietal side in combination with the palatal tubercle typical for the genus *Bensonella*.

##### Distribution.

This species in known only from the type locality.

##### Remarks.

*Bensonellapahpetensis* is not a typical representative of its genus due to the concrescent angular and parietal lamellae. However, we have placed it in this genus because of the presence of the palatal tubercle as well as the triangular-conical shell shape.

**Figure 79.**
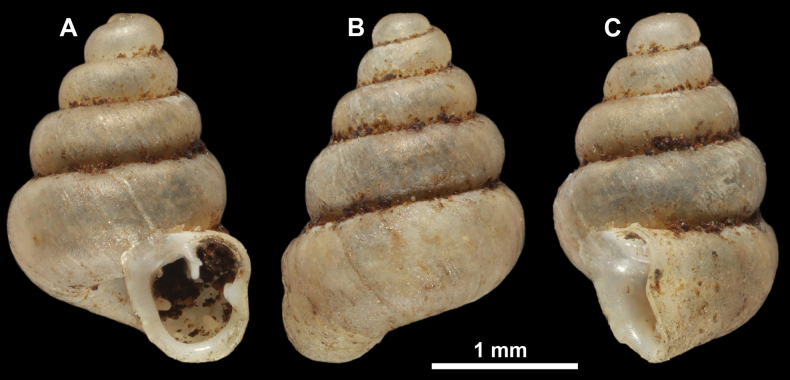
*Bensonellapahpetensis*, syntype (MNHN-IM-2000-33880 (from Inkhavilay and Sutcharit 2024)) **A–C** shell.

#### ﻿ Bensonella palatotridens

Taxon classificationAnimaliaStylommatophoraHypselostomatidae

Jirapatrasilp & Tongkerd, 2024

C4082344-88FD-516C-B093-F4F379DCA9C0

Figs 39U , 80 , 100

Bensonella palatotridens Jirapatrasilp & Tongkerd in Jirapatrasilp et al. 2024: 93, fig. 4.

##### Type material examined.

**Thailand** • holotype; 27. May 1997, S. Panha, J. B. Burch, P. Dumrongrojwattana leg.; CUMZ 14370.

##### Type locality.

“Doi Chiang Dao, Chiang Dao Wildlife Sanctuary, Chiang Dao District, Chiang Mai province, Thailand (19°22′046.3″N, 098°51′08.1″E, 2,015 m. a.m.s.l.)”.

##### Differential diagnosis.

This species is characterised by three plicae on the palatal side of the aperture. See under *B.paviei* and *B.tamphathai*.

**Figure 80.**
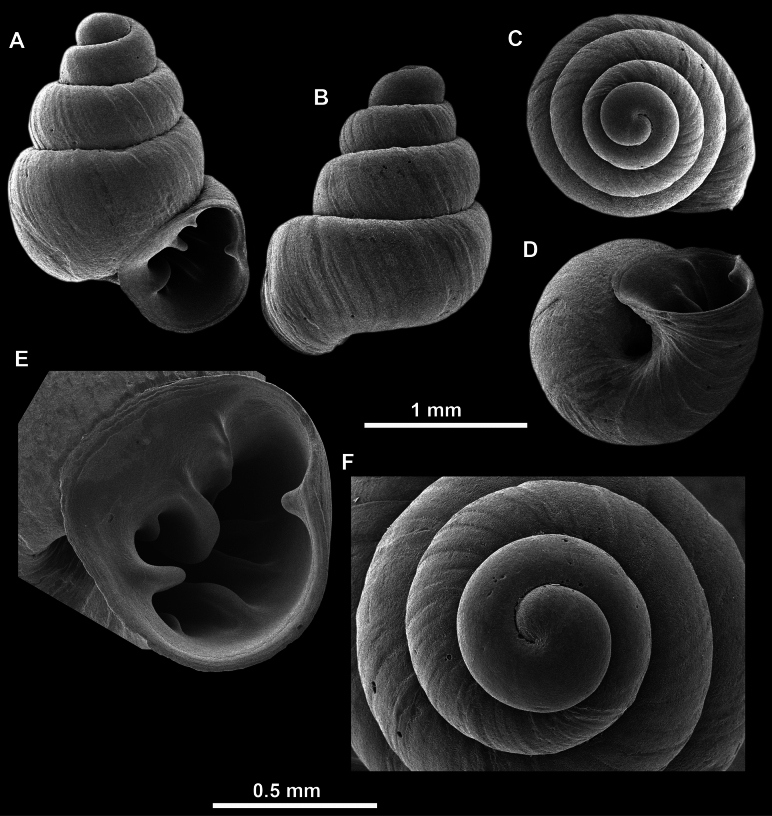
*Bensonellapalatotridens*, holotype (CUMZ 14370) (from Jirapatrasilp et al. 2024) **A–D** shell **E** enlarged apertural view **F** enlarged view of the protoconch.

##### Distribution.

This species is known only from the type locality.

#### ﻿ Bensonella pangmapaensis

Taxon classificationAnimaliaStylommatophoraHypselostomatidae

(Panha & J. B. Burch, 2002)

601594F9-B847-5CC9-882C-68E6D3EFCFE1

Figs 39V , 81 , 100

Paraboysidia pangmapaensis Panha & Burch, 2002c: 87, fig. 5.
Paraboysidia pangmapaensis — Panha and Burch 2008: 114, fig. 97; Dumrongrojwattana and Wongkamhaeng 2024: 324.

##### Material examined.

**Thailand** • 6 shells; Mae Hong Son Province, Mae Hong Son, Road 1095; 19°25'N, 97°58'E; 390 m a.s.l.; 22 Mar. 1988; K. Auffenberg leg.; locality code KA-0596; UF 345697 • 5 shells; Mae Hong Son Province, Mae Hong Son, Road 1095; 19°25'N, 97°58'E; 390 m a.s.l.; 22 Mar. 1988; K. Auffenberg leg.; locality code KA-0595B; UF 345721 • 7 shells; Mae Hong Son Province, 21 km N Mae Hong Son, Fish Cave; 19°26'N, 97°58'E; 380 m a.s.l.; 22 Mar. 1988; K. Auffenberg leg.; locality code KA-0593; UF 345671 • 20 shells; Mae Hong Son Province, 35.5 km NNE of Mae Hong Son on road to Ban Huai Phung off Road 1095; 19°29'N, 97°58'E; 450 m a.s.l.; 21 Mar. 1988; K. Auffenberg leg.; locality code KA-0591; UF 345665 • 34 shells; Mae Hong Son Province, 35.1 km S Mae Hong Son, 1.7 km E of Route 108; 19°5'N, 97°58'E; 850 m a.s.l.; 23 Mar. 1988; K. Auffenberg leg.; locality code KA-0579; UF 345727.

##### Type locality.

“Lod cave, Pang Ma Pa District, Mae Hong Son Province, 19°29'36"N, 98°17'18"E and 19°34′03″N, 98°16′41″E, 800 meters elevation…” (Thailand).

##### Differential diagnosis.

This species is clearly distinguishable by its rough sculpture showing the spiralling pattern in combination with hooked basal and palatal plicae, as well as the inner part of the angular lamella.

**Figure 81.**
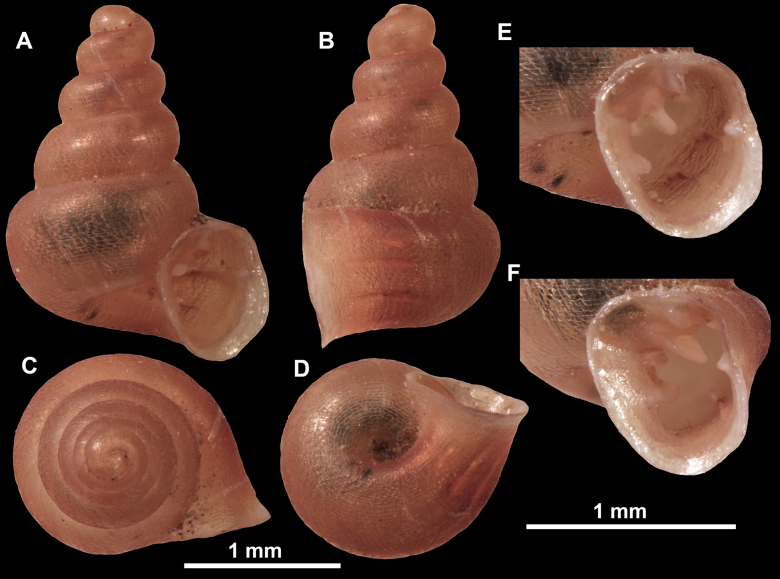
*Bensonellapangmapaensis* from Mae Hong Son province (UF 345697) **A–D** shell **E, F** enlarged apertural views.

##### Distribution.

This species is known from several sampling sites, all located in Mae Hong Son province where the type locality is also situated.

#### ﻿ Bensonella paviei

Taxon classificationAnimaliaStylommatophoraHypselostomatidae

(Bavay & Dautzenberg, 1912)

3A2619A2-810D-57C7-A307-68F2911CAA88

**Figure 82.**
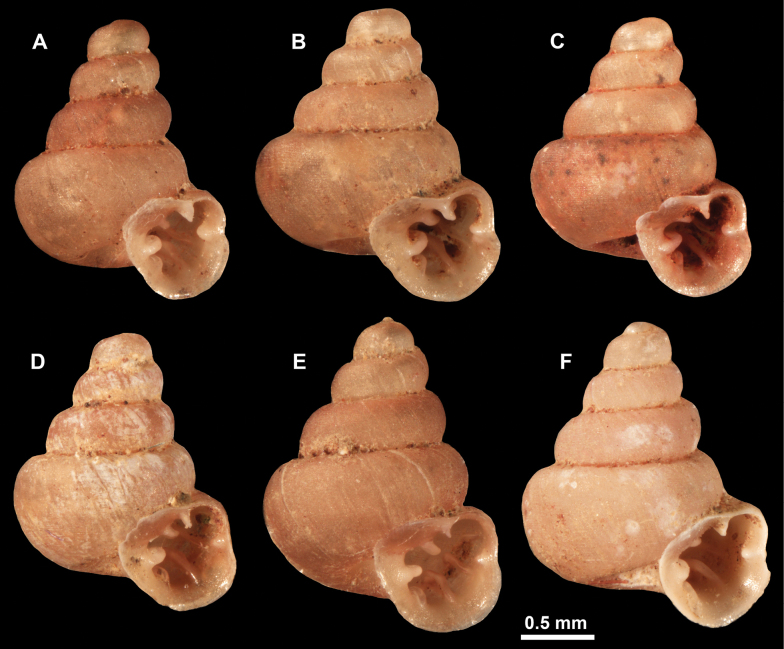
*Bensonellapaviei* from different localities **A** Thailand, Chiang Mai province (coll. HA) **B** Laos, Udomxai province (coll. HA) **C** Laos, Luang Prabang province (coll. HA) **D–F** Vietnam, Son La province (coll. HA).

**Figure 83.**
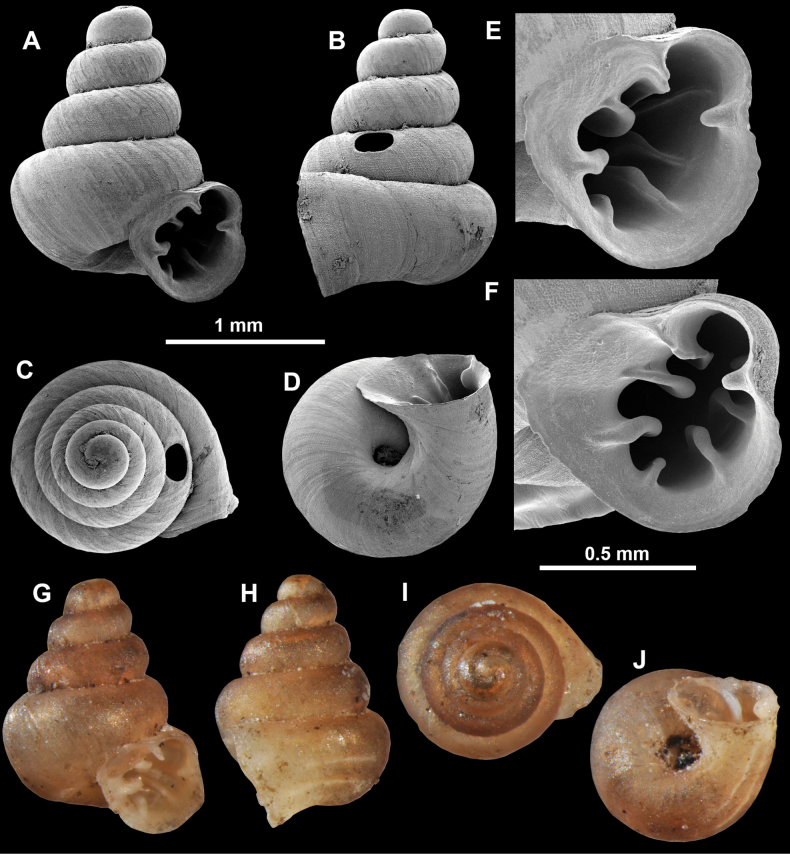
*Bensonellapaviei***A–F** holotype of *P.nabhitabhatai* (CUMZ ver. 064) **G–J** syntype of *B.paviei* (MNHN-IM-2000-35158 (from Páll-Gergely and White 2023)) **A–D, G–J** shell **E, F** enlarged apertural views.

Figs 39W , 82 , 83 , 100

Boysidia paviei Bavay & Dautzenberg, 1912: 20, pl. 3, figs 4–6.
Boysidia (Paraboysidia) paviei — Pilsbry 1917: 203–204, pl. 35, figs 7–8; Schileyko 1998: 138, fig. 156; Chen et al. 1999: 1; Schileyko 2011: 2.
Paraboysidia paviei — van Benthem Jutting 1950: 39.
Paraboysidia nabhitabhatai Panha & Burch, 2002c: 81–84, fig. 3. syn. nov.
Paraboysidia nabhitabhatai — Panha and Burch 2008: 113–114, fig. 96.
Paraboysidia anguloobtusus Inkhavilay & Panha in Inkhavilay et al. 2016: 215–217, figs 1, 2D–F, 4B. syn. nov.
Paraboysidia anguloobtusa — Inkhavilay et al. 2019: 61, fig. 26F.
Bensonella paviei — Páll-Gergely and White 2023: 2015, fig. 3f–k.
Bensonella anguloobtusa — Tongkerd et al. 2024: 171–174, figs 6B, 7A, 13G.
Bensonella nabhitabhatai — Dumrongrojwattana and Wongkamhaeng 2024: 324.

##### Type material examined.

**Vietnam** • 1 syntype; Pac-Kha: Long-ping; C. Messager leg.; MNHN-IM-2000-35158. **Thailand** • holotype of *P.nabhitabhatai*; 1998; S. Panha, P. Dumrongrojwattana, C. Sutcharit, S. Tumpeesuwan leg.; CUMZ ver. 064.

##### Additional material examined.

**Vietnam** • 4 shells; Son La Province, 27 km east-southeast from centre of Phu Yen, Muong Do, Ban Han Mot, southern part of the village; 21°11.731'N, 104°47.129'E; 800 m a.s.l.; 06 Feb. 2020; A. Hunyadi, J.U. Otani & S.V. Pham leg.; coll. HA • 23 shells; Son La Province, Moc Chau district, Van Ho, northeastern edge of Pa Cop towards Bo Nhang; 20°46.001'N, 104°45.203'E; 980 m a.s.l.; 10 Feb. 2020; A. Hunyadi, H.V. Luong, J.U. Otani & S.V. Pham leg.; coll. HA • 31 shells; Son La Province, Yen Chau district, Xa Chieng On, Ban Tram Hoc, Hang Nha Nhung, vicinity of the cave; 20°59.483'N, 104°11.270'E; 970 m a.s.l.; 09 Feb. 2020; A. Hunyadi, H.V. Luong, J.U. Otani & S.V. Pham leg.; coll. HA • 110 shells; Son La Province, Quynh Nhai district, 20 km east from cross towards Thuan Chau, Chieng Khoang, cave above the village; 21°33.441'N, 103°40.909'E; 315 m a.s.l.; 07 Feb. 2020; A. Hunyadi, H.V. Luong, J.U. Otani & S.V. Pham leg.; coll. HA • 10 shells; Son La Province, Moc Chau district, Long Luong, Ban Tan Lap, northern edge of the village; 20°46.835'N, 104°50.752'E; 1165 m a.s.l.; 05 Feb. 2020; A. Hunyadi, H.V. Luong, J.U. Otani & S.V. Pham leg.; coll. HA • 4 shells; Sơn La Province, Thuận Châu district, 900 m from centre of Co Mạ, rock wall above the road; 21°21.186'N, 103°31.676'E; 1300 m a.s.l.; 08 Feb. 2020; A. Hunyadi leg.; **Laos** • 37 shells; Udomxai Province, 6.5 km southeast from centre of Na Mor towards Udomxai, Ban Nathong, Tham Nathong, above cave spring; 20°52.369'N, 101°46.981'E; 635 m a.s.l.; 07 Oct. 2019; A. Hunyadi leg.; coll. HA • 1 shell; Luang Prabang Province, 28.5 km south from Pak Mong towards Luang Prabang, northern bank of Nam Nga, Tham Nam Nga; 20°22.220'N, 102°20.078'E; 350 m a.s.l.; 09 Oct. 2019; A. Hunyadi leg.; coll. HA • 45 shells; Luang Prabang Province, 10.3 km from centre of Luang Prabang towards Xiang Ngeum, east, 1800 m on a path, Phou Xuang, eastern mountain slope; 19°51.526'N, 102°11.100'E; 730 m a.s.l.; 10 Oct. 2019; A. Hunyadi leg.; coll. HA • 26 shells; Luang Prabang Province, 12 km from centre of Nong Khiaw towards Pak Xeng, northeast from Ban Huai Lek, left side of the road; 20°32.585'N, 102°41.161'E; 350 m a.s.l.; 05 Oct. 2019; A. Hunyadi leg.; coll. HA • 103 shells; Luang Prabang Province, 2.8 km northeast from Phou Khoun, rock wall above the cave; 19°26.787'N, 102°26.338'E; 1200 m a.s.l.; 11 Oct. 2019; A. Hunyadi leg.; coll. HA • 113 shells; Udomxai Province, 10 km southeast from centre of Na Mor, 3.8 km east-northeast from Na Xay, northern rock wall; 20°53.371'N, 101°48.963'E; 660 m a.s.l.; 07 Oct. 2019; A. Hunyadi leg.; coll. HA • 5 shells; Xieng Khouang Province, ca 15 km SW of Ban Ko Kieng, ca 19 km SW of Vieng Thong, on the left side of Nam Khan, under rocks and logs in old forest; 19°56.264'N, 103°15.763'E; 570 m a.s.l.; 27 Oct. 2006; A. Abdou, I.V. Muratov leg.; MNHN-IM-2012-27287 • 3 shells; Luang Prabang Province, ca 6 km E of Muang Xiang Ngeun, under rocks in old secondary forest under cliff; 19°44.690'N, 102°15.193'E; 390 m a.s.l.; 21 Nov. 2006; A. Abdou, I.V. Muratov leg.; MNHN-IM-2012-27288 • 2 shells; Luang Prabang Province, Phou Xuang mountain, ca 1.5 km NE of Ban Lak Sip, ca 5 km SE of Luang Prabang, under rocks and logs in old secondary forest under cliff; 19°51.605'N, 102°11.081'E; 640 m a.s.l.; 24 Nov. 2006; A. Abdou, I.V. Muratov leg.; MNHN-IM-2012-27270 • 1 shell; Luang Prabang Province, ca 18 km SE of Muang Xiang Ngeun, on the left side of Nam Khan, under rocks and logs in old forest; 19°40.931'N, 102°19.743'E; 455 m a.s.l.; 30 Oct. 2006; A. Abdou, I.V. Muratov leg.; MNHN-IM-2012-27285 • 9 shells; Xieng Khouang Province, ca 30 km WNW of Phonsavan, ca 47 km ENE of Phou Khoun, limestone, black soil in limestone pockets, clay, under rocks in old secondary forest at base of cliff near cave; 19°32.019'N, 102°51.901'E; 1131 m a.s.l.; 15 Nov. 2006; A. Abdou, I.V. Muratov leg.; MNHN-IM-2012-27272 • 6 shells; Luang Prabang Province, just NE of Phou Khoun, under rocks in old secondary forest above large cave; 19°26.784'N, 102°26.290'E;1177 m a.s.l.; 15 Nov. 2006; A. Abdou, I.V. Muratov leg.; MNHN-IM-2012-27289 • 12 shells; Xieng Khouang Province, Plane of Jars, Site 1, ca 6 km S of Phonsavan, limestone, clay, under rocks near small trees under cliff surrounded by grassland; 19°25.819'N, 103°09.216'E; 1097 m a.s.l.; 14 Nov. 2006; I.V. Muratov leg.; MNHN-IM-2012-27286 • 12 shells; Luang Prabang Province, ca 17 km SE of Muang Xiang Ngeun, along small stream on the left side (ca 2 km S) of Nam Khan, on and under rocks and logs in old forest with banana; 19°40.188'N, 102°18.538'E; 525 m a.s.l.; 31 Oct. 2006; A. Abdou, I.V. Muratov leg.; MNHN-IM-2012-27273 • 22 shells; Luang Prabang Province, ca 6 km N of Phou Khoun, under rocks in dry secondary forest under and above cliff; 19°29.653'N, 102°24.470'E; 1244 m a.s.l.; 16 Nov. 2006; A. Abdou, I.V. Muratov leg.; MNHN-IM-2012-27271 • 28 shells; Xieng Khouang Province, ca 18 km SW of Ban Ko Kieng, ca 22 km SW of Vieng Thong, on the right side of Nam Khan, under rocks and logs at base of cliff in secondary forest; 19°54.842'N, 103°14.392'E; 560 m a.s.l.; 28 Oct. 2006; A. Abdou, I.V. Muratov leg.; MNHN-IM-2012-27284. **Myanmar** • 1 shell; Mandalay, Anesakhan, Dat Taw Gyaint Waterfall; 21°58.760'N, 96°23.116'E; 610 m a.s.l.; 18 Oct. 2018; Hunyadi leg; coll. HA. **Thailand** • 51 shells; Chiang Mai Province, northeastern side of Doi Chiang Dao, 2 km northwest from Wat Tham Chiang Dao; 19°24.016'N, 98°54.680'E; 835 m a.s.l.; 07 Feb. 2015, A. Hunyadi leg.; coll. HA • 3 shells; Mae Hong Song Province, 10.2 km WNW of Soppong, road 1095; 19°33'N, 98°3'E; 820 m a.s.l.; 23 Mar. 1998; K. Auffenberg leg.; locality code KA-0586; UF 345620.

##### Type localities.

“Pac-Kha”, Vietnam (*B.paviei*); “Limestone wall outside of Kao Rao. Cave, Vieng Phouka District, Luang Namtha Province, Laos (20°43'30.1"N, 101°9'4.3"E), 732 m amsl” (*Paraboysidiaanguloobtusus*); “Phadevada, Phukhieo Wildlife Sanctuary, Chaiyapumi Province, 16°3'20"N, 101°34'14"E, 110 meters elevation…” (*P.nabhitabhatai*).

##### Differential diagnosis.

This species is smaller, with less numerous apertural barriers and a narrower umbilicus than *Bensonellamultidentata* sp. nov. *B.palatotridens* is not spirally striated. This species differs from *B.microdentata* sp. nov. in the less glossy shell surface, stronger apertural barriers and non-spiniferous aperture surface (including the barriers). See also under *B.tamphathai*. This species is also similar to *B.nitens* but can be separated by the much less glossy shell surface and stronger and fewer apertural barriers.

##### Distribution.

This species is known from several localities in Laos (Xieng Khouang, Udomxai and Luang Prabang provinces), Vietnam (Son La province), Myanmar (Shan State, see Tongkerd et al. 2024, Mandalay) as well as Thailand (Chiang Mai, Mae Hong Son and Chaiyapumi provinces).

##### Remarks.

*Paraboysidiaanguloobtusa* described from a Kao Rao Cave, Vieng Phouka District, Luang Namtha Province, Laos (20°43'30.1"N, 101°9'4.3"E) and *Paraboysidianabhitabhatai* described from Phadevada, Phukhieo Wildlife Sanctuary, Chaiyapumi Province, Thailand (16°3'20"N, 101°34'14"E) are both herein considered junior synonyms of *B.paviei* since no morphological differences were observed. Inkhavilay et al. (2016) stated that *P.anguloobtusa* has a different shell shape than *B.paviei* and that it has a blunt angular lamella. However, the shell shape of both species is the same (conical to slightly concave-conical) and both species have a blunt angular lamella. Other differences mentioned for apertural barriers (infraparietal situated closer to the parietal and angular lamellae, suprapalatal absent, lamellae more twisted) are considered minor intraspecific variation. The umbilicus in *B.paviei* is not widely opened as stated by Inkhavilay et al. (2016) but rather narrow and of the same appearance as that in *Paraboysidiaanguloobtusa*. Sometimes, the last whorl of *B.paviei* is more or less enlarged, but the apertural barrier arrangement is largely consistent. Spiral striation can vary in strength but is always present.

#### ﻿ Bensonella perfecta

Taxon classificationAnimaliaStylommatophoraHypselostomatidae

Gojšina & Páll-Gergely sp. nov.

8E04333E-CE0E-542B-AEA5-ACF60204DC97

https://zoobank.org/D0B00AA9-89E1-40F3-A8BE-D7EE9E7CE0E9

**Figure 84.**
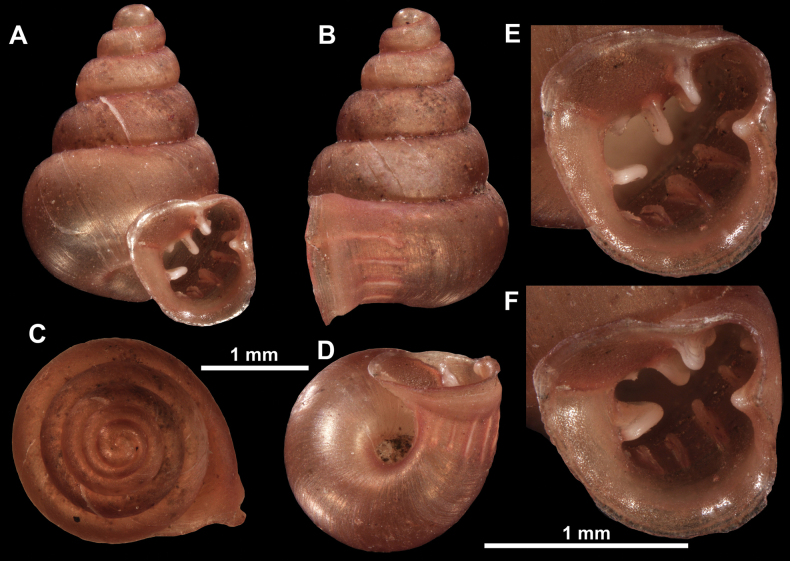
*Bensonellaperfecta* Gojšina & Páll-Gergely, sp. nov., holotype (MNHN-IM-2000-39829) **A–D** shell **E, F** enlarged apertural views.

**Figure 85.**
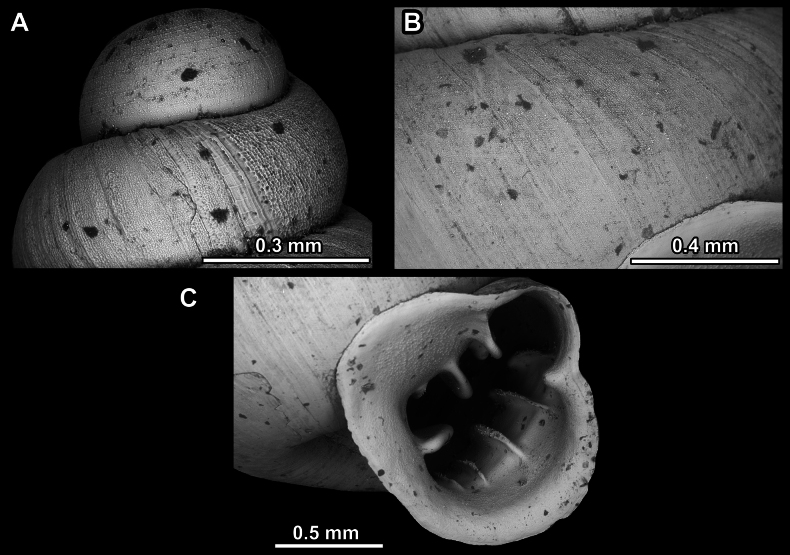
SEM imaging of *Bensonellaperfecta* Gojšina & Páll-Gergely, sp. nov., holotype (MNHN-IM-2000-39829) **A** protoconch surface **B** surface of the last whorl **C** apertural view.

Figs 39X , 84 , 85 , 95

##### Type material.

***Holotype*. Laos** • 1 shell (SH: 2.9 mm; SW: 2.1 mm); Central Laos, Xieng Khouang Province, ca 30 km WNW of Phonsavan, ca 47 km ENE of Phou Khoun, limestone, black soil in limestone pockets, clay, under rocks in old secondary forest at base of cliff; 19°32.019'N, 102°51.901'E; 1131 m a.s.l.; 15 Nov. 2006; A. Abdou, I.V. Muratov leg.; MNHN-IM-2000-39829.

***Paratypes*. Laos** • 2 shells; same data as for holotype; CUMZ 14442; 174 shells; same data as for holotype; MNHN-IM-2000-39830.

##### Additional material examined.

**Laos** • 94 shells (damaged and juveniles, not paratypes); same data as for holotype; MNHN-IM-2012-25390.

##### Type locality.

Central Laos, Xieng Khouang Province, ca 30 km WNW of Phonsavan, ca 47 km ENE of Phou Khoun, limestone, black soil in limestone pockets, clay, under rocks in old secondary forest at base of cliff; 19°32.019'N, 102°51.901'E; 1131 m a.s.l.

##### Diagnosis.

*Bensonella* species with a conical, brownish shell that is not spirally striated. Apertural barriers numerous: five plicae (4 palatals and one basal), all weak and low. Palatal tubercle strong.

##### Description.

Shell conical, pale brown, consisting of 5–5.5 regularly increasing, rounded whorls. Protoconch consisting of 1.75 whorls, same colour as the rest of the shell, finely pitted and spirally striated. Teleoconch with fine pasty like surface sculpture and coarse radial growth lines which are irregularly spaced. Rarely, these radial lines appear as stronger, whitish streaks. A spiralling pattern is very slightly visible on the last whorl (only under SEM) although there are no spiral striae. Last whorl rounded, adnate to the penultimate and slightly descending near the aperture (~ 5–10 ° compared to the shell axis). Apertural profile slightly prosocline to the shell axis. Peristome of same colour as the rest of the shell, expanded but not reflected. There is a weak cervical crest just behind the peristome. Aperture equipped with numerous barriers. Parietal lamella strong and blade-like, not reaching the peristome. Angular lamella closer to the peristome but appears discontinuous as its outer and inner parts are separated by a distinct sinuation. These two parts are roughly equally strong. There are one or two barriers inside the sinulus. Four palatal plicae (upper palatal, interpalatal, lower palatal and infrapalatal). Upper, inter and lower palatal are moderately long and high, equally developed and equally distanced. Sometimes, upper palatal is weak and lower palatal is stronger than others. Infrapalatal plica is much weaker (lower and shorter). Palatal tubercle is strong, sitting on the palatal lip of the peristome just below the line of upper palatal plica. Basal plica situated close to the infrapalatal and equally developed or sometimes weaker. Columellar lamella stronger than in most of the congeners (quite broad and high), almost horizontal, sometimes appears slightly wavy. Infraparietal lamella moderate, weaker than other lamellae but stronger than all the plicae. Surface of all apertural barriers is finely granulated. Additionally, plicae in the palatal and basal areas as well as the infraparietal and angular lamellae are equipped with isolated but strong spines which are positioned medially. Sinulus small and distinctly separated from the rest of the aperture. Umbilicus narrow, slightly elongated, measuring ~ 1/5–1/6 of the shell width.

##### Differential diagnosis.

See under *H.frequens*.

##### Measurements

**(in mm, *n* = 5).**SH = 2.91–3.09; SW = 2.08–2.23; AH = 1.15–1.30; AW = 1.2–1.37.

##### Etymology.

This species is named for the exceptionally fine surface sculpture.

##### Distribution.

This species is known only from the type locality.

#### ﻿ Bensonella plicidens

Taxon classificationAnimaliaStylommatophoraHypselostomatidae

(Benson, 1849)

BB0DFD42-2490-5C53-80E3-F1D9D7472CA7

Figs 39Y , 86

Pupaplicidens Benson, 1849: 126.
Pupaplicidens — Küster 1852: 136, pl. 17, figs 23, 24.
Boysidia plicidens — Pfeiffer 1853: 553; Hanley and Theobald 1874: pl. 100, fig. 8; Sowerby 1876: pl. 16, fig. 151; Ancey 1881: 373; Gude 1914: 294.
Vertigo (Odontocyclas) plicidens — Adams and Adams 1858: 173.
Pupa (Scopelophila) plicidens — Albers and Martens 1860: 296.
Pupa (Odontocyclas) plicidens — Pfeiffer 1879: 350.
Bifidaria (Bensonella) plicidens — Pilsbry and Vanatta 1900: 591.
Bifidaria (Bensonella) landurensis [sic] Pilsbry, 1915: 73.
Boysidia landurensis — Pilsbry 1917: 204, pl. 35, fig. 9.
Paraboysidia landourensis — van Benthem Jutting 1950: 39.
Boysidia (Bensonella) plicidens — Chen and Zhang 2002: 693; Zhang et al. 2014: 572.
Bensonella plicidens — Hwang 2014: 17–20 (partim: fig. 5 shows B.plicidens); Páll-Gergely and White 2023: 2018–2023, figs 3a–e, 4, 5, 9a, b.
Bensonella landourensis and plicidens — Budha and Backeljau 2017. (partim: figs 2B, 3, 5A, F show B.plicidens).

##### Type material examined.

**India** • lectotype; R. McAndrew coll. (ex. W.H. Benson coll.); UMZC I.103325.

##### Type locality.

“ad Landour et Moussoorie, montibus Himalayanis”.

##### Differential diagnosis.

This species is similar to *B.lakainguta*, *B.multihami*, and *B.hooki*, but differs from all by the presence of spiral striation on the shell and absence of hooked apertural barriers. *Bensonellakaroensis* is also not spirally striated, is more ovoid with a blunter apex and shallower suture. See also under *B.spinosa* sp. nov.

**Figure 86.**
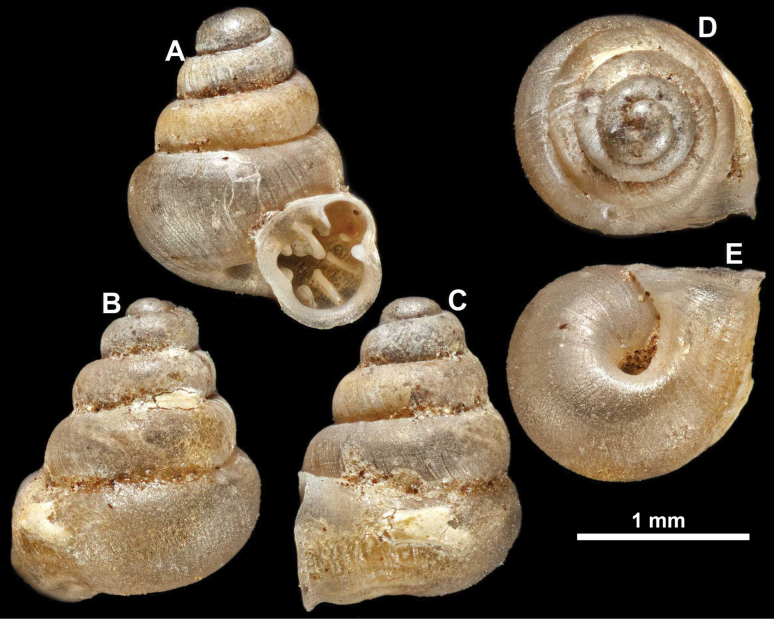
*Bensonellaplicidens*, lectotype (UMZC I.103325) **A–E** shell (from Páll-Gergely and White 2023).

##### Distribution.

SW and SE Himalaya.

#### ﻿ Bensonella sericata

Taxon classificationAnimaliaStylommatophoraHypselostomatidae

Gojšina & Páll-Gergely sp. nov.

2950672B-E126-5E3B-98FF-BFAE09F1D367

https://zoobank.org/6E048946-955A-4193-839D-A97E0153A24D

**Figure 87.**
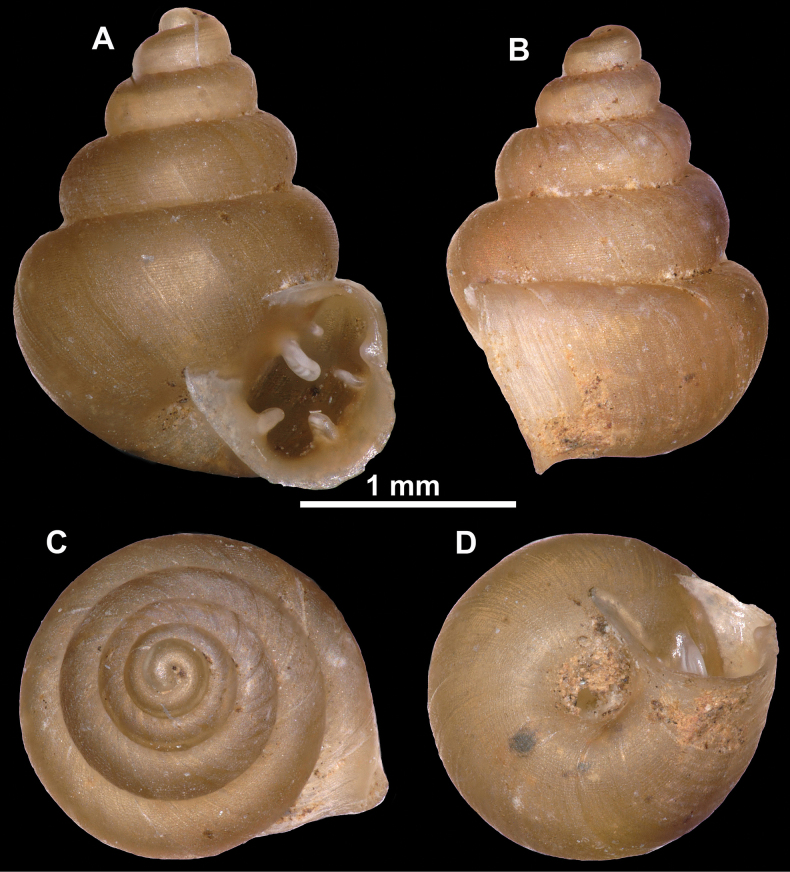
*Bensonellasericata* Gojšina & Páll-Gergely, sp. nov., holotype (UF 380270) **A–D** shell.

**Figure 88.**
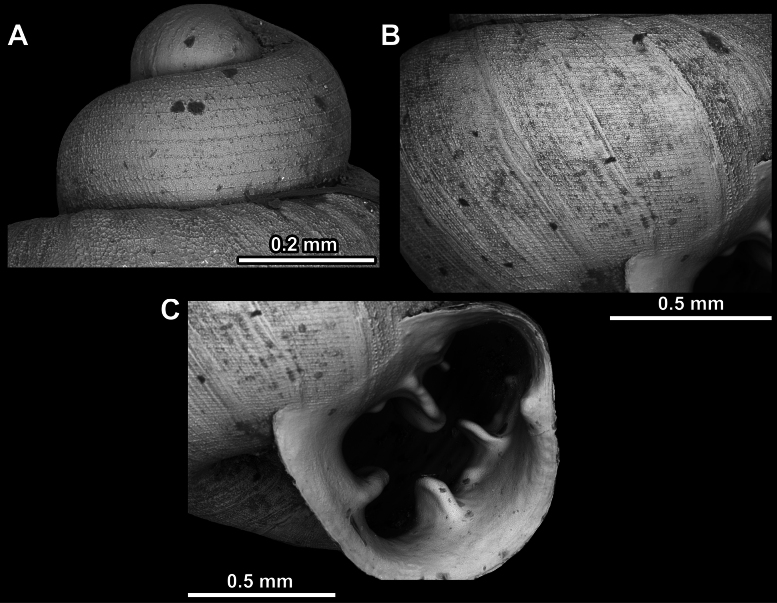
SEM imaging of *Bensonellasericata* Gojšina & Páll-Gergely, sp. nov., holotype (UF 380270) **A** protoconch surface **B** teleoconch surface **C** enlarged apertural view.

Figs 39Z , 87 , 88 , 100

##### Type material.

***Holotype*. Thailand** • 1 shell (SH: 2.38 mm, SW: 1.69 mm); Chiang Rai Province, limestone knoll, Ban Mae Song Nai, 4.0 km N, 6.0 km NW Mae Chan, limestone domes, shaded ledges; 20°11′37″N, 99°33′6″E; 520 m a.s.l.; 10. May 1988; F. G. Thompson leg.; locality code FGT-4425; UF 380270. ***Paratypes*. Thailand** • 4 shells; same data as for holotype; UF 591360.

##### Additional material examined.

**Thailand** • 5 shells (juveniles, not paratypes); same data as for holotype; UF 591368.

##### Type locality.

Thailand, Chiang Rai Province, limestone knoll, Ban Mae Song Nai, 4.0 km N, 6.0 km NW Mae Chan, limestone domes, shaded ledges; 20°11′37″N, 99°33′6″E; 520 m a.s.l.

##### Diagnosis.

A *Bensonella* species with conical to conical-ovoid shell, very dense spiral striation, and a small number of apertural barriers, only four of which are strong (parietal, upper palatal, lower palatal and columellar).

##### Description.

Shell brown, conical to conical-ovoid, whorls 4.5–5, convex, regularly increasing. Protoconch consisting of ~ 1.5 whorls, its sculpture consists of ~ 10 roughly equidistant spiral striae. Teleoconch finely, very densely spirally striated, and ornamented with a few, inconspicuous radial growth lines. Last whorl adnate to the penultimate and slightly descending near the aperture (~ 15 ° compared to the shell axis) making the aperture profile prosocline to the shell axis. Peristome slightly expanded, not reflected. A blunt, weak, knob-like palatal tubercle sits anterior and slightly below the upper palatal plica on the peristome edge. Parietal lamella is the strongest in the aperture and slightly leaned towards the palatal wall. Angular lamella weak, its outer part reduced, inner part sits deep in the aperture. Upper palatal plica in form of a very weak swelling, situated slightly above the palatal tubercle. Interpalatal plica strong, located slightly below the palatal tubercle. Lower palatal plica nearly as strong as the parietal, stronger than the interpalatal. There can be two additional swellings: one between the upper and interpalatal and one between the interpalatal and lower palatal. Basal plica absent. Columellar lamella strong and slightly oblique. Infraparietal lamella in form of indistinct swelling located close to the parietal lamella. Surface of all apertural barriers is smooth. Sinulus wide and relatively well separated from the rest of the aperture due to the strong parietal lamella. Umbilicus moderately wide, measuring 1/5 of shell width.

##### Differential diagnosis.

This species differs from *B.multidentata* sp. nov. by the less numerous apertural barriers (including the lack of the basal plica) and not concave-conical shell. The shell in *B.multidentata* sp. nov. is also more pointed. *Bensonellanitens* sp. nov. is glossier due to the very coarse spiral striae (very dense in *B.sericata* sp. nov.), concave conical and has slender and sharp projections proximally and distally from the palatal plicae. See also under *B.tamphathai*.

##### Measurements

**(in mm, *n* = 4).**SH = 2.26–2.39; SW = 1.69–1.73; AH = 0.94–1.01; AW = 0.87–0.94.

##### Etymology.

The specific epithet refers to the silky surface of the shell (as if “covered in silk”).

##### Distribution.

This species is known only from the type locality.

##### Remarks.

Coordinates of the sample point in Myanmar just next to the Thai border.

#### ﻿ Bensonella serrata

Taxon classificationAnimaliaStylommatophoraHypselostomatidae

Gojšina, Hunyadi & Páll-Gergely sp. nov.

F1476E80-5930-5973-AD61-C74A6F4A36EE

https://zoobank.org/9FB7F1F9-65DB-495B-9867-04B4F203DD49

##### Type material.

***Holotype*. Myanmar** • 1 shell (SH: 2.71 mm, SW: 1.92 mm); Shan State, 16 km from centre of Taunggyi towards Hopong, 1.5 km along road #4, Shwe Pyi Aunchonda monastery; 20°47.263'N, 97°8.239'E; 1110 m a.s.l.; 08 Oct. 2018; A. Hunyadi, K. Okubo & J. U. Otani leg.; CUMZ 14443. ***Paratypes*. Myanmar** • 15 shells; same data as for holotype; coll. HA • 13 shells; Shan State, Hopong, Sam Phu, Cave Ae-5 at ridge above village Ho Hwe; 20°41.103'N, 97°16.198'E; 02 Feb. 2019; J. Grego leg., coll. JG.

##### Additional material examined.

**Myanmar** • 3 shells (damaged, not paratypes); same data as for holotype; coll. HA • 5 shells (damaged/ juveniles, not paratypes); Shan State, Hopong, Sam Phu, Cave Ae-5 at ridge above village Ho Hwe; 20°41.103'N, 97°16.198'E; 02 Feb. 2019; J. Grego leg., coll. JG.

##### Type locality.

Myanmar, Shan State, 16 km from centre of Taunggyi towards Hopong, 1.5 km along road #4, Shwe Pyi Aunchonda monastery; 20°47.263'N, 97°8.239'E; 1110 m a.s.l.

##### Diagnosis.

*Bensonella* species with pasty shell surface, last whorl adnate to the penultimate and palatal plicae with serrated, elevated inner and very low outer parts.

**Figure 89.**
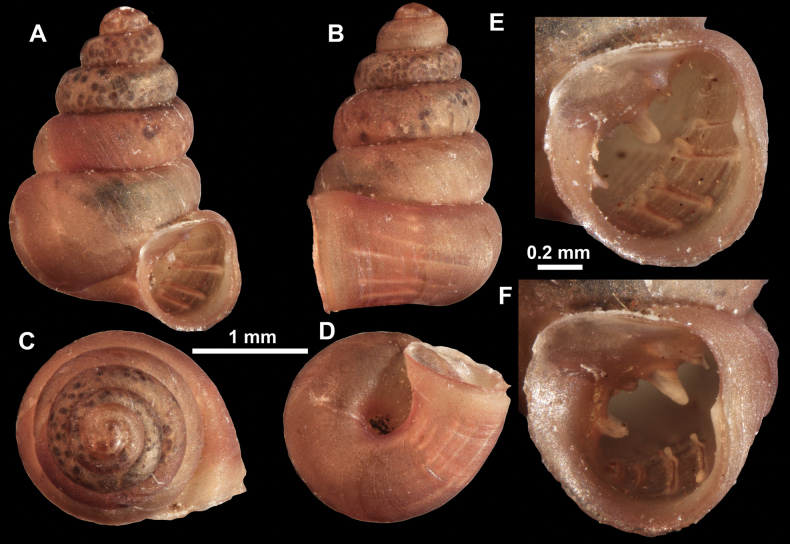
*Bensonellaserrata* Gojšina, Hunyadi & Páll-Gergely, sp. nov., holotype (CUMZ 14443) **A–D** shell **E, F** enlarged apertural views.

##### Description.

Shell triangular, slightly conical-ovoid, brown, not very glossy, consisting of 5–5.5 regularly increasing whorls separated by a deep suture. All whorls convex, rounded. Protoconch consisting of 1.5 whorls, finely pitted, showing spiralling pattern even though without clear spiral striae. The boundary between the protoconch and the teleoconch is visible due to the presence of radial growth lines on the teleoconch. Teleoconch surface finely dimpled, pasty and with less numerous, weak radial growth lines, spiral striae absent. Last whorl also rounded, adnate to the penultimate near the aperture. Last whorl slightly descending immediately behind the aperture (~ 10 ° compared to the shell axis) making the apertural profile slightly prosocline to the shell axis. Peristome weakly expanded but not reflected, its edges are thickened. Aperture equipped with numerous barriers, two of them relatively strong (parietal, columellar), others weak. Parietal lamella is the strongest and highest in the aperture. Angular lamella is the longest in the aperture, much longer than the parietal, very slender and low but continuous. Its inner and outer parts are higher than its middle part. Columellar lamella similar in length to the parietal but much weaker. There is one weak infraparietal lamella. On the palatal side, there are four main palatal plicae which are all almost equally strong and long (upper palatal, two interpalatals, and a lower palatal). They are all elevated at their inner ends and sloping towards the outer end, ending very low. These low parts are equipped with numerous small spines (serrated), thus resembling a saw. Palatal tubercle, sitting on the palatal lip, is weak but clearly present. There are several suprapalatal plicae (with similar morphology as in the main palatals) all weak and located inside the sinulus (the length and number (usually two to four) of these plicae is variable and not useful in species identification). There are usually two basal plicae (although one of them could be homologous with the infrapalatal plica), which are long but can occasionally be shorter. Surface of all barriers is finely granulated. Sinulus not strongly isolated from the rest of the aperture due to the low angular lamella and weak palatal tubercle. Umbilicus is narrow, measuring 1/8 of the shell width.

**Figure 90.**
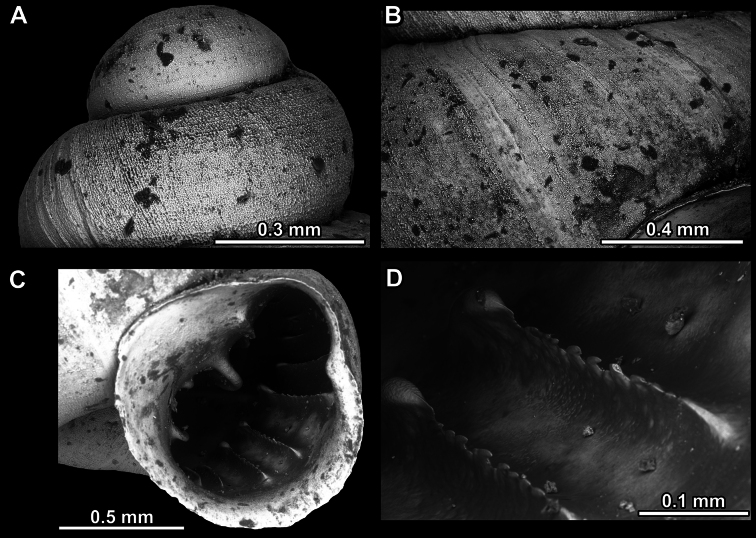
SEM imaging of *Bensonellaserrata* Gojšina, Hunyadi & Páll-Gergely, sp. nov., holotype (CUMZ 14443) **A** protoconch surface **B** teleoconch surface **C** enlarged apertural view **D** enlarged view of the palatal plicae.

##### Differential diagnosis.

This species can be separated from its congeners by the presence of equally strong palatal plicae with elevated inner and very low outer portions equipped with numerous spines.

##### Measurements

**(in mm, *n* = 5).**SH = 2.52–2.86; SW = 1.80–2.06; AH = 0.94–1.03; AW = 0.83–0.94.

##### Etymology.

This species is named for the serrated (saw-like) palatal plicae.

##### Distribution.

This species is known from two localities situated ca 18 km apart (straight-line distance).

#### ﻿ Bensonella spelaea

Taxon classificationAnimaliaStylommatophoraHypselostomatidae

Gojšina, Grego & Páll-Gergely sp. nov.

85FB2666-1444-51FF-A1AD-40E199238617

https://zoobank.org/260C97CC-545C-464D-B2F2-A8F66A014DE8

##### Type material.

***Holotype*. Myanmar** • 1 shell (SH: 3.3 mm, SW: 2.4 mm); Kayah State, Hpruso district, Maw Thi Do Village, entrance of Phruno river cave; 19°22.744'N, 97°2.570'E; 12 Feb. 2019; J. Grego leg.; CUMZ 14444. ***Paratypes*. Myanmar** • 7 shells; same data as for holotype; coll. JG • 1 shell; same data as for holotype; coll. HA.

##### Type locality.

Myanmar, Kayah State, Hpruso district, Maw Thi Do Village, entrance of Phruno river cave; 19°22.744'N, 97°2.570'E.

##### Diagnosis.

*Bensonella* species with 7–9 apertural barriers, most peculiar of these is the transversal plica (strong, high but with a concave frontal surface resembling a cave entrance).

##### Description.

Shell triangular, conical-ovoid, brownish, consisting of 5–5.5 convex, regularly increasing, rounded whorls separated by a deep suture. Boundary between the protoconch and the teleoconch is only clearly visible after SEM imaging because of the delicate sculpture and somehow weathered type series. Protoconch is spirally striated, probably ~ 1.75 whorls). Teleoconch of fine, pasty sculpture with dense but fine radial growth lines and without signs of spiral striation. Last whorl rounded, adnate to the penultimate. Aperture profile sinuated in its upper portion (concave) but convex in its middle and lower sections. Peristome expanded, especially at the parietal side where it leans on the penultimate whorl in form of a strong callus but not reflected. There is a moderate cervical crest located a short distance behind the peristome. Aperture equipped with relatively small number of barriers, seven to nine. Angular, parietal, upper palatal, transversal, palatal tubercle, columellar and infraparietal are always present, while basal and supracolumellar only sometimes. Parietal lamella heart-shaped in profile due to the sinuation located in its middle part (this makes the middle part of the lamella lower than outer and inner parts). Angular lamella also appears curvy, and it is curved to the palatal side. Upper palatal plica relatively weak and low. Transversal plica large (the largest in the aperture), high but with a strongly concave frontal surface (this gives the appearance of the transversal plica as an entrance to a cave). Basal very short and low when present. Columellar lamella very strong and thick (roughly the same as the parietal lamella). Supracolumellar lamella weak, dot like (sometimes absent). Infraparietal lamella present but much weaker than both the parietal and the columellar. Surface of all apertural barriers is granulated to almost spiniferous. Sinulus small and distinctly separated from the rest of the aperture due to the strong and closely positioned angular lamella and palatal tubercle. Umbilicus initially narrow and the widening at the last whorl, elongated, measuring ~ 1/6–1/7 of the shell width.

**Figure 91.**
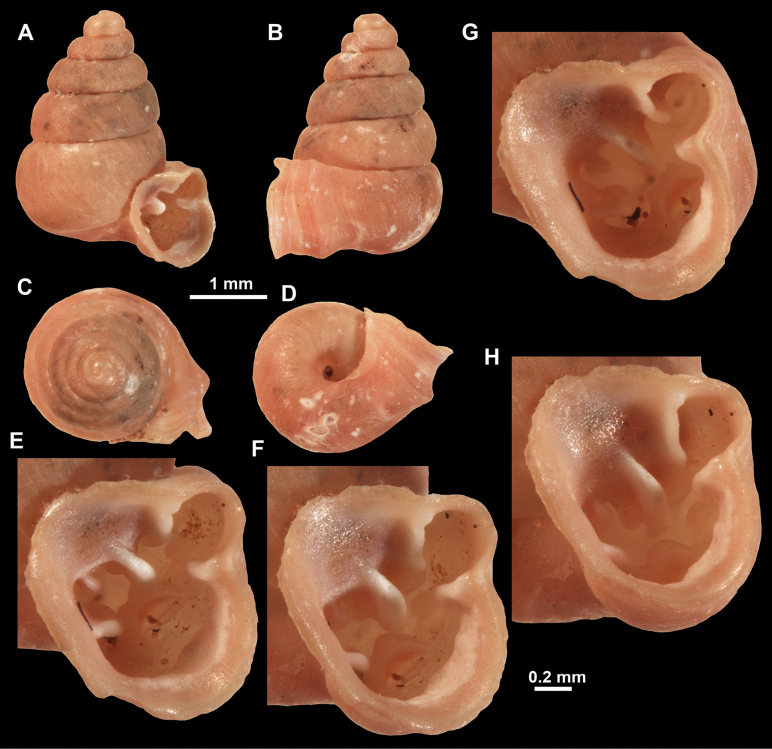
*Bensonellaspelaea* Gojšina, Grego & Páll-Gergely, sp. nov., holotype (CUMZ 14444) **A–D** shell **E–H** enlarged apertural views.

**Figure 92.**
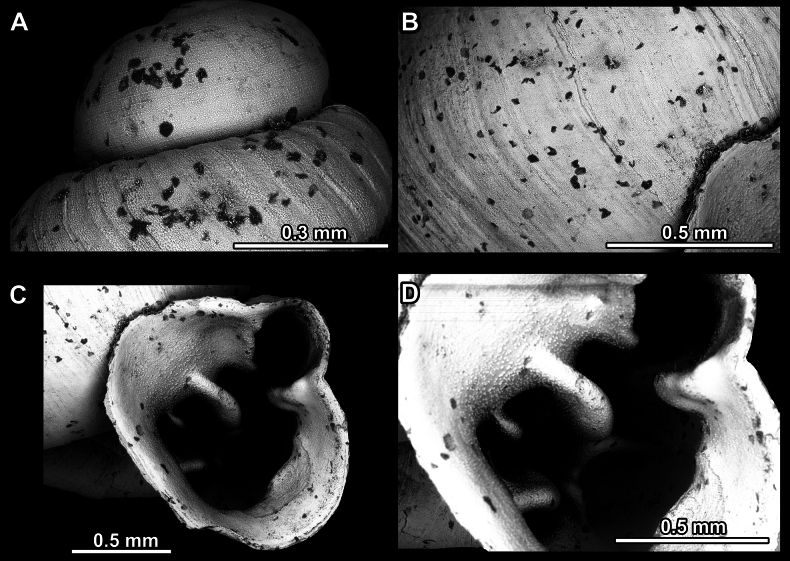
SEM imaging of *Bensonellaspelaea* Gojšina, Grego & Páll-Gergely, sp. nov., holotype (CUMZ 14444) **A** protoconch surface **B** teleoconch surface **C–D** enlarged apertural views.

##### Differential diagnosis.

This species is the largest one with a transversal plica. This plica shows the peculiar appearance with the strongly concave frontal surface resembling a cave entrance.

##### Measurements

**(in mm, *n* = 5).**SH = 2.48–3.38; SW = 1.98–2.4; AH = 1.08–1.30; AW = 0.95–1.12.

##### Etymology.

Named after the cave-like transversal plica.

##### Distribution.

This species is known only from the type locality.

#### ﻿ Bensonella spinosa

Taxon classificationAnimaliaStylommatophoraHypselostomatidae

Gojšina, Hunyadi & Páll-Gergely sp. nov.

86E9288D-3623-5017-999C-4A2A38DBE0DA

https://zoobank.org/8F54C76C-4461-4418-954D-F9536A580A9F

##### Type material.

***Holotype*. Myanmar** • 1 shell (SH: 2 mm, SW: 1.5 mm); Shan State, 5.7 km south-southwest from centre of Pinlaung, Wingabar Taung; 20°4.152'N, 96°46.232'E; 1510 m a.s.l.; 04 Oct. 2018; A. Hunyadi, K. Okubo & J. U. Otani leg.; CUMZ 14445. ***Paratypes*. Myanmar** • 14 shells; same data as for holotype; coll. HA.

##### Type locality.

Myanmar, Shan State, 5.7 km south-southwest from centre of Pinlaung, Wingabar Taung; 20°4.152'N, 96°46.232'E; 1510 m a.s.l.

##### Diagnosis.

*Bensonella* with triangular-conical, reddish-brown shell. Teleoconch fine, pasty, not spirally striated. All barriers and aperture surface around them very roughly spiniferous.

**Figure 93.**
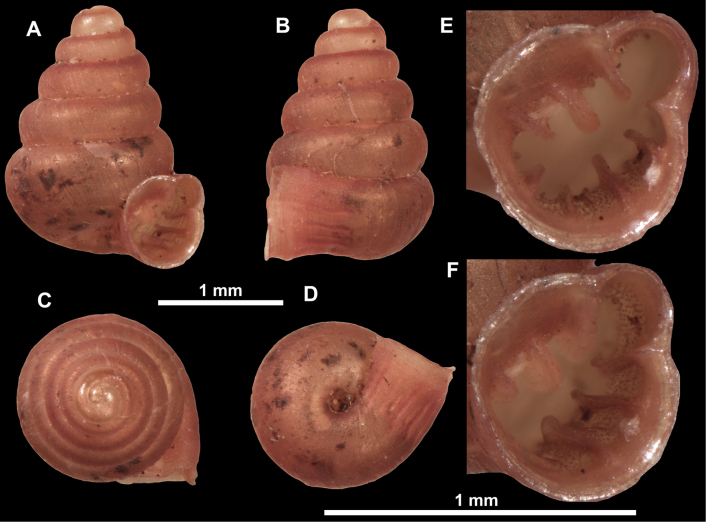
*Bensonellaspinosa* Gojšina, Hunyadi & Páll-Gergely, sp. nov., holotype (CUMZ 14445) **A–D** shell **E, F** enlarged apertural views.

##### Description.

Shell triangular, slightly conical-ovoid, reddish brown, consisting of 5–5.5 convex, rounded whorls separated by a deep suture. Protoconch of ~ 1.5 whorls, finely pitted, showing very weak spiralling pattern, lighter than the rest of the shell but with no clearly visible boundary with the teleoconch. Teleoconch finely dimpled (pasty) and finely radially striated but without spiral striae. Last whorl adnate to the penultimate and very slightly ascending near the aperture (~ 5–10 ° compared to the shell axis), making the aperture profile weakly opisthocline to the shell axis. Peristome of the same colour as the rest of the shell, expanded and not reflected. Aperture equipped with numerous barriers. Parietal lamella is the strongest in the aperture, high but not curved, directed towards the palatal wall. Angular lamella slightly lower than the parietal but otherwise very similar, continuous. There are three main palatal plicae (upper palatal, interpalatal and lower palatal) and one additional small interpalatal plica situated below the stronger interpalatal and a lower palatal. Upper palatal as strong as the main interpalatal. Lower palatal plica is the strongest. Infrapalatal plica weak. Basal plica 2 × stronger than the infrapalatal. All palatal plicae are higher at their inner parts and sloping towards their outer parts. There is a strong palatal tubercle sitting on the palatal lip of the peristome, in front of the upper palatal plica. Peristome is distinctly sinuated behind this tubercle. Columellar lamella ~ 2 × stronger than the basal and positioned obliquely, directed towards the parietal lamella. Subcolumellar similar to the infrapalatal. Supracolumellar lamella similar to the subcolumellar. Infraparietal lamella strong as the columellar. All apertural barriers are very strongly spiniferous as well as the surface of the aperture around them. Sinulus small and well separated from the rest of the aperture due to the strong angular lamella. Umbilicus very narrow, dot like.

**Figure 94.**
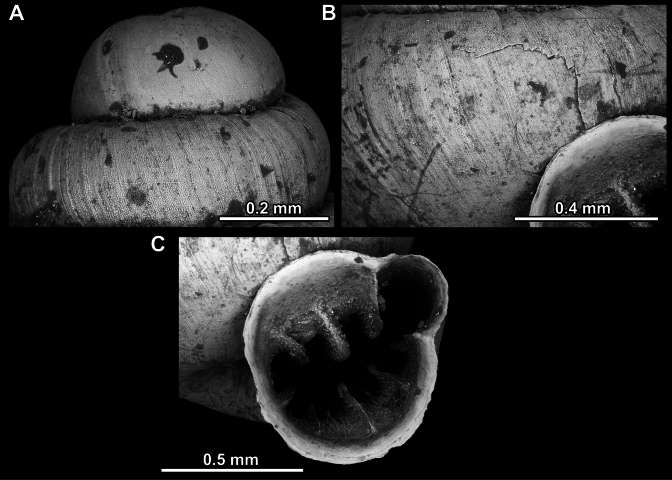
SEM imaging of *Bensonellaspinosa* Gojšina, Hunyadi & Páll-Gergely, sp. nov., holotype (CUMZ 14445) **A** protoconch surface **B** teleoconch surface **C** enlarged apertural view.

**Figure 95.**
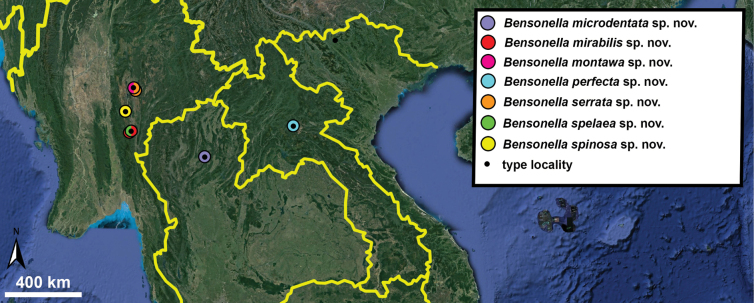
Distribution map of some species belonging to the *B.plicidens* group.

##### Differential diagnosis.

This species is most similar to *B.plicidens* from which it differs by the absence of spiral striation as well as spiniferous barriers and aperture surface (both smooth in *B.plicidens*). *Bensonellalakainguta*, *B.multihami* and *B.hooki* all have more numerous and hooked apertural barriers and they are not spiniferous.

##### Measurements

**(in mm, *n* = 5).**SH = 1.81–2.14; SW = 1.37–1.5; AH = 0.66–0.79; AW = 0.63–0.75.

##### Etymology.

The specific epithet is due to the numerous spines in the aperture.

##### Distribution.

This species is known only from the type locality.

#### ﻿ Bensonella taiyaiorum

Taxon classificationAnimaliaStylommatophoraHypselostomatidae

Tongkerd & Panha, 2024

E5B7E7D8-D8AE-5096-BD83-A826BFED1EC7

Bensonella taiyaiorum Tongkerd & Panha in Tongkerd et al. 2024: 174–176, figs 7B, 8, 13H.

##### Type material examined.

**Myanmar** • holotype; collector unknown; CUMZ 14380.

##### Type locality.

“Dragon Rock, Pindaya Township, Taunggyi District, Shan State, Myanmar (20°55'31.5"N, 96°39'01.2"E; 1300 m a.s.l.)”.

##### Differential diagnosis.

This species differs from *B.lophiodera* by the stronger apertural barriers, narrower umbilicus, and the lack of a sharp cervical crest. *Bensonelladracula* sp. nov. is smaller, has a second palatal tubercle and lacks the suprapalatal plica.

**Figure 96.**
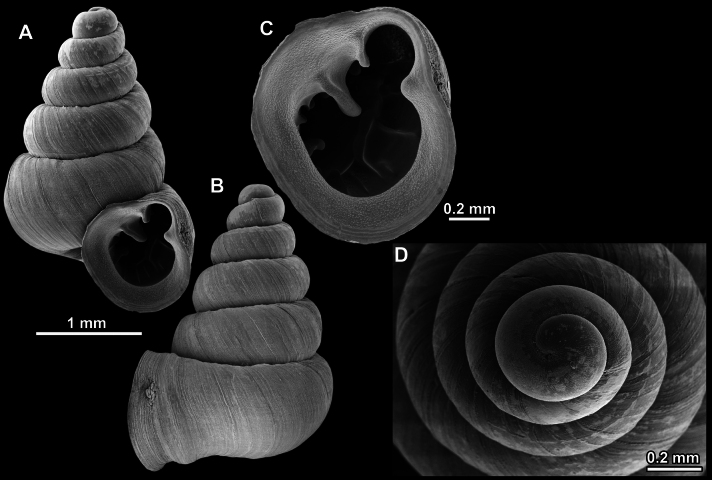
*Bensonellataiyaiorum*, holotype (CUMZ 14380) (from Tongkerd et al. 2024) **A–B** shell **C** enlarged apertural view **D** enlarged view of the protoconch.

**Figure 97.**
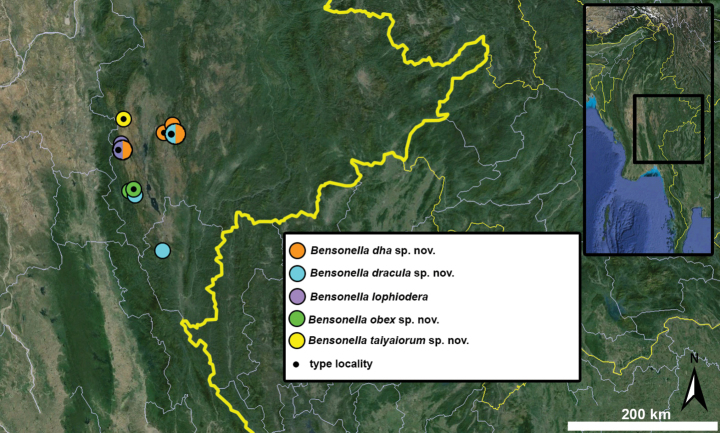
Distribution map of some *Bensonella* species from Myanmar.

##### Distribution.

This species is known only from the type locality.

#### ﻿ Bensonella tamphathai

Taxon classificationAnimaliaStylommatophoraHypselostomatidae

(Panha & J. B. Burch, 2000)

13D45606-1CD5-5703-8CD6-9312C6349E9E

Paraboysidia tamphathai Panha & Burch, 2000: 107, fig. 2.
Paraboysidia tamphathai — Panha and Burch 2008: 115, fig. 98.
Bensonella tamphathai — Dumrongrojwattana and Wongkamhaeng 2024: 324.

##### Material examined.

**Thailand** • 4 shells; Lampang Province, limestone ridge, Ban Pang La; 18°35′7″N, 99°50′32″E; 400 m a.s.l.; 14. May 1988; F.G. Thompson leg.; locality code FGT-4438; UF 346895 • 11 shells; Lampang Province, limestone knoll 1 km NE of Ban Pang La; 18°33′15″N, 99°52′11″E; 400 m a.s.l.; 14. May 1988; F.G. Thompson leg.; locality code FGT-4436; UF 346878 • 14 shells; same data as previous; locality code FGT-4435; UF 380388 • 3 shells; Lampang Province, limestone mtn. 3 km SE of Ban Mae Moa ledge on NW slope of mtn., leaf litter; 18°14′3″N, 99°42′49″E; 500 m a.s.l.; 15. May 1988; F.G. Thompson leg.; locality code FGT-4443; UF 346921 • 2 shells; Lampang Province, 6 km SW Mae Pak, limestone hill, 17°57′53″N, 98°51′7″E (provided coordinates point in Lamphun province); 600 m a.s.l.; 13 June 1987; F.G. Thompson leg.; locality code FGT-4328; UF 346579.

##### Type locality.

“Tam Pha Thai National Park, Lampang Province,18°36'20"N, 99°53'49"E, 490 meters elevation” (Thailand).

##### Differential diagnosis.

The most similar species is *B.paviei* which is however smaller and has less pronounced spiral sculpture. Apertural barriers are also more numerous than in *B.paviei*. *Bensonellapalatotridens* is not spirally striated. *Bensonellatamphathai* has a blunter conical shell (i.e., more barrel-shaped), narrower umbilicus and fewer basal plicae than *B.multidentata* sp. nov. *Bensonellamultidentata* sp. nov. is also much more densely spirally striated which makes it shell surface less shiny than in *B.tamphathai*. *Bensonellanitens* sp. nov. has a wider umbilicus, is more concave-conical and has a thin, sharp, and long projections of palatal plicae (proximally and distally). Also, the last whorl is *B.nitens* sp. nov. is much narrower than in *B.tamphathai* (i.e., it contributes less to the total shell height than in *B.tamphathai*). *Bensonellasericata* sp. nov. is much more densely spirally striated, lacks the basal plica and has weaker upper palatal plica. *Bensonellamicrodentata* sp. nov. has weaker apertural barriers which are roughly spiniferous.

**Figure 98.**
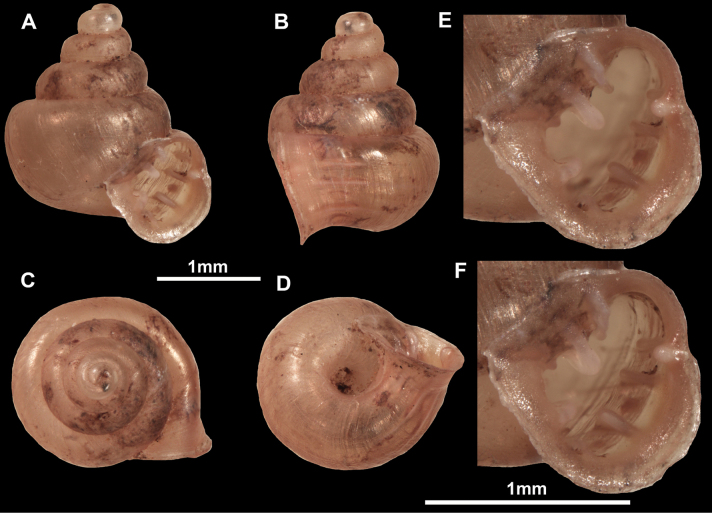
*Bensonellatamphathai* from Lampang province near Ban Pang La (UF 346895), Thailand **A–D** shell **E, F** enlarged apertural views.

**Figure 99.**
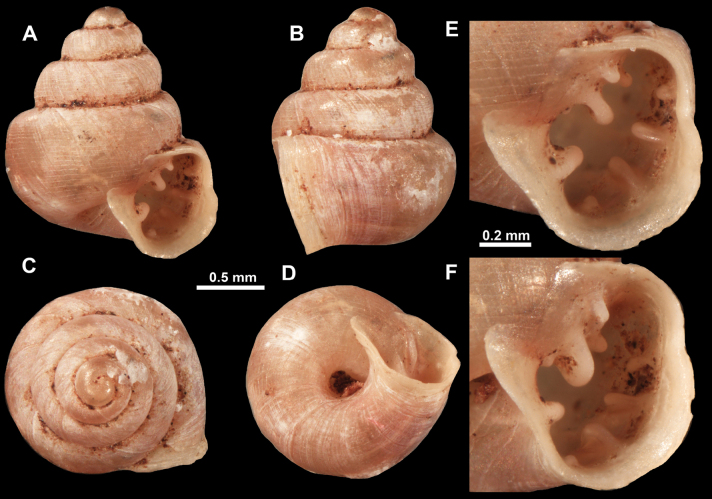
Bensonellacf.tamphathai from Lampang province near Ban Mae Moa (UF 346921), Thailand **A–D** shell **E, F** enlarged apertural views.

**Figure 100.**
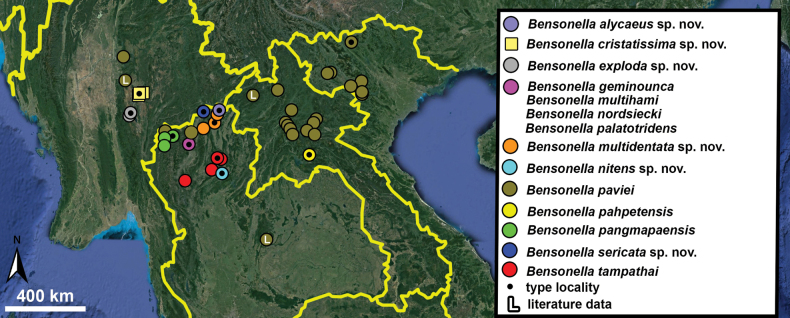
Distribution map of some species belonging to *B.plicidens* group.

##### Distribution.

This species is known from Lampang Province, Thailand.

#### ﻿2. Bensonella wangviangensis

Taxon classificationAnimaliaStylommatophoraHypselostomatidae

group

81B09EC7-FFAF-5287-BFE1-EC2B8F089434

##### Diagnosis.

This species group is characterised by angular lamella as strong as the parietal or even stronger, fully reaching the peristome edge, palatal tubercle not in its typical form but clearly represents a slightly discontinued part of the upper palatal plica (thus, it is of more lamella-like than tubercle-like appearance) and closely situated angular lamella and palatal tubercle which clearly separated the sinulus from the rest of the aperture.

##### Remarks.

Four species are assigned to this group and they inhabit northern Thailand (Loei Province) as well as adjacent territories in Laos (Vientiane and Khammouane provinces). Due to the same distribution and very similar overall shell morphology, these species are probably closely related.

#### ﻿ Bensonella cardiostoma

Taxon classificationAnimaliaStylommatophoraHypselostomatidae

Gojšina, Vermeulen & Páll-Gergely sp. nov.

1B07E1B3-0EC6-57AD-B2E3-370E1B034C1B

https://zoobank.org/44528248-CD8D-4171-86CF-E12B58A5741F

**Figure 101.**
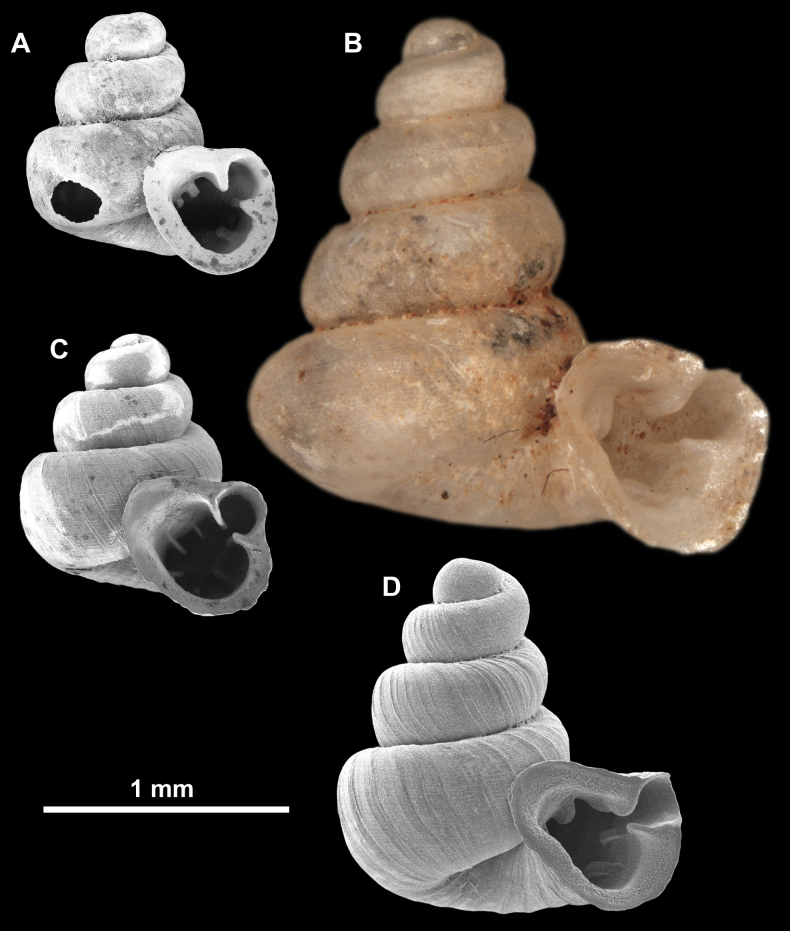
Synoptic view of species belonging to the *Bensonellawangviangensis* group **A***B.cardiostoma* sp. nov. **B***B.fracta* sp. nov. **C***B.mitochondria* sp. nov. **D***B.wangviangensis*.

**Figure 102.**
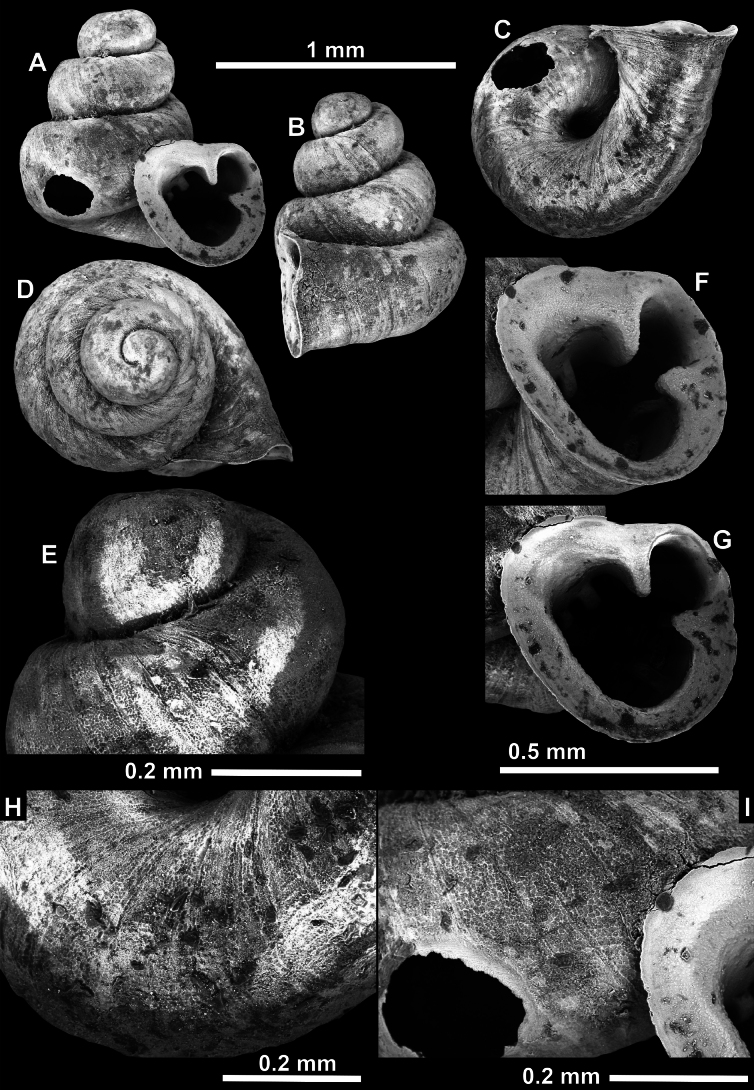
*Bensonellacardiostoma* Gojšina, Vermeulen & Páll-Gergely, sp. nov., holotype (CUMZ 14447) **A–D** shell **E** enlarged view of the protoconch **F, G** enlarged apertural views **H, I** enlarged views of the surface of the last whorl.

Figs 101A , 102 , 107

##### Type material.

***Holotype*. Laos** • 1 shell (SH: 1.12 mm; SW: 1.17 mm); Vientiane Province, Vang Vieng District, Nakay Village, steep forested limestone hill; 19°08.731'N, 102°22.481'E; 323 m a.s.l.; 24 Oct. 2006; A. Schuiteman leg.; locality code JJV 15174; CUMZ 14447.

##### Type locality.

Laos, Vientiane Province, Vang Vieng District, Nakay Village, steep forested limestone hill, 19°08.731'N, 102°22.481'E, 323 m a.s.l.

##### Diagnosis.

*Bensonella* species with obscure, weak spiral striae, asymmetric heart-shaped aperture, an angular lamella, and a palatal tubercle on the peristome and six barriers (upper palatal, lower palatal, basal, columellar, infraparietal, parietal) situated deeper inside the aperture.

##### Description.

Shell colourless (white), conical-ovoid with strongly widened last whorl. Whorls 3.5, nearly rounded, slightly shouldered. Protoconch consisting of 1.25 whorls, pitted, showing spiralling pattern, although no clear spiral striae are visible. Teleoconch sculpture roughly pitted. Last whorl adnate to the penultimate, very slightly ascending (~ 10 ° compared to the shell axis), thus making the apertural profile opisthocline to the shell axis. Aperture asymmetric heart-shaped, the parietal part being the shield-like extended peristome. Angular lamella and palatal tubercle situated on the peristome, both are pointed, directed towards each other. Other barriers, altogether 6, are lamella-like, elongated, low. Upper palatal plica sits behind palatal tubercle, lower palatal situated between the basal and upper palatal plica. Columella lamella situated halfway between basal and infraparietal lamella, infraparietal and parietal situated close to each other. Peristome strongly expanded, not reflected. Surface of all apertural barriers is finely granulated. Sinulus relatively narrow and clearly separated from the rest of the aperture due to the strong and closely positioned angular lamella and palatal tubercle. Umbilicus very narrow, shows only last whorl. Last quarter whorl sigmoid.

##### Differential diagnosis.

See under *B.mitochondria* sp. nov. and *B.wangviangensis*.

##### Measurements

**(in mm, *n* = 1).**SH = 1.12; SW = 1.17; AH = 0.56; AW = 0.58.

##### Etymology.

Named after the heart-shaped aperture.

##### Distribution.

This species is known only from the type locality.

#### ﻿ Bensonella fracta

Taxon classificationAnimaliaStylommatophoraHypselostomatidae

Gojšina, Hunyadi & Páll-Gergely sp. nov.

E4B317B9-D829-5265-900D-725F9B28567D

https://zoobank.org/DC60AC55-09A0-4B54-9D74-A3162A27F079

**Figure 103.**
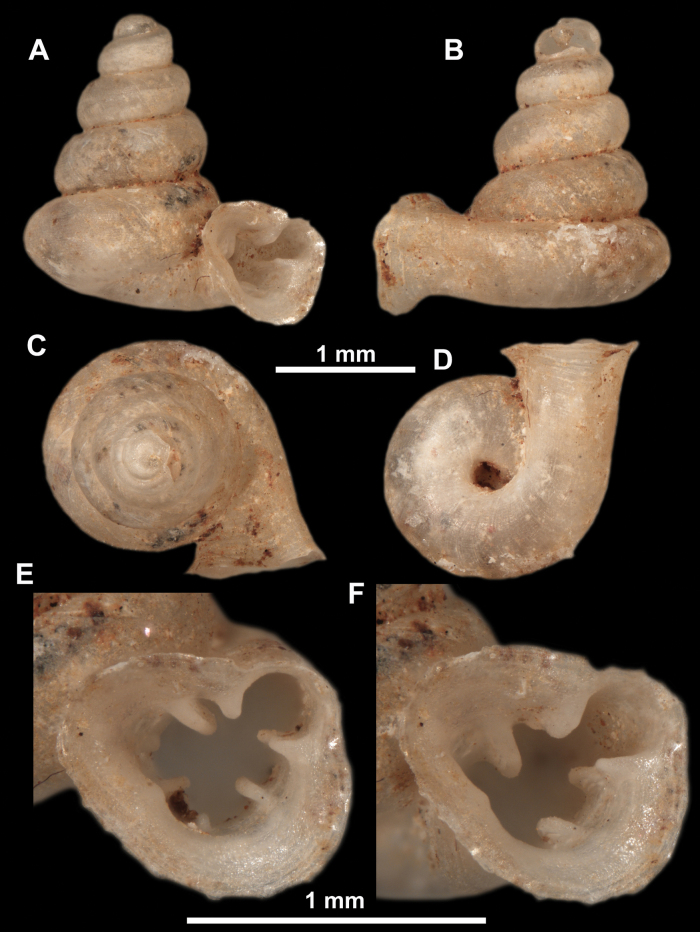
*Bensonellafracta* Gojšina, Hunyadi & Páll-Gergely, sp. nov., holotype (CUMZ 14448) **A–D** shell **E, F** enlarged apertural views.

**Figure 104.**
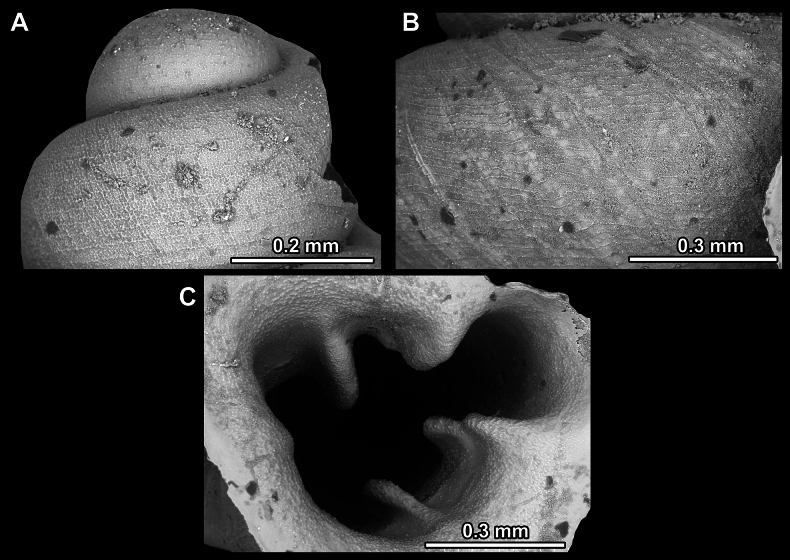
SEM imaging of *Bensonellafracta* Gojšina, Hunyadi & Páll-Gergely, sp. nov., holotype (CUMZ 14448) **A** protoconch surface **B** teleoconch surface **C** enlarged apertural view.

Figs 101B , 103 , 104 , 107

##### Type material.

***Holotype*. Thailand** • 1 shell (SH: 2.08 mm; SW: 2.09 mm); Loei Province, Nong Hin district, 20.3 km southwest from centre of Nong Hin towards Pha Wai, left side of road no. 3029; 17°2.471'N, 101°43.655'E; 705 m a.s.l.; 28 Feb. 2023; A. Hunyadi & J.U. Otani leg.; CUMZ 14448.

***Paratypes*. Thailand** • 5 shells; same data as for holotype; coll. HA.

##### Type locality.

Thailand, Loei Province, Nong Hin district, 20.3 km southwest from centre of Nong Hin, Pha Wai; left side of road no. 3029; 17°2.471'N, 101°43.655'E; 705 m a.s.l.

##### Diagnosis.

*Bensonella* species with spirally striated teleoconch and strongly convex whorls separated by a deep suture. Last whorl narrow, slightly detached, rounded. Apertural barriers usually 7. Upper palatal plica bipartite, consisting of anterior and posterior bulge.

##### Description.

Shell conical, light yellowish-brownish, opaque. It is consisting of ~ 5 regularly increasing, strongly convex whorls separated by a deeply impressed suture. Protoconch finely pitted, showing a spiralling pattern (which is stronger terminally) and consisting of ~ 1.5 whorls, coloured as the rest of the shell. Teleoconch with sculpture consisting of fine, raised, and innumerable spiral striae which are occasionally crossed by much weaker radial growth lines (rarely present as strong white streaks). The sculpture of the teleoconch gets more prominent as the whorls are increasing, thus being most noticeable on the last two whorls. Last whorl relatively narrow (low), slightly detached from the penultimate, and slightly ascending near the aperture (~ 5–10 ° compared to the shell axis). It is completely rounded and strongly convex. Peristome the same colour as the rest of the shell, strongly expanded with finely pitted surface, not reflected. Aperture equipped with five main barriers (angular, parietal, upper palatal, lower palatal, and columellar). Angular lamella short and thick, directed towards the lower palatal plica. Parietal lamella longer than the angular, leaned towards the upper palatal plica. Upper palatal plica usually bipartite, consisting of anterior and posterior bulge. Anterior bulge is closer to the peristome, smaller and lower. Posterior bulge is longer, higher, and stronger, positioned behind the anterior (deeper in the aperture). Origin of the anterior bulge is not known but it may be homologous with the palatal tubercle usually found in many typical *Bensonella* species. Columellar lamella developed to the similar extent as the palatal plicae. In front of the columellar lamella, there is sometimes a small swelling on the peristome. Apart from these main barriers, there is usually an additional smaller basal plica and one even smaller knob-like lamella in the columello-parietal embayment. Only the angular lamella and anterior bulge of the upper palatal plica are reaching t peristome. Surface of all apertural barriers is finely granulated. Sinulus small, well separated from the rest of the shell due to the closely situated angular lamella and upper palatal plica. Umbilicus moderately wide (1/5–1/6 of the shell width), showing only the penultimate whorl. Umbilical groove absent.

##### Differential diagnosis.

*Bensonellafracta* sp. nov. is much larger than all other representatives of *Bensonellawangviangensis* group. See also under *B.wangviangensis*.

##### Measurements

**(in mm, *n* = 4).** SW1 = 2.09–2.18; SW2 = 1.31–1.48; SH = 1.9–2.08; AH = 0.81–1.1; AW = 0.89–1.23.

##### Etymology.

The first examined specimen of this species had a crack on the apical whorls, which gave the specific epithet *fracta*.

##### Distribution.

This species is known only from the type locality.

##### Remarks.

This species is not a typical representative of this group since the angular lamella is quite small and not almost enclosing the sinulus. However, it is placed here because of the high morphological similarity with mostly *B.wangviangensis*. A tubercle-like swelling in the columellar-parietal embayment was found to be missing in one specimen. Otherwise, this species does not seem to be much variable regarding apertural barriers.

#### ﻿ Bensonella mitochondria

Taxon classificationAnimaliaStylommatophoraHypselostomatidae

Gojšina, Vermeulen & Páll-Gergely sp. nov.

01F35930-07FC-5417-ACE0-35B7545C9ABF

https://zoobank.org/4CFB8D44-B70A-4C97-A4FB-8E750B5405AB

**Figure 105.**
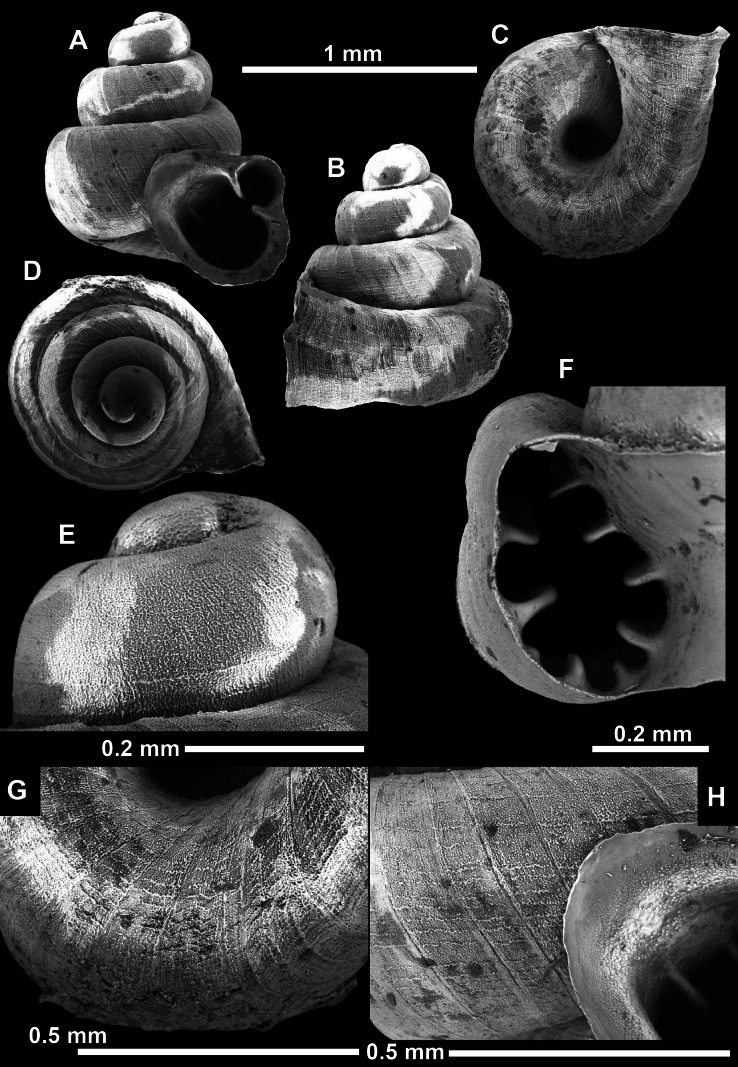
*Bensonellamitochondria* Gojšina, Vermeulen & Páll-Gergely, sp. nov. **A–D** shell, holotype (CUMZ 14449) **E** enlarged protoconch view, holotype (CUMZ 14449) **F** enlarged apertural view (from the back, last whorl broken), paratype (coll. JJV) **G, H** enlarged views of the surface of the last whorl, holotype (CUMZ 14449).

Figs 103C , 105 , 107

##### Type material.

***Holotype*. Laos** • 1 shell (SH: 1.21 mm; SW: 1.27 mm); Khammouane Province, Thakhek Distr., Ban Nakok, base of forested limestone hill; 17°25.993'N, 105°07.880'E; 222 m a.s.l.; 14 Nov. 2006; A. Schuiteman leg.; locality code JJV 15157; CUMZ 14449. ***Paratypes*. Laos** • 9 shells; same data as for holotype; coll. JJV.

##### Additional material examined.

**Laos** • 2 shells (juveniles, not paratypes); same data as for holotype; coll. JJV.

##### Type locality.

Laos, Khammouane Province, Thakhek Distr., Ban Nakok, base of forested limestone hill; 17°25.993'N, 105°07.880'E; 222 m a.s.l.

##### Diagnosis.

A colourless *Bensonella* species with shouldered last whorl and spirally striated teleoconch and nearly heart-shaped aperture. Apertural barriers numerous and almost all equally strong, umbilicus moderately wide.

##### Description.

Shell conical-ovoid, colourless. Whorls 3.5, initial whorls rounded, last two strongly shouldered, separated by a deep suture. Protoconch consisting of ~ 1 whorl, very finely pitted, with signs of indistinct and widely spaced spiralling pattern. Teleoconch with coarse spiral striae which are crossed by stronger, coarse, not numerous, and almost regularly spaced radial growth lines. Approximately 25 widely spaced spiral striae present on the last whorl in standard apertural view. Last whorl adnate to the penultimate, slightly ascending near the aperture (~ 10–15 ° compared to the shell axis). Peristome continuous, strong, and expanded, especially at the parietal side where it leans on the penultimate whorl. Aperture nearly heart-shaped, equipped with a strong angular lamella, which is curved and almost merges with the prominent palatal tubercle, thus nearly enclosing the small sinulus (which makes it strongly separated from the rest of the aperture). Angular lamella reaches the peristome. There are eight to nine other barriers situated deep in the aperture and not reaching the peristome. Additional two lamellae at the parietal side (equally strong parietal and infraparietal), one columellar lamella, one subcolumellar, one basal plica, two palatal plicae (sometimes with additional, weak infrapalatal plica) and a palatal tubercle. Upper and lower palatal plicae strong, the former is situated slightly above the palatal tubercle. Palatal tubercle quite strong and elevated. Basal plica weaker than the palatals but stronger than the subcolumellar lamella. Columellar lamella slightly stronger than them but weaker than upper and lower palatal plica. Surface of all apertural barriers is finely granulated. Umbilicus moderately wide, measuring ~ 1/5 of the shell width and showing only the penultimate whorl.

##### Differential diagnosis.

This species differs from *B.cardiostoma* sp. nov. most clearly by the spirally striated shell and a wider umbilicus.

##### Measurements

**(in mm, *n* = 5).**SH = 1.21–1.33; SW = 1.21–1.37; AH = 0.57–0.62; AW = 0.58–0.67.

##### Etymology.

The appearance of the apertural barriers when the last whorl is broken behind them resembles the morphology of a mitochondria (Fig. 105F), hence the specific epithet which is to be used as a noun in apposition.

##### Distribution.

This species in known only from the type locality.

#### ﻿ Bensonella wangviangensis

Taxon classificationAnimaliaStylommatophoraHypselostomatidae

(Panha & Tongkerd, 2003)

CBE9A7CC-1E83-58C7-BB32-97846454363A

**Figure 106.**
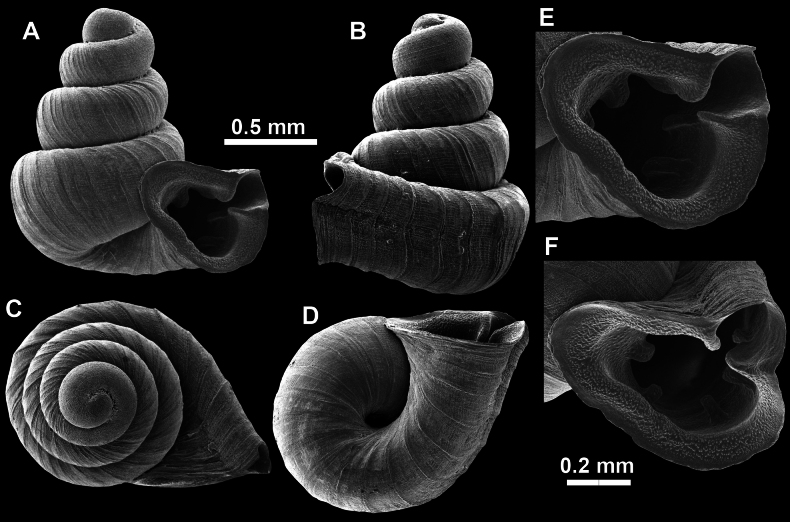
*Bensonellawangviangensis*, paratype (CUMZ ver. 089) **A–D** shell **E, F** enlarged apertural views.

**Figure 107.**
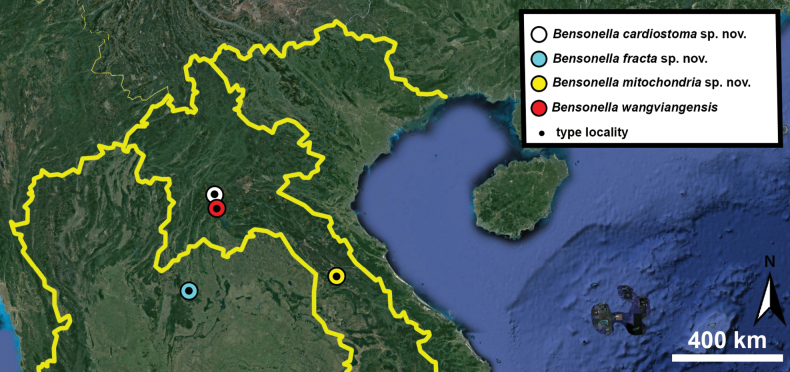
Distribution map of species belonging to the *Bensonellawangviangensis* group.

Figs 103D , 106 , 107

Paraboysidia wangviangensis Panha & Tongkerd in Panha et al. 2003: 123–128, figs 2–3.
Paraboysidia wangviangensis — Inkhavilay et al. 2019: 62.

##### Type material examined.

**Laos** • 1 paratype; from the type locality; 1998; S. Panha leg.; CUMZ ver. 089.

##### Type locality.

“Tam Chang Cave, Wangviang, Laos, 18°54′47″N, 102°26′33″E, 140 meters elevation…” (Laos).

##### Differential diagnosis.

This species differs from *B.cardiostoma* sp. nov. and *B.mitochondria* sp. nov. by the more elongated shell, larger size as well as depressed and more elongated aperture. Additionally, *B.cardiostoma* sp. nov. is not spirally striated. Apart from the clearly smaller size, this species can further be separated from *B.fracta* sp. nov. by the shouldered last whorl, which is rounded in *B.fracta* sp. nov.

##### Distribution.

This species is known only from the type locality.

##### Remarks.

We examined the types of this species and could add several notes to the original description. This species is originally described as having six apertural barriers (angular, parietal, infraparietal, two upper palatals and a lower palatal). From the Fig. 106, it can be clearly seen that there are eight barriers, the ones already described and additionally one interpalatal and a columellar lamella. The “second upper palatal plica” from the original description is in the position of usually mentioned palatal tubercle (typical for *Bensonella*), although it does not look like a tubercle but more like a proper lamella. Nevertheless, we treat them homologous.

#### ﻿ Bensonella

Taxon classificationAnimaliaStylommatophoraHypselostomatidae

sp. 1

25F2B0EE-4652-56B4-A402-93225CF307F8

##### Material examined.

**Thailand** • 1 shell; Phangnga Province, 32 km NE Khok Kloi, Wat Tham Suwan Khuha; 8°27'N, 98°27'E; 27 Mar. 1988; 20 m a.s.l.; K. Auffenberg leg.; locality code KA-0601; UF 345752.

##### Remarks.

Only a single, weathered shell was in this sample which made the precise idenitifcation impossible. Based on the morphology of apertural barriers, they were possibly hooked.

#### ﻿ Bensonella

Taxon classificationAnimaliaStylommatophoraHypselostomatidae

sp. 2

2011362C-F184-5D21-BCE9-062C3F325157

##### Material examined.

**Thailand** • 1 shell; Chiang Rai prov., Tham Tumluang cave, 7 km S, 2 k W of Mae Sai. Around caves, leaf litter; 20°22.1667'N, 99°51.4667'E; 08. May 1988; F. G. Thompson leg.; locality code FGT-4419; UF 380223.

##### Remarks.

A single, damaged specimen was in this sample which made its identification impossible.

#### ﻿ Bensonella

Taxon classificationAnimaliaStylommatophoraHypselostomatidae

sp. 3

4F4D5B05-735E-5878-8058-202718A703A4

Fig. 108

##### Material examined.

**Laos** • Luang Namtha Province, Luang Namtha ca 43.8 km southwest – Vieng Phou Kha, Phou Lan, right side of the road 100 m; 20°44.5278'N, 101°11.1011'E; 770 m a.s.l.; 08 Oct. 2019; A. Hunyadi, K. Okubo, J. U. Otani leg., coll. HA.

##### Remarks.

Only a single, rather weathered, and partly damaged specimen of *Bensonella* which is the most similar to *B.lakainguta* and related species (*B.hooki*, *B.montawa* sp. nov., *B.multihami*) could be examined from Laos. Most of the aperture is obstructed by the dirt that could not be cleaned which is why majority of the barriers were invisible. The identity of this species remains unresolved until more specimens are collected.

**Figure 108.**
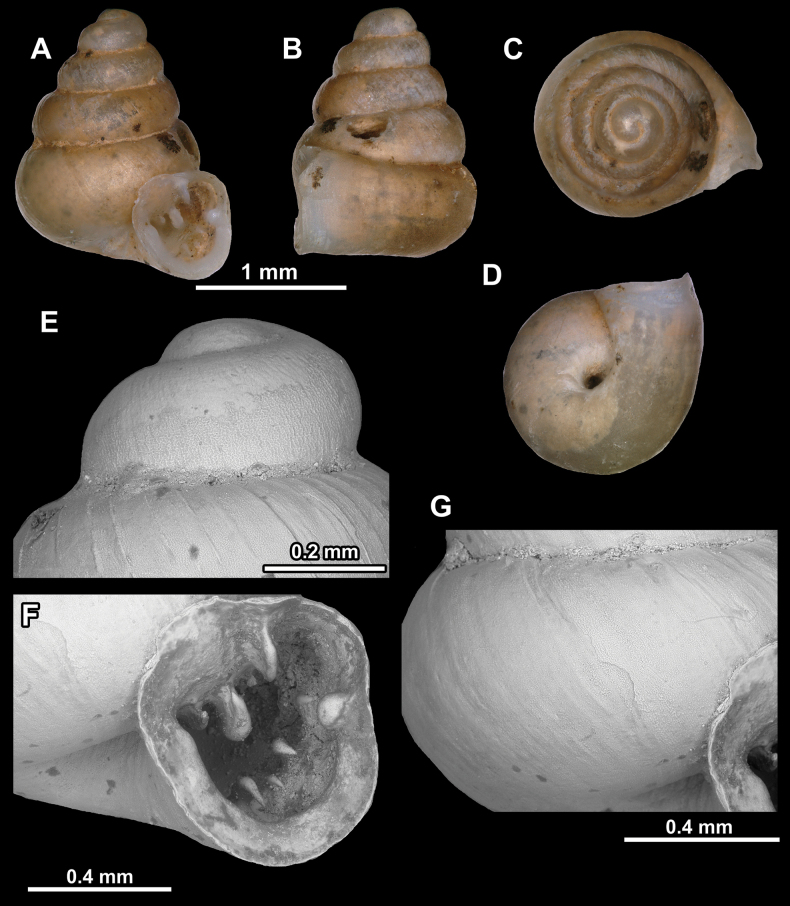
*Bensonella* sp. 3 from Luang Namtha Province, Laos **A–D** shell **E** enlarged view of the protoconch **F** enlarged view of the aperture **G** view of the last whorl surface sculpture.

#### ﻿﻿Genus Boysidia

Taxon classificationAnimaliaStylommatophoraHypselostomatidae

Ancey, 1881

E1CB45B8-27A6-5B21-BF4D-022023DB33F5

Pupa (Boysidia) Ancey, 1881: 373.

##### Diagnosis.

The genus *Boysidia* remains unrevised. This is urgently needed because many species are misunderstood due to poor original descriptions/drawings or the uncertain status of type specimens. The genus currently includes the following species: *Boysidiachiangmaiensis* Panha & J. B. Burch, 2002, *B.conspicua* (Möllendorff, 1885), *B.dilamellaris* D.-N. Chen, Y.-H. Liu & W.-X. Wu, 1995, *B.dorsata* (Ancey, 1881), *B.gracilis* F. Haas, 1937, *B.guiyangensis* T.-C. Luo, D.-N. Chen, G.-Q. Zhang, La. Zhang, Li Zhang & T.-W. Li, 1998, *B.hangchowensis* (Pilsbry & Y. Hirase, 1908), *B.houaphanica* Inkhavilay & Sutcharit, 2024, *B.huangguoshuensis* T.-C. Luo, D.-N. Chen & G.-Q. Zhang, 2000, *B.hunana* (Gredler, 1881), *B.jinpingensis* M. Tian, B. Fao & Y.-X. Chen, 2015, *B.nanjiangensis* Z.-L. Zhang, W.-H. Zhang & T.-C. Luo, 2011, *B.orientalis* B. Rensch, 1935, *B.phatangensis* Dumrongrojwattana & Assawawattagee, 2018, *B.ringens* van Benthem Jutting, 1950; *B.robusta* Bavay & Dautzenberg, 1912, *B.shilinensis* D.-N. Chen, M. Wu & G.-Q. Zhang, 1999, *B.strophostoma* (Möllendorff, 1885), *B.taibaiensis* D.-N. Chen, M. Wu & G.-Q. Zhang, 1999, *B.tamtouriana* Pokryszko & Auffenberg, 2009, *B.tholos* Panha & J. B. Burch, 2002, *B.tongguanensis* D.-N. Chen & W.-H. Zhang, 2002, *B.xianfengensis* W.-H. Zhang, D.-N. Chen & W.-C. Zhou, 2014, *B.xiaoguanensis* W.-H. Zhang, D.-N. Chen & W.-C. Zhou, 2014, *B.xishanensis* D.-N. Chen, M. Wu & G.-Q. Zhang, 1999, *B.xiuwenensis* W.-H. Zhang, T.-C. Luo & W.-C. Zhou, 2010.

The species listed below are excluded from the genus *Bensonella* and transferred to *Boysidia* due to the following traits (not shared among all these species): i) apertural dentition more similar to *Boysidia* (columellar lamella very strong and long, absence of three barriers on the parietal side); ii) surface sculpture similar to *Boysidia* (radial growth lines, no spiral striae); iii) larger shell sizes (frequently over 4 mm), which is more common in *Boysidia* representatives.

#### ﻿ Boysidia gittenbergeri

Taxon classificationAnimaliaStylommatophoraHypselostomatidae

(Maassen, 2008) comb. nov.

ACA17536-8885-544E-8295-97B068E1C844

Paraboysidia gittenbergeri Maassen, 2008: 237–239, figs 5, 6.
Paraboysidia gittenbergeri — Inkhavilay et al. 2016: 215–216, figs 1, 2A–C, 4A, tables 1, 2; Inkhavilay et al. 2019: 61, fig. 27A.

##### Material examined.

None.

##### Type locality.

“NW Laos, province Luang Namtha, district Vieng Phouka, Oung (= Khama word for cave) Pra Ngiene, 500 m SE of Ban (= village) Phou Lek, at the entrance of the cave”.

##### Remarks.

We have excluded this species from *Bensonella* and classified it in the genus *Boysidia* due to the combination of the following traits: i) large shell size (nearly 6 mm); ii) absence of the palatal tubercle typical for *Bensonella*; iii) shell shape more similar to some *Boysidia* species (e.g., *B.chiangmaiensis*, *B.tholos*, *B.lamothei*).

#### ﻿ Boysidia hupeana

Taxon classificationAnimaliaStylommatophoraHypselostomatidae

(Gredler, 1901)

F896FB6E-8C4E-59BA-880C-F22CD4EB712C

Figs 109

Hypselostoma hupeanum Gredler, 1901: 151.
Boysidia (Paraboysidia) hupeana — Pilsbry 1917: 206; Haas 1937: 8–9.
Paraboysidia hupeana — van Benthem Jutting 1950: 38; Zilch 1984: 165.
Paraboysidia kitteli Maassen, 1999: 123, figs 5–7. syn. nov.
Boysidia (Paraboysidia) hubeina [sic] — Chen and Zhang 2002: 692.
Boysidia (Boysidia) gongyaoshanensis Yang, Zhang & Chen, 2012: 550 (English description), 550–551 (Chinese description), figs 1–3. syn. nov.

##### Type material examined.

**Indonesia** • 1 paratypes of *P.kitteli*; from the type locality; 17 Aug. 1993; ex. coll. Hemmen, Wiesbaden; coll. PGB • 1 paratype of *P.kitteli*; from the type locality; 16 June 1990; ex. coll. Hemmen, Wiesbaden; coll. PGB • 4 paratypes of *P.kitteli*; from the type locality; Aug. 1993; ex. coll. W.J.M. Maassen; coll. PGB.

##### Additional material examined.

**China** • 1 shell; Badung, Hubei, coll. Moellendorff; SMF 10775.

##### Type localities.

“Südwest Hupé”, China (*B.hupeana*); “Gongyao Hill, Xingyi County (25°N, 104°08'E), Guizhou province, China” (*B.gongyaoshanensis*); “N. Sumatra, Karo Highlands, Kuta Buluh, 40 km N of Brastagi, near the entrance of the cave Liangdehar in leaf litter at the foot of limestone rocks” (*B.kitteli*).

##### Remarks.

Boysidia (Boysidia) gongyaoshanensis, described from Gongyao Hill, Xingyi County (25°N, 104°08'E), Guizhou province, China, is herein treated a junior synonym of *B.hupeana* since no morphological differences were noticed. Also, no differences in shell morphology were noticed between *B.kitteli* and *B.hupeana* even though they are described from localities which are ca 3200 km from each other. Even though the distance is enormous, they are treated conspecific due to the lack of conchological differences. Both new synonyms were not compared to *B.hupeana* in their respective original descriptions (Maassen 1999; Yang et al. 2012).

**Figure 109.**
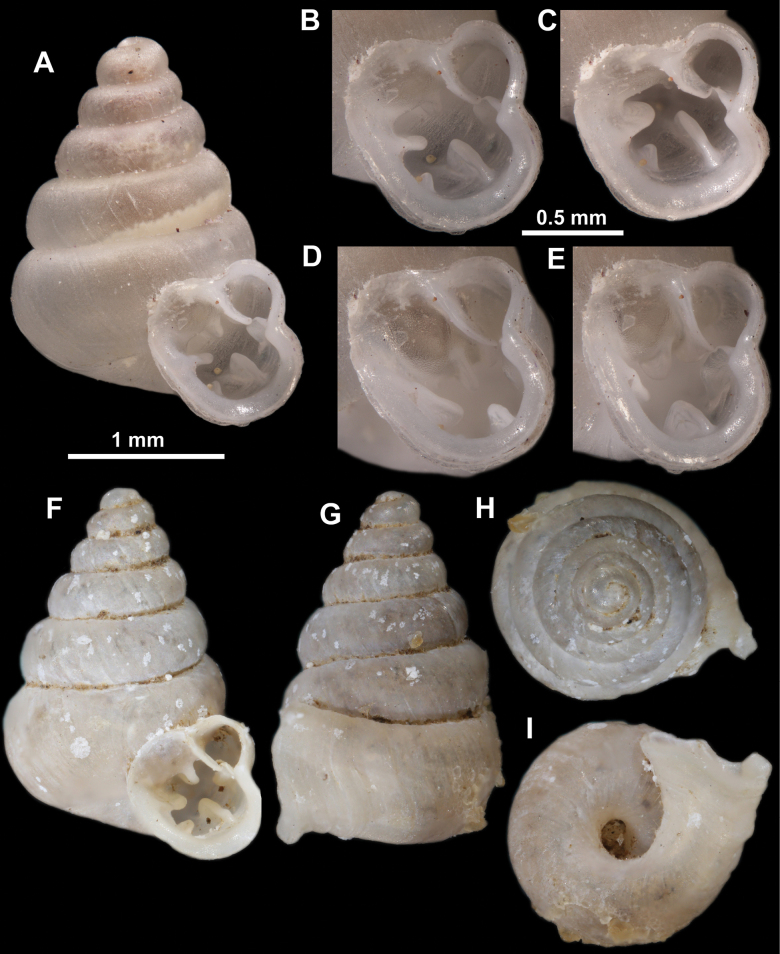
*Boysidiahupeana***A–E** paratype of *B.kitteli* (coll. PGB) F–I specimen from Badung, Hubei (SMF 10775) **A, F–I** shell **B–E** enlarged apertural views.

#### ﻿ Boysidia lamothei

Taxon classificationAnimaliaStylommatophoraHypselostomatidae

Bavay & Dautzenberg, 1912

63963AC6-652A-566A-A731-262CF2E57043

Boysidia lamothei Bavay & Dautzenberg, 1912: 21, pl. 3, figs 7–9.
Boysidia (Paraboysidia) lamothei — Pilsbry 1917: 202–203, pl. 35, figs 3–6; Schileyko 2011: 2.
Paraboysidia lamothei — van Benthem Jutting 1950: 39.

##### Type material examined.

**Vietnam** • syntype; MNHN-IM-2000-35160.

##### Type locality.

“Ban-Lao”, Vietnam.

##### Remarks.

This species is treated as a member of *Boysidia* due to the shell shape being more similar to some other congeners such as *B.robusta* and *B.jinpingensis* and a strong and oblique columellar lamella which extends to the expanding peristome.

#### ﻿ Boysidia paralella

Taxon classificationAnimaliaStylommatophoraHypselostomatidae

(Inkhavilay & Panha, 2016) comb. nov.

26135924-C761-5D7E-A806-0B6583719D01

Paraboysidia paralella Inkhavilay & Panha in Inkhavilay et al. 2016: 220, figs 1, 3A–C, 4C.
Paraboysidia paralella — Inkhavilay et al. 2019: 62, fig. 27B.

##### Material examined.

**Laos** • holotype; CUMZ 7059.

##### Type locality.

“Limestone wall near the entrance of Kao Rao Cave, Vieng Phouka District, Luang Namtha Province, Laos (20°43'30.1"N, 101°9'4.3"E), 732 m amsl”.

##### Remarks.

This species is assigned to *Boysidia* because of similar shell shape, surface sculpture and apertural barrier arrangement (especially strong columellar lamella) to some *Boysidia* species such as *B.gittenbergeri*, and *B.hupeana*.

#### ﻿ Boysidia salwiniana

Taxon classificationAnimaliaStylommatophoraHypselostomatidae

(Theobald, 1870)

E7FD78A3-18A8-5E2F-AE11-487531096348

Pupasalwiniana Theobald, 1870: 400.
Pupasalwiniana — Hanley and Theobald 1874: pl. 100, fig. 9; Sowerby 1876: pl. 16, fig. 150; Nevill 1878: 23.
*Pupasalwineana* [sic] — Godwin-Austen 1888: 244.
Boysidia (Paraboysidia) salwiniana — Pilsbry 1917: 206–208, pl. 33, fig. 11.
Paraboysidia salwiniana — van Benthem Jutting 1950: 40.
Bensonella salwiniana — Tongkerd et al. 2024: 167–171, figs 4, 5, 6A, 13F.

##### Material examined.

**Myanmar** • 1 shell; Burma NHMUK 1912.4.16.66.

##### Type locality.

“Shan States”, Myanmar.

##### Remarks.

Tongkerd et al. (2024) provided the first description of the genitalia and radula of this species. They have also pointed out that the type specimens could not be located.

#### ﻿ Boysidia shuitianbaensis

Taxon classificationAnimaliaStylommatophoraHypselostomatidae

T.-C. Luo, D.-N. Chen, G.-Q. Zhang, La. Zhang, Li Zhang & T.-W. Li, 1998

4007E7BA-4304-56AE-9150-78EC0F36563A

Boysidia (Bensonella) shuitianbaensis Luo et al., 1998: 2 (Chinese description), 3–4 (English description), fig. 2.

##### Material examined.

None.

##### Type locality.

“Collected from Shuitianba town (26°7'N, 106°8'E), Guiyang City, Guizhou Province, China”.

##### Remarks.

According to the original description, this species should be assigned to *Boysidia* due to the presence of only four barriers and a merged angulo-parietal lamella.

### ﻿Additional *Boysidia* material examined

#### ﻿ Boysidia ringens

Taxon classificationAnimaliaStylommatophoraHypselostomatidae

van Benthem Jutting, 1950

78541F9F-33E6-59D6-8EAC-2028999B26AD

Fig. 110

Boysidia ringens van Benthem Jutting, 1950: 11–12, fig. 3.

##### Type material examined.

**Malaysia** • 23 paratypes; from the type locality; M. W. F. Tweedie leg.; RMNH Moll.137127.

##### Additional material examined.

**Malaysia** • 7 shells; Kedah, Gunung Keriang (N of Alor Setar), Oct. 1998, Hemmen, leg. Wiesbaden ex. coll.; coll. PGB • 1 shell; Kedah, Gunung Keriang, 7.5 km NW of Alor Setar; ex. coll. Maassen; coll. PGB.

**Figure 110.**
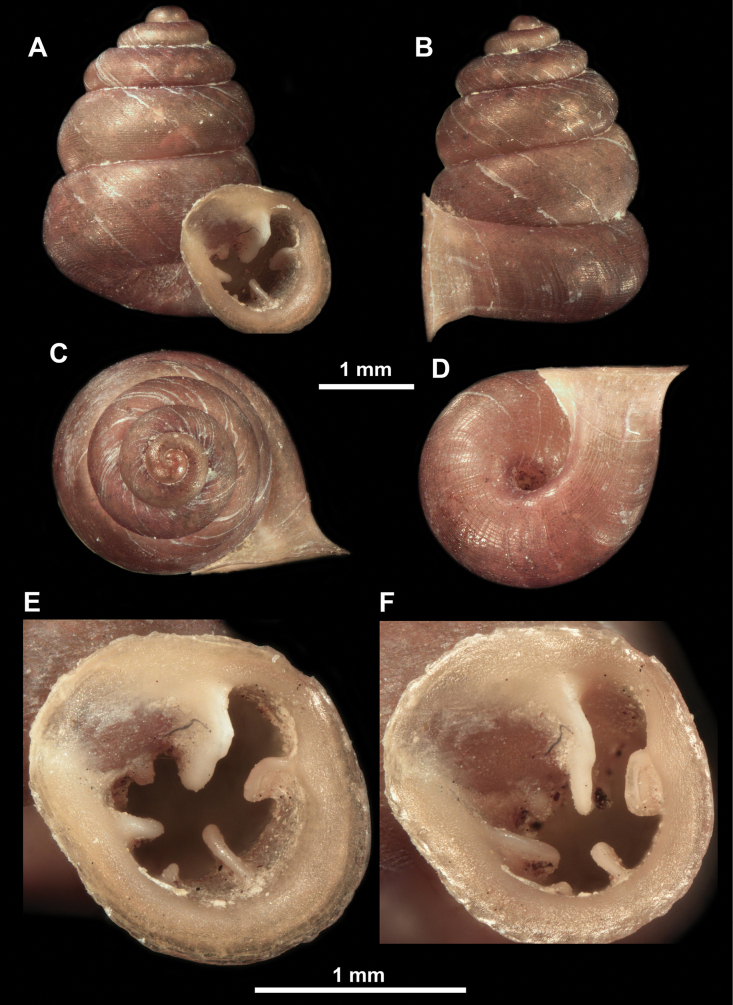
*Boysidiaringens*, paratype (RMNH Moll.137127) **A–D** shell **E, F** enlarged apertural views.

##### Type locality.

“Mount Keriang, Kedah”, Malaysia.

##### Cross-diagnosis.

This species is not similar to any other congener.

##### Distribution.

This species is known only from the type locality.

#### ﻿﻿Genus Hypselostoma

Taxon classificationAnimaliaStylommatophoraHypselostomatidae

Benson, 1856

4B7B972D-41A7-5753-9C4B-D3938597E898

Hypselostoma Benson, 1856b: 342 (replacement name forTanystoma Benson, 1856, non Motschulsky 1845, Carabidae, Coleoptera).
Gyliotrachela Tomlin, 1930: 24. (replacement name forGyliauchen Pilsbry 1917 non Nicoll 1915, Platyhelminthes, Trematoda) syn. nov.
Antroapiculus Panha & Burch, 2002a: 144–148. syn. nov.

##### Diagnosis.

This genus is subdivided into four species groups: i) *H.bensonianum* group ii) *H.hungerfordianum* group; iii) *H.terae* group and iv) *H.tubiferum* group. This subdivision is based on the shell surface sculpture (coupled with apertural barrier morphology and geographic distribution in *H.terae* group) which is raised spirally striated in *H.bensonianum* group, granulated (sandpaper-like) in *H.hungerfordianum* group and fine, pasty in *H.terae* and *H.tubiferum* group. Most representatives share the depressed-conical or conical shell shape with last whorl adnate to or detached from the penultimate.

##### Remarks.

*Hypselostomaaenigma* sp. nov. is provisionally placed in this genus due to the specific apertural barrier arrangement. *Hypselostomatorta* sp. nov. is also not a typical representative since it has non-raised spiral striae which are probably not homologous to the raised ones as usual for the genus.

Genera *Gyliotrachela* and *Antroapiculus* are both treated as junior synonyms of *Hypselostoma* due to the following facts: i) *Antroapiculus* is described based on a unique shell (flat with strongly downwards descending aperture) and a single lamella on the parietal side (parietal lamella). This however does not seem to warrant separation of a new genus since the flat shell (*H.tridentatum*, *H.khaowongkot*, *H.utongense*) and strongly descending aperture (*H.srakeoense*, *H.chaunosalpinx*, *H.fungus* sp. nov., *H.transitans*, *H.torticollis*) are shared with other congeners. Furthermore, apertural barrier arrangement is also similar to *H.torticollis*, *H.fungus* sp. nov., *H.depressum* which indicates that the combination of characters in *H.pendulum* could just be an autapomorphic state which could be changed rapidly during the evolution; ii) *Gyliotrachela* is treated distinct from *Hypselostoma* solely based on the separated parietal and angular lamella on the parietal side (Pilsbry 1917). Since we have found several examples of species with almost exactly the same shell morphology, but with different apertural barrier arrangements (Fig. 262), we find it less likely that they belong to different genera and find this criterium insufficient.

###### ﻿1. *Hypselostomabensonianum* group

**Diagnosis.***Hypselostomabensonianum* group is characterised by raised and usually widely spaced spiral striation (in form of threads).

**Remarks.** A total of 46 species belong to this group. This group has a wide distribution across Southeast Asia as well as in Australia where only one species can be found. In Southeast Asia, this group spans from central and eastern Myanmar to Taiwan as well as southwards all the way to Indonesia. Northernmost localities on mainland Southeast Asia are known from Luang Prabang Province in Laos.

#### ﻿ Hypselostoma adela

Taxon classificationAnimaliaStylommatophoraHypselostomatidae

(F.G. Thompson & Upatham, 1997) comb. nov.

3057154D-0548-5D0A-9080-72205C62B448

**Figure 111.**
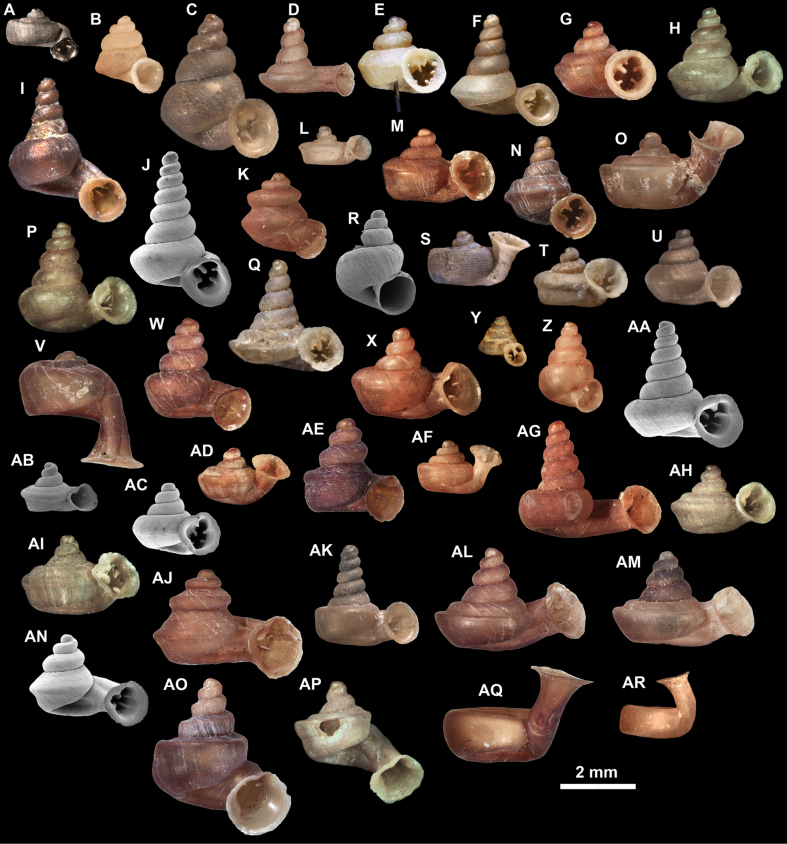
Synoptic view of species belonging to the *Hypselostomabensonianum* group **A***H.adela***B***H.aenigma* sp. nov. **C***H.annamiticum***D***H.aquila* sp. nov. **E***H.australe***F***H.benetuitum***G***H.bensonianum***H***H.cambodjense***I***H.chaunosalpinx***J***H.chedi***K***H.circumcarinatum* sp. nov. **L***H.cucumense***M***H.cf.cultura***N***H.depressum***O***H.diarmaidi***P***H.dilatatum***Q***H.discobasis***R***H.edentatum***S***H.erawan***T***H.everetti***U***H.fruhstorferi***V***H.fungus* sp. nov. **W***H.geckophilum* sp. nov. **X***H.holimanae***Y***H.insularum***Z***H.iunior* sp. nov. **AA***H.khaochakan***AB***H.khaochongpran***AC***H.khaowongense***AD***H.kohrin***AE***H.platybasis* sp. nov. **AF***H.populare* sp. nov. **AG***H.rupestre***AH***H.salpinx***AI***H.saxicola***AJ***H.sculpturatum* sp. nov. **AK***H.smokon***AL***H.sorormajor* sp. nov. **AM***H.sororminor* sp. nov. **AN***H.taehwani***AO***H.torta* sp. nov. **AP***H.torticollis***A**Q *H.tridentatum***AR***H.utongense*.

**Figure 112.**
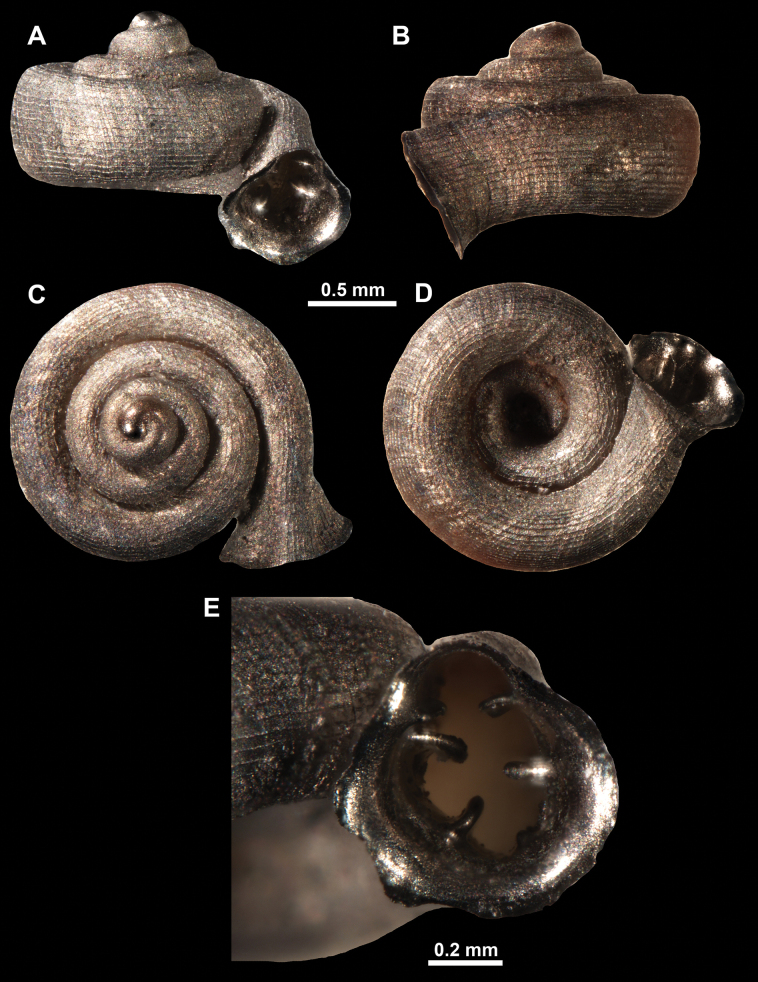
*Hypselostomaadela*, holotype (UF 00113508) **A–D** shell **E** enlarged apertural view. The specimen is coated for the purpose of SEM imaging, which is why the original colouration is not retained.

**Figure 113.**
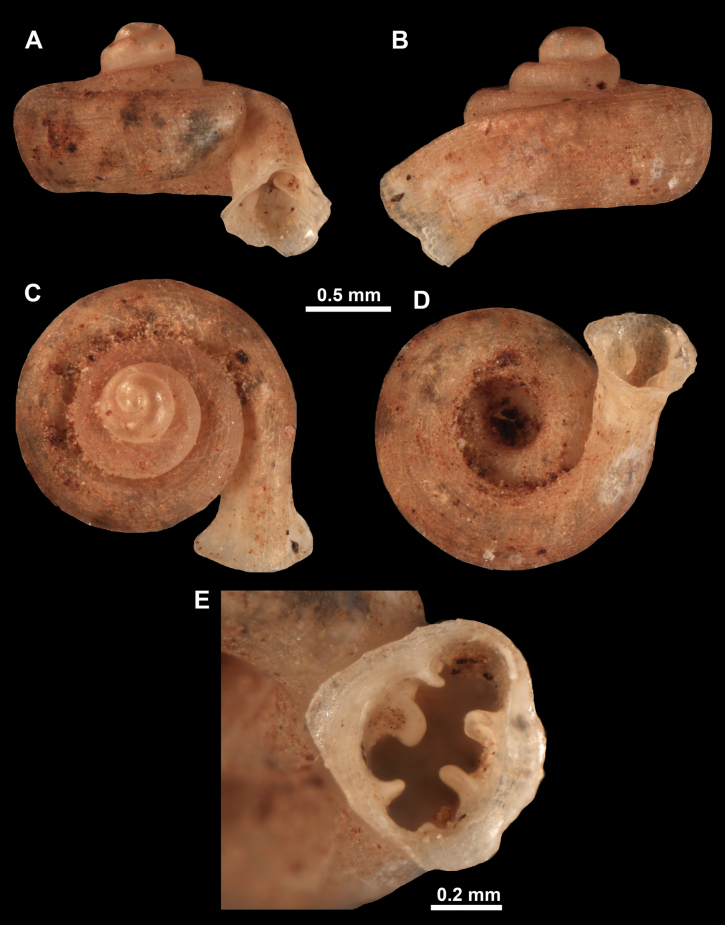
*Hypselostomaadela*, paratype (UF 00113509) **A–D** shell **E, F** enlarged apertural views.

Figs 111A , 112 , 113 , 154

Gyliotrachela adela Thompson & Upatham, 1997: 234–235, figs 44–47.
Gyliotrachela adela — Tongkerd et al. 2004: 143, fig. 2; Panha and Burch 2008: 65–66, fig. 57; Dumrongrojwattana and Wongkamhaeng 2024: 323, fig. 8.

##### Type material examined.

**Thailand** • holotype; 03 June 1987; F. G. Thompson leg.; UF 00113508; 4 paratypes; from the type locality; 03 June 1987; F. G. Thompson leg.; UF 00113509.

##### Additional material examined.

**Thailand-South** • 16 shells; Suratthani Province, 28.4 km E Surat Thani, 19 km S Hwy. 401, dry, scrubby evergreen forest, leaf litter, rocks, boulders; 9.15°N, 99.6°E; 40 m a.s.l.; 19 Apr. 1988; K. Auffenberg leg.; locality code KA-0675; UF 591322 • 15 shells; Suratthani Province, limestone outcrop along Hwy. 401, 2.7 km W junc Hwys. 4142 & 401; 9°10'N, 99°40'E; 90 m a.s.l.; 19 Apr. 1988; K. Auffenberg leg.; locality code KA-0673; UF 591323 • 2 shells; Suratthani Province, 15.3 km NE Ban Na San off road to Tha Rom Yen National Park; 8°53'N, 99°26'E; 40 m a.s.l.; 20 Apr. 1988; K. Auffenberg leg.; locality code KA-0678; UF 345389 • 5 shells; Suratthani Province, 13.8 km SE Don Sak, 5 km SW Hwy. 4142; 9°18'N, 99°43'E; 50 m a.s.l.; 14 Apr. 1988; K. Auffenberg leg.; locality code KA-0670; UF 345284 • 1 shell; Suratthani Province, 1 km NE of Na San, 100 m limestone mountain; 8.812°N, 99.382°E; 20 June 1987; F.G. Thompson leg.; locality code FGT-4309; UF 529617 • 35 shells; Suratthani Province, Hwy. 401, 2.7 km W junction Hwy. 4142 and 401, evergreen forest on rocky hillside, below cliff, base of cliff; 9°10'N, 99°40'E; 90 m a.s.l.; 19 Apr. 1988; K. Auffenberg leg.; locality code KA-0674; UF 345331 • 3 shells; Suratthani Province, 4.5 km S Don Sak, E side of Hwy. 4142, evergreen forest, limestone outcrop, base of cliff; 9°18'N, 99°42'E; 40 m a.s.l.; 18 Apr. 1988; K. Auffenberg leg.; locality code KA-0672; UF 345302 • 5 shells; Suratthani Province, 15.3 km NE Ban Na San off road to Thi Rom Yen National Park; 8°53'N, 99°26'E; 40 m a.s.l.; 20 Apr. 1988; K. Auffenberg leg.; locality code KA-0678; UF 591320 • 12 shells; Suratthani Province, 13.8 km SE Don Sak, 5 km SW Hwy. 4142; 9°18'N, 99°43'E; 50 m a.s.l.; 17 Apr. 1988; K. Auffenberg leg.; locality code KA-0669; UF 345277 • 9 shells; Suratthani Province, 15.3 km NE Ban Na San off road to Tha Rom Yen National Park; 8°53'N, 99°26'E; 40 m a.s.l.; 20 Apr. 1988; K. Auffenberg leg.; locality code KA-0679; UF 345397 • 16 shells; Suratthani Province, 28.4 km E Surat Thani, 1.9 km S Hwy. 401, dry, scrubby evergreen forest, leaf litter, rocks, boulders; 9.15°N, 99.6°E; 40 m a.s.l.; 19 Apr. 1988; K. Auffenberg leg.; locality code KA-0675; UF 436269 • 2 shells; Suratthani Province, limestone hill (with monastery) left side off rd. #4009 (~ 11 km N of Waing Sa); 08°41.590'N, 099°22.689'E; ex. coll. Hemmen, Wiesbaden; coll. PGB • 8 shells; Nakhon Si Thammarat Province, 15.6 km SE Don Sak, 1 km SW Hwy. 4142; 9°17'N, 99°46'E; 50 m a.s.l.; 15 Apr. 1988; K. Auffenberg leg.; locality code KA-0667; UF 345253 • 16 shells; Nakhon Sri Thammarat Province, 15.6 km SE Don Sak, 1 km SW Hwy. 4142; 9°17'N, 99°46'E; 50 m a.s.l.; 17 Apr. 1988; K. Auffenberg leg.; locality code KA-0668; UF 345258 • 2 shells; Nakhon Sri Thammarat Province, limestone hill right off road #4142 ca km 38.2 (road Khanom-Don Sak); Mar. 2000; ex. coll. Hemmen, Wiesbaden; coll. PGB • 4 shells; Krabi Province, Phi Phi islands, Phi Phi Don island, climbing rock above the western end of the beach in Ton Sai Bay, at the base of limestone rocks in the forest; 7°44.063'N, 98°45.947'E; 50 m a.s.l.; Mar. 2010; A. Reischütz leg; coll. REI • 1 shell; Phattalung Province, Khao Ok Thalu, rock wall; 07°37.506'N, 100°05.330'E; 19 Feb. 2015; A. Hunyadi leg.; coll. HA • 1 shell; Phatthalung Province, Khao Pu-Khao Ya Nat. Parl, Si Banphot district, ca 6 km NNW of Taphaen, 7°40.738'N, 99°50.822'E; 24 Feb. 2023; A. Hunyadi, K. Okubo & J.U. Otani leg.; coll. HA • 1 shell; Songkhla Province, 31.3 km NW Hat Yai, 1.2 km W of Hwy. 43; 7°10'N, 100°16'E; 80 m a.s.l.; 08 Apr. 1988; K. Auffenberg leg.; locality code KA-0636B; UF 344945. **Malaysia** • 3 shells; Pahang, Bandar Pusat Jengka, west 15 km, Hutan Lipur Gunung Senyum; 03°41.862'N, 102°25.980'E; 85 m a.s.l.; 23 Jan. 2013; A. Hunyadi leg.; coll. HA.

##### Type locality.

“Thailand, Surat Thani Province, box canyon in limestone range 6 km south of Na San; 100 m altitude (08°39.6'N, 99°23.9'E)”.

##### Differential diagnosis.

This species is not similar to any other congener and is unique due to the combination of the following characters: small size, yellowish shell, spirally striated teleoconch with descending detached last whorl and aperture equipped with several equally developed barriers (without smaller ones). See under *H.cucumense*.

##### Distribution.

This species in known from several localities in Thailand and one in Peninsular Malaysia. Our record from Malaysia is the first record of this species in the country.

##### Remarks.

Apertural barriers were constant in number in all the specimens examined. We have examined the material from northern Thailand hosted in UF with the following data: 1 shell; Mae Hong Song Province, Tham Lod Cave, 1.5 km SE Ban Tham Lod Village, 6 km N Sappong on Road 1095; 19°34'N, 98°9'E; 720 m a.s.l.; 19 Mar. 1988; K. Auffenberg leg.; locality code KA-0576; UF 591321; 7 shells; Mae Hong Song Province, Tham Lod Cave, 1.5 km SE Ban Tham Lod Village, 6 km N Sappong on Road 1095; 19°34'N, 98°9'E; 720 m a.s.l.; 19 Mar. 1988; K. Auffenberg leg.; locality code KA-0674; UF 345523. Since these localities are quite far from the actual distribution area of this species, we believe that these samples were mislabelled and that this species actually does not occur in Mae Hong Son Province.

#### ﻿ Hypselostoma aenigma

Taxon classificationAnimaliaStylommatophoraHypselostomatidae

Gojšina, Grego & Páll-Gergely sp. nov.

A7F1DE4E-D361-5EDB-A8AA-9BE749373388

https://zoobank.org/B0C84B3D-5A3B-4370-B49D-A1EC626CC0EE

**Figure 114.**
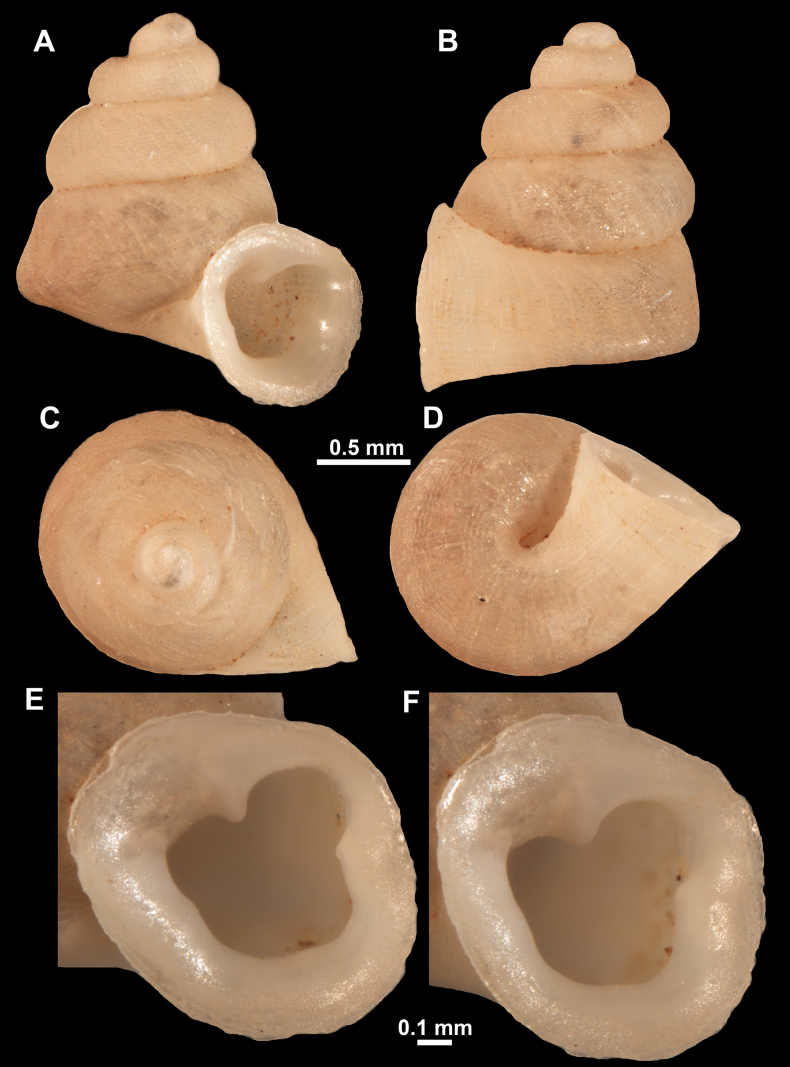
*Hypselostomaaenigma* Gojšina, Grego & Páll-Gergely, sp. nov., holotype (CUMZ 14452) **A–D** shell **E, F** enlarged apertural views.

**Figure 115.**
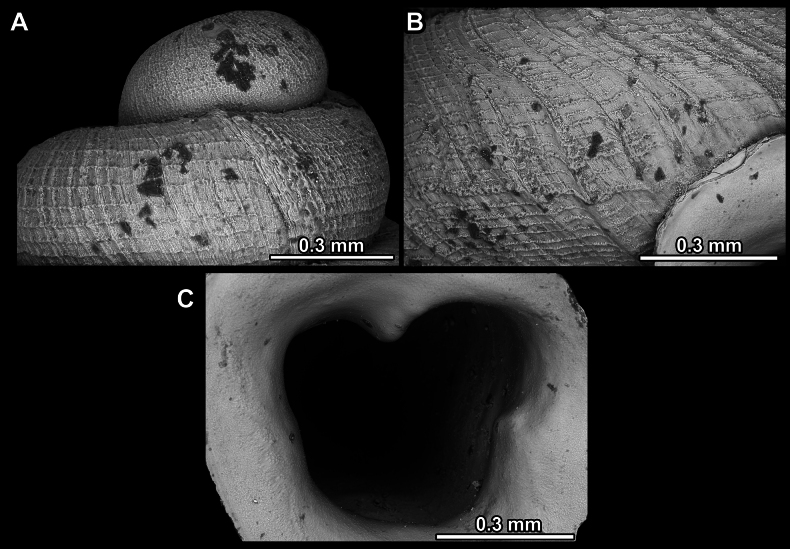
SEM imaging of *Hypselostomaaenigma* Gojšina, Grego & Páll-Gergely, sp. nov., holotype (CUMZ 14452) **A** protoconch surface **B** teleoconch surface **C** enlarged apertural view.

Figs 111B , 114 , 115 , 158

##### Type material.

***Holotype*. Myanmar** • 1 shell (SH: 2.1 mm; SW: 1.7 mm); Kayin State, Kamarmaung, Phon Tho Village, Pan Do Mi Mountain spring cave at W foot of the mountain; 17°19.3575'N, 97°43.4842'E 19. May 2019; J. Grego leg; CUMZ 14452.

##### Type locality.

Myanmar, Kayin State, Kamarmaung, Phon Tho Village, Pan Do Mi Mountain spring cave at W foot of the mountain; 17°19.3575'N, 97°43.4842'E.

##### Diagnosis.

*Hypselostoma* species with conical-ovoid shell, spirally striated whorls, and the last whorl with a keel below the centre of the periphery. Apertural barriers three but the parietal lamella is the only tooth-like barrier. Palatal plica and columellar lamella are present as swellings on the peristome.

##### Description.

Shell conical-ovoid, pale yellowish, consisting of nearly 4.5 whorls separated by a deep suture. All whorls, except the last one, regularly rounded and convex. Last whorl with a blunt keel below the centre of the periphery and weakly concave to almost flat whorl outline both above and below the keel. Protoconch of same colour as the rest of the shell, finely pitted showing spiralling pattern which continues as more distinct spiral striae terminally. It consists of ~ 1.75 whorls. Teleoconch coarsely sculptured with irregularly spaced radial growth lines but also with dense spiral striae. Spacing between the two spiral striae equals the width of two or three striae. Last whorl adnate to the penultimate and very slightly ascending (~ 7 ° compared to the shell axis), making the apertural profile weakly opisthocline to the shell axis. Peristome white, thick, and expanded (especially on the parietal side) but not reflected. Apertural barriers largely reduced and of very unusual appearance for any hypselostomatid. Parietal lamella is the only „tooth-like“ barrier in the aperture but it is very low and short. Palatal plica and columellar lamella not present in their typical forms but rather as very weak and indistinct swellings on the peristome, visible only in the frontal view. These swellings are probably homologous to the two mentioned barriers. There are no other barriers in the aperture. Surface of all apertural barriers is very finely granulated (almost smooth). Sinulus wide due to the weak parietal lamella and palatal plica. Umbilicus extremely narrow, dot like and slightly elongated. Umbilical groove absent.

##### Differential diagnosis.

This species is not similar to any other congener or species within other genera due to the unique appearance of apertural barriers.

##### Measurements

**(in mm, *n* = 1)**: SH = 2.1; SW = 1.7; AH = 0.97; AW = 0.93.

##### Etymology.

The specific epithet *aenigma* comes from a Latin word for a riddle (which refers to the unique appearance of the shell and unclear relationship with other congeners) and is to be used as a noun in apposition.

##### Distribution.

This species is known only from its type locality.

#### ﻿ Hypselostoma annamiticum

Taxon classificationAnimaliaStylommatophoraHypselostomatidae

Möllendorff, 1900

9BB9CA10-F75D-5259-9436-1730BFCC2306

**Figure 116.**
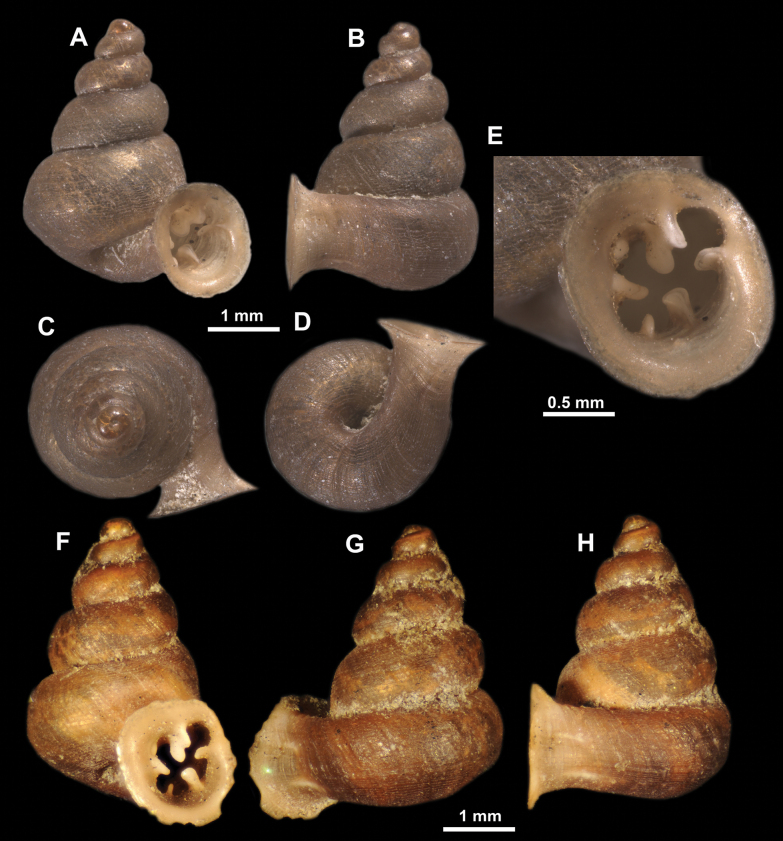
*Hypselostomaannamiticum***A–E** holotype of *H.annamiticumannamiticum* (SMF 4871) **F–H** paralectotype of *H.annamiticumaltius* (ANSP 45167) **A–D, F–H** shell **E** enlarged apertural view.

Figs 111C , 116 , 154

Hypselostoma annamiticum Möllendorff, 1900: 133.
Hypselostoma annamiticum — Möllendorff 1901a: 50; Fischer and Dautzenberg 1904: 408; Pilsbry 1917: 180–181; Zilch 1984: 161; van Benthem Jutting 1950: 41.
Hypselostoma annamiticum altius Pilsbry, 1917: 181, pl. 31, figs 11–13. syn. nov.
Hypselostoma annamiticum altius — van Benthem Jutting 1950: 41; Schileyko 2011: 3.
Hypselostoma annamiticum annamiticum — Schileyko 2011: 3.

##### Type material examined.

**Vietnam** • holotype of *H.annamiticumannamiticum*; “Phuc-Son, Touranne, Annam”; ex. coll. Möllendorff; SMF 4871 • 11 paratypes of *H.annamiticumannamiticum*; same data as for the holotype; ex. coll. Möllendorff; SMF 4872 • 1 paralectotype of *H.annamiticumaltius*; from the type locality; ANSP 45167.

##### Type localities.

“Phuc-son”, Vietnam (*H.annamiticumannamiticum*), “Annam (Fruhstorfer)”, Vietnam (*H.annamiticumaltius*).

##### Differential diagnosis.

This species is superficially similar and geographically approximate to *A.turritus* sp. nov., but the suture of this species is shallower, the shell is more slender, the umbilicus is wider and the apertural dentition is also different (no concrescent barriers on the parietal side in the new species vs angulo-parietal lamella in *H.annamiticum*).

##### Distribution.

This species is known only from Annam, Vietnam.

##### Remarks.

Holotype of *H.annamiticumannamiticum* has a vestigial lamella between the columellar lamella and angulo-parietal lamella. Some paratypes do not have this lamella or it is further less developed, sometimes also located much deeper inside the aperture. In some specimens, upper palatal plica has an additional palatal tubercle which is sometimes connected with the former. *Hypselostomaannamiticumaltius* is synonymised with the nominotypical subspecies since we could observe no morphological differences. The fact that it is higher than the nominotypical subspecies (Pilsbry 1917) does not seem to justify its distinctness.

#### ﻿ Hypselostoma aquila

Taxon classificationAnimaliaStylommatophoraHypselostomatidae

Gojšina, Hunyadi & Páll-Gergely sp. nov.

F07A1734-8C12-57F0-B396-DF7E9AAE416F

https://zoobank.org/7C755981-9B66-44EC-8EF6-EAA68281930F

**Figure 117.**
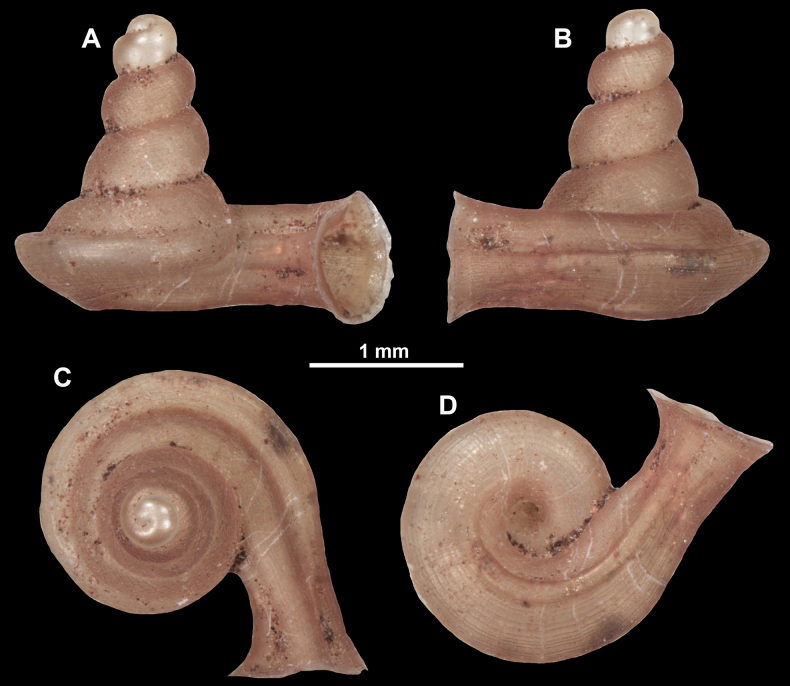
*Hypselostomaaquila* Gojšina, Hunyadi & Páll-Gergely, sp. nov., holotype (CUMZ 14461) **A–D** shell

**Figure 118.**
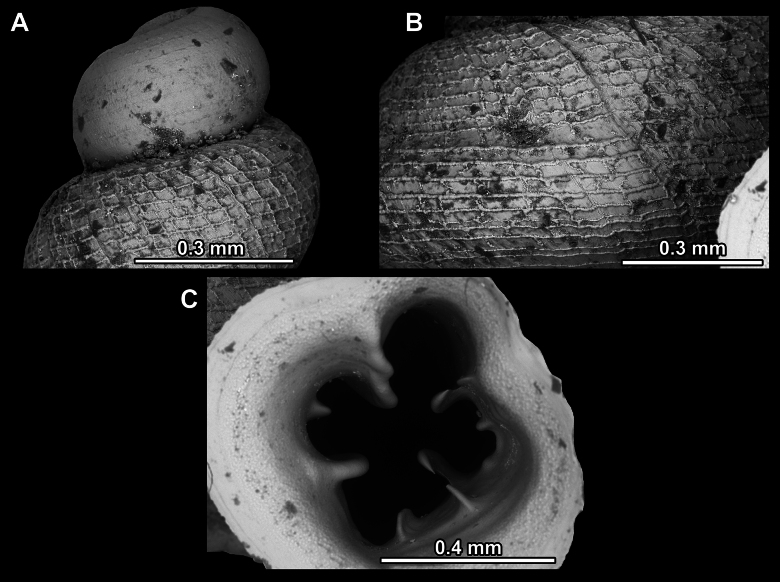
SEM imaging of *Hypselostomaaquila* Gojšina, Hunyadi & Páll-Gergely, sp. nov., holotype (CUMZ 14461) **A** protoconch surface **B** teleoconch surface **C** enlarged apertural view.

Figs 111D , 117 , 118 , 177

##### Type material.

***Holotype*. Cambodia** • 1 shell (SH: 1.98 mm; SW1: 2.39 mm); Steung Treng Province, 55.7 km northwest + 2 km north from Stung Treng Mekong Bridge, Chap Phleung Mt. (Neak Khiev Mt.); 13°47.821'N, 105°36.205'E; 135 m a.s.l.; 26 Oct. 2023, A. Hunyadi & J.U. Otani leg.; CUMZ 14461. ***Paratypes*. Cambodia** • 107 shells; same data as for holotype; coll. HA • 1 shell; same data as for holotype; coll. VG • 229 shells; Steung Treng Province, Stung Treng Mekong Bridge, 36.3 km NWN + 5 km N, Phnom Chhnok; 13°46.573'N, 105°44.878'E; 120 m a.s.l.; 25 Oct. 2023; A. Hunyadi & J.U. Otani leg.; coll. HA.

##### Additional material examined.

**Cambodia** • 4 shells (juveniles/ damaged, not paratypes); Steung Treng Province, Stung Treng Mekong Bridge, 36.3 km NWN + 5 km N, Phnom Chhnok; 13°46.573'N, 105°44.878'E; 120 m a.s.l.; 25 Oct. 2023; A. Hunyadi & J.U. Otani leg.; coll. HA.

##### Type locality.

Cambodia, Steung Treng Province, 55.7 km northwest + 2 km north from Stung Treng Mekong Bridge, Chap Phleung Mt. (Neak Khiev Mt.); 13°47.821'N, 105°36.205'E; 135 m a.s.l.

##### Diagnosis.

A *Hypselostoma* species with a slender, concave-conical shell, bluntly and strongly keeled last whorl (and a deep groove above it), raised spiral striation, hooked apertural barriers (upper palatal, and lower palatal) and a wide umbilicus which is initially narrow.

##### Description.

Shell concave-conical, light-brown, consisting of 4.5–5 whorls separated by a deep suture. Protoconch nearly white, consisting of ~ 1.25 coarsely spirally striated whorls. There are ~ 12 weak spiral striae on the protoconch. Protoconch and initial teleoconch whorls rounded, penultimate weakly convex. Last whorl with strong but blunt keel positioned at the centre of the periphery. Above the keel there is a deep groove on the last 0.75–1 whorl. All whorls densely sculptured with strong, thick spiral striae crossed by less dense radial growth lines and occasionally by a few thin, whitish radial streaks. Spacing between the spiral striae irregular and ranges from the width of two to the width of three spiral striae. Last whorl moderately detached from the penultimate and weakly ascending (≤ 10 ° compared to the shell axis). Peristome expanded, not reflected, its surface finely pitted. Aperture equipped with four strong, main barriers (angulo-parietal, upper palatal, lower palatal and columellar) and several smaller ones. Angulo-parietal lamella strong and leaned towards the palatal side, almost reaching the expanding peristome. It is consisting of main, parietal part, and a smaller, pointed angular part which are separated by a weak sinuation. Upper and lower palatal plicae roughly the same size, both divided and the inner parts are hooked (with the tip of hooks pointing outside), slender and high, while the outer parts are low and slender. A weak swelling is also observed at the palatal side (in front of the upper palatal plicae) which is probably homologous to the palatal tubercle in the majority of *Bensonella* species. Columellar lamella almost horizontal, approximately as high as the palatal plicae. There are four smaller barriers, two interpalatal plicae, one basal plica and one infraparietal lamella. Surface of all apertural barriers is smooth or with very weak granules. Sinulus rounded, distinctly separated from the rest of the aperture. Umbilicus initially narrow and then abruptly widening, measuring between 1/3 and 1/4 of the shell width. There is a periumbilical keel situated on the last ~ 0.75 whorl near the centre of the last whorl, but rather slightly towards the umbilicus. Along this keel there are shallow grooves on both sides.

##### Differential diagnosis.

See under *H.benetuitum*, *H.sorormajor* sp. nov., and *H.sororminor* sp. nov.

##### Measurements

**(in mm, *n* = 5).**SH = 1.91–2.04; SW1 = 2.16–2.39; SW2 = 1.35–1.42; AH = 0.80–0.86; AW = 0.69–0.82.

##### Etymology.

This species is named for the apertural barriers resembling claws of an eagle (*aquila* = eagle in Latin). To be used as a noun in apposition.

##### Distribution.

This species is known from two localities in Steung Treng province.

##### Remarks.

The largest, and still unexplored limestone hill in Steung Treng Province (13°47.941'N, 105°43.7671'E) was inaccessible during the collecting efforts in 2023 due to the works of a cement factory. Due to the proximity of this hill to the type locality of *H.aquila* sp. nov., it is possible that this species can be found here as well and be threatened by quarrying.

#### ﻿ Hypselostoma australe australe

Taxon classificationAnimaliaStylommatophoraHypselostomatidae

Odhner, 1917

C8B98D25-1281-5848-A1F2-73EC40401E94

Figs 111E , 119

Hypselostoma australis Odhner, 1917: 98, pl. 3, figs 107–109.
Gyliotrachela australis — Iredale 1937: 305; van Benthem Jutting 1950: 44; Pokryszko 1996: 1117, figs 31, 33, 34.
Gyliotrachela ningbingia Solem, 1981: 91, figs 3, 4, 10, 14–16, 18, 19.
Gyliotrachela catherina Solem, 1981: 91–92, figs 5, 6, 11, 17; Pokryszko 1996: 1117.
Gyliotrachela ninbingia [sic] — Pokryszko 1996: 1117.

##### Type material examined.

**Australia** • syntype of *H.australisaustralis*; SMNH 1667.

##### Type locality.

“caves at Chillagoe” (North Queensland, Australia).

##### Differential diagnosis.

This species is similar in shape to several Southeast Asian representatives, but it can be separated clearly by its different shell surface sculpture which consists of equally strong, rib-like radial growth lines and spiral striae. See also under *H.australenapierana*.

##### Distribution.

This species in known from several localities in N Australia as well as under several synonyms (Iredale 1937; Solem 1981).

##### Remarks.

Pokryszko (1996) synonymised *G.catherina* and *G.ningbingia* with *H.australe*, mentioning that the former two show only biometrical differences from *H.australe* which does not justify their distinctness.

**Figure 119.**
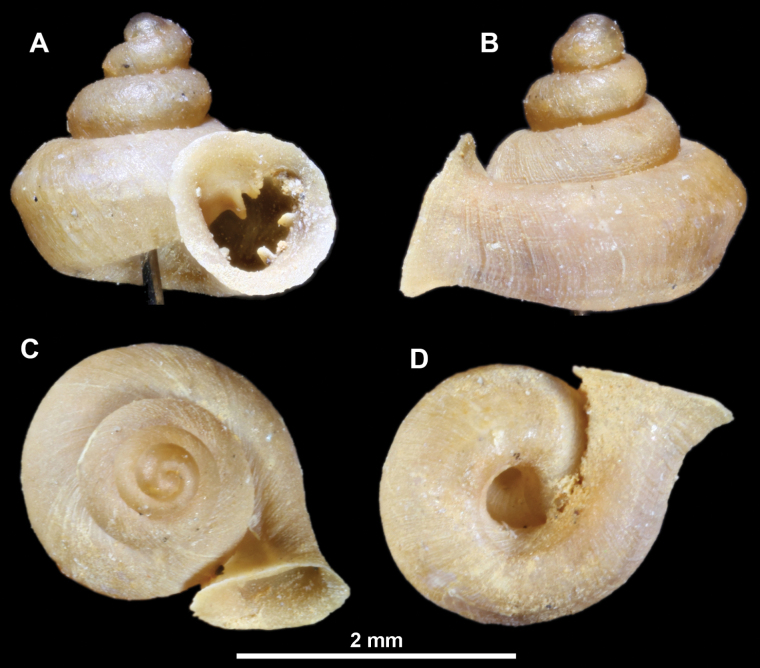
*Hypselostomaaustraleaustrale*, syntype (SMNH 1667) **A–D** shell.

#### ﻿ Hypselostoma australe napieranum

Taxon classificationAnimaliaStylommatophoraHypselostomatidae

(Solem, 1981) comb. nov.

85DACDD1-EDF4-5505-908A-F4380E1483E5

Gyliotrachela napierana Solem, 1981: 90–91, figs 1, 2, 9, 13.
Gyliotrachela australis napierana — Pokryszko 1996: 1117–1118, fig. 32.

##### Material examined.

None.

##### Type locality.

“Station WA-325, 0.6 km south-west of road along north side of Napier Range, 5.9 km north-west of Yammera Gap, Western Australia”.

##### Differential diagnosis.

This subspecies is different from the nominotypical by the more numerous and stronger apertural barriers.

##### Distribution.

This subspecies is known from several localities in Napier Range, western Australia.

#### ﻿ Hypselostoma benetuitum

Taxon classificationAnimaliaStylommatophoraHypselostomatidae

Vermeulen, Luu, Theary & Anker, 2019

3A1138DB-104A-5264-AEB0-BC20F3A8A63D

**Figure 120.**
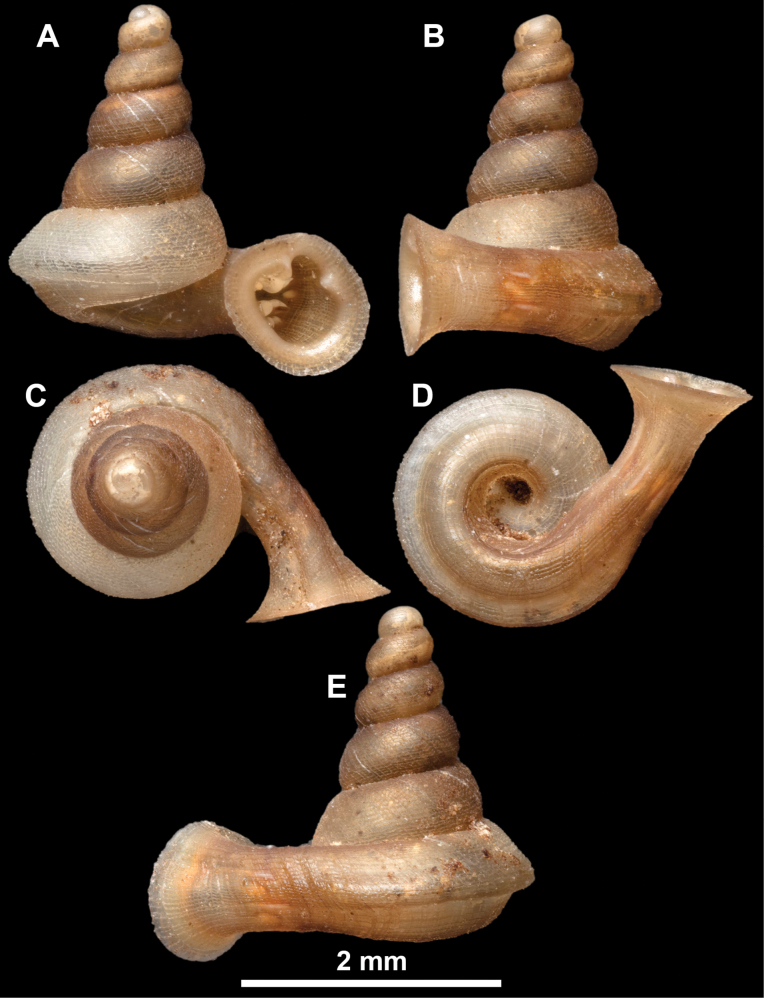
*Hypselostomabenetuitum*, holotype (NHMUK 20180575) **A–E** shell.

Figs 111F , 120 , 182

Hypselostoma benetuitum Vermeulen et al., 2019: 38–39, figs 64–68.
Hypselostoma benetuitum — Sutcharit et al. 2020: 18, fig. 7E, F; Sutcharit et al. 2023: 1290.

##### Type material examined.

**Cambodia** • holotype; J. Vermeulen leg.; NHMUK 20180575.

##### Type locality.

“Phnom Kampong Trach”, Cambodia.

##### Differential diagnosis.

This species is similar in shell shape to other Cambodian representatives (*H.discobasis*, *H.sorormajor* sp. nov., *H.sororminor* sp. nov., *H.aquila* sp. nov.) but differs from all of them by the presence of a keel with a concave surface below it but flat above it (vice versa in other species). Furthermore, this species has hooked apertural barriers in contrast to *H.discobasis* and *H.sororminor* sp. nov. See also under *H.cambodjense*.

##### Distribution.

This species is known from Kampot province, Cambodia.

#### ﻿ Hypselostoma bensonianum

Taxon classificationAnimaliaStylommatophoraHypselostomatidae

W.T. Blanford, 1863

3B188960-22AF-5A64-BE96-BC58CF98526A

**Figure 121.**
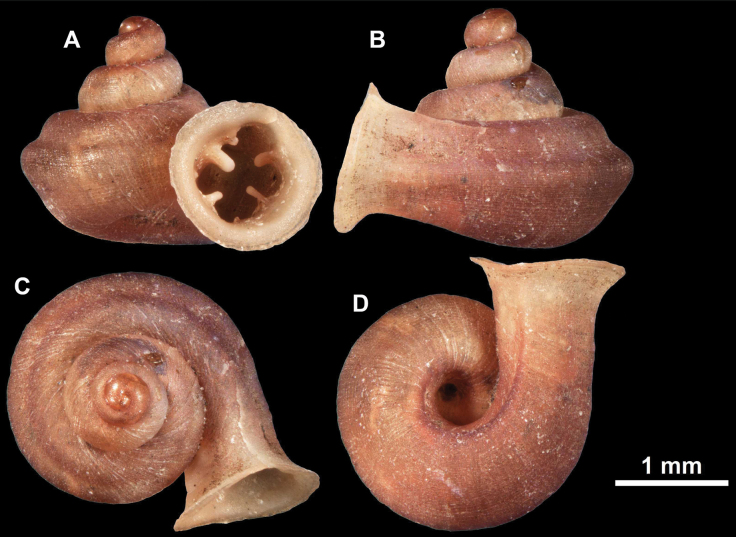
*Hypselostomabensonianum*, syntype (NHMUK 20191141) (from Gojšina et al. 2022) **A–D** shell.

Figs 111G , 121 , 158

Hypselostoma bensonianum Blanford, 1863: 326–327.
Hypselostoma bensonianum — Hanley and Theobald 1870: 4, pl. 8 fig. 2; Pfeiffer 1875: 488; Möllendorff 1891: 338; Gude 1914: 299.
Pupa (Hypselostoma) bensoni — Nevill 1878: 193.
Gyliauchen bensonianus — Pilsbry 1917: 211; pl. 37 figs 11, 12.
Gyliotrachela bensoniana — van Benthem Jutting 1950: 44.
Gyliotrachela bensonianum — Gojšina et al. 2022: 136–137, figs 4–5; Tongkerd et al. 2024: 179, fig. 13L.

##### Type material examined.

**Myanmar** • 5 syntypes; NHMUK 20191141.

##### Type locality.

“Mya Leit Doung, Ava” (nowadays Melai Taung 21.8°N, 96.2°E).

##### Differential diagnosis.

This species differs from all other congeners by its keeled last whorl and spirally striated teleoconch in combination with a small number of relatively strong apertural barriers (usually up to seven). See also under *H.aunglini*.

##### Distribution.

This species is known only from the type locality.

#### ﻿ Hypselostoma cambodjense

Taxon classificationAnimaliaStylommatophoraHypselostomatidae

van Benthem Jutting, 1962

48CA0F81-C7CF-5488-8677-77A1045A9067

**Figure 122.**
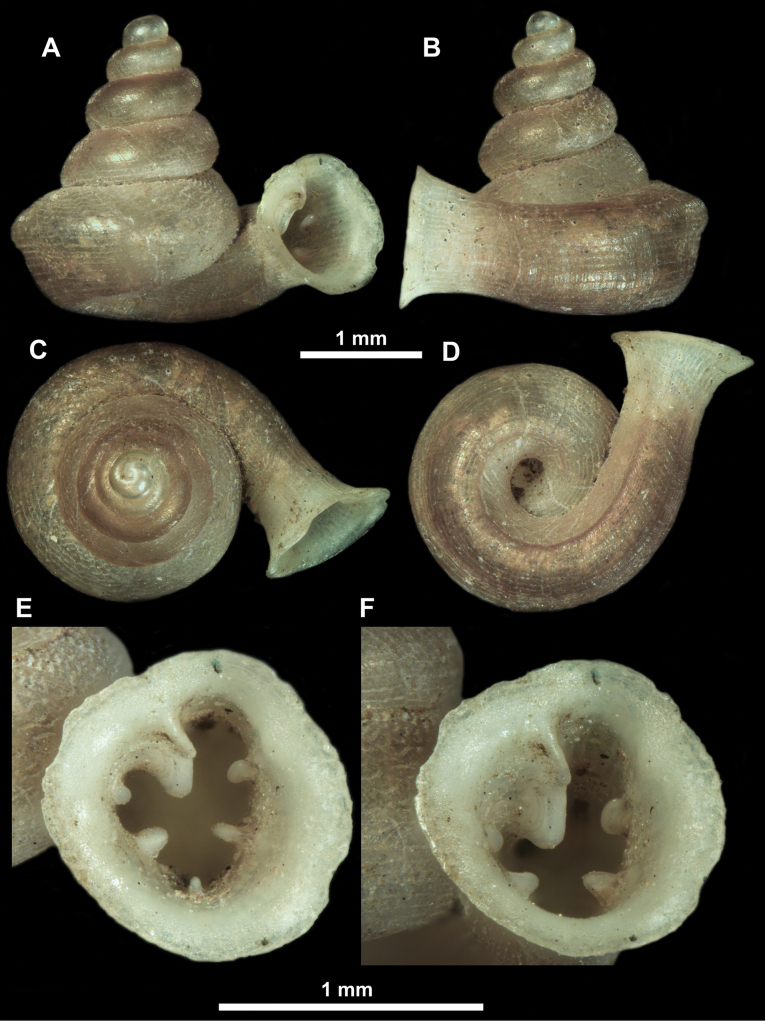
*Hypselostomacambodjense*, paratype (RMNH Moll.137169) **A–D** shell **E, F** enlarged apertural views.

Figs 111H , 122 , 182

Hypselostoma cambodjense van Benthem Jutting, 1962: 3–5, fig. 1.
Hypselostoma cambodjense — Vermeulen et al. 2019: 39; Sutcharit et al. 2020: 18, figs 3H, 8A–C; Sutcharit et al. 2023: 1290.

##### Type material examined.

**Cambodia** • 4 paratypes; from the type locality; 1960; E. Saurin leg.; RMNH.Moll.137169.

##### Type locality.

“Phnom Can Long, à 6 km au Sud de Tuk Méas, Cambodge”.

##### Differential diagnosis.

This species differs from *H.benetuitum* by the absence of hooked apertural barriers. Also, the keel is slightly sharper in *H.benetuitum* with a more concave surface below it.

##### Distribution.

This species in known from Kampot province (Cambodia) and Kien Giang province (Vietnam) (Vermeulen et al. 2019).

##### Remarks.

Of four paratypes examined, one of them had the last whorl fully adnate to the penultimate.

#### ﻿ Hypselostoma chaunosalpinx

Taxon classificationAnimaliaStylommatophoraHypselostomatidae

(Vermeulen, Luu, Theary & Anker, 2019) comb. nov.

481B339C-BD1D-5132-BEE3-54F346A4FDCB

**Figure 123.**
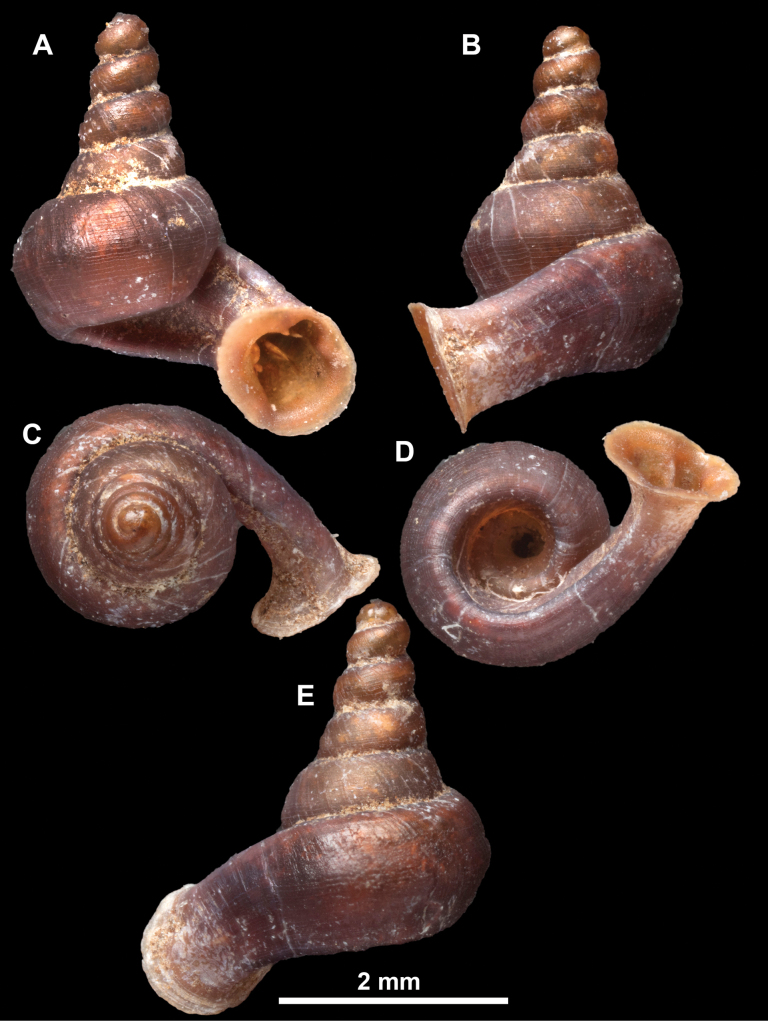
*Hypselostomachaunosalpinx*, holotype (NHMUK 20180573) **A–E** shell.

Figs 111I , 123 , 182

Anauchen chaunosalpinx Vermeulen et al., 2019: 35, figs 57–59.
Anauchen chaunosalpinx — Sutcharit et al. 2023: 1290.

##### Type material examined.

**Cambodia** • holotype; NHMUK 20180573.

##### Type locality.

“Cambodia: Kampot Province, Kampong Trach area: Phnom Kampong Trach”.

##### Differential diagnosis.

This species is not similar to any other congener due to the combination of conical shell with wide last whorl which is strongly descending and has several hooked apertural barriers. See under *H.depressum*.

##### Distribution.

This species is known only from the type locality.

#### ﻿ Hypselostoma chedi

Taxon classificationAnimaliaStylommatophoraHypselostomatidae

Panha, 1998

F0E65A96-447B-5B67-A47F-6595A1846C14

**Figure 124.**
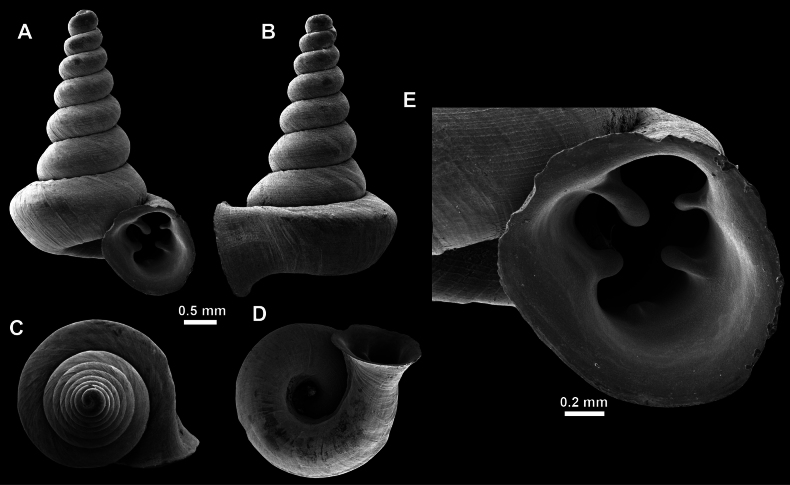
*Hypselostomachedi*, paratype (CUMZ ver. 004) **A–D** shell **E** enlarged apertural view.

Figs 111J , 124 , 154

Hypselostoma chedi Panha, 1998b: 61–63, fig. 2.
Anauchen chedi — Burch and Panha 2002: 246, fig. 4; Tongkerd et al. 2004: 143, fig. 2; Panha and Burch 2008: 50–51, fig. 46; Dumrongrojwattana and Wongkamhaeng 2024: 323, fig. 7.
Hypselostoma chedi — Hemmen and Hemmen 2001: 41.

##### Type material examined.

**Thailand** • paratype; S. Panha leg.; CUMZ ver. 004; 2 paratypes; from the type locality; SMF 331470.

##### Additional material examined.

**Thailand** • 36 shells; Nakhon Sawan Province, 19.5 km NE intersection Hwys 11 and 32; 15°14'N, 100°16'E; 50 m a.s.l.; 02. May 1988; K. Auffenberg leg.; locality code KA-0696; UF 346127/2, UF 346138/34.

##### Type locality.

“Thailand, Tebpratan Nature Reserve area, Kampangpet Province, 15°54′52″N, 99°52′63″E, 140 meters elevation”.

##### Differential diagnosis.

see under *H.smokon* and *H.khaochakan*.

##### Distribution.

This species is known from Nakhon Sawan and Kampangpet provinces.

##### Remarks.

Coordinates provided in the original description are not correct.

#### ﻿ Hypselostoma circumcarinatum

Taxon classificationAnimaliaStylommatophoraHypselostomatidae

Gojšina, Auffenberg & Páll-Gergely sp. nov.

44A493DF-BA62-5C4F-B85B-476735F7175E

https://zoobank.org/C5BBC776-A6D4-4A94-B11E-22B2D8A1C10B

**Figure 125.**
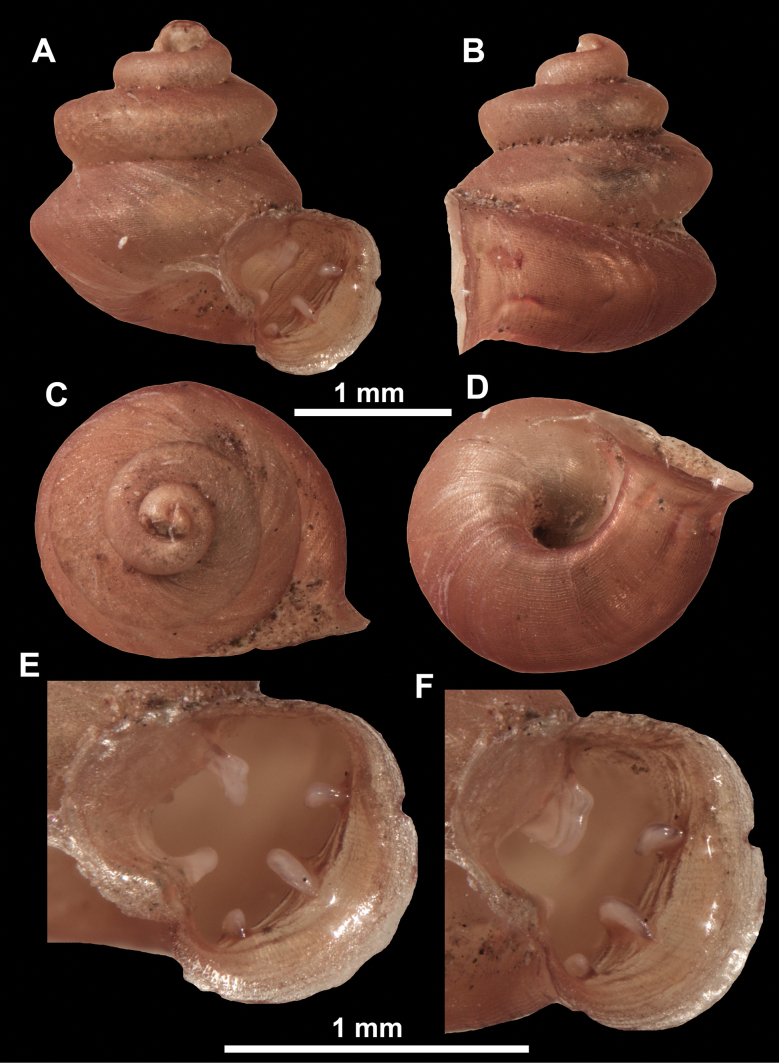
*Hypselostomacircumcarinatum* Gojšina, Auffenberg & Páll-Gergely, sp. nov., holotype (UF 345933) **A–D** shell **E, F** enlarged apertural views.

**Figure 126.**
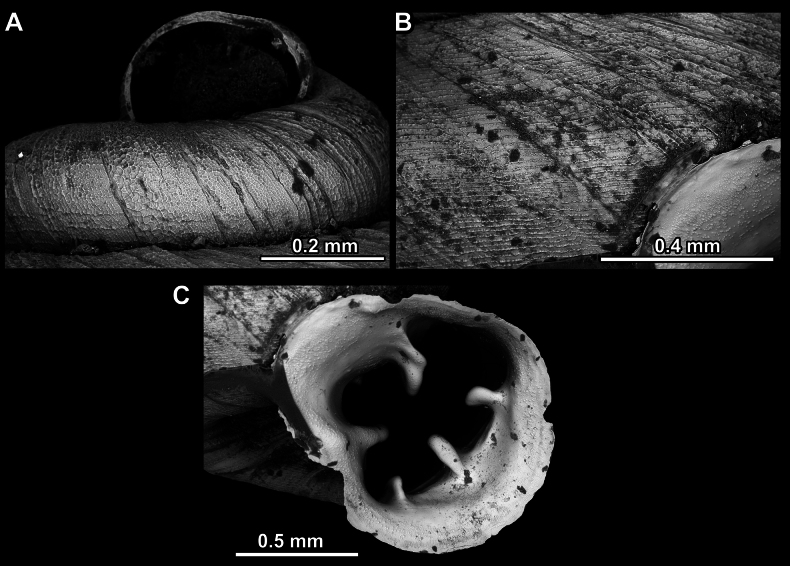
SEM imaging of *Hypselostomacircumcarinatum* Gojšina, Auffenberg & Páll-Gergely, sp. nov., holotype (UF 345933) **A** protoconch surface **B** teleoconch surface **C** enlarged apertural view.

Figs 111K , 125 , 126 , 154

##### Type material.

***Holotype*. Thailand** • 1 shell (SH: 2.3 mm; SW: 2.2 mm); Kanchanaburi Province, 4.3 km SW Kanchanaburi, Taoist Buddhist temple; 13°59'N, 99°31'E; 40 m a.s.l.; 13. May 1988, K. Auffenberg leg.; UF 345933. ***Paratypes*. Thailand** • 1 shell; same data as for holotype; CUMZ 14456 • 3 shells (2 whole and 1 with complete aperture); same data as for holotype; UF 591336.

##### Additional material examined.

**Thailand** • 4 shells (damaged, without last whorl, not paratypes); same data as for holotype; UF 583722.

##### Type locality.

Kanchanaburi Province, 4.3 km SW Kanchanaburi, Taoist Buddhist temple; 13°59'N, 99°31'E; 40 m a.s.l.

##### Diagnosis.

*Hypselostoma* species with very deep sutures and spirally striated whorls. Penultimate and the last whorl are keeled. Last whorl not detached from the penultimate. Aperture equipped with six barriers (angulo-parietal, upper palatal, lower palatal, basal, columellar and a small infraparietal). Umbilicus initially narrow, abruptly widening at the last whorl.

##### Description.

Shell conical-ovoid, consisting of 4–4.5 whorls separated by a deep suture. Protoconch pitted, showing some spiralling pattern, the same colour as the rest of the shell, consisting of ~ 1.5 whorls. Teleoconch surface sculptured with coarsely spaced radial lines crossed by much stronger and dense spiral striae. Spacing between two spiral striae approximates the width of one or two striae. First teleoconch whorl rounded. All other teleoconch whorls strongly keeled, last one the most prominent. Surface above the keel on the penultimate whorl is almost flat, (like a platform) but is much more steep (sloping) on the last whorl. Last whorl adnate to the penultimate and slightly ascending near the aperture (~ 5–10 ° compared to the shell axis). Peristome not very strong but expanded, not reflected. It is forming a weak callus on the parietal side. Aperture equipped with five main barriers (angulo-parietal, upper palatal, lower palatal, basal and columellar) and one small infraparietal lamella which is knob-like (although it can be absent as well). Angulo-parietal lamella is the strongest in the aperture, consisting of larger parietal part and indistinct angular part so that it appears slightly bilobed. Upper palatal plica short, weaker than the lower palatal and slightly bent towards it. Lower palatal plica roughly as strong as the columellar, situated halfway between upper palatal and basal plica. Basal plica small, slightly weaker than the upper palatal. Columellar lamella strong, directed towards the upper palatal plica and almost reaching the expanding peristome. Peristome is weakly swollen in front of the columellar lamella. Surface of all apertural barriers is finely granulated to almost smooth. Sinulus wide and not distinctly separated from the rest of the aperture. Umbilicus initially very narrow and then abruptly widening at the last whorl, measuring ~ 1/5–1/6 of the shell width. A weak groove is visible running alongside the umbilicus but it is getting lost near the middle part of the last whorl.

##### Differential diagnosis.

See under *H.sculpturatum* and *H.khmerianum*.

##### Measurements

**(in mm, *n* = 4).**SH = 1.93–2.46; SW = 2.09–2.24; AH = 1.02–1.16; AW = 1.05–1.18.

##### Etymology.

The two largest whorls (last and the penultimate) are keeled which gives the initial impression that the whole shell is keeled, hence the specific epithet.

##### Distribution.

This species is known only from the type locality.

##### Remarks.

Some specimens had a more depressed shell which was even slightly wider than high.

#### ﻿ Hypselostoma cultura

Taxon classificationAnimaliaStylommatophoraHypselostomatidae

(Tanmuangpak & Dumrongrojwattana, 2022) comb. nov.

4F79B037-4FEE-57BF-A78B-9D8FC032481A

**Figure 127.**
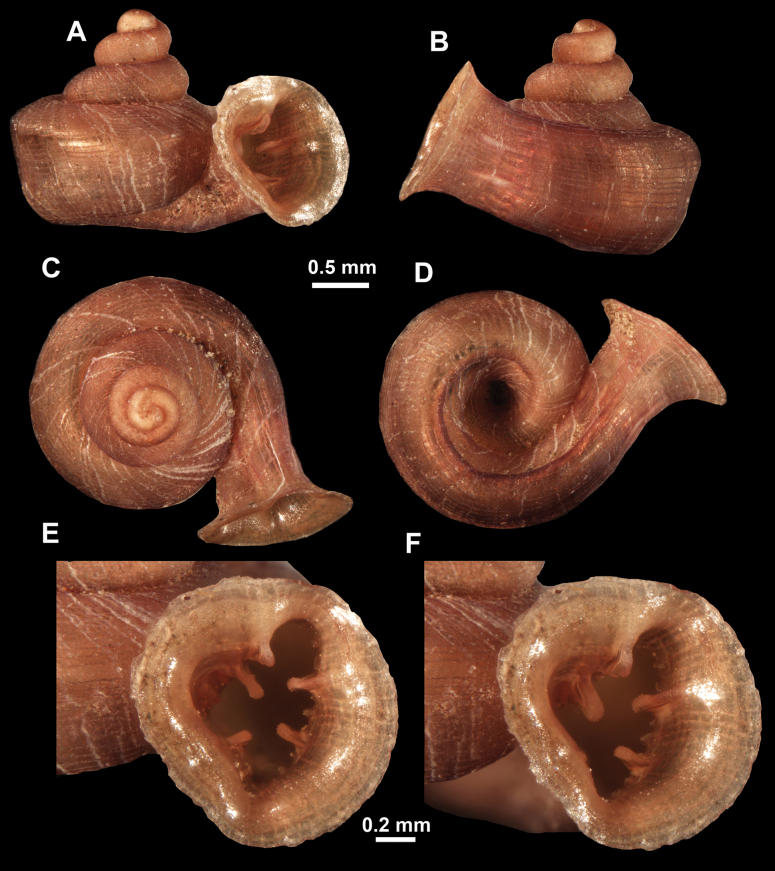
Hypselostomacf.cultura from Ko Sichang Island, Chonburi province (coll. HA) **A–D** shell **E, F** enlarged apertural views.

Figs 111M , 127 , 154

Gyliotrachela cultura Tanmuangpak & Dumrongrojwattana, 2022: 408–415, fig. 2.

##### Material examined.

**Thailand** • 52 shells; Chonburi Province, Ko Sichang, Tha Thewawong, vicinity of Mondop Roi Phraphutthabat; 13°10.256'N, 100°48.305'E; 140 m a.s.l.; 11 Mar. 2023; A. Hunyadi leg.; coll. HA • 3 shells; Chonburi Province, Si Racha, Ko Si Chang; 25 Oct. 2019; J.U. Otani leg.; coll. PGB • 2 shells; Lampang Province, Maemoh district, Wat Tam Innaeramitr; 10 Aug. 2014; J.U. Otani leg.; coll. PGB.

##### Type locality.

“Agricultural areas in Mueang Loei District, Loei Province, Thailand (17°34′43.020″N, 101°51′04.020″E)”.

##### Differential diagnosis.

See under *H.khaowongense*.

##### Distribution.

This species is, apart from the type locality in Loei province, known from two more localities: one in Lampang and one in Chonburi province.

##### Remarks.

This species might be conspecific with *H.khaowongense*. The differences mentioned by Tanmuangpak and Dumrongrojwattana (2022) between this species and *H.khaowongense* (therein under *Gyliotrachelasaraburiensis*) are only regarding the number of apertural barriers, which are very variable in *H.khaowongense*. We have noticed additionally that the shells of *H.cultura* are strongly shouldered when observed laterally (e.g., as in Tanmuangpak and Dumrongrojwattana 2022: Fig. 2E, K) which is not typical for *H.khaowongense*. Due to the absence of relevant comparative material, we do not formally synonymise the two species. Specimens examined by us from Chonburi (Fig. 127) and Lampang provinces show very similar shell morphology as figured in Tanmuangpak and Dumrongrojwattana (2022), just with a wider umbilicus. We do not intend to describe this species as separate from *H.cultura* until more comparative material becomes available.

#### ﻿ Hypselostoma cucumense

Taxon classificationAnimaliaStylommatophoraHypselostomatidae

Panha, 1998

A15C56E7-CB1B-581C-8B04-FC2C5095E548

**Figure 128.**
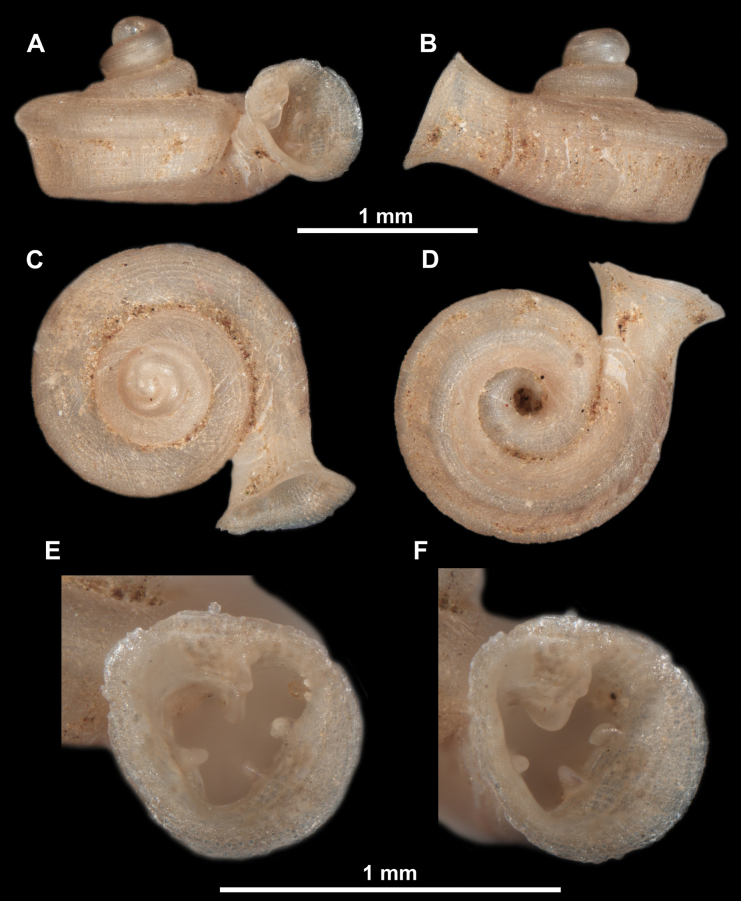
*Hypselostomacucumense* from Phetchaburi province (near the type locality, coll. HA) **A–D** shell **E–F** enlarged apertural views.

Figs 111L , 128 , 154

Hypselostoma cucumensis Panha, 1998b: 65–66, figs 1b, 3.
Hypselostoma cucumensis — Hemmen and Hemmen 2001: 41; Panha and Burch 2008: 90, fig. 77; Tongkerd et al. 2004: 143, fig. 2; Dumrongrojwattana and Wongkamhaeng 2024: 324, fig. 8.

##### Material examined.

**Thailand** • 35 shells; Phetchaburi Province, Khao Yoi, northeastern side of the mountain; 13°14.316'N, 99°49.493'E; 30 m a.s.l.; 24 Feb. 2015; A. Hunyadi leg.; coll. HA.

##### Type locality.

“Limestone hills at Kangkrajan National Park, Petchaburi Province, 13°14′9″N, 99°49′43″E, 80 meters elevation” (Thailand).

##### Differential diagnosis.

This species resembles *H.adela* in shell size, surface sculpture and colouration. However, in *H.adela*, the angular and parietal lamellae are separated, the barriers are generally stronger and the last whorl is always at least slightly descending (slightly ascending in *H.cucumense*). See also under *H.populare* sp. nov. and *H.khaochongpran*.

##### Distribution.

This species is known from the type locality and its immediate surroundings.

#### ﻿ Hypselostoma depressum

Taxon classificationAnimaliaStylommatophoraHypselostomatidae

(Vermeulen, Luu, Theary & Anker, 2019) comb. nov.

5708339F-AFB4-5FC3-B90A-B009FD182773

**Figure 129.**
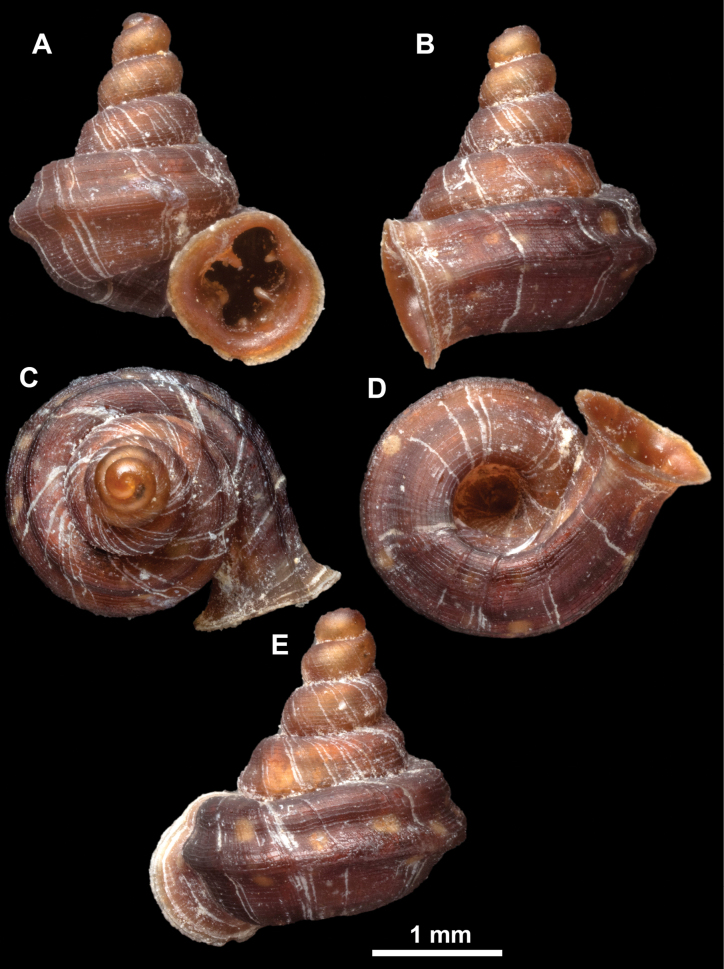
*Hypselostomadepressum*, holotype (NHMUK 20180574) **A–E** shell.

Figs 111N , 129 , 182

Anauchen depressus Vermeulen et al., 2019: 35–37, figs 60–63.
Anauchen depressus — Sutcharit et al. 2023: 1290.

##### Type material examined.

**Cambodia** • holotype; NHMUK 20180574.

##### Type locality.

“Cambodia: Kampot Province, Kampot area: Phnom La’Ang, cave with shrine at its entrance, first chamber with collapsed roof…southeast end…”.

##### Differential diagnosis.

This species differs from *H.chaunosalpinx* by the keeled last whorl and less numerous apertural barriers, none of which are hooked. The umbilicus is also wider in *H.chaunosalpinx*. See also under *H.taehwani*.

##### Distribution.

This species is known only from the type locality.

##### Remarks.

The type locality of this species was inaccessible during collecting efforts in 2023 due to the restricted access and the presence of concrete walls around the locality. This indicates that the species could be seriously threatened by quarrying.

#### ﻿ Hypselostoma diarmaidi

Taxon classificationAnimaliaStylommatophoraHypselostomatidae

(Panha & J.B. Burch, 2003) comb. nov.

9D3638C4-2F88-5881-A13C-F3B751B4DEE6

**Figure 130.**
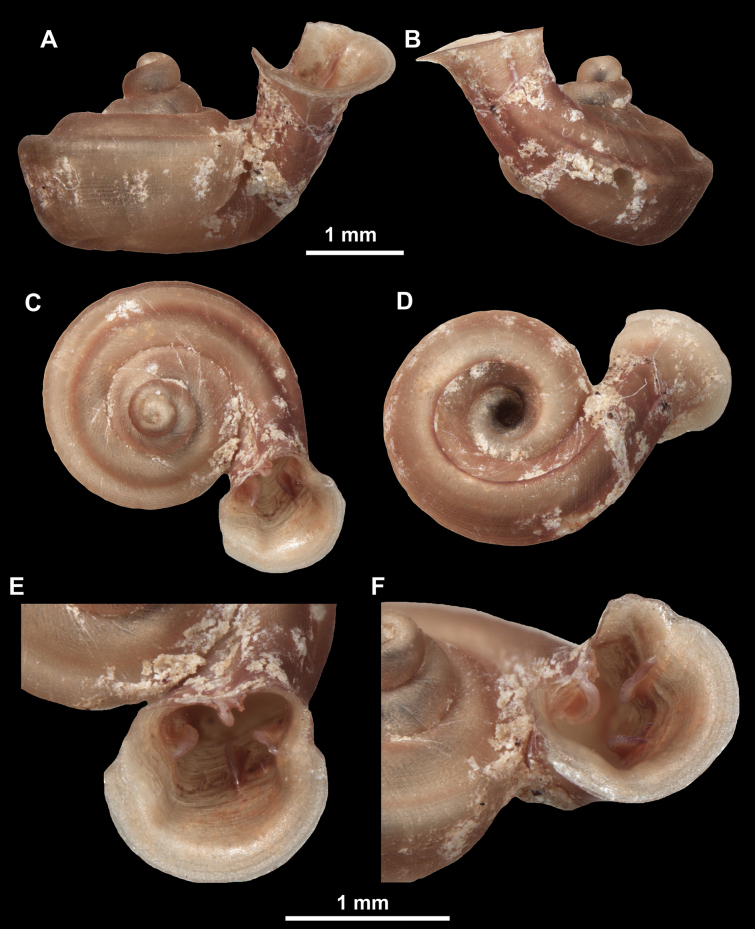
*Hypselostomadiarmaidi* from near the type locality (coll. PGB) **A–D** shell **E, F** enlarged apertural views.

**Figure 131.**
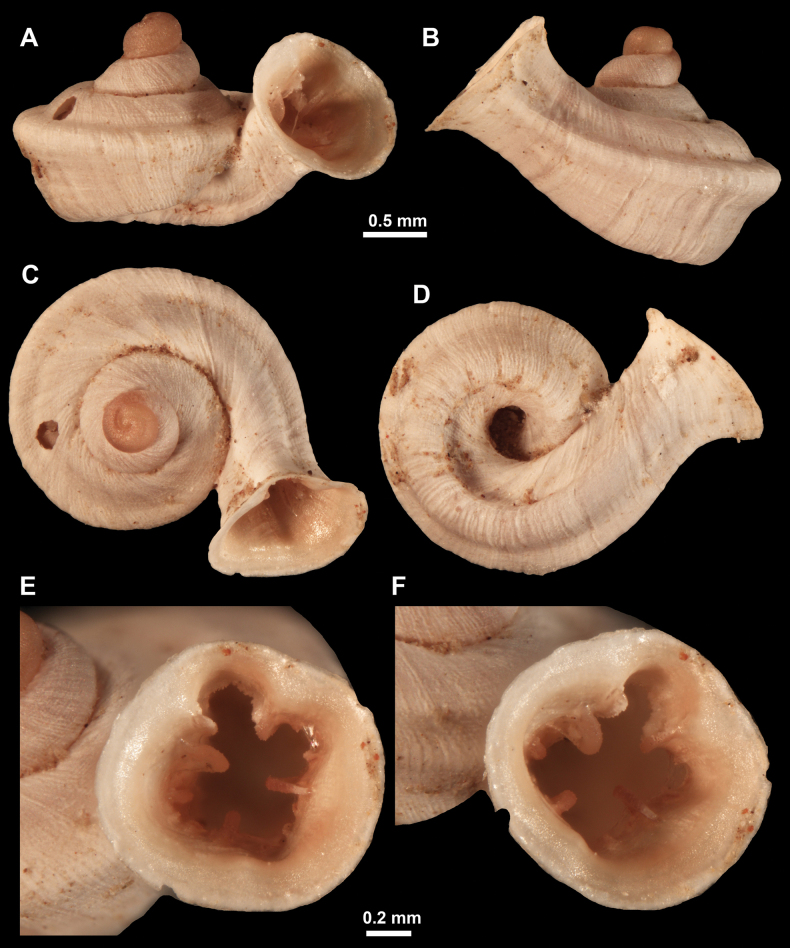
*Hypselostomadiarmaidi* from Songkhla province (UF 344927) **A–D** shell **E, F** enlarged apertural views.

Figs 111O , 130 , 131 , 154

Gyliotrachela diarmaidi Panha & Burch in Burch et al. 2003: 157–162, figs 10, 11.
Gyliotrachela diarmaidi — Tongkerd et al. 2004: 143, fig. 2; Panha and Burch 2008: 67–69, fig. 59; Dumrongrojwattana and Wongkamhaeng 2024: 323, fig. 8.

##### Material examined.

**Thailand** • 2 shells; Rayong Province, Wang Chan; 10 Aug. 2013 • 9 shells; Songkhla Province, 31.3 km NW Hat Yai 1.2 km W of Hwy. 43. Limestone outcrop, evergreen forest, leaf litter, roots and sticks, small solution pits; 7.1667°N, 100.2667°E; 80 m a.s.l.; 08 Apr. 1988; K. Auffenberg leg.; UF 344927 • 1 shell; same data as previous; locality code KA-0636B; UF 344944.

##### Type locality.

“Pluangthong Mountain, west of Pluangthong Temple, Botong District, Chonburi Province, 13°11'5"N, 101°34'59"E, 110 meters elevation, Thailand”.

##### Differential diagnosis.

See under *H.kohrin* and *H.surakiti*.

##### Distribution.

Apart from the type locality, this species is known from one other sampling site near the type locality as well as from the Rayong, Sa Kaeo, Prachinburi, and Songkhla provinces (Dumrongrojwattana and Wongkamhaeng 2024).

#### ﻿ Hypselostoma dilatatum

Taxon classificationAnimaliaStylommatophoraHypselostomatidae

van Benthem Jutting, 1962

B839F2EC-620B-5114-979C-A2D3A83BB218

**Figure 132.**
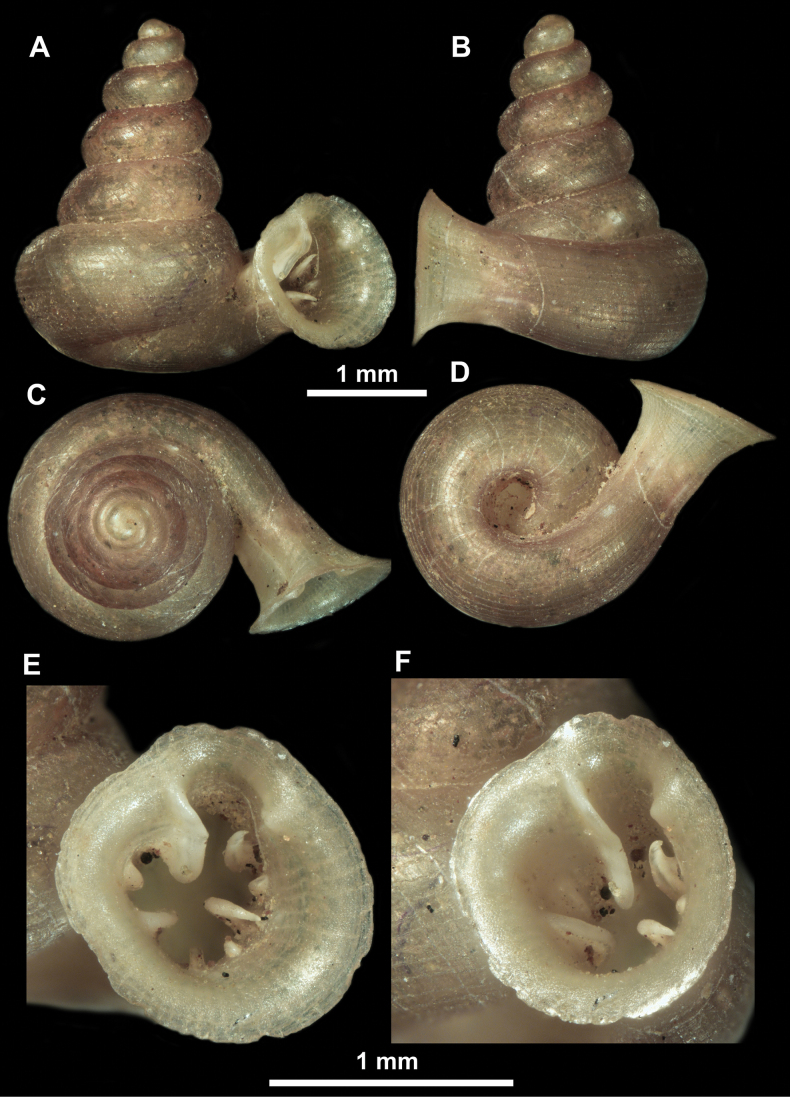
*Hypselostomadilatatum*, paratype (RMNH Moll.137167) **A–D** shell **E, F** enlarged apertural views.

Figs 111P , 132 , 182

Hypselostoma dilatatum van Benthem Jutting, 1962: 5–6, fig. 2.
Hypselostoma dilatatum — Schileyko 2011: 3; Vermeulen et al. 2019: 39.

##### Type material examined.

**Vietnam** • 2 paratypes; Nui Xo Ngach, 16 km SE of Haiten; 1960; E. Saurin leg.; RMNH.Moll.137167.

##### Additional material examined.

**Vietnam** • 4 shells; Kien Giang Province, Kien Luong district, Binh An commune, Ba Nui village, cave Mo So, limestone rocks; 10°13.659'N, 104°36.975'E; 18 Sept. 2018; I. Dedov, N. Simov, R. Bekchiev, P. Beron leg., coll. PGB.

##### Type locality.

“Xa Ngach, à 16 km. au S.-E. de Hatien, Sud Vietnam”.

##### Differential diagnosis.

This species is similar to *H.cambodjense* from which it differs by its hooked apertural barriers and a narrower umbilicus. See also under *H.rupestre*.

##### Distribution.

This species is known from the Vietnam part of the Mekong Delta limestone.

##### Remarks.

One paratype is figured, the second paratype was corroded and with broken hooked barriers in the aperture. It was also slightly larger than the figured specimen.

#### ﻿ Hypselostoma discobasis

Taxon classificationAnimaliaStylommatophoraHypselostomatidae

Vermeulen, Luu, Theary & Anker, 2019

DE7A437E-A5D7-5E68-9019-5C1AB86ED722

**Figure 133.**
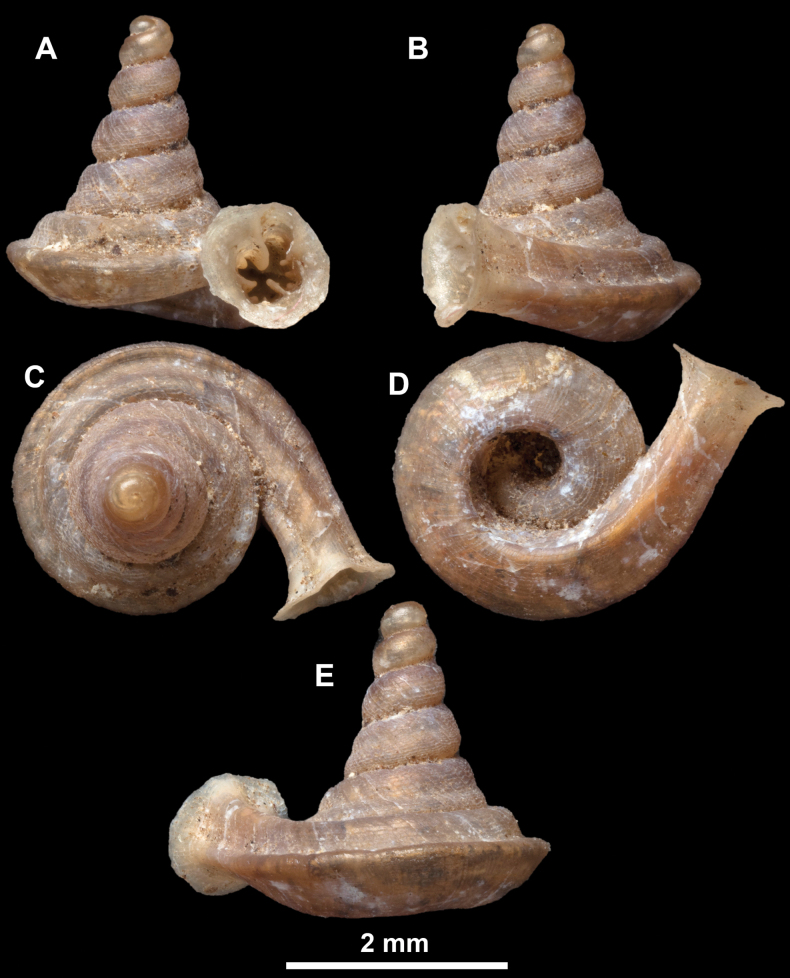
*Hypselostomadiscobasis*, holotype (NHMUK 20180576) **A–E** shell.

Figs 111Q , 133 , 182

Hypselostoma discobasis Vermeulen et al., 2019: 39–40, figs 66–68.
Hypselostoma discobasis — Sutcharit et al. 2023: 1290.

##### Type material examined.

**Cambodia** • holotype; NHMUK 20180576.

##### Type locality.

“Cambodia: Kampot Province, Kampot area: Phnom La’Ang, southeast-end”.

##### Differential diagnosis.

See under *H.sorormajor* sp. nov. and *H.sororminor* sp. nov.

##### Distribution.

This species in known only from the type locality.

##### Remarks.

The type locality of this species was inaccessible during collecting efforts in 2023 due to the restricted access and the presence of concrete walls around the locality. This indicates that the species could be seriously threatened by quarrying.

#### ﻿ Hypselostoma edentatum

Taxon classificationAnimaliaStylommatophoraHypselostomatidae

(Panha & J. B. Burch, 2002)

1F3670D6-19EB-5212-998F-3831EF24233F

**Figure 134.**
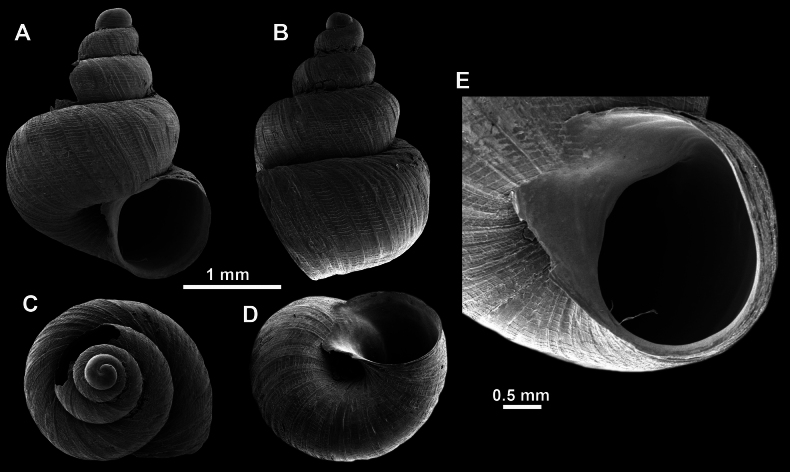
*Hypselostomaedentatum*, holotype (CUMZ ver.022) **A–D** shell **E** enlarged apertural view.

Figs 111R , 134 , 154

Systenostoma edentatum Panha & Burch, 2002d: 121–124, fig. 4.
Systenostoma edentata — Panha and Burch 2008: 119–120, fig. 102.
Hypselostoma edentata — Jochum et al. 2014: 31; Dumrongrojwattana and Wongkamhaeng 2024: 324, fig. 8.

##### Type material examined.

**Thailand** • holotype; 1997; S. Panha leg.; CUMZ ver.022.

##### Additional material examined.

**Thailand** • 12 shells; Chiang Rai Province, limestone knoll, 4 km NE of Ban Pa Ngae limestone knoll, base of limestone ledge; 19°34.3167'N, 99°59.2333'E; 410 m a.s.l.; F. G. Thompson leg.; 12. May 1988; locality code FGT-4429; UF 380304.

##### Type locality.

“Tamphatai National Park…, Phrae Province, 18°36”20'N, 99°53”49'E, 650 meters elevation…, Thailand…”.

##### Differential diagnosis.

This species differs from all of its congeners by the absence of apertural barriers, which makes it superficially more similar to some *Tonkinospira* species. From all similar toothless *Aulacospira* species from Thailand, this species differs by the presence of raised spiral striation. See also under *H.panhai*.

##### Distribution.

This species in known from Lampang and Chiang Rai provinces in Thailand.

##### Remarks.

The type locality of this species is not in Phrae but in Lampang province. Specimens from Chiang Rai province shared the same shell morphology but were slightly more depressed than the holotype.

#### ﻿ Hypselostoma erawan

Taxon classificationAnimaliaStylommatophoraHypselostomatidae

Panha & J. B. Burch, 2003

988ED459-6B5B-5B25-8865-3E486C6D3832

**Figure 135.**
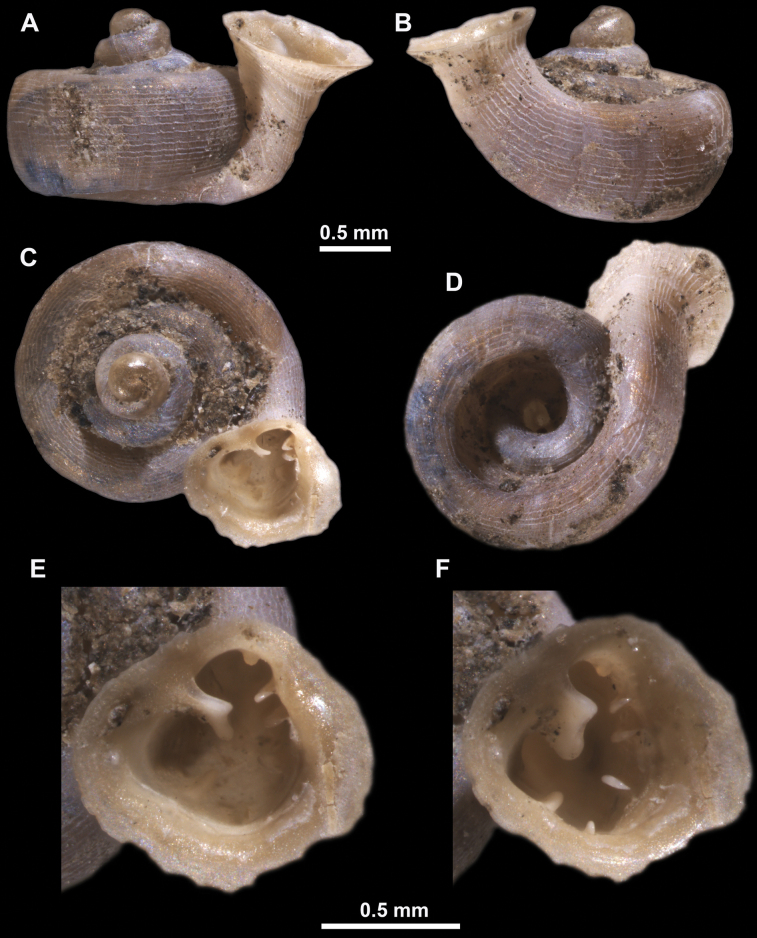
*Hypselostomaerawan*, paratype (SMF 331455) **A–D** shell **E, F** enlarged apertural views.

Figs 111S , 135 , 154

Hypselostoma erawan Panha & Burch in Burch et al. 2003: 169–174, figs 14, 15.
Hypselostoma erawan — Tongkerd et al. 2004: 145, fig. 4A–C; Panha and Burch 2008: 91–92, fig. 78; Dumrongrojwattana and Wongkamhaeng 2024: 324, fig. 8.

##### Type material examined.

**Thailand** • 3 paratypes; from the type locality; SMF 331455.

##### Type locality.

“A limestone mountain in the southern part of Erawan National Park, Triyoke District, Karnchanaburi Province, 14°12'15"N, 99°7'58"E, 70 meters elevation, Thailand”.

##### Differential diagnosis.

See under *H.populare* sp. nov.

##### Distribution.

This species is known only from the type locality.

#### ﻿ Hypselostoma everetti

Taxon classificationAnimaliaStylommatophoraHypselostomatidae

E. A. Smith, 1896

7A5D2E44-9F7D-5849-B4C7-E64BDBD5A197

**Figure 136.**
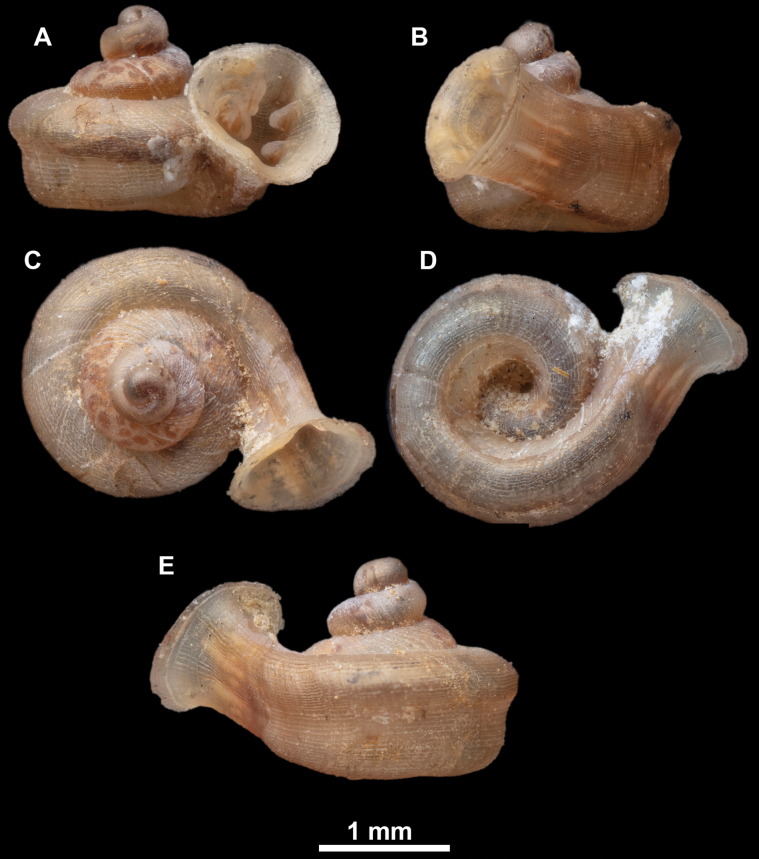
*Hypselostomaeveretti*, syntype (NHMUK 1896.5.17.1–6) **A–E** shell.

**Figure 137.**
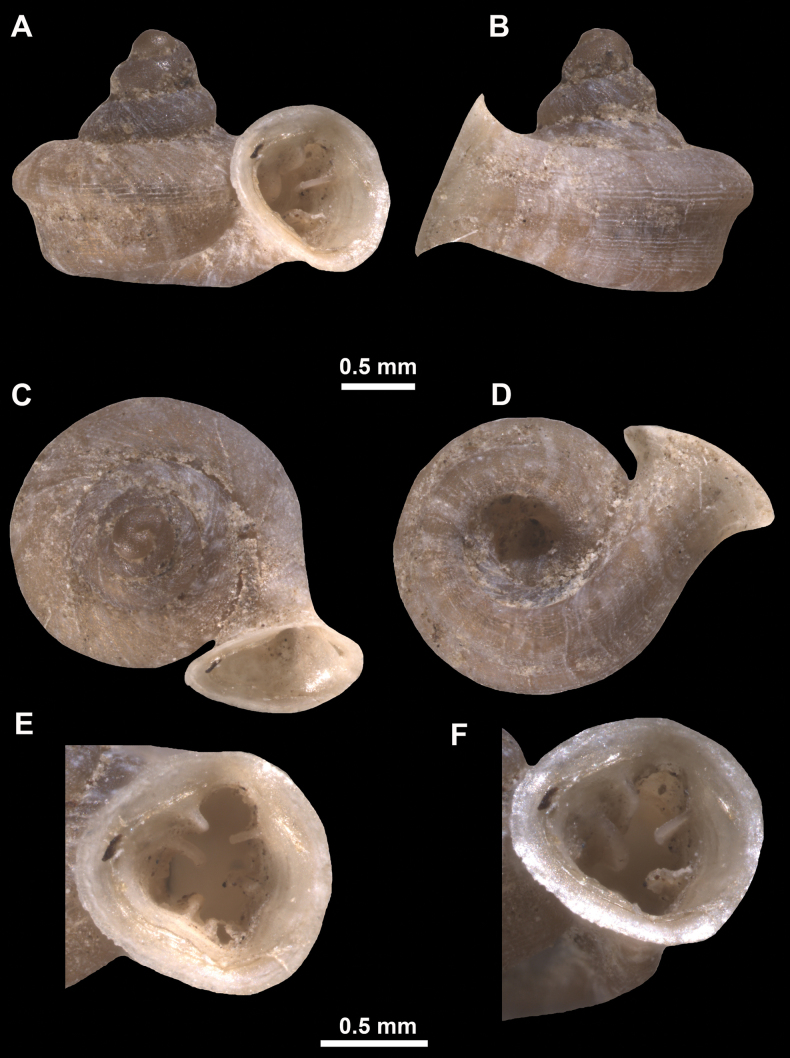
*Hypselostomaeveretti*, syntype of *H.dohertyi* (SMF 4586) **A–D** shell **E, F** enlarged apertural views.

Figs 111T , 136 , 137 , 140

Hypselostoma everetti Smith, 1896: 148, Plate 10, fig. 9–9b.
Hypselostoma dohertyi Fulton, 1899: 215, pl. 11, fig. 17. syn. nov.
Gyliauchen everetti — Pilsbry 1917: 218–219, pl. 37, figs 4, 6, 10.
Gyliauchen dohertyi — Pilsbry 1917: 219–220, pl. 37, figs 7–9.
Gyliotrachela everetti — van Benthem Jutting 1950: 45; Vermeulen and Whitten 1998: 80–81, fig. 64.
Gyliotrachela dohertyi — van Benthem Jutting 1950: 45; Zilch 1984: 165.

##### Type material examined.

**Indonesia** • syntype of *H.everetti*; NHMUK 1896.5.17.1–6; syntype of *H.dohertyi*; SMF 4586/1.

##### Additional material examined.

**Indonesia** • 2 shells; Kalao island, Celebes; SMF 227446 • 2 shells; Kalao island, T. H. Aldrich coll.; UF 00112280. • 1 shell; Timor island, 1 km W. Baucau; 18. Aug. 1971; F. G. Thompson leg.; locality code FGT-1729; UF 210107.

##### Type localities.

“Kalao island”, Indonesia (*H.everetti*); “Tenimber I”, (= Tanimbar islands, Indonesia) (*H.dohertyi*).

##### Differential diagnosis.

This species differs from other congeners by the presence of strong keel (almost like a shoulder) positioned slightly above the centre of the periphery and a very wide umbilicus.

##### Distribution.

This species is known from Timor island, Kalao island, Sulawesi, Java, Bali, Lesser Sunda islands and Tanimbar islands (latter is the type locality of *H.dohertyi*).

##### Remarks.

During examination of the type material, we found no clear differences between *H.everetti* and *H.dohertyi*. The differences mentioned by Fulton (1899) in the original description include the narrower shell, less detached aperture, “not perspectively umbilicated” shell and that the shell is “more closely coiled below”. The umbilicus appears very slightly narrower in *H.dohertyi* but this falls within the intraspecific variability. Other characters mentioned are also very similar between the two species and also fall within the intraspecific variability, which is especially true for the level of the detachment of the last whorl.

#### ﻿ Hypselostoma fruhstorferi

Taxon classificationAnimaliaStylommatophoraHypselostomatidae

Möllendorff, 1897

CB7BF46F-98FE-5A10-89D4-556B076B7402

**Figure 138.**
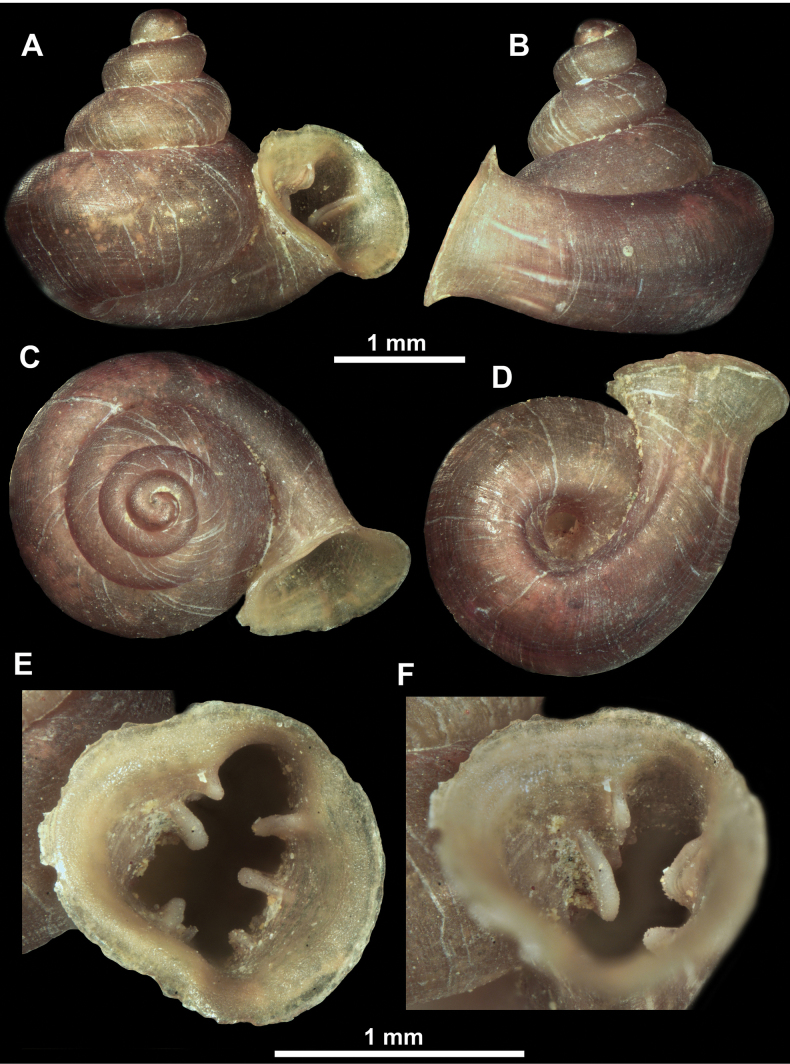
*Hypselostomafruhstorferi*, paratype of *G.concreta* (RMNH Moll.137155) **A–D** shell **E, F** enlarged apertural views.

**Figure 139.**
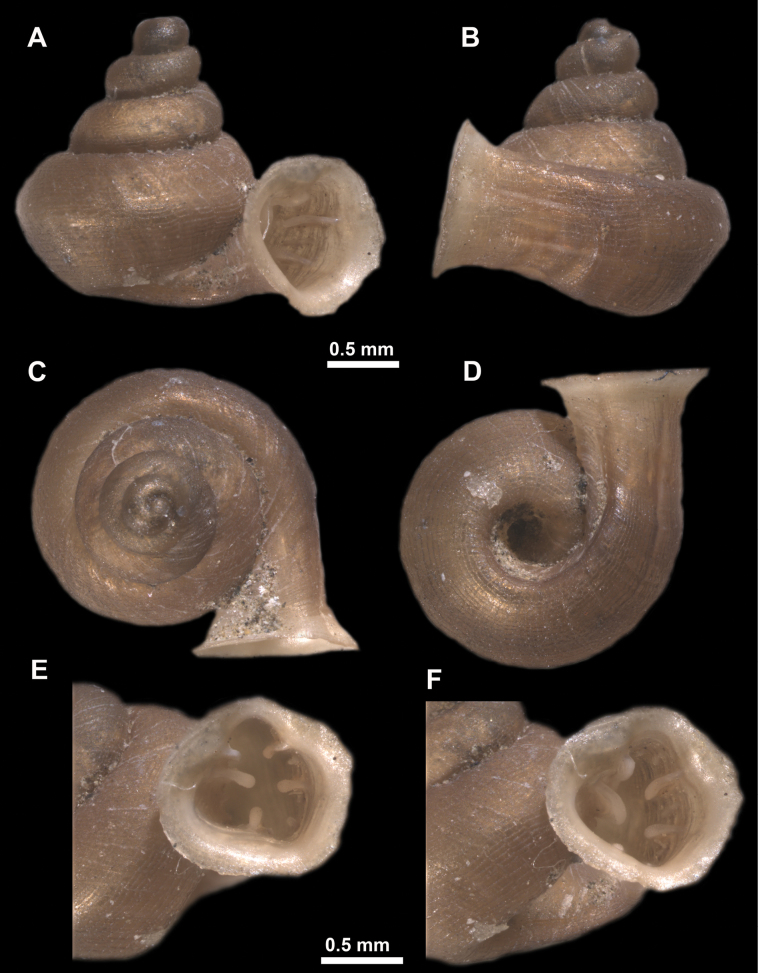
*Hypselostomafruhstorferi*, lectotype (SMF 4580) **A–D** shell **E, F** enlarged apertural views.

**Figure 140.**
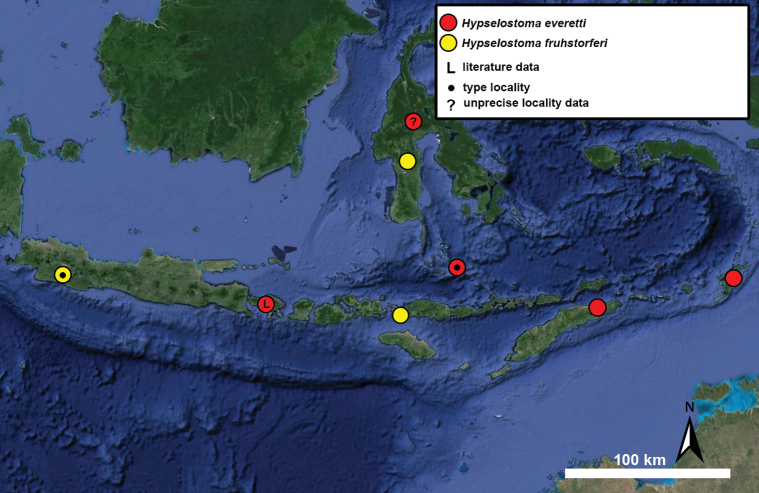
Distribution map of *H.everetti* and *H.fruhstorferi*.

Figs 111U , 138 , 139 , 140

Hypselostoma fruhstorferi Möllendorff, 1897: 70–71.
Gyliauchen fruhstorferi — Pilsbry 1917: 217, pl. 37, figs 1–3; van Benthem Jutting 1950: 46.
Gyliotrachela concreta van Benthem Jutting, 1949a: 64–65, fig. 1. syn. nov.
Gyliotrachela concreta — van Benthem Jutting 1950: 44.
Gyliotrachela fruhstorferi — van Benthem Jutting 1952: 318; Zilch 1984: 165.

##### Type material examined.

**Indonesia** • lectotype of *H.fruhstorferi*; SMF 4580 • 5 paralectotypes of *H.fruhstorferi*; SMF 4581 • 16 paratypes of *G.concreta*; from the type locality; Sept. 1948; G. A. Tammes-Bolt leg.; RMNH.Moll.137155.

##### Additional material examined.

**Indonesia** • 5 shells; Sulawesi, South-Sulawesi, Lemo SE of Rantepao; 700 m a.s.l.; May 1995; W. J. M. Maassen leg.; coll. PGB • 2 shells; Lesser Sunda islands, Rintja island, ca 1 mile S Rintja village, mouth of cave; Apr. 1994; W. Auffenberg leg.; UF 220736.

##### Type localities.

“Java” (Djampang, Java, Indonesia) (*H.fruhstorferi*); “Sulawesi, Main road from Makalé to Kalossi, South Celebes, 700–800 m alt.” (*G.concreta*).

##### Differential diagnosis.

This species is probably most similar to the geographically distant *H.khaowongense* and for differences, see under that species.

##### Distribution.

This species in known from Lesser Sunda islands, Java and Sulawesi islands, Indonesia.

##### Remarks.

Slightly variable smaller barriers. Some specimens of *G.concreta* had two and some three barriers between the lower palatal and columellar one. Interpalatal plica can be well developed or weaker.

We observed no differences between the type specimens of *G.concreta* and *H.fruhstorferi*. Van Benthem Jutting (1949a) compared the newly described *G.concreta* with *H.fruhstorferi* but her decision was based on variable characters such as the level of detachment of the last whorl, broadness of the last whorl (which is, by us, not observed as different between the types) and level of keel development (namely slightly weaker in *G.concreta*, but this difference is very slight). We treat all these differences as intraspecific variability.

#### ﻿ Hypselostoma fungus

Taxon classificationAnimaliaStylommatophoraHypselostomatidae

Gojšina, Hunyadi & Páll-Gergely sp. nov.

F3E4D81E-73B5-5F01-BAD8-C70631BE810A

https://zoobank.org/BBF832C9-2A1C-4DE8-B133-3C886EBFF38E

**Figure 141.**
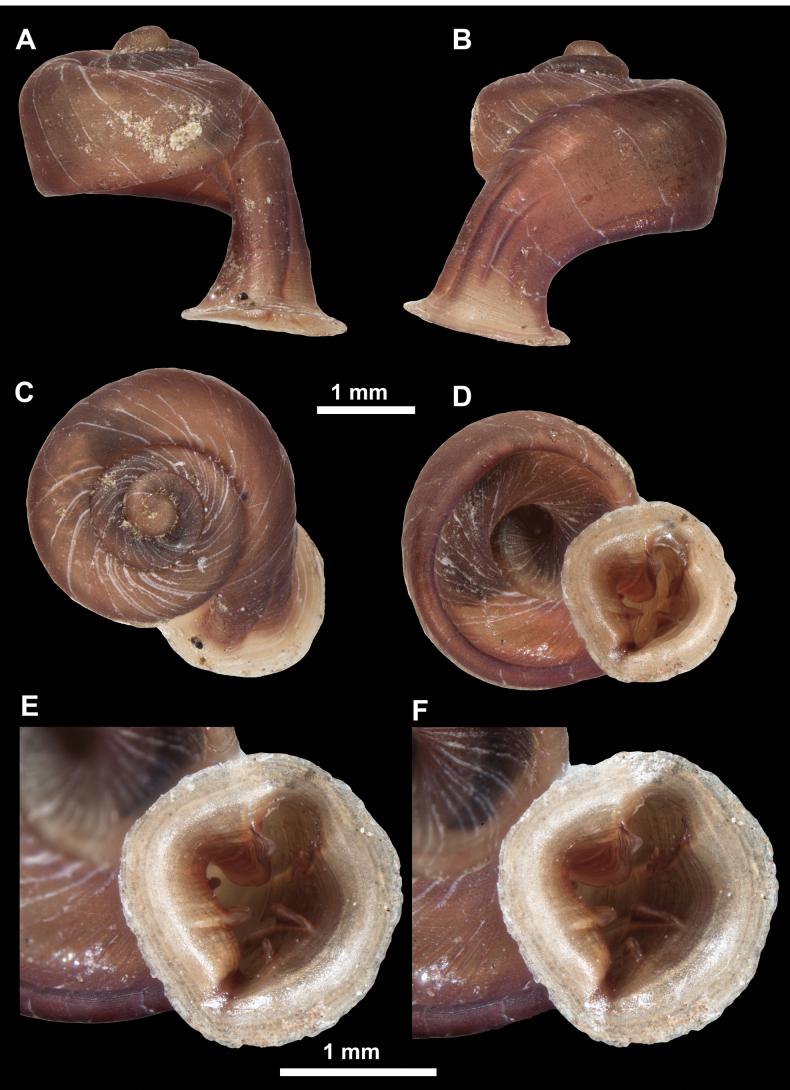
*Hypselostomafungus* Gojšina, Hunyadi & Páll-Gergely, sp. nov., holotype (CUMZ 14453) **A–D** shell **E, F** enlarged apertural views.

**Figure 142.**
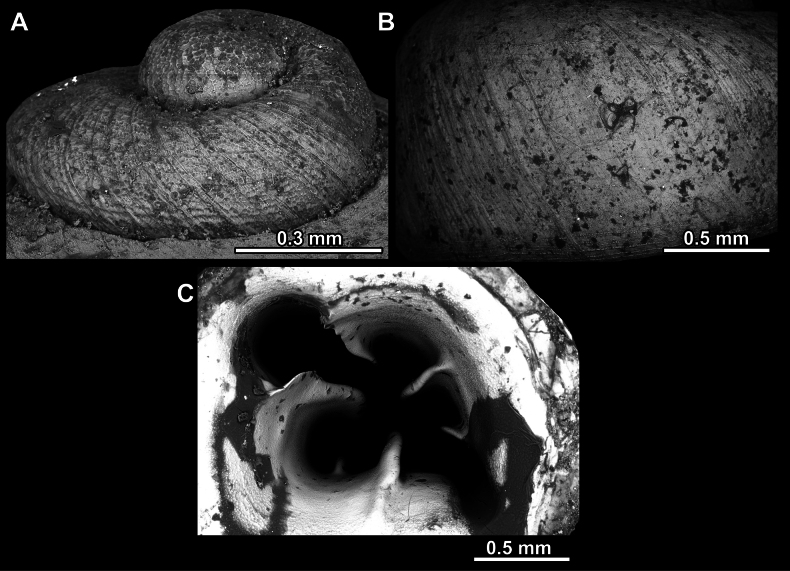
SEM imaging of *Hypselostomafungus* Gojšina, Hunyadi & Páll-Gergely, sp. nov., holotype (CUMZ 14453) **A** protoconch surface **B** teleoconch surface **C** enlarged apertural view.

Figs 111V , 141 , 142 , 177

##### Material examined.

***Holotype*. Cambodia** • 1 shell (SH2: 2.94 mm; SW: 3.09 mm); Steung Treng Province, 36.3 km northwest and 5 km north from Stung Treng Mekong Bridge, Phnom Chhnok; 13°46.573'N, 105°44.878'E; 120 m a.s.l.; 25 Oct. 2023, A. Hunyadi & J.U. Otani leg.; CUMZ 14453.

***Paratypes*. Cambodia** • 219 shells; same data as for holotype; coll. HA • 1 shell; same data as for holotype; coll. VG.

##### Additional material examined.

**Cambodia** • 3 shells (broken, not paratypes); same data as for holotype; coll. HA.

##### Type locality.

Steung Treng Province, 36.3 km northwest and 5 km north from Stung Treng Mekong Bridge, Phnom Chhnok; 13°46.573'N, 105°44.878'E; 120 m a.s.l.

##### Diagnosis.

*Hypselostoma* species with a large last whorl (previous whorls sunken into it) and spirally striated teleoconch. Last whorl strongly detached from the penultimate and descending at almost 80 ° compared to the shell axis. Aperture equipped with four main barriers (angulo-parietal, upper palatal, lower palatal and columellar) and some smaller ones. Basal furrow present. Umbilicus extremely wide, almost as wide as the whole shell.

##### Description.

Shell depressed-conical, brown, and not glossy, consisting of 3.5–4 whorls separated by a deep suture. Protoconch rounded, roughly pitted and consisting of one whorl. Initial teleoconch whorls bluntly keeled and sunken into the last whorl (but sometimes they appear more elevated). Last whorl contributing to almost 100% of the shell height. It is extending obliquely around the previous whorls and is strongly shouldered both on its upper and lower sides, which gives the shell a unique appearance. Surface of the teleoconch is irregularly, very finely and densely spirally striated. Whitish radial streaks are numerous and present on all teleoconch whorls but unevenly spaced (rather randomly positioned). Last whorl is strongly detached from the penultimate and strongly descending (~ 80 ° compared to the shell axis). This makes the aperture profile almost perpendicular to the shell axis. The so called “trumpet” is quite broad (similar to e.g., *H.chaunosalpinx*). Peristome is dirty white (or light brown), thick and strongly expanded, not reflected. Aperture is equipped with four main barriers (angulo-parietal, upper palatal, lower palatal and columellar) and additional smaller ones. The parietal part of the angulo-parietal lamella is strong and very high (blade-like), but the angular is very short and pointed towards the palatal side. Upper palatal plica is directed towards the lower palatal plica but is weaker. Lower palatal plica is long and low in its inner part and much higher in its outer part (closer to the peristome). This plica is similar to the columellar lamella but more slender. Basal (?) plica is situated closer to the lower palatal. It is not clear whether this plica actually represents the infrapalatal or the basal. In the region where usually the basal plica is present, there is a deep basal furrow which is also rarely encountered in this group (similar structure is also present in *H.torta* sp. nov.). This furrow is stretching all the way to the expanding peristome. Columellar lamella almost horizontal, thick, and long. A distinct infraparietal lamella is present (sometimes absent) between the columellar and parietal lamellae. Of all these barriers, only the angulo-parietal and columellar lamellae are reaching the expanding peristome. Surface of all apertural barriers is finely granulated. Sinulus narrow, well separated from the rest of the aperture. Umbilicus extremely wide (almost as wide as the whole shell). The lower edge of the last whorl (towards the umbilicus) is very strongly ridged (almost forming a cliff-like structure), leaving the walls of the umbilicus very steep (like a playground slide). The walls of the umbilicus are especially densely sculptured with white radial streaks.

##### Differential diagnosis.

See under *H.srakeoense*, *H.pendulum*, *H.torticollis*, and *H.transitans*.

##### Measurements

**(in mm, *n* = 5).** SH1 = 2.83–2.97; SH2 = 1.62–1.85; SW = 3.09–3.85; AH = 1.82–2; AW = 1.77–2.05.

##### Etymology.

This species’ shape resembles a mushroom, which gave its specific epithet (Lat. *fungus*). The detached last whorl resembles a stalk while the rest of the shell resembles a cap. To be used as a noun in apposition.

##### Distribution.

This species is known only from its type locality.

##### Remarks.

The largest, and still unexplored limestone hill in Steung Treng Province (13°47.941'N, 105°43.7671'E) was inaccessible during the collecting efforts in 2023 due to the works of a cement factory. Due to the proximity of this hill to the type locality of *H.fungus* sp. nov., it is possible that this species can be found here as well and be threatened by quarrying.

#### ﻿ Hypselostoma geckophilum

Taxon classificationAnimaliaStylommatophoraHypselostomatidae

Gojšina, Hunyadi & Páll-Gergely sp. nov.

FD90B3CA-36CF-5905-B485-09C7BCA2100D

https://zoobank.org/D5241125-6F0C-43E2-972C-4D4767F07444

**Figure 143.**
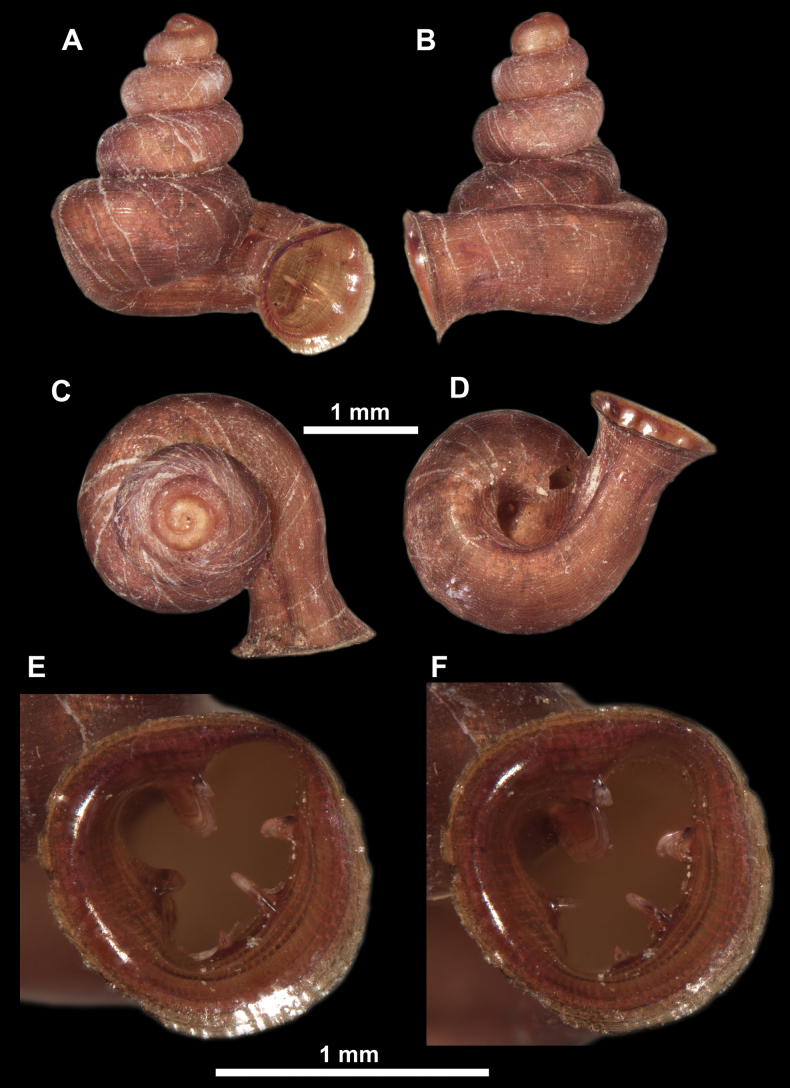
*Hypselostomageckophilum* Gojšina, Hunyadi & Páll-Gergely, sp. nov., holotype (CUMZ 14451) **A–D** shell **E, F** enlarged apertural views.

**Figure 144.**
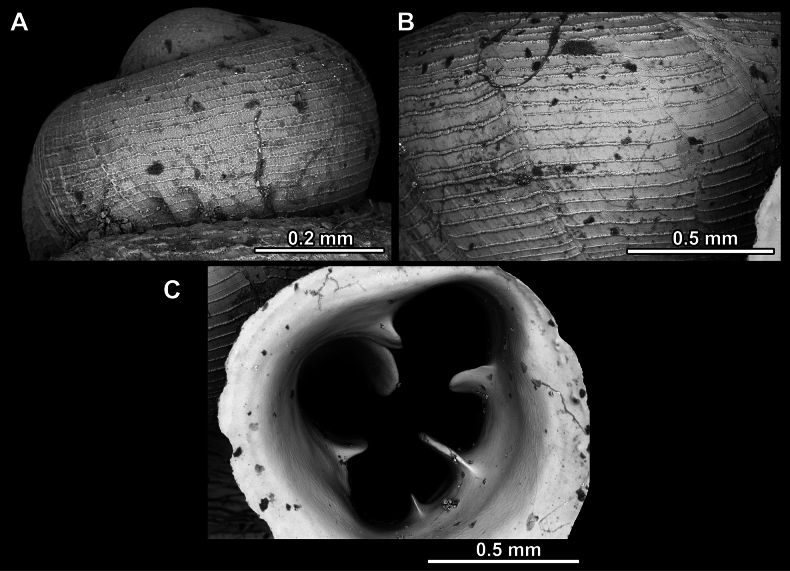
SEM imaging of *Hypselostomageckophilum* Gojšina, Hunyadi & Páll-Gergely, sp. nov., holotype (CUMZ 14451) **A** protoconch surface **B** teleoconch surface **C** enlarged apertural view.

Figs 111W , 143 , 144 , 154

##### Type material.

***Holotype*. Thailand** • 1 shell (SH: 2.92 mm; SW1: 2.74 mm); Chanthaburi Province, Kaeng Hang Maeo district, Tham Khao Wongkot; 12°53.236'N, 101°49.065'E; 60 m a.s.l.; 08 Mar. 2023; A. Hunyadi leg.; CUMZ 14451. ***Paratypes*. Thailand** • 105 shells; same data as for holotype; coll. HA • 1 shell; same data as for holotype; coll. VG.

##### Additional material examined.

**Thailand** • 14 shells (juveniles/ damaged, not paratypes); same data as for holotype; coll. HA.

##### Type locality.

Thailand, Chanthaburi Province, Kaeng Hang Maeo district, Tham Khao Wongkot; 12°53.236'N, 101°49.065'E; 60 m a.s.l.

##### Diagnosis.

Shell concave-conical, last whorl shouldered, previous whorls weakly convex. Teleoconch with raised spiral striae and strong whitish streaks. Last whorl detached from the penultimate, slightly turned downwards. Aperture equipped with five barriers (angulo-parietal, upper palatal, lower palatal, basal, and columellar). Umbilicus initially narrow but suddenly expanding at the last whorl.

##### Description.

Shell concave-conical (due to the strongly expanded last whorl), light brown, weakly glossy, opaque. It is consisting of 4.5–5 step-like, regularly increasing whorls separated by a very deep suture. All whorls except for the last one are rounded, convex. Protoconch slightly lighter than the rest of the shell, spirally striated (~ 25 densely arranged spiral striae) and consisting of ~ 1.25 whorls. Boundary between protoconch and teleoconch not particularly clear but visible as stronger spiral striation and darker surface. Teleoconch densely and strongly spirally striated, especially on the last whorl. Spiral striae slightly almost regularly spaced, leaving the space between the two striae up to the width of three or rarely two striae. Spiral striae get more densely arranged at the lower part of the last whorl than those at the centre of the periphery. There are ~ 33 spiral striae on the last whorl in standard view. Strong and wide radial white streaks cross the spiral striae. These streaks vary in number and are mostly present on the last three whorls. Last whorl shouldered, slightly detached from the penultimate and slightly descending near the aperture (~ 5–10 ° compared to the shell axis). Peristome of the same colour as the rest of the shell, or very slightly lighter. It is expanded and not reflected. Aperture equipped with four main barriers (angulo-parietal, upper palatal, lower palatal and columellar). Angular part of the angulo-parietal lamella reaching the peristome, very small and pointed, leaned towards the upper palatal plica. Parietal part of the angulo-parietal lamella is much stronger and high, blade shaped. Upper palatal plica moderately developed, slightly curved towards the lower palatal and similarly strong. Lower palatal plica high and narrow, making it particularly slender. Columellar lamella strong as the palatal plicae, almost horizontal and very slightly leaned towards the lower palatal plica. Between these main barriers, only a small and weak basal one is additionally observed. Surface of all apertural barriers is smooth. Sinulus wide, not strongly separated from the rest of the aperture. Umbilicus initially very narrow, suddenly expanded at the last whorl, measuring between 1/3 and ¼ of the shell width. A deep groove is running from the columellar side of the peristome towards the inner walls of the umbilicus.

##### Differential diagnosis.

See under *H.platybasis* sp. nov.

##### Measurements

**(in mm, *n* = 5).** SW1 = 2.48–2.74; SW2 = 1.68–1.75; SH = 2.62–2.92; AH = 1.09–1.21; AW = 1.12–1.25.

##### Etymology.

The specific epithet is due to the fact that there were many geckos at its habitat.

##### Distribution.

This species is known only from the type locality.

##### Remarks.

The concrescent angulo-parietal lamella can have the appearance of the typical form (with strong parietal and weaker, pointed angular part) or can have the appearance of only a single, parietal lamella with a very slightly visible swelling which represents the angular part. Infrapalatal lamella can be well visible or very weakly developed.

#### ﻿ Hypselostoma holimanae

Taxon classificationAnimaliaStylommatophoraHypselostomatidae

F.G. Thompson & H.G. Lee, 1988

BBEC67F6-B354-55BF-B0DB-B1AD4A6CD73C

**Figure 145.**
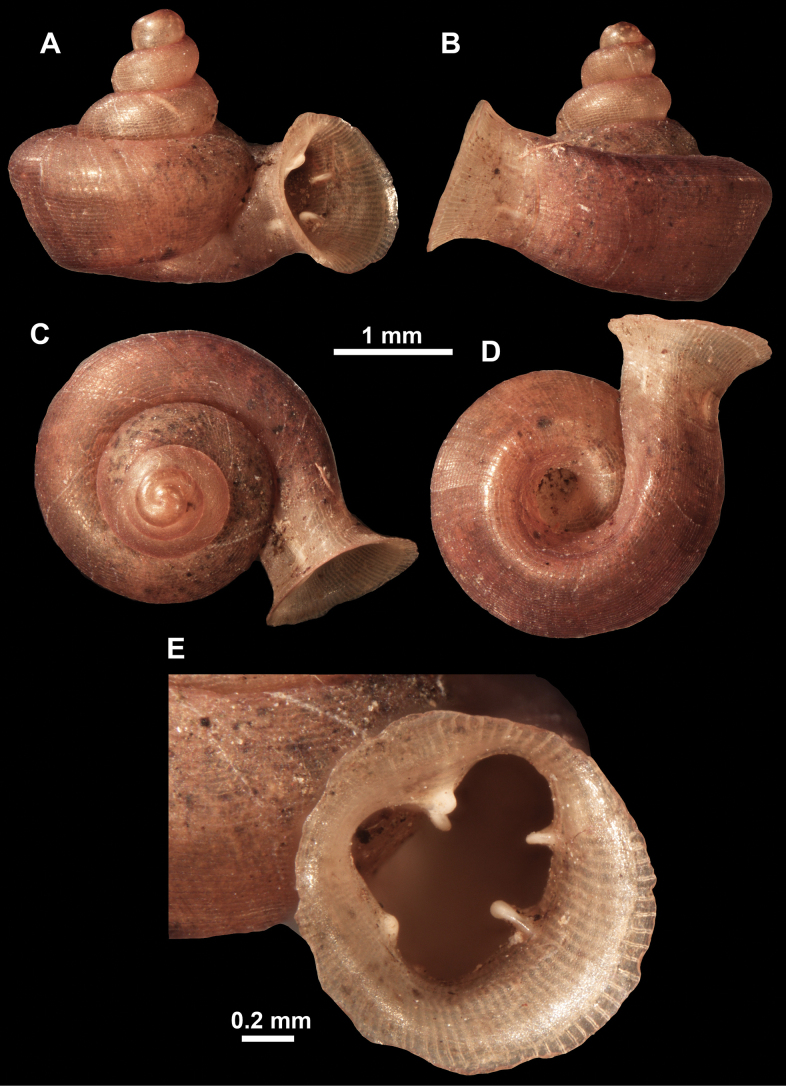
*Hypselostomaholimanae*, holotype (UF 00113427) **A–D** shell **E** enlarged apertural view.

Figs 111X , 145 , 154

Hypselostoma holimanae Thompson & Lee, 1988: 78, figs 1–6.
Hypselostoma holimanae — Thompson and Upatham 1997: 223; Hemmen and Hemmen 2001: 41; Tongkerd et al. 2004: 143, fig. 2; Panha and Burch 2008: 92–93, fig. 79; Dumrongrojwattana and Wongkamhaeng 2024: 324, fig. 8.

##### Type material examined.

**Thailand** • holotype; UF 00113427 • 14 paratypes; from the type locality; UF 00113483/1; UF 00113428/13.

##### Additional material examined.

**Thailand** • 10 shells; Kanchanaburi province, 15 km W Kanchanaburi; 14.154°N, 99.291°E; 15 Mar. 1987; S. Holiman leg.; UF 540579.

##### Type locality.

“Thailand, Kanchanaburi province, small limestone range on the west border of Kanchanaburi Agricultural College, about 15 km west of Kanchanaburi”.

##### Differential diagnosis.

See under *H.khaowongense*.

##### Distribution.

This species is known only from the type locality.

#### ﻿ Hypselostoma insularum

Taxon classificationAnimaliaStylommatophoraHypselostomatidae

Pilsbry, 1908

54099BE0-415F-5295-8A15-FEBE8D8920BB

**Figure 146.**
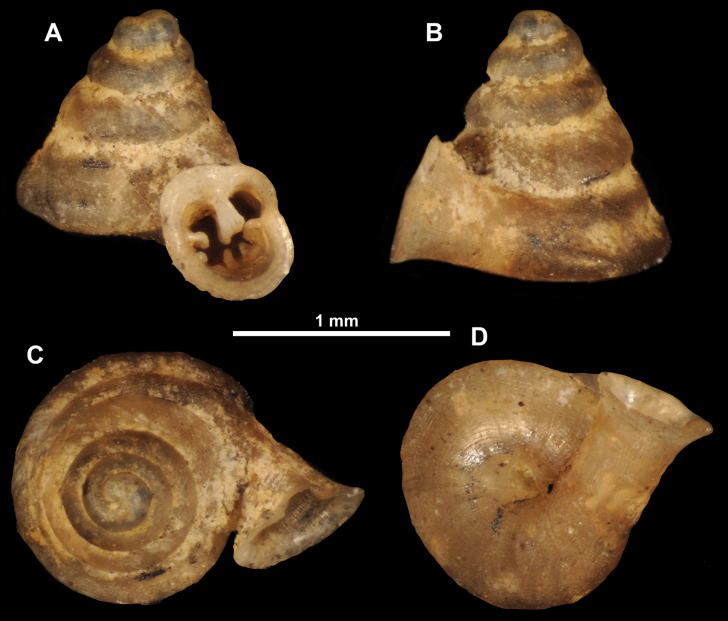
*Hypselostomainsularum*, lectotype (ANSP 95252) **A–D** shell.

**Figure 147.**
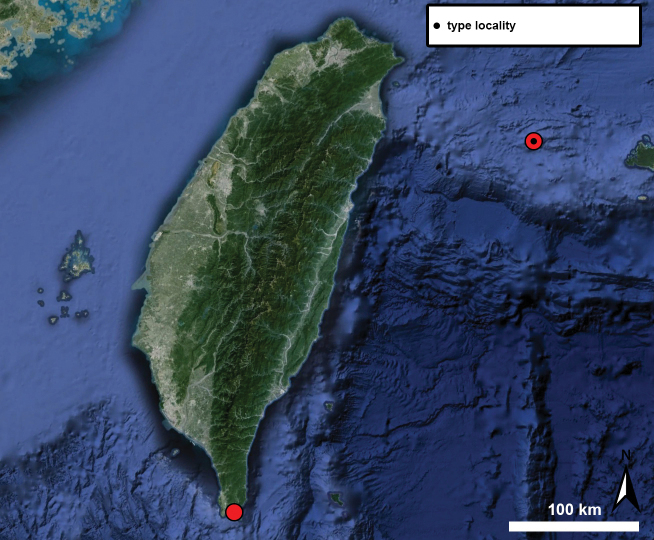
Distribution map of *H.insularum*.

Figs 111Y , 146 , 147

Hypselostoma insularum Pilsbry, 1908: 41, fig. 2.
Hypselostoma insularum — Pilsbry 1917: 182–183, pl. 32, figs 1–4, 6; van Benthem Jutting 1950: 41.
Hypselostoma kentingensis Hwang, 2014: 30–32, fig. 2. syn. nov.

##### Type material examined.

**Japan** • lectotype of *H.insularum*; ANSP 95252.

##### Type localities.

“Yonakunijima, Ryuyku”, Japan (*H.insularum*); “TAIWAN: Kenting Tropical Botanical Garden, Kenting, Hengchun, Pingdung, 21°58'04.5"N 120°48'55.3"E, alt. 295 m” (*H.kentingensis*).

##### Differential diagnosis.

This species is unique by its shell shape which resembles a pyramid (due to the sharp keel located at the base of the last whorl and moderately deep suture). All apertural barriers are strong but this is especially true for the angulo-parietal lamella.

##### Distribution.

This species is known from the type locality and Kenting Tropical Botanical Garden, Kenting, Hengchun, Pingdung, Taiwan (21°58'04.5"N, 120°48'55.3"E). The latter is the type locality of *H.kentingensis*.

##### Remarks.

*Hypselostomakentingensis* is considered a junior synonym of this species since no major differences were observed between the two. All differences mentioned by Hwang (2014) in the original description are considered intraspecific variability. The statement in Hwang (2014) that *H.kentingensis* is smaller is not correct given the fact that the dimensions of the two species largely overlap (see Pilsbry 1917).

#### ﻿ Hypselostoma iunior

Taxon classificationAnimaliaStylommatophoraHypselostomatidae

Gojšina & Páll-Gergely sp. nov.

AF42A8DD-D596-56FD-A549-09F2206CF774

https://zoobank.org/273B0FAF-6025-4960-A806-8F4A90BB07F1

**Figure 148.**
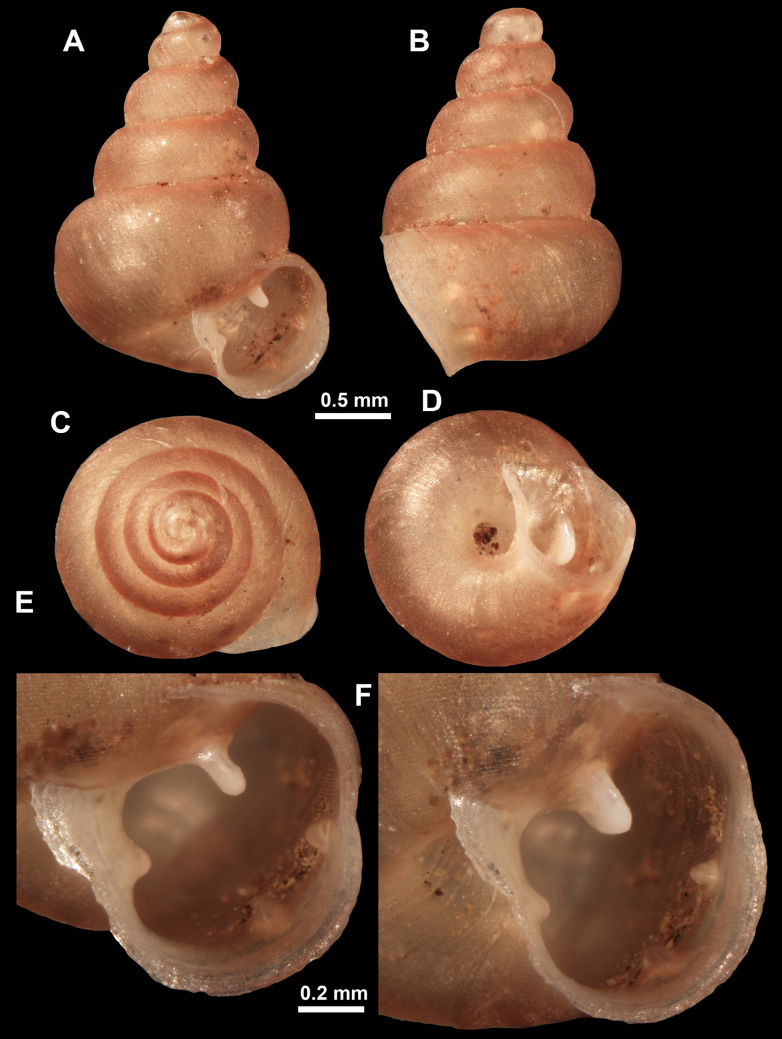
*Hypselostomaiunior* Gojšina & Páll-Gergely, sp. nov., holotype (UF 347376) **A–D** shell **E, F** enlarged apertural views.

**Figure 149.**
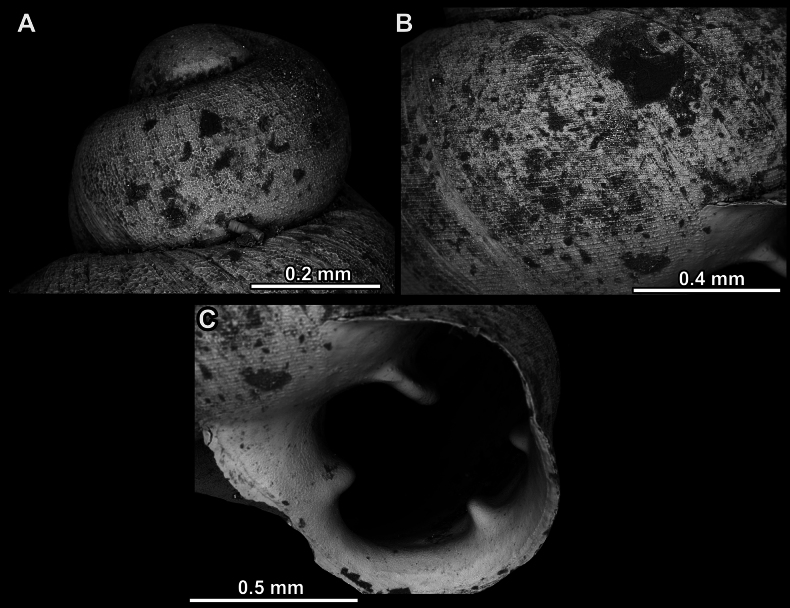
SEM imaging of *Hypselostomaiunior* Gojšina & Páll-Gergely, sp. nov., holotype (UF 347376) **A** protoconch surface **B** teleoconch surface **C** enlarged apertural view.

Figs 111Z , 148 , 149 , 154

##### Type material.

***Holotype*. Thailand** • 1 shell (SH: 2.44 mm, SW: 1.70 mm); Nan Province, Pha Tup cave, Ban Pha Tup, 12 km N of Nan; 18°51′0″N, 100°44′9″E; 260 m a.s.l.; 29 Apr. 1988; F.G. Thompson leg.; UF 347376. ***Paratypes*. Thailand** • 1 shell; same data as for holotype; CUMZ 14454 • 3 shells; same data as for holotype; UF 591327 • 3 shells; Nan Province, Ban Pha Sing, 16 km N of Nan; 18°52′26″N, 100°45′0″E; 270 m a.s.l.; 28 Apr. 1988; F.G. Thompson leg.; locality code FGT-4349, UF 347358.

##### Additional material examined.

**Thailand** • 7 shells (juveniles, not paratypes); same data as for holotype; UF 583729.

##### Type locality.

Thailand, Nan Province, Pha Tup cave, Ban Pha Tun, 12 km N of Nan; 18°51′0″N, 100°44′9″E; 260 m a.s.l.

##### Diagnosis.

*Hypselostoma* with very dense spiral striation on the teleoconch. Apertural barriers very weak and four (parietal, upper palatal, lower palatal and columellar). Umbilicus narrow, widening at the last whorl.

##### Description.

Shell conical, light yellow to brown, consisting of 5–5.5 convex, rounded whorls separated by a deep suture. Protoconch slightly lighter than the rest of the shell, consisting of ~ 1.5 whorls, densely spirally striated (striae clearer terminally). Teleoconch very finely, densely spirally striated crossed by stronger and much coarser, irregularly spaced radial growth lines. Last whorl rounded to slightly shouldered, adnate to the penultimate and slightly to moderately descending (~ 25–35 ° compared to the shell axis) making the aperture profile prosocline to the shell axis. Peristome white but not much expanded, not reflected. It is only particularly expanded on the columellar side where it forms a strong margin which is, however, not covering the umbilicus. The peristome is discontinuous on the parietal side where it is completely merged with the penultimate whorl. Aperture almost as wide as high. Apertural barriers few and much weaker than in the majority of congeners. There are four barriers: parietal, upper palatal, lower palatal and columellar. Parietal lamella is the strongest in the aperture but still somehow weak, moderately high and curved towards the palatal side. Two palatal plicae (upper and lower palatal) are very weak, like small thickenings. Columellar lamella also weak and shares the same appearance as the palatal plicae but situated slightly closer to the peristome edge. None of the apertural barriers are reaching the thin peristome. Surface of all apertural barriers is almost smooth but very finely granulated. Sinulus wide and not strongly isolated from the rest of the aperture. Umbilicus moderately wide, measuring 1/5 of the shell width, initially narrow and suddenly widening at the last whorl (which gives it a slightly elongated appearance).

##### Differential diagnosis.

This species is similar to *A.eotvosi* with which is also geographically adjacent. It differs by the much larger sizes in *A.eotvosi*, narrower umbilicus and the lack of spiral striation. The shell of *A.eotvosi* is also more pointed, especially near the apex. See also under *H.panhai*.

##### Measurements

**(in mm, *n* = 6).**SH = 2.27–2.48; SW = 1.67–1.72; AH = 0.81–0.92; AW = 0.84–0.96.

##### Etymology.

This species is named *iunior* for the very weak teeth and weakly expanded peristome which is common in subadult hypselostomatid specimens.

##### Distribution.

This species is known only from its type locality.

##### Remarks.

Since the most similar species (*H.panhai*) belongs to the genus *Hypselostoma* (as shown in a phylogenetic study by Tongkerd et al. 2004), this species is also included in this genus even though it fits in the diagnosis of *Anauchen*.

#### ﻿ Hypselostoma khaochakan

Taxon classificationAnimaliaStylommatophoraHypselostomatidae

(Panha & J. B. Burch, 2003) comb. nov.

61815C3D-F190-5356-966A-2C30D2E9D12C

Gyliotrachela khaochakan Panha & Burch in Burch et al. 2003: 138–143, figs 4–5.
Gyliotrachela srirachaensis Panha & Burch in Panha et al. 2004: 69–70, fig. 8. syn. nov.
Gyliotrachela khaochakan — Tongkerd et al. 2004: 143, fig. 2; Panha and Burch 2008: 69–70, fig. 60; Dumrongrojwattana and Wongkamhaeng 2024: 323, fig. 8.
Gyliotrachela srirachaensis — Panha and Burch 2008: 81, fig. 70; Dumrongrojwattana and Wongkamhaeng 2024: 324, fig. 8.

##### Type material examined.

**Thailand** • 2 paratypes of *H.khaochakan*; from the type locality; SMF 331445 • 1 paratype of *H.khaochakan*; from the type locality; CUMZ 44041 • 1 paratype of *G.srirachaensis*; from the type locality; S. Panha leg.; CUMZ ver. 44008.

##### Additional material examined.

4 shells; Sa Kaeo Province, Khao Chakan district, vicinity of Wat Tham Khao Chakan; 13°39.668'N 102°05.195'E; 80 m a.s.l.; 05 Mar. 2023, A. Hunyadi & J.U. Otani leg.; coll. HA.

##### Type localities.

“Chakan Mountain, east of Khaochakan Temple, Khaochakan District, Srakeaw Province, 13°39'17"N, 102°5'12"E, 70 meters elevation, Thailand” (*H.khaochakan*); “Sichang Island, Sriracha, Chonburi Province, 13°20'05"N, 100°55'24"E, 70 meters elevation” (*H.srirachaensis*).

##### Differential diagnosis.

This species differs from *H.chedi* by the separated angular and parietal lamellae. See also under *H.smokon*.

##### Distribution.

This species is, apart from the type locality, known also from Sichang Island (type locality of *H.srirachaensis*).

**Figure 150.**
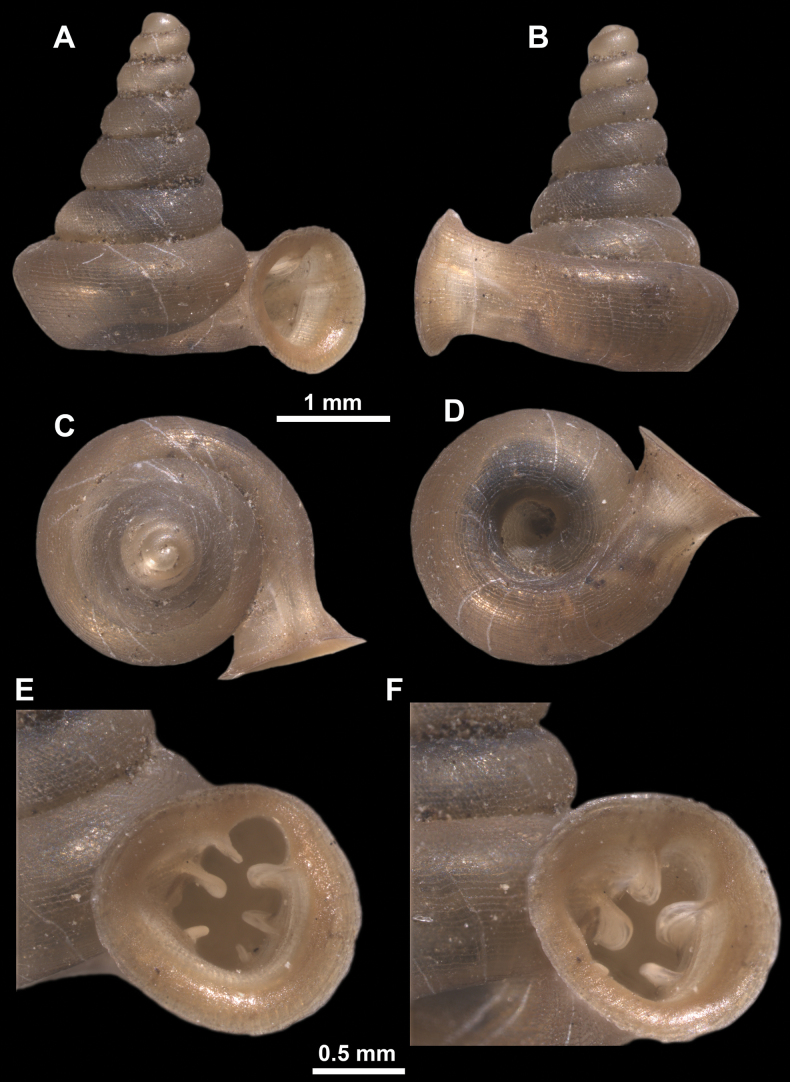
*Hypselostomakhaochakan*, paratype (SMF 331445) **A–D** shell E, **F** enlarged apertural views.

##### Remarks.

*Gyliotrachelasrirachaensis* is a junior synonym of *H.khaochakan* since we could not find any morphological differences. The two species were not compared in the original description of *G.srirachaensis*.

**Figure 151.**
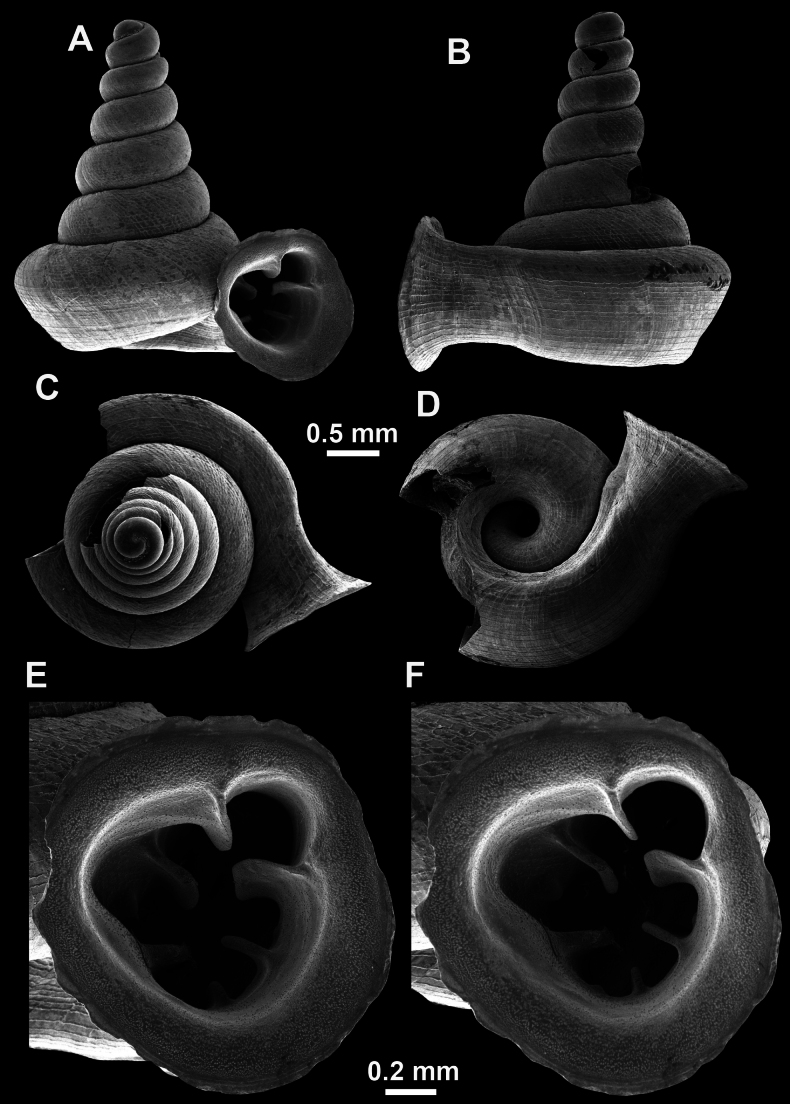
*Hypselostomakhaochakan*, paratype (CUMZ 44041) **A–D** shell E, **F** enlarged apertural views.

**Figure 152.**
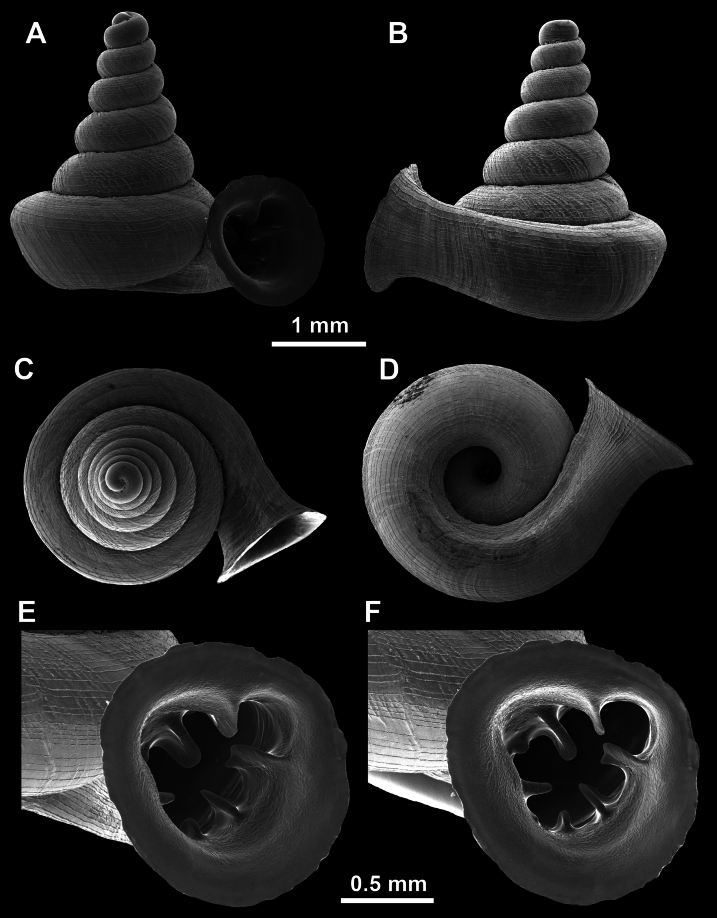
*Hypselostomakhaochakan*, paratype of *G.srirachaensis* (CUMZ ver. 44008) **A–D** shell **E, F** enlarged apertural views.

#### ﻿ Hypselostoma khaochongpran

Taxon classificationAnimaliaStylommatophoraHypselostomatidae

(Panha & J. B. Burch, 2002)

9D27F6D2-C6B2-533B-BAFB-726332D252B5

Anauchen khaochongpran Panha & Burch, 2002e: 22, fig. 3.
Hypselostoma khaochongpran — Panha and Burch 2008: 94, fig. 80; Dumrongrojwattana and Wongkamhaeng 2024: 324, fig. 8.

##### Type material examined.

**Thailand** • holotype; 1999; S. Panha leg; CUMZ 4022.

##### Type locality.

“Khaochongpran, Ratchaburi province, 13°35′38″N, 99°40′0″E, 60 meters elevation” (Thailand).

##### Differential diagnosis.

This species is strikingly similar to *H.cucumense* but differs by the much weaker apertural barriers and less distinctly ascending last whorl.

**Figure 153.**
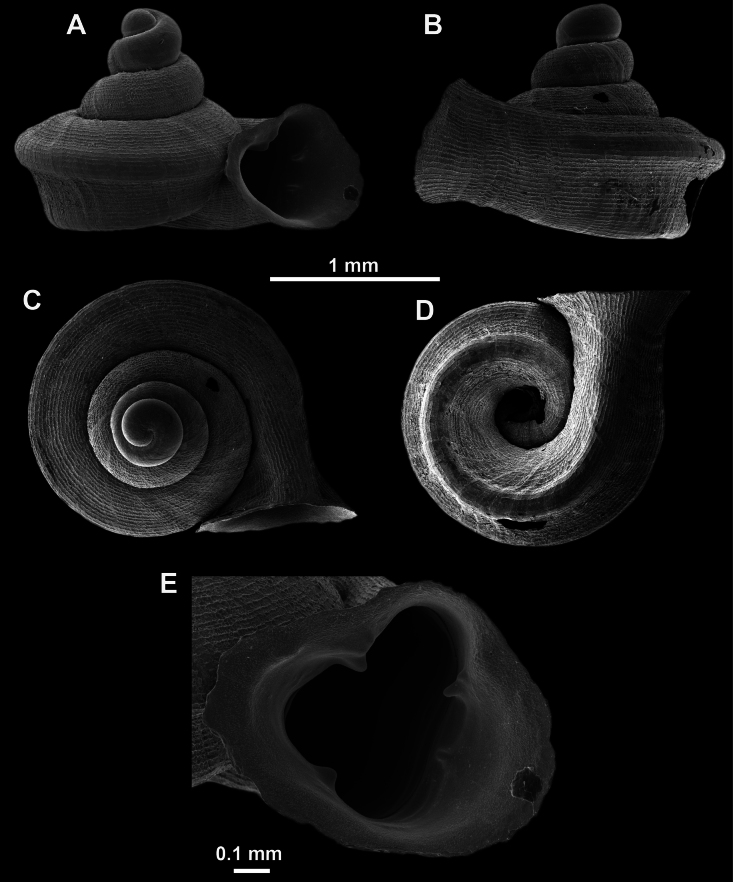
*Hypselostomakhaochongpran*, holotype (CUMZ 4022) **A–D** shell **E** enlarged apertural view.

**Figure 154.**
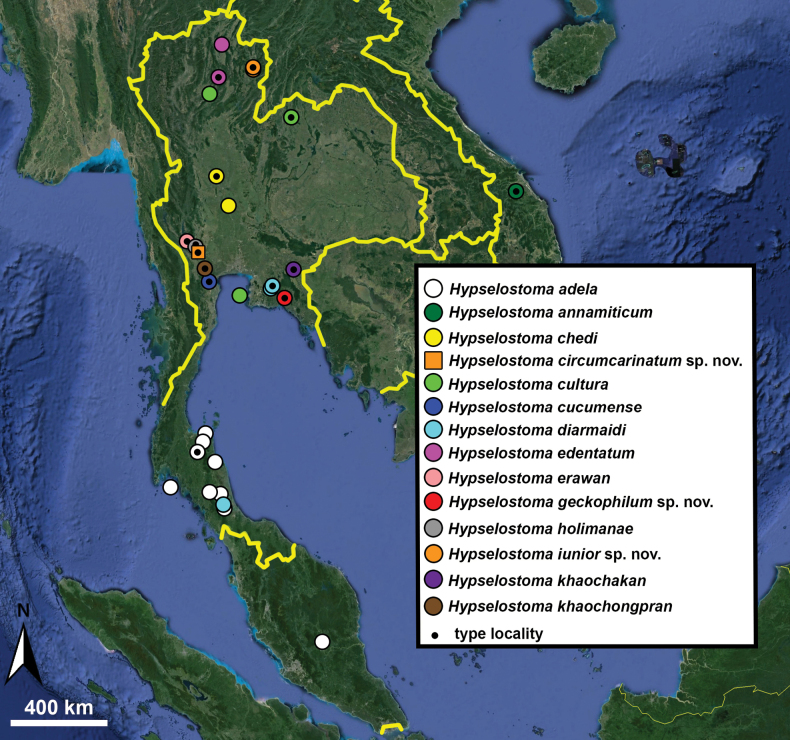
Distribution map of some species belonging to the *H.bensonianum* group.

##### Distribution.

This species is known only from the type locality.

#### ﻿ Hypselostoma khaowongense

Taxon classificationAnimaliaStylommatophoraHypselostomatidae

Panha, 1998

5FE53108-D508-5895-9AEB-2A12B43BEA2A

Hypselostoma khaowongensis Panha, 1998b: 66, fig. 4.
Hypselostoma khaowongensis — Hemmen and Hemmen 2001: 41.
Gyliotrachela saraburiensis Panha & Burch in Burch et al. 2003: 143–151, figs 6–7. syn. nov.
Gyliotrachela muangon Panha & Burch in Panha et al. 2004: 67–68, fig. 7. syn. nov.
Gyliotrachela khaowongensis — Panha and Burch 2008: 71–72, fig. 62; Dumrongrojwattana and Wongkamhaeng 2024: 323, fig. 8.
Gyliotrachela saraburiensis — Tongkerd et al. 2004: 143, fig. 2; Panha and Burch 2008: 78–80, fig. 68; Dumrongrojwattana and Wongkamhaeng 2024: 324, fig. 8.
Gyliotrachela muangon — Panha and Burch 2008: 76–77, fig. 66; Dumrongrojwattana and Wongkamhaeng 2024: 324, fig. 8; Gojšina et al. 2022: 137, fig. 6; Tongkerd et al. 2024: 182–184, figs 11, 13O.

##### Type material examined.

**Thailand** • holotype; S. Panha leg.; CUMZ ver. 001 • paratype of *G.saraburiensis*; from the type locality; CUMZ 44047 • 2 paratypes of *H.khaowongense*; from the type locality; SMF 331439 • 3 paratypes of *G.saraburiensis*; from the type locality; SMF 331446 • 2 paratypes of *G.muangon*; from the type locality; S. Panha leg.; SMF 331459.

##### Additional material examined.

**Thailand-North** • 24 shells; Chiang Rai Province, Chiang Rai ca 5 km west from centre of Wat Phra That Tham Doi Kong Khao; 9°54.820'N, 99°46.642'E; 11 Feb. 2015; A. Hunyadi leg.; coll. HA • 33 shells; Chiang Rai Province, ca 5 km WNW of Chiang Rai, Tham Phra cave by Mae Nam Kok river, limestone rocks near the cave entrance; 19°55.047'N, 99°47.342'E; 420 m a.s.l; Sep. 2007; A. Reischütz leg.; coll. REI • 12 shells; Chiang Mai Province, 5 km northwest from Chiang Dao, vicinity of Wat Tham Chiang Dao; 19°23.628'N, 98°55.711'E; 450 m a.s.l.; 07 Feb. 2015; A. Hunyadi leg.; coll. HA • 13 shells; Chiang Mai Province, Doi Pha Sao Mountain, 3 km W of Ban Prang Mao; 19°25.98'N, 99°3.48'E; 500 m a.s.l.; 19 Jun. 1987; F.G. Thompson leg.; locality code FGT-4338; UF 528706 • 21 shells; Chiang Mai Province, NW side of Doi Pha Sam Sao ravine with Dipterocarp forest, leaf litter; 19°24.45'N, 99°2.9333'E; 800 m a.s.l.; 20 May 1988; F.G. Thompson leg.; locality code FGT-4453; UF 347006 • 59 shells; Chiang Mai Province, NW side of Doi Pha Sam Sao ravine with Dipterocarp forest, leaf litter; 19°24.45'N, 99°2.9333'E; 800 m a.s.l.; 20. May 1988; F. G. Thompson leg.; locality code FGT-4452; UF 346992 • 150 shells; Mae Hong Son Province, 13.9 km NW Pai, along road 1095; 19°23'N, 98°23'E; 780 m a.s.l.; 18 Mar. 1988; K. Auffenberg leg.; locality code KA-0571; UF 345938 • 3 shells; Mae Hong Son Province, Ban Tham Lod Village, 7 km N Soppong on Road 1095; 19°34'N, 98°9'E; 790 m a.s.l.; 18 Mar. 1988; K. Auffenberg leg.; locality code KA-0574; UF 591328 • 20 shells; Mae Hong Son Province, 21 km N Mae Hong Son, Fish Cave; 19°26'N, 97°58'E; 380 m a.s.l.; 22 Mar. 1988; K. Auffenberg leg.; locality code KA-0593; UF 345672 • 40 shells; Mae Hong Son Province, 40 km N of Mae Hong Son; 19°27'N, 97°59'E; 550 m a.s.l.; 22 Mar. 1988; K. Auffenberg leg.; locality code KA-0588; UF 345643 • 1 shell; Mae Hong Son Province, 21 km N Mae Hong Son, Fish Cave; 19°26'N, 97°58'E; 380 m a.s.l.; 22 Mar. 1988; K. Auffenberg leg.; locality code KA-0595A; UF 345702 • 7 shells; Mae Hong Son Province, Mae Hong Son, Road 1095; 19°25'N, 97°58'E; 390 m a.s.l.; 22 Mar. 1988; K. Auffenberg leg.; locality code KA-0595B; UF 591329 • 3 shells; Mae Hong Son Province, Mae Hong Son, Road 1095; 19°25'N, 97°58'E; 390 m a.s.l.; 22 Mar. 1988; K. Auffenberg leg.; locality code KA-0595; UF 345699 • 1 shell; Lampang Province, 6 km SW Mae Pak, limestone hill; 17°57.8833'N, 98°51.1167'E; 600 m a.s.l.; 13 June 1987; F.G. Thompson leg.; locality code FGT-4328; UF 591330 • 11 shells; Lampang Province, limestone dome at pass 11 km SSW of Ban Pang La; 18°27.9667'N, 99°48.3833'E; 610 m a.s.l.; 14 May 1988; F.G. Thompson leg.; locality code FGT-4441; UF 591331 • 2 ethanol preserved shells; same locality data as previous; F.G. Thompson leg.; locality code FGT-4440; UF 346908 • 43 shells; Phrae Province, limestone knoll 4 km N of Ban Nam Rin, deep rocks and leaves; 18°10.1333'N, 99°56.6833'E; 200 m a.s.l.; 16. May 1988; F.G. Thompson leg.; locality code FGT-4444; UF 346931 • 1 shell; Phrae Province, Than Pha Nan Cave, 6 km NE of Rang Kwang; 18°23.1'N, 100°20.7667'E; 300 m a.s.l.; 30 Apr. 1988; F.G. Thompson leg.; locality code FGT-4400; UF 347403 • 2 shells; Phrae Province., 4 km N of Ban Nam Rin valley with limestone ridges, leaf litter at base of boulders; 18°10.1333'N, 99°56.6833'E; 200 m a.s.l.; 16. May 1988; F. G. Thompson leg.; locality code FGT-4445; UF 346935 • 5 ethanol preserved shells; Phrae Province, 11 km W of Phrae, on Road 1023 teak plantation and cut forest; 18°10.6833'N, 100°4.2333'E; 290 m a.s.l.; 16. May 1988; F.G. Thompson leg.; locality code FGT-4446; UF 346967 • 19 shells; same locality data as previous; F.G. Thompson leg.; locality code FGT-4447; UF 591332. **Thailand-Northeast** • 28 shells; Petchabun Province, 24.4 km S. Petchabun on Hwy. 2275, evergreen forest in back of outcrop, leaf litter next to rocks; 16°16'N, 101°10'E; 120 m a.s.l.; 02. May 1988; K. Auffenberg leg.; locality code KA-0693; UF 346096 • 1 shell; Nakhon Ratchasima Province, 3.4 km W of Ban Mu Si; 14.534°N, 101.380°E; 380 m a.s.l.; 05. May 1987; F.G. Thompson leg.; locality code FGT-4241; UF 534868 • 9 shells; Nakhon Ratchasima Province, Pak Chong district, mountain above Wat Thep Phithak Punnaram; 14°36.865'N, 101°15.926'E; 500 m a.s.l.; 04 Mar. 2023; A. Hunyadi leg.; coll. HA. **Thailand-West** • 16 shells; Kanchanaburi Province, Erawan Nat. Park, Erawan Falls Trail; 14°22.310'N, 99°08.699'E; 85 m a.s.l.; 17 Feb. 2015; A. Hunyadi leg.; coll. HA • 10 shells; Petchabun Province, 24.4 km S. Petchabun on Hwy. 2275, evergreen forest in back of outcrop, leaf litter next to rocks; 16°16'N, 101°10'E; 120 m a.s.l.; 02. May 1988; K. Auffenberg leg.; locality code KA-0694; UF 346100. **Thailand-Central** • 10 shells; Phitsanulok Province, Noen Maprang district, Chomphu, Tham Phra Wang Daeng; 16°40.651'N, 100°41.287'E; 02 Mar. 2023; A. Hunyadi, K. Okubo & J.U. Otani leg.; coll. HA • 37 shells; Nakhon Sawan Province, 19.5 km NE intersection of Hwys 11 and 32, limestone outcrop, base of cliff under *Georissa* zone; 15°14'N, 100°16'E (provided coordinates point in Chai Nat Province); 50 m a.s.l.; 02. May 1988; K. Auffenberg leg.; locality code KA-0697; UF 346135 • 1 shell; Nakhon Sawan Province, 19.5 km NE intersection of Hwys 11 and 32; 16°14'N, 100°16'E; 50 m a.s.l.; 02. May 1988; K. Auffenberg leg.; locality code KA-0697; UF 346126 • 1 shell; Lopburi Province, Tha Wung district, Khao Samo Khon, Wat Tham Chang Pueak; 14°54.139'N, 100°30.151'E; 30 m a.s.l.; 03 Mar. 2023; A. Hunyadi leg.; coll. HA • 2 shells; Saraburi Province, behind Phra Phutthabat, limestone hill with scrubby evergreen forest, leaf litter among rocks; 14.733°N, 100.800°E; 50 m a.s.l.; 03. May 1988; K. Auffenberg leg.; locality code KA-0699; UF 508979. **Laos** • 27 shells; Luang Prabang Province, old forest on Phousi Mountain in Luang Prabang, clay, black soil, on and under rocks; 19°53.411'N, 102°08.216'E; 374 m a.s.l.; 13 Nov. 2004; A. Abdou & I. Muratov leg.; MNHN-IM-2012-25387 • 6 shells; Luang Prabang Province, 3.1 km from centre of Nong Khiaw towards Pak Xeng, Tham Pha Toke, vicinity of the cave; 20°33.215'N, 102°37.722'E; 345 m a.s.l.; 05 Oct. 2019; A. Hunyadi, K. Okubo & J.U. Otani leg.; coll. HA • 24 shells; Luang Prabang Province, 12 km from centre of Nong Khiaw towards Pak Xeng, northeast from Ban Huai Lek, left side of the road; 20°32.585'N, 102°41.161'E; 345 m a.s.l.; 05 Oct. 2019; A. Hunyadi, K. Okubo & J.U. Otani leg.; coll. HA. **Myanmar** • 3 shells; Shan State, c. 3.6 km NNE of Hsihseng centre, limestone hill; 20°11′15″N, 97°16′07″E; 1040 m a.s.l.; 07 Oct. 2018; A. Hunyadi, K. Okubo & J.U. Otani leg.; coll. HA • 7 shells; Shan State, 9.5 km from centre of Hsihseng; 20°14.066'N, 97°15.514'E; 1140 m a.s.l.; 07 Oct. 2018; A. Hunyadi, K. Okubo & J.U. Otani leg.; coll. HA • 21 shells; Shan State, 3.6 km north-northeast from centre of Hsihseng; 20°11.248'N, 97°16.114'E; 1040 m a.s.l.; 07 Oct. 2018; A. Hunyadi, K. Okubo & J.U. Otani leg.; coll. HA • 63 shells; Shan State, ca 16 km from centre of Taunggyi centre towards Hopong, 1.5 km north on rd. #4, „Shwe Pyi Aunchonda” monastery; 20°47.263'N, 97°8.239'E;1110 m a.s.l.; 08 Oct. 2018; A. Hunyadi, K. Okubo & J.U. Otani leg.; coll. HA • 57 shells; Shan State, ca 6 km east from centre of Hsihseng; 20°8.002'N, 97°18.024'E; 500 m a.s.l.; 07 Oct. 2018; A. Hunyadi, K. Okubo & J.U. Otani leg.; coll. HA.

##### Type localities.

“A limestone hill near Takli District, Nakornsawan Province, 15°16′52″N, 100°22′38″E, 114 meters elevation”, Thailand (*H.khaowongense*); “East of Tepitak Tamaram Temple, Tepitak Mountain, Muang Saraburi District, Saraburi Province, 14°36'57"N, 101°15'50"E, 510 meters elevation. Thailand” (*G.saraburiensis*); “Muangon Cave, San Kam Pang District, Chiangmai Province, 18°47'11"N, 99°14'16"E, 280 meters elevation” (*G.muangon*).

##### Differential diagnosis.

This species is similar to *H.holimanae* but is clearly different by the separated angular and parietal lamellae (fused in *H.holimanae*) as well as the presence of numerous and variable apertural barriers (only four in *H.holimanae*). *Hypselostomafruhstorferi* has a narrower umbilicus and fewer apertural barriers. This species is also similar to *H.cultura* from which it can be separated by the less prominently shouldered last whorl and a narrower umbilicus.

**Figure 155.**
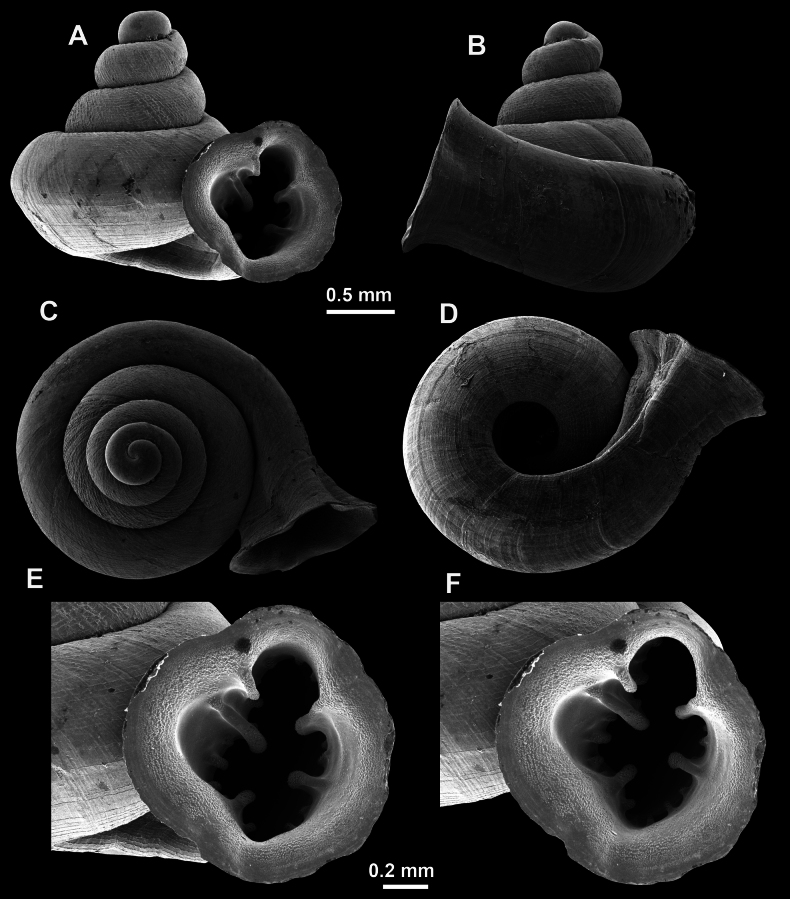
*Hypselostomakhaowongense*, holotype (CUMZ ver. 001) **A–D** shell **E, F** enlarged apertural views.

**Figure 156.**
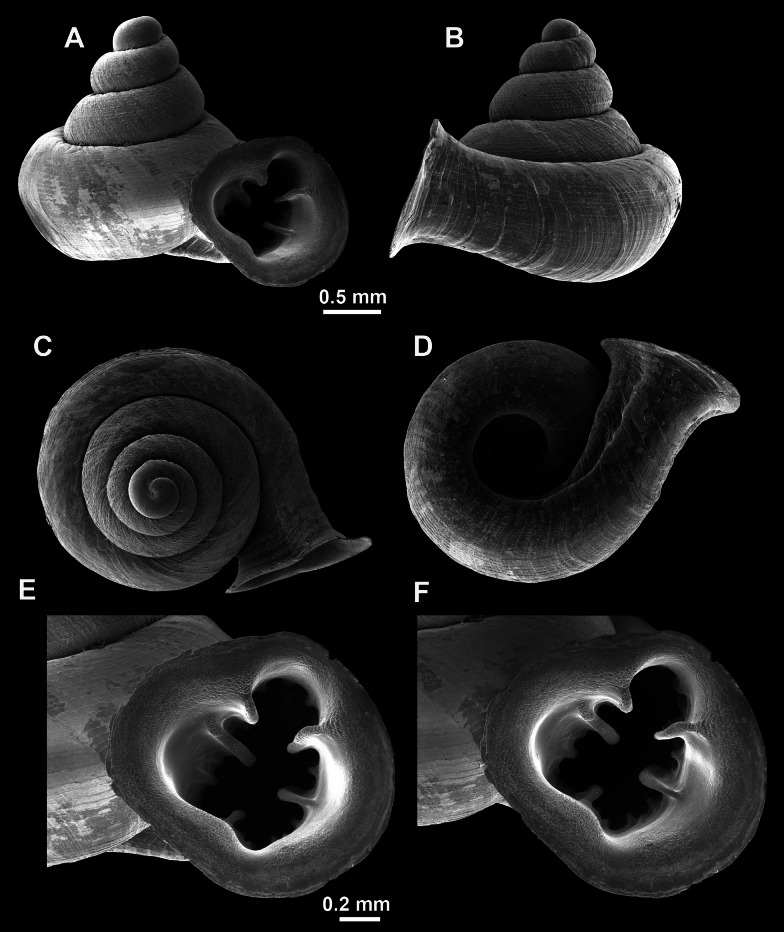
*Hypselostomakhaowongense*, paratype of *G.saraburiensis* (CUMZ 44047) A–**D** shell E, **F** enlarged apertural views.

##### Distribution.

Clearly a widespread species: its distribution stretches from Chiang Rai province in N Thailand to the southernmost known localities in Ratchaburi and Chonburi provinces. Westwards, this species stretches to Taunggyi in Myanmar and the easternmost locality is known from Luang Prabang province in Laos.

##### Remarks.

Examined specimens had the usual main barriers and a larger number of smaller barriers between them of very variable number (usually 2–5 between each of the main barriers). Umbilicus was always wide but sometimes more or less so (specimens from Chiang Mai (Thailand) had the narrowest umbilicus). Last whorl is always at least very slightly keeled, in some specimens more prominent. Spiral striation is present, in some populations hardly observable and in some very rough. During comparison of the type material of *G.saraburiensis* and *G.muangon*, we have not observed any difference between these species and *H.khaowongense*. The former two are not compared with the latter in the respective original descriptions. Because of no difference observed, we treat both *G.saraburiensis* and *G.muangon* as junior synonyms of *H.khaowongense*.

**Figure 157.**
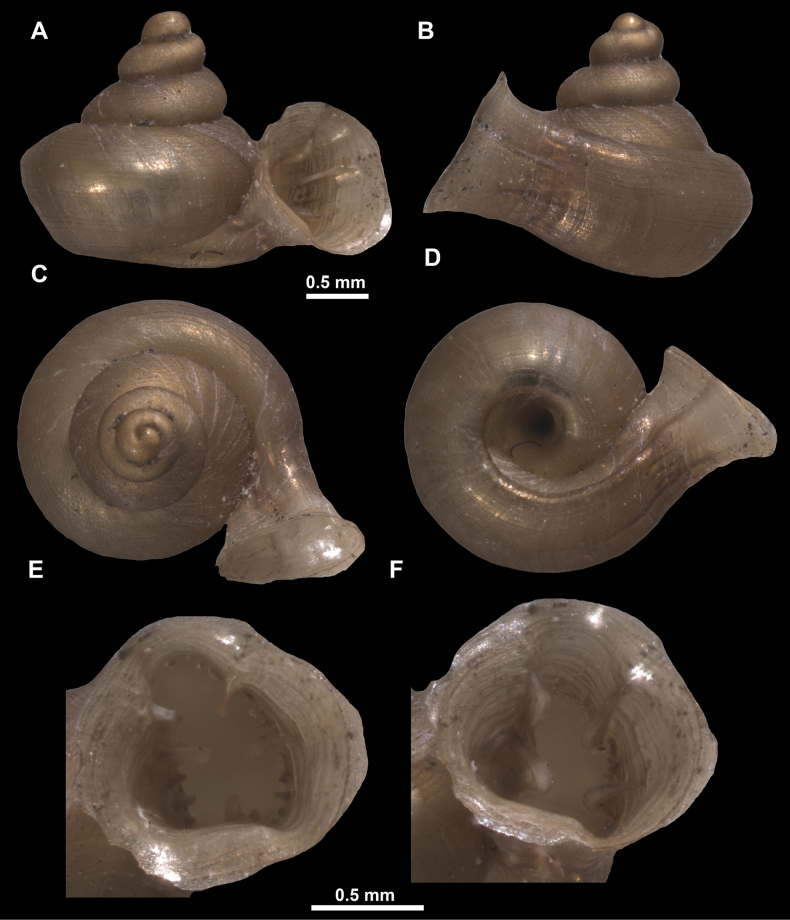
*Hypselostomakhaowongense*, paratype of *G.muangon* (SMF 331459) **A–D** shell **E, F** enlarged apertural views.

**Figure 158.**
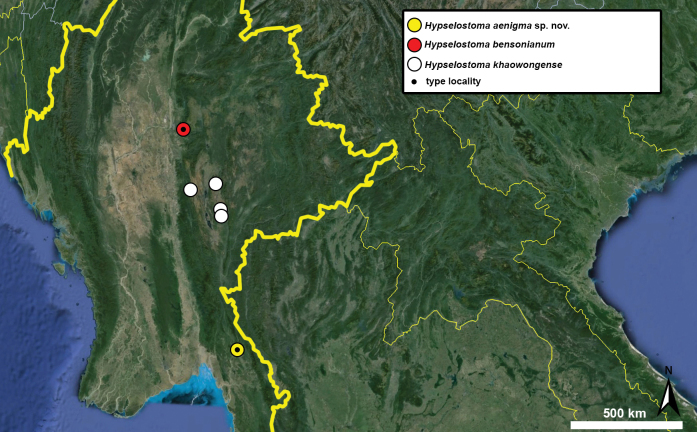
Distribution map of *Hypselostomabensonianum* group in Myanmar.

#### ﻿ Hypselostoma khaowongkot

Taxon classificationAnimaliaStylommatophoraHypselostomatidae

(Panha & J. B. Burch, 2004) comb. nov.

A0D2A6E1-94C7-52C9-A7AC-44D38F0BCDD3

Fig. 185

Gyliotrachela khaowongkot Panha & Burch in Panha et al. 2004: 65–67, fig. 6.
Gyliotrachela khaowongkot — Panha and Burch 2008: 72–73, fig. 63; Dumrongrojwattana and Wongkamhaeng 2024: 323, fig. 8.

##### Material examined.

**Thailand** • holotype; S. Panha leg.; CUMZ ver. 44031.

##### Type locality.

“Khaowongkot, Ban Mee District, Lopburi Province, 15°1'13"N, 100°32'44"E, 60 meters elevation”.

##### Differential diagnosis.

See under *H.tridentatum* and *H.utongense*.

##### Distribution.

This species is known only from the type locality.

#### ﻿ Hypselostoma kohrin

Taxon classificationAnimaliaStylommatophoraHypselostomatidae

(Panha & J. B. Burch, 2003) comb. nov.

651CF96A-72E6-58D6-A9D4-4462F99BCC7C

Gyliotrachela kohrin Panha & Burch in Burch et al. 2003: 151–157, figs 8–9.
Gyliotrachela kohrin — Tongkerd et al. 2004: 143, fig. 2; Panha and Burch 2008: 74–75, fig. 64; Dumrongrojwattana and Wongkamhaeng 2024: 323, fig. 8.

##### Material examined.

**Thailand** • 1 shell; Chonburi Province, Koh Rin, 25 km from eastern coast of Thailand; 26 Oct. 2013; J. U. Otani leg.; coll. PGB • 2 shells; Chonburi Province, Pattaya, Koh Rin, 28 June 2014; J. U. Otani leg.; coll. PGB.

##### Type locality.

“On a limestone hill on Kohrin (Rin Island), which is a small island located in the upper Gulf of Thailand, south of Pattya Beach, eastern coast of Thailand, about 25 kilometers from the coast, Satthip District, Chonberi Province, 12°48'5"N, 100°42'4"E, 30 meters elevation, Thailand”.

##### Differential diagnosis.

This species is similar to *H.diarmaidi* from which it can be separated by the last whorl distally shortly detached as well as the weaker keel and not so concave surface above it. See also under *H.surakiti*.

**Figure 159.**
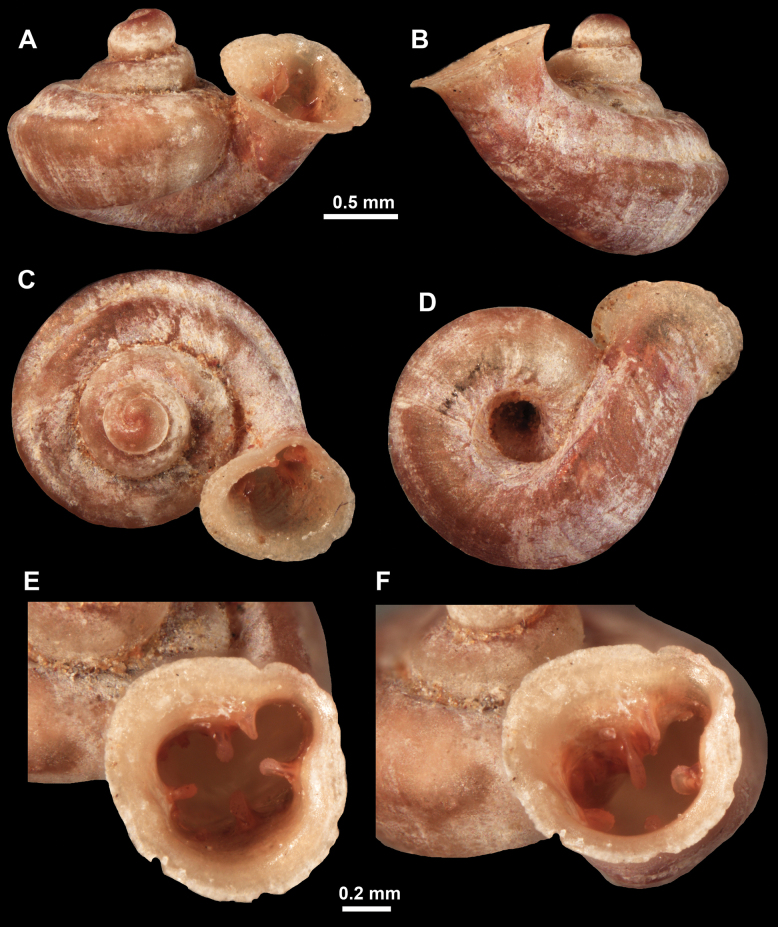
*Hypselostomakohrin*, specimen from the type locality (coll. PGB) **A–D** shell E, **F** enlarged apertural views.

##### Distribution.

This species is known only from the type locality.

#### ﻿ Hypselostoma panhai

Taxon classificationAnimaliaStylommatophoraHypselostomatidae

J. B. Burch & Tongkerd, 2003

4D3E4329-4DE8-5AF5-94AC-5CA1C0254D1E

Fig. 185

Hypselostoma panhai Burch & Tongkerd in Burch et al. 2003: 178–183, fig. 18.
Hypselostoma panhai — Tongkerd et al. 2004: 145, fig. 4D–H; Panha and Burch 2008: 95–97, fig. 82; Dumrongrojwattana and Wongkamhaeng 2024: 324, fig. 8.

##### Type material examined.

**Thailand** • holotype; CUMZ 44055.

##### Type locality.

“Chongkhaokad, west of the World War II museum, near the famous bridge of the now abandoned railroad of World War II, Triyoke District, Kanchanaburi Province, 14°24'8"N, 98°53'23"E, 140 meters elevation, Thailand”.

##### Differential diagnosis.

This species is similar to *H.edentatum* but the latter lacks apertural barriers. However, this species is most similar to *H.iunior* sp. nov. from which it can be separated by the less conical shell, deeper sutures, more convex whorls, clearly weaker parietal callus, slightly wider umbilicus, and much coarser spiral striation (dense in *H.iunior* sp. nov.).

##### Distribution.

This species is known only from the type locality.

#### ﻿ Hypselostoma platybasis

Taxon classificationAnimaliaStylommatophoraHypselostomatidae

Gojšina, Hunyadi & Páll-Gergely sp. nov.

DF288825-80A6-5621-A850-B69ACDD03977

https://zoobank.org/2AC8739A-4E0F-4B44-AE11-4F43450E07AE

##### Type material.

***Holotype*. Cambodia** • 1 shell (SH: 2.60 mm; SW: 2.56 mm); Steung Treng Province, 55.7 km northwest + 2 km north from Stung Treng Mekong Bridge, Chap Phleung Mt. (Neak Khiev Mt.); 13°47.821'N, 105°36.205'E; 135 m a.s.l.; 26 Oct. 2023, A. Hunyadi & J.U. Otani leg.; CUMZ 14457. ***Paratypes*. Cambodia** • 160 shells; same data as for holotype; coll. HA • 1 shell; same data as for holotype; coll. VG.

##### Additional material examined.

**Cambodia** • 10 shells (juveniles, not paratypes); same data as holotype; coll. HA.

##### Type locality.

Steung Treng Province, 55.7 km northwest + 2 km north from Stung Treng Mekong Bridge, Chap Phleung Mt. (Neak Khiev Mt.); 13°47.821'N, 105°36.205'E; 135 m a.s.l.

##### Diagnosis.

*Hypselostoma* species with bluntly keeled penultimate (at the centre of the periphery) and shouldered last whorl. Teleoconch densely spirally striated. Last whorl very slightly detached from the penultimate and with a sharp shoulder. Apertural barriers few (angulo-parietal (parietal), upper palatal, lower palatal and columellar) and relatively weak. Umbilicus initially narrow and then abruptly widening at the last whorl.

**Figure 160.**
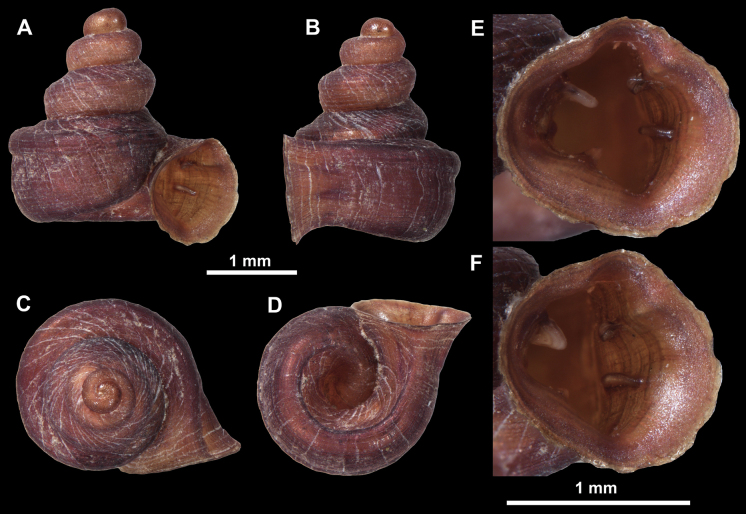
*Hypselostomaplatybasis* Gojšina, Hunyadi & Páll-Gergely, sp. nov., holotype (CUMZ 14457) **A–D** shell **E, F** enlarged apertural views.

##### Description.

Shell conical or slightly concave-conical, dark brown, consisting of 4–4.5 whorls separated by a deep suture. Protoconch rounded, slightly glossy, roughly pitted, consisting of ~ 1.5 whorls. Initial teleoconch whorls rounded, penultimate whorl bluntly keeled. Teleoconch sculptured with strong, raised spiral striae which are crossed by weaker radial growth lines usually in form of whitish radial streaks. There are ~ 30 spiral striae on the last whorl in standard view. The spacing between two striae ranges to ~ 2–3 × the width of one stria. Basis of the last whorl flat. Last whorl with a sharp shoulder (almost like a keel), adnate or very slightly detached from the penultimate, very slightly descending near the aperture (less than 5 ° compared to the shell axis), almost straight. Peristome brown, not very strong but still expanded and not reflected. Aperture equipped with four main barriers (parietal (angulo-parietal), upper palatal, lower palatal and columellar), none of which reach the expanding peristome. Angulo-parietal lamella is the strongest, longest and the highest in the aperture. There is usually no sign of the angular part. Upper and lower palatal plicae both low and relatively weak, almost equally strong. Basal plica clearly present and moderately strong or absent (with only a slight swelling in its place). Columellar lamella developed to a similar extent as both palatal plicae. There is one additional swelling in the columello-parietal embayment which corresponds to the infraparietal lamella. Surface of all apertural barriers is finely granulated. Sinulus wide and not distinctly separated from the rest of the aperture. Umbilicus initially narrow, but abruptly widening at the last whorl, becoming very wide, and measuring slightly > ½ of the shell width. There is a deep groove running from the peristome edge towards the umbilicus along the inner side of the last whorl. This groove is terminating at the transition from the last whorl to penultimate.

**Figure 161.**
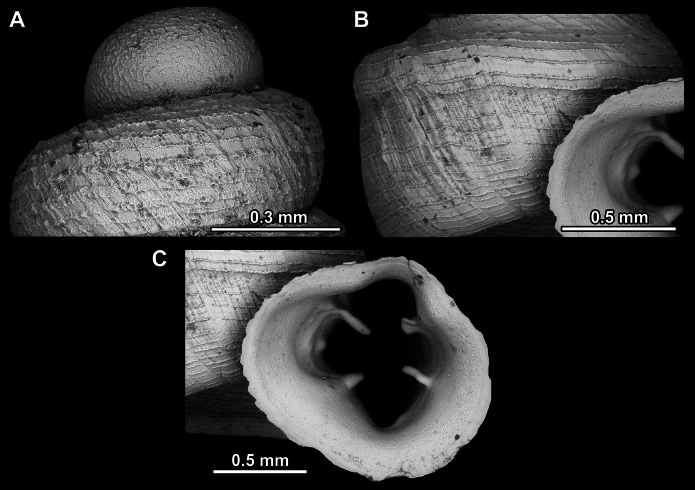
SEM imaging of *Hypselostomaplatybasis* Gojšina, Hunyadi & Páll-Gergely, sp. nov., holotype (CUMZ 14457) **A** protoconch surface **B** teleoconch surface **C** enlarged apertural view.

##### Differential diagnosis.

This species is the most similar to *H.geckophilum* sp. nov. from which it differs in the more rectangular shape of the last whorl and a stronger shoulder. Umbilicus is also much narrower in *H.geckophilum* sp. nov. and the barriers are more slender.

##### Measurements

**(in mm, *n* = 5).**SH = 2.36–2.75; SW = 2.56–2.85; AH = 1.20–1.32; AW = 1.25–1.47.

##### Etymology.

This species is named for the flat base of the shell.

##### Distribution.

This species is only known from the type locality.

##### Remarks.

The largest, and still unexplored limestone hill in Steung Treng Province (13°47.941'N, 105°43.7671'E) was inaccessible during the collecting efforts in 2023 due to the works of a cement factory. Due to the proximity of this hill to the type locality of *H.platybasis* sp. nov., it is possible that this species can be found here as well and be threatened by quarrying.

#### ﻿ Hypselostoma populare

Taxon classificationAnimaliaStylommatophoraHypselostomatidae

Gojšina, Hunyadi & Páll-Gergely sp. nov.

8506BD43-20BD-50BD-B237-29928BE771F5

https://zoobank.org/AC806246-C424-45C4-AB3E-B3BBE4230BE3

##### Type material.

***Holotype*. Thailand** • 1 shell (SH: 1.4 mm; SW: 2.2 mm); Krabi Province, 9.2 km northwest from Krabi towards Phang Nga, right side of the road; 08°07.305'N, 98°52.231'E; 40 m a.s.l.; 21 Feb. 2015, A. Hunyadi leg.; CUMZ 14455. ***Paratypes*. Thailand** • 5 shells; same data as for holotype; coll. HA • 1 shell; Chumphon Province, Pathio, 500 m from the junction Ban Tham Tong - Thung Yang Beach, large rock; 10°54.328'N, 99°29.903'E; 20 m a.s.l.; 23 Feb. 2015; A. Hunyadi leg.; coll. HA • 10 shells; Ranong Province, south of Kra Buri, Tham Phra Kayang, southeastern part of the rock, 10°19.480'N, 98°45.933'E; 22 Feb. 2015; A. Hunyadi leg.; coll. HA • 17 shells; Chumphon Province, 2.5 km northeast from Pathio, Tham Khao Phlu, 10°43.851'N, 99°19.242'E; 30 m a.s.l.; 23 Feb. 2015; A. Hunyadi leg.; coll. HA • 2 shells; Krabi Province, Railay (= Rai Leh) Beach West, Railay Highlands, at the base of limestone rocks; 8°0.865'N, 98°50.219'E; 30 m a.s.l.; Sep. 2007; A. Reischütz leg.; coll. REI • 3 shells; Krabi Province, N of Krabi, Tiger Cave Temple (=Wat Tham Suea), along the ~ 200 steps to the cave, at the base of limestone rocks; 8°7.603'N, 98°55.466'E; 90 m a.s.l.; Sep. 2007; A. Reischütz leg.; coll. REI.

##### Additional material examined.

**Thailand** • 8 shells (damaged/ juveniles, not paratypes); Chumphon Province, 2.5 km northeast from Pathio, Tham Khao Phlu, 10°43.851'N, 99°19.242'E; 30 m a.s.l.; 23 Feb. 2015; A. Hunyadi leg.; coll. HA • 5 shells (damaged/ juveniles, not paratypes); Ranong Province, south of Kra Buri, Tham Phra Kayang, southeastern part of the rock, 10°19.480'N, 98°45.933'E; 22 Feb. 2015; A. Hunyadi leg.; coll. HA.

##### Type locality.

Krabi Province, 9.2 km northwest from Krabi towards Phang Nga, right side of the road; 08°07.305'N, 98°52.231'E; 40 m a.s.l.

##### Diagnosis.

Shell depressed, concave-conical, last whorl strongly detached from the penultimate and ascending at nearly 60–70° angle. Teleoconch strongly spirally striated. Aperture equipped with four blunt barriers (angulo-parietal, upper palatal, lower palatal, and columellar). Umbilicus very wide.

**Figure 162.**
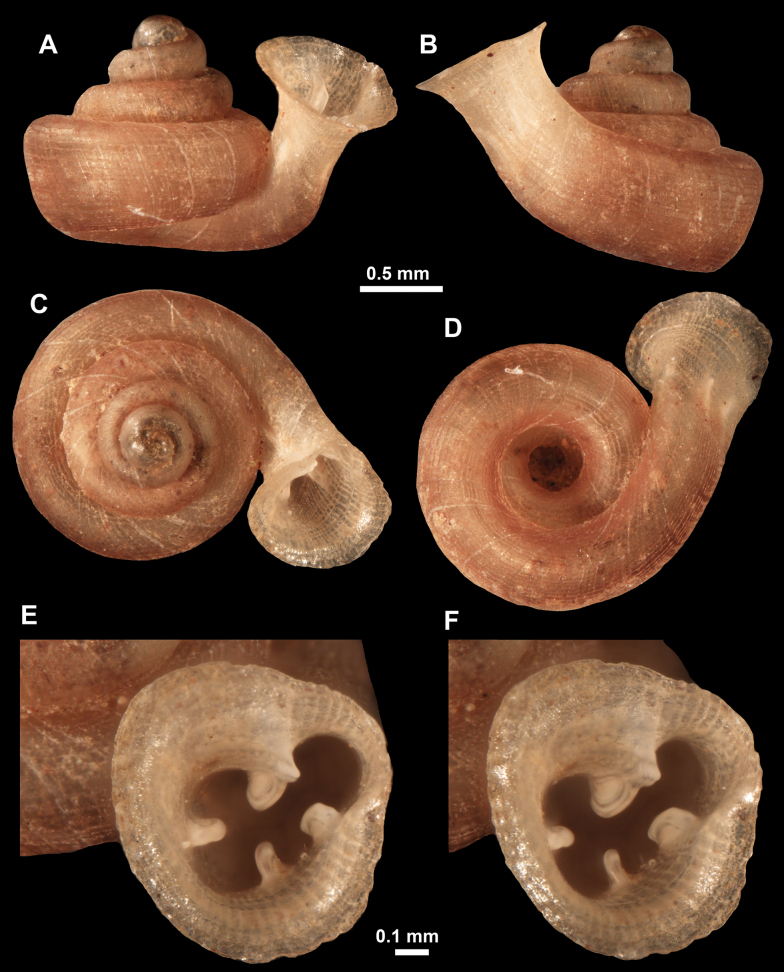
*Hypselostomapopulare* Gojšina, Hunyadi & Páll-Gergely, sp. nov., holotype (CUMZ14455) **A–D** shell **E, F** enlarged apertural views.

##### Description.

Shell depressed, concave-conical (due to the strongly enlarged last whorl), consisting of 3.75–4.25 whorls separated by a very deep suture. Colouration yellowish or very pale brownish, opaque. Protoconch initially almost smooth and then finely pitted, showing spiralling pattern and consisting of 1.25–1.5 whorls. Boundary between the protoconch and teleoconch clearly visible due to the change of microsculpture. Teleoconch strongly and regularly spirally striated, spiral striae thread-like raised. Space between two spiral striae measuring approximately the width of two to four spiral striae. There are ~ 30 spiral striae on the last whorl in standard view. Weaker, irregularly spaced radial growth lines are also visible crossing the spiral striae. Some irregularly spaced radial white streaks are occasionally present. Last whorl shouldered, strongly detached from the penultimate and ascending strongly near the aperture (~ 60–70° compared to the shell axis). Peristome lighter than the rest of the shell, fragile, expanded and not reflected. Aperture equipped with four moderately strong barriers (angulo-parietal, upper palatal, lower palatal, and columellar). Angulo-parietal lamella is the strongest in the aperture, consisting of two parts corresponding to angular and parietal lamellae in the former *Gyliotrachela*. The angular part of the angulo-parietal lamella is very small and pointy, leaning towards the palatal wall. The parietal part of the angulo-parietal lamella is much stronger and positioned deeper in the aperture, leaned towards the columellar lamella. Both palatal plicae and a columellar lamella are developed to similar level, relatively short and blunt. Upper palatal plica nearly fully rectangular in shape, lower palatal plica and columellar lamella more rounded and positioned even deeper in the aperture than the upper palatal plica. The apertural barriers are not very variable. None of these barriers reach the peristome. Surface of all apertural barriers is finely granulated. Sinulus rounded and well separated from the rest of the aperture. Umbilicus open, very wide, measuring ~ 1/3 of the shell width and showing all previous whorls.

**Figure 163.**
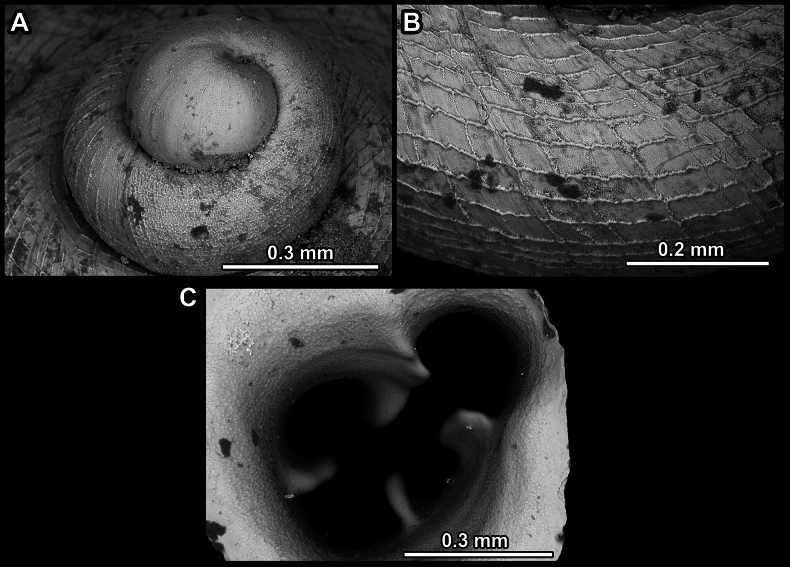
SEM imaging of *Hypselostomapopulare* Gojšina, Hunyadi & Páll-Gergely, sp. nov., holotype (CUMZ 14455) **A** protoconch surface **B** teleoconch surface **C** enlarged apertural view.

##### Differential diagnosis.

This species is nearly identical in external appearance to *Hypselostomaerawan*. However, *H.erawan* clearly differs in the appearance of the apertural barriers. *Hypselostomaerawan* has more barriers than the new species and palatal plicae are hooked (neither of the barriers in *H.populare* sp. nov. are hooked). *Acinolaemuscolpodon* F. G. Thompson & Upatham, 1997 is smaller and also has hooked barriers. *Hypselostomacucumense* has the same appearance of the apertural barriers but the last whorl in *H.populare* sp. nov. is without a keel. The umbilicus is also initially narrow in *H.cucumense* and then widening at the last whorl, but it is wide even in its initial parts in *H.populare* sp. nov.

##### Measurements

**(in mm, *n* = 5).**SH = 1.33–1.41; SW = 2.13–2.22; AH = 0.79–0.86; AW = 0.7–0.87.

##### Etymology.

Named after the fact that many sampling sites of this species are popular touristic destinations.

##### Distribution.

This species is known from Krabi, Chumphon and Ranong provinces in Thailand.

##### Remarks.

This species is not very variable in terms of shell size and apertural dentition but in some specimens the spire is more depressed conical than in others.

#### ﻿ Hypselostoma rupestre

Taxon classificationAnimaliaStylommatophoraHypselostomatidae

van Benthem Jutting, 1962

DC1C4FFC-49A9-5C5F-84B0-51C6EBCE57D1

Hypselostoma rupestre van Benthem Jutting, 1962: 6–8, fig. 3.
Hypselostoma rupestre — Schileyko 2011: 3–4; Vermeulen et al. 2019: 40.

##### Material examined.

**Cambodia** • 32 shells; Kampot Province, Kampong Trach district, Phnom Teuk Thom (northwest from Wat Aranhatry); 10°26.335'N, 104°28.822'E; 400 m a.s.l.; 21 Oct. 2023; A. Hunyadi, T. Ishibe, K. Okubo, J.U. Otani leg.; coll. HA.

##### Type locality.

“Nui Hon Chong (Cap de la Table), à 32 km au S.-*E. de* Hatien, Sud Vietnam”.

##### Differential diagnosis.

This species differs from *H.cambodjense* by its rounded whorls. It is thus superficially more similar to *H.dilatatum* but the latter has several hooked apertural barriers (they are all blunt in *H.rupestre*).

**Figure 164.**
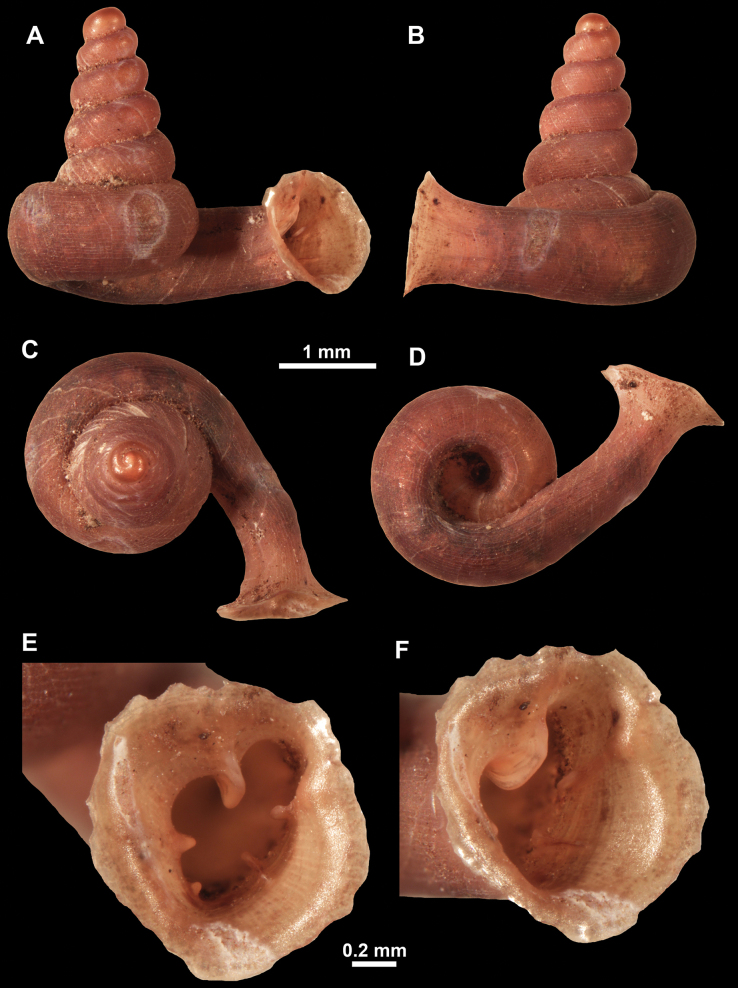
*Hypselostomarupestre* from Cambodia (coll. HA) **A–D** shell **E, F** enlarged apertural views.

##### Distribution.

This species in known from several localities in Kien Giang province (Vietnam). We have also found this species in Kampot province, Cambodia (see under Material examined).

#### ﻿ Hypselostoma salpinx

Taxon classificationAnimaliaStylommatophoraHypselostomatidae

(van Benthem Jutting, 1961) comb. nov.

8DB4A8D8-1791-5734-9885-34CE3BDB6270

Gyliotrachela salpinx van Benthem Jutting, 1961: 38–39, pl. 10, fig. 4.
Gyliotrachela salpinx — Maassen 2001: 76.

##### Type material examined.

**Malaysia** • 60 paratypes; from the type locality; Aug. 1950; Raffles Museum ex. coll.; RMNH.Moll.137154.

##### Additional material examined.

**Malaysia** • 6 shells; Pahang, 10 km northeast from Raub, 1.5 km northwest from Sungai Ruan, vicinity of Bukit Serdam limestone rock; 03°49.817'N, 101°55.387'E; 170 m a.s.l.; 20 Jan. 2013; A. Hunyadi leg.; coll. HA • 6 shells; Pahang, Tok Machang (end of Raub), road Raub-Sungai Ruan; Oct. 1998; ex. coll. Hemmen, Wiesbaden; coll. PGB. **Thailand** • 4 damaged shells; Thailand, Suratthani Province, Angthong islands, Mae Koh island, footpath to the Lagoon, at the base of limestone rocks; 9°39.460'N, 99°39.976'E; 20 m a.s.l.; Sep. 2007; A. Reischütz leg.; coll. REI (cf. salpinx) • 1 shell; Phattalung Province, 6.4 km E of Hwy. 4081, 1 km SW of Khao Chai Son; 7°27'N, 100°11'E; 11 Apr. 1988; K. Auffenberg leg.; locality code KA-0648; UF 591343 • 1 shell; same locality data as previous; 11 Apr. 1988; K. Auffenberg leg.; locality code KA-0648; UF 591342; 3 shells; Phattalung Province, 6 km NW of Phattalung, 1 km NE of Hwy. 4048; 7°40'N, 100°3'E; 60 m a.s.l.; 13 Apr. 1988; K. Auffenberg leg.; locality code KA-0653; UF 591345 • 5 shells; Phattalung Province, 6.4 km E of Hwy. 4 on Hwy. 4081, 1 km SW of Khao Chai Son, large limestone outcrop with solution pits; 7.450°N, 100.183°E; 50 m a.s.l.; 11 Apr. 1988; K. Auffenberg leg.; locality code KA-0649; UF 591344 • 1 shell; Phattalung Province, Khao Pu Khao Ya National Park, Matcha cave vicinity; 7°39.980'N, 99°52.343'E; 125 m a.s.l.; 25 Feb. 2023; A. Hunyadi leg.; coll. HA • 35 shells; Songkhla Province, 31.3 km NW of Hat Yai, 1.2 km W of Hwy. 43; 7°10'N, 100°16'E; 80 m a.s.l.; 08 Apr. 1988; K. Auffenberg leg.; locality code KA-0635; UF 344926 • 5 shells; same locality data as previous; 08 Apr. 1988; K. Auffenberg leg.; locality code KA-0636; UF 591341.

##### Type locality.

“Bukit Serdam, near Raub, Pahang” (Malaysia).

##### Differential diagnosis.

See under *H.saxicola* and *H.coriaceum* sp. nov.

##### Distribution.

This species is known only from the limited area (Raub) in Malaysia and Suratthani, Phattalung and Songkhla provinces in Thailand.

**Figure 165.**
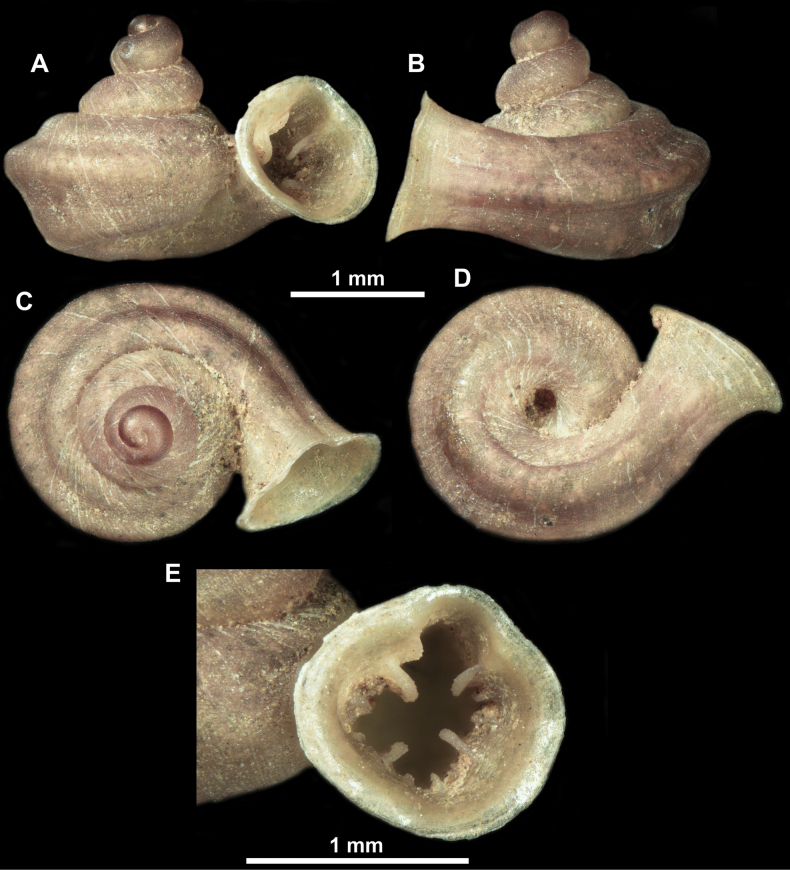
*Hypselostomasalpinx*, paratype (RMNH Moll.137154) **A–D** shell **E** enlarged apertural view.

**Figure 166.**
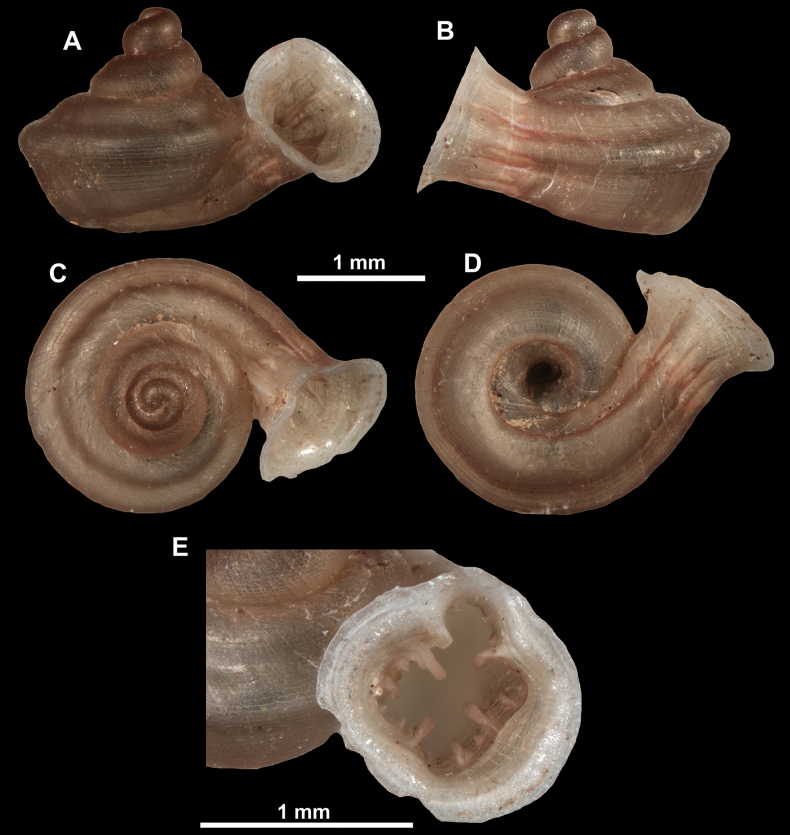
*Hypselostomasalpinx* from Phattalung Province, Thailand (UF 591342) **A–D** shell **E** enlarged apertural view.

#### ﻿ Hypselostoma saxicola

Taxon classificationAnimaliaStylommatophoraHypselostomatidae

(van Benthem Jutting, 1960) comb. nov.

FBA32FF5-A19C-5B32-A91D-752F4B11A18A

Gyliotrachela saxicola van Benthem Jutting, 1960: 14–16, fig. 1.
Gyliotrachela saxicola — Maassen 2001: 76.

##### Type material examined.

**Malaysia** • 22 paratypes; Perlis, N of Kangar, limestone hill near Kampong Tebing Tinggi, on the face of the rock; 19 Dec. 1958; W. S. *S. van* der Feen-van Benthem Jutting leg.; RMNH.Moll.137139.

##### Type locality.

“Limestone hill near Kampong Tebing Tinggi, N. of Kangar, Perlis”, Malaysia.

##### Differential diagnosis.

This species is most similar to *H.salpinx* from which it can be separated by smaller number of overall stronger barriers and wider umbilicus (which is initially narrower in *H.salpinx*). See also under *H.coriaceum* sp. nov.

##### Distribution.

This species is known only from the type locality.

##### Remarks.

In paratypes examined, besides the five barriers that are always present, there were additional 1–3 smaller. These are interpalatal, infrapalatal and infraparietal and they were present in some and absent in other specimens. As a consequence, the number of barriers varied from 6–8 (as there was always at least one of the smaller). This was also noted by van Benthem Jutting (1960) in the original description. She also noted that the last whorl can be more or less detached or completely adnate, but this variation is not useful for separation of the species.

**Figure 167.**
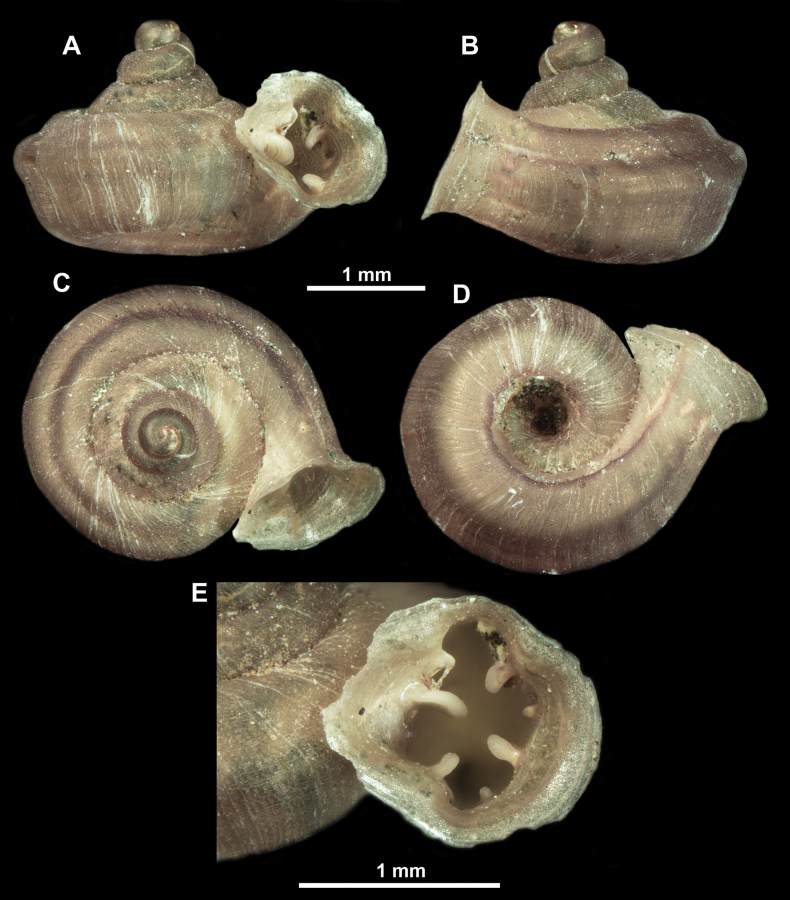
*Hypselostomasaxicola*, paratype (RMNH Moll.137139) **A–D** shell **E** enlarged apertural view.

#### ﻿ Hypselostoma sculpturatum

Taxon classificationAnimaliaStylommatophoraHypselostomatidae

Gojšina, Hunyadi & Páll-Gergely sp. nov.

3570D9AD-BB17-5A1D-B6E8-A3D70AE6E5B5

https://zoobank.org/C15FAC41-6ED5-422B-A0EC-DBC8C31DDC98

##### Type material.

***Holotype*. Cambodia** • 1 shell (SH: 2.70 mm; SW1: 3.80 mm); Steung Treng Province, 36.3 km northwest + 5 km north from Stung Treng Mekong Bridge, Phnom Chhnok; 13°46.573'N, 105°44.878'E; 120 m a.s.l.; 25 Oct. 2023; A. Hunyadi & J.U. Otani leg.; CUMZ 14458.

***Paratypes*. Cambodia** • 51 shells; same data as for holotype; coll. HA • 1 shell; same data as for holotype; coll. VG.

##### Additional material examined.

**Cambodia** • 4 shells (juveniles, not paratypes); same data as holotype; coll. HA.

##### Type locality.

Cambodia, Steung Treng Province, 36.3 km northwest + 5 km north from Stung Treng Mekong Bridge, Phnom Chhnok; 13°46.573'N, 105°44.878'E; 120 m a.s.l.

##### Diagnosis.

*Hypselostoma* species with all teleoconch whorls keeled, detached last whorl and strongly spirally striated shell surface. There are five strong barriers in the aperture (angulo-parietal, upper palatal, lower palatal, basal and columellar). Umbilicus initially narrow, suddenly widening at the last whorl.

##### Description.

**Figure 168.**
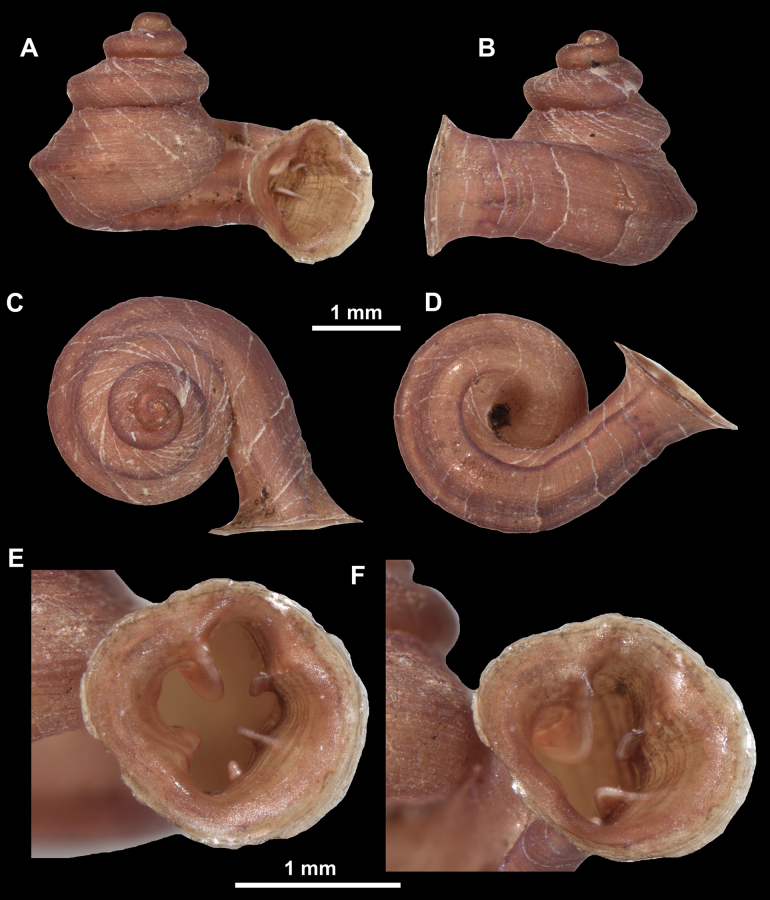
*Hypselostomasculpturatum* Gojšina, Hunyadi & Páll-Gergely, sp. nov., holotype (CUMZ14458) **A–D** shell **E, F** enlarged apertural views.

Shell conical-ovoid, brownish, not glossy. It is consisting of 4–4.5 whorls separated by a deep suture. Protoconch rounded, of ~ 1.75 finely pitted whorls of the same colour as the rest of the shell. Teleoconch sculptured with strong, raised spiral striae. Spacing between two striae equals the width of one stria or is twice as wide as a stria. Spiral striae are more densely arranged on the initial teleoconch whorls, and more coarsely on the last whorl. The number of striae on the last whorl is ~ 25 in standard view. There are several white, unevenly positioned, radial streaks crossing the spiral striae. All teleoconch whorls are keeled, and the strength of the keel is increasing towards the last whorl which is especially strongly keeled. Surfaces of the whorls above and below the centrally positioned keel are flat, to very slightly concave (above the keel on the last whorl). Last whorl moderately detached from the penultimate, distanced from the shell axis. From the lateral view (Fig. 168B), it can be seen that the last whorl is initially slightly ascending and then straightening right near the aperture so that the aperture profile is almost parallel to the shell axis (leaving the aperture only slightly ascending at ~ 5–10 ° compared to the shell axis). Peristome light brownish, expanded and not reflected. Aperture equipped with five barriers (angulo-parietal, upper palatal, lower palatal, basal, and columellar). Angulo-parietal strong and high, lamella usually appears as a single barrier (parietal) but has a very short swollen part in front of it which most probably represents the remains of the angular part. Upper palatal plica moderately high, slightly curved towards the lower palatal plica (somehow similar to the upper palatal plica in *H.serpa*). Lower palatal plica slightly higher, longer, and more slender than the upper palatal but similar in width. It is pointing towards the embayment between the columellar and parietal lamellae. Basal plica more closely positioned to the lower palatal (not in its usual position between the lower palatal and columellar), ~ 2 × lower than the latter and short. Columellar lamella thick and broad, more closely resembling a very large swelling than a distinct barrier. There is also a small swelling between the columellar and parietal lamellae. Surface of all apertural barriers is finely granulated. Sinulus narrow, distinctly separated from the rest of the aperture. Umbilicus initially very narrow and suddenly widening at the last whorl. It is measuring between ½ and ¹⁄3 of the shell width. There is a deep groove running from the peristome edge towards the umbilicus along the inner side of the last whorl. This groove is terminating at the transition from the last whorl to penultimate.

**Figure 169.**
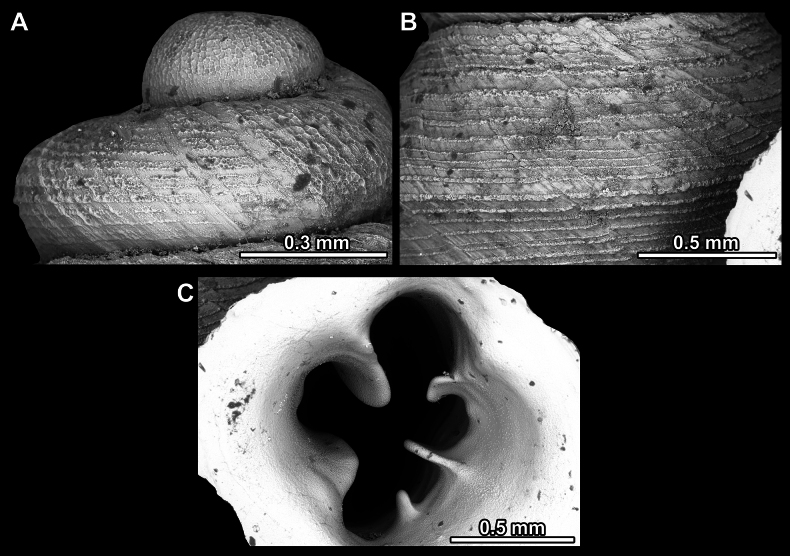
SEM imaging of *Hypselostomasculpturatum* Gojšina, Hunyadi & Páll-Gergely, sp. nov., holotype (CUMZ 14458) **A** protoconch surface **B** teleoconch surface **C** enlarged apertural view.

##### Differential diagnosis.

This species superficially resembles *H.circumcarinatum* sp. nov. but the latter has its last whorl adnate from the penultimate, weaker barriers, narrower umbilicus, and less prominent shell sculpture. The new species is distinguished from *H.khmerianum* by the concrescent angular and parietal lamella as well as by the presence of spiral striation.

See under *H.taehwani*.

##### Measurements

**(in mm, *n* = 5).**SH = 2.35–2.70; SW1 = 3.52–3.80; SW2 = 2.08–2.18; AH = 1.32–1.62; AW = 1.40–1.79.

##### Etymology.

This species is named for the strong surface sculpture of its shell.

##### Distribution.

This species is only known from the type locality.

##### Remarks.

The largest, and still unexplored limestone hill in Steung Treng Province (13°47.941'N, 105°43.7671'E) was inaccessible during the collecting efforts in 2023 due to the works of a cement factory. Due to the proximity of this hill to the type locality of *H.sculpturatum* sp. nov., it is possible that this species can be found here as well and be threatened by quarrying.

#### ﻿ Hypselostoma smokon

Taxon classificationAnimaliaStylommatophoraHypselostomatidae

(Panha & J. B. Burch, 2004)

96887433-8F08-5E53-8FEF-1899AD1850DA

Anauchen smokon Panha & Burch in Panha et al. 2004: 60, fig. 3.
Hypselostoma smokon — Panha and Burch 2008: 99–100, fig. 85; Dumrongrojwattana and Wongkamhaeng 2024: 324, fig. 8.

##### Type material examined.

**Thailand** • 2 paratypes; from the type locality; S. Panha leg.; SMF 331457.

##### Type locality.

“Smokon Mountain, Banmi District, Lopburi Province, 14°54'54"N, 100°29'45"E, 30 meters elevation”.

##### Differential diagnosis.

This species is similar in shell shape to *H.chedi* and *H.khaochakan*. From them, *H.smokon* clearly differs by the concrescent angular and parietal lamellae. It is most similar to *H.chedi* which has its last whorl bluntly keeled (shouldered in *H.smokon*) and narrower umbilicus.

**Figure 170.**
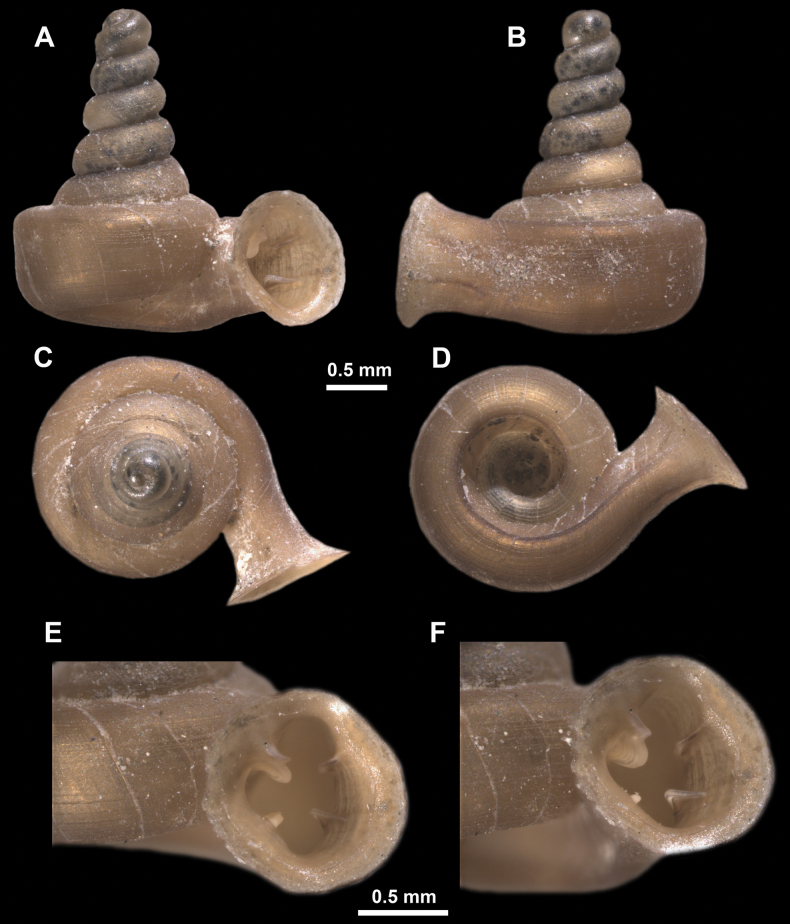
*Hypselostomasmokon*, paratype (SMF 331457) **A–D** shell E, **F** enlarged apertural views.

##### Distribution.

This species is known only from the type locality.

##### Remarks.

In the original description, the species was placed in the genus *Anauchen* because of the absent angular lamella. However, it is present as an angular part of the joined angulo-parietal lamella which justifies its placement in the genus *Hypselostoma*.

#### ﻿ Hypselostoma sorormajor

Taxon classificationAnimaliaStylommatophoraHypselostomatidae

Gojšina, Hunyadi & Páll-Gergely sp. nov.

F0E153FF-8E5E-5689-ADB2-4DF76E7EEE3F

https://zoobank.org/68BDC479-896A-490C-A3A5-1C0230CCF20B

##### Type material.

***Holotype*. Cambodia** • 1 shell (SH: 2.70 mm; SW1: 3.91 mm); Steung Treng Province, 55.7 km northwest + 2 km north from Stung Treng Mekong Bridge, Chap Phleung Mt. (Neak Khiev Mt.); 13°47.821'N, 105°36.205'E; 135 m a.s.l.; 26 Oct. 2023, A. Hunyadi & J.U. Otani leg.; CUMZ 14459. ***Paratypes*. Cambodia** • 28 shells; same data as for holotype; coll. HA.

##### Additional material examined.

**Cambodia** • 3 shells (2 damaged and 1 juvenile, not paratypes); same data as for holotype; coll. HA • 41 shells; Steung Treng Province, 40 km northwest + 2 km north from Stung Treng Mekong Bridge, limestone mountain at the left side of the joining road; 13°45.568'N, 105°43.644'E; 135 m a.s.l.; 26 Oct. 2023; A. Hunyadi & J.U. Otani leg.; coll. HA • 78 shells; Steung Treng Province, Stung Treng Mekong Bridge, 45 km NWN + 400 m N, limestone hill, right side of the road #64; 13°45.866'N, 105°41.154'E; 180 m a.s.l.; 26 Oct. 2023; A. Hunyadi & J.U. Otani leg.; coll. HA • 1 shell; same data as previous; coll. VG • 26 shells; Steung Treng Province, 36.3 km northwest + 3.1 km north from Stung Treng Mekong Bridge, limestone mountain on the left side of the joining road; 13°45.585'N, 105°44.930'E; 130 m a.s.l.; 25 Oct. 2023; A. Hunyadi & J. U. Otani leg.; coll. HA.

##### Type locality.

Cambodia, Steung Treng Province, 55.7 km northwest + 2 km north from Stung Treng Mekong Bridge, Chap Phleung Mt. (Neak Khiev Mt.); 13°47.821'N, 105°36.205'E; 135 m a.s.l.

##### Diagnosis.

A *Hypselostoma* species with depressed, concave-conical shell, bluntly and strongly keeled last whorl, raised spiral striation, hooked apertural barriers (upper palatal, interpalatal, lower palatal and basal) and very wide umbilicus. Angular and parietal lamellae fused. Shell width reaches 4 mm.

**Figure 171.**
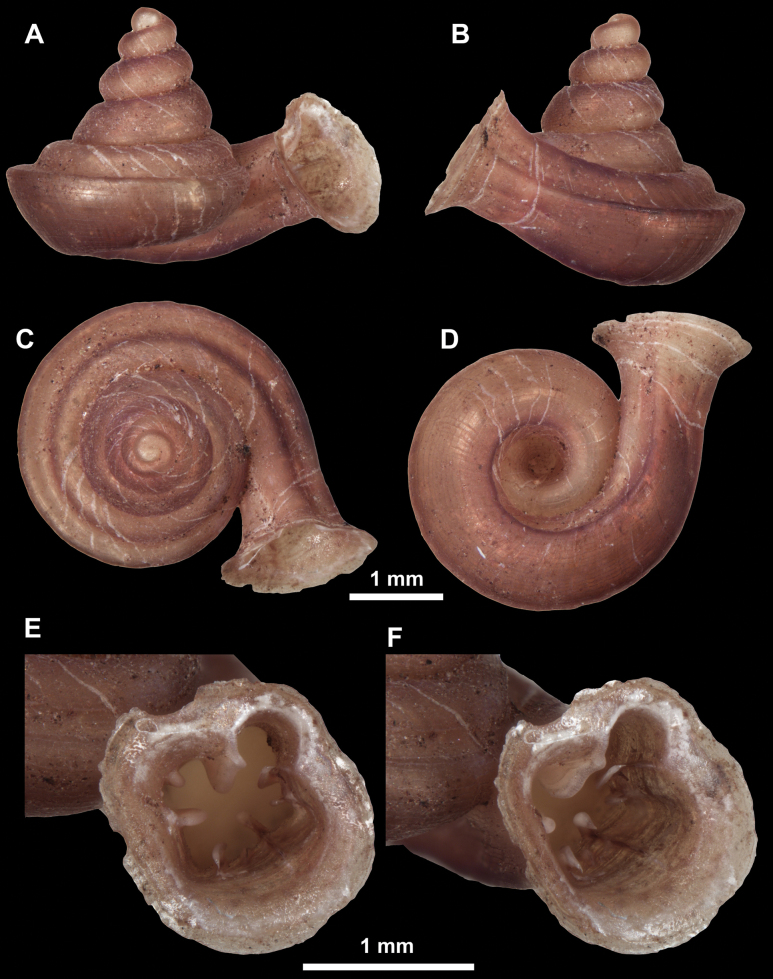
*Hypselostomasorormajor* Gojšina, Hunyadi & Páll-Gergely, sp. nov., holotype (CUMZ14459) **A–D** shell **E, F** enlarged apertural views.

##### Description.

Shell depressed, concave-conical, brown to yellowish-brown, consisting of 4.75–5.5 whorls separated by a deep suture. Protoconch slightly lighter than the rest of the shell, finely pitted, showing spiralling pattern initially and clearer spiral striae terminally. It is consisting of ~ 1.25–1.5 whorls. Protoconch and initial teleoconch whorls rounded, penultimate weakly convex. Last whorl with strong but blunt keel positioned at the centre of the periphery. Above the keel there is a deep or rarely shallow groove. All whorls densely sculptured with strong, raised spiral striae crossed by less dense radial growth lines and occasionally by a few thick, whitish radial streaks. Spacing between the spiral striae irregular, but usually around the width of two to four spiral striae. Last whorl moderately detached from the penultimate and moderately ascending (~ 35 ° compared to the shell axis). Peristome expanded, not reflected, its surface finely pitted. Aperture equipped with four main barriers (angulo-parietal, upper palatal, lower palatal, and columellar) and several smaller ones. Angulo-parietal lamella strong, its angular part is leaned towards the palatal side while the parietal part is leaned towards the columellar side. Angular part is pointed and smaller than the parietal. It is the only barrier reaching the expanding peristome. Sometimes, there is a deep constriction between the angular and parietal part so that these lamellae appear almost separated (as in former *Gyliotrachela*). Upper and lower palatal plicae are hooked, pointing outside and roughly the same size. Columellar lamella thick and almost horizontal. Of smaller barriers, there is usually one hooked interpalatal, one infrapalatal, one basal plica (hooked or blunt) and one blunt lamella in the columello-parietal transition embayment. Of them, the strongest one is the basal, which is almost as strong as the palatals. A distinct swelling is also observed at the palatal side (in front of the upper palatal and interpalatal plicae) which is probably homologous to the palatal tubercle in the majority of *Bensonella* species. Surface of all apertural barriers is finely granulated. Sinulus rounded, distinctly separated from the rest of the aperture. Umbilicus very wide, measuring ~ ½ of the shell width. There is a periumbilical keel situated on the last ~ 0.75 whorl right above the umbilicus. Along this keel there is a deep groove on the umbilical side.

**Figure 172.**
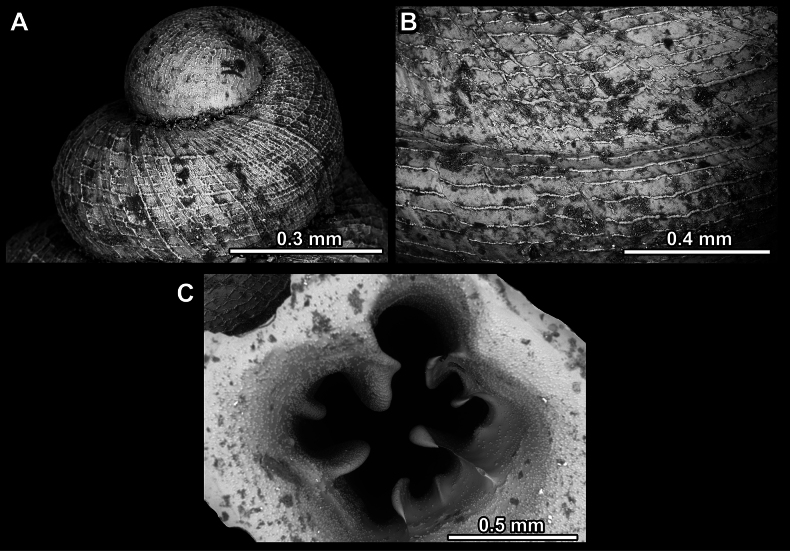
SEM imaging of *Hypselostomasorormajor* Gojšina, Hunyadi & Páll-Gergely, sp. nov., holotype (CUMZ 14459). **A** protoconch surface **B** teleoconch surface **C** enlarged apertural view.

##### Differential diagnosis.

This species differs from *H.discobasis* in the less slender shell and hooked apertural barriers. *Hypselostomaaquila* sp. nov. is much smaller, more slender and has a narrower umbilicus. For differences from *H.sororminor* sp. nov. see under that species.

**Figure 173.**
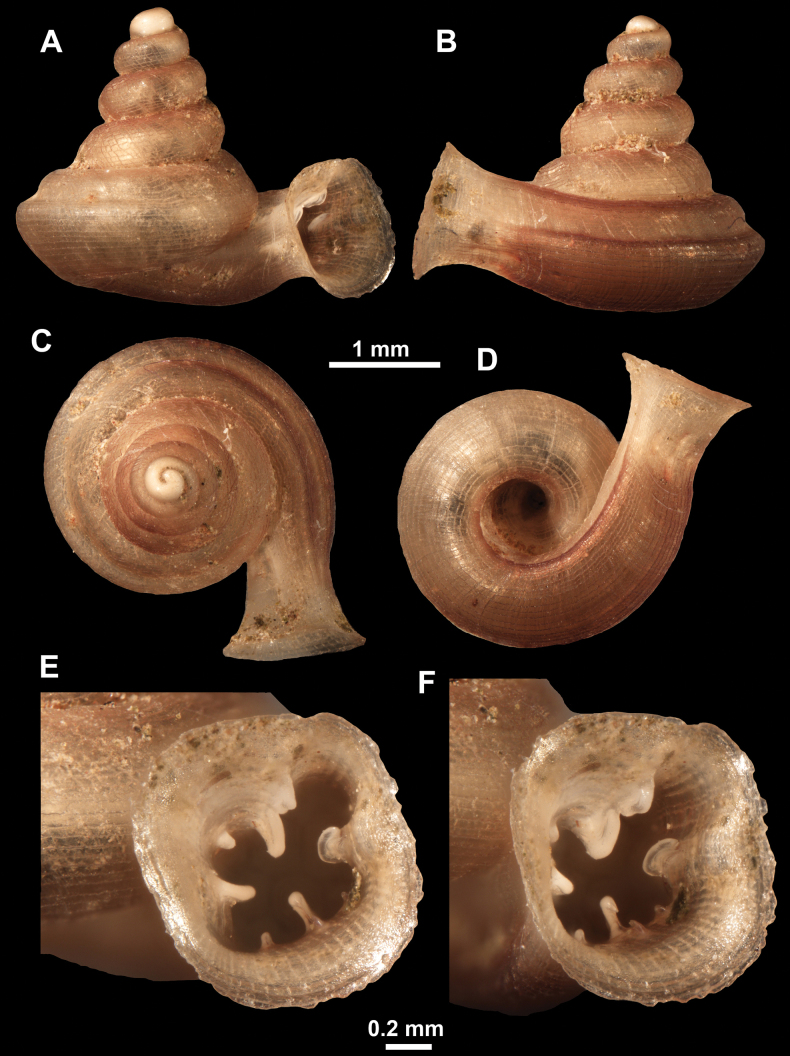
*Hypselostomasorormajor* Gojšina, Hunyadi & Páll-Gergely, sp. nov., specimen with almost separated angular and parietal lamellae (coll. HA) **A–D** shell E, **F** enlarged apertural views.

##### Measurements

**(in mm, *n* = 15).**SH = 1.62–2.78; SW1 = 2.44–4.01; SW2 = 1.57–2.56; AH = 0.85–1.71; AW = 0.81–1.46.

**Figure 174.**
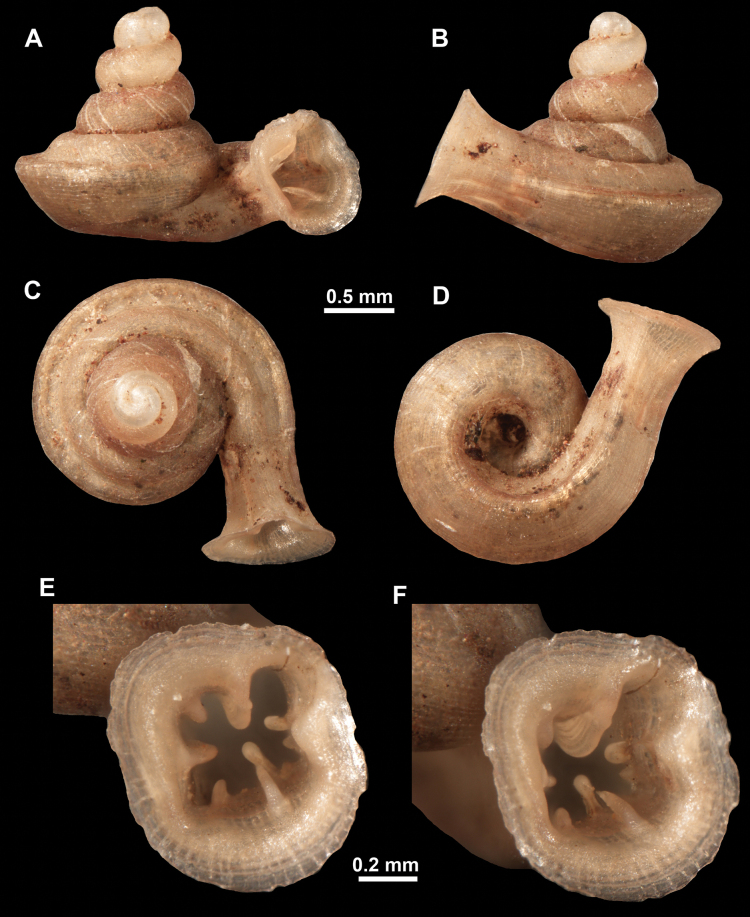
*Hypselostomasorormajor* Gojšina, Hunyadi & Páll-Gergely, sp. nov., smaller specimen (SW ~ 2.5 mm) (coll. HA) **A–D** shell E, **F** enlarged apertural views.

##### Etymology.

The name of this species is derived from two Latin words, *soror* meaning sister and *major* meaning greater, larger, or elder, which serves as a comparison with similar but smaller *H. sororminor* sp. nov.

##### Distribution.

This species is known from four closely situated localities in Steung Treng Province.

##### Remarks.

This is a very variable species in terms of shell size. These different “forms” were not regarded as distinct species since there were no other significant morphological differences noticed. The angulo-parietal lamellae were sometimes clearly fused, while sometimes almost completely separated. The largest, and still unexplored limestone hill in Steung Treng Province (13°47.941'N, 105°43.7671'E) was inaccessible during the collecting efforts in 2023 due to the works of a cement factory. Due to the proximity of this hill to the type locality of *H.sorormajor* sp. nov., it is possible that this species can be found here as well and be threatened by quarrying.

#### ﻿ Hypselostoma sororminor

Taxon classificationAnimaliaStylommatophoraHypselostomatidae

Gojšina, Hunyadi & Páll-Gergely sp. nov.

B902884D-17DF-528C-9546-4B957F1870A0

https://zoobank.org/4801B9B0-1DDB-4000-BF92-02AC62B71BF5

##### Type material.

***Holotype*. Cambodia** • 1 shell (SH: 2.44 mm; SW1: 3.57 mm); Steung Treng Province, 36.6 km northwest + 3.1 km north from Stung Treng Mekong Bridge, limestone mountain on the left side of the joining road; 13°45.585'N, 105°44.930'E; 130 m a.s.l.; 25 Oct. 2023, A. Hunyadi & J.U. Otani leg.; CUMZ 14460. ***Paratypes*. Cambodia** • 32 shells; same data as for holotype; coll. HA • 1 shell; same data as for holotype; coll. VG.

##### Additional material examined.

**Cambodia** • 2 shells (juveniles/ damaged, not paratypes); same data as for holotype; coll. HA.

##### Type locality.

Cambodia, Steung Treng Province, 36.6 km northwest + 3.1 km north from Stung Treng Mekong Bridge, limestone mountain on the left side of the joining road; 13°45.585'N, 105°44.930'E; 130 m a.s.l.

##### Diagnosis.

A *Hypselostoma* species with depressed, concave-conical shell, bluntly and strongly keeled last whorl, raised spiral striation, blunt apertural barriers (altogether 6 or 7) and very wide umbilicus. Angular and parietal lamellae fused.

##### Description.

Shell depressed, concave-conical, brown, consisting of 5–5.5 whorls separated by a deep suture. Protoconch lighter than the rest of the shell, coarsely spirally striated (~ 12 striae) and consisting of 1.25–1.5 whorls. Protoconch and initial teleoconch whorls rounded, penultimate weakly convex. Last whorl with strong but blunt keel positioned at the centre of the periphery. Above the keel there is a deep groove. All whorls densely sculptured with strong, raised spiral striae crossed by less dense radial growth lines and occasionally by a few thin, whitish radial streaks. Spacing between the spiral striae irregular, but usually around the width of two, three, or four spiral striae. Last whorl slightly to moderately detached from the penultimate and slightly to moderately ascending near the aperture (~ 25–35 ° compared to the shell axis). Peristome expanded, not reflected, its surface finely pitted. Aperture equipped with four strong, main barriers (angulo-parietal, upper palatal, lower palatal, and columellar) and several smaller ones. Angulo-parietal lamella strong, its angular part is leaned towards the palatal side while its parietal part is leaned towards the columellar side. Angular part is pointed and smaller than the parietal. It is the only barrier reaching the expanding peristome. Upper and lower palatal plicae approximately the same size, the lower palatal being longer and more slender. Upper palatal plica slightly bent towards the lower palatal. Columellar lamella very thick and almost horizontal. Of smaller barriers, a small and weak interpalatal plica is present, stronger (but still weak) basal and a moderate knob-like plica in the columello-parietal transition embayment. A weak swelling is also observed at the palatal side (in front of the upper palatal plicae) which is probably homologous to the palatal tubercle in the majority of *Bensonella* species. Surface of all apertural barriers is densely granulated. Sinulus rounded, distinctly separated from the rest of the aperture. Umbilicus very wide, measuring between 1/3 and ½ of the shell width. There is a periumbilical keel situated on the last ~ 0.75 whorl right above the umbilicus. Along this keel there is a deep groove on the umbilical side.

**Figure 175.**
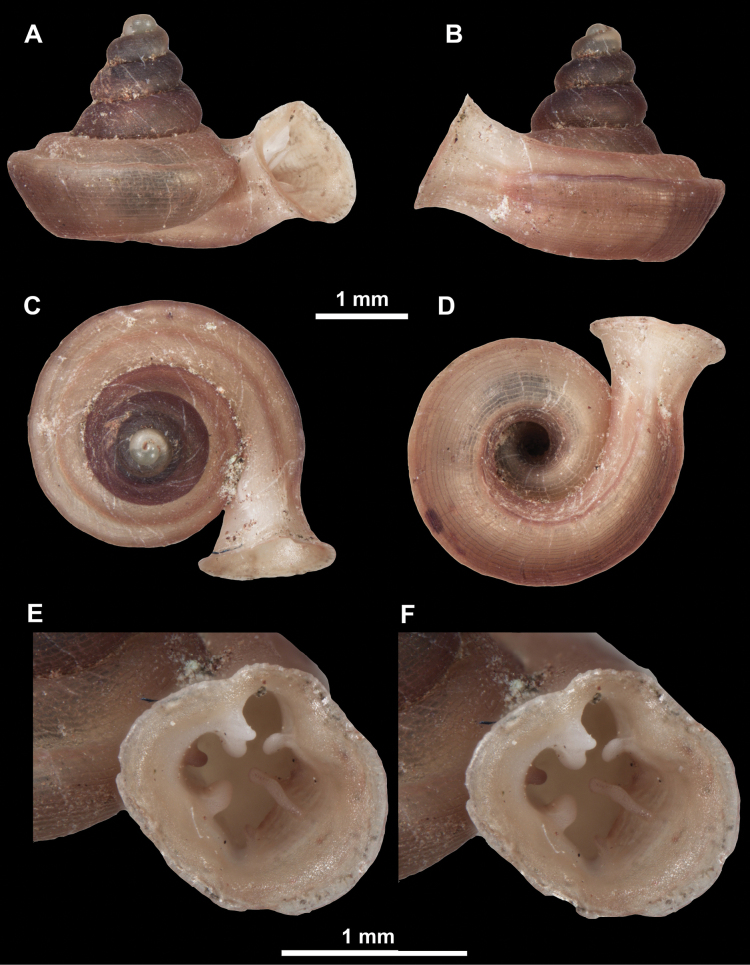
*Hypselostomasororminor* Gojšina, Hunyadi & Páll-Gergely, sp. nov., holotype (CUMZ 14460) **A–D** shell **E, F** enlarged apertural views.

##### Differential diagnosis.

This species differs from *H.discobasis* in the less slender shell and less numerous apertural barriers which are also thicker. It differs from *H.sorormajor* sp. nov. in its smaller dimensions and apertural barrier arrangement: it has less numerous barriers, none of which are hooked (in contrast to *H.sorormajor* sp. nov.). *Hypselostomaaquila* sp. nov. is smaller, more slender, has a narrower umbilicus and hooked barriers.

**Figure 176.**
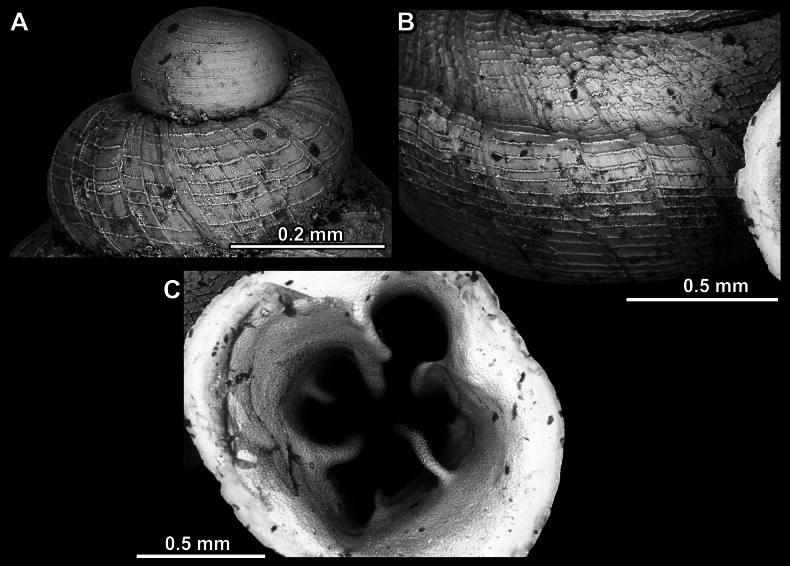
SEM imaging of *Hypselostomasororminor* Gojšina, Hunyadi & Páll-Gergely, sp. nov., holotype (CUMZ 14460) **A** protoconch surface **B** teleoconch surface **C** enlarged apertural view.

##### Measurements

**(in mm, *n* = 5).**SH = 2.37–2.57; SW1 = 3.20–3.61; SW2 = 2.05–2.40; AH = 1.25–1.42; AW = 1.22–1.26.

##### Etymology.

The name of this species is derived from two Latin words, *soror* meaning sister and *minor* meaning smaller which serves as a comparison with similar but usually larger *H. sorormajor* sp. nov.

**Figure 177.**
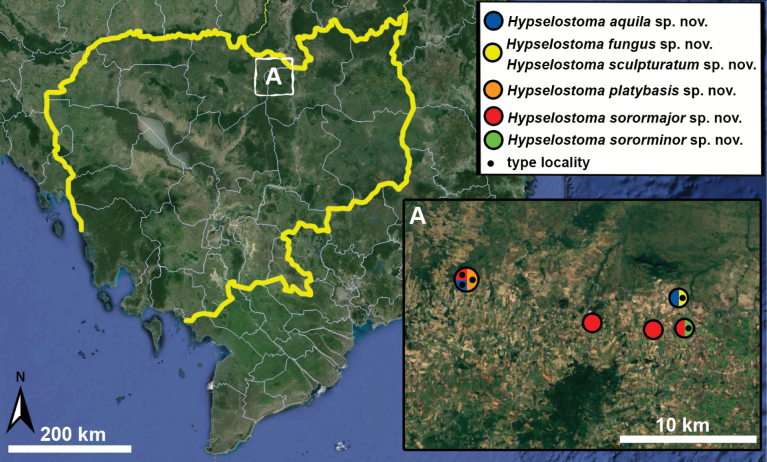
Distribution map of some species belonging to *Hypselostomabensonianum* group in Cambodia.

##### Distribution.

This species is only known from the type locality.

##### Remarks.

The largest, and still unexplored limestone hill in Steung Treng Province (13°47.941'N, 105°43.7671'E) was inaccessible during the collecting efforts in 2023 due to the works of a cement factory. Due to the proximity of this hill to the type locality of *H.sororminor* sp. nov., it is possible that this species can be found here as well and be threatened by quarrying.

#### ﻿ Hypselostoma taehwani

Taxon classificationAnimaliaStylommatophoraHypselostomatidae

Panha & J. B. Burch, 2003

4C91D7CE-8F85-5BCC-9074-56E6CD0C0B92

Hypselostoma taehwani Panha & Burch in Burch et al. 2003: 174–178, figs 16, 17.
Hypselostoma taehwani — Panha and Burch 2008: 101–102, fig. 86.

##### Type material examined.

**Thailand** • 1 paratype; from the type locality; CUMZ 44053.

##### Type locality.

“Central area of Tamrong Temple, Muang Petchburi District, near the main Petchkasem artery of the Asian Hwy. to southern Thailand, Petchaburi Province, 13°01'32"N, 99°55'11"E, 10 meters elevation, Thailand”.

##### Differential diagnosis.

This species is strikingly similar to *H.sculpturatum* sp. nov. They can be separated by the following characters: i) initial teleoconch whorls and the penultimate whorl are more angled in *H.sculpturatum* sp. nov.; ii) umbilicus is wider and has a wider, deeper, and longer umbilical groove; iii) basal plica is in *H.taehwani* situated closer to the columellar lamella but always closer to the lower palatal plica in *H.sculpturatum* sp. nov. Furthermore, the protoconch is spirally striated in *H.taehwani* but pitted in *H.sculpturatum* sp. nov. *Hypselostomadepressum* has a wider umbilicus, much less detached last whorl near the aperture as well as the narrower spire. See also under *H.burchi*.

**Figure 178.**
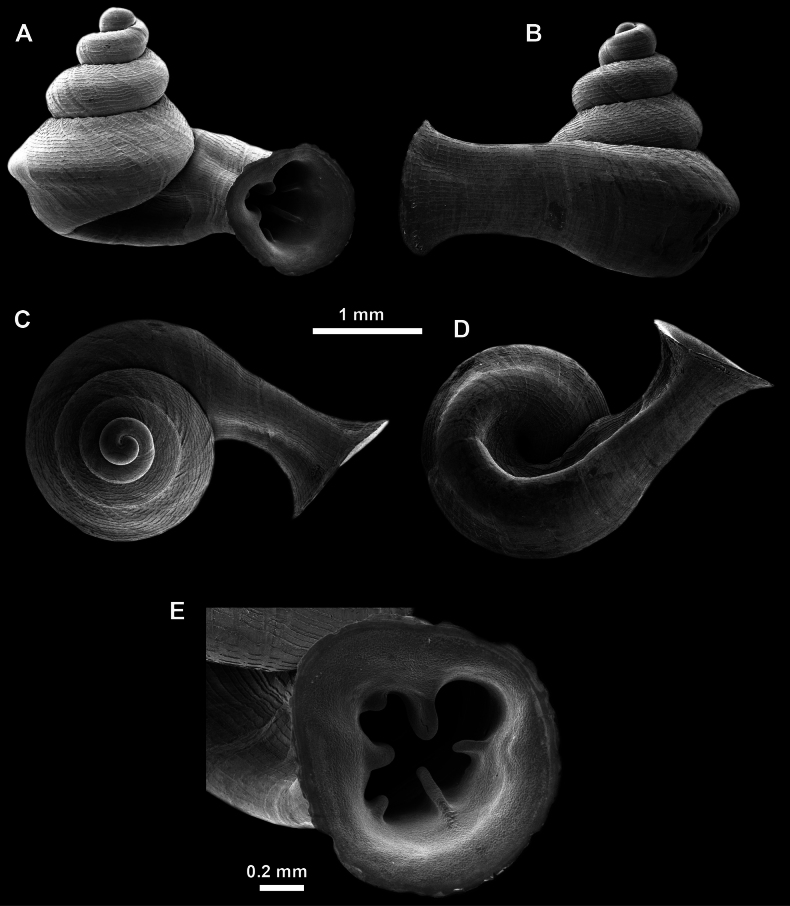
*Hypselostomataehwani*, paratype (CUMZ 44053) **A–D** shell **E** enlarged apertural view.

##### Distribution.

This species is known only from the type locality.

#### ﻿ Hypselostoma torta

Taxon classificationAnimaliaStylommatophoraHypselostomatidae

Gojšina, Auffenberg & Páll-Gergely sp. nov.

EDCFC5D2-D0C2-5AF6-B17E-4596E8649E84

https://zoobank.org/8DBC5B3E-E6A9-40E8-96F1-023952657F47

##### Type material.

***Holotype*. Thailand** • 1 shell (SH: 3.8 mm, SW: 3.5 mm); Nakhon Sawan Province 19.5 m NE intersection Hwys 11 and 32; 15°14'N, 100°16'E; 50 m a.s.l.; 02. May 1988; K. Auffenberg leg.; locality code KA-0696; UF 591362. ***Paratypes*. Thailand** • 2 shells; same data as for holotype; CUMZ 14462 • 20 shells; same data as for holotype; UF 346125 • 18 shells; same data as for holotype; locality code KA-0697; UF 346133 • 4 ethanol-preserved shells; same data as for holotype; locality code KA-0696; UF 591361.

**Figure 179.**
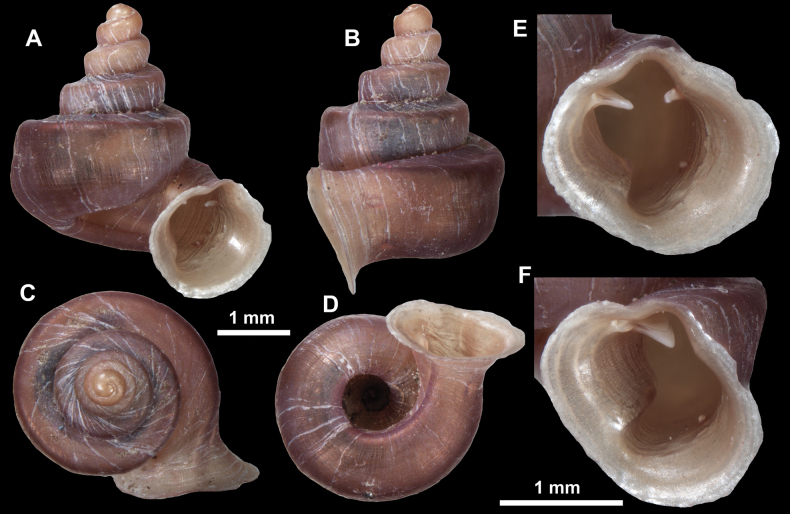
*Hypselostomatorta* Gojšina, Auffenberg & Páll-Gergely, sp. nov., holotype (UF 591362) **A–D** shell **E, F** enlarged apertural views.

##### Additional material examined.

**Thailand** • 5 shells (juveniles, not paratypes); same data as for holotype; locality code KA-0697; UF 583727.

##### Type locality.

Thailand, Nakhon Sawan Province 19.5 m NE intersection Hwys 11 and 32; 15°14'N, 100°16'E; 50 m a.s.l.

##### Diagnosis.

A *Hypselostoma* with all teleoconch whorls shouldered and very weakly spirally striated. Spiral striae not raised. Last whorl detached from the penultimate and slightly descending. Four weak barriers in the aperture. Columellar lamella is like a strong swelling, forming a basal furrow.

**Figure 180.**
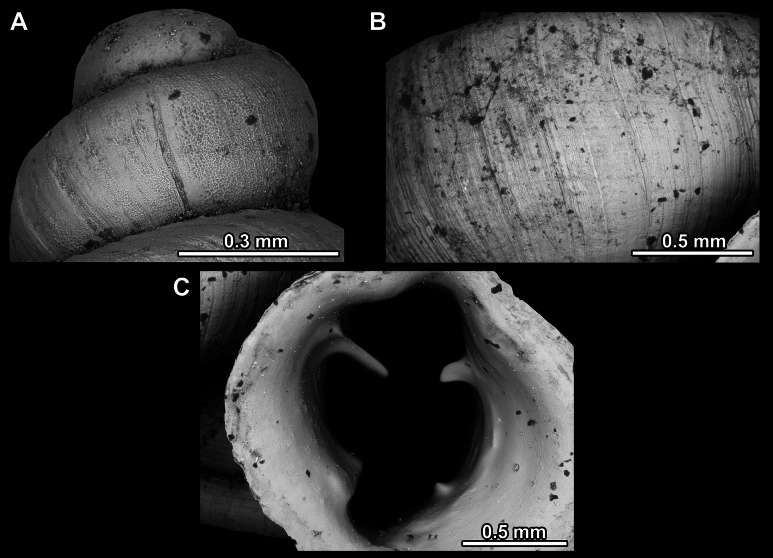
SEM imaging of *Hypselostomatorta* Gojšina, Auffenberg & Páll-Gergely, sp. nov., holotype (UF 591362) **A** protoconch surface **B** teleoconch surface **C** enlarged apertural view.

##### Description.

Shell concave-conical (due to the strongly enlarged last whorl), brownish, consisting of 5–5.5 whorls separated by a very deep suture. Protoconch finely pitted, not showing spiralling pattern, rounded, same colour as the initial teleoconch whorls but slightly lighter than the last whorl. First two teleoconch whorls weakly shouldered, penultimate strongly and the last whorl is very strongly shouldered. The outline of the last and the penultimate whorl above the shoulder is concave (forming a flat platform). This surface is on initial teleoconch whorls more sloping. Whole teleoconch surface is weakly spirally striated (these spiral striae are not thread-like raised as in many congeners, but very blunt and indistinct) and occasionally crossed by radial growth lines and several thicker, whitish radial streaks which are rather unevenly positioned. These whitish streaks seem to be the densest on the penultimate whorl. Last whorl convex at its base, towards the umbilicus. It is slightly to moderately detached from the penultimate whorl and slightly descending near the aperture (~ 15–25 ° compared to the shell axis). Peristome very strong, dirty white, expanded but not reflected. Aperture equipped with three or four barriers (parietal (angulo-parietal?), upper palatal, lower palatal (sometimes absent), and columellar). There are no smaller barriers between the main ones. Parietal lamella is strong, high, and triangular when observed in perpendicular view. There is a very small tubercle-like swelling in front of the parietal lamella which may be homologous with the angular part of the usually present angulo-parietal (or just angular) in other congeners. Upper palatal plica moderately strong, almost twice as weak as the parietal lamella. Lower palatal plica very weak and low, dot-like, sometimes absent. Columellar lamella not present in its usual form (its borders are not clearly delimited) but as a rather strong, swelled part of the aperture which forms a prominent basal furrow below it (similar to the one present in *H.fungus* sp. nov.). The shape of the columellar side of the aperture is also reminiscent of the playground slide. Surface of all apertural barriers is smooth. Sinulus wide, distinctly separated from the rest of the aperture. Umbilicus wide, measuring ~ 1/3 of the shell width, expanding at the last whorl. Inside the umbilicus, there is a strong groove present, stretching across the whole last whorl.

##### Differential diagnosis.

This species is not similar to any of its congeners. It most closely resembles some *Aulacospira* species (e.g., *A.pluangtong*, *A.smaesarnensis*). However, it has a much wider umbilicus than both species as well as spiral striation and way more strongly shouldered whorls.

##### Measurements

**(in mm, *n* = 5).**SH = 3.45–3.8; SW = 2.83–3.62; AH = 1.62–1.82; AW = 1.38–1.84.

##### Etymology.

Since all whorls of this species are shouldered, the shell has an appearance of the wedding cake. The specific epithet *torta* comes from a Serbian word *torta* meaning a cake. To be used as a noun in apposition.

##### Distribution.

This species is known only from the type locality.

##### Remarks.

The shell of this species can sometimes be more depressed so that it is almost as wide as high. This species may belong to a separate genus or it may be closely related to some *Aulacospira* species (*A.pluangtong*, *A.smaesarnensis*). However, due to the different shell shape (concave-conical), height (> 3.4 mm) and surface sculpture (delicate, non-raised spiral striae) from typical *Aulacospira* species (inhabiting the Philippines), we provisionally place this species in the genus *Hypselostoma*.

#### ﻿ Hypselostoma torticollis

Taxon classificationAnimaliaStylommatophoraHypselostomatidae

(van Benthem Jutting, 1962) comb. nov.

B2D28E0D-A127-5D35-B9CD-D2BA3574FFE6

Gyliotrachela torticollis van Benthem Jutting, 1962: 8–10, fig. 4.
Gyliotrachela torticollis — Sutcharit et al. 2023: 1293–1295, fig. 3.

##### Type material examined.

**Cambodia** • 3 paratypes; Battambang, Phuom Truong Mean; 1960; E. Saurin leg.; RMNH.Moll.137136.

##### Type locality.

“Phum Troung Mean, à 15 km au S.-*O. de* Battambang, Cambodge” (Cambodia).

##### Differential diagnosis.

*Hypselostomafungus* sp. nov. has merged angular and parietal lamellae, wider umbilicus, last whorl without a keel and a flatter shell. See also under *H.transitans*, *H.pendulum*, *H.srakeoense*.

##### Distribution.

This species is known only from the Battambang province, Cambodia.

**Figure 181.**
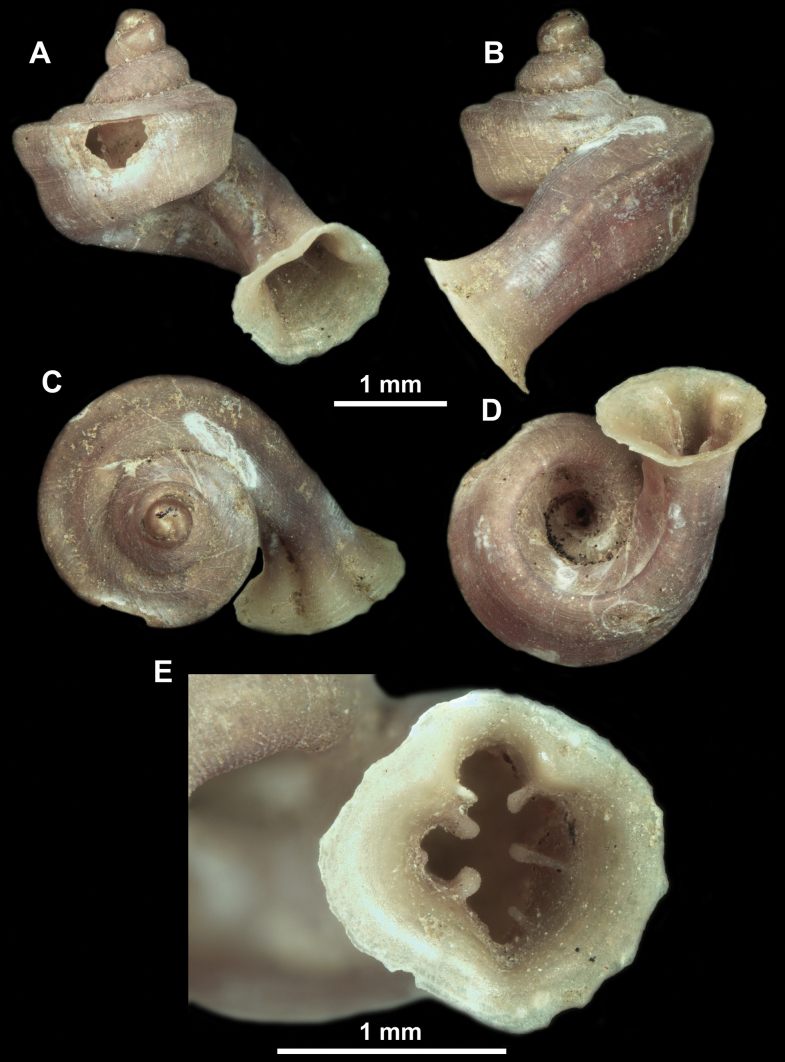
*Hypselostomatorticollis*, paratype (RMNH Moll.137136) (**A, B** and **E** are used from Sutcharit et al. 2023) **A–D** shell **E** enlarged apertural view.

##### Remarks.

Of three examined paratypes, one did not have the aperture as downturned as the others.

**Figure 182.**
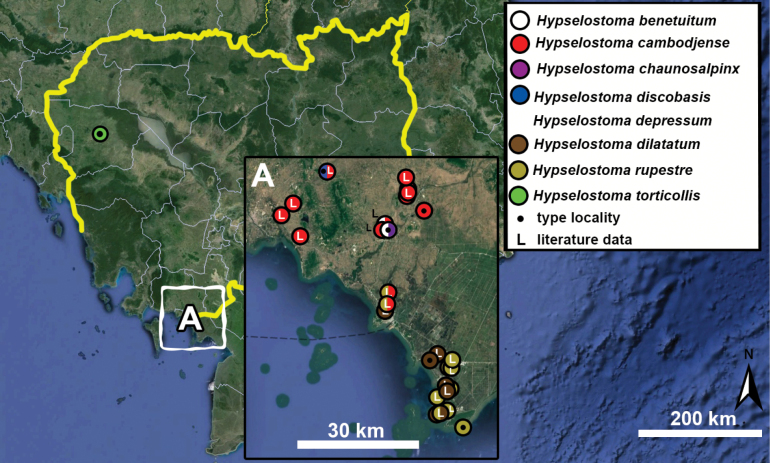
Distribution map of some species belonging to *Hypselostomabensonianum* group in Cambodia.

#### ﻿ Hypselostoma tridentatum

Taxon classificationAnimaliaStylommatophoraHypselostomatidae

(Panha & J.B. Burch, 2004) comb. nov.

3F547692-39FE-575F-881C-38DB805CFDE1

Gyliotrachela tridentatus Panha & Burch in Panha et al. 2004: 70–71, fig. 9.
Gyliotrachela tridentata — Panha and Burch 2008: 86–87, fig. 75.
Gyliotrachela tridentatus — Dumrongrojwattana and Wongkamhaeng 2024: 324, fig. 8.

##### Material examined.

**Thailand** • 2 shells; Srakeo Province, Plubpluengtong, Phloeng Thong Cave, Khao Phiap; 24 Dec. 2012; K. Okubo leg.; coll. PGB. **Cambodia** • Battambang Province, Phnum Proek District, Phnom Prampi Cave; 13°18.834'N, 102°36.490'E; 90 m a.s.l.; 10 Oct. 2023; A. Hunyadi, J.U. Otani leg., coll. HA.

##### Type locality.

“Plubpluengtong limestone hills, Srakeo Province, 13°27'07"N, 102°12'49"E, 80 meters elevation” (Thailand).

##### Differential diagnosis.

This species has fewer barriers in the aperture than *H.khaowongkot* which is the most similar species. It also does not have columellar lamella (present in *H.khaowongkot*) and lacks the strongly spiniferous surface of apertural barriers (also present in *H.khaowongkot*). See also under *H.utongense*.

**Figure 183.**
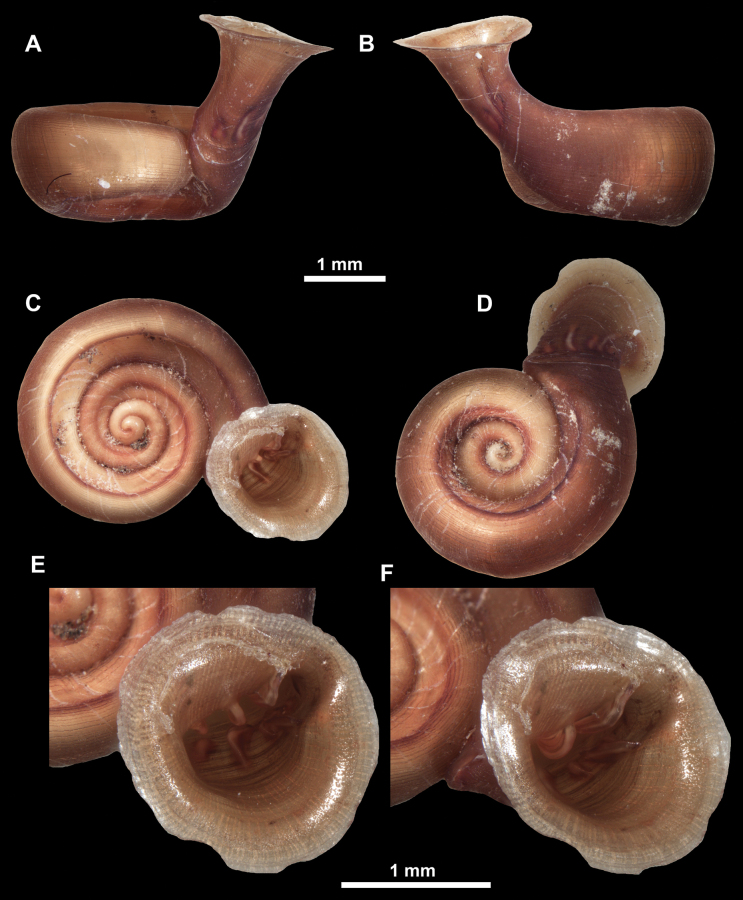
*Hypselostomatridentatum*, specimen from the type locality (coll. PGB) **A–D** shell **E, F** enlarged apertural views.

##### Distribution.

This species is, apart from the type locality, also known from Phnom Prampi Cave in Battambang Province which is a first record from Cambodia.

#### ﻿ Hypselostoma utongense

Taxon classificationAnimaliaStylommatophoraHypselostomatidae

Panha & J. B. Burch, 2004

E032DFB5-6C20-5AAC-9BAD-EEFA5BC7BC80

Hypselostoma utongensis Panha & Burch in Panha et al. 2004: 72–73, fig. 10.
Hypselostoma utongensis — Panha and Burch 2008: 103, fig. 87; Dumrongrojwattana and Wongkamhaeng 2024: 324, fig. 8.

##### Material examined.

**Thailand** • 16 shells; Kanchanaburi Province, Erawan National Park, Erawan Falls Trail; 14°22.310'N, 99°08.699'E; 17 Feb. 2015; A. Hunyadi leg.; coll. HA.

##### Type locality.

“Tam Sua Hill, Utong District, Supanburi Province, 14°34'27"N, 99°46'26"E, 80 meters elevation”.

##### Differential diagnosis.

This species is similar to two *Hypselostoma* which share the completely (or almost completely) flat shell with strongly upturning last whorl (*H.khaowongkot* and *H.tridentatum*). From them, it most obviously differs by its hooked upper and lower palatal plicae which are blunt in the former.

**Figure 184.**
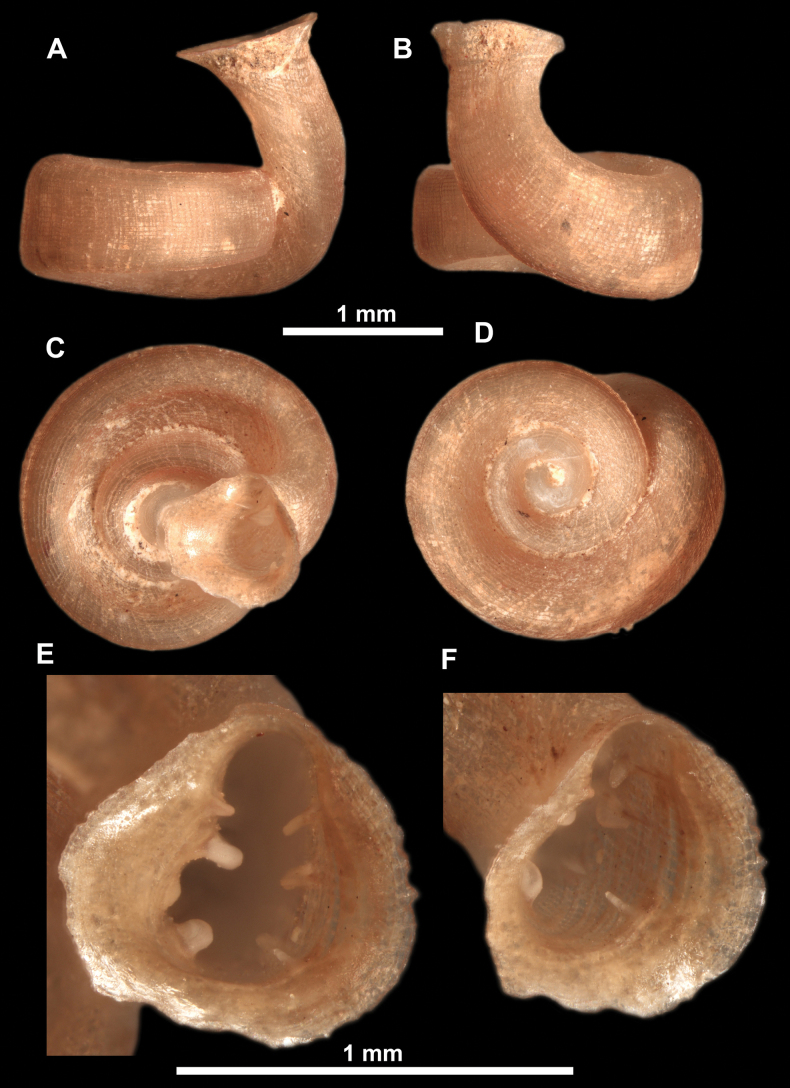
*Hypselostomautongense* from Erawan National Park, Kanchanaburi province (coll. HA) **A–D** shell **E, F** enlarged apertural views.

##### Distribution.

This species is known from two provinces in Thailand, Supanburi (type locality) and Kanchanaburi (this study).

##### Remarks.

In some specimens, previous whorls are partly sunken into the last while in others completely.

###### ﻿2. *Hypselostomahungerfordianum* group

**Diagnosis.***Hypselostomahungerfordianum* group is characterised by a peculiar shell surface sculpture, as if “scaly, granulated”. This surface sculpture is unique and we assume that all the species which share this trait are possibly more closely related.

**Remarks.** This group has representatives across Southeast Asia and includes 19 species. The distribution spans from Kayin State in Myanmar and eastwards to western Cambodia (Battambang Province). Southernmost localities are known in Peninsular Malaysia while northernmost come from Loei Province in Thailand.

**Figure 185.**
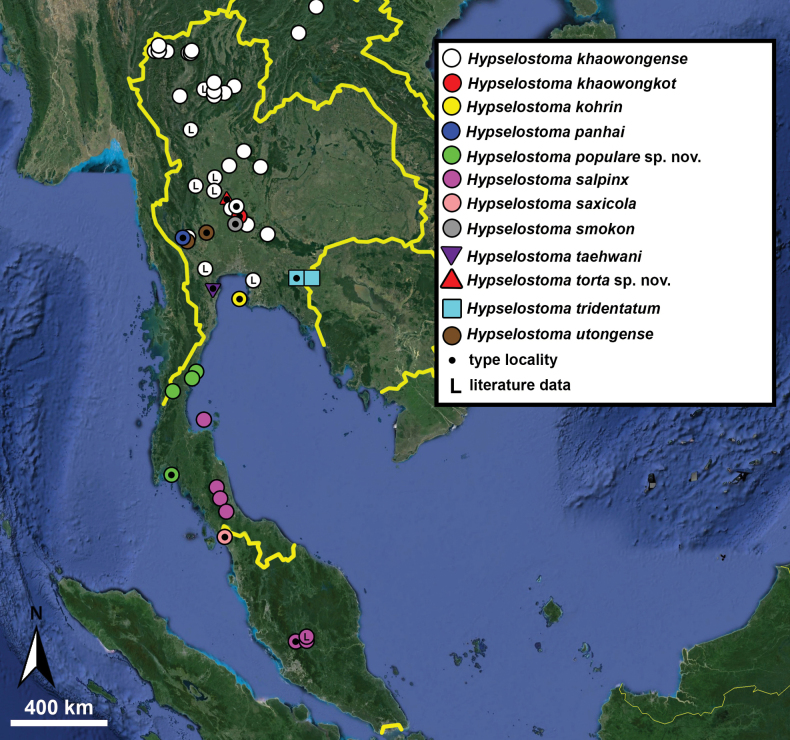
Distribution map of some species belonging to *Hypselostomabensonianum* group.

#### ﻿ Hypselostoma aunglini

Taxon classificationAnimaliaStylommatophoraHypselostomatidae

(Tongkerd & Panha, 2024) comb. nov.

4CB784AA-75FB-58F1-ADD3-E08328FCC5AE

**Figure 186.**
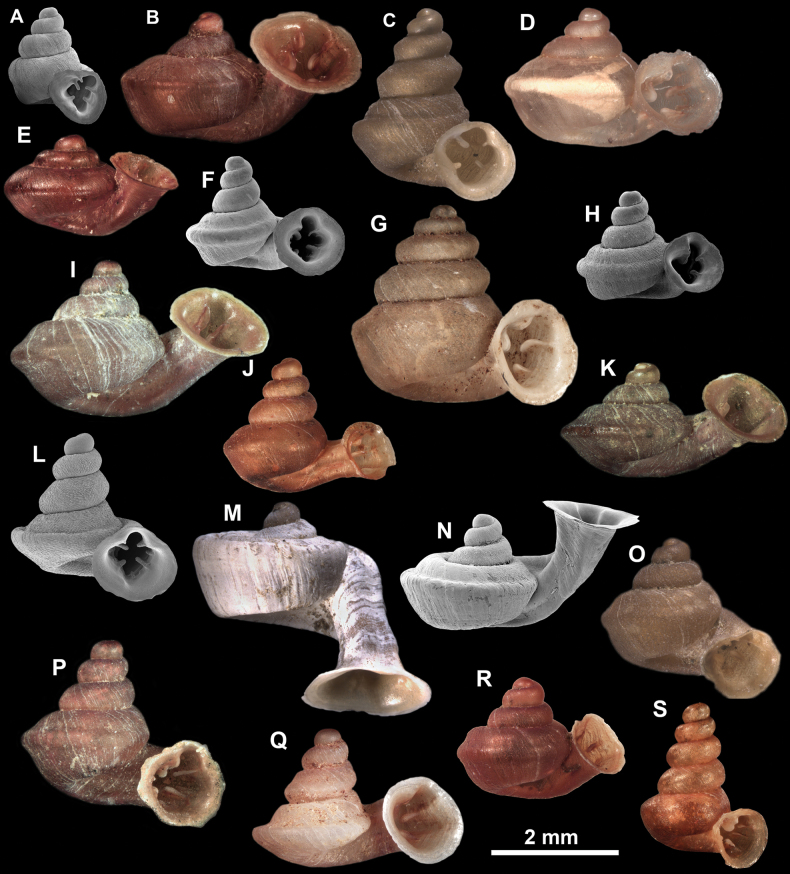
Synoptic view of species belonging to *Hypselostomahungerfordianum* group **A***H.aunglini***B***H.bubalus* sp. nov. **C***H.chatnareeae***D***H.coriaceum* sp. nov. **E***H.fortunatum* sp. nov. **F***H.hungerfordianum***G***H.khmerianum***H***H.loei***I***H.luctans***J***H.ophis* sp. nov. **K***H.piconis***L***H.sichang***M***H.srakeoense***N***H.surakiti***O***H.transitans***P***H.venustum***Q***H.vesovici* sp. nov. **R***H.vicinum* sp. nov. **S***H.vujici* sp. nov.

**Figure 187.**
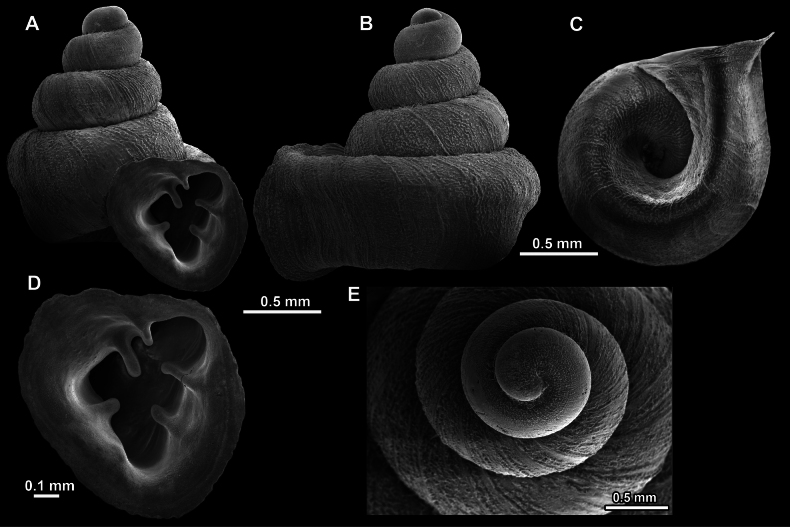
*Hypselostomaaunglini*, holotype (CUMZ 14383) (from Tongkerd et al. 2024) **A–C** shell **D** enlarged apertural view **E** enlarged view of the protoconch.

Figs 186A , 187 , 223

Gyliotrachela aunglini Tongkerd & Panha in Tongkerd et al. 2024: 184–187, figs 12, 13P.

##### Type material examined.

**Myanmar** • holotype; collector unknown; CUMZ 14383.

##### Type locality.

“Kaw Gon Cave, Hpa-An, Kayin State, Myanmar (…; 16°49'22.2"N, 97°35'08.9"E)”.

##### Differential diagnosis.

The most similar congener from Myanmar is *H.bensonianum* which is however spirally striated (granulated in *H.aunglini*) and has its last whorl keeled (shouldered in *H.aunglini*).

##### Distribution.

This species is known only from the type locality.

#### ﻿ Hypselostoma bubalus

Taxon classificationAnimaliaStylommatophoraHypselostomatidae

Gojšina, Hunyadi & Páll-Gergely sp. nov.

22793DE1-34D1-595F-82D6-D1F19C6CE6D9

https://zoobank.org/826C711E-9D22-4472-99C8-A37DD0FDF0A7

**Figure 188.**
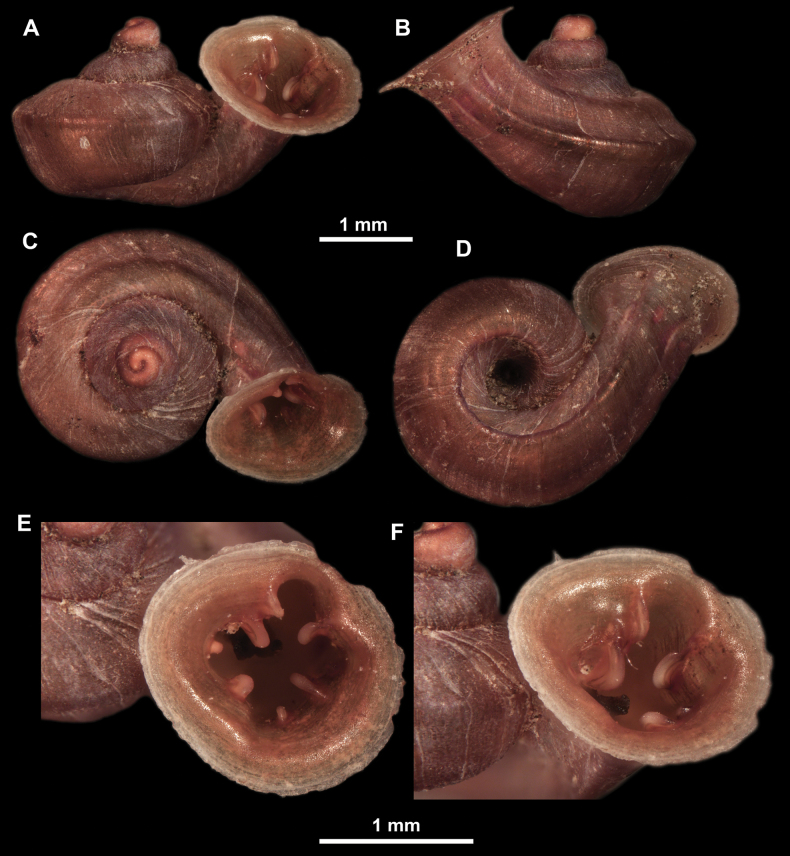
*Hypselostomabubalus* Gojšina, Hunyadi & Páll-Gergely, sp. nov., holotype (CUMZ 14466) **A–D** shell **E, F** enlarged apertural views.

**Figure 189.**
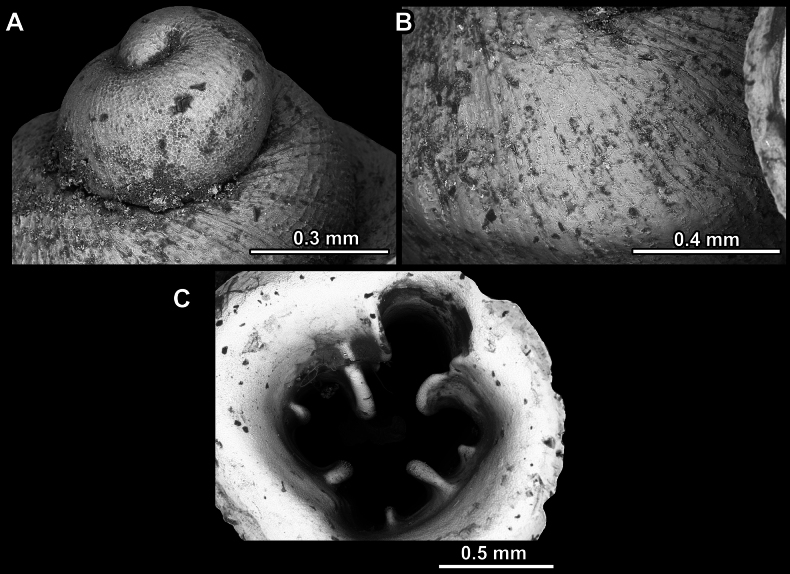
SEM imaging of *Hypselostomabubalus* Gojšina, Hunyadi & Páll-Gergely, sp. nov., holotype (CUMZ 14466) **A** protoconch surface **B** teleoconch surface **C** enlarged apertural view.

Figs 186B , 188 , 189 , 223

##### Type material.

***Holotype*. Malaysia** • 1 shell (SH: 2 mm; SW: 3.7 mm); Kedah, 1.5 km east from Kodiang, Gua Kerbau; 06°23.431'N, 100°18.904'E, 14 Jan. 2013; A. Hunyadi leg.; CUMZ 14466.

***Paratypes*. Malaysia** • 216 shells; same data as for holotype; coll. HA • 1 shell; same data as for holotype; coll. VG.

##### Additional material examined.

**Malaysia** • 25 shells (damaged, not paratypes); same data as for holotype; coll. HA.

##### Type locality.

Malaysia, Kedah, 1.5 km east from Kodiang, Gua Kerbau; 06°23.431'N, 100°18.904'E.

##### Diagnosis.

Shell depressed, concave-conical. Teleoconch finely radially and spirally striated (localised, just below and above the keel on the last whorl). Last whorl detached from the penultimate and strongly ascending. Aperture wide, equipped with five main barriers (angular, parietal, upper palatal, lower palatal and columellar) and two smaller barriers (basal and infraparietal). Umbilicus wide, measuring 50% of the shell width.

##### Description.

Shell depressed, concave-conical, chestnut brown, very weakly glossy and not translucent. Whorls 3.5–4, initially weakly convex, separated by a deep suture, regularly increasing until the last whorl which is strongly enlarged and flat. Protoconch lighter than the rest of the shell, finely pitted without spiralling pattern, consisting of ~ 1.25 whorls. Teleoconch very finely granulated (sandpaper-like surface) and radially striated with additional whitish radial streaks which are weak, widely, and irregularly spaced. Spiral striae present but localised, clearly visible only on the last whorl above and below the keel, elsewhere absent. Sometimes, spiral striae are very difficult to observe on the whole shell. Last whorl strongly enlarged in comparison to previous ones, bluntly keeled very slightly above the centre of the periphery (or sometimes at the centre of the periphery) and almost flat above and weakly convex below the keel. Keel is strongest near the aperture. Last whorl moderately to strongly ascending near the aperture (~ 50–65 ° compared to the shell axis) and moderately detached from the penultimate whorl. Peristome very pale brownish, strongly expanded but not reflected. Aperture equipped with five main barriers (angular, parietal, upper palatal, lower palatal, and columellar). Angular lamella short and small, directed towards the upper palatal plica. Parietal lamella strong and high, not much longer than the angular and only reaching its profile. Upper palatal plica slightly weaker than the parietal lamella and bent towards the lower palatal plica. Lower palatal plica straight, equally strong as the upper palatal but not bent. Columellar lamella is the broadest in the aperture but relatively short. Between these main barriers, additional small basal and infraparietal plicae visible. Surface of all apertural barriers is very finely spiniferous medially and very finely granulated laterally. Sinulus small and distinctly separated from the rest of the aperture. Umbilicus very wide, measuring half of the shell width and showing all previous whorls. A deep and wide groove is running from the columellar side of the peristome alongside the umbilicus.

##### Differential diagnosis.

See under *H.luctans*.

##### Measurements

**(in mm, *n* = 5).**SH = 2–2.23; SW1 = 3.40–4.13; SW2 = 1.95–2.41; AH = 1.44–1.78; AW = 1.33–1.61.

##### Etymology.

The type locality of this species, Gua Kerbau, means “Buffalo cave” in Malay. The specific epithet *bubalus* is also a scientific name for a buffalo, which is to be used as a noun in apposition.

##### Distribution.

This species is known only from the type locality.

##### Remarks.

Although this species shows some spiral striation, it is placed into *H.hungerfordianum* group because of the predominant sandpaper-like surface on the teleoconch.

#### ﻿ Hypselostoma chatnareeae

Taxon classificationAnimaliaStylommatophoraHypselostomatidae

(Panha & J. B. Burch, 2003) comb. nov.

D8BFAE57-1B8E-536B-9614-63946671F915

**Figure 190.**
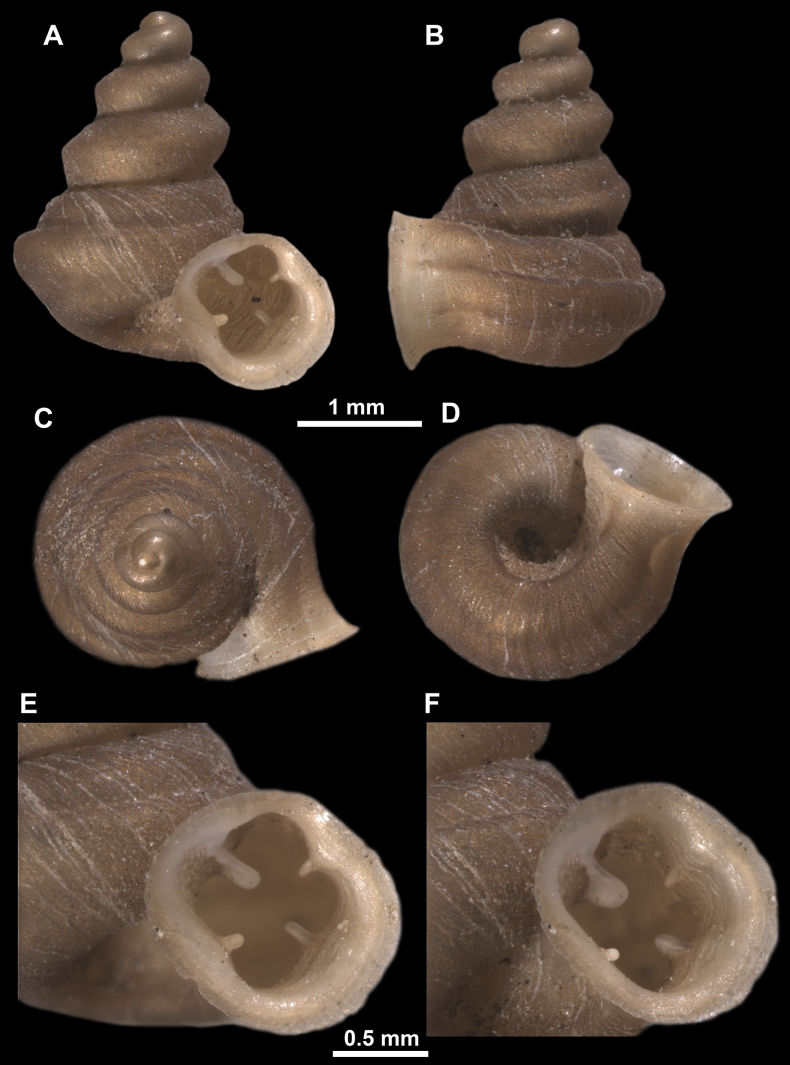
*Hypselostomachatnareeae*, paratype (SMF 331447) **A–D** shell **E, F** enlarged apertural views.

Figs 186C , 190 , 223

Anauchen chatnaeerae Panha & Burch in Burch et al. 2003: 132–138, figs 2, 3.
Anauchen chatnaeerae — Tongkerd et al. 2004: 143, fig. 2; Panha and Burch 2008: 49–50, fig. 45; Dumrongrojwattana and Wongkamhaeng 2024: 323, fig. 7.

##### Type material examined.

**Thailand** • 3 paratypes; from the type locality; SMF 331447.

##### Additional material examined.

**Thailand** • 1 shell; Tam Sua Hill, Utong, Suphanburi; 21 Dec. 2002; K. Okubo leg.; coll. PGB.

##### Type locality.

“Tam Sua, on the limestone hill on the west side of Tam Sua Temple, Utong District, Suphanburi Province, 14°17'31"N, 100°7'31"E, 20 meters elevation, Thailand”.

##### Differential diagnosis.

This species is not similar to any of its congeners. The shell shape (conical) and apertural dentition (one barrier on the parietal side) strongly resembles some *Anauchen* species (e.g., *A.angthongensis*) but the surface is granulated as in *H.hungerfordianum* and all whorls are keeled. See also under *H.khmerianum*.

##### Distribution.

Apart from the type locality, this species is also known from one more nearby locality.

##### Remarks.

Even though this species has high morphological similarities to some *Anauchen* species (e.g., *A.angthongensis*), the shell surface sculpture is characteristically granulated (as in *H.hungerfordianum*) which is the reason it is transferred to this genus.

#### ﻿ Hypselostoma coriaceum

Taxon classificationAnimaliaStylommatophoraHypselostomatidae

Gojšina & Páll-Gergely sp. nov.

6625028B-8286-5989-8D58-D5F30D7FF4F1

https://zoobank.org/FF1C3C65-2BFF-4E84-9CD2-2807DFCE8F34

**Figure 191.**
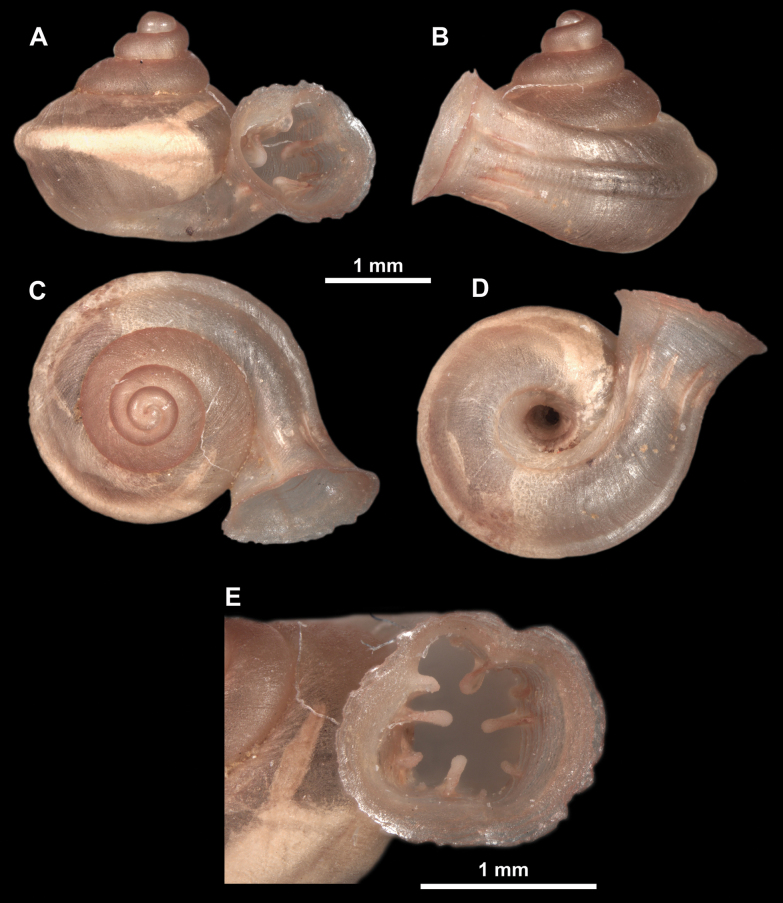
*Hypselostomacoriaceum* Gojšina & Páll-Gergely, sp. nov., holotype (CUMZ 14464) **A–D** shell **E** enlarged apertural view.

**Figure 192.**
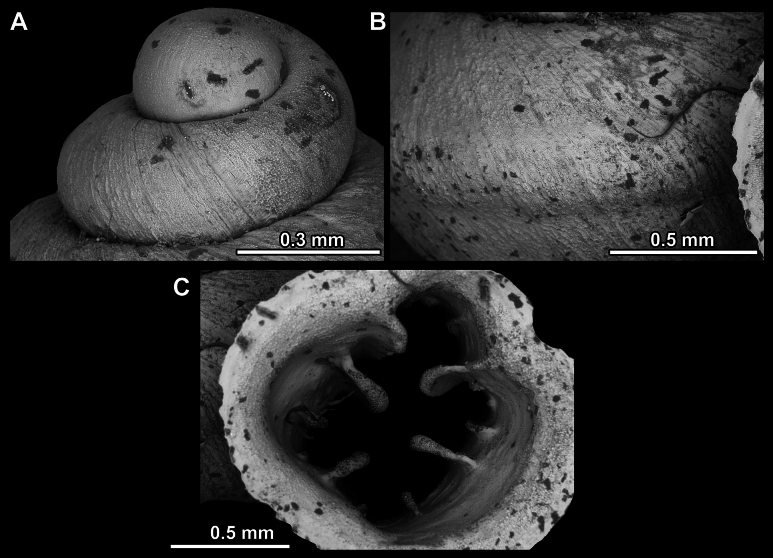
SEM imaging of *Hypselostomacoriaceum* Gojšina & Páll-Gergely, sp. nov., holotype (CUMZ 14464) **A** protoconch surface **B** teleoconch surface **C** enlarged apertural view.

Figs 186D , 191 , 192 , 223

##### Type material.

***Holotype*. Malaysia** • 1 shell (SH: 2.11 mm; SW1: 3.35 mm); Langkawi Island, Tanjung Rhu; 23 Aug. 1998; Hemmen, Wiesbaden ex. coll.; CUMZ 14464. ***Paratypes*. Malaysia** • 30 shells; same data as for holotype; coll. PGB • 1 shell; same data as for holotype; coll. VG • 1 shell; same data as for holotype; coll. HA • 4 shells; Kedah, limestone outcrop near Tanjung Rhu, Langkawi Island; 6°26′51.48″N, 99°48′38.89″E; 16. May 2009; M. E. Marzuki leg.; coll. PGB.

##### Type locality.

Malaysia, Langkawi Island, Tanjung Rhu.

##### Diagnosis.

Shell depressed-conical, yellowish, and glossy. Last whorl bluntly keeled. Aperture detached from the penultimate whorl and slightly ascending upwards. Teleoconch finely granulated and radially striated. Spiral striation absent. Aperture equipped with five main strong barriers (parietal, upper and lower palatal, basal, and columellar) and additional smaller one of variable number.

##### Description.

The yellowish, glossy, and weakly translucent, depressed-conical shell is consisting of 4–4.5 whorls separated by a deep suture. The finely pitted protoconch is showing a weak spiralling pattern, consisting of ~ 1.5 whorls without a clear boundary with teleoconch. Teleoconch finely granulated, sandpaper-like (surface microsculpture similar to *H.hungerfordianum*) and inconspicuously regularly radially striated. Last whorl bluntly keeled, slightly detached from the penultimate and slightly to moderately ascending upwards (~ 25–35 ° compared to the shell axis). Peristome expanded and not reflected, with sharp edges. Aperture equipped with five main barriers (parietal, angular, upper palatal, lower palatal, and columellar) and variable number of smaller ones. Angular lamella is the weakest among the main five, the rest of the main barriers are developed roughly to the same level. They are all high and straight. Parietal and angular lamellae are directed towards the palatal wall. In front of the upper palatal plica, a small swelling is present which is probably homologous with the palatal tubercle present in other genera (e.g., *Bensonella*, see Páll-Gergely and White 2023). Between the angular lamella and upper palatal plica, one or two additional plicae are present. There are also usually two interpalatal plicae, two between lower palatal and columellar (including basal plica) and two between the columellar and parietal lamellae. None of these smaller barriers reach the profiles of the main ones, and none of the apertural barriers reach the peristome. Surface of all apertural barriers is very finely spiniferous medially and very finely granulated laterally. Sinulus parabolic and not very strongly separated from the rest of the aperture. Umbilicus open, initially narrow, and then abruptly widening at the last whorl, measuring ~ ¼ of the shell width and showing the penultimate whorl. A strong groove can be observed running from the peristome towards the inner side of the umbilicus.

##### Differential diagnosis.

This species is larger than both *H.salpinx* and *H.saxicola* and it additionally differs from them by the absence of spiral striation and a narrower umbilicus.

##### Measurements

. SH = 2.05–2.32; SW1 = 3.12–3.37; SW2 = 1.85–2.09; AH = 1.33–1.58; AW = 1.28–1.40.

##### Etymology.

The specific epithet is due to its “leathery” surface sculpture.

##### Distribution.

Known only from Tanjung Rhu, Langkawi Island, Malaysia.

#### ﻿ Hypselostoma fortunatum

Taxon classificationAnimaliaStylommatophoraHypselostomatidae

Gojšina, Hunyadi & Páll-Gergely sp. nov.

F491D1DE-91CC-5D1A-9CD3-0E7865390DA9

https://zoobank.org/73754B02-33DC-44E1-8447-66187845B533

**Figure 193.**
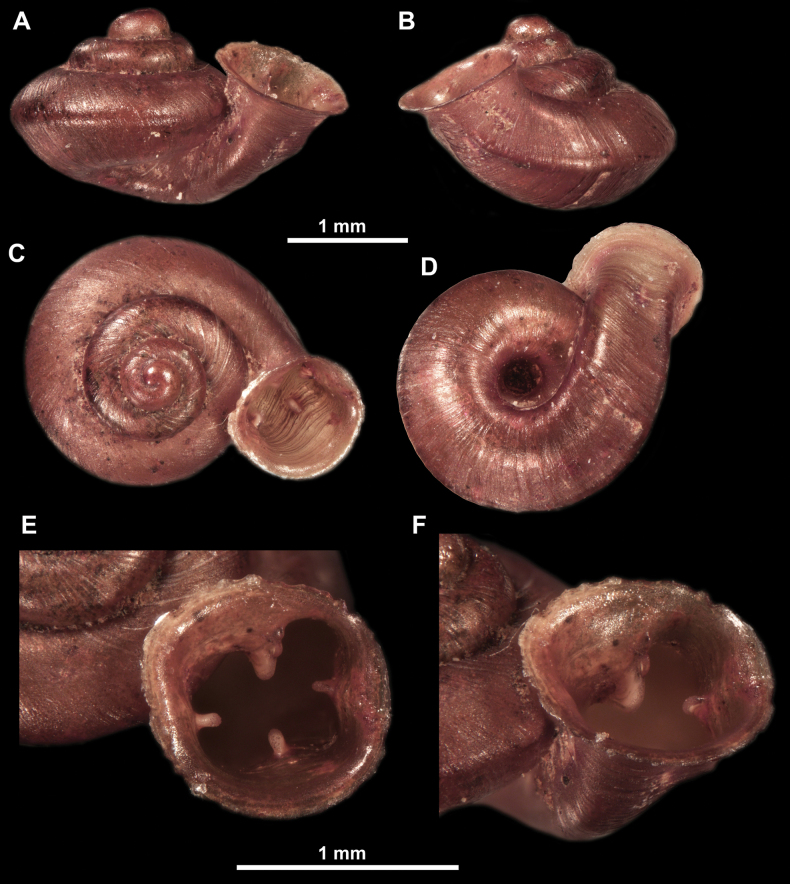
*Hypselostomafortunatum* Gojšina, Hunyadi & Páll-Gergely, sp. nov., holotype (CUMZ 14465) **A–D** shell **E, F** enlarged apertural views.

**Figure 194.**
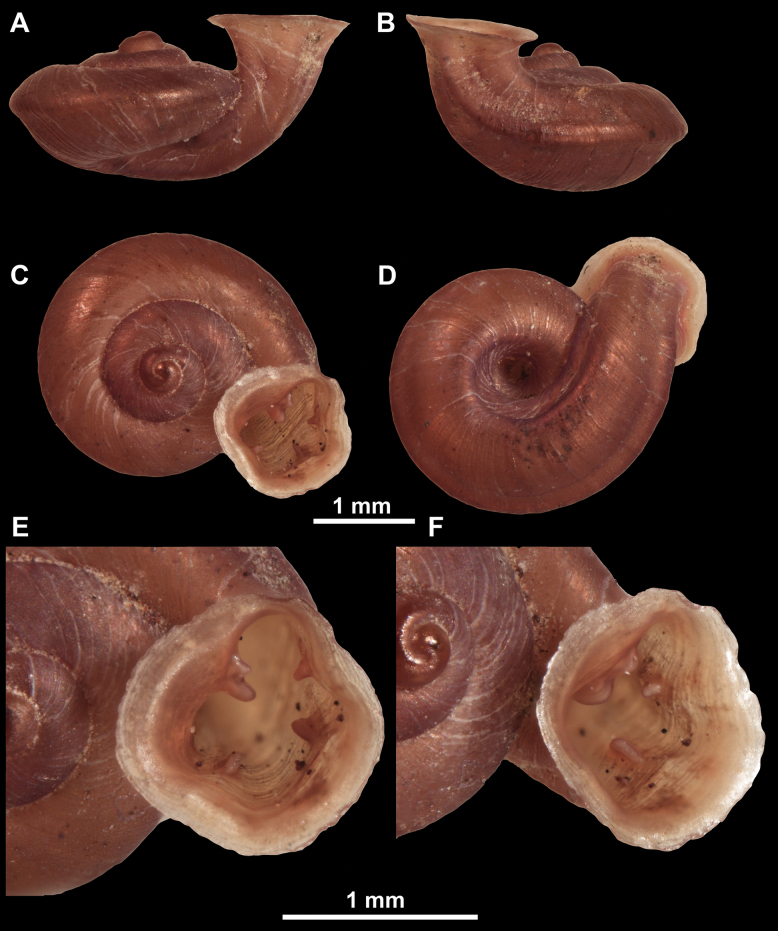
*Hypselostomafortunatum* Gojšina, Hunyadi & Páll-Gergely, sp. nov., a strongly depressed form (UF 344932) **A–D** shell E, **F** enlarged apertural views.

**Figure 195.**
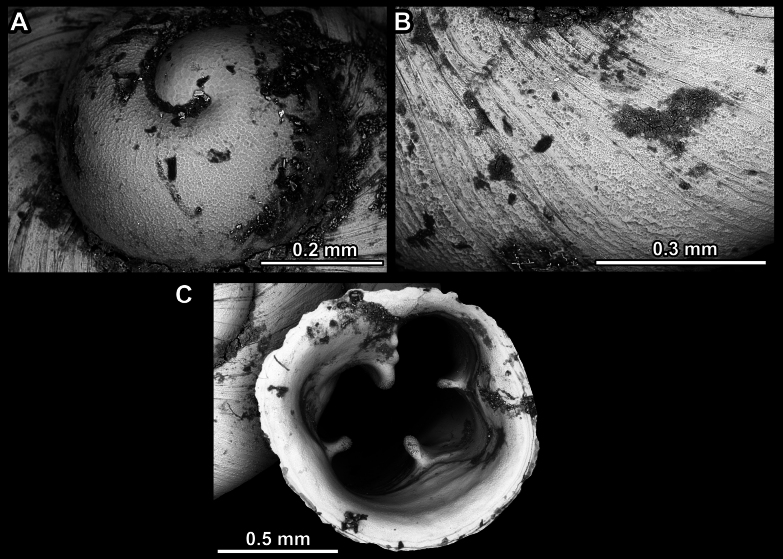
SEM imaging of *Hypselostomafortunatum* Gojšina, Hunyadi & Páll-Gergely, sp. nov., holotype (CUMZ 14465) **A** protoconch surface **B** teleoconch surface **C** enlarged apertural view.

Figs 186E , 193 , 194 , 195 , 223

##### Type material.

***Holotype*. Thailand** • 1 shell (SH: 1.57 mm; SW1: 2.83 mm); Phattalung Province, Khao Ok Thalu, rock wall; 07°37.506'N 100°05.330'E; 19 Feb. 2015; A. Hunyadi leg.; CUMZ 14465. ***Paratypes*. Thailand** • 20 shells; same data as for holotype; coll. HA.

##### Additional material examined.

**Thailand** • 6 shells (juveniles/damaged, not paratypes); same data as for holotype; coll. HA • 11 shells; Songkhla Province, 31.3 km NW Hat Yai, 1.2 km W of Hwy. 43; 7°10'N, 100°16'E; 80 m a.s.l.; 08 Apr. 1988; K. Auffenberg leg.; locality code KA-0636A; UF 344932 • 1 shell; same locality data as previous; 08 Apr. 1988; K. Auffenberg leg.; locality code KA-0635; UF 591324.

##### Type locality.

Thailand, Phattalung Province, Khao Ok Thalu, rock wall; 07°37.506'N, 100°05.330'E.

##### Diagnosis.

*Hypselostoma* with depressed-conical shell, teleoconch with finely sandpaper-like surface and radially but not spirally striated. Aperture equipped with only four relatively weak barriers (angulo-parietal, upper palatal, lower palatal and columellar).

##### Description.

Shell depressed-conical, chestnut brown, glossy, not translucent. It is consisting of 3.5–4 regularly increasing, convex whorls separated by a deep suture. Protoconch of the same colour as the teleoconch, finely pitted, not showing spiralling pattern and consisting of ~ 1.25 whorls. Teleoconch sculpture finely granulated (sandpaper-like) with additional radial growth lines which are more or less regularly spaced. Last whorl bluntly keeled at the centre of the periphery, slightly to moderately detached from the penultimate whorl and strongly ascending near the aperture (usually ranging from 65 to almost 90 ° compared to the shell axis). The keel is the most prominent on the last whorl behind the peristome. Peristome brown, weakly expanded, not reflected. Aperture equipped with four main barriers (angulo-parietal, upper palatal, lower palatal and columellar), all of which are relatively weak. Angulo-parietal lamella is the strongest in the aperture, consisting of a high parietal part and weak, tubercle-like angular part which is sometimes weakly sinuated in the middle. The angular part is sometimes also more distinctly separated from the parietal. Upper palatal plica relatively weak and straight. Lower palatal plica only slightly stronger (broader and higher) than the upper palatal. Columellar lamella horizontal, developed to the same extent as the lower palatal plica. Weak basal plica and infraparietal lamella sometimes present. All barriers weaker than in the majority of the congeners, all finely spiniferous medially but indistinctly granulated to almost smoot laterally. Sinulus wide and not strongly separated from the rest of the aperture. Umbilicus moderately wide to wide, measuring ~ 1/3–1/5 of the shell width and clearly showing the penultimate whorl. A relatively shallow groove runs towards the inner side of the umbilicus, terminating at the penultimate whorl.

##### Differential diagnosis.

See under *H.pattalungense* and *H.depressispira*.

##### Measurements

**(in mm, *n* = 5).**SH = 1.46–1.58; SW1 = 2.67–2.90; SW2 = 1.74–1.92; AH = 1.06–1.26; AW = 1.05–1.23.

##### Etymology.

The shape of the aperture, between the barriers, resembles a four-leaf clover which is a common symbol of luck, hence the specific epithet which is derived from the Latin word for lucky (*fortunatus*).

##### Distribution.

This species is known from Phattalung and Songkhla provinces (S Thailand).

##### Remarks.

This species is present in one more distinct form superficially resembling *H.depressispira*. Namely, in this form the spire is much more depressed and the umbilicus is noticeably wider. We decided not to treat this form a separate species since other characters (apertural dentition, general shell shape, and its surface sculpture) are fully shared with the “typical form” of *H.fortunatum* sp. nov.

#### ﻿ Hypselostoma hungerfordianum

Taxon classificationAnimaliaStylommatophoraHypselostomatidae

Möllendorff, 1891

FEDD6A30-FC4F-5729-BA22-BE5005C13FA0

**Figure 196.**
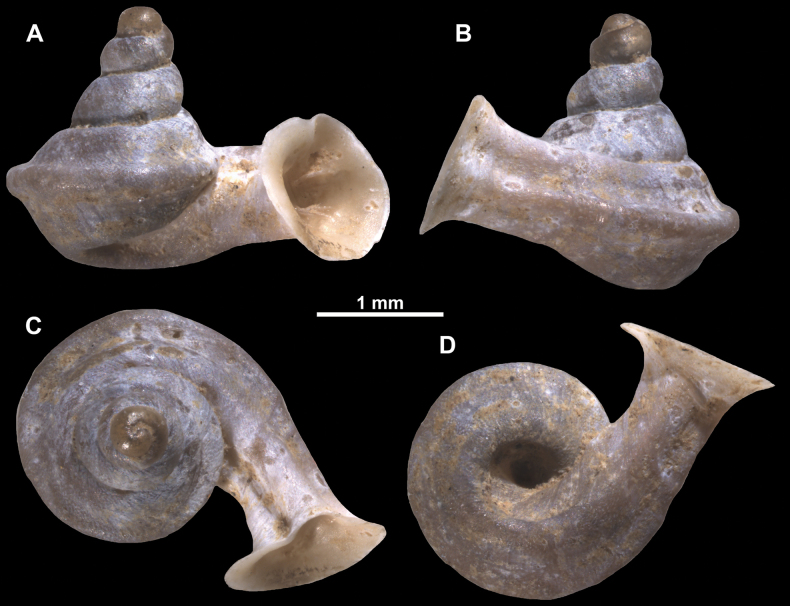
*Hypselostomahungerfordianum*, lectotype (SMF 4587) **A–D** shell.

**Figure 197.**
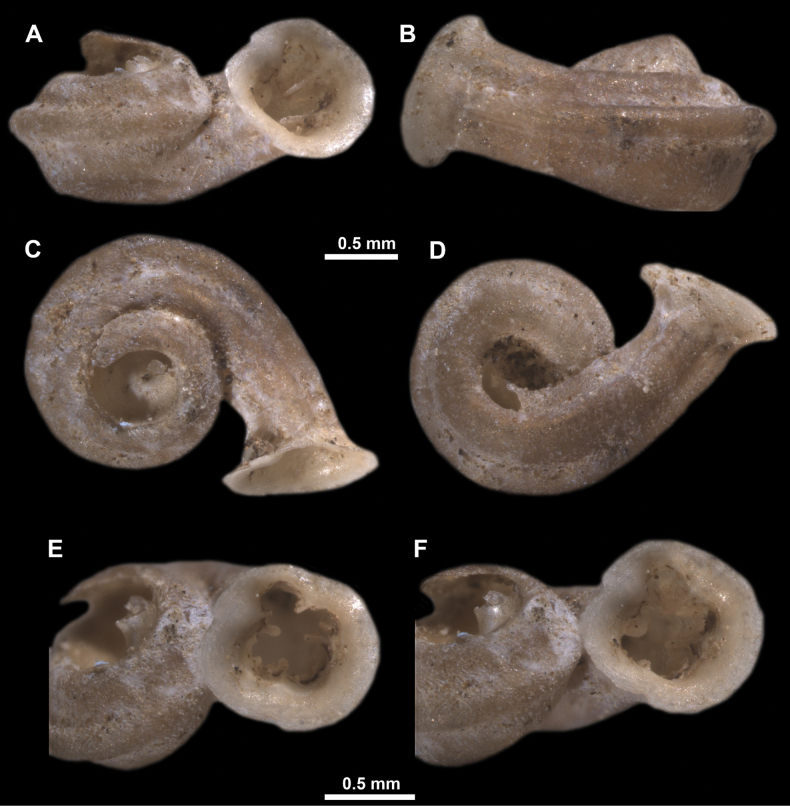
*Hypselostomahungerfordianum*, holotype of *H.striolatum* (SMF 4591) **A–D** shell **E, F** enlarged apertural views.

**Figure 198.**
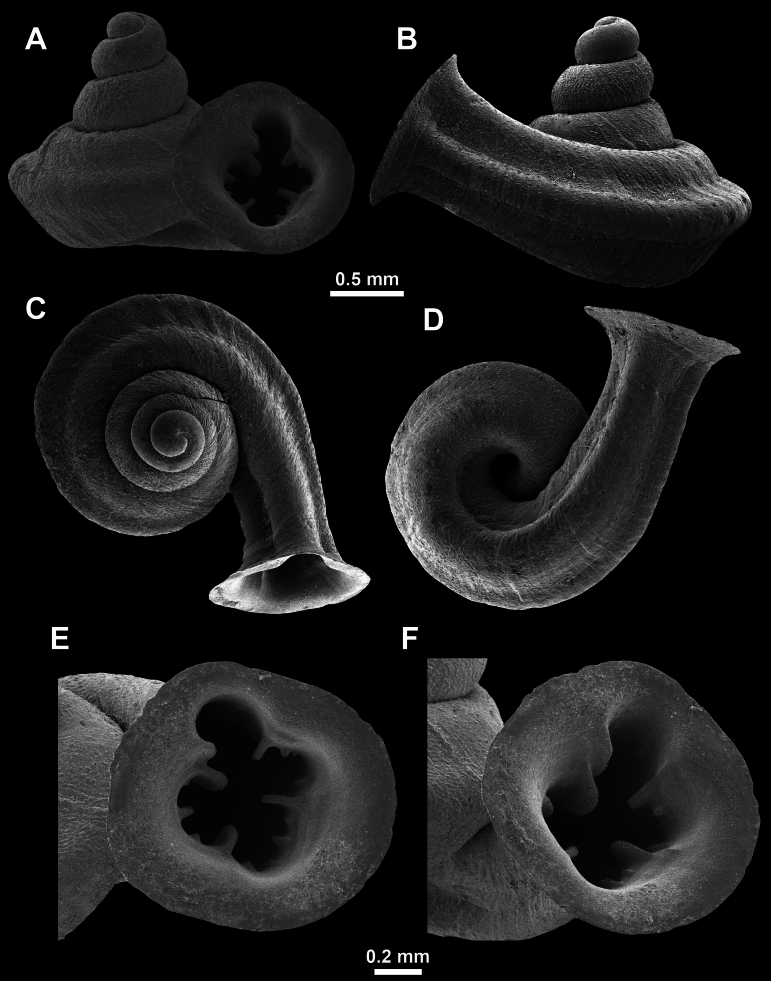
*Hypselostomahungerfordianum*, holotype of *H.satulensis* (CUMZ ver. 44028) **A–D** shell **E, F** enlarged apertural views.

**Figure 199.**
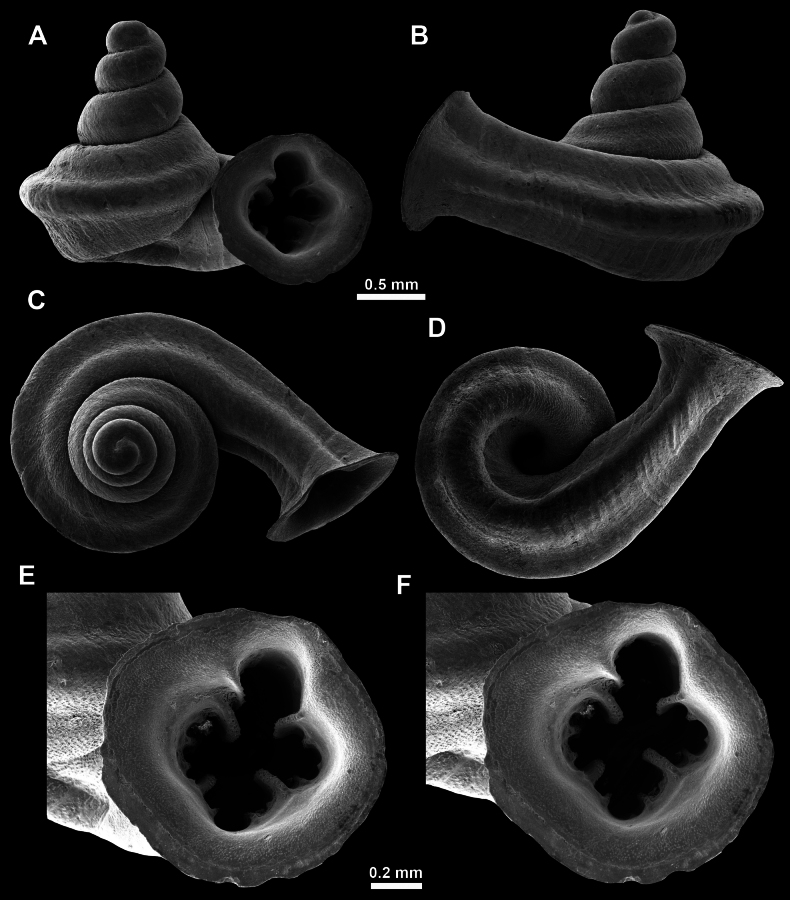
*Hypselostomahungerfordianum*, paratype of *G.khaochongensis* (CUMZ ver. 010) **A–D** shell **E, F** enlarged apertural views.

**Figure 200.**
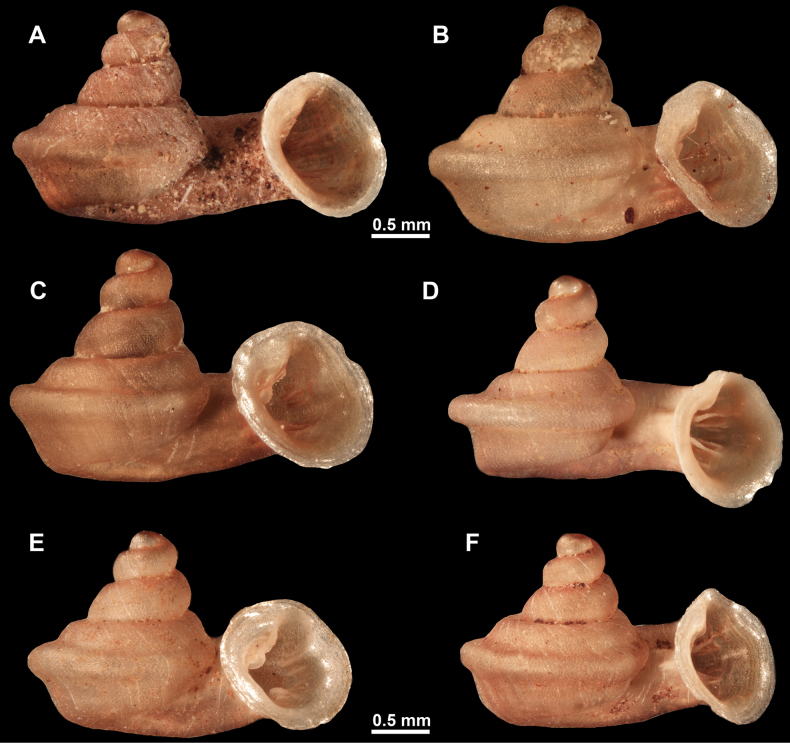
*Hypselostomahungerfordianum* from different localities **A** Malaysia, Kelantan province (coll. HA) **B** Malaysia, Pahang province (coll. HA) **C** Malaysia, Kuala Lumpur (coll. HA) **D** Thailand, Krabi province (coll. HA) **E** Thailand, Nakhon Si Thammarat provinve (coll. HA) **F** Malaysia, Perlis province (coll. HA).

**Figure 201.**
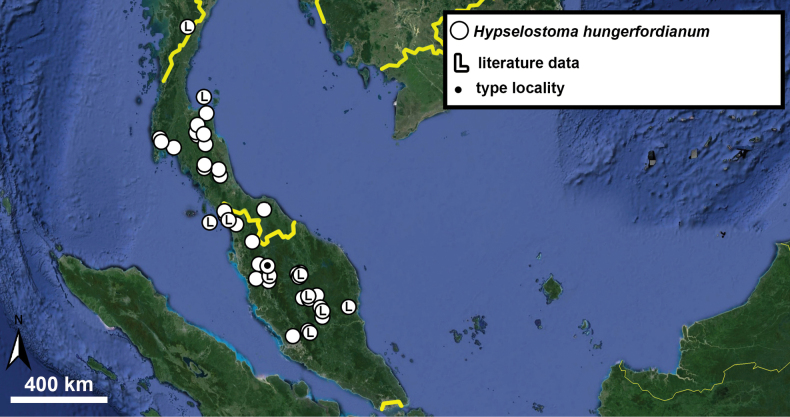
Distribution map of *Hypselostomahungerfordianum*.

Figs 186F , 196 , 197 , 198 , 199 , 200 , 201

Hypselostoma hungerfordianum Möllendorff, 1891: 337, pl. 30, figs 7, 7a.
Hypselostoma striolatum Möllendorff, 1894: 152. syn. nov.
Hypselostoma hungerfordianum — Sykes 1902: 61.
Hypselostoma striolatum — Fischer and Dautzenberg 1904: 408.
Gyliauchen hungerfordianus — Pilsbry 1917: 212, pl. 36, figs 1–4.
Gyliauchen striolatus — Pilsbry 1917: 215.
Gliotrachela hungerfordiana [sic] — Laidlaw 1933: 214.
Gliotrachela striolatus [sic] — Laidlaw 1933: 214.
Gyliotrachela hungerfordiana — van Benthem Jutting 1949b: 60; van Benthem Jutting 1950: 46; van Benthem Jutting 1950: 26; Zilch 1959: 164, fig. 563; van Benthem Jutting 1960: 14; Zilch 1984: 166; Davison 1995: 239; Schileyko 1998: 142, fig. 162; Maassen 1999: 122–123; Schilthuizen 1999: 283; Maassen 2001: 75–76; Foon et al. 2017: 79, fig. 30B; Hoekstra and Schilthuizen 2011: 267–272, fig. 2; Foon and Marzuki 2023: 142, fig. 1AP; Tongkerd et al. 2024: 180–182, figs 10, 13M.
Gyliotrachela striolata — van Benthem Jutting 1950: 46; Zilch 1984: 166; Thompson and Upatham 1997: 233; Hemmen and Hemmen 2001: 41.
Gyliotrachela khaochongensis Panha in Panha 1998a: 53–56, fig. 2.
Gyliotrachela khaochongensis — Hemmen and Hemmen 2001: 41; Panha and Burch 2008: 70–71, fig. 61. Tongkerd et al. 2024: 180, fig. 10B.
Hypselostoma satulensis Panha & Burch in Panha et al. 2004: 75–76, fig. 12. syn. nov.
Hypselostoma satulensis — Panha and Burch 2008: 98–99, fig. 84; Dumrongrojwattana and Wongkamhaeng 2024: 324, fig. 8.
Gyliotrachela striolatus [sic] — Panha and Burch 2008: 81–82, fig. 71.
Gyliotrachela phoca Tongkerd & Panha, 2013 in Tongkerd et al. 2013: 71–75, figs 5–7.
Gyliotrachela phoca — Tongkerd et al. 2024: 180. Dumrongrojwattana and Wongkamhaeng 2024: 324, fig. 8.

##### Type material examined.

**Malaysia** • lectotype of *H.hungerfordianum*; SMF 4587. **Thailand** • holotype of *H.striolatum*; SMF 4591 • holotype of *H.satulensis*; S. Panha leg.; CUMZ ver. 44028; paratype of *G.khaochongensis*; from the type locality; CUMZ ver. 010.

##### Additional material examined.

**Thailand-South** • 6 shells; Suratthani Province, 6 km S of Na San, limestone mountain; 8.660°N, 99.398°E; 03 June 1987; F. Thompson leg.; locality code FGT-4311; UF 530647 • 2 shells; Suratthani Province, Hwy. 401, 2.7 km W junction Hwy. 4142 and 401, evergreen forest on rocky hillside; 9°10'N, 99°40'E; 90 m a.s.l.; 19 Apr. 1988; K. Auffenberg leg.; locality code KA-0674; UF 591325 • 34 shells; Suratthani Province, 15.3 km NE Ban Na San off road to Tha Rom Yen National Park; 8°53'N, 99°26'E; 40 m a.s.l.; 20 Apr. 1988; K. Auffenberg leg.; locality code KA-0678; UF 345386 • 12 shells; Suratthani Province, 1 km NE of Na San, 100 m limestone mountain; 8.812°N, 99.382°E; 02 June 1987; F.G. Thompson leg.; locality code FGT-4309; UF 547905 • 3 shells; Suratthani Province, Angthong islands, Wia Talap island, steep path to Buaboke cave, at the base of limestone rocks; 8°7.603'N, 98°55.466'E; 50 m a.s.l.; Sep. 2007; A. Reischütz leg.; coll. REI • 1 shell; Suratthani Province, 15.3 km NE Ban Na San off road to Tha Rom Yen National Park; 8°53'N, 99°26'E; 40 m a.s.l.; 20 Apr. 1988; K. Auffenberg leg.; locality code KA-0678; UF 591326 • 3 shells; Phangnga Province, 32 km NE Khok Kloi, Wat Tham Suwan Khuha; 08°27'N, 98°27'E; 20 m a.s.l.; 27 Mar. 1988; K. Auffenberg leg.; locality code KA-0601; UF 345751 • 1 shell; Phangnga Province, limestone knoll, 5.7 km SW intersection Hwys 4 & 4152; 8°27'N, 98°29'E; 20 m a.s.l.; 28 Mar. 1988; K. Auffenberg leg.; locality code KA-0602; UF 345771 • 1 shell; Krabi Province, 5.6 km SW of Ao Luek Tai, on uneven surface of large boulder at the base of limestone cliff; 8°20.772'N, 98°40.968'E; 20–25 m a.s.l.; 16 Mar. 2020; ex. coll. S. Aiken; coll. HA • 8 shells; Krabi Province, 9.2 km northwest from Phang Nga; 08°07.305'N, 98°52.231'E; 40 m a.s.l.; 21 Feb. 2015; A. Hunyadi leg.; coll. HA • 3 shells; Krabi Province, Viewpoint Hill, Railay (=Rai Leh) Beach West, at the base of limestone rocks; 8°0.511'N, 98°50.248'E; 40 m a.s.l.; Sep. 2007; A. Reischütz leg.; coll. REI • 4 shells; Nakhon Si Thammarat Province, Lan Saka, N side of Hwy. 4015; 08°25'N, 99°45'E; 16 Apr. 1988; K. Auffenberg leg.; locality code KA-0665; UF 345226 • 8 shells; Nakhon Si Thammarat Province, circa 38 km WNW of Tha Sala, outcrop 1.5 km W of junction Hwys 4186 and 4188; 8°43'N, 99°47'E; 80 m a.s.l.; 16 Apr. 1988; K. Auffenberg leg.; locality code KA-0661; UF 345194 • 16 shells; Nakhon Si Thammarat Province, Wat Tham Thong Panara, vicinity of the rock temple; 08°25.278'N, 99°22.762'E; 19 Feb. 2015; A. Hunyadi leg.; coll. HA • 23 shells; Nakhon Si Thammarat Province, circa 38 km WNW of Tha Sala, outcrop 1.5 km W of junction Hwys 4186 and 4188; 08°43'N, 99°47'E; 80 m a.s.l.; 16 Apr. 1998; K. Auffenberg leg.; locality code KA-0661; UF 345193 • 20 shells; Phattalung Province, 6 km NW of Phattalung, 1 km NE of Hwy. 4048; 7°40'N, 100°3'E; 60 m a.s.l.; 13 Apr. 1988; K. Auffenberg leg.; locality code KA-0653; UF 345102 • 54 shells; Phatthalung Province, Khao Pu-Khao Ya Nat. Park, Si Banphot district, Ruesi Cave; 7°41.797'N, 99°50.162'E; 25 Feb. 2023; A. Hunyadi, K. Okubo & J.U. Otani leg.; coll. PGB • 25 shells; Phattalung Province, 6.4 km E of Hwy. 4 on Hwy. 4081, 1 km SW of Khao Chai Son; 7°27'N, 100°11'E; 50 m a.s.l.; 11 Apr. 1988; K. Auffenberg leg.; locality code KA-0648; UF 345063 • 14 shells; Phattalung Province, 6.4 km E of Hwy. 4 on Hwy. 4081, 1 km SW of Khao Chai Son; 7°27'N, 100°11E; 11 Apr. 1988; K. Auffenberg leg.; locality code KA-0648; UF 345064 • 7 shells; Phattalung Province, 16.2 km S of junction of Hwy.s 4 and 4122; 7°25'N, 99°59'E; 150 m a.s.l.; 09 Apr. 1988; K. Auffenberg leg.; locality code KA-0637; UF 344971 • 4 shells; Phattalung Province, 6 km NW of Phattalung, 1 km NE of Hwy. 404; 7°40.02'N, 100°3'E; 60 m a.s.l.; 13 Apr. 1988; K. Auffenberg leg.; locality code KA-0653; UF 436278 • 18 shells; Phattalung Province, 15.1 km W Hwy. 41 on Hwy. 4164, Khaopu and Khaoya National Park; 7°47'N, 99°56'E; 160 m a.s.l.; 14 Apr. 1988; K. Auffenberg leg.; locality code KA-0657; UF 345154 • 6 shells; Phattalung Province, 6.4 km E Hwy. 4 on Hwy. 4081, 1 km SW Khao Chai Son; 7°27'N, 100°10.98'E; 11 Apr. 1988; K. Auffenberg leg.; locality code KA-0649; UF 345073 • 17 shells; Phattalung Province, 16.2 km S of junction of Hwys 4 and 4122; 7°25'N, 99°59'E; 150 m a.s.l.; 09 Apr. 1988; K. Auffenberg leg.; locality code KA-0637; UF 344970 • 3 shells; Phattalung Province, Khao Pu Khao Ya National Park, vicinity of Matcha cave; 7°39.980'N, 99°52.343'E; 125 m a.s.l.; 25 Feb. 2023; A. Hunyadi leg.; coll. HA • 23 shells; Phattalung Province, Khao Pu Khao Ya National Park, Si Banphot district, ca 6 km north-northwest from Taphaen; 7°40.738'N, 99°50.822'E; 85 m a.s.l.; 24 Feb. 2023; A. Hunyadi, K. Okubo & J.U. Otani leg.; coll. HA • 1 shell; Yala Province, 6.3 km W of Yala, Ban Na Tham, Wat Khuha Phi Muk; 6°33'N, 101°15'E; 40 m a.s.l.; 01 Apr. 1988; K. Auffenberg leg.; locality code KA-0612; UF 344665 • 3 shells; Yala Province, 6.3 km W of Yala, Ban Na Tham, Wat Khuha Phi Muk; 6°33'N, 101°15'E; 40 m a.s.l.; 01 Apr. 1988; K. Auffenberg leg.; locality code KA-0612; UF 344664 • 25 shells; Yala Province, Mueang Yala district, Na Tham, Silp Cave (~ 9 km from centre of Yala); 6°31.316'N. 101°13.982'E; 21 Feb. 2023; A. Hunyadi, K. Okubo & J.U. Otani leg.; coll. HA. **Malaysia** • 2 shells; Selangor, Templer Park, Bukit Takun; NNW of Kuala Lumpur; Nov. 1998; ex. coll. Hemmen; coll. PGB • 46 shells; Kedah, Baling, eastern side of Bukit Baling; 55 m a.s.l.; 05°40.575'N, 100°54.803'E; 11 Jan. 2013; A. Hunyadi leg.; coll. HA • 9 shells; Kelantan, Gua Musang, environment of the cave entrance; 04°52.974'N, 101°58.116'E; 135 m a.s.l.; 17 Jan. 2013; A. Hunyadi leg.; coll. HA • 7 shells; Perlis, Kaki Bukit, vicinity of Gua Kelam; 06°38.674'N, 100°12.170'E; 45–55 m a.s.l.; 13 Jan. 2013; A. Hunyadi leg.; coll. HA • 11 shells; Pahang, 15 km west from Bandar Pusat Jengka, Hutan Lipur Gunung Senyum; 03°41.862'N, 102°25.980'E; 85 m a.s.l.; 23 Jan. 2013; A. Hunyadi leg.; coll. HA • 3 shells; Perak, 800 m north from Padang Rengas on the road no. A107, eastern side of Gunung Pondok; 04°47.195'N, 100°50.493'E; 90 m a.s.l.; 08 Jan. 2013; A. Hunyadi leg.; coll. HA • 12 shells; Kelantan, Gua Musang, 6 km Pulai, Gua Madu, vicinity of the cave; 04°50.213'N, 101°56.982'E; 120 m a.s.l.; 17 Jan. 2013; A. Hunyadi leg.; coll. HA • 3 shells; Selangor, Kuala Lumpur E - Kanching, Templer Park, Perangsang Templer Golf Club, rock; 03°17.878'N, 101°38.438'E; 100 m a.s.l.; 25 Jan. 2013; A. Hunyadi leg.; coll. HA • 22 shells; Perak, Lenggong, Taman Awam Gua Tok Giring; 05°07.128'N, 100°.351'E; 90 m a.s.l.; 06 Jan. 2013; A. Hunyadi leg.; coll. HA • 131 shells; Perak, northeast from Chemor, 2 km east from Kampung Kanthan, western side of the mountain; 04°44.799'N, 101°07.794'E; 105 m a.s.l.; 08 Jan. 2013; A. Hunyadi leg.; coll. HA • 304 shells; Pahang, Bukit Cinta Manis, south-southeastern side, 800 m from Lebuhraya Karak towards Kampung Cinta Manis; 03°26.714'N, 102°00.814'E; 130 m a.s.l.; 22 Jan. 2013; A. Hunyadi leg.; coll. HA • 18 shells; Perlis, Kangar, Bukit Kaya/Bukit Lagi, northeastern edge of the mountain; 06°26.076'N, 100°11.469'E; 5 m a.s.l.; 12 Jan. 2013; A. Hunyadi leg.; coll. HA • 67 shells; Pahang, Gua Bama, Kuala Lipis 9 km, Padang Tungku; 04°11.652'N, 101°57.936'E; 120 m a.s.l.; 19 Jan. 2013; A. Hunyadi leg.; coll. HA • 180 shells; Pahang, 20 km southeast from Jerantut, Gua Kota Gelanggi, below Gua Balai; 03°54.000'N, 102°28.412'E; 115 m a.s.l.; 21 Jan. 2013; A. Hunyadi leg.; coll. HA • 4 shells; Kelantan Province, Gua Musang, behind railway station; 04°53.033'N, 101°58.178'E; J.U. Otani leg.; coll. PGB • 6 shells; Pahang Province, Lipis, Kenong Rimba park; 04°12.952'N, 102°11.263'E; 83 m a.s.l.; J.U. Otani leg.; coll. PGB • 2 shells; Pahang Province, Jerantut, Taman Negara, Kuala Tahan, Gua Luas; 04°30.640'N, 102°26.548'E; 75 m a.s.l.; J.U. Otani leg.; coll. PGB • 1 shell; Pahang Province, Jerantut, Kg. Baheru, Gua Kota Gelanggi, Gua Terang Bulan; J.U. Otani leg.; coll. PGB • 8 shells; Kedah, 1.5 km east from Kodiang, Gua Kerbau; 06°23.431'N, 100°18.904'E, 14 Jan. 2013; A. Hunyadi leg.; coll. HA • 5 shells; Kelantan Province, Gua Musang, 264 km from KL, Route 8; 191 m a.s.l.; 04°46.685'N, 102°00.095'E; J.U. Otani leg.; coll. PGB • 5 shells; Selangor, Templer Park, Bukit Takun; NNW of Kuala Lumpur; Nov. 1999; J. Hemmen leg.; coll. PGB • 5 shells; Kelantan Province, Gua Madu, south of Gua Musang; 04°52.405'N, 101°57.925'E; 02 Nov. 2000; J. Hemmen leg.; coll. PGB • 8 shells; Pahang, Kota Gelanggi, Jengka-Jerantut road, on limestone surfaces covered with mosses; 03°53.4758'N, 102°28.388'E; 29. May 2011; M. E. Marzuki leg.; coll. PGB.

##### Type localities.

“Perak” (Bukit Pondong, Malaysia) (*H.hungerfordianum*); “Kuankalong Limestone Hill, Satul Province, 6°52'59"N, 100°07'38"E, 110 meters elevation”, Thailand (*H.satulensis*); “Samui islands, Gulf of Siam”, Thailand (*H.striolatum*).

##### Differential diagnosis.

See under *H.sichang* and *H.loei*.

##### Distribution.

This species is widely spread in Malaysia and adjacent regions of Thailand. Also known from Myanmar where the northernmost locality of the species is known (Tongkerd et al. 2024). The southernmost localities are positioned in and near the city of Kuala Lumpur (Malaysia).

##### Remarks.

This species can have a more or less elongated spire. The umbilicus is usually consistent in its width but we have observed slight variations. The apertural dentition is variable in terms of the number of smaller barriers between the five main ones (angular, parietal, upper palatal, lower palatal and columellar). *Gyliotrachelastriolata* was known only from a single damaged type, which retained its last whorl only. Even in damaged condition, it was clear that there are no differences between *G.striolata* and *H.hungerfordianum* which is why they are treated conspecific. *Hypselostomasatulensis* was described from Satun province in S Thailand and shows no morphological differences from *H.hungerfordianum*. Its type locality is practically located in the middle of the distribution area of *H.hungerfordianum*. We have examined the material from northern Thailand hosted in UF with the following data: 2 shells; Mae Hong Son Province, Tham Lod Cave, 1.5 km SE Ban Tham Lod Village; 19°34'N, 98°9'E; 720 m a.s.l.; 19 Mar. 1988; K. Auffenberg leg.; locality code KA-0576; UF 345527. Since this locality is quite far from the actual distribution area of this species, we believe that this sample was mislabelled and that this species actually does not occur in Mae Hong Son Province.

#### ﻿ Hypselostoma khmerianum

Taxon classificationAnimaliaStylommatophoraHypselostomatidae

(Sutcharit & Panha, 2023) comb. nov.

68759A08-9ECD-5694-B83A-BEF1F1366DBD

**Figure 202.**
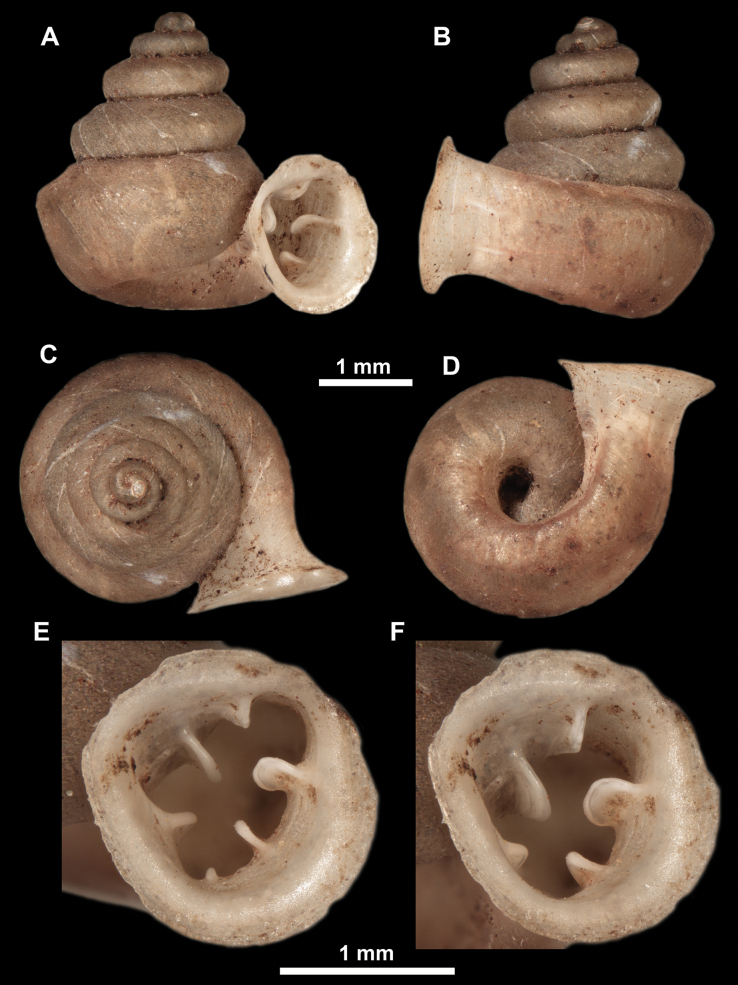
*Hypselostomakhmerianum* from Sa Kaeo Province, Thailand (coll. HA) **A–D** shell **E, F** enlarged apertural views.

Figs 186G , 202 , 223

Gyliotrachela khmeriana Sutcharit & Panha in Sutcharit et al. 2023: 1295–1299, fig. 4.

##### Type material examined.

**Cambodia** • holotype; CUMZ 14206.

##### Additional material examined.

**Thailand** • 15 shells (4 adult, 1 of them broken); Sa Kaeo Province, Khlong Hat district, Tham Phet Pho Thong; 13°24.946'N, 102°19.691'E; 03 June 2023; A. Hunyadi leg.; coll. HA.

##### Type locality.

“The limestone hills at Phnom Sampeov Mountain, Banan District, Battambang Province, Cambodia (13.026000°N, 103.101011°E)”.

##### Differential diagnosis.

This species is not similar to any hitherto described congener due to the combination of conical shell, absence of spiral striation, all whorls keeled, strong but not numerous apertural barriers, and separated angular and parietal lamellae. The shell shape somehow resembles *H.sculpturatum* but it differs from it by its adnate last whorl, separated angular and parietal lamellae and not spirally striated teleoconch. *Hypselostomacircumcarinatum* sp. nov. has its whorls keeled, a merged angulo-parietal lamella and spirally striated whorls. It is different from *H.chatnareeae* by the separated angular and parietal lamellae, weaker keel, and the absence of a deep groove above the keel.

##### Distribution.

This species is known from the type locality in Cambodia and is now reported for the first time in Thailand (Sa Kaeo province).

#### ﻿ Hypselostoma loei

Taxon classificationAnimaliaStylommatophoraHypselostomatidae

Panha & Prateespasen, 2005

75F14CD8-BADC-5C09-9EDD-1807B489DA0F

**Figure 203.**
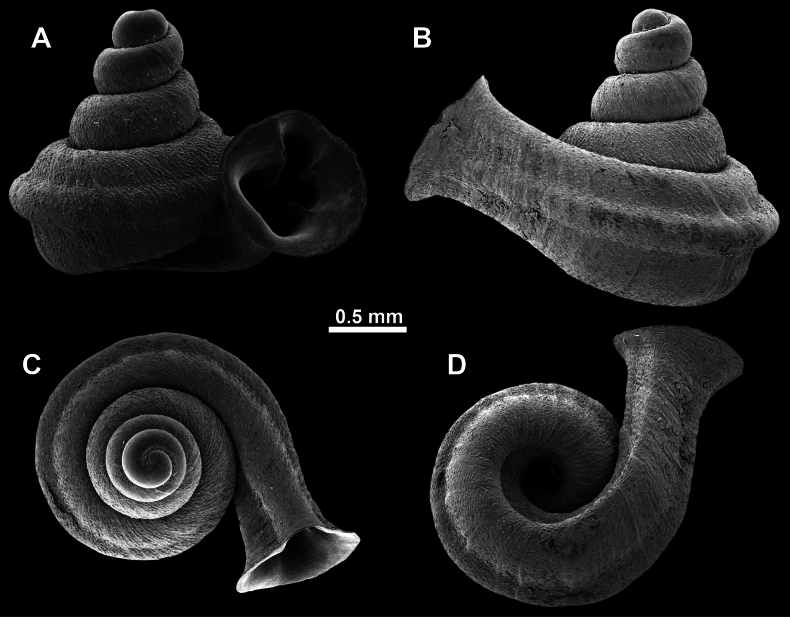
*Hypselostomaloei*, paratype (CUMZ ver. 025) **A–D** shell

Figs 186H , 203 , 223

Hypselostoma loei Panha & Prateespasen, 2005: 99, fig. 2.
Hypselostoma loei — Panha and Burch 2008: 94–95, fig. 81; Dumrongrojwattana and Wongkamhaeng 2024: 324, fig. 8.

##### Type material examined.

**Thailand** • 1 paratype; from the type locality; CUMZ ver. 025.

##### Additional material examined.

**Thailand-Central** • 3 shells; Phitsanulok Province, Noen Maprang district, Chomphu, Tham Phra Wang Daeng; 16°40.651'N, 100°41.287'E; 150 m a.s.l.; 02 Mar. 2023; A. Hunyadi & J.U. Otani leg.; coll. HA • **Thailand- Northeast** • 54 shells; Loei Province, Nong Hin district, 20.3 km southwest from the centre of Nong Hin towards Pha Wai, left side of the road no. 3029; 17°2.471'N, 101°43.655'E; 705 m a.s.l.; 28 Feb. 2023; A. Hunyadi & J.U. Otani leg.; coll. HA • 46 shells; Loei Province, Mueang Loei district, rock wall above Wat Tham Piya Thammarangsi; 17°27.896'N, 101°51.578'E; 27 Feb. 2023; A. Hunyadi & J.U. Otani leg.; coll. HA • 9 shells; Loei Province, 9.2 km NW Loei Buddhist temple, up and N of main cave entrance, among leaves; 17°35'N, 101°44'E; 28 Apr. 1988; K. Auffenberg leg.; locality code KA-0685, UF 345859 • 1 shell; Loei Province, 1 km E Ban Huai Muang; 17.474°N, 101.889°E; 375 m a.s.l.; 21. May 1987; F.G. Thompson leg.; locality code FGT-4274, UF 527260 • 11 shells; Nong Bua Lamphu Province, Na Klang district, Wat Phuttha Banphot - Tham Pha Choo; 17°18.931'N, 102°7.042'E; 27 Feb. 2023; A. Hunyadi & J.U. Otani leg.; coll. HA • 13 shells; Khon Kaen Province, Phu Pha Man district, rocks above Wat Phu Hua Chang; 16°39.170'N, 101°48.094'E; 345 m a.s.l.; 01 Mar. 2023; A. Hunyadi & J.U. Otani leg.; coll. HA • 10 shells; Khon Kaen Province, Chum Phae district, Na Nong Tum, Kled Kaew cave; 16°49.021'N, 101°57.382'E; 580 m a.s.l.; 01 Mar. 2023; A. Hunyadi & J.U. Otani leg.; coll. HA

##### Type locality.

“Limestone hills in Loei Province, 16°45'47"N. 101°59'8"E, 110 meters elevation…Thailand…”.

##### Differential diagnosis.

This species is similar to *H.hungerfordianum* but the latter has separate angular and parietal lamellae and more numerous barriers.

##### Distribution.

Known from Loei, Phitsanulok, Khon Kaen and Nong Bua Lamphu provinces in Thailand.

##### Remarks.

The striking similarity of this species with *H.hungerfordianum* suggests that *Gyliotrachela* is a junior synonym of *Hypselostoma*.

#### ﻿ Hypselostoma luctans

Taxon classificationAnimaliaStylommatophoraHypselostomatidae

(van Benthem Jutting, 1950) comb. nov.

63EBE0B3-3D4D-577B-A515-8FC59B135BDD

**Figure 204.**
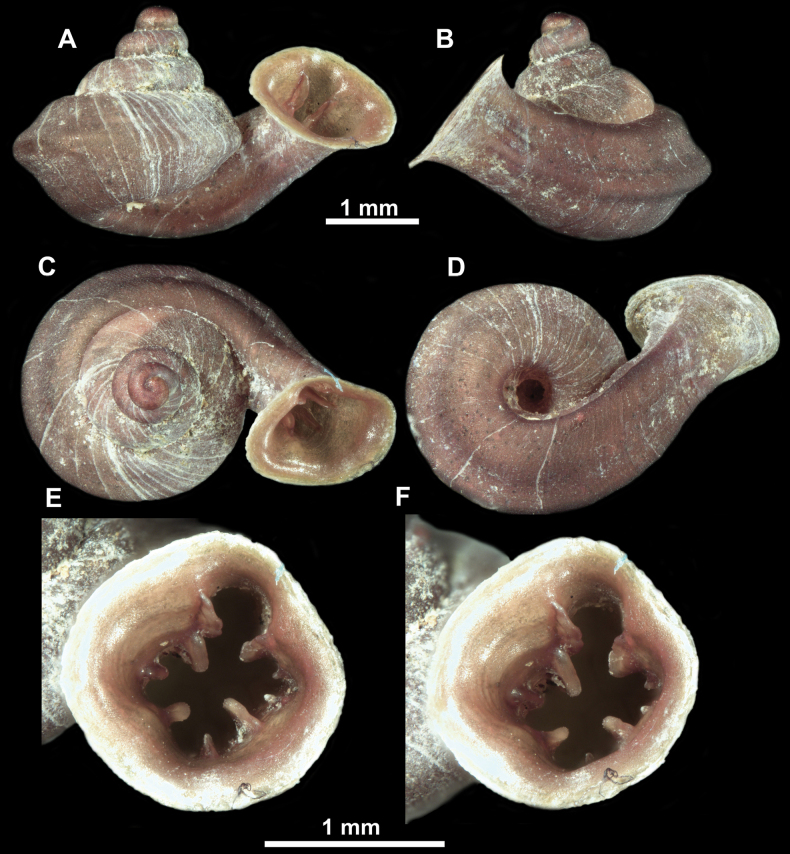
*Hypselostomaluctans*, paratype (RMNH.Moll.137158) **A–D** shell **E–G** enlarged apertural views.

**Figure 205.**
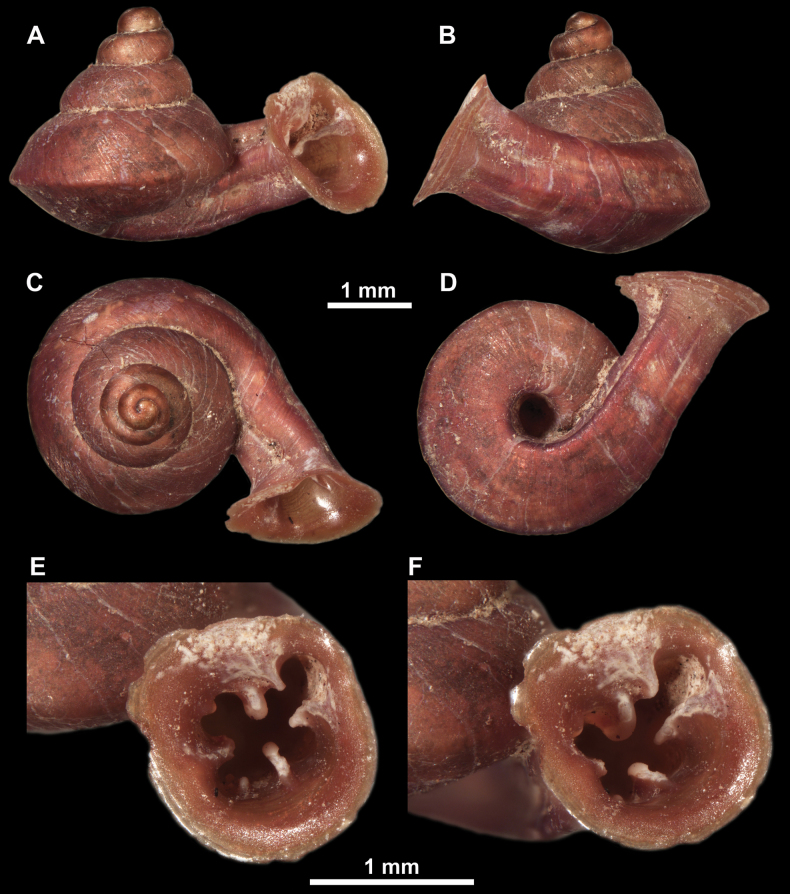
*Hypselostomaluctans* from Yala province, Thailand (coll. HA) **A–D** shell **E, F** enlarged apertural views.

Figs 186I , 204 , 205 , 223

Gyliotrachela luctans van Benthem Jutting, 1950: 33–34, fig. 19.
Gyliotrachela luctans — van Benthem Jutting 1950: 46; Zilch 1984: 166; Maassen 2001: 76; Foon et al. 2017: 79, fig. 30C.

##### Type material examined.

**Malaysia** • 23 paratypes; from the type locality; 1938; Raffles Museum Singapore ex. coll.; RMNH.Moll.137158.

##### Additional material examined.

**Thailand** • 7 shells; Yala Province, 9.5 km from the junction to Bannang Sata, 1700 m north from the road no 4077, left side of the side road; 6°19.803'N, 101°13.135'E; 120 m a.s.l.; 22 Feb. 2023; A. Hunyadi leg.; coll. HA.

##### Type locality.

“Gunong Pondok, Padang Rengas, Perak”, Malaysia.

##### Differential diagnosis.

This species is similar to *H.bubalus* sp. nov. with which it is also geographically approximate. It differs from it by a clearly narrower umbilicus. *Hypselostomabubalus* sp. nov. is also spirally striated on some isolated parts of the shell (above or below the keel), but *H.luctans* is not. See also under *H.piconis*.

##### Distribution.

This species is known from Peninsular Malaysia and Yala Province in Thailand.

##### Remarks.

There can be two interpalatal plicae, and in some cases, none are visible. Umbilicus can sometimes be slightly wider, with a stronger groove inside (than the figured specimen).

#### ﻿ Hypselostoma ophis

Taxon classificationAnimaliaStylommatophoraHypselostomatidae

Gojšina, Hunyadi & Páll-Gergely sp. nov.

94691362-CCF7-5175-A3DC-2CE31A574849

https://zoobank.org/6C352298-6F8E-4977-9BC1-1307D993A47D

**Figure 206.**
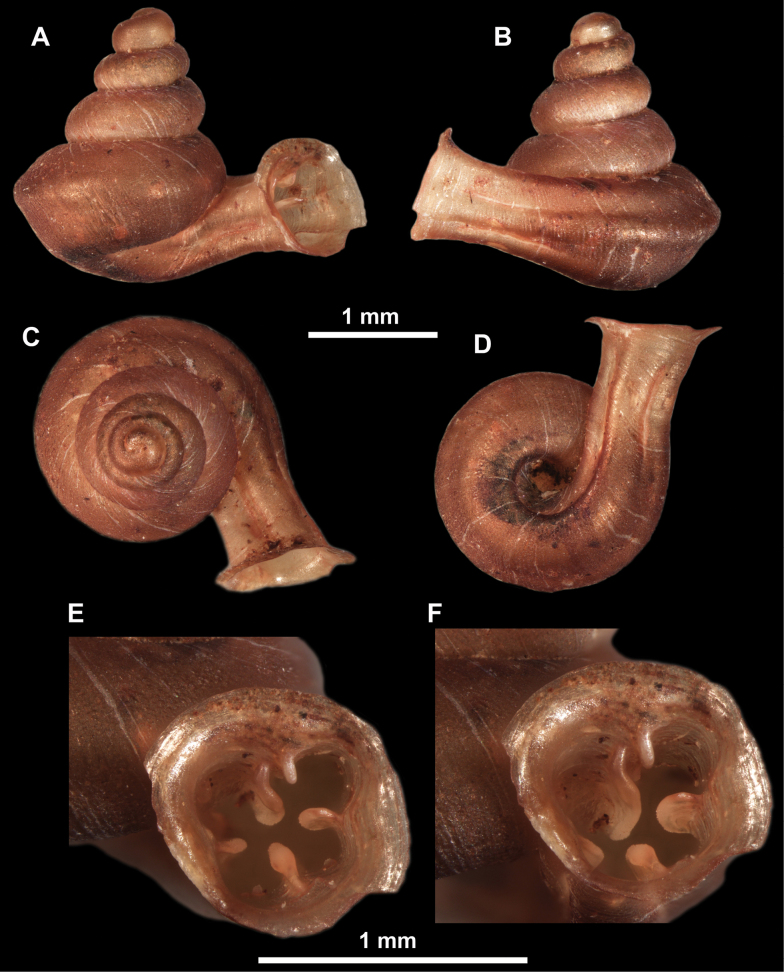
*Hypselostomaophis* Gojšina, Hunyadi & Páll-Gergely, sp. nov., holotype (CUMZ 14467) **A–D** shell **E, F** enlarged apertural views.

**Figure 207.**
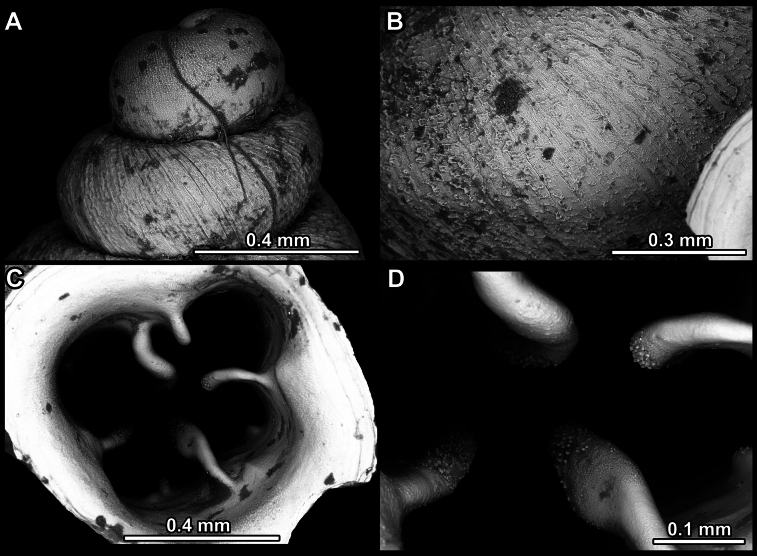
SEM imaging of *Hypselostomaophis* Gojšina, Hunyadi & Páll-Gergely, sp. nov., holotype (CUMZ 14467) **A** protoconch surface **B** teleoconch surface **C, D** enlarged apertural views.

Figs 186J , 206 , 207 , 223

##### Type material.

***Holotype*. Thailand** • 1 shell (SH: 2.1 mm; SW: 2.6 mm); Phattalung Province, Phattalung, Khao Ok Thalu, rock wall; 07°37.506'N, 100°05.330'E; 19 Feb. 2015; A. Hunyadi leg.; CUMZ 14467. ***Paratypes*. Thailand** • 5 shells (3 whole and 2 last whorls with retained apertures); same data as for holotype; coll. HA.

##### Additional material examined.

**Thailand** • 1 shell (fragment, not paratype); same data as for holotype; coll. HA.

##### Type locality.

Thailand, Phattalung Province, Phattalung, Khao Ok Thalu, rock wall; 07°37.506'N, 100°05.330'E.

##### Diagnosis.

A *Hypselostoma* species with sandpaper-like shell surface and a bluntly keeled last whorl which is detached from the penultimate and slightly ascending upwards. Five main barriers in the aperture (parietal, angular, upper palatal, lower palatal and columellar). Parietal lamella very long and wavy. Umbilicus moderately wide.

##### Description.

Shell shape concave-conical (due to the strongly enlarged last whorl), consisting of 4.5–5 convex whorls separated by a deep suture. Colouration dark brown, weakly glossy and opaque. Protoconch finely pitted, showing very weak spiralling pattern, consisting of ~ 1.25–1.5 whorls, coloured the same as the teleoconch. Teleoconch very finely granulated (sandpaper-like) with additional radial growth lines. Also, some irregularly spaced radial white streaks are occasionally visible. Last whorl bluntly keeled, moderately detached from the penultimate and slightly ascending upwards (~ 20 ° compared to the shell axis). In lateral view, two strong grooves are visible near the aperture, one above and one below the central keel. Peristome expanded and not reflected, slightly lighter than the rest of the shell. Aperture equipped with five strong and thick barriers (parietal, angular, upper palatal, lower palatal, and columellar) and several smaller ones. Parietal lamella very strong, wavy, and very long. Angular lamella short and sometimes separated in inner and outer part. Inner part is weaker. Both palatal plicae very strong, nearly as strong as the parietal lamella or even stronger but clearly shorter. In front of the upper palatal plica, a small swelling is present which is probably homologous with the palatal tubercle present in other genera (e.g., *Bensonella*, see Páll-Gergely and White 2023) Columellar lamella nearly horizontal, stronger only than the angular among the main barriers. Additionally, smaller barriers are present between the main ones. There is usually one in the interpalatal region, one basal and one between the columellar and parietal. None of these barriers reach the peristome. Surface of all barriers is very finely spiniferous medially and very finely granulated laterally. Sinulus rounded and visibly separated from the rest of the aperture. Umbilicus open and moderately wide, measuring 1/3 of the shell width and showing the penultimate whorl. A strong groove running from the peristome towards the umbilicus is visible. This groove is getting lost near the penultimate whorl.

##### Differential diagnosis.

See under *H.venustum*.

##### Measurements

**(in mm, *n* = 4).**SH = 1.76–2.1; SW1 = 2.38–2.6; SW2 = 1.48–1.54; AH = 0.88–0.95; AW = 0.85–0.89.

##### Etymology.

The specific epithet is derived from the Greek work *ophis* (snake) which is in special reference to the curved and long parietal lamella (which is also one of the longest in all hitherto described *Hypselostoma* species). To be used as a noun in apposition.

##### Distribution.

This species is known only from the type locality.

#### ﻿ Hypselostoma piconis

Taxon classificationAnimaliaStylommatophoraHypselostomatidae

van Benthem Jutting, 1949

6BC13778-D39F-5922-9375-461E690CAAB2

**Figure 208.**
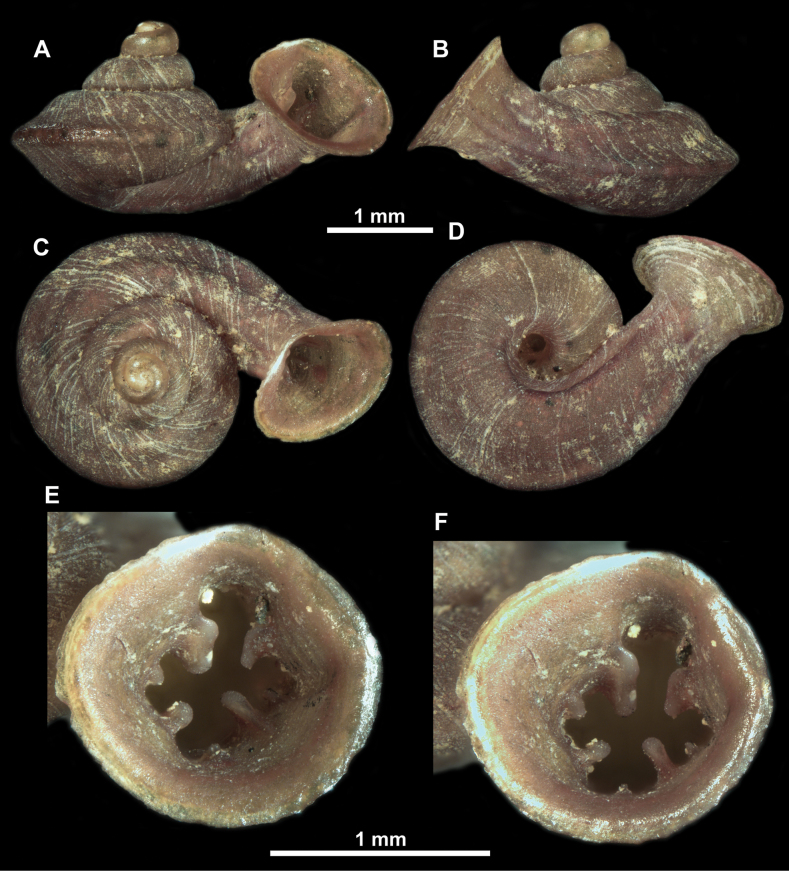
*Hypselostomapiconis*, paratype (RMNH.Moll.140161) **A–D** shell **E, F** enlarged apertural views.

Figs 186K , 208 , 223

Hypselostoma piconis van Benthem Jutting, 1949b: 59, pl. 2., figs a–d.
Hypselostoma piconis — van Benthem Jutting 1950: 24, 42, fig. 13; Maassen 2001: 77.

##### Type material examined.

**Malaysia** • 1 paratype; from the type locality; Raffles Museum, Singapore ex. coll.; RMNH.Moll.140161.

##### Additional material examined.

**Malaysia** • 9 shells; Perak, 8–10 km northeast from Sungai Siput, east from Batu Lima, cave temple; 04°51.985'N, 101°07.333'E; 70 m a.s.l.; 07 Jan. 2013; A. Hunyadi leg.; coll. HA.

##### Type locality.

“Sungei Siput, Perak”, Malaysia.

##### Differential diagnosis.

*Hypselostomapiconis* is strikingly similar to *H.luctans* from which it can be separated by the appearance of the apertural barriers, most importantly the concrescent angular and parietal lamellae, which are separate in *H.luctans*.

##### Distribution.

This species is known only from the type locality and its surroundings.

##### Remarks.

The fact that the major difference between *H.piconis* and *H.luctans* is the appearance of the barriers on the parietal side further supports the indistinctness of *Gyliotrachela* and its synonymy with *Hypselostoma*.

#### ﻿ Hypselostoma sichang

Taxon classificationAnimaliaStylommatophoraHypselostomatidae

(Panha & J. B. Burch, 2002) comb. nov.

95DC87B3-CABE-5743-B611-F5C3C4BD9EF8

**Figure 209.**
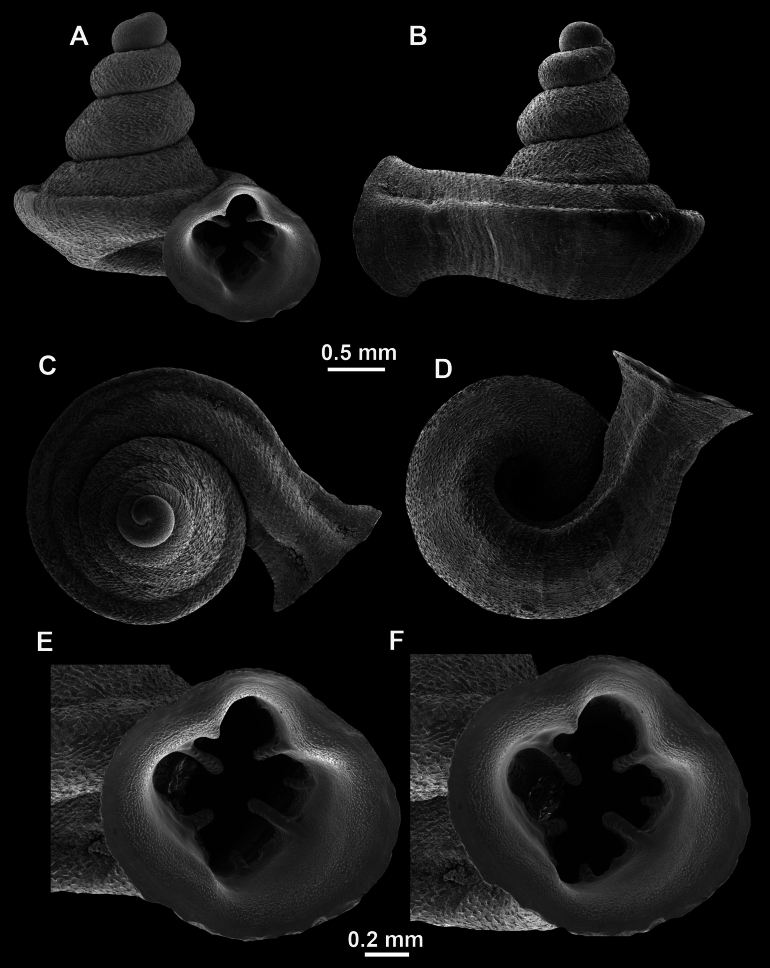
*Hypselostomasichang*, holotype (CUMZ 44001) **A–D** shell **E, F** enlarged apertural views.

**Figure 210.**
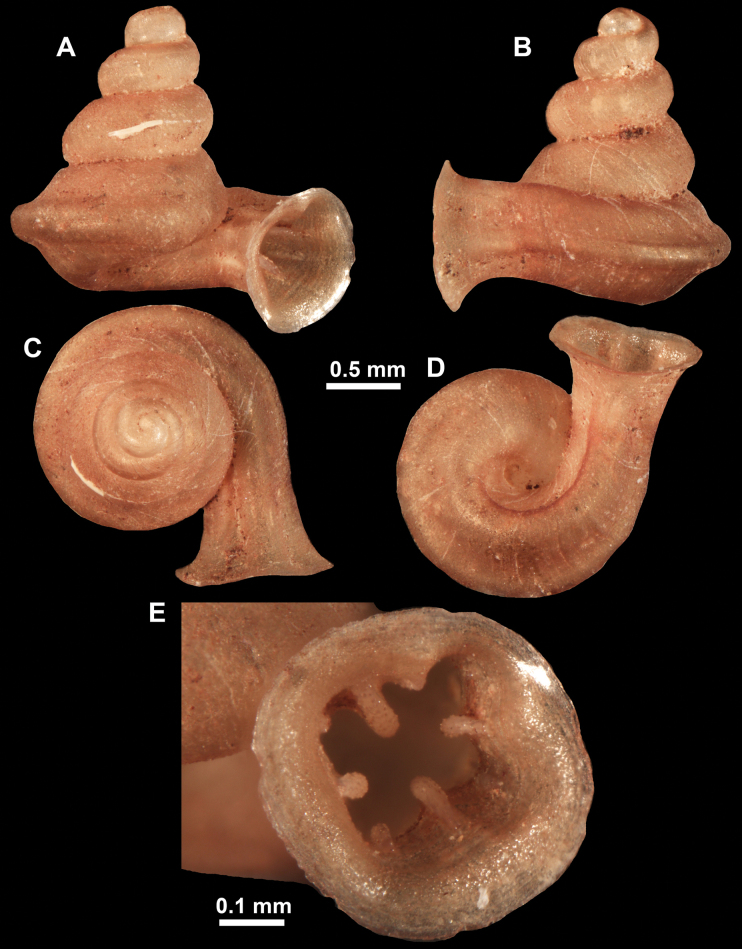
*Hypselostomasichang* from Sa Kaeo province (coll. HA) **A–D** shell **E** enlarged apertural view.

Figs 186L , 209 , 210 , 223

Anauchen sichang Panha & Burch, 2002e: 21, fig. 2.
Gyliotrachela sichang — Panha and Burch 2008: 80, fig. 69; Dumrongrojwattana and Wongkamhaeng 2024: 324, fig. 8.

##### Type material examined.

**Thailand** • holotype; 2001; S. Panha leg.; CUMZ 44001 • 2 paratypes; from the type locality; 2001; S. Panha leg.; SMF 331463.

##### Additional material examined.

**Thailand** • 10 shells; Sa Kaeo Province, Wang Sombun district, vicinity of Tham Khoa Phlapphueng Thong; 13°26.873'N, 102°13.071'E; 06 Mar. 2023; A. Hunyadi leg.; coll. HA.

##### Type locality.

“Sichang island, Si Racha, Chon Buri Province, 13°20′05″N, 100°55′24″E, 70 meters elevation” (Thailand).

##### Differential diagnosis.

This species is similar to *H.hungerfordianum* from which it can be separated by the much stronger and lower peripheral keel, very rough, sandpaper-like shell surface sculpture as well as the narrower umbilicus. See also under *H.vesovici* sp. nov.

##### Distribution.

This species is, apart from the type locality, known from one more sampling site in Sa Kaeo Province.

#### ﻿ Hypselostoma srakeoense

Taxon classificationAnimaliaStylommatophoraHypselostomatidae

(Panha & J. B. Burch, 2004)

A887FB65-FABB-5BFD-ACD4-C14C7083EBAF

**Figure 211.**
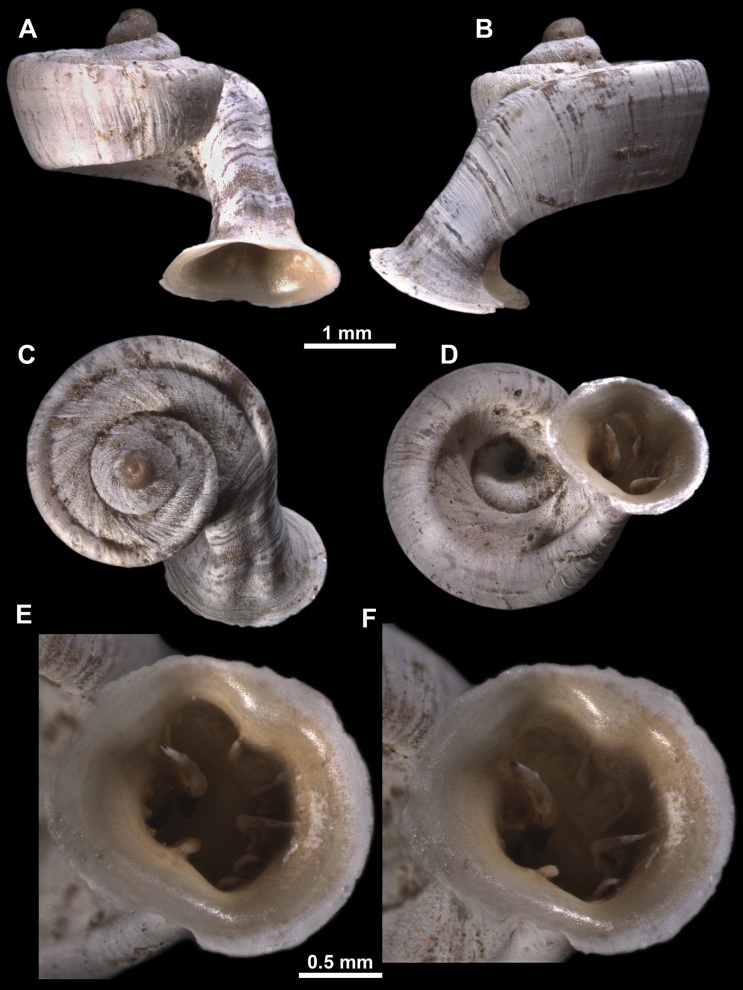
*Hypselostomasrakeoense*, paratype (SMF 331473). **A–D** shell **E, F** enlarged apertural views.

Figs 186M , 211 , 223

Anauchen srakeoensis Panha & Burch in Panha et al. 2004: 62–63, fig. 4.
Anauchen srakeoensis — Panha and Burch 2008: 52–53, fig. 48; Dumrongrojwattana and Wongkamhaeng 2024: 323, fig. 7.
Hypselostoma srakeoensis — Sutcharit et al. 2023: 1291–1293, fig. 2.

##### Type material examined.

**Thailand** • 1 paratype; from the type locality; S. Panha leg.; SMF 331473.

##### Additional material examined.

**Thailand** • 1 shell; Srakeo Province, Khaochakan District, Wat Khao Chakan; 22 June 2014; P. Dumrongrojwattana leg.; ex. coll. Maassen; coll. PGB.

##### Type locality.

“Plubpluengtong limestone hills, Srakeo Province, 13°48'02"N, 102°12'49"E, 110 meters elevation” (Thailand).

##### Differential diagnosis.

This species is superficially similar to several congeners with strongly enlarged last whorl which is also sharply descending. The two most similar species are *H.torticollis* and *H.fungus* sp. nov. However, *H.torticollis* has separated lamellae on the parietal side and it is also less depressed with clearly narrower umbilicus. *Hypselostomafungus* sp. nov. has more rounded last whorl, wider umbilicus, more apertural barriers and is spirally striated. See also under *H.pendulum*.

##### Distribution.

This species is known from four sampling sites, two in Sa Kaeo province (Thailand) and two in Banteay Meanchey province (Cambodia) (Sutcharit et al. 2023).

#### ﻿ Hypselostoma surakiti

Taxon classificationAnimaliaStylommatophoraHypselostomatidae

(Panha & J. B. Burch, 2003)

C1EECA34-E5E1-5C94-A4A2-8728002CE654

**Figure 212.**
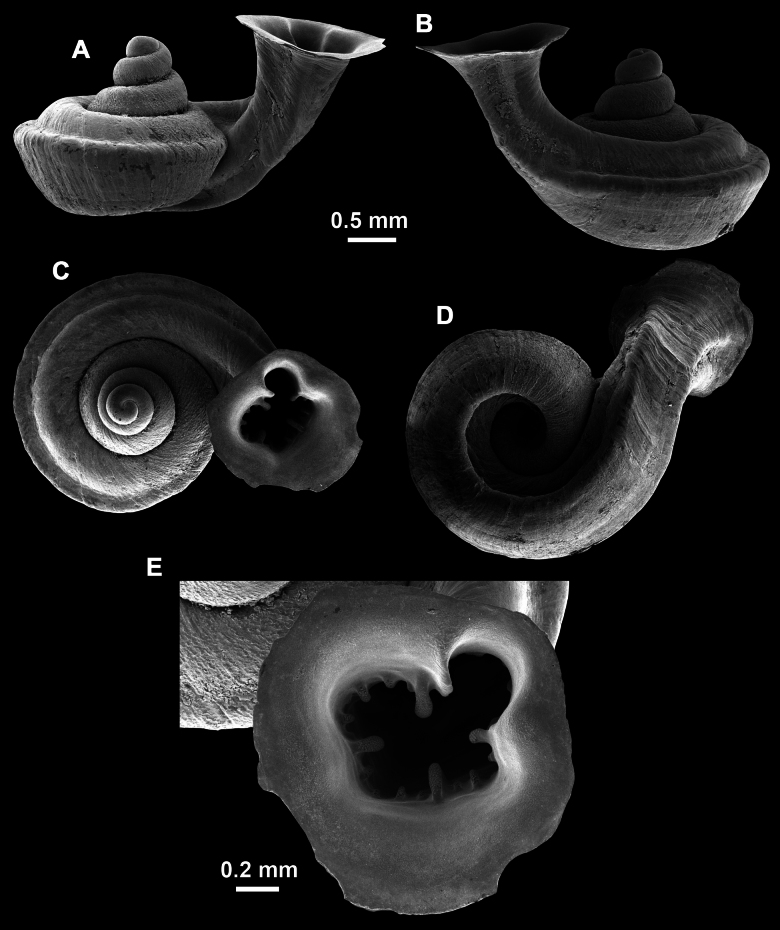
*Hypselostomasurakiti*, paratype (CUMZ ver. 078) **A–D** shell **E** enlarged apertural view.

Figs 186N , 212 , 223

Gyliotrachela surakiti Panha & Burch in Burch et al. 2003: 162–169, figs 12, 13.
Gyliotrachela surakiti — Tongkerd et al. 2004: 143, fig. 2; Panha and Burch 2008: 82–84, fig. 72; Dumrongrojwattana and Wongkamhaeng 2024: 324, fig. 8.

##### Type material examined.

**Thailand** • 2 paratypes; from the type locality; SMF 331450 • 1 paratype; from the type locality; CUMZ ver078.

##### Type locality.

“South of Puttabanpot Temple, Pajoh Village, Nawang District, Nongbualumpoo Province, 17°19'1"N, 102°7'3"E, 320 meters elevation, Thailand”.

##### Differential diagnosis.

This species is by the level of its last whorl ascension similar to *H.kohrin* and *H.diarmaidi*. It can be separated from *H.kohrin* and *H.diarmaidi* by the absence of typical spiral striation on the shell which is present in the latter two.

##### Distribution.

This species is known only from the type locality.

##### Remarks.

In *H.surakiti*, we could observe some irregular and weak spiral striae on the last whorl although they are not in form of typical raised spiral striae.

#### ﻿ Hypselostoma transitans

Taxon classificationAnimaliaStylommatophoraHypselostomatidae

Möllendorff, 1894

D376B983-3A16-5298-A454-CD46DC6DD0E3

**Figure 213.**
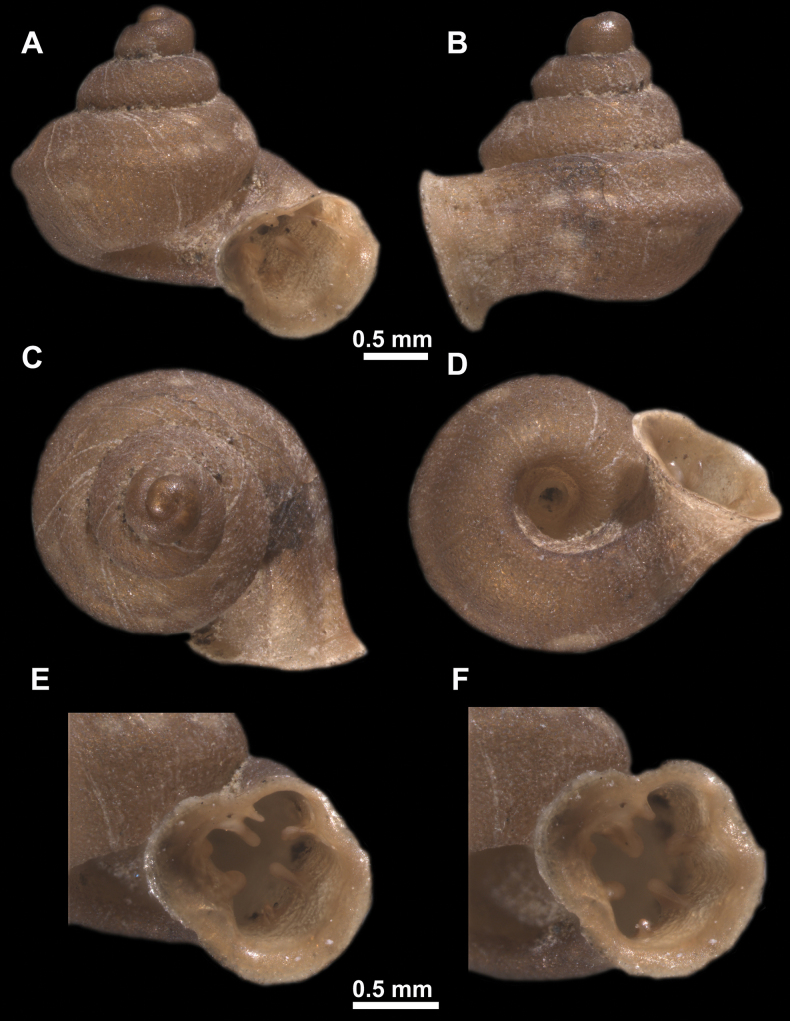
*Hypselostomatransitans*, lectotype (SMF 4590) **A–D** shell **E, F** enlarged apertural views.

**Figure 214.**
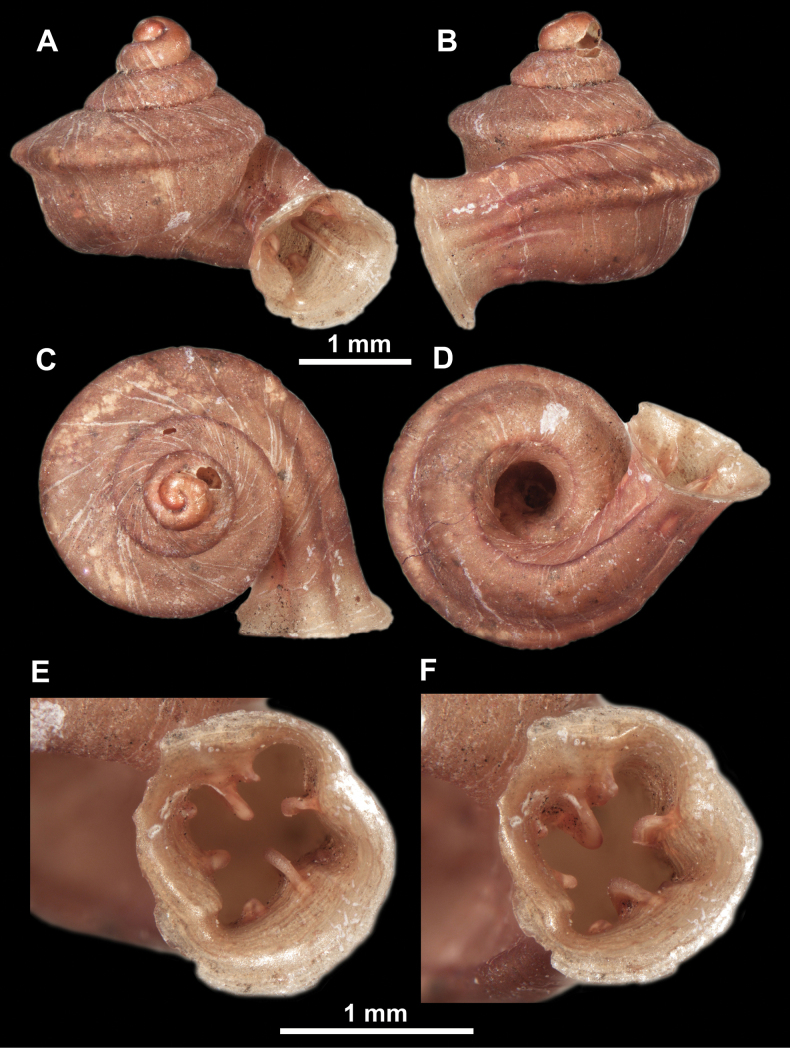
*Hypselostomatransitans*, specimen from Tham Khao Phlu (coll. HA) **A–D** shell **E, F** enlarged apertural views.

Figs 186O , 213 , 214 , 223

Hypselostoma transitans Möllendorff, 1894: 16, figs 12, 13.
Hypselostoma translucidum [sic] — Fischer and Dautzenberg 1904: 408.
Gyliauchen transitans — Pilsbry 1917: 214–215, pl. 36, figs 5–8.
Gliotrachela transitans [sic] — Laidlaw 1933: 214.
Gyliotrachela transitans transitans — van Benthem Jutting 1950: 47; van Benthem Jutting 1950: 28; Thompson and Upatham 1997: 233.
Gyliotrachela transitans — Zilch 1984: 166; Hemmen and Hemmen 2001: 41; Panha and Burch 2008: 85, fig. 74; Dumrongrojwattana and Wongkamhaeng 2024: 324, fig. 8.
Hypselostoma transitans — Fischer and Dautzenberg 1904: 408.

##### Type material examined.

**Thailand** • 1 lectotype; SMF 4589 • 8 paralectotypes; from the type locality; SMF 4590.

##### Additional material examined.

**Thailand** • 2 shells; Chumphon Province, 2.5 km northeast from Pathio, Tham Khao Phlu; 10°43.851'N, 99°19.242'E; 23 Feb. 2015; A. Hunyadi leg.; coll. HA • 22 fragments and 1 complete shell; Suratthani Province, Angthong islands, Wia Talap island, steep path to Buaboke cave, at the base of limestone rocks; 8°7.603'N, 98°55.466'E; 50 m a.s.l.; Sep. 2007; A. Reischütz leg.; coll. REI.

##### Type locality.

“Samui islands, Gulf of Siam”, Thailand.

##### Differential diagnosis.

*Hypselostomatorticollis* is similar due to the down-turning aperture and same arrangement of the apertural barriers but this species has a much wider umbilicus, more enlarged last whorl and is spirally striated. *Hypselostomafungus* sp. nov. is spirally striated, has merged angular and parietal lamellae, more down-turning last whorl and a much wider umbilicus. See also under *H.venustum*.

##### Distribution.

This species in known from Chumphon and Suratthani provinces in Thailand.

##### Remarks.

In some paralectotypes, a lamella between the columellar and parietal was positioned much deeper in the aperture. We have examined a distinct form of this species from Tham Khao Phlu (Chumphon province). This form had a wider umbilicus and a sharper keel as well as more enlarged last whorl in comparison to previous ones. However, we decided not to treat it as distinct species due to the fact that we have also found a transitional form between the typical *H.transitans* (close to its type locality) and the one from Tham Khao Phlu.

#### ﻿ Hypselostoma venustum

Taxon classificationAnimaliaStylommatophoraHypselostomatidae

(van Benthem Jutting, 1950)

A02A9D5E-1C08-555B-A9BE-CCC1A11E5401

**Figure 215.**
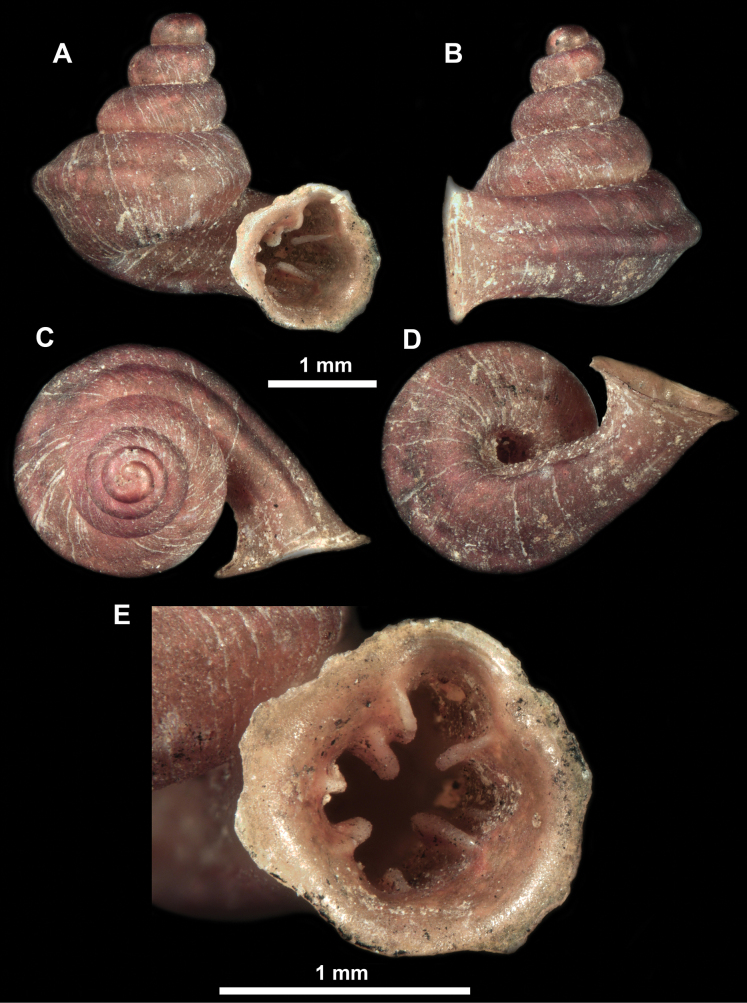
*Hypselostomavenustum*, holotype (RMNH.Moll.137140) **A–D** shell **E, F** enlarged apertural views.

**Figure 216.**
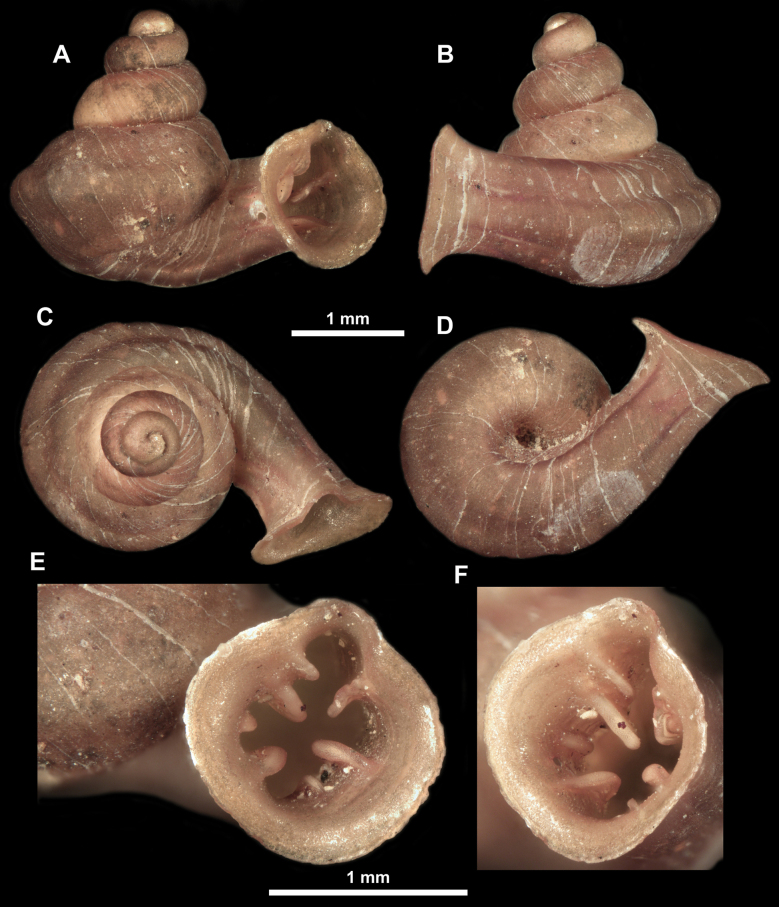
*Hypselostomavenustum*, paratype of *G.transitanshelioscopia* (RMNH.Moll.137144) **A–D** shell **E, F** enlarged apertural views.

Figs 186P , 215 , 216 , 223

Gyliotrachela transitans venusta van Benthem Jutting, 1950: 28–29, 47, fig. 16.
Gyliotrachela transitans venusta — Maassen, 2001: 76.
Gyliotrachela transitans helioscopia van Benthem Jutting, 1950: 29–31, fig. 17. syn. nov.
Gyliotrachela transitans heliscopia [sic] — Maassen, 2001: 76.

##### Type material examined.

**Malaysia** • holotype of *G.transitansvenusta*; Raffles Museum, Singapore ex. coll.; RMNH.Moll.137140 • 8 paratypes of *G.transitanshelioscopia*; from the type locality; Raffles Museum, Singapore ex. coll.; 1947; RMNH.Moll.137144.

##### Additional material examined.

**Malaysia** • 3 shells; Pahang, 20 km southeast from Jerantut, Gua Kota Gelanggi, under Gua Balai; 03°54.000'N, 102°28.412'E; 115 m a.s.l.; 21 Jan. 2013; A. Hunyadi leg.; coll. HA.

##### Type localities.

“Gunong Pondok, Padang Rengas, Perak”, Malaysia (*H.venustum*); “Kota Tongkat, Pahang”, Malaysia (*G.transitanshelioscopia*).

##### Differential diagnosis.

This species is similar to *H.ophis* sp. nov., from which it can be separated by narrower umbilicus and shorter and wavy parietal lamella. *Hypselostomatransitans* has more rough shell sculpture as well as the descending last whorl and a wider umbilicus. See also under *H.vesovici* sp. nov.

##### Distribution.

This species is known only from three localities in Peninsular Malaysia (Gunong Pondok, Kota Tongkat and Gua Kota Gelanggi).

##### Remarks.

Van Benthem Jutting (1950) recognised two additional subspecies of *G.transitans*, namely *G.transitanshelioscopia* and *G.transitansvenusta*. However, these two taxa should not be considered as subspecies since they differ from the nominotypical subspecies in several very important characters: last whorl is in nominotypical subspecies strongly descending near the aperture while in *G.transitansvenusta* it is slightly ascending; whorls are more depressed in nominotypical subspecies; shell surface is also different, being more sandpaper-like in nominotypical subspecies and almost smooth to very finely sculptured in other two subspecies. Finally, umbilicus is wider in *G.transitans*. Because of all aforementioned clear differences, we conclude that *H.venusta* is a good species separated from *H.transitans*. *Gyliotrachelatransitanshelioscopia* is its junior synonym due to the virtually identical shell morphology. Van Benthem Jutting (1950) noted that in *G.transitansvenusta*, the shell is less elevated, peripheral keel sharper and longitudinal grooves deeper than in *G.transitanshelioscopia*. These differences are only minor and fall within the intraspecific variability, thus there are no clear characters which allow the separation of these two taxa.

#### ﻿ Hypselostoma vesovici

Taxon classificationAnimaliaStylommatophoraHypselostomatidae

Gojšina & Páll-Gergely sp. nov.

3E3B47F9-AE5F-59EA-86FC-7143B4614F37

https://zoobank.org/0E185F99-7FEB-4A8C-94C9-F2B387F07854

**Figure 217.**
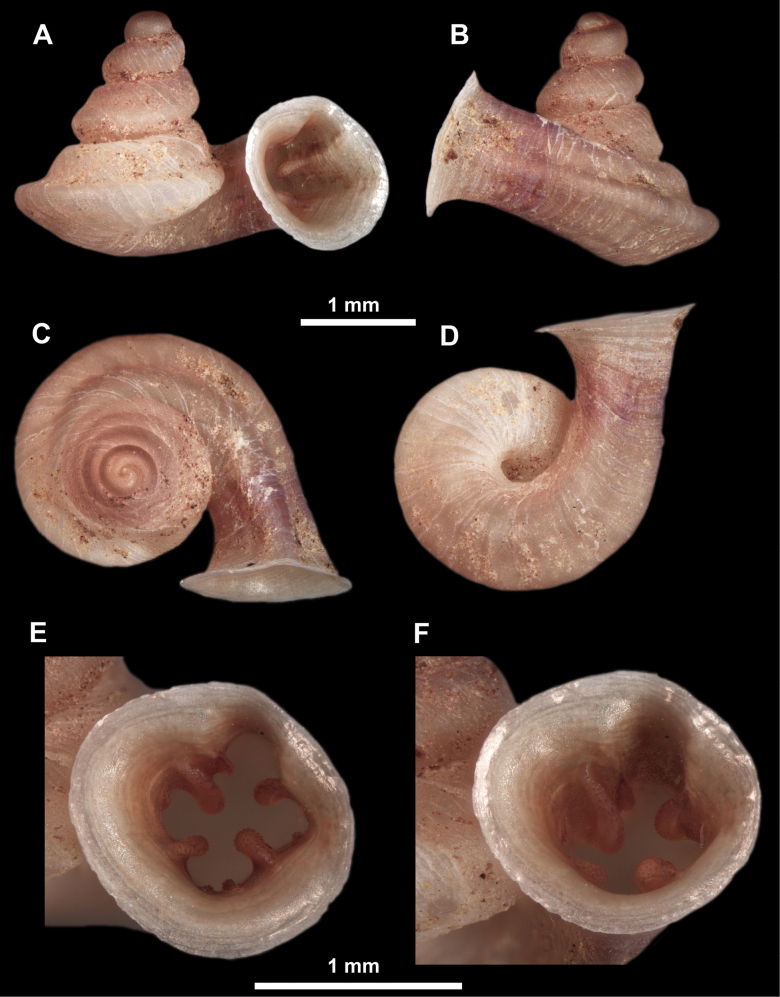
*Hypselostomavesovici* Gojšina & Páll-Gergely, sp. nov., holotype (CUMZ 14463) **A–D** shell **E, F** enlarged apertural views.

**Figure 218.**
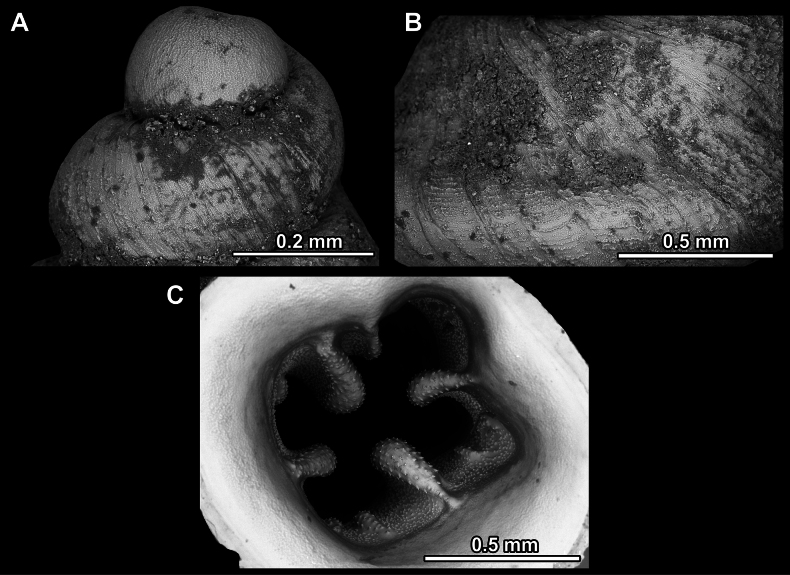
SEM imaging of *Hypselostomavesovici* Gojšina & Páll-Gergely, sp. nov., holotype (CUMZ 14463) **A** protoconch surface **B** teleoconch surface **C** enlarged apertural view.

Figs 186Q , 217 , 218 , 223

##### Type material.

***Holotype*. Malaysia** • 1 shell (SH: 2.18 mm; SW1: 3.12 mm); Gua Telinga, Kuala Tahan, Taman Negara, Jerantut, Pahang; 4°22.326'N, 102°23.747'E; 69 m a.s.l.; T. Ishibe, K. Ohara, K. Okubo & J.U. Otani leg.; CUMZ 14463. ***Paratypes*. Malaysia** • 10 shells; same data as for holotype; coll. PGB • 1 shell; same data as for holotype; coll. VG • 1 shell; same data as for holotype; coll. HA.

##### Type locality.

Malaysia, Gua Telinga, Kuala Tahan, Taman Negara, Jerantut, Pahang; 4°22.326'N, 102°23.747'E; 69 m a.s.l.

##### Diagnosis.

Shell concave-conical, with roughly granulated (sandpaper-like) surface sculpture. Last two whorls keeled below the periphery, keel especially strong on the last whorl. Last whorl detached from the penultimate, directed upwards. Aperture equipped with five main barriers and usually five smaller ones. All barriers are very broad and thick, very strongly spiniferous. Umbilicus very narrow, dot-like.

##### Description.

Shell concave-conical (due to the strongly enlarged last whorl), pinkish-brown in colour, corneous. It is consisting of 4.5–5 regularly increasing whorls separated by a deeply impressed suture. Protoconch finely pitted, not showing spiralling pattern, consisting of 1.25–1.5 whorls and slightly darker than the rest of the shell. Protoconch- teleoconch boundary clearly visible due to the change in shell surface sculpture. Teleoconch roughly granulated, sandpaper-like. On the last whorl near the keel, granules can in some specimens be arranged in a way to provoke a spiralling pattern although no regular spiral striae are developed. Radial lines are strong, widely and irregularly spaced, ~ 17 radial lines on the last whorl in standard view. They are more densely arranged on the initial teleoconch whorls. Protoconch and initial teleoconch whorls rounded and convex, last two whorls are keeled below the centre of the periphery. Last whorl especially strongly keeled, with a deep groove above the keel. It is also moderately detached from the penultimate near the aperture, and slightly ascending (~ 15–25 ° compared to the shell axis). Peristome whitish, strongly expanded but not reflected. Aperture large, equipped with five main barriers (parietal, angular, upper palatal, lower palatal and columellar). Angular lamella is the weakest and the shortest among the main five. All other main barriers are relatively thick and broad, almost equally developed. Parietal lamella is the longest in the aperture and surpassing the profile of the angular. Between the main barriers, several smaller ones could be observed. There are usually two interpalatal plicae, two between the lower palatal and the columellar and one knob-like infraparietal lamella. All barriers, as well as the aperture surface, are very strongly spiniferous. These spines are widely spaced (especially on the barriers) and much stronger than in other congeners. Sinulus not very distinctly separated from the rest of the aperture. Umbilicus is very narrow, dot-like, not showing previous whorls and measuring slightly > 1/10 of the shell width. A very shallow groove is visible running alongside the umbilicus.

##### Differential diagnosis.

This species is by the general shell shape similar to *H.venustum* from which it clearly differs by a sharply keeled last whorl below the periphery (provoking a discoid basis of the shell and a concave surface above the keel), thicker apertural barriers with very strongly spiniferous surfaces and a slightly narrower umbilicus with a much shallower groove inside it. Furthermore, the penultimate whorl in *H.venustum* is rounded, not keeled as in *H.vesovici* sp. nov. and the aperture in *H.vesovici* sp. nov. is much larger when compared to the rest of the shell than in *H.venustum*. However, the species which has the same appearance of the last whorl, and thus most similar are *H.sichang* and *H.discobasis*. In contrast to the new species, these two representatives share the different shell surface (very roughly sandpaper-like in *H.sichang* and coarsely spirally striated in *H.discobasis*) and different arrangement of the apertural barriers (less strong and spiniferous, angular and parietal lamellae merged in *H.discobasis*), as well as much wider umbilicus especially in *H.discobasis*. *Hypselostomavesovici* sp. nov. also has clearly much larger aperture when compared to the rest of the shell than in *H.sichang*.

##### Measurements

**(in mm, *n* = 5).**SH = 2.10–2.35; SW1 = 2.53–3.12; SW2 = 1.49–1.77; AH = 1.10–1.23; AW = 1.05–1.25.

##### Etymology.

Named after Dr. Nikola Vesović, a prominent Serbian entomologist and a friend of the first author.

##### Distribution.

Known only from the type locality.

#### ﻿ Hypselostoma vicinum

Taxon classificationAnimaliaStylommatophoraHypselostomatidae

Gojšina, Auffenberg & Páll-Gergely sp. nov.

80BF9D4E-EC8F-5050-810C-AE16FBD94350

https://zoobank.org/9236E2A9-034C-48AB-975A-15C36885984E

**Figure 219.**
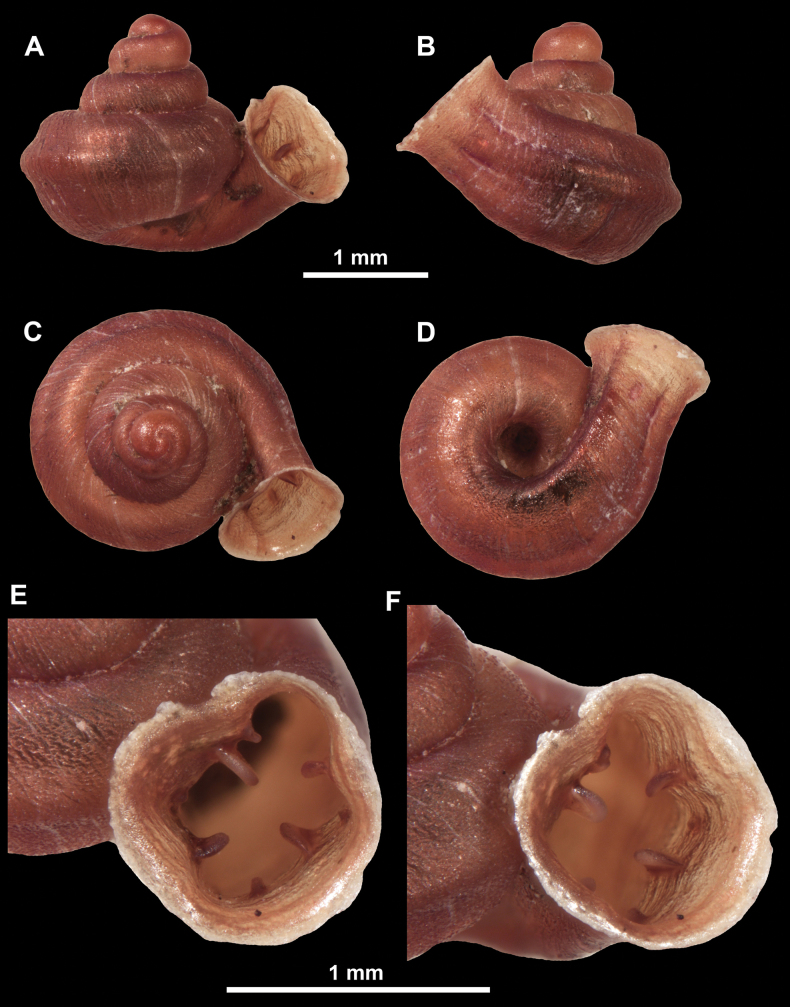
*Hypselostomavicinum* Gojšina, Auffenberg & Páll-Gergely, sp. nov., holotype (UF 345327) **A–D** shell **E, F** enlarged apertural views.

**Figure 220.**
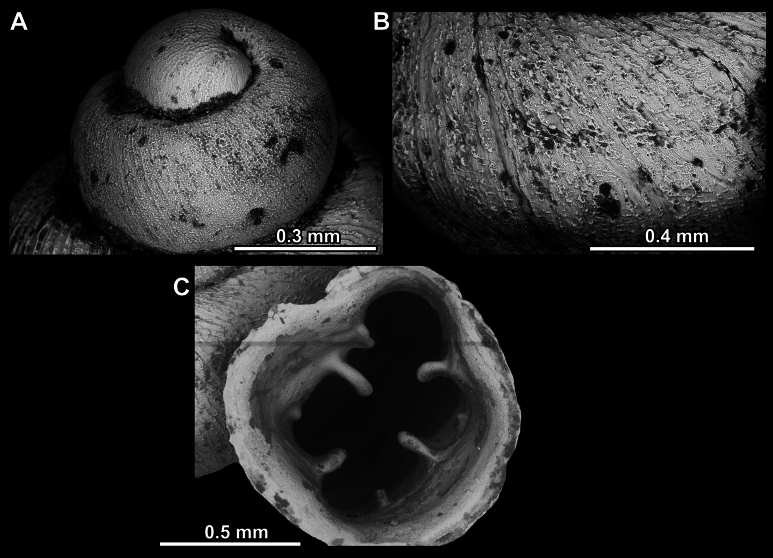
SEM imaging of *Hypselostomavicinum* Gojšina, Auffenberg & Páll-Gergely, sp. nov., holotype (CUMZ 345327) **A** protoconch surface **B** teleoconch surface **C** enlarged apertural view.

Figs 186R , 219 , 220 , 223

##### Type material.

***Holotype*. Thailand** • 1 shell (SH: 1.9 mm, 2.6 mm); Suratthani Province, limestone outcrop along Hwy. 401, 2.7 km W junc Hwys. 4142 & 401, evergreen forest on rocky hillside, below cliff, base of cliff; 9°10'N, 99°40'E; 90 m a.s.l.; 18 Apr. 1988; K. Auffenberg leg.; UF 345327.

***Paratypes*. Thailand** • 1 shell; same data as for holotype; CUMZ 14468 • 51 shells; same data as for holotype; UF 591363 • 45 shells; same data as for holotype; locality code KA-0674, UF 345336 • 16 ethanol-preserved specimens; same data as for holotype; locality code KA-0673, UF 345328.

##### Additional material examined.

**Thailand** • 12 shells (apical whorls/ juveniles, not paratypes); same data as for holotype; UF 583724 • 3 shells (juveniles/damaged, not paratypes); same data as for holotype; locality code KA-0674; UF 583725.

##### Type locality.

Thailand, Suratthani Province, limestone outcrop along Hwy. 401, 2.7 km W junc Hwys. 4142 & 401, evergreen forest on rocky hillside, below cliff, base of cliff; 9°10'N, 99°40'E; 90 m a.s.l.

##### Diagnosis.

A *Hypselostoma* species with roughly granulated (not spirally striated) teleoconch, and keeled, detached, and ascending last whorl. Apertural barriers few (five main ones and usually three smaller ones).

##### Description.

Shell depressed, concave-conical (due to the strongly enlarged last whorl), chestnut brown and weakly glossy, consisting of 3.75–4.25 convex whorls separated by a deep suture. Protoconch is of the same colouration as the teleoconch, finely pitted and showing no spiralling pattern. Boundary between the protoconch and the teleoconch not clear under microscope because of the very similar surface sculpture of the two regions, but the former is ~ 1.5 whorls. Teleoconch roughly granulated (sandpaper-like), weakly sculptured with radial growth lines but devoid of spiral striation (these radial lines are much harder to observe than the prominent granulation). Occasionally, thicker whitish radial streaks are unevenly placed across the surface of the shell. Last whorl with a very blunt keel (which is getting stronger near the aperture) positioned at the centre of the periphery. Shell surface is immediately below and above the keel concave, and then again convex towards the suture and the umbilicus. Last whorl is also very slightly detached from the penultimate and moderately ascending upwards (~ 40–50 ° compared to the shell axis). Peristome dirty white, expanded but not reflected, its surface very finely pitted. There are altogether five main apertural barriers (angular, parietal, upper palatal, lower palatal and columellar) and several smaller ones of variable number. Parietal lamella is the strongest and highest in the aperture, straight but directed towards the palatal side. Angular lamella ~ 2 × lower and shorter than the parietal, but extends closer to the expanding peristome, almost reaching it. Upper and lower palatal plicae almost the same in height and length, the former may be slightly weaker than the latter. Columellar lamella horizontal, almost identical to the lower palatal plica. Among the smaller barriers, there is usually one interpalatal plica which is very small, dot-like. Slightly larger basal plica is present nearly halfway between the lower palatal and columellar. One dot-like swelling is present in the columello-parietal transition embayment. Surface of all apertural barriers is very finely spiniferous medially and very finely granulated laterally. Sinulus wide, not distinctly separated from the rest of the aperture. Umbilicus wide, measuring slightly > 1/4 of the shell width. A shallow groove runs alongside the umbilicus but is visible only around the last quarter of the last whorl.

##### Differential diagnosis.

Less depressed forms of *H.fortunatum* sp. nov. can resemble this species but the barriers in the former are much weaker and angular and parietal lamellae are concrescent. See also under *H.troglodytes*.

##### Measurements

**(in mm, *n* = 5).**SH = 1.73–2.06; SW1 = 2.49–2.85; SW2 = 1.63–1.79; AH = 0.93–1.19; AW = 0.94–1.10.

##### Etymology.

The specific epithet *vicinum* comes from the Latin word for neighbour. This name is provided because the most similar species to the new one, *H. troglodytes*, lives in the neighbouring Peninsular Malaysia.

##### Distribution.

This species is known only from the type locality.

#### ﻿ Hypselostoma vujici

Taxon classificationAnimaliaStylommatophoraHypselostomatidae

Gojšina & Páll-Gergely sp. nov.

BC7FB5AE-88D6-5311-87EF-B079DAE51A20

https://zoobank.org/BA1D33AD-35A2-4B32-895D-9B4F49BFCFD0

**Figure 221.**
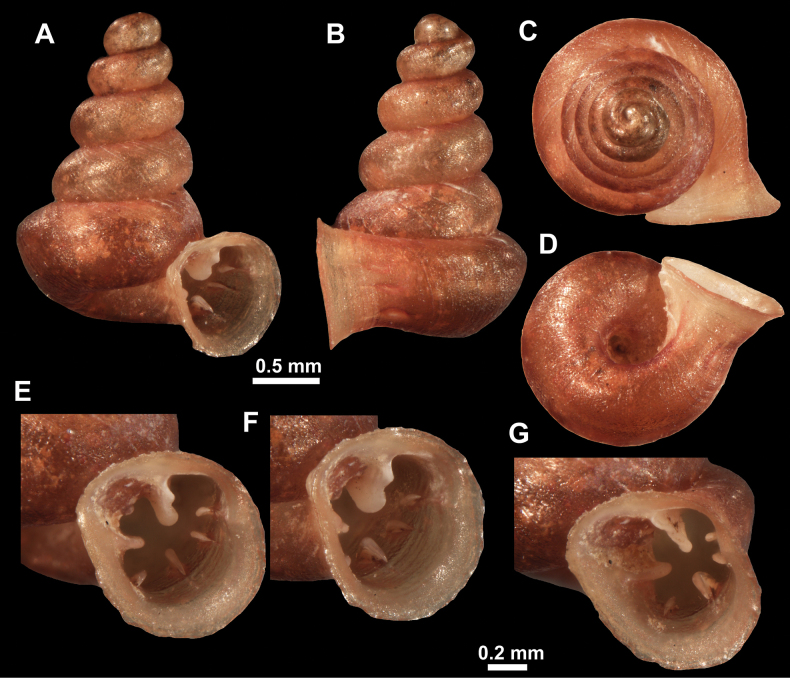
*Hypselostomavujici* Gojšina & Páll-Gergely, sp. nov., holotype (UF 528941) **A–D** shell **E–G** enlarged apertural views.

**Figure 222.**
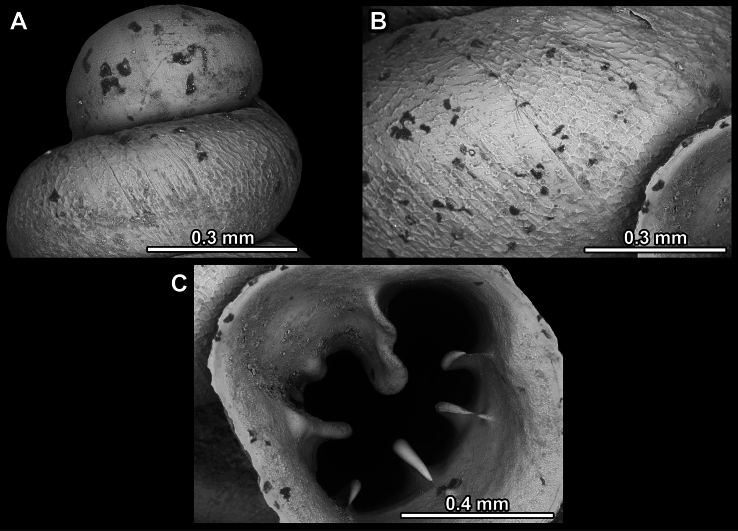
SEM imaging of *Hypselostomavujici* Gojšina & Páll-Gergely, sp. nov., holotype (UF 528941) **A** protoconch surface **B** teleoconch surface **C** enlarged apertural view.

**Figure 223.**
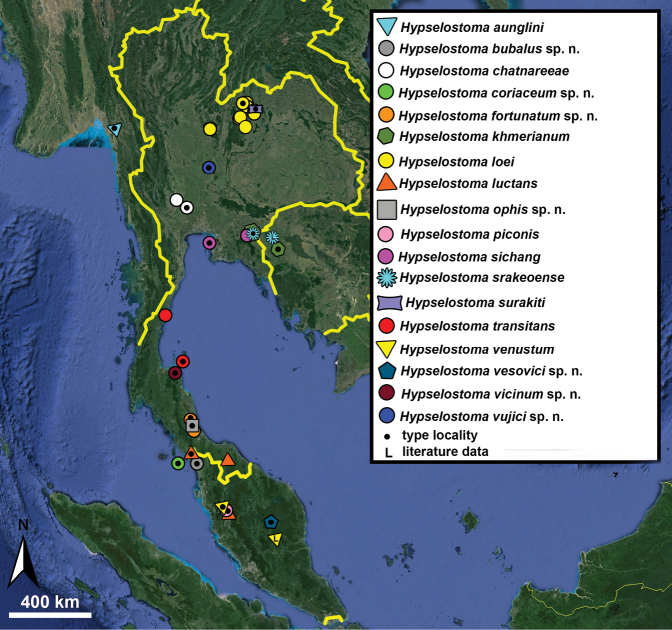
Distribution map of species belonging to *Hypselostomahungerfordianum* group.

Figs 186S , 221 , 222 , 223

##### Type material.

***Holotype*. Thailand** • 1 shell (SH: 2.47 mm; SW: 1.93 mm); Nakhon Sawan Province, 4.0 km NW of Ban Non San; 15°942'N, 99°873'E; 100 m a.s.l.; 12 June 1987; F.G. Thompson leg.; UF 528941. ***Paratypes*. Thailand** • 1 shell; same data as for holotype; CUMZ 14469 • 2 shells; same data as for holotype; UF 591364 • 8 shells (2 whole, others badly damaged, paratypes); same locality data as for holotype; locality code FGT-4325, UF 530691.

##### Additional material examined.

**Thailand** • 3 shells (2 juveniles, 1 last whorl with damaged aperture, not paratypes); same data as for holotype; UF 583723.

##### Type locality.

Thailand, Nakhon Sawan Province, 4.0 km NW of Ban Non San; 15°942'N, 99°873'E; 100 m a.s.l. (provided coordinates are wrong, they cannot be understood by Google Earth).

##### Diagnosis.

A *Hypselostoma* species with 5.75–6 roughly granulated whorls and a very deep suture. Last whorl somehow canted and adnate to the penultimate near the aperture. Palatal plicae and basal plica hooked. Other barriers (angulo-parietal, columellar and infraparietal) blunt. Umbilicus narrow.

##### Description.

Shell high, concave-conical, shiny brown or even colourless, consisting of 5–5.5 whorls separated by a very deep suture. Protoconch almost smooth, with very weak spiralling pattern, consisting of ~ 1.5 whorls but its boundary with the teleoconch is not clearly visible due to the similar surface sculpture. Teleoconch with weak and coarse radial growth lines, which are on the last two whorls visible as white streaks, and rough granulation (sandpaper-like) but no spiral striation. Initial teleoconch whorls rounded, penultimate only very slightly less rounded. Last whorl adnate to slightly detached from the penultimate, with a very weak keel situated near the centre of the periphery. It is slightly descending near the aperture (~ 5–10 ° compared to the shell axis). Peristome dirty white, expanded but not reflected. Aperture rounded (almost as wide as high), equipped with six main barriers (angulo-parietal, upper palatal, interpalatal, lower palatal, basal and columellar) and one weak infraparietal swelling-like lamella. Angulo-parietal lamella is the strongest in the aperture, with clearly distinguishable angular and parietal part and a prominent sinuation between them. Parietal part stronger and higher than the angular, both curved towards the palatal wall. All palatal plicae hooked, pointing outside. Upper palatal and interpalatal plica positioned close together, almost equally as strong. Lower palatal plica stronger than others and situated some distance from the interpalatal. Basal plica also hooked but less clearly than the palatals, strong as the interpalatal and upper palatal but situated closer to the columellar lamella than to the lower palatal plica. Columellar lamella not hooked, long and almost horizontal. A small swelling-like infraparietal lamella is present. Angulo-parietal lamella is reaching the expanding peristome, columellar lamella almost reaching it. Surface of all apertural barriers is finely granulated. Sinulus wide, not distinctly separated from the rest of the aperture. Umbilicus always open but of variable width, measuring ~ 1/6 or ≤ 1/3 of the shell width. Umbilical groove is strong in specimens with wider and weak in specimens with narrower umbilicus.

##### Differential diagnosis.

This species can be separated from all other congeners by the combination of a slender shell with a larger number of whorls (usually ~ 6), roughly granulated teleoconch and hooked basal and three palatal plicae in the aperture.

##### Measurements

**(in mm, *n* = 4).**SH = 2.36–2.62; SW = 1.78–1.93; AH = 0.92–0.96; AW = 0.85–0.93.

##### Etymology.

Named after Mihailo Vujić, a prominent Serbian entomologist and a friend of the first author.

##### Distribution.

This species is known only from the type locality.

##### Remarks.

We have noticed significant intraspecific variability in this species: there were some specimens with elongated-conical, brown shell and some with more depressed spire or much lighter (sometimes even colourless) shell. Umbilicus varied from 1/6 to 1/3 of the shell width with weak or sometimes deep and strong groove inside it.

###### ﻿3. *Hypselostomaterae* group

**Diagnosis.** This species group is characterised by the fine, pasty surface sculpture (like floury dough). Apertural barriers are never spiniferous, never hooked and not numerous: apart from the main five barriers (angular, parietal, two palatals, and a columellar), there are usually up to two more small barriers.

**Remarks.** This group includes 11 species. All of them inhabit Borneo, Peninsular Malaysia, and adjacent regions of Thailand. The morphology of the species within this group agrees well with the distribution pattern.

#### ﻿ Hypselostoma elephas

Taxon classificationAnimaliaStylommatophoraHypselostomatidae

van Benthem Jutting, 1950

90B0F1AA-1BE2-571A-BB7A-656C2E813548

**Figure 224.**
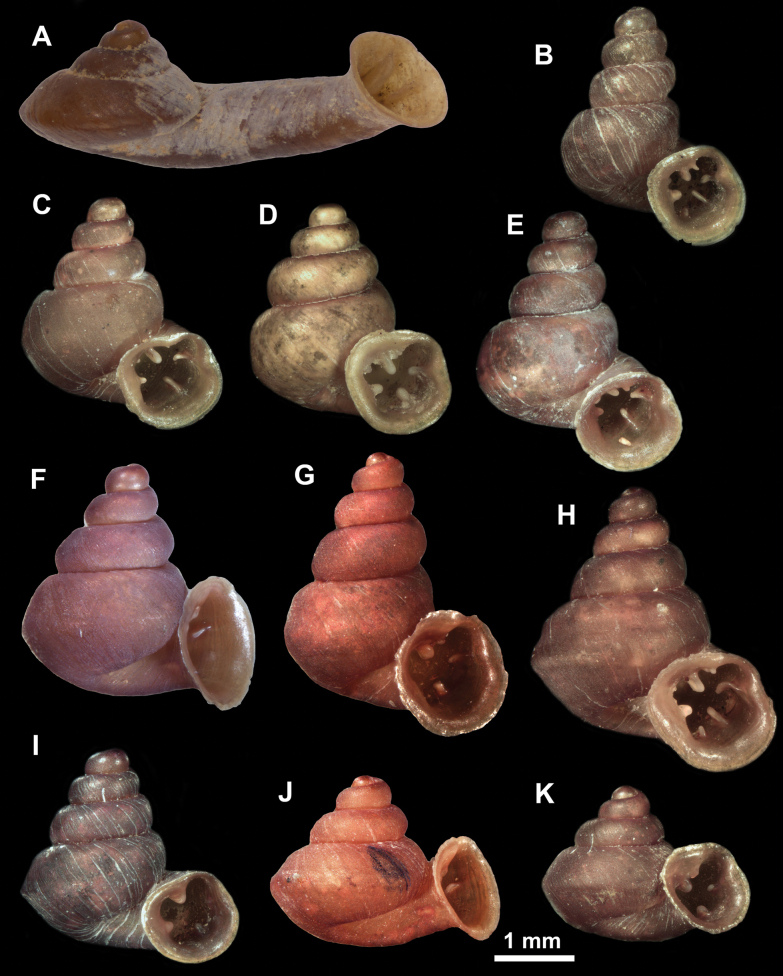
Synoptic view of species belonging to the *Hypselostomaterae* group **A***H.elephas***B***H.emergens***C***H.frequens***D***H.kelantanense***E***H.modestum***F***H.paini***G***H.procerum***H***H.serpa***I***H.terae***J***H.tertiusfrater* nom. nov. **K***H.troglodytes*.

**Figure 225.**
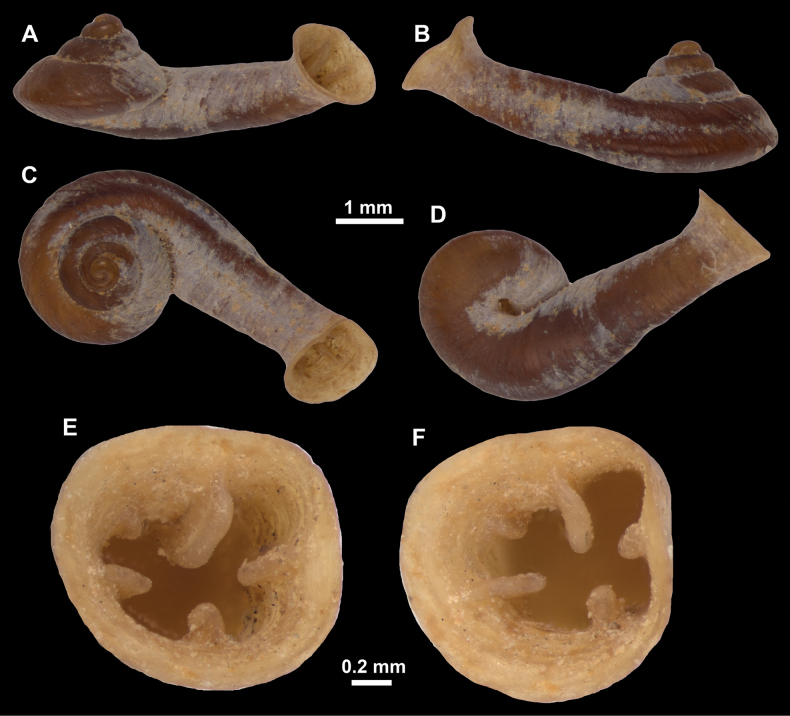
*Hypselostomaelephas*, holotype (RMNH.Moll.137159) **A–D** shell **E, F** enlarged apertural views.

Figs 224A , 225 , 241

Hypselostoma elephas van Benthem Jutting, 1950: 23–24, fig. 12.
Hypselostoma elephas — van Benthem Jutting 1950: 41; Maassen 2001: 77.
Boysidia (Dasypupa) elephas — Thompson and Dance 1983: 109.

##### Type material examined.

**Malaysia** • holotype; RMNH.Moll.137159.

##### Type locality.

“Bukit Tenggek, Pahang”, Malaysia.

##### Differential diagnosis.

See under *H.terae*.

##### Distribution.

This species in known from Bukit Tenggek and Gunung Sagu (IUCN, 2015).

##### Remarks.

This species is critically endangered due to the heavy quarrying of localities where it occurs (Clements 2009; IUCN 2015).

#### ﻿ Hypselostoma emergens

Taxon classificationAnimaliaStylommatophoraHypselostomatidae

(van Benthem Jutting, 1950) comb. nov.

956C9AEC-0FC2-5B8A-ABB2-932BFBB74568

**Figure 226.**
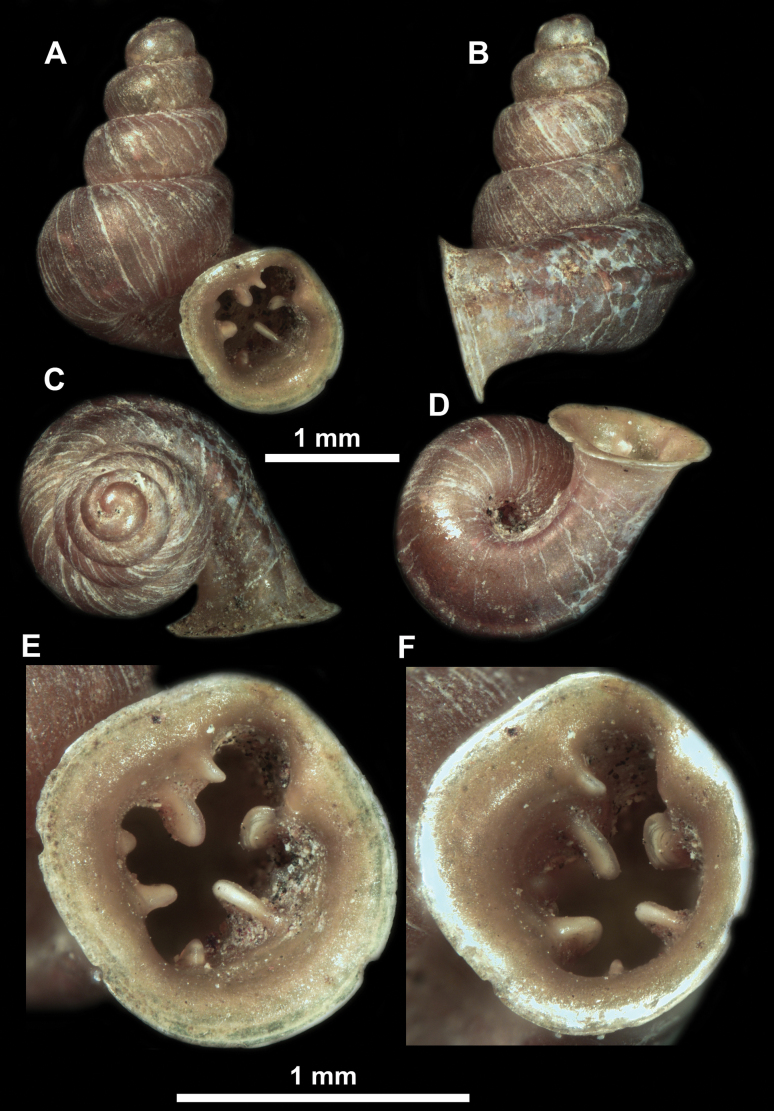
*Hypselostomaemergens*, paratype (RMNH.Moll. 137150) **A–D** shell **E, F** enlarged apertural views.

Figs 224B , 226 , 241

Gyliotrachela emergens van Benthem Jutting, 1950: 34–35, fig. 20.
Gyliotrachela emergens — van Benthem Jutting 1950: 45; Zilch 1984: 165; Maassen 2001: 75.

##### Type material examined.

**Malaysia** • 54 paratypes; from the type locality; 1939; Raffles Museum, Singapore ex. coll.; RMNH.Moll.137150.

##### Additional material examined.

**Malaysia** • 2 shells; Perlis, ca 2 km S of Bukit Keteri (NE of Kangar); 22 Mar. 1998; Hemmen leg.; coll. PGB.

##### Type locality.

“Bukit Chuping, Perlis”, Malaysia.

##### Differential diagnosis.

See under *H.modestum*.

##### Distribution.

This species is known from Bukit Chuping (type locality) and Bukit Keteri (this study), both located in Perlis, N Peninsular Malaysia.

##### Remarks.

Some of the paratypes have more or less protruding and detached last whorl. Also, variability is observed in the keel near the aperture, it can be more or less sharp/pronounced.

#### ﻿ Hypselostoma frequens

Taxon classificationAnimaliaStylommatophoraHypselostomatidae

(van Benthem Jutting, 1950) comb. nov.

7ABF7A5A-9DF3-5033-8C98-46886C710393

**Figure 227.**
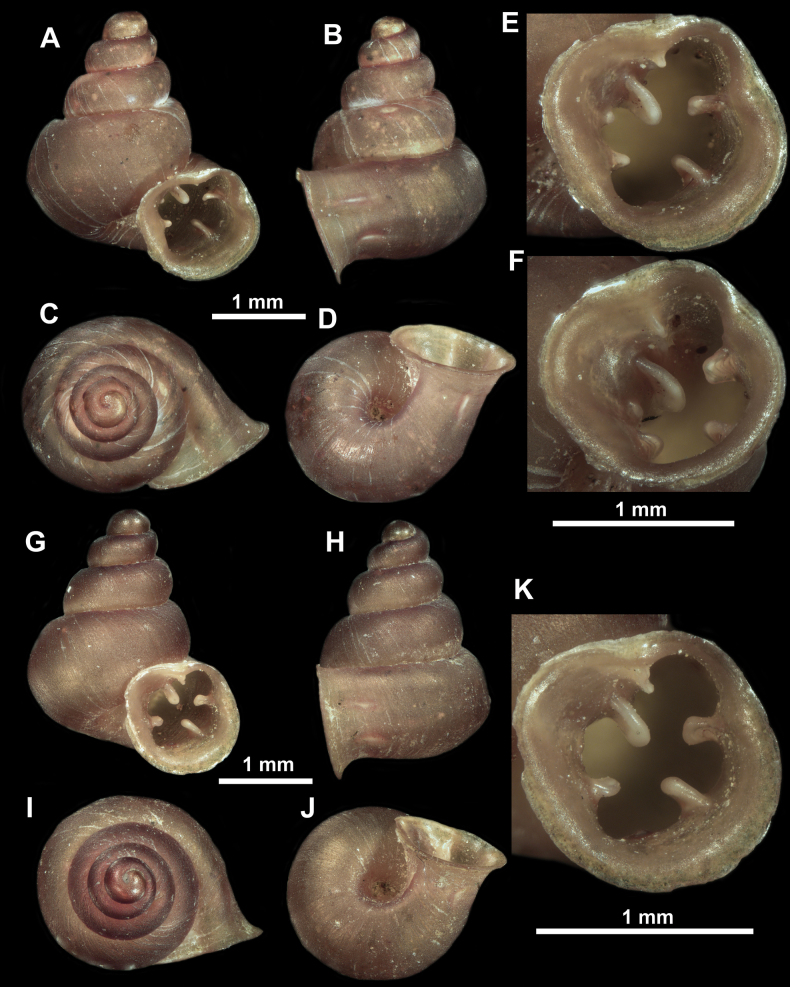
*Hypselostomafrequens*. **A–F** paratype of *H.frequens* (RMNH.Moll.137114) **G–K** paratype of *P.oreia* (RMNH.Moll.137132) **A–D, G–J** shell **E, F, K** enlarged apertural views.

Figs 224C , 227 , 241

Paraboysidia frequens van Benthem Jutting, 1950: 17, fig. 7.
Paraboysidia frequens — van Benthem Jutting 1950: 38; van Benthem Jutting 1960: 14; van Benthem Jutting 1961: 35; Berry 1963: pl. 5, fig. 40; Zilch 1984:165; Maassen 2001: 73–74; Foon and Marzuki 2023: 142, fig. 1AQ.
Paraboysidia oreia van Benthem Jutting, 1961: 37, pl. 9, fig. 3. syn. nov.
Gyliotrachela frequens — Schilthuizen et al. 1999: 284, fig. 2.
Paraboysidia oreia — Maassen 2001: 75; Foon et al. 2017: 79–80, fig. 30D.

##### Type localities.

“Kota Tongkat, Pahang”, Malaysia (*H.frequens*); “Gunong Batu Kurau, Perak”, Malaysia (*P.oreia*).

##### Type material examined.

**Malaysia** • 48 paratypes of *Paraboysidiafrequens*; from the type locality; 1947; Raffles Museum, Singapore ex. coll.; RMNH.Moll.137114 • 23 paratypes of *Paraboysidiaoreia*; from the type locality; RMNH.Moll.137132.

##### Additional material examined.

**Malaysia** •1 shell; Perak, Ipoh, Perak Tong Temple; Oct. 1998; Hemmen leg.; coll. PGB • 5 shells; Selangor, Batu Caves (N of Kuala Lumpur); Nov. 1998; Hemmen leg.; coll. PGB • 1 shell; Selangor, Templer Park, Bukit Takun (NNW of Kuala Lumpur); Nov. 1998; Hemmen leg.; coll. PGB • 4 shells; Kedah, Gunung Keriang (N of Alor Setar); Sept. 1999; Hemmen leg.; coll. PGB.

##### Differential diagnosis.

See under *H.kelantanense*, *H.modestum*, *H.muaklekense* and *H.serpa*. This species also similar to the distant *Bensonellaperfecta* sp. nov. from which it differs by less numerous barriers on the palatal side, the absence of the palatal tubercle on the peristome edge, non-spiniferous apertural barrier surface, less conical shell, and very slightly narrower umbilicus.

##### Distribution.

This species is known from Pahang, Kedah, Perak, and Selangor provinces of Peninsular Malaysia. Possibly more widely spread.

##### Remarks.

In the original description of *Paraboysidiaoreia*, van Benthem-Jutting (1961) mentioned that it differs from *H.frequens* by the less numerous barriers, less inflated and more rounded last whorl. During our examination of the type material, we found no differences between the two species: the last whorl was not more inflated nor more rounded. The apertural barriers were sometimes more numerous in *H.frequens* but this is only true for smaller and more variable barriers which are of none or very limited taxonomic importance. Therefore, we treat *P.oreia* as a junior synonym of *H.frequens*.

#### ﻿ Hypselostoma kelantanense

Taxon classificationAnimaliaStylommatophoraHypselostomatidae

(Sykes, 1902) comb. nov.

67FBD2C2-6B6E-5F39-9C99-D0BC2039C6A9

**Figure 228.**
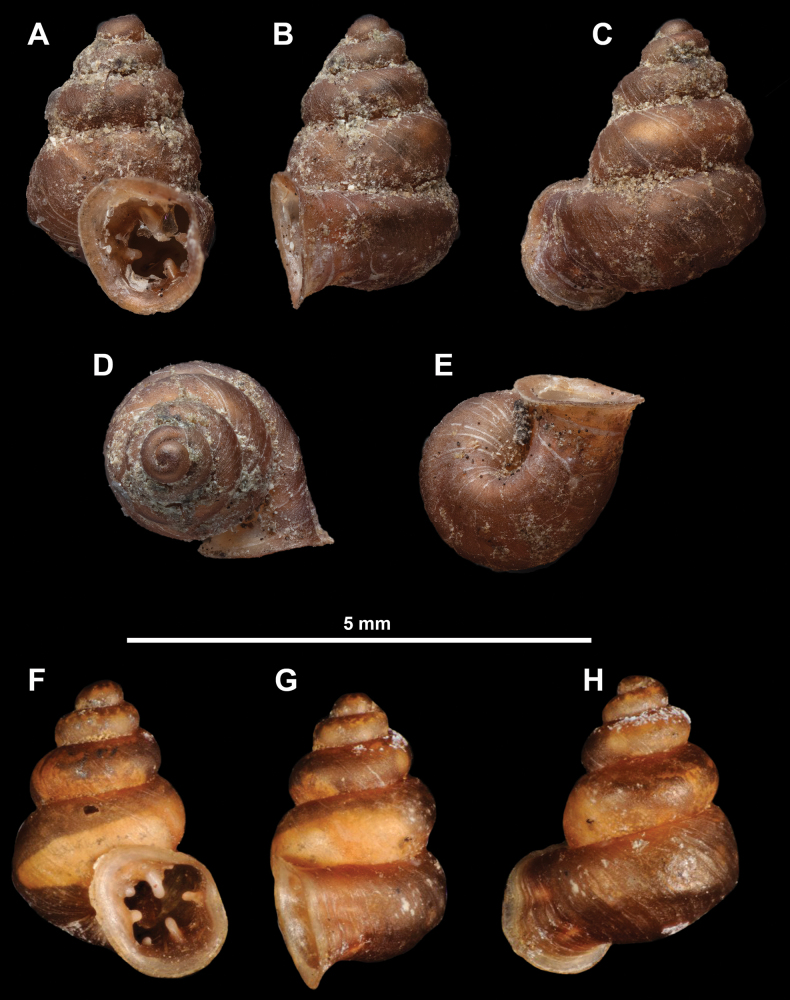
*Hypselostomakelantanense*, syntypes **A–E**NHMUK 1930.9.12.13–15 **F–H**ANSP 98191.

**Figure 229.**
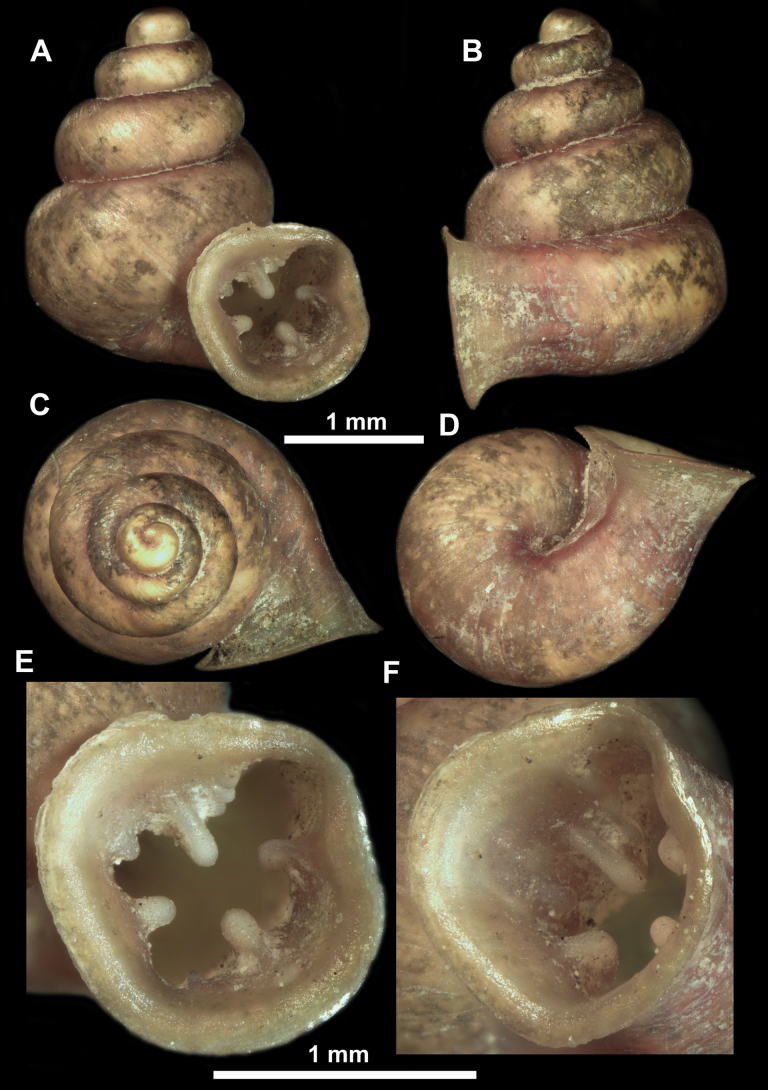
*Hypselostomakelantanense*, paratype of *P.kelantanensisrafflesi* (RMNH.Moll.137125) **A–D** shell **E, F** enlarged apertural views.

**Figure 230.**
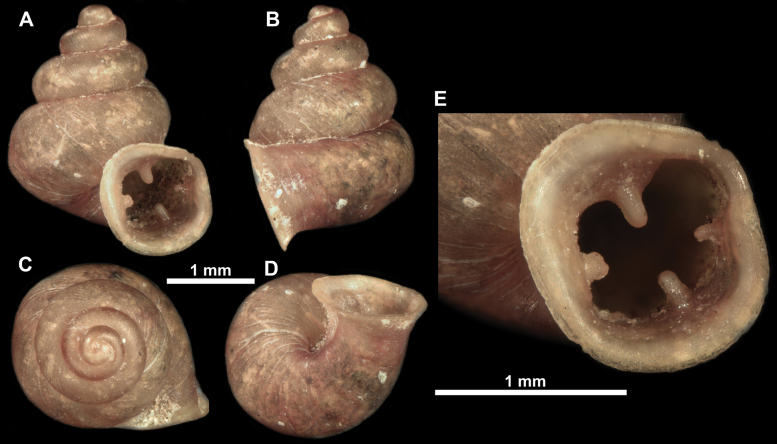
*Hypselostomakelantanense*, paratype of *P.kelantanensistenuidentata* (RMNH.Moll.137127) **A–D** shell **E** enlarged apertural view.

Figs 224D , 228 , 229 , 230 , 241

Boysidia kelantanense Sykes, 1902: 61, pl. 3, fig. 7.
Boysidia kelantanensis — Möllendorff 1902: 139; Pilsbry 1917: 208, pl. 35, figs 10–12.
Paraboysidia kelantanensis — Laidlaw 1933: 213–214.
Paraboysidia kelantanensis kelantanensis — van Benthem Jutting 1950: 12–13, 39, fig. 4; Maassen 2001: 74.
Paraboysidia kelantanensis rafflesi van Benthem Jutting, 1950: 13–14, 39, fig. 5. syn. nov.
Paraboysidia kelantanensis tenuidentata van Benthem Jutting, 1950: 15–17, 39, fig. 6. syn. nov.
Paraboysidia kelantanensis rafflesi — Maassen 2001: 74.
Paraboysidia kelantanensis tenuidentata — Maassen 2001: 74.

##### Type material examined.

**Malaysia** • 1 syntype of *Paraboysidiakelantanensiskelantanensis*; NHMUK 1930.9.12.13–15 • syntypes of *Paraboysidiakelantanensiskelantanensis*; Kelantan; UF 00113222 • 4 paratypes of *Paraboysidiakelantanensisrafflesi*; from the type locality; 1947; Raffles Museum, Singapore ex. coll.; RMNH.Moll.137125 • 1 paratype of *Paraboysidiakelantanensistenuidentata*; from the type locality; Mar. 1939; Raffles Museum, Singapore ex. coll.; RMNH.Moll.137127. Syntypes of *Paraboysidiakelantanensiskelantanensis*; ANSP 98191.

##### Type localities.

“Kelantan”, Malaysia (*H.kelantanensiskelantanensis*); “Kota Tongkat, Pahang”, Malaysia (*H.kelantanensisrafflesi*); “Kramat Pulai, Perak”, Malaysia (*H.kelantanensistenuidentata*).

##### Differential diagnosis.

This species is in terms of apertural dentition and surface sculpture similar to *H.frequens*. However, it has a more rounded shell with more bulging whorls and narrower umbilicus than previous species. See also under *H.serpa*.

##### Distribution.

This species is known from Kelantan, Pahang, and Perak provinces in Peninsular Malaysia.

##### Remarks.

We observed no crucial differences between the two subspecies described by van Benthem Jutting (1950) and the nominotypical subspecies. Furthermore, one paratype of *P.kelantanensisrafflesi* had the identical shell morphology as that of *P.kelantanensistenuidentata* which indicates that the differences mentioned by van Benthem Jutting (1950) (strength of the apertural barriers and overall shell shape) can be considered as a part of intraspecific variability. Some of the examined type specimens are more slender than others. Additionally, some of them had a small lamella between parietal and columellar, while some of them did not.

#### ﻿ Hypselostoma modestum

Taxon classificationAnimaliaStylommatophoraHypselostomatidae

(van Benthem Jutting, 1950) comb. nov.

E46B9263-7248-53E2-947D-2A28B51E5DDC

**Figure 231.**
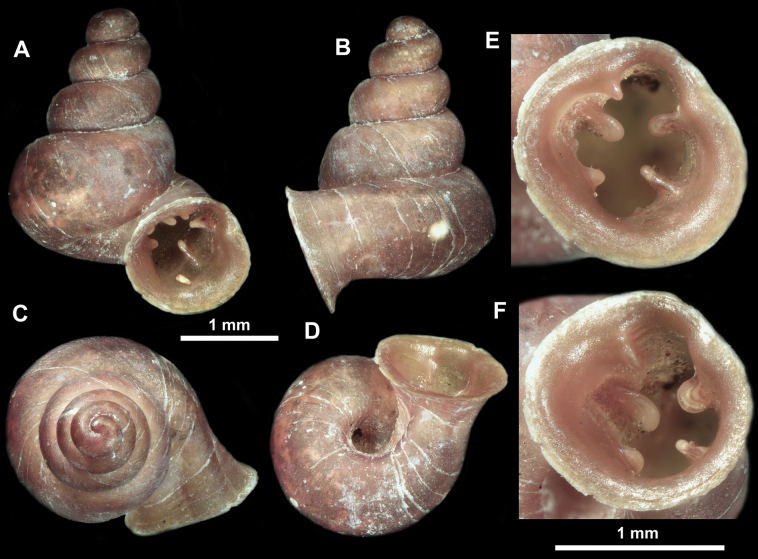
*Hypselostomamodestum*, paratype (RMNH.Moll.137148) **A–D** shell **E, F** enlarged apertural views.

Figs 224E , 231 , 241

Gyliotrachela modesta van Benthem Jutting, 1950: 31–32, fig. 18.
Gyliotrachela modesta — van Benthem Jutting 1950: 46; Davison and Kiew 1990: 25; Maassen 2001: 76.

##### Type material examined.

**Malaysia** • 2 paratypes; from the type locality; 1939; Raffles Museum, Singapore ex. coll.; RMNH.Moll.137148.

##### Type locality.

“Gua Musang, Kelantan”, Malaysia.

##### Differential diagnosis.

This species is very similar to *H.emergens* from which it differs in its narrower last whorl (but also the entire shell) which is also more detached and protruding. The keel on the last whorl is also stronger in *H.emergens* (best seen in the lateral view). *Hypselostomafrequens* has a narrower umbilicus and more enlarged last whorl when compared to the previous ones, which makes the shell less slender. See also under *H.muaklekense* and *H.burchi*.

##### Distribution.

This species is known only from the type locality.

#### ﻿ Hypselostoma paini

Taxon classificationAnimaliaStylommatophoraHypselostomatidae

(F.G. Thompson & Dance, 1983) comb. nov.

56768333-0489-5855-ADBD-69D06A8CB2DC

**Figure 232.**
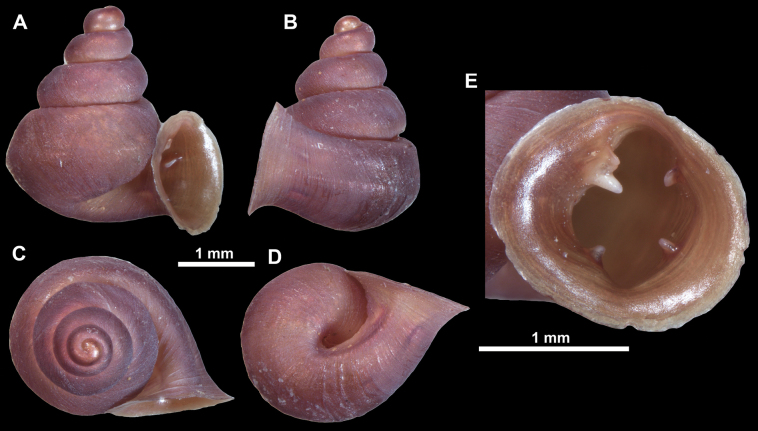
*Hypselostomapaini*, holotype (UF 35949) **A–D** shell **E** enlarged apertural view.

Figs 224F , 232 , 239

Boysidia (Dasypupa) paini Thompson & Dance, 1983: 107–108, figs 9, 10.
Boysidia paini — Dumrongrojwattana and Assawawattagee 2018: 216.

##### Type material examined.

**Malaysia** • holotype; UF 35949 • 6 paratypes; from the type locality; UF 35951.

##### Additional material examined.

**Malaysia** • 4 shells; Borneo, Sarawak, Fifth division, SW flank Gunong Budah, Medalam Valley, trib. Of Limbang R.; UF 35950.

##### Type locality.

“Foot of cliff about 400 yards north of the main to Deer Cave, Melinau Paku Valley, Fourth Div., Sarawak, Borneo, 04°5'N, 114°53'E”.

##### Differential diagnosis.

See under *H.procerum* and *H.tertiusfrater* nom. nov.

##### Distribution.

This species is known only from Sarawak, Borneo.

#### ﻿ Hypselostoma procerum

Taxon classificationAnimaliaStylommatophoraHypselostomatidae

(F. G. Thompson & Dance, 1983) comb. nov.

AF8B9E6C-07B6-5A5A-9511-FBBE4CECAEAF

**Figure 233.**
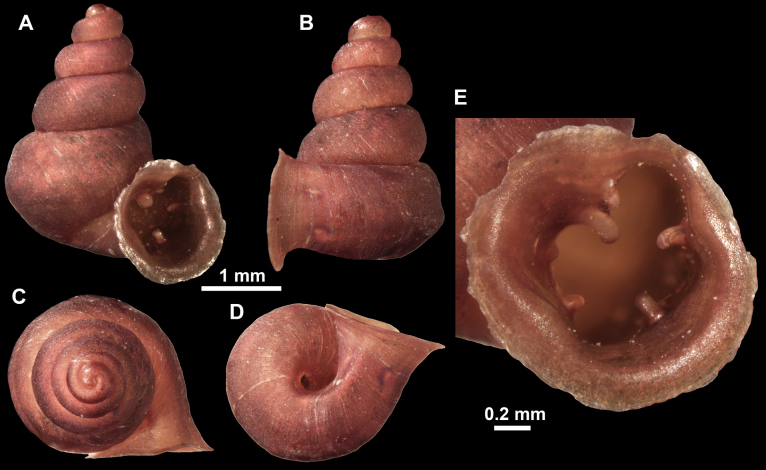
*Hypselostomaprocerum*, holotype (UF 38022) **A–D** shell **E** enlarged apertural view.

Figs 224G , 233 , 239

Boysidia (Dasypupa) procera Thompson & Dance, 1983: 108–109, figs 11, 12.

##### Type material examined.

**Malaysia** • holotype; UF 38022 • 3 paratypes; from the type locality; UF 38023.

##### Type locality.

“Southwest flank of Gunong Budah, Medalam Valley, a tributary of the Limbang river, Fifth Division, Sarawak, Borneo 04°08'N, 115°5'E”.

##### Differential diagnosis.

This species is most similar to *H.paini* which is also described from Borneo. However, this species is more elongated and has a wider umbilicus. See also under *H.tertiusfrater* nom. nov.

##### Distribution.

This species is known only from the type locality.

#### ﻿ Hypselostoma serpa

Taxon classificationAnimaliaStylommatophoraHypselostomatidae

(van Benthem Jutting, 1950) comb. nov.

7ED1CB55-E4BB-5920-83FE-443726198F80

**Figure 234.**
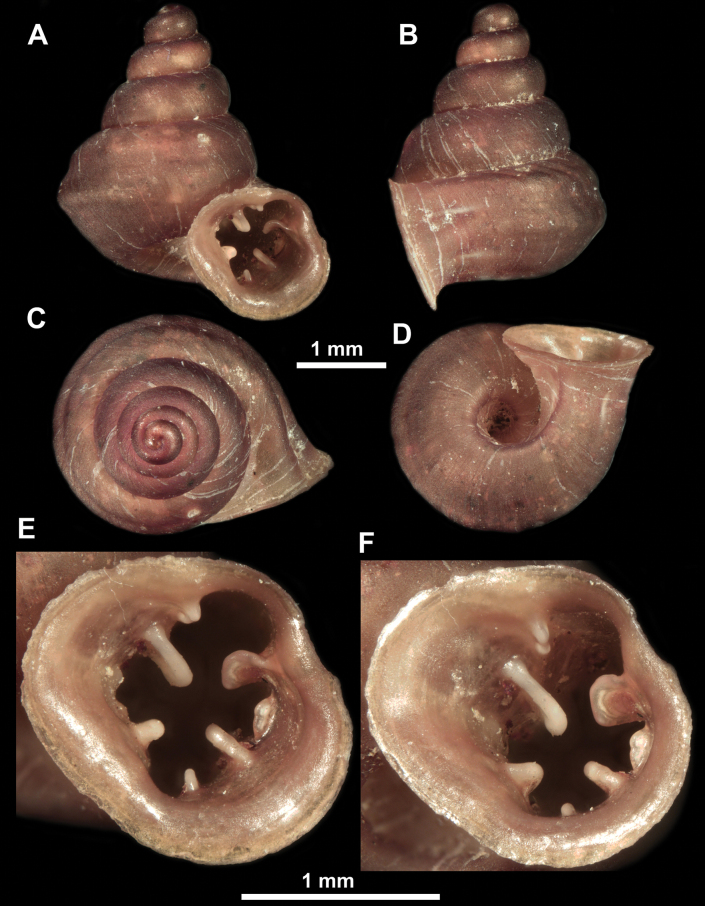
*Hypselostomaserpa*, paratype (RMNH.Moll.140164) **A–D** shell **E, F** enlarged apertural views.

Figs 224H , 234 , 241

Paraboysidia serpa van Benthem Jutting, 1950: 18–19, 40, fig. 8.
Paraboysidia serpa — Maassen 2001: 75.
Paraboysidia tarutao Panha & Burch, 2002c: 85–87, fig. 4. syn. nov.
Gyliotrachela tarutoa [sic] — Panha and Burch 2008: 84, fig. 73.
Gyliotrachela tarutao — Dumrongrojwattana and Wongkamhaeng 2024: 324, fig. 8.

##### Type material examined.

**Malaysia** • 18 paratypes of *H.serpa*; from the type locality; Dec. 1938; Raffles Museum, Singapore ex. coll.; RMNH.Moll.140164. **Thailand** • 1 paratype of *G.tarutao*; from the type locality; 1998; S. Panha leg.; CUMZ ver. 062.

##### Additional material examined.

**Thailand** • 22 shells; Krabi Province, Railay (= Rai Leh) Beach West, Railay Highlands, at the base of limestone rocks; 8°0.865'N, 98°50.219'E; 30 m a.s.l.; Sep. 2007; A. Reischütz leg.; coll. REI • 1 shell; Krabi Province, Phi Phi islands, Phi Phi Don island, climbing rock above the western end of the beach in Ton Sai Bay, at the base of limestone rocks in the forest; 7°44.063'N, 98°45.947'E; 50 m a.s.l.; Mar. 2010; A. Reischütz leg; coll. REI.

##### Type localities.

“Baling, Kedah”, Malaysia (*H.serpa*); “Tarutoa National Park, Satul Province, Thailand, 6°41'58'N, 99°38'48'E, 70 meters elevation” (*P.tarutao*).

##### Differential diagnosis.

*Hypselostomaserpa* is most similar to *H.frequens*, from which it can be separated by the keeled last whorl (rounded in *H.frequens*), the bent upper palatal plica and the wider umbilicus. *Hypselostomakelantanense* has a more ovoid shell shape, more rounded and bulging whorls as well as narrower umbilicus.

##### Distribution.

This species is known from Kedah, Malaysia (the type locality) as well as from Krabi Province and Ko Tarutao Island, Satun province, Thailand (the type locality of *G.tarutao*).

##### Remarks.

In the paratypes, interpalatal plica is simple or bulge like, infraparietal lamella can be present or absent. All examined paratypes have upper palatal plica curved towards the lower one which could be characteristic for this species. *Paraboysidiatarutao* is treated as a junior synonym of this species since no morphological differences were noticed. In the original description (Panha and Burch 2002c), *P.tarutao* was not compared to *H.serpa* which gives the impression that the latter was overlooked.

#### ﻿ Hypselostoma terae

Taxon classificationAnimaliaStylommatophoraHypselostomatidae

Tomlin, 1939

DE00A42C-28DD-5827-AF5C-620726E1EE1F

**Figure 235.**
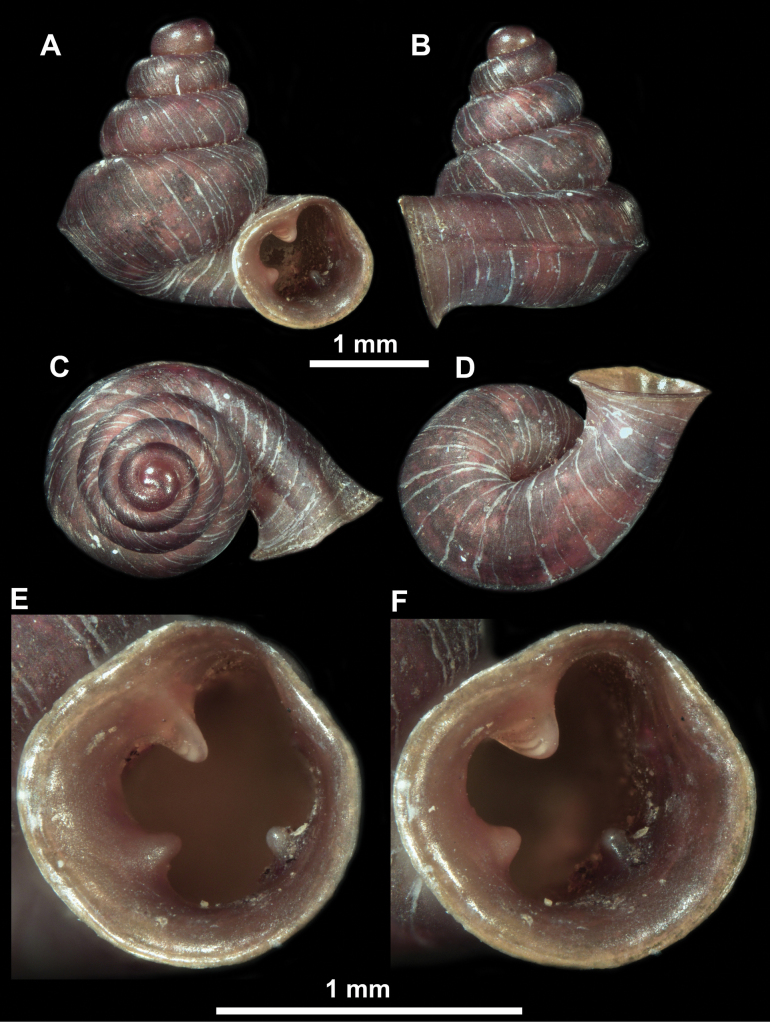
*Hypselostomaterae*, paratype (RMNH Moll.137160) **A–D** shell E, **F** enlarged apertural views.

**Figure 236.**
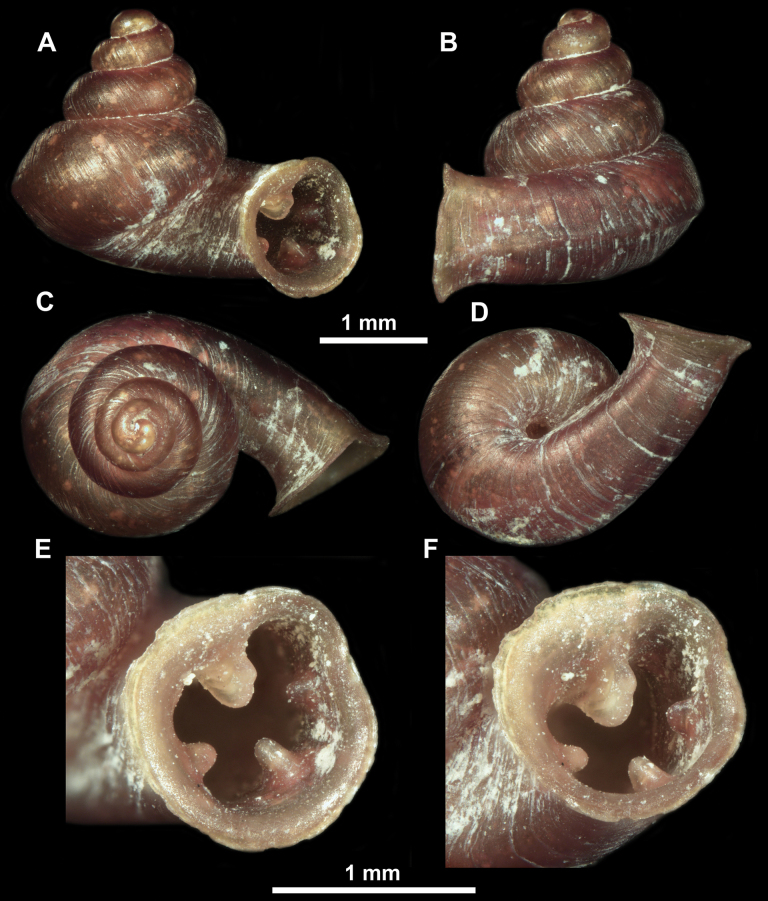
*Hypselostomaperigyra*, paratype (RMNH Moll.137163) **A–D** shell **E, F** enlarged apertural views.

**Figure 237.**
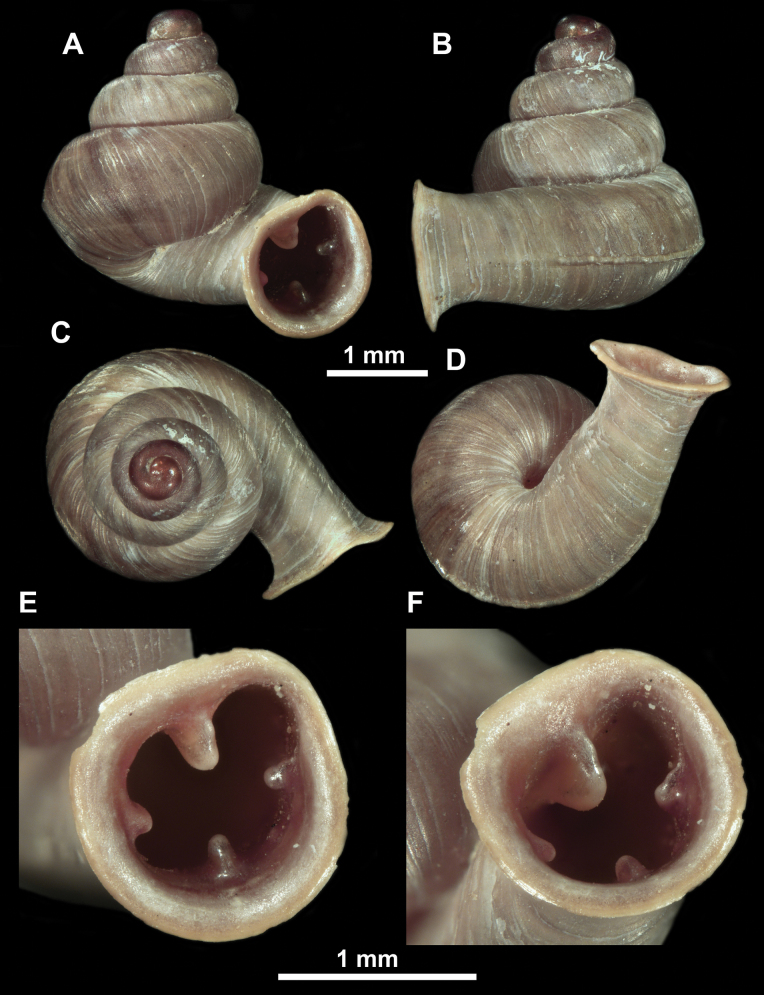
*Hypselostomamegaphonum*, paratype (RMNH Moll.137161) **A–D** shell **E, F** enlarged apertural views.

Figs 224I , 235 , 236 , 237 , 241

Hypselostoma terae Tomlin, 1939: 146, pl. 12, fig. 2.
Hypselostoma terae — van Benthem Jutting 1949b: 59; van Benthem Jutting 1950: 43; van Benthem Jutting 1950: 21–22, fig. 10; Berry 1963: pl.5, fig. 42.
Hypselostoma megaphonum van Benthem Jutting, 1950: 21–23, fig. 11. syn. nov.
Hypselostoma megaphonum — van Benthem Jutting 1950: 42; Maassen 2001: 77.
Hypselostoma perigyra van Benthem Jutting, 1950: 25–26, fig. 14. syn. nov.
Hypselostoma perigyra — van Benthem Jutting 1950: 42; Maassen 2001: 77.
Boysidia (Dasypupa) terae — Thompson and Dance 1983: 109.
Boysidia (Dasypupa) megaphona — Thompson and Dance 1983: 109.
Boysidia (Dasypupa) perigyra — Thompson and Dance 1983: 109.

##### Type material examined.

**Malaysia** • 53 paratypes of *H.terae*; from the type locality; RMNH Moll.137160 • 23 paratypes of *H.perigyra*; from the type locality; RMNH Moll.137163 • 23 paratypes of *H.megaphonum*; from the type locality; RMNH Moll.137161.

##### Additional material examined.

**Malaysia** • 2 shells; Pahang, Bukit Cinta Manis, SSE side, Lebuhraya Karak 800 m - Kampung Cinta Manis; 03°26.714'N, 102°00.814'E; 130 m a.s.l.; 22 Jan. 2013; A. Hunyadi leg.; coll. HA • 2 shells; Pahang, 25 km northwest from Kuantan, east from Kampung Panching, Gua Charas, rock temple; 03°54.692'N, 103°08.839'E; 140 m a.s.l.; 24 Jan. 2013; A. Hunyadi leg.; coll. HA • 1 shell; Selangor, Kual Lumpur E- Kanching, Templer Park, Perangsang Templer Golf Club, limestone rock; 03°17.878'N, 101°38.438'E; 100 m a.s.l.; 25 Jan. 2013; A. Hunyadi leg.; coll. HA • 25 shells; Pahang, 15 km west from Bandar Pusat Jengka, Hutan Lipur Gunung Senyum; 03°41.862'N, 102°25.980'E; 85 m a.s.l.; 23 Jan. 2023; A. Hunyadi leg.; coll. HA.

##### Type localities.

“Bukit Chintamani, Pahang” (Bukit Cinta Manis), Malaysia (*H.terae*); “Bukit Charas, near Kuantan, Pahang”, Malaysia (*H.megaphonum*); “Bukit Takun, Kanching, Selangor”, Malaysia (*H.perigyra*).

##### Differential diagnosis.

This species differs from *H.elephas* by its less detached and protruding last whorl as well as the much more conical shell.

##### Distribution.

This species is known from Pahang and Selangor provinces in Peninsular Malaysia.

##### Remarks.

*Hypselostomamegaphonum* and *H.perigyra* are synonymised with this species based on the following: i) all three species share the same arrangement and morphology of apertural barriers; ii) umbilici of all three species are equally narrow (with very slight variability which can be considered intraspecific); iii) all three species have the same narrow and sharp peripheral keel and iv) all three species are of similar size and distributed geographically close. All differences mentioned by van Benthem Jutting (1950) in the original description (last whorl inflation level, umbilicus width and apertural barrier strength) are recognised as intraspecific variability. *Hypselostomamegaphonum* can be interpreted as an intermediary form between *H.terae* and *H.perigyra* based on the following characters: i) last whorl more inflated than in *H.terae* but less than in *H.perigyra*; ii) apertural barriers stronger than in *H.terae* but weaker than in *H.perigyra*; iii) umbilicus wider than in *H.terae* but narrower than in *H.perigyra*.

#### ﻿ Hypselostoma tertiusfrater

Taxon classificationAnimaliaStylommatophoraHypselostomatidae

Gojšina & Páll-Gergely nom. nov.

41331670-11B9-5CA4-9DC7-D3CF8F5B35DB

**Figure 238.**
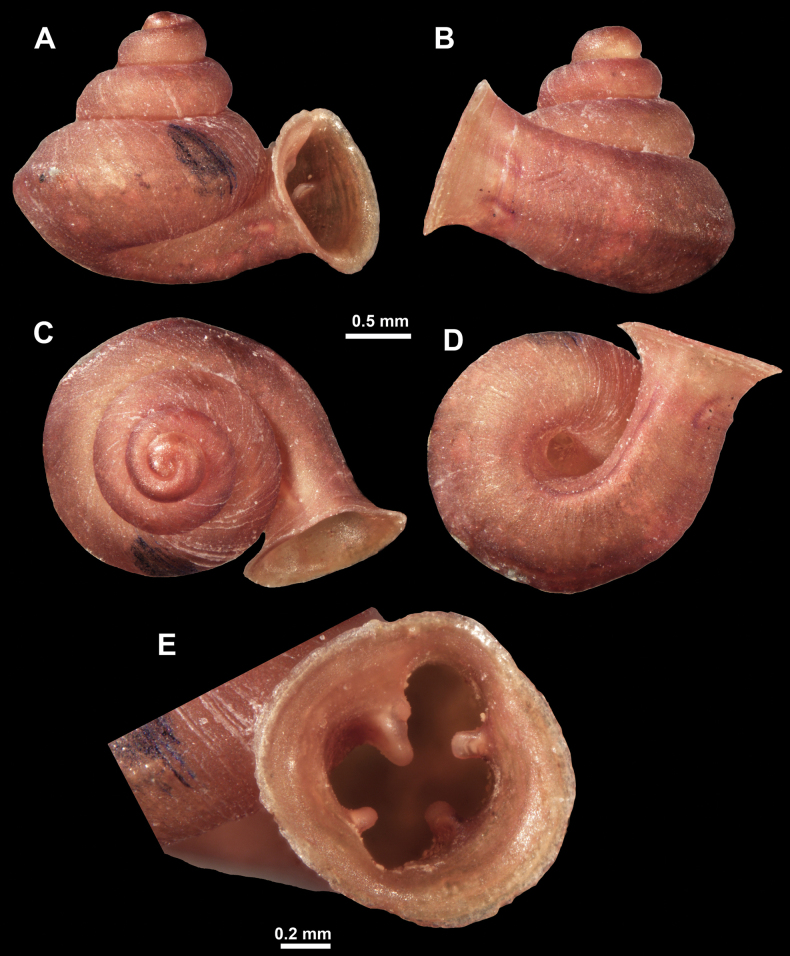
*Hypselostomatertiusfrater* Gojšina & Páll-Gergely nom. nov., holotype (UF 35944) **A–D** shell **E** enlarged apertural view. **A** and **E** are used from Inkhavilay and Sutcharit 2024.

**Figure 239.**
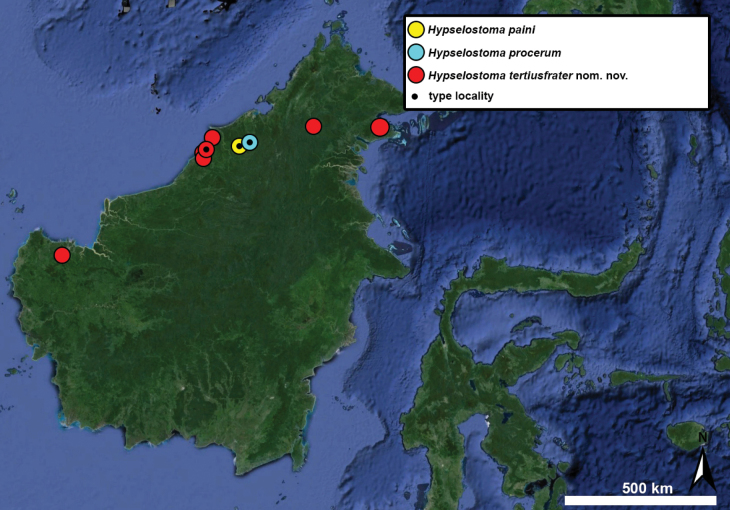
Distribution of species belonging to *Hypselostomaterae* group in Borneo.

Figs 224J , 238 , 239

Boysidia (Dasypupa) salpinx Thompson & Dance, 1983: 106–107, figs 2–8.
Boysidia salpinx — Dumrongrojwattana and Assawawattagee 2018: 216; Marzuki et al. 2021: 103–104, figs 45E, 50B; Inkhavilay and Sutcharit 2024: 441, fig. 1D.

##### Type material examined.

**Malaysia** • holotype; UF 35944 • 33 paratypes; from the type locality; UF 36281/2, used for SEM by F.G. Thompson, destroyed; UF 35946/31.

##### Additional material examined.

**Malaysia** • 3 shells; Borneo, Sarawak, 4^th^ Div., Niah Caves Nat. Park, near Painted Cave; Aug. 2002; coll. PGB • 24 shells; Borneo, Sarawak, SE portion, Bau area, Gunong Kapor, entrance to main cave; UF 35945 • 397 shells; Borneo, Sabah, ca 20 km upstream from Simatuoh, E side of Sapulut R., Batu Punggul; 16. May 1988; D. K. Dorman leg; locality code DKD-91, UF 196670 • 190 shells; Borneo, Sarawak, Fourth division, Niah Caves National park, summit of Gunong Subis, SE Batu Niah; 03°48'N, 113°46′E; 29 Apr. 1988; D. K. Dorman leg.; locality code DKD-77, UF 196488 • 15 shells; same data as previous; UF 196487 • 1 shell; Borneo, Sabah, ca 20 km upstream from Simatuoh, E side of Sapulut R., Batu Punggul; 04°38.8'N, 116°36.7'E; 16. May 1988; D. K. Dorman leg.; locality code DKD-90, UF 196667 • 91 shells; same data as previous; locality code DKD-091; UF 196669 • 26 shells; Borneo, Sarawak, SW portion, S of Serian, E side Gunong Selabor, main entrance Lobang Batu cave; UF 35948 • 25 shells; Borneo, Sarawak, SW portion S side Gunong Selabor, S of Serian; UF 35947 • 3 shells; Sabah, ca. 25 km W Kunak, Baturong Caves; 04°37'N, 118°09'E; 12. Jun.1988; David K. Dorman leg.; locality code DKD-102; UF 196829.

##### Type locality.

“Gunong Subis, limestone massif about 40 mi SW of Mira, Niah area, Fourth Div., Sarawak, Borneo, 03°51'N, 113°45'E”.

##### Differential diagnosis.

This species is clearly different from the two congeners from Borneo (*H.paini* and *H.procerum*) by its depressed-conical shell (vs conical) and a wider umbilicus.

##### Etymology.

The specific epithet is made of *tertius* meaning third and *frater* meaning brother, given the fact that this is one of three *Hypselostoma* species described from Borneo.

##### Distribution.

This species in known from Sarawak and Sabah, Borneo.

##### Remarks.

*Boysidiasalpinx* F.G. Thompson & Dance, 1983 is moved to the genus *Hypselostoma* herein, and thus, becomes a secondary homonym of *Hypselostomasalpinx* (van Benthem Jutting, 1961). Therefore, *Hypselostomatertiusfrater* nom. nov. is proposed as a replacement name.

#### ﻿ Hypselostoma troglodytes

Taxon classificationAnimaliaStylommatophoraHypselostomatidae

(van Benthem Jutting, 1950) comb. nov.

64C84E2F-AE2A-5EC2-AA14-A4B042FB6EA4

**Figure 240.**
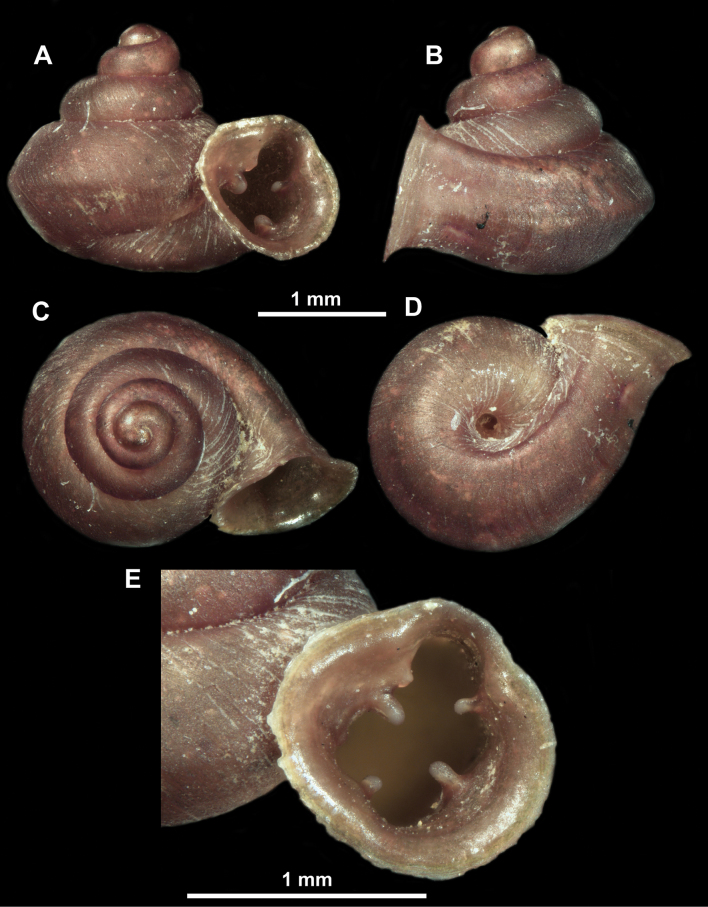
*Hypselostomatroglodytes*, paratype (RMNH.Moll.137138) **A–D** shell **E** enlarged apertural view.

**Figure 241.**
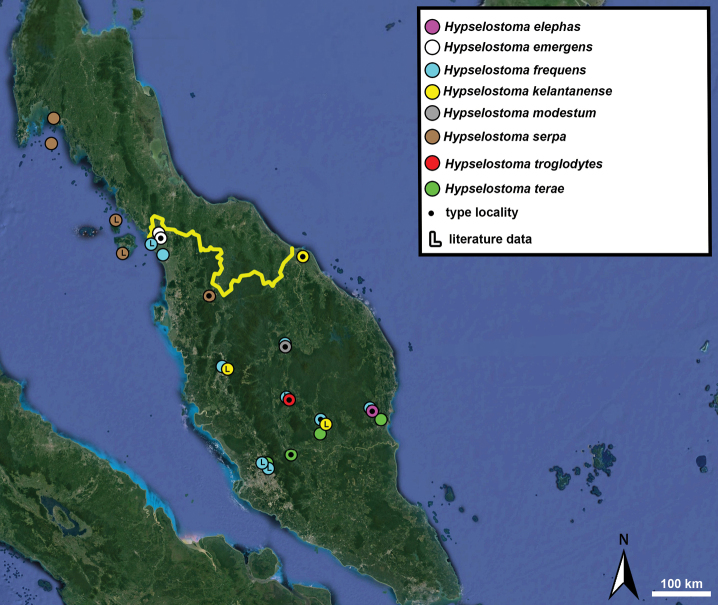
Distribution map of species belonging to *Hypselostomaterae* group.

Figs 224K , 240 , 241

Gyliotrachela troglodytes van Benthem Jutting, 1950: 35–36, 47, fig. 21.
Gyliotrachela troglodytes — Maassen 2001: 77.

##### Type material examined.

**Malaysia** • 3 paratypes; from the type locality; Sept. 1941; Raffles Museum, Singapore ex. coll.; RMNH.Moll.137138.

##### Type locality.

“Gua Bama, Padang Tengku, Pahang”, Malaysia.

##### Differential diagnosis.

This species can be separated from *H.vicinum* sp. nov. by the notably narrower umbilicus, weaker peripheral keel, and the absence of the basal and one interpalatal plica. Furthermore, the shell surface sculpture seems to be less rough than in *H.vicinum* sp. nov.

##### Distribution.

This species is known only from the type locality.

##### Remarks.

In the original description, an infraparietal lamella is mentioned. We observed it in paratypes but it should be noted that it is dot-like and very small.

###### ﻿4. *Hypselostomatubiferum* group

**Diagnosis.***Hypselostomatubiferum* group is devoid of any peculiar shell surface sculpture. It is smooth or finely pitted with some radial growth lines.

**Remarks.** This group includes nine species. Their distribution spans from Guangxi, Guizhou, and Yunnan provinces in China southwards to Peninsular Malaysia. Westernmost localities are known from central Myanmar.

#### ﻿ Hypselostoma burchi

Taxon classificationAnimaliaStylommatophoraHypselostomatidae

(Panha, 1998) comb. nov.

30801580-F371-587F-B602-B63E31FA6065

**Figure 242.**
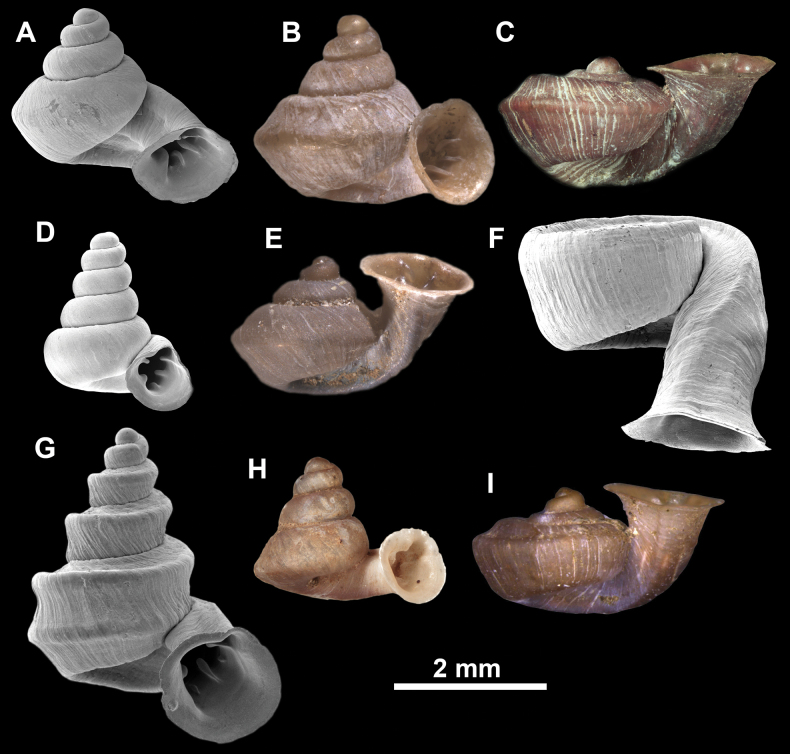
Synoptic view of species belonging to *Hypselostomatubiferum* group **A***H.burchi***B***H.crossei***C***H.depressispira***D***H.muaklekense***E***H.pattalungense***F***H.pendulum***G***H.phupaman***H***H.similare***I***H.tubiferum*.

**Figure 243.**
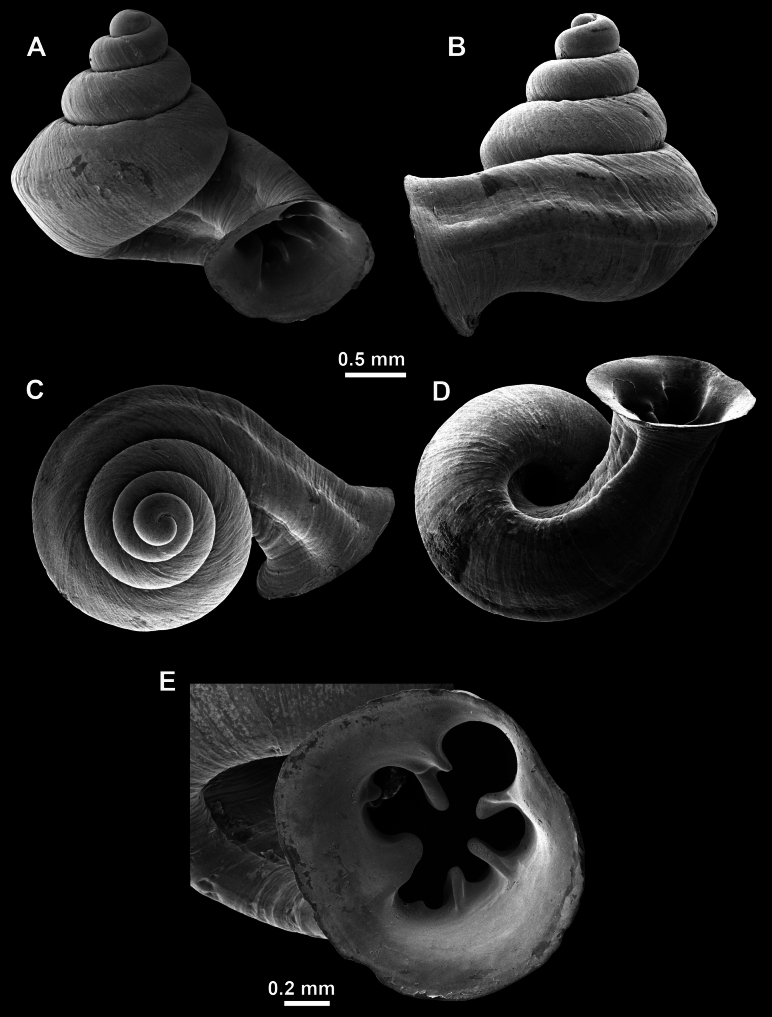
*Hypselostomaburchi*, holotype (CUMZ ver. 015) **A–D** shell **E** enlarged apertural view.

Figs 242A , 243 , 259

Gyliotrachela burchi Panha, 1998c: 123–124, fig. 2.
Gyliotrachela burchi — Hemmen and Hemmen 2001: 41, fig. 9; Panha and Burch 2008: 66–67, fig. 58; Dumrongrojwattana and Wongkamhaeng 2024: 323, fig. 8.

##### Type material examined.

**Thailand** • holotype; 1996; S. Panha leg.; CUMZ ver. 015.

##### Type locality.

“Pattalung province, 7°40′17″N, 100°1′11″E, 70 meters elevation”. (Thailand)

##### Differential diagnosis.

This species is similar in shell shape to *H.taehwani* but the latter is spirally striated and has concrescent angular and parietal lamellae. Also, *H.taehwani* has a stronger keel than *H.burchi* and deeper grooves both above and below the keel. It also resembles *H.modestum* but the last whorl in *H.burchi* is keeled and more strongly detached and descending. The umbilicus is also wider in *H.burchi*.

##### Distribution.

This species is known only from the type locality.

#### ﻿ Hypselostoma crossei

Taxon classificationAnimaliaStylommatophoraHypselostomatidae

Morlet, 1886

9CCDDBD2-0882-511E-8F19-9CB8F5C96193

Figs 242B , 244 , 245 , 246 , 247 , 248 , 249 , 250 , 251

Hypselostoma crossei Morlet, 1886: 2–3.
Hypselostoma crossei — Möllendorff 1891: 338.
Hypselostoma crossei brevituba Möllendorff, 1901b: 76. syn. nov.
Hypselostoma crossei endodonta Möllendorff, 1901b: 76. syn. nov.
Hypselostoma brevituba — Fischer and Dautzenberg 1904: 408.
Hypselostoma crossei endodonta — Fischer and Dautzenberg 1904: 408.
Gyliotrachela crossei — Pilsbry 1917: 215–216; van Benthem Jutting 1950: 44; Inkhavilay et al. 2019: 147, fig. 17I; Thompson and Upatham 1997: 233.
Gyliotrachela crossei brevituba — Pilsbry 1917: 217, pl. 36, figs 9–13; van Benthem Jutting 1950: 45; Zilch 1984: 165; Schileyko 2011: 3.
Boysidia (Bensonella) xingyinensis Guo, Zhou & Luo, 2006: 541–542 (Chinese description), 542–543 (English description), figs 1–4. syn. nov.
Gyliotrachela crossei endodonta — Pilsbry 1917: 217; van Benthem Jutting 1950: 45; Zilch 1984: 165; Schileyko 2011: 3.
Boysidia (Bensonella) tianxingqiaoensis Luo, Chen & Zhang, 2000: 147 (Chinese description), 150 (English description), figs 1–4. syn. nov.
Boysidia (Bensonella) tianxingqiaoensis — Chen and Zhang 2002: 693.
Gyliotrachela crossei crossei — Schileyko 2011: 3.
Gyliotrachela plesiolopa Inkhavilay & Panha in Inkhavilay et al. 2016: 222, figs 1, 3D–F, 4D. syn. nov.
Gyliotrachela plesiolopa — Inkhavilay et al. 2019: 60, fig. 26D.
Boysidia tianxingqiaoensis — Dumrongrojwattana and Assawawattagee 2018: 216.
Boysidia xingyinensis — Dumrongrojwattana and Assawawttagee 2018: 216.
Gyliotrachela tianxingqiaoensis – Gojšina et al. 2022: 132, figs 2–5; Tongkerd et al. 2024: 182, fig. 13N.

##### Type material examined.

**Vietnam** • syntype of *H.crosseicrossei*; coll. Morlet; MNHN-IM-2000-35155 • lectotype of *G.crosseibrevituba*; ex. coll. Fruhstorfer; coll. Möllendorff; SMF 4582 • lectotype of *G.crosseiendodonta*; ex. coll. Fruhstorfer; coll. Möllendorff; SMF 4584. **China** • holotype of *B.tianxingqiaoensis*; 13 Aug. 1997; D.-N. Chen leg.; IZCAS 025075 • holotype of *B.xingyinensis*; 02 Oct. 2004; Guo Y.-H, Zhou W.-C., Luo T.-C. leg.; IZCAS 069205. **Laos** • 2 paratypes of *G.plesiolopa*; from the type locality; ex. coll. S. Panha; SMF 357824.

##### Additional material examined.

**Vietnam** • 1 shell; Cao Bang Province, wet limestone wall; Jul. 2006.; M. Calo leg.; coll. PGB • 1 shell; Ninh Binh Province, Ninh Binh, Hoa Lu Temple; 20°15.738'N, 105°57.886'E; 8 m a.s.l.; 01 Oct. 2009; Hemmen leg; coll. PGB • 1 shell; Hoa Binh Province, ca 15 km rd. Tang Dao to Co Luong (rd #15 left side); 20°35.951'N, 105°01.905'E; 16 Oct. 2011.; Hemmen leg; coll. PGB • 5 shells; Quang Ninh Province, Ha Long Bay area, Tien Ong Cave on Hang Trai Island; 20°48.96'N, 107°07.33'E; 6 Sept. 2003.; Hemmen leg; coll. PGB • 1 shell; Quang Ninh Province, near Dau Go Isl., Halong Bay; 20°53.853'N, 107°00.825'E; 2 m a.s.l.; 18 Nov. 2005; J.U. Otani leg.; coll. PGB • 23 shells; Ninh Binh Province, Nho Quan District, Cuc Phuong N. P., near kiosk at entrance to Cave of the prehistoric man (Dong Nguoi Xua); 20°17.613'N, 105°40.063'E; 247 m a.s.l.; 12 Apr. 2007; K. Okubo & J.U. Otani leg.; coll. PGB • 2 shells; Quang Ninh Province, Halong Bay area, Hang Trai Id., Tieng Ong cave; 20°48.96'N, 107°07.33'E; Hemmen leg; coll. PGB • 15 shells; Hai Phong city, Cat Ba island, Gia Luan Commune; 20°51.402'N, 106°58.961'E; 4 m a.s.l.; 18 Nov. 2005; J.U. Otani leg.; Coll, PGB • 3 shells; Cao Bang Province, Pac Rao, 300 m from junction to Xa Canh Tien towards Trung Khanh, right side of the road; 22°49.385'N, 106°30.742'E; 530 m a.s.l.; 28 May 2012; A. Hunyadi leg.; coll. HA • 2 shells; Lang Son Province, Lang Son, NNE side of Nui Vong Phu; 21°51.183'N, 106°44.950'E; 11 Nov. 2011; A. Hunyadi leg.; coll. HA • 1 shell; Lang Son Province, north of Chi Lang, pass along the path (north of Đong Banh); 21°34.945'N, 106°30.567'E; 75 m a.s.l.; 13 Nov. 2011; A. Hunyadi leg.; coll. HA • 5 shells; Lang Son, vicinity of Chua Tam Thanh; 21°51.353'N, 106°44.809'E; 265 m a.s.l.; 21 Feb. 2020; A. Hunyadi leg.; coll. HA • 1 shell; Thai Nguyen Province, Cho Chu, rock wall at the northeastern part of the village; 21°54.613'N, 105°39.195'E; 90 m a.s.l.; 21 Nov. 2011; A. Hunyadi leg.; coll. HA • 34 shells: Lang Son Province, Lang Son, Thanh Nha Mac, southern group of rocks; 21°51.358'N, 106°45.035'E; 295 m a.s.l.; 26 May 2012; leg. A. Hunyadi; coll. HA • 7 shells; Lang Son Province, Huu Lung district, Minh Tien, Cau Cheo Minh Tien, southern side of the bridge; 21°33.605'N, 106°17.427'E; 20 m a.s.l.; 20 Feb. 2020; A. Hunyadi leg.; coll. HA • 7 shells; Thanh Hoa Province, north of Thanh Hoa, Ham Rong Eny, Đong Tien Son; 19°51.108'N, 105°46.783'E; 35 m a.s.l.; 24 Nov. 2011; A. Hunyadi leg.; coll. HA • 60 shells; Hoa Binh Province, Tan Lac district, Quy Hau, 1300 m west on road no. 6, rock wall; 20.63222°N, 105.26955°E; 150 m a.s.l.; 11 Feb. 2020; A. Hunyadi, H.V. Luong, J.U. Otani & S.V. Pham leg.; coll. HA • 54 shells; Son La Province, 32.2 km northwest from centre of Son La towards Tuan Giao, Phong Lang, Tong Lanh, northeastern part of the village; 21°26.283'N, 103°43.965'E; 550 m a.s.l.; 07 Feb. 2020; A. Hunyadi, H.V. Luong, J.U. Otani & S.V. Pham leg.; coll. HA • 180 shells; Son La Province, Quynh Nhai district, 20 km north from junction of Thuan Chau, Chieng Khoang, cave above the village; 21°33.441'N, 103°40.909'E; 315 m a.s.l.; 07 Feb. 2020; A. Hunyadi, H.V. Luong, J.U. Otani & S.V. Pham leg.; coll. HA • 155 shells; Thanh Hoa Province, Quan Hoa district, Khu di tich Hang Ma, Song Luong gorge; 20°23.888'N, 105°4.016'E; 100 m a.s.l.; 12 Feb. 2020; A. Hunyadi, H.V. Luong, J.U. Otani & S.V. Pham leg.; coll. HA • 386 shells; Thanh Hoa Province, 3.6 km northwest from centre of Ngoc Lac, Lang Sat, 600 m north on the road no 15, under rock wall; 20°5.897'N, 105°21.578'E; 55 m a.s.l.; 13 Feb. 2020; A. Hunyadi, H.V. Luong, J.U. Otani & S.V. Pham leg.; coll. HA • 51 shells; Hoa Binh Province, Mai Chau, 550 m west of Cho Mai Chau, stream bank; 20.66029°N, 105.07804°E; 265 m a.s.l.; 11 Feb. 2020; A. Hunyadi, H.V. Luong, J.U. Otani & S.V. Pham leg.; coll. HA. **China** • 1 shell; Guangxi, Guigang Shi, Guzhang Xiang, Chuanshancun, road leading to north, limestone rock wall on the east; 23°20.865'N, 109°19.169'E; 150 m a.s.l.; 10 Sept. 2009; A. Hunyadi leg.; coll. HA • 17 shells; Guangxi, Chongzuo Shi, Longzhou Xian, Wude Xiang, Banxintun, vicinity of the junction; 22°35.239'N, 106°46.096'E; 350 m a.s.l.; 24 Sept. 2013; A. Hunyadi & M. Szekeres leg.; coll. HA • 2 shells; Yunnan, Yuxi shi, Chengjiang Xian, Luchong Ziran Fengjingqu, under Liushanting; 24°33.842'N, 102°50.614'E; 1778 m a.s.l.; 18 Mar. 2011; A. Hunyadi leg.; coll. HA • 2 shells; Guangxi, Guigang Shi, Guzhang Xiang, rocks above Chuanshancun; 23°21.056'N, 109°19.247'E; 200 m a.s.l.; 10 Sept. 2009; A. Hunyadi leg.; coll. HA • 9 shells; Guangxi, Laibin Shi, Wuxuan Xian, Wuxuan Tongling Zhen road, north of Yagangcun, rock wall; 23°29.827'N, 109°38.534'E; 21 Sept. 2013; A. Hunyadi & M. Szekeres leg.; coll. HA • 3 shells; Guangxi, Hechi Shi, Du an Yaozu Zizhixian, Gaoling Xiang, 2 km west from Dingfucun; 24°03.197'N, 108°01.290'E; 320 m a.s.l.; 10 Aug. 2009; A. Hunyadi leg.; coll. HA • 17 shells; Yunnan, Wenshan Zhuangzu Miaozu Zizhizhou, Guangnan Xian, Babao Zhen, southeastern edge of Babao; 23°44.682'N, 105°24.875'E; 1130 m; 24 Mar. 2011; A. Hunyadi leg.; coll. HA • 33 shells; Guangxi, Laibin Shi, Wuxuan Xian, Wuxuan Tongling Zhen road, west from Yagangcun, rocks; 23°29.151'N, 109°38.375'E; 80 m a.s.l.; 21 Sep 2013; A. Hunyadi & M. Szekeres leg.; coll. HA • 11 shells; Guangxi, Bose Shi, Leye Xian, southern edge of Molicun, left bank of Buliu He; 24°39.436'N, 106°43.245'E; 540 m a.s.l.; 08 Sep. 2013; A. Hunyadi & M. Szekeres leg.; coll. HA • 17 shells; Guangxi, Bose Shi, Lingyun Xian, Xiajia Xiang, southeastern edge of Xiajia, rock wall above the graves; 24°17.508'N, 106°38.376'E; 440 m a.s.l.; 22 Oct. 2009; A. Hunyadi leg.; coll. HA • 5 shells; village across from Yangchangshilin, Moyangzhen, Luodianxian, Guizhousheng; 25°32′39″N, 106°49′39.14″E; 908 m a.s.l.; T. Ishibe, K. Okubo, J.U. Otani leg.; coll. PGB • 4 shells; same data as previous; coll. PGB • 9 shells; Daxiaojingfengjingqu, Moyangzhen, Lodianxian, Guizhousheng; 25°33′39″N, 106°51′39″E; 450 m a.s.l.; T. Ishibe, K. Okubo, J. U. Otani leg.; coll. PGB • 8 shells; same data as previous; T. Ishibe, K. Okubo, J. U. Otani leg.; coll. PGB • 1 shell; Xianshanyuan, Shuangqiao-zhen, Wuming-xian, Guangxi Zhuanzu Zizhiou; 23°02.08015'N, 108°17.68551'E; 177 m a.s.l.; Ohai leg.; coll. PGB.

##### Type localities.

“Tonkin” (Montagne de ľÉléphant, Tonkin, Vietnam) (*H.crosseicrossei*); “Lang-son” (Tonkin, N Vietnam) (*H.crosseibrevituba*); “Lang-son” (Tonkin, N Vietnam) (*H.crosseiendodonta*); “collected from Tianxingqiao Town, Zhenning Bouyeizu Miaozu Zizhixian, Guizhou Province (26°N, 105°07'E), Guizhou Province”, China (*G.tianxingqiaoensis*); “collected from Nahui Town, Xingyi County (25°1'N, 104°8'E), Guizhou Province, China” (*G.xingyinensis*); “Limestone outcrop in Nawit Village, Viengxay District, Houaphane Province, Laos (20°22'37.3"N, 104°16'43.2"E), 695 m amsl” (*G.plesiolopa*).

**Figure 244.**
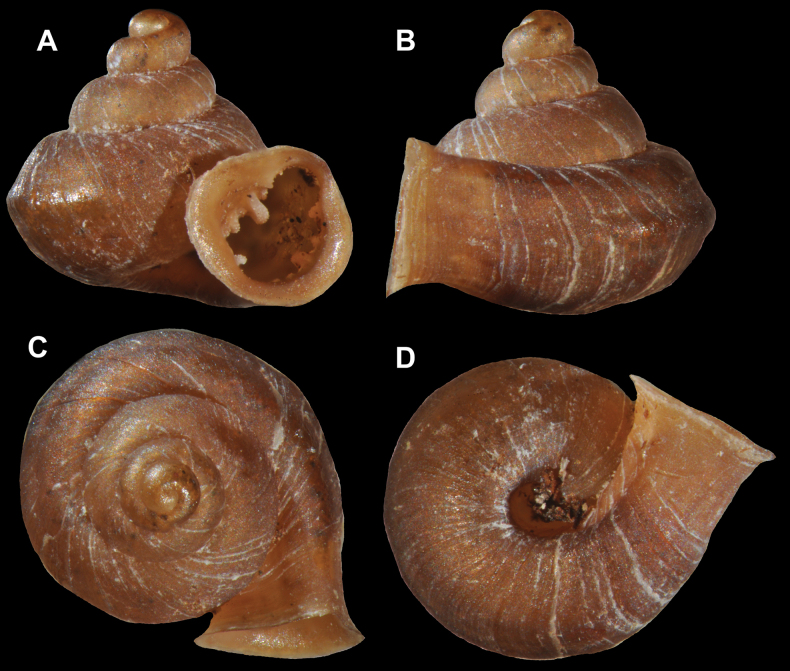
*Hypselostomacrossei*, syntype of *H.crosseicrossei* (MHNH-IM-2000-35155)

**Figure 245.**
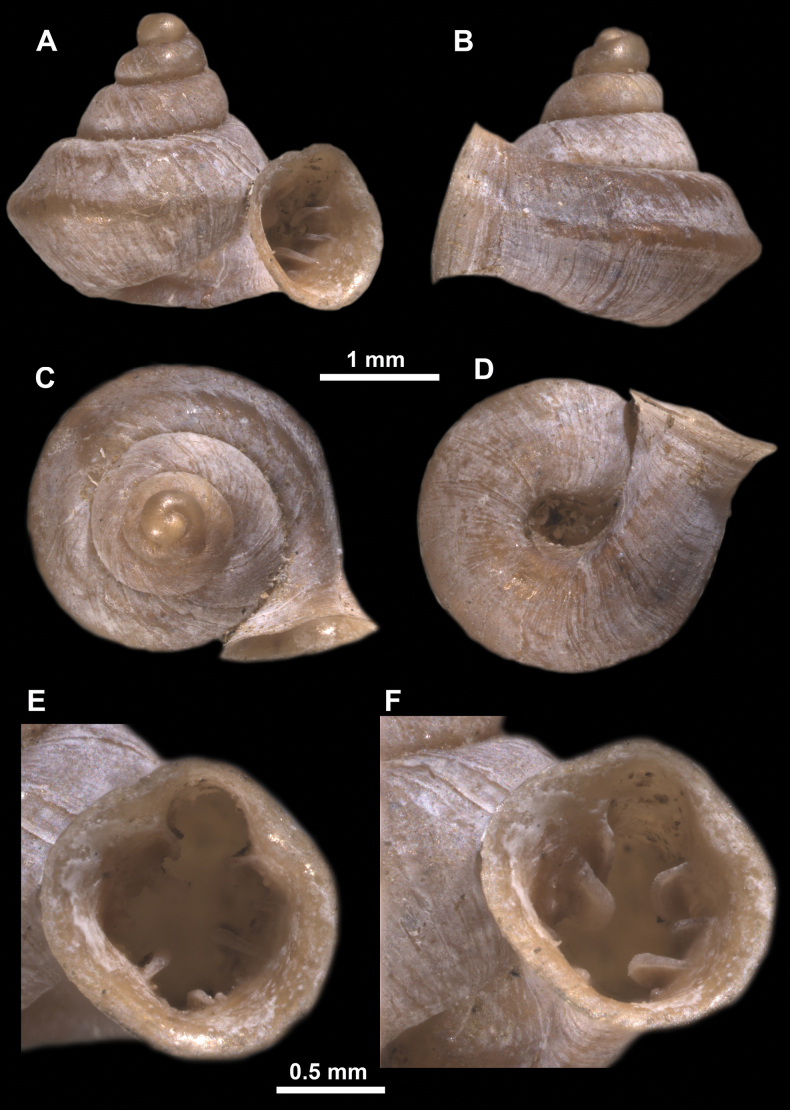
*Hypselostomacrossei*, lectotype of *H.crosseibrevituba* (SMF 4582) **A–D** shell **E, F** enlarged apertural views.

**Figure 246.**
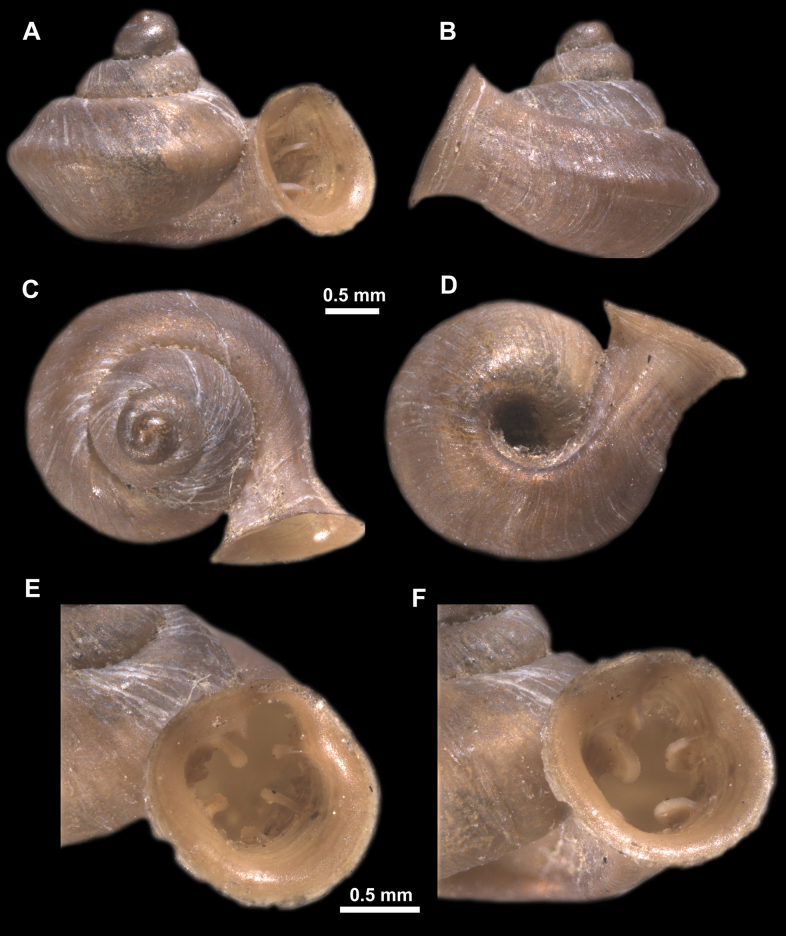
*Hypselostomacrossei*, lectotype of *H.crosseiendodonta* (SMF 4584) **A–D** shell **E, F** enlarged apertural views.

**Figure 247.**
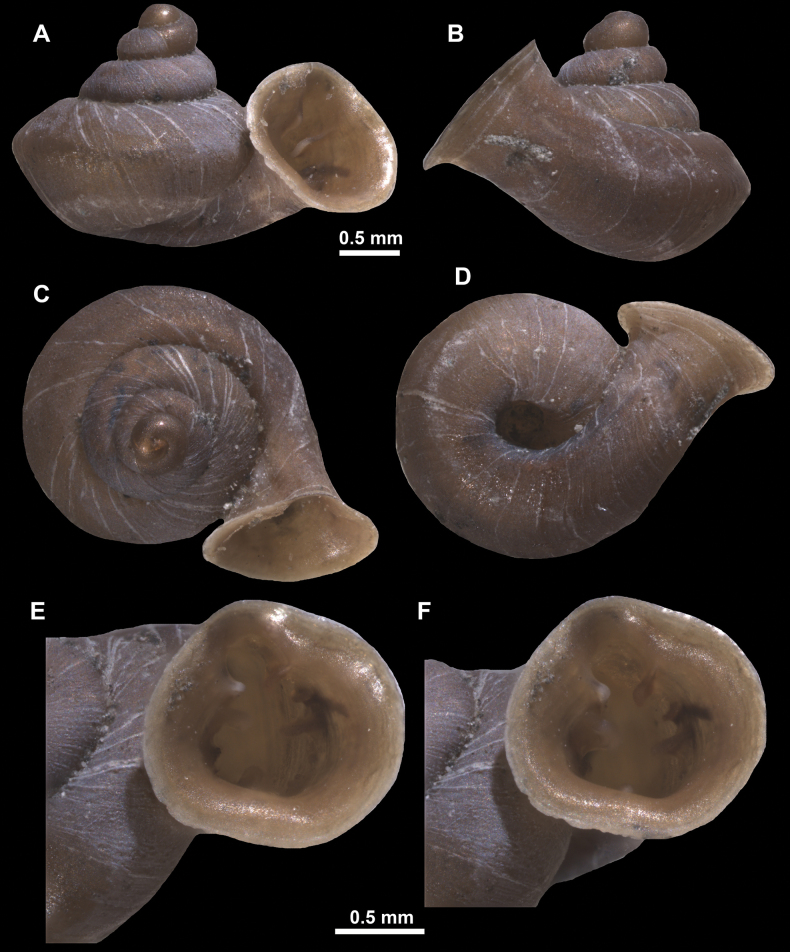
*Hypselostomacrossei*, paratype of *G.plesiolopa* (SMF 357824) **A–D** shell **E, F** enlarged apertural views.

##### Differential diagnosis.

This species differs from all its congeners by the combination of the dark brown shell, which is moderately umbilicate, numerous and variable apertural barriers, almost smooth shell surface (pasty), as well as the absence of the spiral striation.

**Figure 248.**
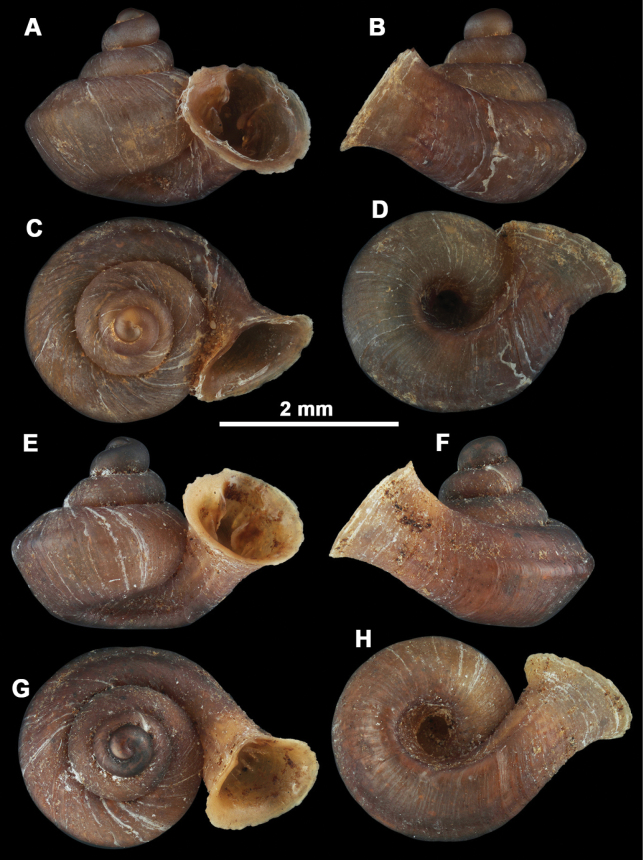
*Hypselostomacrossei***A–D** holotype of *B.tianxingqiaoensis* (IZCAS 025075 (from Gojšina et al. 2022)) **E–H** holotype of *B.xingyinensis* (IZCAS 069205) (photo. Z.-Y. Chen).

##### Distribution.

This species is known from a wide area covering China, N Vietnam, Myanmar, and Laos (from where it is known as *G.plesiolopa*).

##### Remarks.

The examination of type specimens of *H.crosseibrevituba* and *H.crosseiendodonta* and numerous additional samples of *H.crossei*, led us to the conclusion that this species is very variable regarding the appearance of the last whorl and also regarding barrier numbers, as was also observed by Pilsbry (1917). These were the characters used to describe the subspecies and there are no reasons to support their distinctness. *Gyliotrachelaplesiolopa*, *G.xingyinensis* and *G.tianxingqiaoensis* are junior synonyms of this species since no major morphological differences were observed. The latter two are originally described in exactly the same way, using exactly the same sentences, and are not compared mutually (or to *H.crossei*) but to other very distant species (Luo et al. 2000; Guo et al. 2006). Inkhavilay et al. (2016) pointed out that *G.plesiolopa* has fewer whorls (four) than *H.crossei* (five). However, this is not true due to the fact that we examined the types of the nominotypical subspecies of *H.crossei* and the other two described subspecies all of which showed between 3.5–4 whorls. According to them, *H.crossei* is also larger, but there is a clear continuum in size variation up to 1 mm. Therefore, we do not consider this character as relevant.

**Figure 249.**
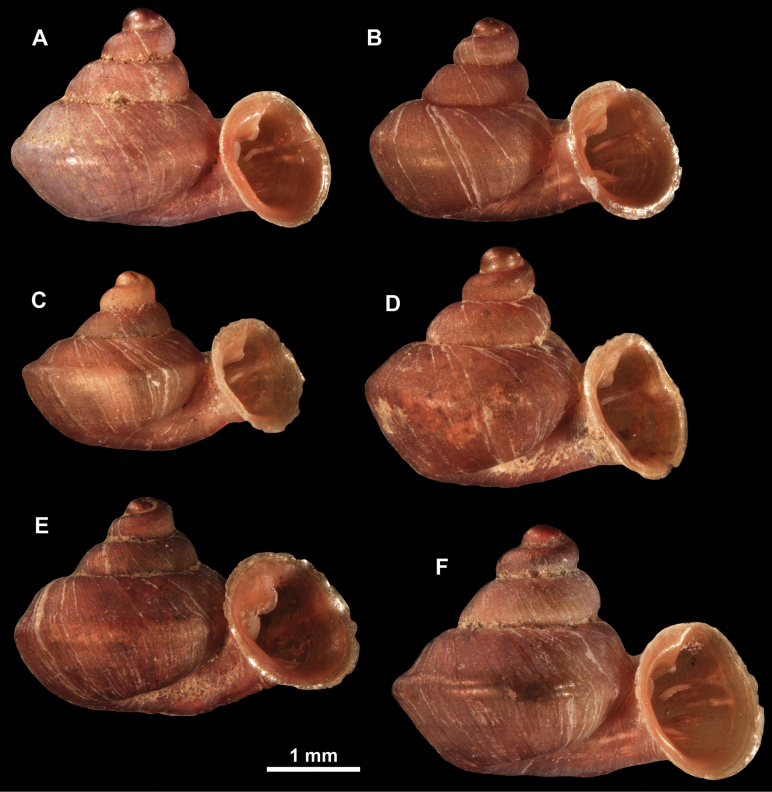
*Hypselostomacrossei* from different localities **A** China, Guangxi province (coll. HA) **B** China, Guizhou province (coll. PGB) **C** China, Yunnan province (coll. HA) **D** Vietnam, Cao Bang province (coll. HA) **E** Vietnam, Hoa Binh province (coll. HA) **F** Vietnam, Lang Son province (coll. HA).

**Figure 250.**
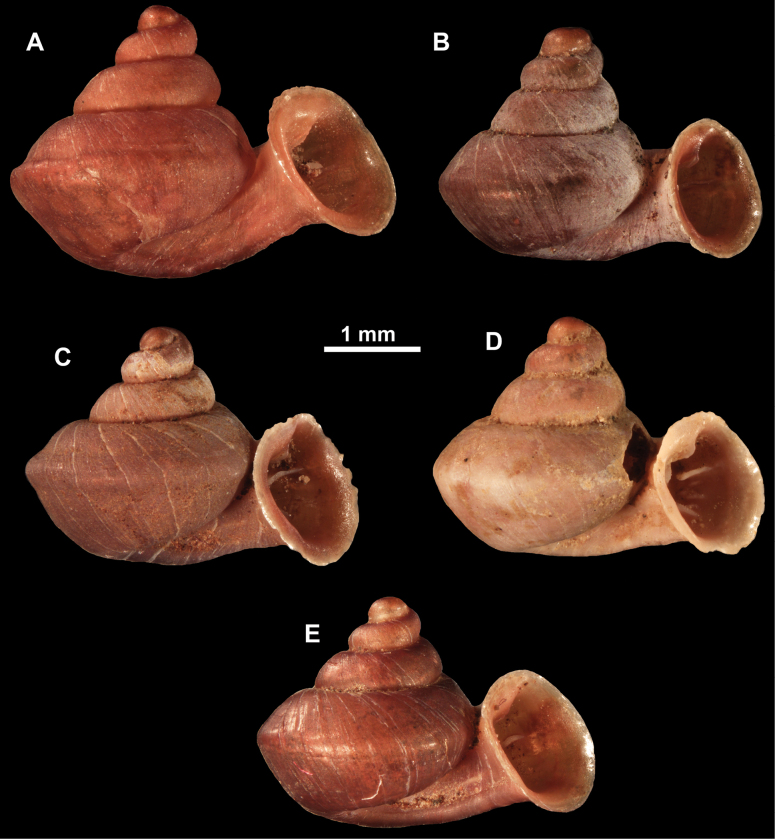
*Hypselostomacrossei* from different localities **A** Vietnam, Quang Ninh province (coll. PGB) **B** Vietnam, Ninh Binh province (coll. PGB) **C** Vietnam, Son La province (coll. HA) **D** Vietnam, Thai Nguyen province (coll. HA) **E** Vietnam, Thanh Hoa province (coll. HA).

**Figure 251.**
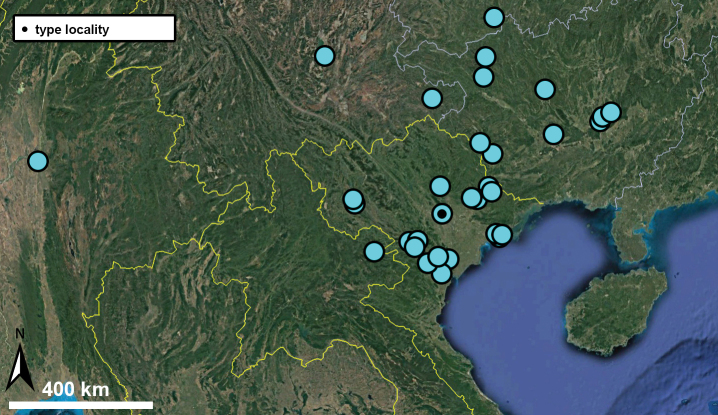
Distribution map of *H.crossei*.

#### ﻿ Hypselostoma depressispira

Taxon classificationAnimaliaStylommatophoraHypselostomatidae

(van Benthem Jutting, 1949) comb. nov.

1006C0BD-8D01-5D49-9CF0-FC8C8121A780

**Figure 252.**
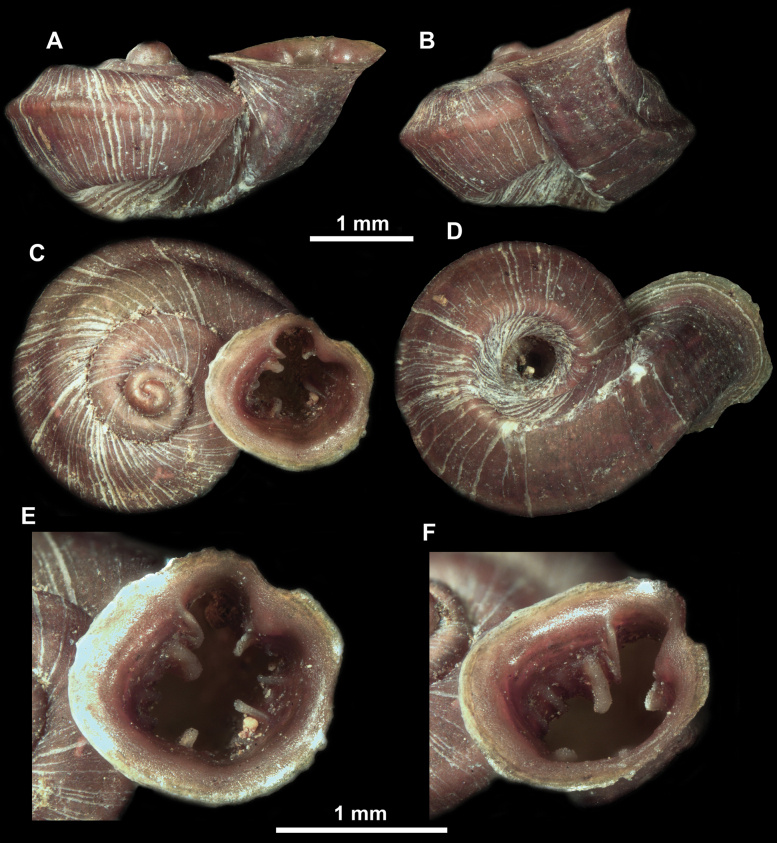
*Hypselostomadepressispira*, paratype (RMNH.Moll.137152) **A–D** shell **E, F** enlarged apertural views.

Figs 242C , 252 , 259

Gyliotrachela depressispira van Benthem Jutting, 1949b: 60, pl. 3.
Gyliotrachela depressispira — van Benthem Jutting 1950: 27, 45, fig. 15; Berry 1963: 361; Maassen 2001: 75.

##### Type material examined.

**Malaysia** • 12 paratypes; Pahang, Bukit Chintamani; ex. coll. Raffles Museum, Singapore; RMNH.Moll.137152.

##### Additional material examined.

**Malaysia** • 2 shells; Pahang, 25 km northwest from Kuantan, north of Kampung Panching, Gua Charas, rock temple; 03°54.692'N, 103°08.839'E; 140 m a.s.l.; 24 Jan. 2013; A. Hunyadi leg.; coll. HA • 28 shells; Kelantan, Gua Musang, vicinity of cave entrance; 04°52.974'N, 101°58.116'E; 135 m a.s.l.; 17 Jan. 2013; A. Hunyadi leg.; coll. HA • 37 shells; Kelantan, Gua Musang, 6 km towards Pulai, Gua Madu, vicinity of cave, 04°50.213'N, 101°56.982'E; 120 m a.s.l.; 17 Jan. 2013; A. Hunyadi leg.; coll. HA • 25 shells; Pahang, Gua Bama, Kuala Lipis 9 km, Padang Tungku; 04°11.652'N, 101°57.936'E; 120 m a.s.l.; 19 Jan. 2013; A. Hunyadi leg.; coll. HA • 201 shells; Pahang, Bukit Cinta Manis, south-southeastern side, Lebuhraya Karak 800 m towards Kampung Cinta Manis; 03°26.714'N, 102°00.814'E; 22 Jan. 2013; A. Hunyadi leg.; coll. HA • 3 shells; Pahang, Kota Gelanggi, Jengka-Jerantut road, on limestone surfaces covered with mosses; 03°53′28.55″N, 102°28′23.28″E; 29. May 2011; M. E. Marzuki leg.; coll. PGB • 4 shells; Pahang, Gua Bama at Kp. Relong (~ 14 km N of Kuala Lipis); Oct. 1998; Hemmen leg; coll. PGB • 6 shells; Pahang, Gua Bama (~ 12/13 km N of Kuala Lipis); 04°11.643'N, 101°57.925'E; Nov. 2000; Hemmen leg; coll. PGB.

##### Type locality.

“Bukit Chintamani, Pahang”, Malaysia.

##### Differential diagnosis.

This species bears some resemblance to the more depressed form of *H.fortunatum* sp. nov. However, *H.depressispira* has much more apertural barriers as well as separated angular and parietal lamellae. The last whorl is also more prominently keeled in *H.depressispira* and the umbilicus is slightly wider. See also under *H.tubiferum*.

##### Distribution.

This species is known from Peninsular Malaysia.

##### Remarks.

While examining the types, variability was observed in the smaller barriers. In some specimens, there were two and in others three interpalatal plicae. The number of lamellae between the columellar and parietal ranged between three and four. Number of barriers between lower palatal plica and columellar lamella ranged from two to three. Some specimens from Gua Musang (Kelantan) had only one barrier between the lower palatal and columellar and only one dot-like barrier between the columellar and parietal. Otherwise, the apertural barrier variability of other samples examined by us matches that of the type series.

#### ﻿ Hypselostoma muaklekense

Taxon classificationAnimaliaStylommatophoraHypselostomatidae

(Panha & J.B. Burch, 2002) comb. nov.

0E054234-EDE1-5B4F-921E-1FBBCFC7B938

**Figure 253.**
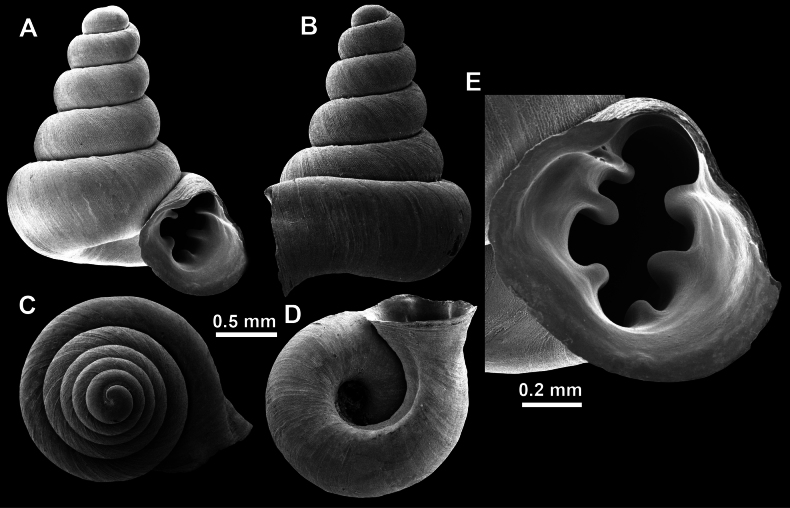
*Hypselostomamuaklekense*, holotype (CUMZ ver. 020) **A–D** shell **E** enlarged apertural view.

Figs 242D , 253 , 259

Paraboysidia muaklekensis Panha & Burch, 2002c: 79–81, fig. 2.
Gyliotrachela muaklekensis — Panha and Burch 2008: 75–76, fig. 65; Dumrongrojwattana and Wongkamhaeng 2024: 324, fig. 8.

##### Type material examined.

**Thailand** • holotype; 1997; S. Panha leg.; CUMZ ver. 020.

##### Type locality.

“Tepitak mountain, Muaklek District, Saraburi Province, 14°36'57"N, 101'15'50"E, 700 meters elevation” (Thailand).

##### Differential diagnosis.

This species is most similar to *H.modestum* from which it can be separated by the wider umbilicus, three strong apertural barriers on the palatal side (two in *H.modestum*) and smaller dimensions (SH = 2.9–3.1 and SW = 2.3–2.7 in *H.modestum* while SH = 1.8–2.0 and SW = 1.4–1.5 in *H.muaklekense*). *Hypselostomafrequens* also has only two strong barriers on the palatal side, is less slender and larger (SH = 2.8–3.5, SW = 2.4–2.6).

##### Distribution.

This species is known only from the type locality.

#### ﻿ Hypselostoma pattalungense

Taxon classificationAnimaliaStylommatophoraHypselostomatidae

Panha & J. B. Burch, 2004

43388661-50C6-56CF-8C52-39786E1CA04A

**Figure 254.**
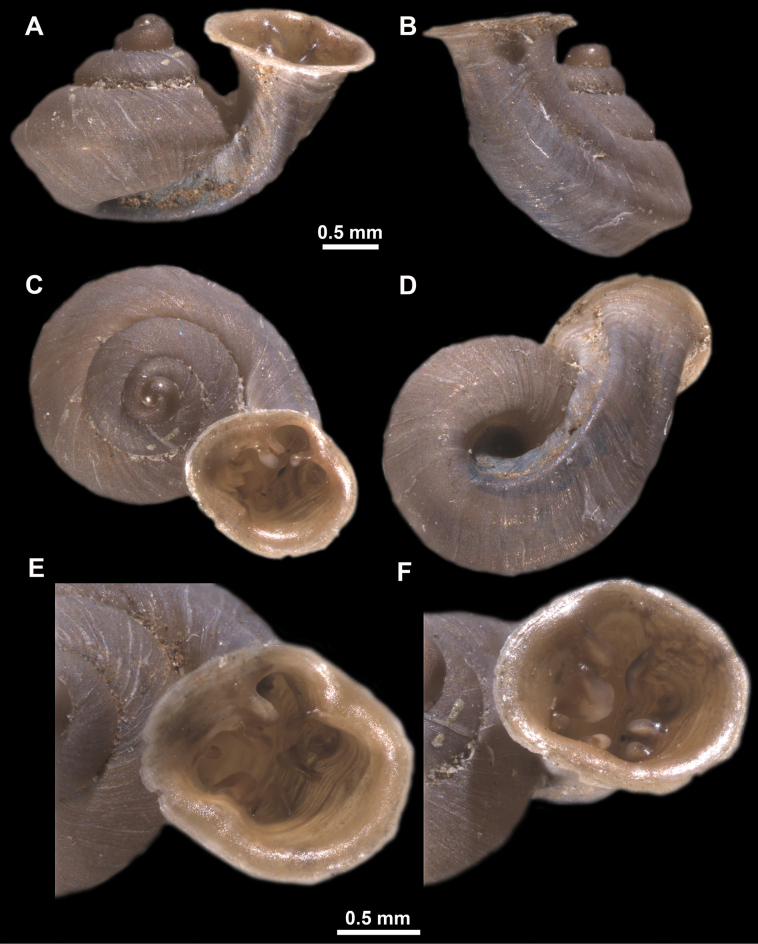
*Hypselostomapattalungense*, paratype (SMF 331456) **A–D** shell **E, F** enlarged apertural views.

Figs 242E , 254 , 259

Hypselostoma pattalungensis Panha & Burch in Panha et al. 2004: 73–75, fig. 11.
Hypselostoma pattalungensis — Panha and Burch 2008: 97–98, fig. 83; Dumrongrojwattana and Wongkamhaeng 2024: 324, fig. 8.

##### Type locality.

“Ko Si Ko Ha (Ko Na Thewada), Limestone Hill, Pattalung Province” (Thailand).

##### Type material examined.

**Thailand** • 3 paratypes; from the type locality; S. Panha leg.; SMF 331456.

##### Additional material examined.

**Thailand** • 3 shells; Phattalung Province, 6.4 km E of Hwy. 4081, 1 km SW of Khao Chai Son; 7°27'N, 100°11'E; 50 m a.s.l.; 11 Apr. 1988; K. Auffenberg leg.; locality code KA-0648; UF 345066 • 1 shell; same locality data as previous; UF 591338 • 13 shells; Songkhla Province, 31.3 km NW Hat Yai, 1.2 km W of Hwy. 43; 7°10'N, 100°16'E; 80 m a.s.l.; 08 Apr. 1988; K. Auffenberg leg.; locality code KA-0636; UF 344933 • 1 shell; same locality data as previous; 08 Apr. 1988; K. Auffenberg leg.; locality code KA-0635; UF 591337.

##### Differential diagnosis.

This species is most similar to *H.fortunatum* sp. nov., from which it differs by stronger and more numerous apertural barriers, separated angular and parietal lamellae as well as the higher spire.

##### Distribution.

This species is known from Phattalung and Songkhla provinces, Thailand.

##### Remarks.

Intraspecific variability is noticed on the appearance of the subcolumellar lamella, it can be present or completely absent.

#### ﻿ Hypselostoma pendulum

Taxon classificationAnimaliaStylommatophoraHypselostomatidae

(Panha & J. B Burch, 2002) comb. nov.

70BC59E3-8C9A-575A-9A6F-EC2897365365

**Figure 255.**
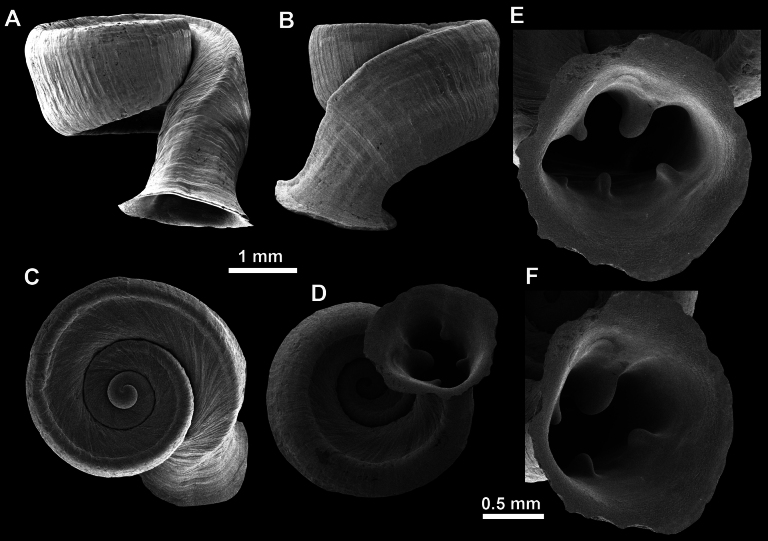
*Hypselostomapendulum*, paratype (CUMZ ver. 077) **A–D** shell **E, F** enlarged apertural views.

Figs 242F , 255 , 259

Antroapiculus pendulus Panha & Burch, 2002a: 144–148, figs 2, 3.
Anthroapiculus pendulus [sic] — Dumrongrojwattana and Wongkamhaeng 2024: 323, fig. 7.

##### Type material examined.

**Thailand** • 1 paratype; from the type locality; May 1998.; S. Panha, J. B. Burch, P. Dumrongrojwattana, C. Sutcharit, S. Tumpeesuwan, P. Tongkerd, W. Wanarat, S. Klomtong leg.; CUMZ ver. 077.

##### Type locality.

“Chonglom Mountain, Bhumiphol Dam reservoir, Samngao District, Tak province 17°14′51″N, 98°56′21″E” (Thailand).

##### Differential diagnosis.

This species is not similar to any other congener due to the completely flat but high and broad last whorl which is also strongly descending near the aperture (almost 90° angle). The parietal side of the aperture is with only a single, parietal lamella while the angular is absent. *Hypselostomasrakeoense*, *H.torticollis*, and *H.fungus* sp. nov. are superficially similar but none of them are flat and the latter two are also spirally striated. *Hypselostomatorticollis* additionally has separated parietal and angular lamellae on the parietal side.

##### Distribution.

This species is known only from the type locality.

#### ﻿ Hypselostoma phupaman

Taxon classificationAnimaliaStylommatophoraHypselostomatidae

(Panha & J. B. Burch, 2002) comb. nov.

A03D76F2-6579-50AB-BD54-A01D5609C6CF

**Figure 256.**
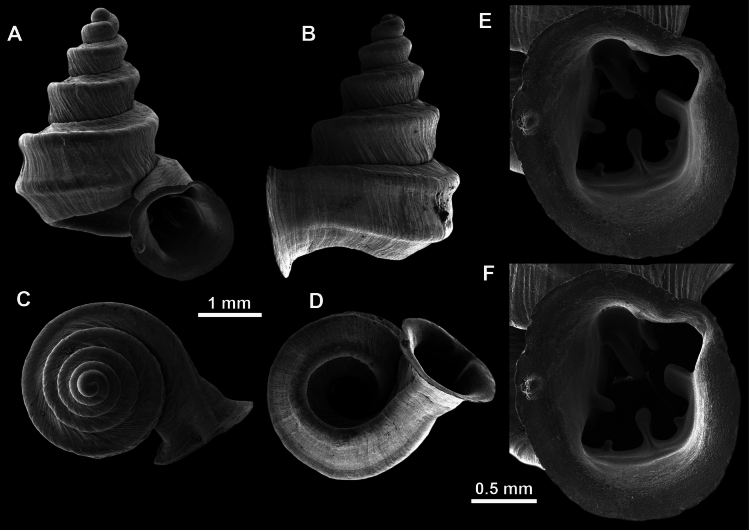
*Hypselostomaphupaman*, paratype (CUMZ ver. 086) **A–D** shell **E, F** enlarged apertural views.

Figs 242G , 256 , 259

Paraboysidia phupaman Panha & Burch, 2002c: 90, fig. 6.
Gyliotrachela phupaman — Panha and Burch 2008: 77–78, fig. 67; Dumrongrojwattana and Wongkamhaeng 2024: 324, fig. 8.

##### Type material examined.

**Thailand** • 1 paratype; from the type locality; 1998; S. Panha, P. Dumrongrojwattana, C. Sutcharit, S. Tumpeesuwan leg.; CUMZ ver. 086.

##### Type locality.

“Phupaman mountains, Petchaboon Province, Thailand, 16°39′52″N, 101°54′17″E, 60 meters elevation”.

##### Differential diagnosis.

This species is not similar to any of its congeners due to the unique, double-keeled last whorl.

##### Distribution.

This species is known only from the type locality.

#### ﻿ Hypselostoma similare

Taxon classificationAnimaliaStylommatophoraHypselostomatidae

Gojšina, Hunyadi & Páll-Gergely sp. nov.

7F69B785-C94B-5EDF-9CC9-05798722236A

https://zoobank.org/4BFB6B63-AFFA-451B-84FD-125A2979EB7B

**Figure 257.**
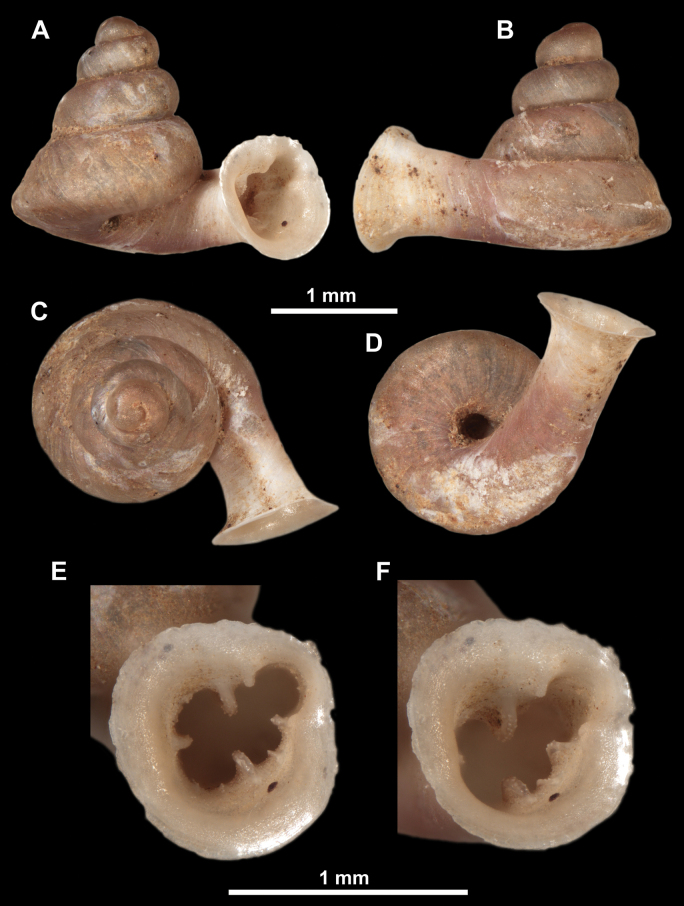
*Hypselostomasimilare* Gojšina, Hunyadi & Páll-Gergely, sp. nov., holotype (CUMZ 14470) **A–D** shell **E, F** enlarged apertural views.

**Figure 258.**
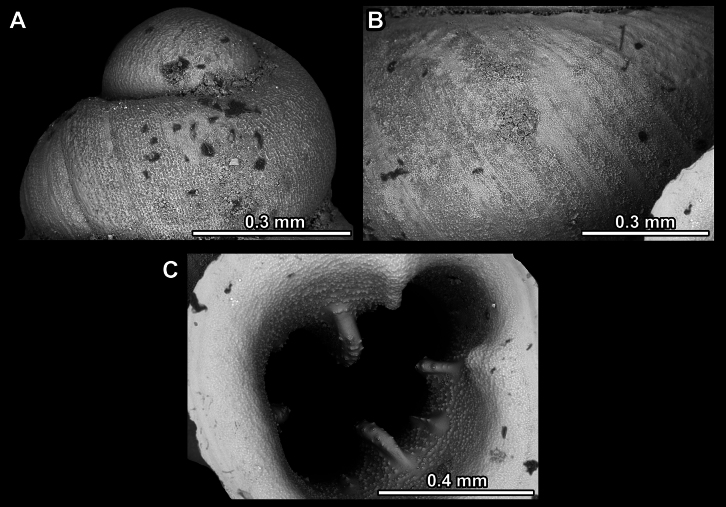
SEM imaging of *Hypselostomasimilare* Gojšina, Hunyadi & Páll-Gergely, sp. nov., holotype (CUMZ 14470) **A** protoconch surface **B** teleoconch surface **C** enlarged apertural view.

**Figure 259.**
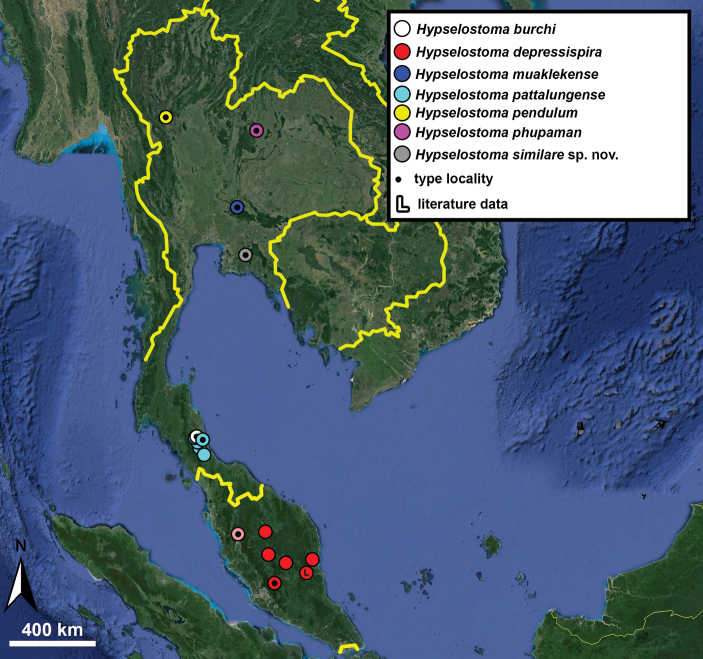
Distribution map of species belonging to *Hypselostomatubiferum* group.

Figs 242H , 257 , 258 , 259

##### Type material.

**Holotype. Thailand** • 1 shell (SH: 1.83 mm; SW1: 2.51 mm); Rayong Province, Khao Chamao district, Wat Tham Khao Prathun, gorge above the temple; 13°07.439'N, 101°35.850'E; 115 m a.s.l.; 09 Mar. 2023, A. Hunyadi leg.; CUMZ 14470. ***Paratypes*. Thailand** • 2 shells; same data as for holotype; coll. HA.

##### Type locality.

Thailand, Rayong Province, Khao Chamao district, Wat Tham Khao Prathun, gorge above the temple; 13°07.439'N, 101°35.850'E; 115 m a.s.l.

##### Diagnosis.

Shell triangular conical. Teleoconch almost smooth, finely radially but not spirally striated. Last whorl detached from the penultimate and ascending, keeled below the centre of the periphery. Aperture equipped with weak barriers, five main (angular, parietal, upper palatal, lower palatal and columellar) and several smaller ones. Umbilicus narrow, measuring 1/7–1/8 of the shell width.

##### Description.

Shell conical, light brownish, opaque, consisting of 3.75–4.25 weakly convex and regularly increasing whorls separated by a relatively shallow suture. Protoconch finely pitted, without spiral striae, consisting of ~ 1.5 whorls, same colour as the rest of the shell. Teleoconch sculpture delicate, very finely dimpled (pasty) and crossed by relatively weak radial growth lines. Spiral striae absent. Occasionally, a few stronger whitish radial streaks can be observed at the last whorl near the aperture. Last whorl weakly detached from the penultimate and slightly ascending near the aperture (~ 15–20 ° compared to the shell axis). It is bluntly but prominently keeled below the periphery, making the last whorl oblique to the shell axis and not convex but with a sloping, flat outline. There is a slight furrow above the keel. Peristome thick, whitish, expanded and not reflected. All apertural barriers are weak. Aperture equipped with five main barriers (angular, parietal, upper palatal, lower palatal, and columellar) and several smaller ones of variable number and appearance. There are usually no plicae in the sinulus, usually one plica in the interpalatal region, two or three plicae in the region of the basal plica and usually one or two lamellae in the columello-parietal region. Angular lamella tubercle-like, almost reaching the peristome. Behind it, more deeply situated, there is one additional part which also belongs to the angular lamella. Parietal lamella stronger and higher when compared to the others (except lower palatal plica), not reaching the profile of the angular tubercle. Lower palatal plica developed to the same extent as the parietal lamella and slightly higher and stronger than the upper palatal plica. Columellar lamella horizontal, developed equally as the upper palatal plica. Anterior to the upper palatal plica, there is one more tubercle-like part, possibly homologous with the palatal tubercle found in the genus *Bensonella* (see Páll-Gergely and White 2023) or it even might represent a part of the upper palatal plica. Weaker barriers are all positioned deeper in the aperture and never reaching the profiles of the main ones. A tubercle-like swelling is present in the columellar-parietal transition embayment. All barriers are strongly and coarsely spiniferous. Sinulus small, rounded and distinctly separated from the rest of the aperture. Umbilicus narrow, with almost perpendicular walls, showing only a part of the penultimate whorl and measuring ~ 1/7–1/8 of the shell width.

##### Differential diagnosis.

By its last whorl keeled at its base, this species in superficially most similar to *H.insularum* and *Hypselostoma* species inhabiting the Philippines. However, this species most clearly differs from these representatives by the presence of two lamellae on the parietal side (angular and parietal) and more numerous barriers in the aperture including smaller ones. *Hypselostomainsularum* further has strong spiral striae which are absent in the new species.

##### Etymology.

Named after the fact that this species is in shell shape very similar to some *Hypselostoma* species inhabiting the Philippines.

##### Measurements

**(in mm, *n* = 3).**SH = 1.82–1.91; SW1 = 2.34–2.51; SW2 = 1.45–1.51; AH = 0.98–1.01; AW = 0.9–0.98.

##### Distribution.

This species is known only from the type locality.

##### Remarks.

All barriers in the aperture very weak, smaller ones are hardly observable, only in form of very weak lines. Single interpalatal plica present or absent. Sinulus plicae present or absent.

#### ﻿ Hypselostoma tubiferum

Taxon classificationAnimaliaStylommatophoraHypselostomatidae

(Benson, 1856)

F475DBCA-4004-5DAD-A0BA-4AF63CEA4EF7

**Figure 260.**
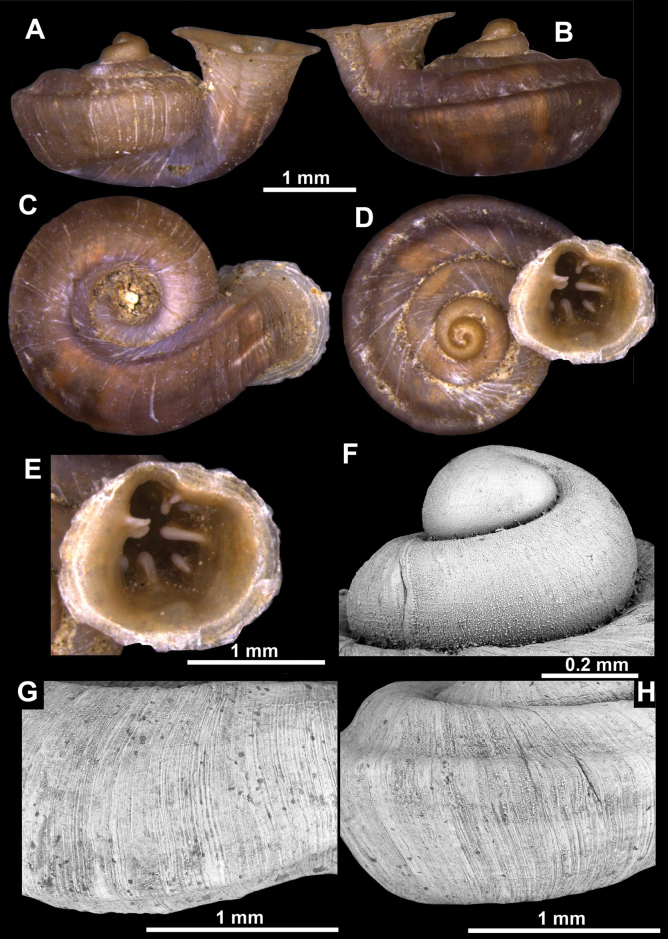
*Hypselostomatubiferum* (NHMUK 1888.12.4.17–22) **A–D** shell **E** enlarged apertural view **F** enlarged view of the protoconch **G, H** enlarged view of the surface of the last whorl (from Gojšina et al. 2022).

**Figure 261.**
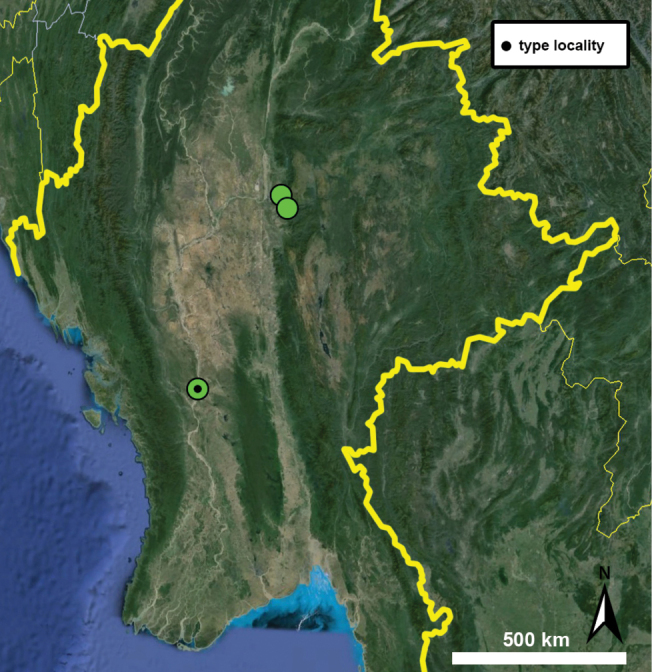
Distribution map of *Hypselostomatubiferum*.

Figs 242I , 260 , 261

Tanystoma tubiferum Benson, 1856a: 130.
Hypselostoma tubiferum — Pfeiffer 1859: 325; Blanford 1863: 326; Hanley and Theobald 1870: 4, pl. 8 fig. 3; Stoliczka 1871: 173, pl. 7 fig. 1; Pfeiffer 1876 in 1875–1876: 488; Möllendorff 1891: 338; Gude 1914: 298; Pilsbry 1917: 178–179, pl. 31 figs 1–5; van Benthem Jutting 1950: 43; Zilch 1984: 163; Gojšina et al. 2022: 137, fig. 7; Tongkerd et al. 2024: 188, fig. 13Q.
Pupa (Hypselostoma) tubifera — Nevill 1878: 193.

##### Material examined.

**Myanmar** • 6 shells; Tondung/Thyet Mio; W. Theobald leg.; NHMUK 1888.12. 4.17–22 • 35 shells; Mandalay, Patheingyi, near Shin Bodh Mell Waterfall; 21°59.281'N, 96°12.909'E; 225 m a.s.l.; 16 Oct. 2018; A. Hunyadi leg.; coll. HA • 31 shells; Mandalay, Kyaukse, Kho Nan Shin Cave Pagoda; 21°44.153'N, 96°20.229'E; 110 m a.s.l.; 17 Oct. 2018; A. Hunyadi leg.; coll. HA • 45 shells; Mandalay, Dee Dote, Waterfall; 21°42.564'N, 96°21.282'E; 185 m a.s.l.; 17 Oct. 2018; A. Hunyadi leg.; coll. HA • 3 shells; "India" (probably referring to former British India); F. F. Linter coll.; UF 00112342 • 1 shell; India (probably referring to former British India), Tankarin; 01. Jun. 1998– 30. Jun. 1998; UF 540729.

##### Type locality.

“Thyet-Mio prope ripas fluminis Irawadi Burmanici, saxis calcareis adhaerens” (nowadays Thayet), Myanmar.

##### Differential diagnosis.

This species is superficially similar to *H.depressispira* from which it can be separated by the concrescent angulo-parietal lamella (separated in *H.depressispira*) and fewer barriers in the aperture which are developed to roughly the same extent.

##### Distribution.

This species is, apart from the type locality, known from several localities in Mandalay, Myanmar.

##### Remarks.

Colouration of the animal body was observed and described by Stoliczka (1871). It was described as pale grey with blackish tentacles. Foot is short and ovate-elongated. The striking similarity of this species with *H.depressispira* suggests that *Gyliotrachela* is a junior synonym of *Hypselostoma*.

#### ﻿ Hypselostoma

Taxon classificationAnimaliaStylommatophoraHypselostomatidae

sp. 1

71181B30-F41C-5C2A-AC2A-D8A7A05DAA17

##### Material examined.

**Thailand** • 2 shells; Trang Province, Kantang district, Yong Ling beach; 4 July 2014; J.U. Otani leg.; coll. PGB. **Malaysia** • 344 shells; Kelantan Province, 19 km south of Gua Musang; Oct. 1999; Hemmen leg.; coll. PGB.

##### Remarks.

Two samples from Thailand and Malaysia represent two different species, both of them probably most similar to *H.burchi*. Two specimens from Thailand are slightly more slender, lack interpalatal plica and have slightly narrower umbilicus. Specimens from Malaysia have slightly more detached and descending last whorl and they lack the interpalatal plica as well. Due to the absence of proper comparison material of *H.burchi*, we do not describe these taxa in this work.

**Figure 262.**
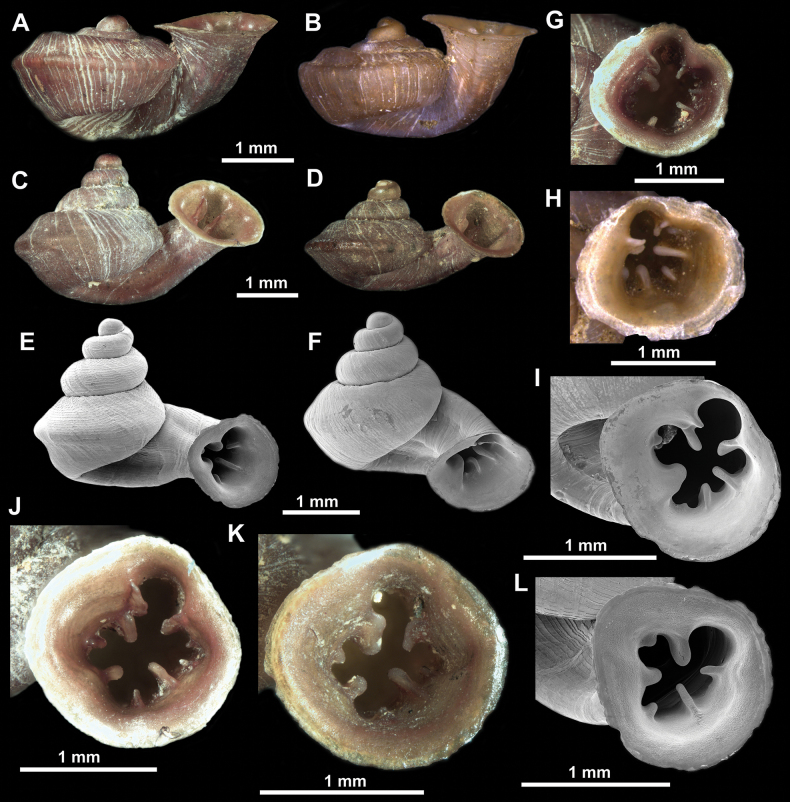
Comparison of superficially very similar species which were formerly placed in different genera **A, G***H.depressispira* (formerly in *Gyliotrachela*) **B, H***H.tubiferum***C, J***H.luctans* (formerly in *Gyliotrachela*) **D, K***H.piconis***E, L***H.taehwani***F, I***H.burchi* (formerly in *Gyliotrachela*).

#### ﻿ Hypselostoma

Taxon classificationAnimaliaStylommatophoraHypselostomatidae

sp. 2

6392CEF4-3226-5ADC-95BA-D9B10781109B

##### Material examined.

**Thailand** • 1 shell; Suratthani Province, 4.5 km S Don Sak, E side of Hwy. 4142, evergreen forest, limestone outcrop, base of cliff; 9°18'N, 99°42'E; 18 Apr. 1988; K. Auffenberg leg.; locality code KA-0674; UF 345307.

##### Remarks.

A single fragment, identification impossible.

## ﻿﻿Discussion

### ﻿Comments on some characters used for the distinction of hypselostomatid genera

As aforementioned, characters used for traditional distinction of genera are: i) the level of last whorl detachment (detached or adnate) and ii) the appearance of the lamellae on the parietal side (parietal and angular lamellae merged or separated, angular lamella absent). We note that the shell size can also be important for generic placement.

#### ﻿﻿Appearance of the last whorl

According to Pilsbry (1917), the free last whorl is present in *Hypselostoma* and *Gyliotrachela*, a character used for their distinction from *Boysidia*, *Paraboysidia*, and *Anauchen*. Subsequent descriptions of species with a detached last whorl in e.g., *Anauchen*, has proven that the appearance of the last whorl is no longer considered significant in the separation of these genera (Vermeulen et al. 2019). We have noticed that this trait can be variable to some extent within the same population (or different populations) of one species. Examples of this can be found in some widespread and variable species such as *H.crossei*, which led to descriptions of several subspecific taxa (e.g., *H.crosseibrevituba* which is characterised by a short, almost adnate, trumpet, see Möllendorff 1901b). These subspecies are all herein treated as junior synonyms of the nominotypical subspecies because they completely fall within the intraspecific variability and a clear continuum of character states has been observed.

Another example of a different last whorl appearance within the same population, is found in the type series of *H.cambodjense*. In these samples, we found a specimen with a completely adnate last whorl which is also straight (in contrast to other specimens in which the last whorl is detached and slightly turned upwards).

Because of this, last whorl detachment is not a reliable character for generic distinction, and is subjected to a certain amount of intraspecific variability. This variability is not large since we have not found a population of the same species with different last whorl appearance (such as ascending and descending within one species). It is clear that the level of detachment of the last whorl is a reliable character for species-level identification if there are no overlapping examples between populations of two species.

#### ﻿﻿Appearance of the apertural barriers

Since the last whorl is now considered insignificant on the generic level, the only character used for genus distinction is the appearance of the apertural barriers. According to Pilsbry (1917), *Gyliotrachela* and *Paraboysidia* had separate angular and parietal lamellae, *Boysidia* and *Hypselostoma* had a bifid angulo-parietal lamella and *Anauchen* had a parietal lamella only. We have found several populations of hypselostomatids with a typical *Anauchen* shape (e.g., *A.angthongensis*, *A.obesus* sp. nov.) but with a bifid angulo-parietal lamella, which is sometimes also represented as a single (parietal) lamella. Furthermore, examining the literature and type material, we have found some species which are very similar in shell shape, but have a single parietal lamella (*A.huaykhakang*) or bifid angulo-parietal lamella (*A.utaithaniensis*).

As for the genus *Gyliotrachela*, the only feature that distinguishes it from *Hypselostoma* is separate angular and parietal lamellae. However, it has been found that there are several cases where two species belonging to these two genera also have a superficially identical shell morphology (Fig. 262). Examples of such species are *Hypselostomatubiferum* and *Hypselostomadepressispira* as well as *H.piconis* and *H.luctans*. Even in some cases where the shell of *H.utongense* is completely flat, it looks superficially very similar to *H.tridentatum* or *H.khaowongkot*. Furthermore, *H.chedi* and *H.smokon* are superficially very similar to *H.khaochakan* and geographically they are also quite approximate, so it is very unlikely that they belong to two different genera. This also applies to *H.taehwani* and *H.burchi*. We have not yet come across a population of *Gyliotrachela*/*Hypselostoma* with specimens with both concrescent and separate lamellae on the parietal side, but in “*Anauchen*” this was the case. It seems that the appearance of the lamellae on the parietal side alone does not justify the separation of the two genera, which is why *Gyliotrachela* is treated here as a junior synonym of *Hypselostoma*.

Apertural barriers, however, are not of zero importance on the generic level. Typical *Bensonella* species (grouped around the type species, *B.plicidens*) largely share a consistent barrier arrangement (with some exceptions, e.g., *B.pahpetensis*). This is manifested in the form of three lamellae on the parietal side (infraparietal, parietal, and angular) and an additional strong palatal tubercle sitting on the palatal lip. This means that the apertural barriers, at the generic level, should be used in combination with other characters (shell size, shape, and surface sculpture) for a proper assignment to the genus.

#### ﻿﻿Shell size

Size of the shell can also be important on the genus-level, but even here, the situation is far from clear. Genera not treated herein (such as *Acinolaemus*, *Angustopila*, *Clostophis*, *Tonkinospira*) are by far much smaller in size (including the world’s smallest land snail species, see Páll-Gergely et al. 2022). In *Anauchen*, some species are provisionally placed based on a single parietal lamella and adnate last whorl, even though they are by far much smaller than the type species of the genus (Gojšina et al. 2023; Páll-Gergely 2023a). These species almost certainly do not belong to *Anauchen* and could even be considered genera of their own. However, given the confusion at the genus level, erecting new genera for these species would be premature and this would not be based on stable morphological characters. This means that the shell size could be more important in the future as we gather more morphological and phylogenetic evidence.

### ﻿Homology of apertural barriers and their “hooked“ appearance

After examining numerous specimens, we were able to draw several important conclusions regarding the homology of apertural barriers. There is a continuum of the appearance of the barriers on the parietal side among different genera. In the former *Gyliotrachela*, angular and parietal lamellae are separate, but this is different from species to species. For instance, in *H.khmerianum* (originally described under *Gyliotrachela*) the angular and parietal lamellae are quite distant from each other but less so in some other species (including the type species, *H.hungerfordianum*). *Hypselostomakhaowongkot* has its angular and parietal lamella very closely positioned but still separate (even though it was originally described under *Hypselostoma*). In *Hypselostoma*, the angular portion of the concrescent angulo-parietal lamella can be more or less pronounced (pointed) or be almost absent. Finally, in many *Anauchen* species, there is only one clearly visible lamella (parietal). This makes it clear that there is a continuum of parietal barrier appearance between these three genera. The angular lamella of former *Gyliotrachela* is clearly homologous to the angular part of the angulo-parietal lamella in *Hypselostoma*, while the parietal lamella is homologous to the larger (parietal) portion of the angulo-parietal lamella. The single ”parietal” lamella in *Anauchen* is probably only a completely fused angulo-parietal and is thus homologous to both these lamellae. This means that the angular lamella is not absent in *Anauchen*, as traditionally interpreted, but is completely fused so that the boundary is not discernible.

Regarding the palatal plicae, the palatal tubercle in *Bensonella* is most probably homologous to the small swelling which can occasionally be seen in several *Hypselostoma* and *Anauchen* species (Gojšina et al. 2023). Very rarely, there is a unique barrier on the palatal side which is positioned perpendicularly to the shell axis (e.g., in *B.nordsiecki*, *B.obex* sp. nov., *B.dracula* sp. nov.). We propose that this barrier should be called “transversal palatal plica“. It seems that the homology of the transversal palatal plica is clear. This plica originated by fusion of the interpalatal and lower palatal plica, while the outer portions remained unfused in some cases. This is strongly supported by the fact that in e.g., *B.taiyaiorum*, two line-like extensions can be seen extending from the transversal palatal plica toward the peristome edge. The one closer to the separated upper palatal plica is actually an interpalatal, while the one below it is the lower palatal. In some species/specimens, it seemed that the fusion only partly occurred so that two distinct parts are clearly visible (in *B.lophiodera*). This transversal plica surely provides more efficient defence against predators since it resembles the appearance of the “barricade”. In *Bensonella*, frequently there are multiple palatal or basal plicae of the same appearance (height, width, length) which makes the naming and the homology of them more complicated. It is not always clear whether several barriers in the basal region represent multiple basal plicae or some are infrapalatal or subcolumellar. We tried to standardize the nomenclature of these barriers as follows: the upper palatal plica is located behind the palatal tubercle or slightly above it at the beginning of the sinulus; the interpalatal plica is located behind and slightly below the palatal tubercle, while lower palatal plica is located halfway between the palatal tubercle and basal plica. However, when several equally strong palatal plicae exist, it might not be possible to unambiguously separate interpalatals from the lower palatal or even infrapalatal. In these cases, homology is unclear (Fig. 9).

Hooked apertural barriers are not uncommon in hypselostomatids (altogether 17 species of described within the genera in scope have at least one barrier hooked). We have noticed that the barriers on the parietal and columellar sides are relatively rarely hooked (e.g., in *B.hooki* and *B.lakainguta*), while palatal plicae are always hooked (in species with hooked barriers). It is known that these hooks (together with the protruding, detached last whorl) contribute to the defence of the specimen against different predators. If hooks and the protruding last whorl were not removed, the survival rate was very high (94%). Removal of these hooks led to higher vulnerability and higher death rate (survival rate was 20% lower than with hooks, see Cameron 2016). If the protruded last whorl is removed together with the hooks, death rate was very high (survival rate was 43% lower than in the control group) (Cameron 2016 and references therein). Within a single population of species with normally hooked barriers, hooks may sometimes be missing. This is found in *H.sorormajor* sp. nov. where a basal plica can be blunt or distinctly hooked. In general, species with hooked barriers were always regarded as distinct from the one without hooks.

### ﻿Further notes

We have found some species that are geographically very distant from each other but morphologically almost identical, such as *Bensonellakitteli* and *B.hupeana*, which have been described from type localities more than 3000 km apart. A similar example is found in *B.boettgeri* which was described from Java (Indonesia). This species shares the identical shell traits as *B.novemdentata* from Laos even though their type localities are located ~ 2900 km away from each other. One more identical specimen from India is herein examined by us and treated conspecific with *B.boettgeri*. We do not know whether this is a result of insufficient exploration, parallel evolution, or a long-distance dispersal but these species are herein all treated conspecific since not a single difference in shell morphology could be observed. A similar case is present and explained in Páll-Gergely et al. (2015) for *Angustopila*.

There are also several species with continuous, broad distributions. *Hypselostomakhaowongense* is reported from many localities in Thailand and also Myanmar and Laos. *Hypselostomahungerfordianum* is apparently widespread in southern Thailand and Malaysia. *Hypselostomacrossei* is distributed in a belt extending from Myanmar through northern Laos and Vietnam (possibly also in Thailand). It also occurs in China, where it is known under two synonyms (*H.tianxingqiaoensis* and *H.xingyinensis*). This makes it clear that not all species are stenoendemic and that special attention should be paid to the widespread species. It should also be noted that these widespread species show considerable intraspecific variation. *Hypselostomacrossei* is so variable with respect to the arrangement of the apertural barriers that they no longer have taxonomic significance. This species is also variable in the level of the last whorl protrusion and even shell surface to some extent. In the same population of *H.crossei*, we have found specimens with no spiral striation as well as specimens with very faint spiral striation. Another widespread species, *H.hungerfordianum*, is also quite variable in some conchological characters such as the level of the last whorl detachment, the number of smaller apertural barriers, and sometimes also the width of the umbilicus.

*Hypselostomakhaowongense* is a widely spread species which shows variability similar to the one found in *H.crossei*. In *H.khaowongense*, there is a highly variable number of smaller barriers between the larger ones, the last whorl can be rounded or slightly keeled, the umbilicus can vary in width (although always wide) and even spiral surface sculpture can be more or less pronounced.

We can also separate a group of species which share the same, unique shell microsculpture in the form of very fine granules (herein classified under *H.hungerfordianum* group). It is very unlikely that this type of unique microsculpture has independently evolved several times in this group, so we can consider that all these species are probably more closely related.

Some species clearly show enormous variability in the number and arrangement of apertural barriers, while others are less or far less variable. A smaller number of species possess hooked (claw-like) barriers, which has already been described as a sign of rapid adaptive evolution (Wada and Chiba 2013) that is not due to close phylogenetic relationships.

*Antroapiculus* was described as a monotypic genus due to the unique combination of shell features, such as the very high but flat last whorl, the strongly sloping last whorl and the parietal side equipped with a single lamella. There are also species such as *Hypselostomasrakeoense* (described as *Anauchensrakeoensis*) and *H.torticollis* (described as *Gyliotrachelatorticollis*) and *H.fungus* sp. nov. that have the same degree of downward rotation of the aperture, and *H.srakeoense* even has a single lamella on the parietal side, just like *Antroapiculus*. *Antroapiculuspendulus* also has a very high last whorl and a wide trumpet, which gives it a unique appearance. This feature is also shared with *H.chaunosalpinx*, described from Cambodia, further supporting the sharing of features (overlap), and justifying the synonymy with *Hypselostoma*.

Based on molecular data, Tongkerd et al. (2004) have shown that species with strikingly different shells, but with the same surface sculpture, can be closely related (as in the case of *Hypselostomaerawan* and *H.panhai*). This can be used as a basis for further taxonomic studies which are needed urgently to solve the current confusion at the genus level. It was impossible to propose a new and appropriate system of placing species into genera since hypselostomatids are a very diverse group with numerous transitional forms in terms of shell size, shape, and appearance of the apertural barriers. We believe that a thorough phylogenetic work, in combination with morphological and anatomical characters, can solve this more than a century-long mystery.

## ﻿﻿Additional information

### Author ORCIDs

Vukašin Gojšina https://orcid.org/0000-0002-0413-9304

András Hunyadi https://orcid.org/0000-0003-3488-2817

Chirasak Sutcharit https://orcid.org/0000-0001-7670-9540

Piyoros Tongkerd https://orcid.org/0000-0001-9221-9293

Kurt Auffenberg https://orcid.org/0000-0001-9660-6776

Jozef Grego https://orcid.org/0000-0002-4977-0415

Jaap J. Vermeulen https://orcid.org/0000-0002-8505-0319

Alexander Reischütz https://orcid.org/0000-0002-8844-7004

Barna Páll-Gergely https://orcid.org/0000-0002-6167-7221

### Data availability

All of the data that support the findings of this study are available in the main text.

## References

[B1] Adams H Adams A (1854–1858) The genera of recent Mollusca; arranged according to their organization. John Van Voorst, London. Vol. 2: 1–92 [pls 61–72 (1854); 93–284, pls 73–96 (1855); 285–412, pls 97–112 (1856); 413–540, pls 113–128 (1857); 541–660, i–xl, pls 129–138 (1858)].

[B2] Albers JC von Martens E (1860) Die Heliceen nach natürlicher Verwandtschaft systematisch geordnet. Manuskript besorgt von Eduard von Martens. Wilhelm Engelmann, Leizig [i–xviii] 1–359. 10.5962/bhl.title.11218

[B3] Ancey CF (1881) Description de mollusques terrestres nouveaux. Le Naturaliste 3(47): 373–374.

[B4] Bavay A Dautzenberg P (1904) [“1903”] Description de coquilles nouvelles de l’Indo-Chine. (3^e^ suite). Journal de Conchyliologie 51(3): 201–236 [pls 7–11].

[B5] Bavay A Dautzenberg P (1909) [“1908”] Molluscorum terrestrium Tonkinorum diagnoses. Journal de Conchyliologie 56(4): 229–251.

[B6] Bavay A Dautzenberg P (1912) Description de coquilles nouvelles de l’Indo-Chine (7^e^ suite). Journal de Conchyliologie 60(1): 1–54 [pls 1–6].

[B7] Benson WH (1849) Descriptions of four new Asiatic species of the genus *Pupa* of Draparnaud. Annals and Magazine of Natural History, Series 2, 4(20): 125–128. 10.1080/03745486009496158

[B8] Benson WH (1856a) Description of *Tanystomatubiferum*, a Burmese form related to the genus Anostoma of Lamarck. Annals and Magazine of Natural History, Series 2, 17(98): 129–131. 10.1080/00222935608697483

[B9] Benson WH (1856b) Remarks on the genera *Tanystoma*, *Nematura*, and *Anaulus*. Annals and Magazine of Natural History, Series 2, 17(100): 342–343. 10.1080/00222935608697520

[B10] Benson WH (1860) On *Clostophis* and *Rhiostoma*, new Burmese genera of land-shells. Annals and Magazine of Natural History, Series 3, 5(26): 95–97. 10.1080/00222936008697183

[B11] Berry AJ (1963) The genital system of the Malayan limestone hill snail *Gyliotracheladepressispira*, with notes on breeding. Proceedings of the Zoological Society of London 141(2): 361–369. 10.1111/j.1469-7998.1963.tb01616.x

[B12] Blanford WT (1863) Contributions to Indian malacology. No. IV. Descriptions of new land shells from Ava, and other parts of Burma. The Journal of the Asiatic Society of Bengal 32(4): 319–327

[B13] Bouchet P Rocroi JP Hausdorf B Kaim A Kano Y Nützel A Parkhaev P Schrödl M Strong EE (2017) Revised classification, nomenclator and typification of gastropod and monoplacophoran families. Malacologia 61(1–2): 1–526. 10.4002/040.061.0201

[B14] Budha PB Backeljau T (2017) First report of *Paraboysidialandourensis* (Pilsbry, 1915) from Nepal, with description of a putative new species and a note on *Bensonellaplicidens* (Benson, 1849) (Stylommatophora: family unclear). In: Budha PB (Ed.) Taxonomy of terrestrial molluscs of Nepal.PhD dissertation, Evolutionary Ecology Group, University of Antwerp, 189–201.

[B15] Burch JB Panha S (2002) [“2000”] The pupillid genus *Anauchen* in Thailand (Pulmonata: Stylommatophora). Walkerana 11(26): 239–248.

[B16] Burch JB Panha S Tongkerd P (2003) [“2002”] New taxa of Pupillidae (Pulmonata: Stylommatophora) from Thailand. Walkerana 13 (29/30): 129–187.

[B17] Cameron R (2016) Slugs and snails. William Collins, London [i–xvii], 1–508.

[B18] Chen Z-Y (2023) A new species of *Anauchen* Pilsbry, 1917 from Guangxi, China (Gastropoda: Stylommatophora: Hypselostomatidae). Revue Suisse de Zoologie 130(1): 89–92. 10.35929/RSZ.0090

[B19] Chen D-N Zhang W-H (2002) Zoogeographical analysis of the *Boysidia* (Ancey, 1881) in China, description of a new species (Pulmonata: Stylommatophora: Pupillidae). Acta Zootaxonomica Sinica 27(4): 690–698.

[B20] Chen D-N Liu Y-H Wu W-X (1995) Two new species of land snails from Shaanxi Province, China (Pulmonata: Stylommatophora: Pupillidae). Acta Zootaxonomica Sinica 20(3): 274–277.

[B21] Chen D-N Wu M Zhang G-Q (1999) Studies on the genus *Boysidia* Ancey from China with descriptions of six new species (Pulmonata: Stylommatophora: Pupillidae). Acta Zootaxonomica Sinica 24(2): 124–135.

[B22] Chen Y-X Tian M Fan B (2016) Terrestrial molluscs in Yunnan. Science Press, Beijing [i–ix], 1–259. [in Chinese]

[B23] Chen Z-Y Huang B Páll-Gergely B (2022) Exceptional among the exceptional: a new species of *Stenogyropsis* Möllendorff, 1899 (Eupulmonata: Camaenidae), with a review of the free last whorl in terrestrial gastropods. Journal of Molluscan Studies 88: eyac015. 10.1093/mollus/eyac015

[B24] Clements R (2009) *Hypselostomaelephas*. The IUCN Red List of Threatened Species 2009: e.T168177A6462664. [accessed on 30 October 2024]

[B25] Davison GWH (1995) The terrestrial Molluscan fauna of Temengor Forest Reserve, Hulu Perak, Malaysia. Malayan Nature Journal 48(3–4): 233–248.

[B26] Davison GWH Kiew R (1990) Survey of flora and fauna of limestone hills in Kelantan with recommendations for conservation. Report WWF Malaysia, WWF 3829 & MYS 161/89: 1–99.

[B27] Dumrongrojwattana P Assawawattagee S (2018) A new species of the genus *Boysidia* (Pulmonata: Pupillidae) from Northern Thailand. Sains Malaysiana 47(2): 215–219. 10.17576/jsm-2018-4702-01

[B28] Dumrongrojwattana P Tanmuangpak K (2020) The terrestrial microsnail genus *Aulacospira* Möllendorff, 1890 (Eupulmonata, Stylommatophora, Hypselostomatidae) in Thailand with key to Thai species. ZooKeys 980: 23–42. 10.3897/zookeys.980.54100 33192136 PMC7642177

[B29] Dumrongrojwattana P Wongkamhaeng K (2024) Two decades of microsnails (Gastropoda: Prosobranchia: Pulmonata) study in Thailand and ongoing research. In: Claude J, Kitana N (Eds) On the Edge of the Sixth Mass Extinction in Biodiversity Hotspots: Facts, needs, solutions and opportunities in Thailand and adjacent countries, Center of Learning Network for the Region (CLNR), Chulalongkorn University, 313–336.

[B30] Egorov RV (2013) Treasure of Russian shells. Supplement 3. Part 3: A review of the genera of the terrestrial pectinibranch molluscs (synopsis mainly based on published data). Littoriniformes: Liareidae, Pupinidae, Diplommatinidae, Alycaeidae, Cochlostomatidae. Roman Egorov, Moscow, 1–61.

[B31] Fang F Wang J Chen Y (2015) Studies on the genus *Boysidia* Ancey from China, with description of one new species (Pulmonata: Stylommatophora: Pupillidae). Sichuan Journal of Zoology 34(5): 688–694.

[B32] Fischer H Dautzenberg P (1904) Catalogue des mollusques terrestres et fluviatiles de ľIndo-Chine orientale cités jusqu’ à ce jour. In: Leroux E (Ed.) Mission Pavie Indo-Chine 1879–1895, Études diverses III Recherches sur l’histoire naturelle de l’Indo-Chine orientale.Leroux, E., Paris, 390–450. 10.5962/bhl.title.5099

[B33] Foon JK Marzuki M Bin E (2023) A checklist of land snails (Mollusca, Gastropoda) of Batu Caves, Selangor, Malaysia. Malayan Nature Journal 75(1): 133–148.

[B34] Foon JK Clements GR Liew TS (2017) Diversity and biogeography of land snails (Mollusca, Gastropoda) in the limestone hills of Perak, Peninsular Malaysia. ZooKeys 682: 1–94. 10.3897/zookeys.682.12999 PMC5523159 28769723

[B35] Fulton HC (1899) A list of the species of land Mollusca collected by Mr. W. Doherty in the Malay Archipelago; with descriptions of some supposed new species and varieties. Proceedings of the Malacological Society of London 3: 212–219 [pl. 11].

[B36] Godwin-Austen HH (1888) On some land-mollusks from Burmah, with descriptions of some new species. Proceedings of the Zoological Society of London 56(2): 240–245. 10.1111/j.1469-7998.1888.tb06701.x

[B37] Gojšina V Chen Z-Y Páll-Gergely B (2022) Notes on Hypselostomatidae (Gastropoda: Eupulmonata: Pupilloidea) from Myanmar. Archiv für Molluskenkunde 151(2): 131–139. 10.1127/arch.moll/151/131-139

[B38] Gojšina V Schilthuizen M Páll-Gergely B (2023) Three new hypselostomatid species (Gastropoda: Eupulmonata: Pupilloidea) from El Nido, the Philippines. Archiv für Molluskenkunde 152(1): 1–9. 10.1127/arch.moll/152/001-009

[B39] Gredler VM (1885) Zur Conchylien-Fauna von China. VIII. Stück. Bozen, 1–19.

[B40] Gredler VM (1901) Drei neue Land-Conchylien aus China. Nachrichtsblatt der Deutschen Malakozoologischen Gesellschaft 33(9–10): 150–153.

[B41] Gude GK (1914) The fauna of British India, including Ceylon and Burma. Mollusca-II. Trochomorphidae-Janellidae. Taylor & Francis, London [i–xii], 1–520. 10.5962/bhl.title.12891

[B42] Guo Y-H Zhou W-C Luo T-C (2006) One new species of the genus *Boysidia* (Pulmonata: Stylommatophora: Pupillidae). Acta Zootaxonomica Sinica 31(3): 541–543.

[B43] Haas F (1937) Neue und kritische Pupilliden. Archiv für Molluskenkunde 69 (1/2): 2–17.

[B44] Hanley SC Theobald W (1870–1876) Conchologia Indica, being illustrations of the land and freshwater shells of British India. L. Reeve & Co, London, Part 1: 1–10, pls 1–20 (1870); part 2: 11–18, pls 21–40 (1870); part 3: 19–28, pls 41–60 (1872); part 4: 29–34, pls 61–80 (1873); part 5: 35–40, pls 81–100 (1874); part 6: 41–48, pls 101–120 (1874); part 7: 49–56, pls 121–140 (1875); part 8: 57–65, i–xviii, pls 141–160 (1876).

[B45] Hemmen J Hemmen C (2001) Aktualisierte Liste der terrestrischen Gastropoden Thailands. Schriften zur Malakozoologie 18: 35–70.

[B46] Hoekstra P Schilthuizen M (2011) Phylogenetic relationships between isolated populations of the limestone dwelling microsnail *Gyliotrachelahungerfordiana* (Gastropoda: Vertiginidae). Journal of Zoological Systematics and Evolutionary Research 49(4): 266–272. 10.1111/j.1439-0469.2011.00623.x

[B47] Hwang C-C (2014) A new subspecies of land snail *Bensonellaplicidenslakainguta* (Gastropoda, Vertiginidae) from southern Taiwan. Bulletin of Malacology 37: 15–26.

[B48] Inkhavilay K Sutcharit C (2024) A new pulmonate microsnail species in the genus *Boysidia*, with remarks on *Bensonella* and *Krobylos* species (Pupilloidea: Hypselostomatidae) from northern Laos. Raffles Bulletin of Zoology 72: 438–449.

[B49] Inkhavilay K Sutcharit C Tongkerd P Panha S (2016) New species of micro snails from Laos (Pulmonata: Vertiginidae and Diapheridae). Journal of Conchology 42(4): 213–232.

[B50] Inkhavilay K Sutcharit C Bantaowong U Chanabun R Siriwut W Srisonchai R Pholyotha A Jirapatrasilp P Panha S (2019) Annotated checklist of the terrestrial molluscs from Laos (Mollusca, Gastropoda). ZooKeys 834: 1–166. 10.3897/zookeys.834.28800 31105437 PMC6495053

[B51] Iredale T (1937) A basic list of the land Mollusca of Australia. The Australian Zoologist 8(4): 287–333.

[B52] IUCN (2015) Annual Report of the Species Survival Commission and the Global Species Programme. IUCN Species Survival Commission, Cambridge, 1–351.

[B53] Jirapatrasilp P Panha S Sutcharit C Tongkerd P (2024) Four new species of micro land snail in the genus *Bensonella* Pilsbry & Vanatta, 1900 (Stylommatophora: Hypselostomatidae) from Thailand. Archiv für Molluskenkunde 153(1): 87–98. 10.1127/arch.moll/153/087-098

[B54] Jochum A Slapnik R Kampschulte M Martels G Heneka M Páll-Gergely B (2014) A review of the microgastropod genus *Systenostoma* Bavay & Dautzenberg, 1908 and a new subterranean species from China (Gastropoda, Pulmonata, Hypselostomatidae). ZooKeys 410: 23–40. 10.3897/zookeys.410.7488 PMC4042706 24899848

[B55] Kobelt W (1902) Das Tierreich. Eine Zusammenstellung und Kennzeichnung der rezenten Tierformen. Lieferung 16. Mollusca: Cyclophoridae. R. Friedländer und Sohn, Berlin [i–xxxix], 1–662.

[B56] Küster HC (1841–1855) Die Gattungen *Pupa*, *Megaspira*, *Balea* und *Tornatellina*. In Abbildungen nach der Natur mit Beschreibungen. In: Systematisches Conchylien-Cabinet von Martini und Chemnitz. Ersten Bandes, fünfzehnte Abtheilung. Bauer & Raspe, Nürnberg, 1–20, pls 1–2 (1841); 21–68, pls 3, 8–10 (1843); 69–76, pls 11–12 (1844); 77–88, pl. 13 (1845); 89–96, pls 4–5, 14 (1847); 97–128, pls 6, 15–16 (1850); 129–152, pls 7, 17–21 (1852); 153–194 (1855).

[B57] Laidlaw FF (1933) A list of the land and fresh-water Mollusca of the Malay Peninsula. Journal of the Malayan Branch of the Royal Asiatic Society 11(2,117): 211–234.

[B58] Luo T-C Chen D-N Zhang G-Q Zhang L Zhang L Li T-W (1998) Two new species of the terrestrial mollusks from China. Journal of the Guizhou Normal University (Natural Science) 16(2): 1–4.

[B59] Luo T-C Chen D-N Zhang G-Q (2000) Two new species of Pupillidae from China (Pulmonata: Stylommatophora: Pupillidae). Acta Zootaxonomica Sinica 25(2): 147–151.

[B60] Maassen WJM (1999) Some new records of Streptaxidae and Hypselostomatidae from Sumatra, Indonesia, with descriptions of three new species. Basteria 63(4–6): 121–126.

[B61] Maassen WJM (2001) A preliminary checklist of the non-marine molluscs of west-Malaysia. “A handlist”. De Kreukel, extra edition: 1–155 [pls 1–3].

[B62] Maassen WJM (2008) Remarks on a small collection of terrestrial molluscs from north-west Laos, with descriptions of three new species (Mollusca: Pulmonata: Streptaxidae, Vertiginidae). Basteria 72(4–6): 233–240.

[B63] Mabille J (1887a) Molluscorum Tonkinorum diagnoses. A. Masson, Meulan 1–18.

[B64] Mabille J (1887b) Sur quelques mollusques du Tonkin. Bulletins de la Société malacologique de France 4: 73–164 [pls 1–4].

[B65] Marzuki ME Liew TS Mohd-Azlan J (2021) Land snails and slugs of Bau limestone hills, Sarawak (Malaysia, Borneo), with the descriptions of 13 new species. ZooKeys 1035: 1–113. 10.3897/zookeys.1035.60843 33958931 PMC8096804

[B66] Möllendorff OF von (1890) Die Landschnecken-Fauna der Insel Cebu. Bericht über die Senckenbergische Naturforschende Gesellschaft, 1889/1890: 189–292 [pls 6–9]. 10.5962/bhl.title.13047

[B67] Möllendorff OF von (1891) On the land and freshwater shells of Perak. Proceedings of the Zoological Society of London 59(3): 330–348 [pl. 30]. 10.1111/j.1096-3642.1891.tb01757.x

[B68] Möllendorff OF von (1894) On a collection of land-shells from the Samui Islands, Gulf of Siam. Proceedings of the Zoological Society of London 62(1): 146–156 [pl. 16].

[B69] Möllendorff OF von (1897) Neue Landschnecken von Java. Nachrichtsblatt der Deutschen Malakozoologischen Gesellschaft 29(5–6, 7–8): 57–72, 89–97.

[B70] Möllendorff OF von (1900) Zur Binnenmollusken-Fauna Annams III. Nachrichtsblatt der Deutschen Malakozoologischen Gesellschaft 32(7–8): 117–121, 32(9–10): 129–139.

[B71] Möllendorff OF von (1901a) Zur Binnenmollusken-Fauna von Annam, IV. Nachrichtsblatt der Deutschen Malakozoologischen Gesellschaft 33(3–4): 45–50.

[B72] Möllendorff OF von (1901b) Diagnosen neuer von H. Fruhstorfer in Tongking gesammelter Landschnecken. Nachrichtsblatt der Deutschen Malakozoologischen Gesellschaft 33(5–6, 7–8): 65–81, 110–119.

[B73] Möllendorff OF von (1902) Binnenmollusken aus Hinterindien. 1. Landschnecken von Kelantan, Ostküste der Halbinsel Malacca. Nachrichtsblatt der Deutschen Malakozoologischen Gesellschaft 34(7–8): 135–149.

[B74] Morlet L (1886) Diagnoses de mollusques terrestres et fluviatiles du Tonkin. Mane et Noble, Paris, 1–7. 10.5962/bhl.title.14949

[B75] Motschoulsky V de (1845) Observations sur le Musée entolomogique de l`Université Impériale de Moscou. Bulletin de la Sociéte Impériale des Naturalistes de Moscou 18(4): 332–388 [pls 5–7].

[B76] Nevill G (1878) Hand List of Mollusca in the Indian Museum, Calcutta: Gastropoda. Pulmonata and Prosobranchia-Neurobranchia. Calcutta (Office of the Superintendent of Government Printing), [i–xv] 1–338. 10.5962/bhl.title.6561

[B77] Nicoll WM (1915) The trematode parasites of North Queensland. III. Parasites of fishes. Parasiology 8(1): 22–40. 10.1017/S0031182000010398

[B78] Odhner NH (1917) Results of Dr. E. Mjöbergs Swedish scientific expeditions to Australia 1910–1913. Mollusca. Kungliga Svenska Vetenskapsakademiens Handlingar 52(16): 1–115 [pls 1–3]. 10.5962/bhl.title.12985

[B79] Páll-Gergely B (2020) *Campolaemus* Pilsbry, 1892 is not a hypselostomatid, but a streptaxid (Gastropoda: Eupulmonata). Ruthenica 30(1): 69–73. 10.35885/ruthenica.2021.30(1).8

[B80] Páll-Gergely B (2023a) *Anauchen* (?) *kozari* n. sp., a new Hypselostomatid species from Laos (Gastropoda: Stylommatophora: Pupilloidea). Acta Phytopathologica et Entomologica Hungarica 58(1): 61–69. 10.1556/038.2023.00171

[B81] Páll-Gergely B (2023b) *Anaucheneotvosi* n. sp. from the “Shan-Siam Boundary” (Gastropoda: Stylommatophora: Hypselostomatidae). Journal of Conchology 44(5): 451–455.

[B82] Páll-Gergely B White TS (2023) Solving the mystery of the misunderstood *Bensonellaplicidens* (Benson, 1849) (Gastropoda: Stylommatophora: Hypselostomatidae). Journal of Natural History 56(45–48): 2011–2029. 10.1080/00222933.2022.2152750

[B83] Páll-Gergely B Hunyadi A Jochum A Asami T (2015) Seven new hypselostomatid species from China, including some of the world’s smallest land snails (Gastropoda, Pulmonata, Orthurethra). ZooKeys 523: 31–62. 10.3897/zookeys.523.6114 PMC4602296 26478698

[B84] Páll-Gergely B Muratov I V Asami T (2016) The family Plectopylidae (Gastropoda, Pulmonata) in Laos with the description of two new genera and a new species. ZooKeys 592: 1–26. 10.3897/zookeys.592.8118 PMC4926634 27408542

[B85] Páll-Gergely B Schilthuizen M Örstan A Auffenberg K (2019) A review of *Aulacospira* Möllendorff, 1890 and *Pseudostreptaxis* Möllendorff, 1890 in the Philippines (Gastropoda, Pupilloidea, Hypselostomatidae). ZooKeys 842: 67–83. 10.3897/zookeys.842.33052 31130806 PMC6517363

[B86] Páll-Gergely B Hunyadi A Grego J Reischütz A Buczkó K Vermeulen JJ (2020) *Clostophis* Benson, 1860, is not a monotypic diplommatinid but a speciose hypselostomatid (Gastropoda: Eupulmonata), with descriptions of six new species. Raffles Bulletin of Zoology 68: 350–368.

[B87] Páll-Gergely B Jochum A Vermeulen JJ Anker K Hunyadi A Örstan A Ábel S Dányi L Schilthuizen M (2022) The world’s tiniest land snails from Laos and Vietnam (Gastropoda, Pulmonata, Hypselostomatidae). Contributions to Zoology 91(1): 62–78. 10.1163/18759866-bja10025

[B88] Páll-Gergely B Hunyadi A Vermeulen JJ Grego J Sutcharit C Reischütz A Dumrongrojwattana P Botta-Dukát Z Örstan A Fekete J Jochum A (2023) Five times over: 42 new *Angustopila* species highlight Southeast Asia’s rich biodiversity (Gastropoda, Stylommatophora, Hypselostomatidae). ZooKeys 1147: 1–177. 10.3897/zookeys.1147.93824 37383941 PMC10297824

[B89] Panha S (1998a) [“1997”] Three new species of microsnails from southern Thailand (Pulmonata: Vertiginidae; Prosobranchia: Diplommatinidae). Malacological Review 30(1): 53–59.

[B90] Panha S (1998b) [“1997”] Three new species of *Hypselostoma* from Thailand (Pulmonata: Vertiginidae). Malacological Review 30(1): 61–69.

[B91] Panha S (1998c) [“1997”] A new species of *Gyliotrachela* from Thailand (Pulmonata: Vertiginidae). Malacological Review 30(1): 123–126.

[B92] Panha S Burch JB (2002a) [“1998–1999”] Two new genera of pupillid land snails from Thailand (Pulmonata: Pupillidae: Gastrocoptinae). Malacological Review 31/32: 143–153.

[B93] Panha S Burch JB (2002b) [“1998–1999”] First records and new species of *Boysidia* and *Sinoennea* from Thailand. Malacological Review 31/32: 117–122.

[B94] Panha S Burch JB (2002c) [“2001”] The Pupillid genus *Paraboysidia* in Thailand (Pulmonata: Stylommatophora). Walkerana 12(28): 77–94.

[B95] Panha S Burch JB (2002d) [“1999”] New taxa of Pupillidae (Pulmonata: Stylommatophora) from Thailand. Walkerana 10(24): 113–134.

[B96] Panha S Burch JB (2002e) New pupilloid land snails from Thailand (Pulmonata: Pupillidae). Natural History Journal of Chulalongkorn University 2(1): 21–24.

[B97] Panha S Burch JB (2008) [“2004–2005”] An introduction to the microsnails of Thailand. Malacological Review 37/38(1): 1–155.

[B98] Panha S Prateespasen R (2005) [“2000–2001”] A new gastrocoptine land snail from Thailand (Pulmonata: Stylommatophora: Pupillidae) Malacological Review 33/34: 99–103.

[B99] Panha S Tongkerd P Sucharit C Tumpeesuwan S Vongsombath C (2003) [“2002”] A new species of *Paraboysidia* (Pupillidae: Gastrocoptinae) from Laos. Walkerana 13(29/30): 123–128.

[B100] Panha S Tongkerd P Sutcharit C Burch JB (2004) New pupillid species from Thailand (Pulmonata: Pupillidae). Natural History Journal of Chulalongkorn University 4(2): 57–82.

[B101] Pfeiffer L (1849–1850) Neue Molluskengattungen. Zeitschrift für Malakozoologie 6(7, 8): 9–112 [(1849), 113–116 (1850)].

[B102] Pfeiffer L (1853) Monographia heliceorum viventium. Sistens descriptiones systematicas et criticas omnium huius familiae generum et specierum hodie cognitarum. Volumen tertium. F.A. Brockhaus, Lipsae [i–viii], 1–711.

[B103] Pfeiffer L (1859) Monographia Heliceorum viventium. Sistens descriptiones systematicas et criticas omnium huius familiae generum et specierum hodie cognitarum. Volumen quatrum. F.A. Brockhaus, Lipisae 1: 1–352 [January or February] [(2): 353–920, i–ix] [< November].

[B104] Pfeiffer L (1875–1876) Monographia Heliceorum viventium. Sistens descriptiones systematicas et criticas omnium huius familiae generum et specierum hodie cognitarum. Volumen septimum. F.A. Brockhaus, Lipsae, 1: 1–160 [(1875); (2): 161–320 (1875); (3): 321–480 (1875); (4): 481–674, i–x (1876)].

[B105] Pfeiffer L (1878–1881) Nomenclator heliceorum viventium. [Posthumous work edited by S. Clessin]. Cassel: Fischer. [See Bank et al. 2020, Archiv für Molluskenkunde 149(1): 80 for section dates. pp. 1–64, 1878; 65–192, 1879; 193–384, 1879–80; 385–617, 1881].

[B106] Pilsbry HA (1908) Two genera of land snails new to Japan and Korea. Conchological Magazine 2(8): 39–42.

[B107] Pilsbry HA (1915) A new Indian species of Pupillidae. The Nautilus 29(7): 73–74.

[B108] Pilsbry HA (1916–1918) Manual of Conchology. Second Series: Pulmonata. Vol. 24 Pupillidae (Gastrocoptinae). [(2) 24 (93): 1–112, plates 1–13 (1916); (2) 24 (94): 113–176, plates 14–29 (1917); (2) 24 (95): 177–256, plates 30–38 (1917); (2) 24 (96): 257–380, i–xii, plates 39–49 (1918).] Conchological Department, Academy of Natural Sciences Philadelphia, Philadelphia.

[B109] Pilsbry HA Vanatta EG (1900) A partial revision of the Pupae of the United States. Proceedings Academy of Natural Sciences Philadelphia 52: 582–611 [pls 22–23].

[B110] Pokryszko B (1996) The Gastrocoptinae of Australia (Gastropoda: Pulmonata: Pupilloidae): Systematics, distribution and origin. Invertebrate Systematics 10(5): 1085–1090. 10.1071/IT9961085

[B111] Pokryszko BM Auffenberg K Hlaváč JČ Naggs F (2009) Pupilloidea of Pakistan (Gastropoda: Pulmonata): Truncatellininae, Vertigininae, Gastrocoptinae, Pupillinae (in part). Annales Zoologici 59(4): 423–458. 10.3161/000345409X484847

[B112] Qian ZX Zhou WC [Eds] (2014) Illustrated handbook of common terrestrial mollusks in China. Zhejiang People’s Fine Arts Publishing House, Hangzhou, 1–228. [in Chinese]

[B113] Saenkamon O Wiboonpuech N Techama S Tanmuengpak K Dumrongrojwattana P (2022) Preliminary comparative study of shell, radula and genital system of terrestrial microsnails genus *Gyliotrachela* Tomlin, 1930 and *Anauchen* Pilsbry, 1917 from Eastern Thailand. Burapha Science Journal 27(2): 1055–1065.

[B114] Saurin E (1953) Coquilles nouvelles de l’Indochine. Journal de Conchyliologie 93(4): 113–120 [pl. 4].

[B115] Schileyko AA (1998) Treatise on recent terrestrial pulmonate molluscs. Part 2. Gastrocoptidae, Hypselostomatidae, Vertiginidae, Truncatellinidae, Pachnodidae, Enidae, Sagdidae. Ruthenica, Supplement 2: 129–262.

[B116] Schileyko AA (2011) Check-list of land pulmonate molluscs of Vietnam (Gastropoda: Stylommatophora). Ruthenica 21(1): 1–68.

[B117] Schilthuizen M Vermeulen JJ Davison GWH Gittenberger E (1999) Population structure in a snail species from isolated Malaysian limestone hills, inferred from ribosomal DNA sequences. Malacologia 41(1): 283–296.

[B118] Smith EA (1896) On a collection of land-shells from the Islands of Selayar, Jampea, and Kalao. Annals and Magazine of Natural History, Series 6, 18(104): 144–152 [pl. 10]. 10.1080/00222939608680425

[B119] Solem A (1981) Small land snails from Northern Australia, I: Species of *Gyliotrachela* Tomlin, 1930 (Mollusca: Pulmonata, Vertiginidae). Journal of the Malacological Society of Australia 5(1–2): 87–100. 10.1080/00852988.1981.10673940

[B120] Sowerby GB II (1876) Monograph of the genus Pupinidae. In: Reeve L (Ed.) Conchologia Iconica, or illustrations of the shells of molluscous animals. Vol. 20. London: L. Reeve, London [plates 1–10 + unpaginated text].

[B121] Stoliczka F (1871) Notes on terrestrial Mollusca from the neighbourhood of Moulmein (Tenasserim provinces), with descriptions of new species. The Journal of the Asiatic Society of Bengal, Part II, 40(2): 143–177.

[B122] Sutcharit C Thach P Chhuoy S Ngor PB Jeratthitikul E Siriwut W Srisonchai R Ng TH Pholyotha A Jirapatrasilp P Panha S (2020) Annotated checklist of the land snail fauna from southern Cambodia (Mollusca, Gastropoda). ZooKeys 948: 1–46. 10.3897/zookeys.948.51671 32765170 PMC7381483

[B123] Sutcharit C Ngor PB Páll-Gergely B Jeratthitikul E Siriwut W Srisonchai R Hui Ng T Jirapatrasilp P Panha S (2023) Notes on the hypselostomatid snails (Gastropoda: Heterobranchia) from limestone hills in Western Cambodia with a new record and a new species. Journal of Natural History 57(25–28): 1287–1303. 10.1080/00222933.2023.2223386

[B124] Sykes ER (1902) On a collection of land and fresh water shells from Kelantan, Malay Peninsula. The Journal of Malacology 9(2): 60–63.

[B125] Tanmuangpak K Dumrongrojwattana P (2022) *Gyliotrachelacultura*, a new species of terrestrial microsnail (Gastropoda: Eupulmonata: Vertiginidae) from Thailand. Raffles Bulletin of Zoology, 70: 407–416.

[B126] Thompson FG Dance SP (1983) Non-marine mollusks of Borneo. II Pulmonata: Pupillidae, Clausiliidae. III Prosobranchia: Hydrocenidae, Helicinidae. Bulletin of the Florida State Museum Biological Science 29(3): 101–152. 10.58782/flmnh.gwgk9850

[B127] Theobald W (1870) Descriptions of some new land shells from the Shan States and Pegu. The Journal of the Asiatic Society of Bengal Part II 39(4): 395–402.

[B128] Thompson FG Lee HG (1988) *Hypselostomaholimanae* new species, a pupillid land snail from Thailand. The Nautilus 102(2): 78–81. 10.5962/bhl.part.5183

[B129] Thompson FG Upatham S (1997) Vertiginid land snails from Thailand (Gastropoda, Pulmonata, Pupilloidea). Bulletin of the Florida Museum of Natural History 39(7): 221–245. 10.58782/flmnh.lecm3617

[B130] Tomlin JR le B (1930) Some preoccupied generic names. II. Proceedings of the Malacological Society of London 19(1): 22–24. 10.1093/oxfordjournals.mollus.a064001

[B131] Tongkerd P Lee T Panha S Burch JB O’Foighil D (2004) Molecular phylogeny of certain Thai gastrocoptine micro land snails (Stylommatophora: Pupillidae) inferred from mitochondrial and nuclear ribosomal DNA sequences. Journal of Molluscan Studies 70(2): 139–147. 10.1093/mollus/70.2.139

[B132] Tongkerd P Sutcharit C Panha S (2013) Two new species of micro land snails from two islands in the Andaman Sea (Prosobranchia: Diplommatinidae; Pulmonata: Pupillidae). Tropical Natural History 13(2): 65–76.

[B133] Tongkerd P Lwin N Páll-Gergely B Chanabun R Pholyotha A Prasankok P Seesamut T Siriwut W Srisonchai R Sutcharit C Panha S (2024) Contributions of a small collection of terrestrial microsnails (Pupilloidea, Hypselostomatidae) from Myanmar with description of three new species. ZooKeys 1195: 157–197. 10.3897/zookeys.1195.112112 38525356 PMC10958184

[B134] Van Benthem Jutting WSS (1949a) On a new species of *Gyliotrachela* from Celebes. Basteria 13(4): 64–65.

[B135] Van Benthem Jutting Jutting WSS (1949b) On a collection of non-marine Mollusca from Malaya in the Raffles Museum, Singapore, with an appendix on cave shells. Bulletin of the Raffles Museum 19: 50–77.

[B136] Van Benthem Jutting WSS (1950) The Malayan species of *Boysidia*, *Paraboysidia*, *Hypselostoma* and *Gyliotrachela* (Gastropoda, Pulmonata, Vertiginidae) with a catalogue of all the species hitherto described. Bulletin of the Raffles Museum 21: 5–47.

[B137] Van Benthem Jutting WSS (1952) Systematic studies on the non-marine Mollusca of the Indo-Australian Archipelago. III. Critical revision of the Javanese pulmonate land-snails of the families Ellobiidae to Limacidae, with an appendix on Helicarionidae. Treubia 21(2): 291–435.

[B138] Van Benthem Jutting WSS (1960) Some notes on land and freshwater Mollusca of Malaya. Basteria 24(1–2): 10–20.

[B139] Van Benthem Jutting WSS (1961) Additional new species and new localities of the family Vertiginidae and the genera *Oophana* and *Opisthostoma* from Malaya. Bulletin of the Raffles Museum 26: 34–48 [pls 8–14].

[B140] Van Benthem Jutting WSS (1962) Coquilles terrestres nouvelles de quelques collines calcaires du Cambodge et du Sud Vietnam. Journal de Conchyliologie 102(1): 3–15.

[B141] Vermeulen JJ Whitten T (1998) Fauna Malesiana. Guide to the land snails of Bali. W. Backhuys Publishers, Leiden [i–ix], 1–164.

[B142] Vermeulen JJ Le Cang P Quam Tarn T (2007) New species of terrestrial molluscs (Caenogastropoda, Pupinidae & Pulmonata, Vertiginidae) of the Hon Chong–Ha Tien limestone hills, Southern Vietnam. Basteria 71(1/3): 81–92.

[B143] Vermeulen JJ Luu H T Theary K Anker K (2019) New species of land snails (Mollusca: Gastropoda: Caenogastropoda and Pulmonata) of the Mekong Delta limestone hills (Cambodia, Vietnam). Folia Malacologica 27(1): 7–41. 10.12657/folmal.027.001

[B144] Wada S Chiba S (2013) The dual protection of a micro land snail against a micro predatory snail. PLoS ONE 8(1): e54123. 10.1371/journal.pone.0054123 PMC3541139 23326582

[B145] Yang H-F Zhang W-H Chen D-N (2012) A new species of the genus *Boysidia* (Ancey, 1881) from China (Pulmonata: Stylommatophora: Pupillidae). Acta Zootaxonomica Sinica 37(3): 550–551.

[B146] Zhang W-H Chen D-N Zhou W-C (2014) Two new species of the genus *Boysidia* from China, with a preliminary discussion of its geography (Pulmonata: Stylommatophora: Pupillidae). Zoological Systematics 39(4): 570–575. 10.11865/zs.20140410

[B147] Zilch A (1959–1960) Gastropoda. Teil 2. Euthyneura. In: Schindewolf OH (Ed.) Handbuch der Paläozoologie, 6 (2, 1): 1–200 [(1959); (2, 2): 201–400 (1959); (2, 3): 401–600 (1960); (2, 4): 601–834, I–XII (1960).] Borntraeger, Berlin.

[B148] Zilch A (1984) Die Typen und Typoide des Natur-Museums Senckenberg. Mollusca: Pupillacea: Chondrinidae: Gastrocoptinae, Hypselostomatidae, Aulacospirinae. Archiv für Molluskenkunde 115(1–3): 151–177.

